# Characterization of the definitive classical calpain family of vertebrates using phylogenetic, evolutionary and expression analyses

**DOI:** 10.1098/rsob.130219

**Published:** 2014-04-09

**Authors:** Daniel J. Macqueen, Alexander H. Wilcox

**Affiliations:** Institute of Biological and Environmental Sciences, University of Aberdeen, Tillydrone Avenue, Aberdeen AB24 2TZ, UK

**Keywords:** calpain superfamily, classical calpain, vertebrate evolution, novel calpain, CAPN17, expression classification

## Abstract

The calpains are a superfamily of proteases with extensive relevance to human health and welfare. Vast research attention is given to the vertebrate ‘classical’ subfamily, making it surprising that the evolutionary origins, distribution and relationships of these genes is poorly characterized. Consequently, there exists uncertainty about the conservation of gene family structure, function and expression that has been principally defined from work with mammals. Here, more than 200 vertebrate classical calpains were incorporated in phylogenetic analyses spanning an unprecedented range of taxa, including jawless and cartilaginous fish. We demonstrate that the common vertebrate ancestor had at least six classical calpains, including a single gene that gave rise to *CAPN11*, *1*, *2* and *8* in the early jawed fish lineage, plus *CAPN3*, *9*, *12*, *13* and a novel calpain gene, hereafter named *CAPN17*. We reveal that while all vertebrate classical calpains have been subject to persistent purifying selection during evolution, the degree and nature of selective pressure has often been lineage-dependent. The tissue expression of the complete classic calpain family was assessed in representative teleost fish, amphibians, reptiles and mammals. This highlighted systematic divergence in expression across vertebrate taxa, with most classic calpain genes from fish and amphibians having more extensive tissue distribution than in amniotes. Our data suggest that classical calpain functions have frequently diverged during vertebrate evolution and challenge the ongoing value of the established system of classifying calpains by expression.

## 2. Introduction

The calpains are an ancient superfamily of calcium-dependent cysteine proteases [1–3]. Unlike proteasomes and lysosomes, which degrade their substrates totally, calpains modify their targets by limited proteolysis, changing their functions without destroying them [1]. In doing so, they provide intricate regulation of diverse physiological processes, including gene expression, the cell cycle, intracellular signal transduction, cytoskeletal remodelling and apoptosis (reviewed in [4]). Considering its many vital physiological functions, the calpain system has major relevance to human health and welfare, including in terms of disease [4] and cancer [5].

In mammals, 15 calpain family members are recognized [2], many that are conserved more broadly [6–8]. Calpains are defined as classical or non-classical on the basis of conserved protein domains linked to CysPc, the papain-like protease domain that defines all calpains [1–3]. The classical calpains, which are specific to the animal lineage [3,9], include human CAPN1, 2, 3, 8, 9, 11, 12, 13 and 14, and have c2-like (C2L) and penta-EF-hand (PEF) domains, located C-terminal to CysPc [1–3].

Calpains are also classified by expression breadth in tissues, defining ‘ubiquitous’ and ‘tissue-specific’ types [1,2]. This system can be found in most calpain review articles from the past decade and is based on data established some time ago in mammals. A single published challenge to this system argued for a wider tissue expression for CAPN3 than its ‘muscle-specific’ [1,2] classification might suggest [10]. Such lines of investigation have received limited further attention, suggesting many of the defined ‘tissue-specific’ calpains may have more expression sites than widely realized.

Calpain research has also been limited by the fact that most studies have focused on mammals. In this sense, are the observed gene expression patterns and functions widely applicable? The evolutionary history of one calpain family member suggests this is a valid question. CAPN11—previously called µ/m-calpain in birds—acquired a highly restricted expression pattern during placental mammal evolution, whereas its ancestral function required extensive expression across tissues [7]. CAPN1 and 2 are direct daughter genes to CAPN11 [7], meaning CAPN11 holds a key position in the calpain family. In particular, the iconic ‘ubiquitous’ phenotypes of CAPN1 and 2 were inherited from CAPN11 [7]. Despite this, review articles invariably state that CAPN11 is ‘testis-specific’, and the importance of this calpain is widely unappreciated [11]. This is highly relevant for researchers of non-mammalian species (i.e. around 90% of vertebrate species), where CAPN11 has functional relevance on par with CAPN1 and 2 [7,11]. The extent to which other calpains have diverged in expression or function during vertebrate evolution is unknown.

The conservation of classical calpain expression and function across vertebrates can only be addressed in the light of a phylogeny spanning the major lineages, which is yet to be achieved. In fact, there are major gaps in our understanding of calpain evolution. For example, while it is known that CAPN1, 2, 8, 9 and 11 are ancestral to bony vertebrates [7,8], the evolutionary origins and distribution of CAPN12, 13 and 14 are unknown. Furthermore, key ancient taxa including jawless (e.g. lamprey) and cartilaginous fish (i.e. sharks and chimaeras) are unstudied in terms of calpain biology.

In the light of these outstanding issues, our main objective was to perform a comprehensive study of classical calpain evolution spanning the major vertebrate lineages. Then, as a proxy to understand the conservation of calpain functions during vertebrate evolution, a second aim was to determine the nature of selective constraints acting on each classical family member. With similar rationale, a final objective was to establish tissue-specific expression of all the classical calpain gene family members from distant vertebrate taxa, facilitating a general appraisal of the expression-based system for classifying calpains.

## 3. Material and methods

### 3.1. Bioinformatics

Ensembl (http://www.ensembl.org/index.html) genome assemblies formed the basis of exhaustive searches for vertebrate classical calpain sequences. The species covered are listed below with respect to their taxonomic position and the assembly version used. From jawless fish, searches included sea lamprey (*Petromyzon marinus*, assembly: Pmarinus_7.0). From bony vertebrates, our searches covered ray-finned fish, including spotted gar *Lepisosteus oculatus* (assembly: LepOcu1), which arose before teleosts, plus from the teleosts, Ostariophysi (zebrafish *Danio rerio,* assembly: Zv9), Paracanthopterygi (Atlantic cod *Gadus morhua*, assembly: gadMor1) and Acanthopterygii (platyfish *Xiphophorus maculatus*, assembly: Xipmac4.4.2; three-spined stickleback *Gasterosteus aculeatus*, assembly: BROADS1; tiger pufferfish *Takifugu rubripes*, assembly: FUGU4; tilapia, *Oreochromis niloticus,* assembly: Orenil1.0; medaka *Oryzias latipes* assembly: MEDAKA1). Our searches also covered lobe-finned fish including coelacanth *Latimeria chalumnae* (assembly: LatCha1) and tetrapods, namely amphibians (African clawed frog *Xenopus tropicalis,* assembly: JGI_4.2), reptiles (anole lizard *Anolis carolinensis*, assembly: AnoCar2.0; Chinese softshell turtle *Pelodiscus sinensis*, assembly: PelSin_1.0), birds (chicken *Gallus gallus*, assembly: Galgal4; turkey *Meleagris gallopavo*, assembly: UMD2; zebra finch *Taeniopygia guttata*, assembly: taeGut3.2.4) and mammals (platypus *Ornithorhynchus anatinus*, assembly: OANA5; Tasmanian devil *Sarcophilus harrisii*, assembly: DEVIL7.0; opossum *Monodelphis domestica*, assembly: BROADO5; pig *Sus scrofa*, assembly: Sscrofa10.2; human *Homo sapiens*, assembly: GRCh37; mouse *Mus musculus*, assembly: GRCm38).

Classical calpains obtained from all the above genomes except spotted gar (below) were identified by alignment to human CAPN1 and 2, facilitated by the EnsemblCompara GeneTrees paralogy function [12]. As the gar ‘pre-assembly’ currently lacks annotated gene models, classical calpains were identified by tBLASTn [13] searches using CAPN1, before GenScan [14] transcript predictions corresponding to positive hits were extracted.

Classical calpain sequences were acquired for cartilaginous fish using tBLASTn searches of transcriptome assemblies performed in BioEdit [15]. Transcriptome data were downloaded from SkateBase [16] for three species, including from the elasmobranchs: little skate *Leucoraja erinacea* (Rajiformes; NCBI accession for raw data: SRX036536); small-spotted catshark *Scyliorhinus canicula* (Carcharhiniformes; NCBI accession for raw data: SRX036537) and from the Holocephali: elephant shark *Callorhinchus milii* (Chimaeriformes; NCBI accession for raw data: SRX036538). We also accessed transcriptome data assembled from Roche 454 FLX reads, property of Dr Helen Dooley (University of Aberdeen). This included three species from the elasmobranchs; nurse shark *Ginglymostoma cirratum* and brownbanded bamboo shark *Chiloscyllium punctatum* (both Orectolobiformes), plus small-spotted catshark. Calpain sequences for cartilaginous fish are provided in the electronic supplementary material, figure S1*a*.

### 3.2. Phylogenetic analyses

Two hundred and nineteen classical calpain protein sequences were aligned using MAFFT v. 7 [17] via the GUIDANCE webserver [18], using the GUIDANCE algorithm [19] to gain statistical confidence for each aligned site. Sites were removed below a cut-off of 0.93 confidence [18]. Nine sequences were removed that were highly partial or contained tracts of highly divergent amino acids in normally conserved calpain regions. A high-confidence alignment of 210 sequences spanning 457 amino acid sites was used for phylogenetic analysis (electronic supplementary material, figure S1*b*). On average, each sequence in the alignment covered 96% of the sites, with 45 sequences being partial at the N′ or C′ terminus, missing 16% of the mean total number of sites. However, many of these sequences filled important taxonomic positions so warranted inclusion.

The alignment was uploaded to MEGA v. 5.0 [20] before the best-fitting amino acid substitution model was determined by maximum-likelihood (ML) (JTT [21] assuming among-site rate variation to follow a gamma distribution). The tree-building was performed in Beast v. 1.7 [22] specifying the best-fit substitution model, an uncorrelated lognormal relaxed molecular clock model [23], a Yule speciation prior [24] and a UPGMA starting tree. This method performs as well for phylogenetic reconstruction as unrooted methods, but has the advantage that the tree root can be statistically inferred [23]. This is important here, as the inclusion of distant outgroups (e.g. non-classical calpains) would limit the number of confidently aligned characters, diluting or saturating the phylogenetic signal and increasing the risk of branching artefacts. The Beast analysis was ran twice, with a Markov chain Monte Carlo (MCMC) chain length of 50 million generations, logging the relevant parameters every 1000 generations. The MCMC trace was scrutinized in Tracer v. 1.5 (http://tree.bio.ed.ac.uk/software/tracer/), demonstrating convergence. Effective sample size values were more than 200 for all parameters. A maximum clade credibility tree, based on one run, was created using TreeAnnotator v. 1.7 [22], discarding 10% of trees as burn-in.

A similar approach was used for an additional phylogenetic analysis using a subset of 22 sequences (done for reasons discussed in the Results and Discussion). The sequences were aligned as described above, leading to a confident alignment of 443 amino acids with near complete coverage across sequences (electronic supplementary material, figure S1*c*). The best-fitting substitution model was the same as the main alignment, and a Beast analysis was performed as described above. As a supporting method, we used the same data in unrooted ML analyses using the Phylogeny.fr webserver [25], using the best-fit substitution model, and an approximate-likelihood ratio test [26] to gain statistical support at each node.

### 3.3. Molecular evolutionary analyses

In-frame codon alignments were generated for nine classical calpain family members ancestral to jawed vertebrates. The GUIDANCE webserver was used, including a step to remove poorly aligned sites (0.93 cut-off [18]). The data was based on that used for phylogenetic analyses, with further data added to ensure that different vertebrate groups were represented by multiple species when possible. Codon alignments and their specified phylogenetic trees are provided in the electronic supplementary material, figure S1*d–l*.

Analyses based on non-synonymous (*d*_N_) and synonymous (*d*_S_) substitution rates were performed in HyPhy [27]. Phylogenetic trees for each calpain family member were generated using ML with amino acid data as described above [25]. For each classical calpain codon alignment, a local model was fit allowing every branch in the tree to have its own estimate of *d*_N_ and *d*_S_, achieved by crossing the MG94 codon model [28] with the best-fitting of 203 general time-reversible nucleotide substitution models. To establish variation in parameter estimates, the process was parametrically bootstrapped 500 times [27], providing standard deviation, which was propagated to *d*_N_/*d*_S_ ratios [29]. *d*_S_ > 2.5 was considered to represent mutational saturation, meaning some data were excluded.

While separate *d*_N_/*d*_S_ analyses were trialled for different classical calpain domains, the data were frequently of limited use, especially in the case of PEF and C2L, owing to the short length of aligned data, leading to large variance in *d*_S_ estimates. Thus, a caveat of this approach is that it cannot distinguish constraints acting across different calpain domains.

### 3.4. mRNA expression analyses

We used quantitative polymerase chain replication (qPCR) to determine the relative expression of every classical calpain family member in adult *D. rerio*, *X. laevis, A. carolinensis* and *S. scrofa*. A description of the samples is provided elsewhere [7]. This approach involved re-analysis of existing data for *CAPN11, 1* and *2* [7] as well as generation of novel data for *CAPN3*, *8*, *9*, *12*, *13, 14* and *17*. We designed 31 new primer pairs specific to any identified duplicate genes (electronic supplementary material, table S1). For most species, this was achieved by reference to aligned sequences used in the above analyses. For *X. laevis*, sequences orthologous to those described in *X. tropicalis* were identified by BLASTn [13] searches versus the NCBI nucleotide database. At least one primer in a pair was designed to span an exon–exon boundary.

qPCR was performed using an Mx3005P system (Agilent Technologies). Reactions (15 µl volume) included 5 µl first-strand cDNA (details of samples given elsewhere [7]), 7.5 µl Brilliant III ultra-fast SYBR green (Agilent Technologies) and 400 nM sense/antisense primers (electronic supplementary material, table S1). Cycling conditions were one cycle of 2 min at 95°C, followed by 40 cycles of 10 s at 95°C and 20 s at 65°C, followed by a DNA dissociation analysis in which a single peak was observed in all final assays. Samples were included within plates in duplicate, and each plate contained assays for the selected reference gene *rps13* (primers in [7]). We ran no-template controls, which never produced cycle threshold (Cq) values below 40 at a standardized threshold. Cq data for all genes were analysed in Genex v. 5.4.3 (MultiD Analyses AB). After normalization to *rps13*, expression data were placed on a relative scale for each species and presented in the style of a Northern dot blot [30]. This approach, while accurately defining the expression of each gene in each sample relative to *rps13*, lacks biological replication, ignores the effect of assay efficiency and lacks an exhaustive normalization strategy. Thus, it should be considered semi-quantitative.

### 3.5. *In silico* analyses of human calpain expression

We acquired expressed sequence tag (EST) profiles for each human classic calpain gene from NCBI Unigene (http://www.ncbi.nlm.nih.gov/UniGene/) covering 45 unique tissues. These data represent EST counts expressed relative to the total number of EST counts for each tissue. The mean total number of EST counts was 132 634 per human tissue (177 756, standard deviation, s.d.). Unigene identifiers were: *CAPN1* (911387; represented by 1205 ESTs), *CAPN2* (193910; represented by 1079 ESTs), *CAPN3* (151190; represented by 364 ESTs), *CAPN8* (179134; represented by 66 ESTs), *CAPN9* (713180; represented by 32 ESTs), *CAPN11* (165969; represented by 18 ESTs), *CAPN12* (5795592; represented by 63 ESTs), *CAPN13* (2730229; represented by 71 ESTs) and *CAPN14* (683218; represented by 17 ESTs). We took equivalent data for two established control housekeeping genes, beta-actin (*ACTB*, Unigene ID: 911387, represented by 25 742 ESTs) and eukaryotic translation elongation factor 1 alpha 1 (*EEF1A1*, Unigene ID: 1371506, represented by 27 011 ESTs).

## 4. Results and discussion

### 4.1. Phylogenetic analysis defines the complete vertebrate classical calpain family

**Figure 1.**
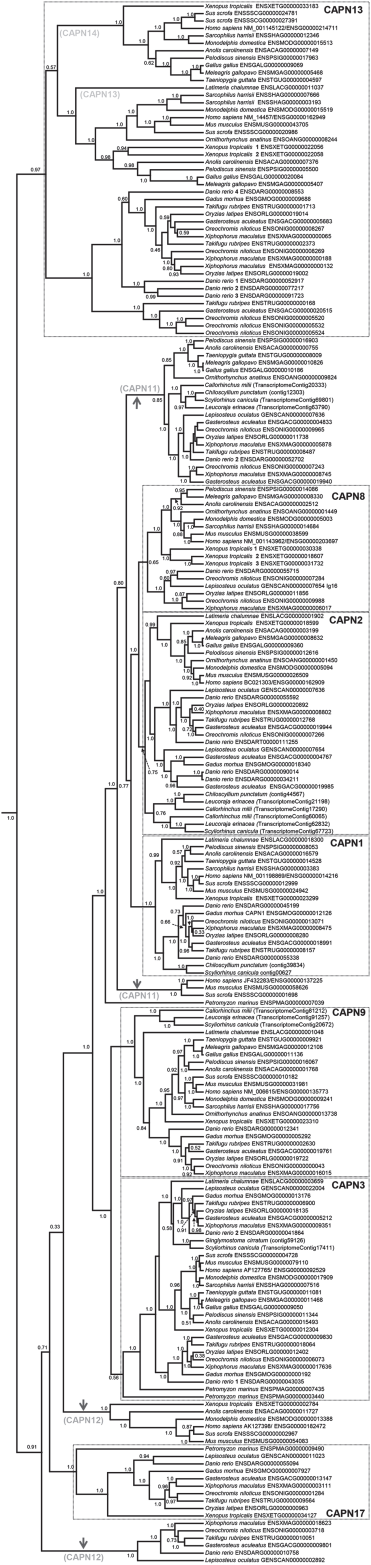
Bayesian phylogenetic analysis of 210 classical calpain sequences spanning vertebrate evolution. Branch lengths are relative to an uncalibrated timescale. Posterior probability values are included for every node. Boxed groups of sequences show vertebrate-wide classical calpain family members. Grey arrowheads highlight branching patterns hypothesized to be erroneous with associated text indicating the correct vertebrate-wide family member (details in main text and figure 3).

**Figure 2.**
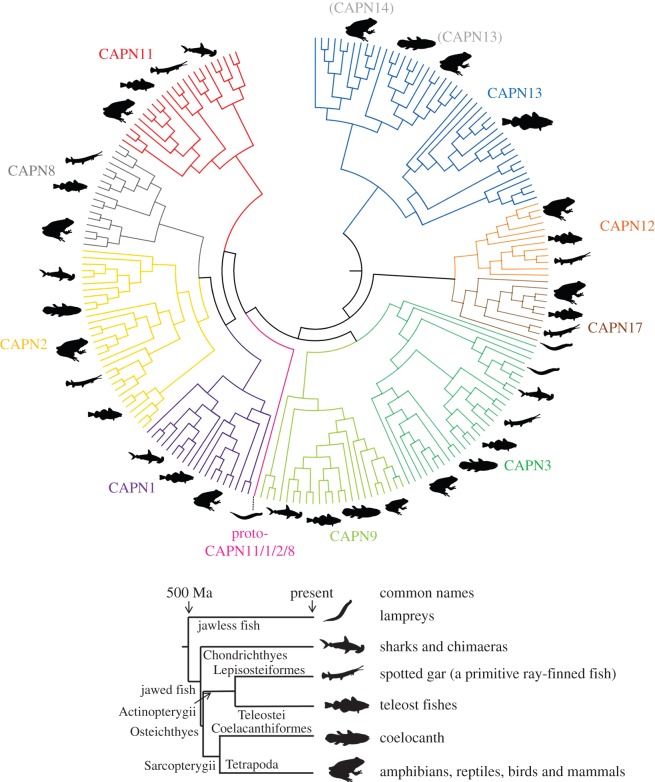
Consensus cladogram summarizing the relationships of classical calpain family members inferred from figure 1. This tree was corrected for two major branching errors (see main text and figure 3). Silhouettes represent the different vertebrate groups included, shown with respect to an accepted phylogeny and timeline.

We identified classical calpain sequences from an unprecedented range of vertebrate lineages, and more than 200 were used in a Bayesian phylogenetic analysis (figure 1). The results were consistent with several current hypotheses about classical calpain relationships [7,8,31]. However, two major branching patterns were inconsistent with previous data, or considered incorrect for other reasons (described below, see figure 1 legend). We thus also provide a consensus tree where these branching mistakes are corrected, allowing readers to rapidly absorb the phylogenetic structure of the definitive vertebrate classical calpain family according to our findings (figure 2).

#### 4.1.1. CAPN13: parent of all classical calpains

The root of the tree splits a well-supported group of sequences containing human CAPN13 and CAPN14 from all others (figure 1, maximal support). This group is not represented by lamprey or sharks/chimaeras (figures 1 and 2). Human CAPN14 is part of a group of tetrapod sequences that splits from a sister group that contains human CAPN13 along with other lobe-finned fish species (figures 1 and 2). The position of coelacanth as the earliest branch in this group suggests that a duplication event separating CAPN13 and 14 occurred during early lobe-finned fish evolution.

The CAPN13/14 group of lobe-finned fish then splits from a group of ray-finned fish sequences (figures 1 and 2). This suggests a single classical calpain (that went on to become CAPN13 and 14 in lobe-finned fish) was present in the jawed vertebrate ancestor. We use the name CAPN13, although CAPN14 is equally applicable, because our data cannot distinguish whether CAPN13 or CAPN14 is ancestral in lobe-finned fish.

The branching of CAPN13 sequences in ray-finned fish (figure 1) suggests that duplicate genes may have been retained from a genome duplication that occurred in the teleost ancestor [32]. Several teleost lineages also have additional CAPN13 copies branching closely in the tree (figure 1), often clustered on the same chromosome (not shown). We also observed that pig and Tasmanian devil have two CAPN14 copies that arose very recently (figure 1). *Xenopus* retains two *CAPN13* genes that are more divergent (figure 1) and may have arisen in an amphibian ancestor. Thus, many vertebrates retain multiple copies of CAPN13/14.

These findings extend limited past data on the evolutionary origins and distribution of CAPN13/CAPN14 based on single mammal species [1,2,33]. However, they agree with these past analyses, which incorporated non-classical calpains [1,2], in suggesting that CAPN13 is the ancestral classical calpain family member. The absence of CAPN13 in lamprey must either reflect gene loss or a lack of representation in the Ensembl genome assembly, as this species is present in more derived classical calpain groups (see below) and is evolutionarily more ancient than jawed vertebrates [34].

#### 4.1.2. CAPN17: a novel classical calpain most related to CAPN12

**Figure 3.**
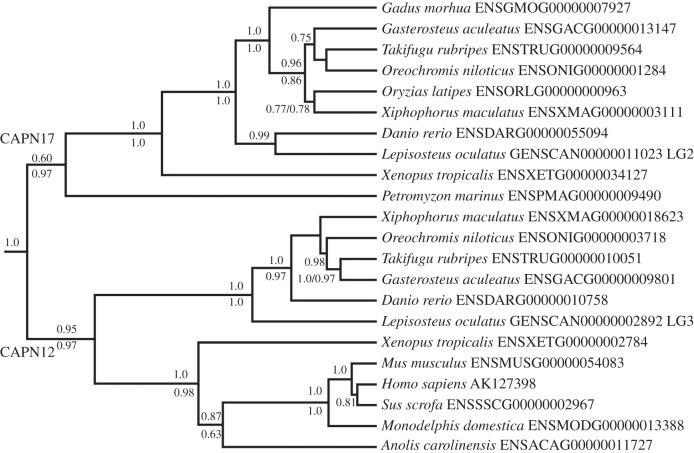
Bayesian phylogenetic analysis of classical calpain sequences that formed hypothesized erroneous groupings in figure 1 (see main text and figure 1 legend). Other details are as provided in the figure 1 legend, except that proportionate bootstrap support values are given from a supporting ML analysis (shown at each node under posterior probability values). Only node support values exceeding 0.50 are shown.

In the remaining tree, the three deepest branching arrangements separate three groups of sequences (figure 1). The most basal group comprises ray-finned fish only, the middle group comprises lamprey, amphibians, plus ray-finned fish and the final group comprises tetrapods only, including human CAPN12 (figure 1). The statistical support near the base of these groups was weak, suggesting the presence of a branching error. We thus performed independent phylogenetic analyses with the sequences involved (figure 3). The resulting trees split into two, rather than three groups (figure 3). The formerly separate ray-finned fish and tetrapod groups were affiliated as a single group that followed expected species relationships (figure 3). This grouping contains human CAPN12, suggesting that CAPN12 has been conserved across the evolution of bony vertebrates (figures 1–3). However, it was not represented by lamprey, sharks or chimaeras (figures 1 and 3). There was also no evidence for *CAPN12* gene duplicates in any represented lineage (figures 1 and 3). These data massively extend previous work on the CAPN12 phylogeny based on single mammal species [1,2,35].

The sister group to CAPN12 comprised the exact sequences that grouped together in the main analysis, with lamprey as the deepest branch (figure 3). This grouping is best explained by the presence of a novel classical calpain family member ancestral to all vertebrates, but that was lost in the common ancestor to terrestrial amniotes, where it is invariably absent. We name this new calpain as CAPN17, in line with existing patterns of nomenclature [1,2] (figure 2). CAPN17 was not represented by shark or chimaera, and there was no evidence for gene duplicates in any vertebrate lineage (figures 1 and 3).

#### 4.1.3. CAPN3 and 9 were present in the common vertebrate ancestor

Sequences branching internally to CAPN12/17 split into two major groups with maximal statistical support (figure 1). The first contained human CAPN9 and was represented by all major vertebrate lineages barring lamprey, with branching patterns largely following expected species relationships (figures 1 and 2). The second group included human CAPN3 and was represented by all the major vertebrate taxa (figures 1 and 2). The two most ancestral branches in this group were both lamprey (figure 1), a pattern inconsistent with the presence of a single vertebrate calpain family member. One possibility is that the common vertebrate ancestor had two *CAPN3* genes with one being lost at the base of jawed fish evolution. Alternatively, this branching pattern might be erroneous considering its weak statistical support (figure 1). For example, the more deep-branching lamprey sequence could represent a *CAPN9* gene. We observed two CAPN3 groups represented by the major teleost lineages (figure 1), consistent with the retention of duplicate copies from the genome duplication [32]. Overall, these data demonstrate that CAPN3 and CAPN9 were present in the common vertebrate ancestor, expanding past work considerably [7,8,31].

#### 4.1.4. Expansion of key classical calpains in jawed vertebrates

A large cluster of sequences branched internally to CAPN3 and 9 that included human CAPN1, 2, 8 and 11 (figures 1 and 2). A lamprey sequence received maximal support as the deepest branch in this group (figures 1 and 2). This suggests that the common vertebrate ancestor possessed a ‘protogene’ that went on to become *CAPN1*, *2*, *8* and *11*.

Branching internal to lamprey is a group of placental mammal CAPN11 sequences, which are separate from a group of CAPN11 sequences from other vertebrates (figure 1). The separation of these CAPN11 groups is a branching error that has been observed before [7]. A true CAPN11 grouping, supported by extensive phylogenetic and synteny data [7], is presented in the consensus tree (figure 2). As CAPN11 is present in shark/chimaera, the data suggest that CAPN11 was present in the jawed vertebrate ancestor (figures 1 and 2).

Splitting from the CAPN11 group, we observed a group of sequences including human CAPN1 and species covering the rest of jawed vertebrate evolution (figures 1 and 2). The branching patterns are largely consistent with expected species relationships, suggesting that *CAPN1* is also an ancestral gene among jawed vertebrates (figures 1 and 2).

Splitting from the CAPN1 group, we observe two further groups of sequences containing human CAPN2 and 8 (figures 1 and 2). The CAPN2 group is represented by the major jawed vertebrate taxa (figures 1 and 2). There is evidence for the presence of CAPN2 duplications in distinct vertebrate lineages. For example, shark and chimaera sequences split into two sister groups (figure 1) represented by lineages that separated more than 400 million years ago (Ma) [36]. The ray-finned fish sequences also split into two groups represented by spotted gar and teleost species (figure 1). This suggests a duplication event occurred before the separation of these lineages around 400 Ma [37]. Additionally, within these ray-finned fish CAPN2 groups, teleost sequences split into further groups (figure 1) suggesting additional duplicated copies have been retained from genome duplication in the teleost ancestor [32].

The CAPN8 grouping is represented by ray- and lobe-finned fish species, but not sharks or chimeras (figures 1 and 2). However, as the CAPN2 group contains shark/chimaera sequences (figures 1 and 2), the data require that the jawed vertebrate ancestor possessed CAPN8. Ray-finned fish CAPN8 sequences split into two groups (figure 1) consistent with a duplication event, potentially in the teleost ancestor [32]. *Xenopus* retains three *CAPN8* gene copies (figure 1).

### 4.2. A roadmap of classical calpain evolution

Our results suggest that the common vertebrate ancestor possessed at least six classical calpains: CAPN13, 12, 17, 3, 9 and ‘proto-CAPN11/1/2/8’. It was previously suggested that the ‘ubiquitous’ calpains CAPN1 and 2 arose by genome duplication in the vertebrate ancestor [31]. These events are now thought to have occurred in the common ancestor of jawed vertebrates and lamprey [38]. Thus, our data either require that CAPN11, 1, 2 and 8 arose during separate duplication events or that the well-supported branching position of the lamprey ‘proto-CAPN11/1/2/8’ sequence is erroneous.

### 4.3. Value of a comprehensive classical calpain phylogeny in vertebrates

The characterization of vertebrate calpains can represent a daunting task outside the mammal lineage. For example, if Ensembl databases are used as the start point for such an investigation, a researcher is typically faced with a large list of genes that are uncharacterized or frequently annotated incorrectly. Our study allows classical calpain sequences from Ensembl to be mapped to well-supported phylogenetic groups with defined nomenclature. If a study species is used that is unrepresented in our analyses, then BLAST searches should allow the relevant phylogenetic group to be identified by reference to a closely related included species.

### 4.4. Selective constraints acting during classical calpain evolution

**Figure 4.**
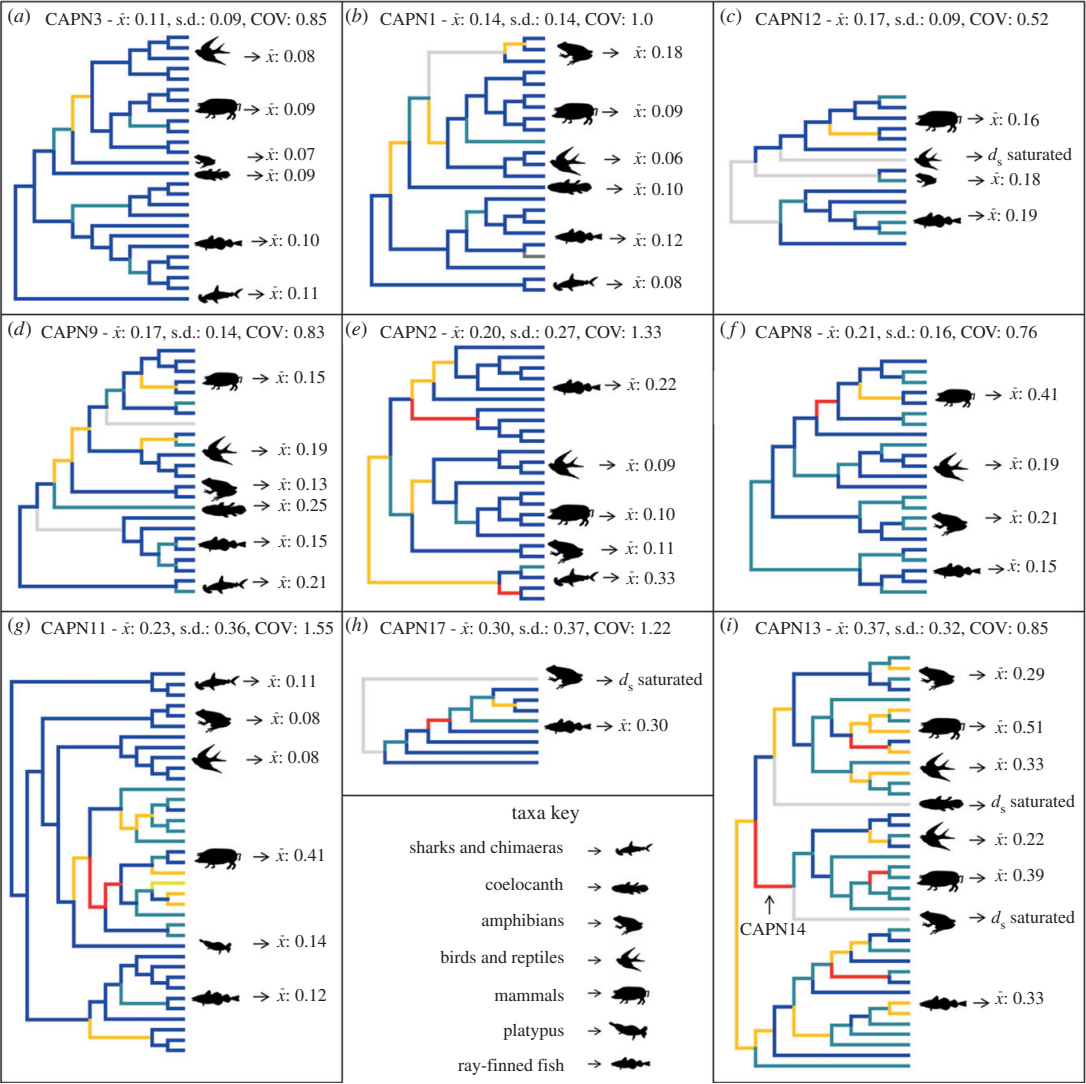
Reconstruction of selective constraints acting on classical calpains ancestral to jawed vertebrates. Different calpain family members are labelled (*a*)–(*i*) in ranked order by the overall strength of purifying selection acting during evolution. For each calpain, an empirical phylogeny is provided with the branches coloured according to five *d*_N_/*d*_S_ ranges: dark blue is 0–0.2; marine blue is 0.2–0.4; yellow is 0.4–1.0; red is >1.0; grey branches, *d*_S_ saturated. Summary statistics are shown including *d*_N_/*d*_S_ means (*x̄*), standard deviation (s.d.) and coefficient of variation (COV).

To gain insights into how natural selection has acted on different classical calpains, we established *d*_N_/*d*_S_ ratios at every branch in phylogenetic trees for family members ancestral to jawed vertebrates (figure 4). Purifying selection, i.e. selection to remove deleterious changes in protein sequence, has been the predominant force for all the classic calpains, with branch-averaged *d*_N_/*d*_S_ values ranging from 0.11 to 0.37 (figure 4*a–i*).

Classical calpain family members were ranked in terms of the strength of purifying selection acting during jawed vertebrate evolution as a whole (figure 4*a*–*i*). CAPN3 has been subject to the strongest level of purifying selection, followed by CAPN1, 12, 9, 2, 8, 11, 17 and 13 (figure 4*a*–*i*). As reported before [7], *d*_N_/*d*_S_ is consistently low for CAPN11 outside placental mammals, yet is much higher and more variable therein (figure 4*g*). Remarkably, if mammals are excluded, then CAPN11 has the lowest and least variable *d*_N_/*d*_S_ for the remaining vertebrates (mean *d*_N_/*d*_S_: 0.1, COV: 0.73). Therefore, for most jawed vertebrate species, CAPN11 has been subject to the strongest relative purifying selection during evolution, reiterating its extensive functional importance. In stark contrast, in placental mammals, CAPN11 is among the least conserved of classical calpains, along with CAPN13 (figure 4*g,i*). However, CAPN13 contrasts with CAPN11, because higher, more variable *d*_N_/*d*_S_ values are present across the vertebrate tree (figure 4*i*), suggesting CAPN13 has undergone persistent functional divergence during evolution.

Even the most conserved vertebrate classical calpains have branches where *d*_N_/*d*_S_ is notably higher than the background, suggesting periods of rapid protein evolution have occurred in a general backdrop of strong purifying selection (figure 4*a*–*i*). For example, for CAPN1, 2, 3 and 9, *d*_N_/*d*_S_ is elevated in deep branches of the lobe-finned fish lineage, either leading to tetrapods or amniotes (figure 4*a,b,e*). Thus, these classical calpains potentially diverged in ancestral functions during this period of evolution.

Interestingly, CAPN2 is not ranked among the most highly conserved vertebrate classical calpains (figure 4*e*). This is a consequence of several branches with high *d*_N_/*d*_S_ values outside the amniotes (figure 4*e*). Consistent with its known importance in mammals [1,2], we observed that CAPN2 of amniotes has been under consistently strong purifying selection on par with CAPN1 and 3 (figure 4*e*). These data point to distinct functional relevance for CAPN2 in different vertebrate taxa. Interestingly, the independent duplication of CAPN2 during cartilaginous and ray-finned fish evolution was followed in both cases by episodic rapid protein evolution for one of the duplicate copies (figure 4*e*). Instances of rapid CAPN8 evolution are also evident in some mammalian branches, suggesting periods of functional divergence have occurred, contrasting the relatively invariant strong purifying selection in other vertebrate groups (figure 4*f*).

Overall, these analyses suggest that episodes of functional divergence have been common during classical calpain evolution, although none more dramatic than already characterized for CAPN11 [7]. Nevertheless, the conservation of mammalian-defined classical calpain functions should not be taken for granted.

### 4.5. Diverse classical calpain mRNA expression across distant vertebrate taxa

**Figure 5.**
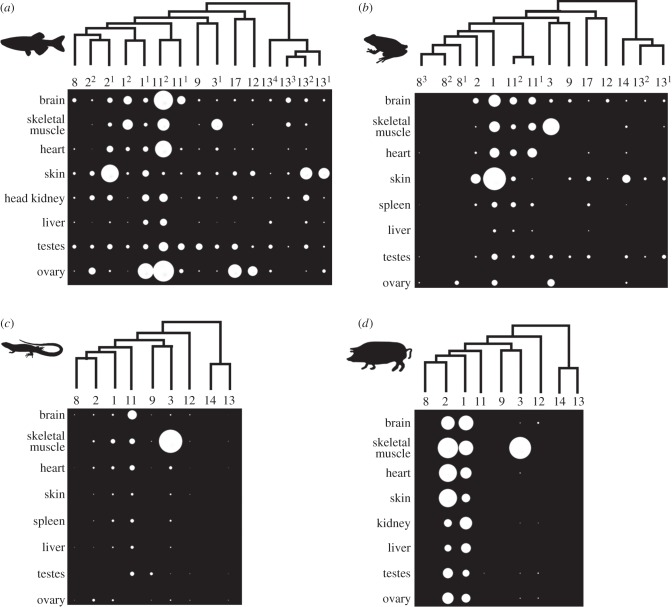
The mRNA expression of complete classical calpain gene systems in adult tissues of four vertebrate species. (*a*) Zebrafish, (*b*) African clawed frog, (*c*) anole lizard, (*d*) pig. Data are shown in the style of a Northern dot blot, but are derived from qPCR data normalized to the expression of *rps13*. Phylogenetic trees indicate the relationships of the classical calpain genes. When duplicated calpain family members are present, a superscript number is provided for each copy and referred to in figure 1 and the electronic supplementary material, table S1.

We profiled the mRNA expression of every classical calpain family member in multiple adult tissues from four vertebrate species separated by more than 300 Myr [34] (figure 5). Seven of the eight studied tissues were common across species. The data provide an unprecedented overview of classical calpain expression across vertebrate taxa. However, differences in expression may reflect ontogenic effects rather than true evolutionary divergence. Accordingly, we do not focus extensively on specific data, instead attempting to draw out broader evolutionary patterns.

Considering the data collectively, there is a striking difference in expression breadth across taxa (figure 5*a–d*). In zebrafish and to a lesser extent frog, all the classical calpains show a considerable degree of mRNA expression across tissues (figure 5*a,b*). Conversely, in anole lizard and pig, several classical calpain genes, including *CAPN8*, *13* and *14*, were barely detected in the same tissues (figure 5*c,d*). Comparing pig and anole lizard, a major apparent difference results from the known shift in *CAPN11* expression from broad to tissue-restricted [7] (figure 5*c,d*). Because these patterns generally track the evolutionary age of the lineages in question, we speculate that classical calpain expression breadth has decreased during the course of lobe-finned evolution, becoming more specialized during amniote and particularly mammalian evolution.

After excluding skeletal muscle (which skews comparisons owing to extensive variation in *CAPN3* abundance), *CAPN1* and *2* contribute 100% of the remaining mRNA expression observed in pig, with the equivalent figure being 31%, 60% and 18% in zebrafish, frog and lizard, respectively. *CAPN11* comprises 40%, 20% and 68% of the remaining mRNA expression in zebrafish, frog and anole lizard, respectively. Outside amniotes, *CAPN13, CAPN12* and *CAPN17* genes contribute a notable fraction of the total classical calpain mRNA in the same tissues (figure 5*a,b*): 22% in zebrafish and 10% in frog. Within its stated limitations, our data provide evidence for systematic divergence in the role of the classical calpain system during vertebrate evolution.

These findings suggest that classifying calpains by tissue expression has limited applicability across vertebrates. Ignoring the divergence in *CAPN11* expression, discussed before in this context [7], we note that *CAPN8* and *9* were not ‘gastrointestinal-tract-specific’ [1,2] in zebrafish, frog or anole lizard, whereas ‘hair-follicle-specific’ *CAPN12* [1,2] was not restricted to a single tissue in any species (figure 5*a–d*). While *CAPN3* mRNA was abundant in skeletal muscle in all species, it was not ‘skeletal muscle-specific’ [1,2], as notable levels of expression, sometimes on par with ‘ubiquitous’ calpains, were observed in other tissues for zebrafish, frog and lizard (figure 5*a–c*). Finally, *CAPN13* and *CAPN14* expression, classified as ‘ubiquitous’ [1,2], despite prior contrary reports [33], ranged from being extensive across tissues to undetectable (figure 5*a–d*).

### 4.6. Classical calpain mRNA expression in humans

**Figure 6.**
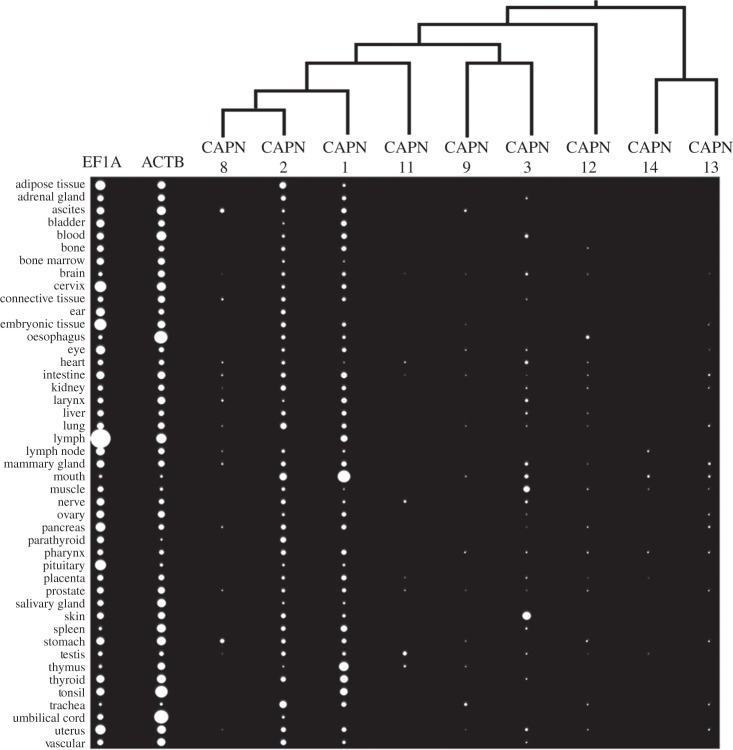
EST profiles approximating the mRNA transcript levels of classical calpain family members in human tissues. The size of each dot represents the number of ESTs representing separate family members divided by the total number of ESTs for each tissue. Phylogenetic relationships of different classical calpains are shown.

Next, we explored classical calpain gene tissue expression in humans exploiting EST profiles (figure 6). While this approach suffers from potential biases, it is reliable in a global sense, considering that 45 human tissues are represented by more than 100 000 ESTs on average. Two included control housekeeping genes had ubiquitous expression profiles, and all classical calpain genes were expressed in multiple tissues, with considerable variation in expression breadth (figure 6). As shown independently [10], *CAPN1* and *2* mRNA was not ubiquitous, being absent in a limited number of tissues, but nevertheless, it was considerably broader than the other classical calpains (figure 6). *CAPN3* was expressed in 26 of 45 tissues, inconsistent with a ‘muscle-specific’ classification [1,2] (figure 6). Interestingly, human *CAPN3* is more highly represented in skin than muscle ESTs (figure 6). While *CAPN8* and *CAPN9*, as expected, were expressed in tissues of the gastrointestinal tract [1,2], there was also expression outside this system (figure 6). *CAPN11* mRNA was not restricted to testis [1,2] (figure 6). *CAPN12* was expressed in 16 of 45 tissues, again inconsistent with its classification [1,2]. *CAPN13* and *14* were expressed in 16 and five of 45 tissues, respectively (figure 6).

### 4.7. Time to reconsider the classification of calpains by expression?

The expression data presented here and elsewhere [7,10] suggest that classifying calpains by tissue expression has limited applicability across taxa and is oversimplified for humans, where it should be most applicable. ‘Tissue-specific’ classical calpains are often expressed more widely than recognized at the mRNA level. We propose that the classification of classical calpain genes according to expression is reconsidered by the field.

### 4.8. Concluding remarks

This work represents the most extensive characterization of the classical calpain phylogeny performed to date. In addition to being a useful resource for future calpain researchers, the defined phylogenetic framework allowed us to systematically explore the evolutionary conservation of orthologous classical calpain functions/expression. Accordingly, we conclude that functional divergence and lineage-specific gene expansion are persistent features of classical calpain evolution in vertebrates. This has practical importance, considering that the same calpain genes may perform distinct roles in different lineages, questioning the general applicability of non-mammalian species (e.g. zebrafish) as human classical calpain models. Finally, we advocate for additional work to better understand the role of the complete classical calpain system of lower vertebrates, particularly for CAPN11, 12, 13 and 17, which are seemingly performing functions that may not even exist in mammals.

## Supplementary Material

Table S1 and Figure S1 - Table S1

## Data accessibility

Phylogenetic data: all sequence alignments and phylogenetic trees are provided in the electronic supplementary material. Ensembl identifiers/NCBI accession numbers for all sequences used in the study (except for cartilaginous fish) are provided in the electronic supplementary material. Sequences for cartilaginous fish are provided separately in the electronic supplementary material.
