# Supplementary material for: Characterization of the definitive classical calpain family of vertebrates using phylogenetic, evolutionary and expression analyses
Source: Open Biol. 2014 Apr 9;4(4):130219. doi: 10.1098/rsob.130219 (PMC4043111; doi:10.1098/rsob.130219)
Supplement: Table S1 and Figure S1 - Table S1 [file rsob130219supp1.pdf]

# Electronic supplementary material

**Table S1**. Details of all new primers used for tissue expression analyses.

| **Species** | ***Gene*** | **Ensembl identifier or accession number** | **Sense Primer** | **Sense Primer Tm (°C)** | **Antisense Primer** | **Antisense Primer Tm (°C)** | **Product Size** |
|---|---|---|---|---|---|---|---|
| *Danio rerio* | *CAPN3* **1** | ENSDARG00000043035 | GAGTTGAGCAGGGTAAACGAAAG | 60.27 | CCTCAAAGCACATCCAGAACTCC | 62.7 | 189 |
| | *CAPN8* | ENSDARG00000055715 | GGTAAAAGAAGCAGGGTTCCAGC | 63.55 | CAGAGCCACTGCAGGATATCC | 59.59 | 198 |
| | *CAPN9* | ENSDARG00000012341 | CCCTGATGTCCTGCTGCGTC | 63.48 | CTTCCTTTGCGGTGCGGTTC | 65.18 | 141 |
| | *CAPN12* | ENSDARG00000010758 | CACCAAAACTGGCAACATACAAG | 60.09 | GTCTTTTTCCAACACTGAGTTTACC | 58.78 | 196 |
| | *CAPN13* **1** | ENSDARG00000052917 | GGATTCCTGTAGAAGTATGATTGCC | 60.68 | GATGTAGGCCTGTTTTCTCTAATGC | 61.06 | 191 |
| | *CAPN13* **2** | ENSDARG00000077217 | GTTCCCAGTGCCCCGAAAATG | 66.09 | CCGCAAAAACACAGAAGCC | 59.05 | 114 |
| | *CAPN13* **3** | ENSDARG00000091723 | CCTGCCAGCTGAACAGTCATTCACC | 64.3 | CAGCATAGGACCCACACACCTTG | 64.11 | 209 |
| | *CAPN13* **4** | ENSDARG00000008553 | CCTCAACTTTCCAGCCCAATG | 61.96 | CCACTTCACCATACTTATCCGCA | 61.79 | 177 |
| | *CAPN17* | ENSDARG00000055094 | ACGGTAAAGAGCACTATTGGTCC | 59.62 | GCCAAAGACATCCAGAACTCTCCATC | 66.55 | 182 |
| | *CAPN3* | ENSACAG00000015493 | GCTGCCGTAACTATCCAGACAC | 59.16 | GCGTTCTTTCCTCCTATTCTTCTG | 61.08 | 143 |
| *Anolis carolinenis* | *CAPN8* | ENSACAG00000002512 | GTTGCAGAGTTCAAGATACTATGGATG | 61.04 | GAGAGTGAAACCTGCTTCCTTGAG | 61.57 | 135 |
| | *CAPN9* | ENSACAG00000001768 | GGATAAACTGAAAAAGTGGATTGG | 59.38 | TGCCTGGAAGACTCTGCTGG | 60.91 | 232 |
| | *CAPN12* | ENSACAG00000011727 | CCAAGACTGGATTAGACAAAGCAC | 60.12 | TCAAGAATGTCAAAGCAGTCATAGTC | 60 | 126 |
| | *CAPN13* | ENSACAG00000007376 | CCTGGTTTTGTGTGTTGTATGATTC | 60.57 | TCATAGTCACCCACTCATCTTCAG | 58.56 | 112 |
| | *CAPN14* | ENSACAG00000007149 | GCAGAACCTCATCAGGAATCAGTG | 62.71 | GCCCTACATCCTTCATTATCATCACC | 64.4 | 200 |
| | *CAPN3* | ENSSSCG00000004728 | CAAGCCCATCATCTTCGTTTC | 59.7 | GCTGGGCTCTGGCTTTGG | 61.24 | 130 |
| *Sus scrofa* | *CAPN8* | XM_003357653.2 | GCCCCTCCCGTTCCACC | 62.74 | CTCAACTCCCAGAAGCCCCT | 60.56 | 150 |
| | *CAPN9* | ENSSSCG00000010182 | CCTCCAAAGCCCACAGCCCC | 68.3 | GCCGCTGGTGTCCATTAGAG | 60.46 | 213 |
| | *CAPN12* | ENSSSCG00000002967 | GCTTCTCTGCCTCTTCCGCC | 63.74 | GCCACCTCCATCCACTGTCTG | 62.33 | 90 |
| | *CAPN13* | ENSSSCG00000020986 | GCCTGTCCTGATTCCTCTCCTC | 61.67 | CAGGTTCATCCACTCCATTTTTGTC | 63.91 | 103 |
| | *CAPN14* | XM_003125263 | CTTGTGGACTTCACTGGAGGGG | 63.19 | ACCCTCTCCTTCCCTGCGTG | 63.61 | 137 |
| | *CAPN3* | NM_001105269.1 | CAAGCCTATCATCTTTGTGTCGG | 61.57 | GCTTTTCTTCCTCGGTCTCTGTG | 62.27 | 165 |
| *Xenopus laevis* | *CAPN8* **2** | BC169897 | CGCATCCAATGACCTGGCTCT | 64.59 | CATTTTGAACAAGGTCTCCAGGC | 62.76 | 76 |
| | *CAPN8* **1** | NM_001085983 | CTACAGAAAACACCGTGAGACCT | 57.99 | CTGGCAGGGATAGTAGGTGTGTG | 61.26 | 88 |
| | *CAPN8* **3** | NM_001088543 | CTTCTGGAGTTCCGTATTCTGTGG | 62.11 | CATACAAGCGATAAAGCCATCAAAG | 62.68 | 219 |
| | *CAPN9* | BC154987 | GAGAAGAAAGCGGACATAAAAGAG | 59.19 | CTGCATGAAGATACCAATCCAGG | 61.28 | 343 |
| | *CAPN12* | BC154987 | CAAATAACATCCAAACACACCCATCTG | 65.85 | GTAAATCCAGCCGCTTCCAG | 59.91 | 237 |
| | *CAPN13* **1** | NM_001086019 | GAACCAGACAGGATTCGTTGATG | 61.06 | CTCCACTTAGGTAAACCCCTCTGC | 62.79 | 224 |
| | *CAPN13* **2** | NM_001093584 | CACCCAGTGCTTGACTTGATG | 58.67 | CAAGGGGATGCCGCTGGAC | 65.64 | 82 |
| | *CAPN14* | ENSXETG00000033183 | CACTTTCGGTTCTGGCGTTTC | 62.19 | CAGTTTGGCGTAGGCTTTCTCC | 63.07 | 150 |
| | *CAPN17* | BJ064215.1 | ATGATAGCCCAGCCTCCTCT | 59.81 | GATCCTTCAGCGCTGGTCTT | 60.11 | 104 |

**Figure S1**. Sequence data used in the study

(*a*) Novel cDNA sequences derived from cartilaginous fish transcriptome assemblies in complete nucleotide form (sense strand).

```
>Ginglymostoma cirratum_contig59126_CAPN3_Partial Coding Sequence
GGAAGTCTGAGAGAGAATGAGAGGAAGAGAGAGCTAAGGCCAATTCTGGAAAATTCAACAGACGTATCGTTCAACAAGTGGATGATAGTTTATTTGTCTTTTTTATTTCAATGTGGAAAACTGTGGAGAAGGCTTATTGTTATTA
TACAAAACAGCATTGAATCTTGTGAGGAACCTGGAACCAAAACTTTTTTGAAGTCCCTGGTGCCACTCGTGTTGTTTGGTGGAGCAGTCTGTCCAAATGATTTCTGCCTTTTACGTCAAGGAAACTGATTGAAATGTTTTGCTAG
TGTTTGTAGCTGAAAAAGTACTAACCTGTTTCCCTGTTTTATGAAACCAAATAGTTTCCTTCCTGCGTCTGCAGTCTGCATATCTTTCTCTTATTAACAGATCTCTTTTTTTACTGCACTGTTTTTGAAACATTGTGTTACTATG
CCTCATGTCATCAACTCGATGCAGGAATCACCACCAGTCCAGACCCAATCCAGAATGGCTGGCAAAGCTGCATTGTATCCTATCATCAATCGTAACAAGGATATTACCTGTGACAAGAGCGGGGTCTTTCAGCAGCTGAAGGACG
AGTGCCTGAGAGAAGGCGTCTTGTTTGAAGATGAGGAATTCCCTGCCAATGACTCCTCACTTTTTTACTCTGTGGGTATGCCCTTCAAGCTGGAATGGAAAAGACCCCCAGAAATCTGTGAGAGTCCAAAGTTCATCATTGGTGG
AGCCTCAAGAACTGATATCTGCCAAGGAGATTTGGGTGATTGCTGGTTCCTGGCTGCCATTGCATGTCTCACTCTTAACGAGAAGCTCCTGTACCGGGTTATCCCTCGTGATCAATATTTTACAGAGAACTATGCTGGAATCTTC
CACTTTCAGTTCTGGCGTTTCGGTGACTGGGTGGATGTCATTGTTGATGACCGAATTCCCACGTTCAACAACCAGTTGGTTTTTACCAAATCTGCTGAGCGAAATGAGTTTTGGAGTGCTCTATTGGAGAAGGCTTACGCCAAGT
TGCATGGATCATATGAAGCACTGAAAGGTGGCAATACCACAGAAGCCATGGAAGATTTTACTGGTGGTGTTACTGAATTCTTTGAGATAAAGGAAGCCCCCAAGGATCTGTTCAAGATCCTGAAGAAGGCAACAGAGAGGGGCTC
CCTTATGGGCTGTTCGATTGATTCTTTCGTACCTCAGCAGTTTGAAACACGAACAACCAGCGGTTTAGTCAGAGGACATGCCTATTCTGTAACCAGTGTGGATGAAGTCACTTACAAAGGTCAGACTGTGAAGTTGGTGCGTTTG
AGGAATCCATGGGGATCAGTGGAGTGGAATGGGTCCTGGAGTGATAAGGCCCGGGAGTGGTCCGAGATTGAGAAAACGCAAAAGCAACGACTTCAGCACCAAAAGAAAGAAGATGGAGAGTTCTGGATGTCTTTTGATGATTTTA
AGAGGAATTTCACAAAGCTGGAGATTTGTAACCTGACACCAGATGCGCTATGCGATGACAAGCTCCATAGGTGGACAGTATCTATCAATGAAGGACGGTGGGTAAAAGGCTGTTCAGCTGGGGGCTGCCGTAATTATCCAGACAC
ATTCTGGACGAATCCTCAGTACCGACTGAAACTCTGTGAGGAGGATGATGATCCTGATGTTGCCGAGGTCCTCTGCACCTTTGTTGTTGCTCTAATGCAGAAAAATCGTCGGAAGGAACGCAGAATGGGTGCCAACTTCCATACC
ATTGGATTTGCTATTTATGAGGT

>Scyliorhinus canicula_TranscriptomeContig67723_CAPN2 (cartilagenous fish specific paralog 2)
CAGCCCAGTGGCTGCAAACGTGTCCTGTTCGCTGTGGCAATGGGCGGTGGGAAGATTACGAAAACTCTCCCCACGCCTCGGTGCAGGTGCAGCCGCACCCAGCTCTGACAGTGCACCACAAGAACAAGGAGAGCAAGTCTCCCTG
CGGTGCGATCCCATCCAGGCCAGGGGCGAATCTGGTGCTGCTTTTCCAGCGACCCGTCCACACACTTTCACCCACTCGAGACTCCTGCAGCGCGGCGTCATTATGTCAGGGATGGCTTCGAGGATCAAGAAGCTGAGGGACCAGG
CGGCGGGTATCGGCTCCTACGGGCAGCCCGTCAAGTTCCTCCGGCAGGATTACGAGGCTCTCCGGCAGCAGTGCCTGGAGACCGGGAGCCTGTTCTGTGACGAGTCCTTCCCCGCCTGTCCTTCGGCGCTGGGCCACAATGAACT
GGGACCGGGCTCCTTCAAAACCCGGGATGTCGAGTGGAGGCGCCCAAAGGACCTGTGTGCGGACCCCAAATTTATTGTTGGAGAAGCTTCACGCACGGACATCTGCCAGGGAGCATTGGGTGACTGCTGGCTCTTGGCTGCCATT
GCGTCGTTGACCCTGAATAATGAGATTCTGTCCAGGGTTGTTCCCAGGGATCAAAACTTTGATTCTGATTATGCAGGAATCTTTCATTTCCAGTTCTGGCAGTACGGAGAATGGGTGGATGTGGTAATTGATGATAGGTTGCCCA
CAAAGGATGATCAGCTGATGTTTGTGCACTCTGCATCTGGGAATGAGTTCTGGAGTGCTCTGCTGGAGAAAGCCTATTCAAAGTTAAATGGATCGTATGAAGCGCTTTCTGGAGGATCCACCACTGAAGGGTTTGAAGATTTTAC
GGGAGGAATTTCGGAGTGGTACGAATTAGAAAAAGCACCAAGCAACCTCTTTAAGATCATCCGAAAGGCATTAAGAAGTGGGTCACTGCTTGGCTGCTCTATCAATATCACCAGTTCAGCAGAGACGGAGGCAATCACTTCACAG
AAGTTGGTCAAAGGTCACGCTTACTCCGTTACTGGAGCAGAAATAGTGAATTATCAAGGTGATGAGGAGAAACTGATCCGCATCCGGAACCCGTGGGGTCAAGTGGAGTGGACAGGAAGCTGGAGTGACAAAGGGGCTGAGTGGG
ATTGCATCCCTGATGAAGAAAGAGAGAGGCTGTGTAACGAATCAGATGATGGAGAATTCTGGATGTCATTTACCGATTTTCTCCAAAATTTCTCGCGAGTGGAAATCTGCAACCTCACTCCTGACAGCCTTAGCAGTGATGAAGT
TGGCAAGTGGAGCATGGTGATGTACAATGGGAGCTGGAGAACTGGATCTACTGCAGGGGGGGTGCCGAAACTATCCAAGGACTTTTTGGACAAATCCTCAATTTAAGATCCAGTTGGATGAAGCAGATGATGATCCTACTGATGAC
GATGATAAATGTAGCTTCCTGGCTGGTGTGATACAAAAGAATCGTAGATGTAAACGGAAAATGGGCGAGGACATGCGTACCATAGGCTTCGCTATCTATGAGGTCCCTGAAGAGTACCAAAACCAGACCAATGTTCAGCTGAAGA
GAGATTTTTTCATGACGAATGCATCCAGCGCAAGATCGGAAACCTTCATCAACCTGCGGGAGGTCATGAGCCGGCTTGCTCTTGAACCTGGGCAATATTTCATTATACCATCCACTTTCGAGCCAAATCAAAGTTGCGACTTTTT
GATTCGTATCTTTTCAGAAAAACAAAGTAACGTACAAATAATGGATACTATCGTTGATGCGAAACTGGAAGATGAGGAGGTCGAAGAAGAAGACATTAGTGACAGTTTCGAGAATATGTTTCAACATTTAGCAGGGCAGGATTCA
GAGATATCTGCCTTTGAGCTGCAAAGAATACTGAACAGAGTTGTAGCTAACCGTGATGACATTAAAACTGATGGTTTTAGTTTGGAAACCTGCCGAAATGTAATAAGCCTATTGGATAAAGATCGATCTGGGAAAATTGGACTAG
TGGAATTCAAGATATTTTGGATAAAATTACAGAAGATACTGAAAATCTTCAAGAAGGTGGATGCTGATGATTCTGGTACCATGAGCTCACATGAAATGCGAATGGCACTTGAAGAAGCAGGTTTCACTCTGAACAATACATTGTT
TCAGATCCTTGTTGCCCGATATGCAGATACTAATCTTCTCATTGACTTTGATAACTTTGTTGGCTCTTTGATCCGTCTGGAAACCATGTTCAGAATATTCAAACAATTAGACAAGGATGATGAGGGATATGCAGAGCTCAATATA
GGCCAGTGGTTAGGATTGGTACAAAACTGAAGTGGTGCTCAACTACAAGAAGCCAGACTTTGGTCAATCAACAAACACTTCCCACAGACTGATTTTAAGCTGTAAAGGAAAAATGGGAACTAACTTAGTCAGTTAGGTTATCATA
AACACATATGGAATGAGGGAATAGGTTTGTTTGTTTTCTTTTAGTGGATCGCTATCCTGTGCTTTAATCAATATTTTGGGTGGTTGAATAATTACCAGCTAGAACTTGCCTTCCCTGATATTTTAATCCATATATAGTGCATTGG
TTTGCATTTTGTAATTGTCA

>Scyliorhinus canicula_TranscriptomeContig17411_CAPN3 Complete Coding Sequence
TCTGCCGGCTGACATCATCTGCTCCTGGGTGAGGTAGTTGCTGCCTCAATAGCCCCTCTGTCTGCTGATTGAGATCGAAGTGAACATCTTTCGCTGGAAACCATGCCATACACTGCATCCGGGCATTTCTGTGACCGCCTGGTGC
GTGAGAGGCAGAGGAGGGAGGGGGAAGGGTCGATTCACAAGCCCATCAGATTTTATGAGCAGAACTTTGTGACGCTGAAGCAGGAATGCCTGCAGAAAAAGATTCTGTTTGAAGATGATACGTTTCCAGCCACTGTAGAGTCCCT
CGGGTATAAAGAACTGGGACAAAAGTCGACAAAAGTGAAGAACATATTGTGGAAAAGACCAAAGGAAATCTGTGACAATCCGAAATTCATCATTGGCGGAGCCTCAAGAACTGATATCTGTCAAGGAGATTTGGGTGACTGCTGG
TTCCTGGCTGCCATTGCTTGTCTAACTCTTAATGAGAAGCTGCTCTACCGGGTCATCCCTAGAGATCAATACTTTACAGAAAACTATGCTGGGATCTTCCATTTCCAGTTCTGGCGTTTCGGTGACTGGGTGGATGTCACTGTTG
```

ACGACCGAATCCCCACCTTCAACAACCAGTTGGTTTTCACTAAATCTGCTGAGAGGAATGAGTTCTGGAGTGCCTTGCTGGAGAAAGCCTATGCCAAGCTGAATGGATCCTATGAAGCGCTGAAGGGTGGCAACACCACGGAAGC
CATGGAAGATTTCACCGGTGGTGTTACTGAATTCTTTGAGATGAAGGAGGCCCCGAAGGATCTGTACAAGATCCTAAAGAAGGCAACAGACAGAGGTTCTCTAATGGGCTGTTCTATTGATTCTTTTGTACCTCTGCAGTTCGAG
ACACGGACTGCCAGTGGCCTGGTCAAGGGTCACGCGTACTCTGTGACCGGTGTGGATGAGGTCACCTATAAAAGTCAGAAGGTGAAATTACTGCGTTTGAGGAACCCATGGGGAGCAGTGGAGTGGAATGGGTCCTGGAGTGACA
AGGCCCGAGAATGGTCTGAGATTGAGAAAATACAAAAGCAGCGGCTACAACATCAAACGAAGGAAGATGGAGAGTTCTGGATGTCTTTTGATGATTTCAAGAGAAATTTCACAAAGCTGGAGATTTGTAACCTGACACCAGATGC
ACTGGGTGATGATAAGCTCCACAAGTGGACTGTGTCTATCAATGAAGGACGCTGGGTAAAAGGCTGTTCAGCTGGGGGCTGCCGCAACTATCCAGACACGTTCTGGACAAACCCTCAGTACCGATTGAAGCTCTGTGAGGAGGAT
GATGATCCTGATGTTTCTGAGGTGCTCTGCACATTTGTCGTCGCTCTGATGCAAAAGAATCGTCGGAAGGAGCGCAGGGTGGGCATCACATTCCATACCATTGGATTCGCTATTTATGAGGTACCCAAAGAGATGCAAAACAATA
AGCAACACCTACCCAAAGATTTCTTCCTCTACCAGGCATCGAAGTGCAGGTCCAAGTCATACATTAACCTACGGGAGGTATCGCAGCGTTTCAGCCTCTCCCCTGGGGAATATGTCATTGTCCCTTCCACATACGATCCACACCA
AGAAGGTGAATTCGTTCTCAGGGTCTTCTCAGAAAAACAGAACCTGTCAGAGGAAACTGAGAACCAGATCATAGCTGTCAACCCTGAGCTGCAGCCTTCATCAACAACAGCCAGGGAGGAGACTGAGGAGGACCTGCAGTTCCGT
CGTATTTTTGAACAGATTTCAGGAGATGATATGCAGATCAGTGCATCTGAAATGAGGAATATCTTAAACCGTGTTGTTAAAAAACACAAGATGAAAACGGAAGGGTTCACCCTCGAATCTTGTCGCAGTATGGTTGCTCTCATGG
ATACGGATGGGTCCGGAAAGCTTAATTTAATGGAGTTTAGGCATCTCTGGAACAAGATTAAGAAATGGCAGACAATCTTTATGGAATATGACAAAGATGCTTCTGGTTTGATTGACAGCTATGAAATGCGCACTGCTGTGAATGA
TGCAGGTTTCCAACTGAACAGTCAGCTCTATGAGATCATCTCCATGCGCTATGCTGATGAAAATATGAACCTGGACTTTGACAGTTTCATCTGTTGTATTGTGAGACTAGAGGGGATGTTCAGAGCTTTCCATGCCTTTGATAAA
GATGGAGATGGAATCATTAAACTCAGTGTTCTGGAGTGGTTGCAACTCACCTTGTATGCTTAAAAATCTCAGGACCTGGTGGCTTACATGCCTGGCTATTACCTCAAACTCAACGCCCAAACCTCAACAGCAGCATATGCAAAAT
CAACCTCTTCTACCTATGATGACATTCAATGCTACTTCAGGAATCCAGGTGTAAAGCCGCTTGTTGCACACTGTCAGATCAGCATGTCTTACTGCAAATGTGTGAATCTTTGCAATTGGGAACATGTGAAGCCAGGATAAAAGTA
CAAGTTTTCTTTATCGCTCTTGTATGATTTTACAATAAAGCCCTTACTCTAAACTCTGCCTTCTGGTGGGTAGATGCATTGCTTGGTATAGCTTTGAGTAGAAGATCCCAGGTTCAGTGTTTGGGATTAGTCATTACAGCTGGTT
TCAGTGCAGTGGGGAATTGACATCCACAGTTCTATTCAGTTGACCCTGCTGCATCAGTAAACATTTTAAAAATAAGGCCAAACTTGGTTGCTGCAGCCTGTGTCAAACCAGTTAATATTGGTGGATTGTTTGAGGAAACATCAAT
TAAATTAATTGCCAGCTTCCACA

>Scyliorhinus canicula_TranscriptomeContig69801_CAPN11 Partial Coding Sequence
GCCCACCAAGGATGGAGAGCTGATGTTCGTCCATTCAGCCGAAAACAATGAATTCTGGAGCGCGCTGCTGGAGAAGGCGTACGCCAAGCTCAGTGGATCGTATGAAGCCCTGTCCGGGGGTAGTACCACTGAAGGATTTGAAGAC
TTCACCGGAGGTGTGGCTGAGATGTATGACCTGGGCAGCGCTCCCCGTGATCTCTACACCATCATCCAGAAGGCAGTGGACAGAGGCTCACTGCTGGGATGCTCCATTGATATCACTGGTGCCTTTGATATGGAAGCCGTGACCT
TCAAGAAGCTGGTCAAGGGTCACGCCTACTCGGTGACAGGGGTCAAAGAGGTTGACTATCGGGGAAGGAAGGAGCGACTGATCAGGATCCGAAATCCCTGGGGCCAGGTGGAGTGGACCGGAGCTTGGAGTGACAATGCATCGGA
ATGGAATCAAATCGATGAGGATGAGCGTGATGGAATGGTCCAGATGGAAGATGGGGAATTCTGGATGGCATTCCAGGAGTTCCTGAAACAGTTTTCCCGGCTGGAGATCTGTAACCTGACTCCCGACACGCTGCAGGATGACATG
ATGAAGAAGTGGAACATGTCTGCGTTCAACGGCTCCTGGAGGAGGGGTAGTACTGCTGGGGGTTGCCGAAACCATGCAGCGACCTTTTGGATCAATCCGCAGTTTAAGATCACATTGGAGGAGGAGGATGATGACCCCGAGGATA
ACGAGATTGCCTGCAGCTTCCTGTGCGCTCTGATGCAGAAAGACCGGCGTCGACACCGCAAACACGGCCAGGATATGCACACCATCGGCTTCGCTGTGTATGAGGTTCCCGATGAGTATCGTGGTTGTCAGAACGTACATTTGAA
GAAGGATTTCTTCCTGACTCACAGTTCGTGCGCTCGCTCAGAAACGTTTATTAACCTGCGAGAGGTTAGCAACCGCATCAAACTTCCTCCTGGAGAGTACGTGATCGTGCCTTCAACCTTCGACCCCAGTCAGGAGGCCGACTTT
GTCCTGAGGGTTTTCACAGAGAAGCAGGCGGAGTCCGAGGAGCTGGATGACCCGATCAGTGCTGACCTGGAGGAAGAGGATGATCTGACTGAAGATGATGTTGATGAAGGTTTCAAGGCCATGTTCTCCCAGCTTGCTGGTCAGG
ACATGGAGATCTCTGTGTTTGAACTACGGACGATCCTCAATCGGGTGCTGTCCAGACATGGTGATTTAAAGACAGATGGATTCAGCTTGGATTCCTGTCGTGGGATGGTCAATCTCTTGGATAAAGATGGCAGTGGTCGCTTGGG
TGTGTTGGAGTTTCAAATCATGTGGAACAAGATTCGCAAGTGGCTGGGTATTTTCCGACAATATGACTTGGACAAGTCTGGCACCATGAGCTCGTATGAAATGAGATTGGCCCTGGAATCAGCAGGTTTTAAAATCAACAACCGA
ATCCACCAGCTGATAGTGGGGCGTTACGCTGAAAATGATGCTTTGGATTTCGATAACTTCATCTCCTGTCTGGTTAGATTGGAAGCAATGTTCAGATCATTCAAAGCCCTGGAGCAAGAGGATGGCACGGTGGAGATGAATATTG
TTGATTGGCTCAGTCTAACCATGTCCGGCTAGAAAATGATTGGAAGATGCTGAGATTGCAAATGCCTCTTTCCTGCTCATTAAGCTGACCTGCCTGCGGTCTGTGTGTAGTTTGATTGATAAAATGGTTTCAGCCGGCAATGAAG
ACAGTTTACTCAGAATTTAATGAAACTATGCATTTCCTCACCTGGGGATATTGTGCAGTGTGAGATCCAGATACTCAGACAACTGGTCCACCCAACCTCTGTGTCACCCCC

>Scyliorhinus canicula_TranscriptomeContig20672_CAPN9 Complete Coding Sequence
CGGTGGAGCAGCCTTGTAGCTAAGGAGAGGTGGAGTGTTGCAGGAAGGGCCAGGACATTCCCGCACTGACAGGTGAACTACACCTTGCTCGGGGACTCGCTCCTGATCTACCAAACATGTCTTATGTGTTCACCTCGGCCAGTGG
GGGCTCCCAGAAAAAGAAAGCGGTGAAAGCCCGCGGGACCCTCGGAGTGAAGGCGAGAAAACTCACCTATCAGGAGCTGAAGAACCAGTGTCTGAAACAGGGGGTTTTATATGAGGACCCGGAATTCCCAGCGGAAGAATCCTCC
CTGTTTTACAGCGAGAAGCCGGCAGTTGCCTTTGAGTGGAAACGGCCGAAAGAGATTTCGAAAGCCCCAAAATTTATTGTTGATGGAGCGTCGAGGACGGATATATGCCAAGGAGACCTGGGCGATTGCTGGTTTCTCGCTGCTG
TTGCTTCGCTGACTCTTAATCAGAAGATAATGGGCCAGGTTGTTCCTCTGGAACAGACCTTTGACCGCAATTATGCTGGAATCTTTTACTTTCGGTTTTGGCAATATAACGAGTGGGTGGAAGTCATAATTGATGATCGTTTGCC
AACCTTTAGAAATCGTCTAGTGTTTTTGCACTCTGCCAGCAATGACGAGTTCTGGAGTGCCCTATTGGAAAAGGCCTATGCTAAATTATATGGCGGCTATGAATCGCTGAAAGGAGGCAACACATTAGAGGCTATGGAAGACTTT
ACTGGTGGAATGGGAGAGACATTCAAGTTGAAAGAGCCACCCCCAAATATGTATACCCTCTTTCACAAGGCATTGAAACGAGGCTCCATGTTGGGATGTTCCATTGACATTACAAGTGTGACTGAGACAGAAGCTCGCACCAAAA
CAGGACTAGTCAAAGGACATGCATATTCTGTTACTGGTTTGGATCAGGTGAACTCCAGAGGTCGCCCAGTGCAGCTGATCCGTGTACGAAATCCCTGGGGTCAAGTAGAGTGGAATGGATCCTGGAGTGACGACTCTACTGAATG
GAAGACAGTGGATGCCGCTGATAATCGAAGGCTGAACATGGTCTCCAAAGATGATGGAGAGTTCTGGATGTCGTTTGATGATTTTAAGAAACATTTTGACACAGTAGAGATCTGTAACCTTACACCGGACTCCCTGGATACCGAT
GCCTTTCACAAATGGGAAGTGACAGTTTTTGAAGGGGCTTGGACCAAAGGCTGCACAGCTGGAGGCTGCAGGAACTTCCAAGACACTTTCTGGATCAACCCGCAGTATAAAGTACAGCTACTGGAGGCTGACGACGATCAAGAAG
ATGGCAAAGATGAATGCACCTTTATCGTAGCTCTCATGCAAAAAGACAGACGCAAATTGCGGAAGGAAGGAGCTAATTTGCTGACCGTTGGGTTTGCCATTTATGAGGCTGAAAATGACATTGATCACCTGTCGAAAGATTTCTT
CCAATTTCATGGTTCGAAAGCAAAGAGCAAGACGTACATCAACGTCCGGGAAAACTCCCAAAGGTTCAAACTTCCACCAGGACAATATGTGATTGTTCCGACTACTTTCCAACCTGAAGCAGAGGCTGAGTTCATTGTTCGATTT
ATTTCGGAGAAGAAAGCTTTAAGCCTTGAAATGGGGGATAAGATTGGTGCAGATCTACCAGAGCCGCCAAAATCAAAATCGAACCCCCAGGATGAAAGTCAGGAGGAAAGGCAATTCCGCTCGCTGTTTGAACAAATCTCAGGGA
AGGATCTGGAAATCAATGCTTATGAACTGCAAGATGTTTTAAATACTGTGTTCTCTAAACAAAAAGAAATGGCACCTGATGGTTTTGAAATTGAGACTTGTCGCAGTATCGTGTCTCTCTACCACAGGAACAACGCTGAAGGACT
TGGGTTTGACGAATTTAAGATCTTCTGGAATCGAATGAAGCAGTGGAAGAGTGCTTTCCTGTATTGCGACCATGACCGATCAGGGACCATGTCAGCTCATGAACTCCGAACTGCCGTCAAGAACTCAGGGTTTCAGATCAACAAC
CAGTTACTGCAGCTGCTGGTCCTCCGCTACACAGACGAGTACCTACAAATCGATTTTGATCACTTCCTCCGTTGCATGGTTCGATTGGAGACTTCATTCAGGCTGTTCAAGTCGCTTGATTCCCGGCAGACTGGTGAGGTGACAA

TGAACTTGCTGCAGTGGCTCCACATGACCATGGACATTTGAAGATGGAACTGGAGATCCTCGTACTTCAGTGCATTCTGCCATCACAAATATCTTGATTTCAGCATTAACAGGGGTACTATGTGTAAACAGAATTAGGTCTTATT
TGTTCACTTGTATTGTACTT

>Scyliorhinus canicula_contig00627_CAPN1 Partial Coding Sequence
CGGTGCGTCTGTTCTCTGATTGGTCGGCCCAGGGCGTGTGGTGGCAGTGATGTATTCTGGGGTGGCGGCTCGAGTCTACAAGCAAAGGCAGAAGCAGCTCGGGGCGGGCAACCACGATCAGGCCTTCAAGTACCTCGGGCAGGAC
TACGAGACGCTACGTGACGACTGCCTTGACCGAGGCGTTCAGTTTGTGGACCCCCACTTTCCCGTCGGGCCCTCCATCCTTGGGTTCAAGGAGCTGGGGCCCGGCACCTCAAAAACCTACGGCATCAGGTGGAAGAGGCCGAAGG
ACATATGTTCAAATCCCCAGTTCATCCTGGATGGGGCAACGCGCACTGACATCTGTCAGGGAGGTCTGGGTGACTGCTGGCTTCTTGCGGCCATCGCTTCGCTGACCCTGAACGAGAAGATCCTCCACCGGGTGGTGCCCCACGG
GCAGAGCTTCCAGCGGCAGTACTGCGGAATATTCCACTTCCAGTTCTGGCAGTTTGGTGAGTGGGTGGACGTGGTGATTGACGACCAGCTCCCGGTGAAGGACGGCAAGCTGGTCTTCGTCCATTCCGCCGCGCGCAACGAGTTC
TGGAGCGCGCTGCTGGAGAAAGCCTACGCCAAGTTAAGCGGTTGCTATGAAGCGTTGTCGGGCGGAAGCACGTCGGAAGGCTTCGAAGACTTCACGGGAGGGGTCACGGAAATGTACGACCTCGGCAAATCGCCGCCGGACCTTT
ACAGAATAATCCAGAAGGCTCTGGAACGTGGCTCTCTCATGGGATGCTCAATCGATATAACCAGTCAGTTCGACATGGAGGCCGTAACGTTCAAGAAGCTGGTCAAAGGTCACGCCTACTCCCTCACCGGCGCCCAGGAGGTGCC
GTGTCGCGGATCGCTGGTCAAACTGGTGCGACTGCGAAACCCCTGGGGGGAGGTGGAATGGACCGGAGCCTGGAGCGACAGCTCCCGTGAATGGAATCAAGTGGATGGGGCCCAGTGCAGCCAATTGCGGATCCAGCTGGAGGAT
GGCGAGTTTTGGATGTCCTTCGCTGACTTCCAAAGGGAATTTAACCGTCTGGAAATCTGCAACCTGACGCCCGACACCCTGAAATCGGACCAGGTCCGCAAATGGAGCACCTCGCTCTACGAGGGCGCCTGGAGGCGCGGGAGCA
CGGCGGGAGGCTGTCGCAACTACGCAGCTACCTATTGGATAAACCCGCAGTTCAAGATCCGCCTGAGGGAGGAAGACGACGACAGGAACGACGTCGAGTCGGGCTGCAGTTTCCTGGTGGCGCTGATGCAGAAAGACCGGCGCAA
GCTGCGCACCGCGGGCAAGGACATGGAGACCATCGGCTTCGCTGTCTACGAGGTCCCGGATGAGTGCGCCGGACAGTCTGCGGTCCACCTGAAGCGGGACTTCTTCCTGACGCACGGCTCGAGGGCCCGCTCCGAGATGTTCATC
AACCTGCGAGAGGTCAGCAGCCGCTTCAAGCTGCCCGCGGCAGTACATCATT

>Chiloscyllium punctatum_contig44567_CAPN2 (cartilaginous fish specific paralog 1) Complete Coding Sequence
ACACCACGACTGGGAGCCCCGCTCAGACCCGGAGCGGGACGGTGCCTGGTGCCTGGTGTCCGGTGTCCGGTGTCCGGGATGTCCGGGATGGCCCTGACCATTTCCAAGGCTCGGCAGAGACGGGCCGGGGTCGGCTCTAATGCAG
CAGCCAGCCCCTTCCTGAAGCAGGAATACGCGGTTCTCCGGGATCACTGTCTCCAAACAGGCTCCTTGTTCCAGGATCACAGCTTCCCGGCCAACGCCAGCTGCCTGGGATACAAGGAACTCGCCCCCAACTCCTACAAAACCAG
GGATGTCAGCTGGCTGAGACCCCGGGAGATCTGCTCCAACCCTCAATTTCTCATTGAAGGAGCCACTCGGACAGACATCTGTCAGGGAGCCCTGGGTGACTGTTGGCTCCTAGCTGCGATCGCCTCTCTGACCCTGAATGAAGAG
ATCCTCAGTCGGGTGGTACCCCCGGGACAATCCTTCGGGCCCGGCTATGCTGGAATCTTCCATTTCCAGTTCTGGCAGTTTGGGGAGTGGGTGGACGTGGTGGTTGATGACCGACTGCCCACCAAGGATGGAAAGCTGATGTTTG
TCCACTCGGCCGAGAGGAATGAGTTCTGGAGCGCTCTCCTGGAGAAGGCCTACGCCAAGTTGAATGGGAGTTATGAAGCTCTGTCGGGAGGCTCAACCACGGAGGGTTTTGAAGATTTTACTGGTGGGGTTTCGGAATGGTTTGA
GCTGAAGTCTGCTCCTCGGGACCTGTTCCACATCATCGAGAAAGCTCTGCAACGCGGATCATTGATGGGCTGCTCCATCGACATCACCAGTGCTGCTGAGACCGAGGCTGTTACCGGGCAGAAGTTGGTGAAGGGACACGCGTAC
TCACTGACTGGGGCCCAGCAGGTTGCGTTCCGAGGTACCCAGGTCCAGTTGGTCCGGATTCGGAATCCCTGGGGAGAGGTGGAATGGACCGGAGCCTGGAGTGACAATTCACCACATTGGAATGGGATATCGGCGGAGGACAGAA
ACCGACTCCGGAATCGGAACGAAGATGGGGAGTTTTGGATGGCTTTCCCTGAATTTCTGAGACATTATTCCCGCCTGGAGATTTGTAACCTGACCCCCGACGCCCTGAGCAGTCAGCAGGTCCTCAAGTGGAATTCGACTCTATT
CAGTGAGAGCTGGAGGAGGGGGTCCACTGCAGGGGGCTGTCGCAACTACCCAGGGACGTTCTGGATGAACCCTCAGTTTAAGATTACCTTGGAGGGCAGTGACCCTGACGACAGTGAGCAGTGCCACTTCCTGGTTGCTCTCATT
CAGAAGAATCGGAGGCAACAGAGGAGGATGGGGGAAGATATGCACACCATTGGATACGCCATCTACGATGTTCCCGATGAGTATAAGGGGGGTGTCCAATATCCATCTGCCAAAGAGTTACTTCCTGACCCACGGCTCCCGTGTAC
GTTCAGAGACTTTTATTAACCTGCGTGAAGTCTCGACTCGATTCCAACTGCCCCCTGGAGAATATCTCGTTGTGCCCTCCACCTTTGAACCTCACCAAAATGGAGATTTCGTTGTGAGGGTCTTTTCGGAGAATCGCTCGCAGAC
CGAGCCAATGGATATTGACCAGGTCCGCGCTGATCTCGATGATGAGGAGTCTGTCAACGAGAGAGATATCCCAGACAACTTCCGGACACTCTTCGAGAAACTGGCAGGACCGGGGAAGGAGATTTCTGCCTTTGGTTTGCAGAGA
ATCTTAAACAAAGTGGTGTCCAGCCGTTCCGACATTCAGACGGATGGATTTGGCCTGGAGACTTGCCGGAATATGGTCAACCTCCTGGATATGGATGGGAACGGGAAGCTGGGTTTGGTGGAGTTTAAGAAACTGTGGGACAAGA
TTCAGAAATTCCTGAAAATCTACAAGAAGAATGATCTGGACCAGTCTGGTACCATGAGCTCCAGTGAGATGCGAGTTGCTGTTGAGGAAGCTGGTTTCCACTTGAATAACCAGCTGACCCAGATCATTGTGGCCCGCTATTCCGA
GGTGGATAATCTCACCCTTGATTTCGATAACTTTGTTTCCTGTCTCGTTCGCCTGGAAGCTGTTTTCAAAATGTTTAATTCTCTGCCCAAGGATGGGGATGGTCTTGTCGAGCTCGGAATGCTGCAGTGGCTCACGTTGGTCATG
GGCTGATGTTGCTGGACCAGCAGTGGACGGGGAAGAGCAATTTCTACTGGGACAGTGCTCCATGAAGTGTCCATTCTGTCTGAGACATGCGCCCTGCTGTCTCTCTCTCTGGGATCTTGTTGCTCAGCCCTGCCTCAGGGCTGCT
TGTGTTTCTAGCAGTGGGTCATATCACCAGTGCACTGTGCTCTGATCTCTGCGGTGGATTAACTCTGACTCTGTTGCCTAATCCTCAGCATGGCTTTCTCTGAGGACCAGGTGGGTGGTTGTACCAACTTGGTCACTTTGTATTT
CTGAC

>Chiloscyllium punctatum_contig12303_CAPN11 Complete Coding Sequence
GAGCTGCGCAATGTTGATGGGCGTGTCAGGAAGACTTTACAAAGAGCGGTTAAAGCAGGAAGGAATGGGTTCTCATGAGAAGGCCCTGAGGTTCCTGAACCAGGATTACGAGGCTTTGAAGCAAGAATGTCTGGAGAATGGGACC
CTCTTTGAAGACCCACAGTTTCCAGCTGTCCCCACAAGCATCGGATTCAAGGAACTCGCTCCACACAGCTCCAAAACCCGCGGCATCATCTGGAAAAGACCACAGGAGATCGCAGCTGACCCACAGTTCATTCTGGCTGGAGCAT
CTCGAACTGATGTTTGTCAGGGAGGACTCGGTGACTGCTGGCTGTTGGCAGCCATTGCCTCACTGACTCTGAACGAGAAAGTGCTTCACCGCGTCGTGCCTCATGGTCAGAACTTCCAGGAGGAATACGCAGGAATTTTCCACTT
CCAGTTCTGGCAGTTTGGGGAGTGGGTGGACGTGGTGATTGATGACCGGCTGCCCACCAAGGACGGAGAGCTGGTGTTTGTCCACTCGGCCGAGAACAATGAATTCTGGAGCGGACTCCTGGAGAAGGCCTACGCCAAGTTGAAT
GGTTCCTATGAAGCCCTGTCAGGGGGCAGCACCACCGAGGGGTTTGAGGATTTTACCGGGGGGGTGGCAGAGATGTACGAACTCCGGAATGCTCCGCGGGATCTCTACACCATCATCAGGAAGGCCCTGGACAGAGGATCACTCC
TCGGCTGCTCCATCGACATCACCAGTGCTTTCGACATGGAAGCAGTGACCTTCAAGAAGCTGGTCAAAGGTCATGCCTACTCTGTGACTGGGGTCAAAGAGGTTGATTATCGGTCTCGGATGGAGAGATTGATCCGGATCCGGAA
TCCCTGGGGTCAAGTGGAATGGACTGGAGCATGGAGTGACAATTCCTCGGAGTGGAATTATATTGACCAGGAAGAGCGTGAAGGGATGGTGCAAATGGAAGATGGAGAGTTCTGGATGGCCTTCCCTGAATTCCTGCGGCAGTTC
TCGCGGTTGGAGGTGTGTAACCTGACACCGGACACACTGCAGGATGACCTGATGAAGAAGTGGAACATGGCCGTGTTTAATGGCTCCTGGCGCCGAGGGAGCACTGCAGGGGGCTGCCGTAACCATCCCGCTACGTTCTGGATCA
ATCCGCAGTTTAAAATCAAGTTGGAGGAAGAGGATGATGATCCTGATGATAATGAGCTTGCTTGCAGCTTCCTGTGTGCTCTGATGCAGAAGGATCGCCGTAGATACCGTCGCCATGGGCAGGACATGCACACCATCGGCTTTGC
CATCTACGAGGTTCCAGATGAGTATCGCGGTTGTCAGAACGTCCATTTGAAGAAGGATTTTTTCCTAACTCACAGCTCGTGTGCTCGATCCGAAACCTTCATTAATCTCCGTGAGGTCAGCAACCGCATCAAGCTGCCCCCTGGG
GAATATGTGATTGTCCCGTCAACCTTTGACCCCAGTAAGGAAGCTGACTTTGTCCTTCGGGTGTTCGCAGAGAAGAACGCTGACACAGAGGAGCTGGATGACCCAATCTCTGCAGACCTGGAAGACGAGGAGGAGATCACAGAGG
ATGATGTTGAAGAAAGTTTCAAGGCCATGTTCCGGCAGCTCGCGGGTGAGGATATGGAGATCAGTGTATTTGAACTGCGGACTATTCTCAATCGAGTTCTCTCCAAACACCGTGATTTGAAAACAGATGGGTTCAGTCTGGATTC

TTGCCGGGGAATGGTTAATCTCCTGGATAAAGATGGCAGTGGGCGTCTTGGTGTGGTGGAGTTTCAGATCATGTGGAATAAAATTCGCAAATGGCTGGGTATTTTCCGACAGTATGATCTGGATAAGTCAGGAACGATGAGTTCC
TATGAGATGCGCCTTGCCTTGGAGTCAGCAGGTTTTAAAATCAACAACCGAATCCACCAAGTGATCGTTGGCCGATACTCAGAGAATGATGTGCTGGACTTCGATAACTTCATCTCCTGCCTCGTCAAACTGGAGGCCATGTTCC
GGTCATTCAAAGCTCTGGAGCAGGAGGATGGGACAGTGGACTTGAATCTTGCTGAGTGGCTGAGTTTGACCATGTCGGGTTAGAAATCCAGATGAAGATGCCGAGATTGCGAGTGACTCCTTCCTGCTTTTTCACAGTGTATCAG
CCTTTGGCCTGTATGTAGCCTGTTTGATGAAACGGGCCTGGGGCGACTCTGAGGAGGCTGTTTACTCAGAGTCTGATGAAACTATGCAAACCCCTTCACCAGAGCACAGTGCAGTACCCGGCCCAGTCCCTCAGAAACTGACACT
CTTCAACTACTGCCCCATTCCCCTACCCACCCCTACCACCAACA

>Chiloscyllium punctatum_contig39834_CAPN1 Partial Coding Sequence
TCTTCCATTTCCAGTTCTGGCAGTTTGGTGAATGGGTGGAGGTGGTGATTGATGACCGACTCCCGGTGAAAGATGGAAAGCTGGTGTTTGTCCACTCGGCGGCTAGGAACGAGTTCTGGAGCGCCCTCCTGGAGAAAGCCTACGC
CAAATTAAATGGTTGTTATGAAGCCCTCTCGGGAGGTAGTACATCTGAAGGTTTTGAGGATTTCACGGGAGGAGTGACGGAAATGTATGACCTTCGTAAATCGCCCCATGACCTTTACCAAATTATCGAAAAGGCTCTGCAACGT
GGTTCACTCATGGGCTGCTCCATTGACATCACCAGCCAGTTCGACATGGAGGCTGTGACGTTCAAGAAACTGGTCAAAGGTCATGCTTACTCCCTCACTGGTGCCCGGGAGGTACGTGCTCGGGGAGGTTTGGTTAAACTGGTGC
GATTGCGAAATCCCTGGGGGGAGGTGGAGTGGACTGGAGCCTGGAGTGACAGGTCGAGTGAATGGAACAGTGTGGACTCCTCGGACTGCAGCTCGATGAGAATAAAGCTGGAGGACGGTGAGTTCTGGATGTCCTTTGGTGACTT
CCTCAGAGAATTCAACCGCTTGGAAATTTGCAACCTGACGCCGGATACTCTGAAATCGGAAACGATCTGCAATTGGAGCACCTCTCTGTACGAGGGCTCCTGGAGGCGTGGGAGCACCGCTGGAGGCTGCCGGAATTATCCAGCC
ACGTTCTGGATAAACCCCCAGTTTAAGATTGAACTGAAGGAAGAGGATGACGACCGGGTGGGAATGGAACCCGGCTGCAGCTTCCTGGTCGCCCTGATGCAGAAAGATCGTCGCAAACTCCGCACCGCGGGCAGGGATATGGAGA
CCATCGGCTTTGCCATCTATGAGGTCCCCCCTGAGTACGCCGGACGCTCAGCTGTTCACCTGAAGCGAGATTTTTTCCTGACACACGGCTCGCAAGCTCGTTCCGAAATGTTCATCAACCTCCGGGAAGTCAGTACCCGCTTCAA
GCTGCCTGCCGGAGAGTACATCATCGTCCCCTCGACCTTTGAACCCAACAAAGAGGCCGACTTCTGCCTGAGGGTGTTCTCTGAGAAGCGCTCACACTCTGAGGAACTCGATGATGAAATCAGTGCTGACCTGGACAGGAGGAGG
AAGTCCCTGAACACGCAATTGATGAAAAATTCAAGAGTCTCTTCAGGCAGTTGGCTGGAGAGGACATGCAGATCACAGTGACTGAGCTCCAGACAATCCTTAACCGTGTGGTAGGAAAACACCAAGACCTGAAGACCGATGGTTT
TAGTCTGGATTCCTGTCGATGTATGATCAATCTCATGGATCGAGACGGGAACGCCAGGCTGGGTCTGACCGAGTTCAACGTGTTGTGGAACAAGATTCGCAAGTGGCTGGGAGTTTTCCGACAGTTTGATCTGGATAAATCCGGC
AGCATGAACGCCTACGAGATGAGGCTAGCGTTGGAGTCTGCAGGGTTTAAACTGAACAATCACATGCACAATCTGCTCATCACACGCTACGCGGAGCCTGACCTTTCTGTCAACTTTGATAACTTCATCTGCTGTTTAATCCGCT
TGGAGAGCATGTACAGATCCTTTGTGATGCTGGATACAGACCGAGATGGTATTGTCACTTTTGATGTGTATCAGTGGCTGATGCTGACCATGTTTACTTGAGAACGATGCTGTGAAGTCTTGCCTCCTGCTGCTACGCCCCACAC
GCGTGCTTCCCACACCAAGTGCCTTCCACTGCTTAGACAGTGGACACTGGCCTTGTCCCGTGTCTTGCGAACGTCCCTCGCATCCCTCCCCCACCAGCAAACACCCC

>Callorhinchus milii_TranscriptomeContig20333_CAPN11 Complete Coding Sequence
GAGCAAAGGATGCTGACAGGAGTCTCCGCTCGGATACAGAAAGACCGTCAGAAACGTGAAGGGATGGGATCGAATGAGAGAGCGCTGCGTTTTCTGAACCAGGATTACGGGGTGCTGCGTGGGGAGTGTCTGGAGAACTGCTGCC
TCTTCGAAGACCCCCAGTTCCCCGCGATTGCCTCATCCATCGGCTTCAAGGAGCTTGGCCCGTACACCTCCAAAACCAGAGGCATCGTCTGGCAGAGACCCACTGAACTTGTTGCTGATCCACAATTCATCACCGATGGAGCAAG
CAGGACTGATGTGTGCCAGGGAGCTTTGGGTGACTGCTGGCTCCTGGCTGCCATCGCGTCTCTGACCCTGAACAATGAGGTGCTGCACCGGGTCGTGCCACATGGACAGAGCTTCCAGGAGGAATATGCTGGAATATTCCATTTC
CAGTTCTGGCAGTACGGGGAGTGGGTGGATGTGGTGATTGACGACCAGCTGCCCACCAAGGATGGAGAACTGGTGTTCGTCCACTCGGCCGAAAACAACGAGTTCTGGAGCGCACTGGTGGAGAAAGCTTATGCCAAGCTGAACG
GCTCGTACGAGGCGCTGTCGGGCGGCAGCACCACGGAAGGGTTTGAGGATTTCACTGGCGGAGTGGCTGAGATGTATCAACTGAACAAAGCGCCACGAGACCTGTACTCCGTCATCAGGAAAGCCGTGGAGCGAGGCTCACTGCT
CGGCTGCTCCATTGATATCACTAGCGCCCACGACCAAGAAGCCGTGACCTTTAAGAAGTTGGTGAAAGGTCACGCTTACTCAGTCACCGGGGTGAAAGAGGTGGATTACCGCGGGCGCCAGGTCAGACTGATCCGAATCCGGAAC
CCATGGGGACAAGTGGAGTGGACAGGATCGTGGAGTGACGGCTCCTCAGAATGGGATCACATTGGCGACGATGAGAGGGATGGAATGGTGAACAAGGAGGACGGAGAGTTCTGGATGGATTTCTCTGAGTTCCTGCGGCAGTTCT
CGAGGCTGGAGATCTGTAACCTGTCTCCGGACGCGCTGCAGATGGAGGAGGTCAGGAAGTGGCACACGGCTGTGTATGACGGGTGTTGGAGACGGGGCAGCACCGCTGGCGGCTGCCGCAACAACCCGGCCACGTTCTGGATTAA
CCCACAGTTTAAGATCACTCTGTCGGAGGAGGATGATGACCCGGAAGATGAGGAGTTGGCCTGTAGCTTCCTGTGCGCGCTGATGCAGAAAGACCGTCGTCGTTTTCGGCGCCAGGGGCAGGACATGCACACCATCGGCTTCGCC
ATCTACGAGGTCCCAGAGGAGTATCACGGCTGTCAGAACGTGCACCTGAAGAAGGATTTCTTCTTGACCCACGGGTCCTGTGCTCGCTCCGAGACCTTCATCAACTTGCGTGAGGTCAGCTCCCGTATCAGGCTGCCCCCTGGAG
AGTACATCATCGTCCCCTCTACCTTCGAGCCCCGGCAGGAAGCTGACTTCGTCCTGAGGGTCTTCACTGAGAAACAAGCTGAGTCTGAGGAGCTGGATGATGAGATCACAGCAGATCTGGAAGATGAGCCAGAGATGTCGGAAGA
TGATGTGGACGAGAATTTCAAAAACATGTTTCGTCAACTGGCTGGAGAGGACATGGAGATCAGTGTCTTTGAACTGAAGACGATCCTAAACCGGGTTCTGTCCCGACATCACGATCTGAAGACTGACGGCTTCTGTCTGGACTCG
TGCCGAACAATGGTTAATTTGATGGACAAGGATGGCAGTGCTCGGCTCGGAATCCTGGAGTTTCAGGTCCTTTGGAACAAGATTCGCAAATGGCTGGGCATCTTCCGACAGTTTGACCTGGACAAGTCTGGAACAATGAGCACTT
ACGAAATGAGACTTGCCCTGGAAGCTTCAGGATTTAAGCTGAACAATCGGATCCTCCAGTCCATCGTCGGGCGCTACGCGGACAATGATACCATCGACTTTGACAACTTCATTGGTGCCCTAGTCAAACTGGAGGTCATGTTCAG
ATCCTTCAAAGCTCTGGAGAAGGAGGATGGCACAATAGAGATGAACATCATGGAGTGGCTTACGCTGACCATGTCCAGCTAGAAAACAGCTTCAACAACCAGACACAGCGAGCAAGTGCCTCTGACTCCAGTGTGATCCTCACTC
TGTACTCTATACAGCCGCTCCCCCTTGATATCCAACCACTACCTTCCTCCTGTATACAATCTCTCTCTCCTGTATACAAGCTTTCCCCTCCGGTATACAGCCTCCCCCTCCGGTATACAGCCTCCCCCTCCGGTATACGGCCTCC
CCCACCTGTATACGGCCTTTCCCTCCTGTATATGGCCTCTTCCTCCTGTATGTAGCCTCTCCCCCATTGTATATGGCCTCTAACCACAGGAATGGGACTCTCACTCCTCCTGTAAATGCCCTCTAGCCACTCCTCCTGTATATGG
CCTCTTGTGTATATGGCCTCTCTTCTCTCTCCTGTATATGACCTCACCACCCCCCCACCCCATAATTGATCTTTTCCCCTATTTTTCATCTCTTCCCCCTACTATTTTGGACTTAACCCCCCTTCATTTTGACCTCTCTCCTGCT
CCAATAGTAACCATGATGACCACAGCCTGTTTTAGCATGTACGATGAATGGCTTCGCTGGCATAAGATGCAATAAACTTTATAAACAATGCATCCTCCCGAG

>Callorhinchus milii_TranscriptomeContig17290_CAPN2 (cartilagenous fish specific paralog 1) Complete Coding Sequence
GGGGAGGGGCTGTGCTGGGGTTCAGTGGAGCTGGAGTTGGTGAGTTGCAGTCGGTGTGAGGTGGGATGAGCGGAATGGCACTGGCGATCCAGCGCTCTCGGCAGCGGGACGCGGGGTTGGGCTCTCGGGGCAATGCGCGGCGGTT
CCTGGGGCAGAGCTACGAGGAGCTGCGGGACCGCTGTCTGCAGACGGGGGTCCTCTTCCAGGACCACAGCTTCCCCGCCGCCCCGGACAGTCTGGGCTACCAGGAGCTGGGCGGCCACTCACACAAAACCCGGGGGGTGGAGTGG
CAGCGCCCACCGGATCTGTGCTCAAGACCCCGGTTTATTATTGAAGGAGCCACCAAGACTGACATTTGCCAGGGCTCCTTGGGGGATTGCTGGCTCCTGGCTGCCATCGCGTCCCTGACTTTGAACAAGGAAGTGTTGGACAGAG
TGGTTCCCGATGGACAGAGCTTTGATTCGGGCTACGCCGGAATCTTCCATTTCCAGTTCTGGCAGTACGGGGAGTGGGTGGATGTGGTGATCGATGACCAGCTGCCCACCAAGGATGGAGAGCTAATGTTTGTCCACTCGGACGA
GCAGAATGAGTTCTGGAGCGCGCTGCTGGAGAAAGCTTACGCCAAGCTGAATGGATCGTATGAGGCTCTGTCGGGTGGATCGACGACCGAGGGGTTTGAGGACTTCACGGGGGGCGTGTCAGAGTGGTACGAGCTGAACAAGGCC
CCTGCGGACCTTTTTCACATCATCGAGAAGGCGCTGAAGAGGGGCTCACTGCTCGGCTGCTCCATCAATATCACGAGTGCATCAGAGACCGAGGCGATCACCCCCGAGAAGCTGGTGAAAGGTCACGCCTATTCCATCACTGGAG

CACAGGAGGTTCGGTTCCGCGGGTCCCAGGTGCAGTTGATCCGGATCAGGAACCCCTGGGGGCAGGTGGAGTGGACGGGAGCCTGGAGTGACAGCTCCTCGCATTGGAACTCAGTGTCACCACAGGATGCAGCCGATCTTCGGAA
TCGCGCCGAGGATGGAGAATTCTGGATGTCGTTCTCGGACTTTAAGAGGCACTATTCTCGGGTTGAGATCTGTAACCTGACGCCTGACACCCTCACCTCCGACAAGATCCTCAAATGGAACTGTGCCTTGTTCAGTGAGAGCTGG
AGACGTGGCTCCACAGCTGGAGGCTGCCGGAACTTCCCAGCCACCTTCTGGATGAACCCCCAGTTTAAGATGGTGCTGGAGGAAATCGACGATGATGGACGAGGGGAGGACGGCTGCAGCGTCCTGGTGGCTCTGATCCAGAAAA
ACCGAAGGAACCTGAGGAAGATGGGCGAGGACATGCACACTGTGGGCTTCGCCATCTATGAGGTCCCTGATGAGTACAAAGGGGTCACAAACGTGCACCTGGAGAAAAGCTACTTCATAACACATGGATCAAAAGCACGATCCGA
GACCTTCATTAACCTACGGGAGGTCTCAGCCCGGTTCTGCCTGCCCCCCGGAGAGTACCTGGTTGTCCCCTCCACTTTCGAACCCAACCAAAATGGGGACTTTGCTCTGCGCGTCTACACAGAGAAACGGGCTGACACTCACACA
ATGGACATTGACCAGGTGTATGCAACCTTCGATGATGAGGTTGATGTTCAGGAGTCCGATGTGAGCAGCAACTTCAGGAGTATGTTTGAAAAACTCGCTGGAGAGGACAAGGAAATCTCGGTATTCGAGCTCCAGAAAATACTGA
ACAAAATCATGTCCAAACGCACTGACATCAAGACGGATGGATTCGGGCTGGAGTCGTGCCGCAACATGGTGCATCTGGTGGACAAAGACGGGAACGGAAAGATGGGATTGGTGGAATTCCAGAAACTGTGGAACAAGGTGCAGAA
GTTCCTGAAAATCTATAAAAATAATGACCTGGATCAGTCGGGAACAATGAACTCGAGTGAAATGAGAGTGGCTGTGGAGGAGGCTGGTTTTCACCTGAATAATAAACTCTGTCAGATCATCGTTTCTCGTTACTATGACAGCGAC
GACCTCACCCTGGACTTTGATAACTTTGTTTCCTGTCTTGTCCGCCTGGAGCTGGTGTTCAAGTTGTTCAATTCTCTCCCCAAGGATGAGGAAGGATTCGCTCAACTCAGCATCCTGCAGTGGCTCACAATGGTATTGTGCTGAA
TTGTCTCATGGACCAGGTCTCTTCAGTAAGATACTGGAGATAGGAACACAGCTGAGTACTGGAGCCTCTGCTTATTGCTGTCTTGTTCCCATCTCTGACCCCAATCAAGTACTGCTGGGGCTCTTTTGGTGGGGGCGATGCTCTG
TGCCTTACTGATCATTGGGGTTGATCTCAGCGGGGGGTGGTGGTGGTGGTTCCTTTCATTACTGATTGATTACTGGCTCAATCTTTGTGTGTGTGTGTGGGGCGGGGGTGGCAGGGCTCCATGTCTTATTCCTCTCTTAGTTCTG
TAATGTGGATTCCGTGCTGCCATTGGTGGTATTTTTACATGATGCAAATTAAACCATTTTGGTGATGAAGCTTTAAAAAAAAAA

>Callorhinchus milii_TranscriptomeContig60065_CAPN2 (cartilagenous fish specific paralog 2) Partial Coding Sequence
AGCCGGGTTGTCCCGAGTAGCCAAAACTTTGACTCTGACTATGCTGGAATCTTCCACTTCCAGTTCTGGCAGTACGGGGAGTGGGTGGACGTGGTGATCGACGATCAGCTGCCGACCATGAACGACAGTCTGATGTTTGTGCACT
CGGCCTCAGGTAACGAGTTCTGGAGCGCTCTGCTGGAGAAGGCCTACTCCAAACTGAATGGATCCTATGAATCTCTCTCGGGAGGAACAACGACTGAAGGGTTTGAGGATTTCACGGGGGGGATCGCAGAGTGCTACGACCTGGA
CAGTGCTCCGCCCAATATCTTTAAAATCATCCAAAAGGCGATAAGTCGTGGCTCTTTGCTGGGATGCTCCATAAACATCACAAGCTCAGAGGATTCTGAGGCAATTACATCACAGAAGTTGGTCAAAGGTCATGCTTACTCCATT
ACTGGAGCAGAATCGGTGACATACAACGATGAGCAAGAAAAACTGATCCGGATCCGGAATCCTTGGGGCCAAGTGGAGTGGACTGGACCTTGGAGCGATGGATCGGATGAGTGGTGTTGCGTCCCCGATGAAGAAAAAGAAGCGC
TATGTTCCCGAGTGGAGGACGGCGAGTTCTGGATGTCATTTTCAGATTTTATGTTTCATTACTCCCGAGTGGAAATCTGTAACTTGACGCCAGATGTTCTGAGTGGAGACGAGATCATCAAATGGAACTTGAAACTCTTTGAGGG
CACCTGGAGAAATGGATCTACCGCTGGAGGATGTCGAAACTATCCAGCCACCTTTTGGGTAAACCCTCAGTTTAAGATCGAGCTGAGGGATACGGACGATGATCCTGATGACGAGGATAATCCGTGCAGCTTCCTGATCGGTTTG
ATCCAGAAGAATCGCAGGCCCAAGAGGAAGATGGGGCAAGACATGCTGACCATTGGCTTCGCTATCTATAAGGTCCCCGATGAGTGTGCAGACCAAACGAATATTCACCTGAAGAGGGATTTCTTCATGAGGCACGCGTCCAAAG
CTCGATCGAACACCTTCACCAACCTGCGAGAGGTCTCGAACCGACTGTCTCTCGAACCCGGAGATTACCTGATTGTCCCGTCCACCTTCGAACCCAACCAAAATGGAAACTTTGCCATCCGATTGTTTACAGAGAAGCAAGCTGG
CGTGCAAGAGATGGATGATGAGGTTGATGCTCAACTGGATGATGAGCCACAGATTGAAGAAGATGACATCAGTAGCAGTTTTGAGAATATGTTTGACCAGTTAGCTGGACAGGACAGCGAAATTTCTGCCTTTGAACTGCAAAGA
ATACTGAACAAAATTCTAAGCCAACGATCAGACCTTAGAAGCGATGGATTCTCCTTAGAAACCTGTCGAAATGTCGTCAGTCTCCTGGATAAAGACGGATCTGGCAAAATTGGATTGGTGGAATTCCGGATATTTTGGTCCAAAA
TACAGAAATATGTGCACGTCTTCAAGCAGATGGATGCTGACGAGTCTGGTACCATGAGTGCATATGAGTTACGACTGTCTCTCCAGGAAATAGGTTTCAATCTGAACAATCATTTGGTTCAGATCCTCATTGCCCGATACGCAGA
CAATAATTTGGAGATCGACTTCGATAACTTTGTTGGATGTTTGGTCCGCTTGGAAACCATGTTCAGAATATTCAAATTATTAGACGAAGGAGGGGAAGGAACCGTGCAGCTTGATATTGTTCAGTGGCTGACTCTGGTCTTCAAT
TGAAGAATCGCTGTGAGACTCTGAGAAGATTTTTTCACCAAAGTAACAAATAACCAGGAGATGCTAAGGGATTGTTGATTTTGTGTTTTTATGTGCTTATTGCGAGTGCTGAAATGAGGGTGTTAGGGACCTTTTGAATGTTAG
AATGAG

>Callorhinchus milii_TranscriptomeContig81212_CAPN9 Partial Coding Sequence
CCCACCAAAGCAGAATTTTCAGGAGAATTATGCTGGCATTTTTCATTTTTGGTTTTGGCAATACAACGAGTGGGTGGAGGTGGTGGTTGATGATCGGTTACCAACCTTCAGAGATCGCTTGGTCTACCTTCACTCTGCTGAGCAA
AATGAGTTCTGGAGTGCCTTGTTGGAAAAGGCCTATGCCAAACTAAATGGCAACTATGAATCGCTAAAGGGAGGAAATACACTGGAGGCCATGGAGGACTTCACTGGGGGATTGGGAGAGATGTTTGATCTCAATGAGTCATCTT
CAGACATGTATGCAACCATTCAGAAGGCACTGCAAAGGCGTTCCATGGTGGGCTGTTCCATTGACATTTCAAGCTCTGCTGAAACTGAGGCTCGCACCCCTTCAGGACTGATTAAAGGACACGCATATTCTGTCACTGGGCTCCA
GGAGATAAAATACAAGGGGAAGCCAGTGCGGCTGATCCGAGTAAGAAACCCTTGGGGTCAAGTGGAGTGGAATGGAGCATGGAGTGACAACTCTGCTGAATGGAAGATGATCGACACGTCTGAACATCGCACACTGAACATGGTC
TCCAAAGACGACGGGGAGTTTTGGATGGCCTTTGAAGATTTCTGCAAACATTTTGGCAAACTAGAAGTCTGTAACCTTACCCCAGATTCTTTGTATGGTGACTCTACTCGGAAATGGAACGTGTCGGTTTATGAAGGGAAGTGGC
AGAAAGGCTCTACGGCGGGAGGCTGCAGAAACTTCCCAGAAACTTTCTGGACAAATCCTCAGTATAAGCTGAGGCTACATGATGCAGATGATGGTAAAAACGACTCTACACTTATGATAGCTATCCTTCAAAAGAACAGACGTAA
ACTGAGGAAAGAAGGAGTTGATTTGTTAACCATTGGATTTGCTATTTATAAGGCAGAACCTGGTGATGACCATTTGCCAGAAGAATTCTTCCAATTTCATGCTTCGGTAGCTAGAAGTAAGAGCTACATTAATGTAAGAGAAATC
TGCCAAAGGTTCCAACTCCCACCTGGAGATTACATCCTTGTTCCAACTACTTTTCAACCTCACCATGAAGCTGATTTTGTCATTCGGATCTTCTCAGAGAAGAAAAATGAATCTCTTGAAATGGGGGATAAAATTGGTATGGATC
TTCCTGATCCACCTACACCATCCAAACTCAACAGGGAGACAGAAGAGGAAAGGCAATTCCGCAAACTTTTCGAAGATATATCAGGAGAGGACCTGGAAATTGACGCATATGAACTGCAGAAGATTTTAAGCACTGTGTTTGCAGC
ACAAAAGGAGCTGGCGACTGACCAGTTTGACATTGAGACGTGCCGAAGTATTGTGTCTCTGTACGCTAAAGATGAAAGAAGGATGCTCGGATTTGAAGAGTTTAAGTACCTCTGGACCAGGATGAAGATGTGGAAGACTGCTTTC
CTGAAGTGTGATAATGACAACTCTGGCACGATATCCTCTTACGAGCTTCGTTCAGCCATCGAAGAAGCAGGATTTCAAGTTAACAACCAACTAATACAGTTGCTAGTCCTGCGATACGCCAACGATTATATGGAGATTTACTTTG
ATAACTTCATCCGATGCCTCGTGCGTCTGGAGACGTCATTCAGATCATTCATGAATTTTGATACCAAAAAGACTGGTGAAATATCCATAAATATGTTGCAGTGGCTCCTCCTGACTATGAACATCTGAAGATCTATTGTGAATTC
TGTGACAAGCTATATCGATTGAGGCAGAAACATGGGCTATTATAGAGAACAGAAATGTGGTGCTATTCTTTGTACTTTTTCCCTCTTTCTTAAAATGTACTCGGTGTATCTAATTTGCACTTGTTAGAAATGTTCTGGAGAAAGC
ATCTATTTCCATTAATGTTGCTTTAATATTTGTAATAAGATTTGAGTTAGGATTTTGCTGCAGGGGGAAACGTCCCTCCTTTCGGAGTTCAATAGTCACAAATGTTCCCGAATGGGATGTGAAGAGAGAGATTAGATTTTGTTTT
TCCTCACAGAGCAGTGGAGGTGTGAAACATATTCCCAATAAAGTGTTGCATTGGTTAACTCCTTAAGAAAAACTGGATCAGTAACTGCTTCCAAACAGTATTTGAGGATACAGAGAAATTGTAGAGTGAGATGAACAAGTATGCT
ACAAATACAATGCACTTCTGAGGAATGGGAATGGAATAATTTCTGTTGAAGGAAAGATATTAGGTTTGAACAGTGGCAATGTGGAGGTGGATGATGATTTGAAAATGTTGCCTGTGGGATCATGGAATATCTGCTGAATTTAATC
CCATAAATGGTGTGATTTCCATGATAATAAAATAACTTTATATAGCATAATTCATGTGGGTAGCGTTTTGTGGTGCTTAAGAGTGGAGGAGCCTGGCACCCAGCTGGAGAGAGGGGAGGTGACCGTAGACACAGTTGAGCAGAAA
TGTTCC

>Leucoraja erinacea_TranscriptomeContig62832_CAPN2 (cartilagenous fish specific paralog 2) Complete Coding Sequence
CGGTCTCGGCATCCTGCTGAACCGCTCTTCCGATCTGTGAAAGCTGCACAGCAGATTCCTTCCAGAGAAGGCAGGTTTTCCACGCCGTGACGAATTGAGTATAGCGTGCCCCCCGCGGATCGGCCGTCATTATGTCAGGAATGGC
ATCTAATATCAAGAAGCAGAGGGAGAGAGCGGCCGGGATCGGCTCCTGGCAGCAACCGGTGAAGTTTCTCAATCAAGATTATGAAGCGATCCGGCAGCATTGCTTGGATACTGGAAACTTGTTCTGTGACGAGTCTTTCCCTGCT
TGTCCTACATCCCTGGGATTCAAAGAACTCGGACCCGATTCGTACAAAACCAGAGACGTCGAGTGGATTCGCCCGAAGGATGTGTGTGGCAACCCTGAATTTATAATTGGTGGAGCATCACGTACTGATATCTGCCAGGGAGCGC
TAGGTGACTGCTGGCTATTGGCTGCTATTGCATCACTAACCCTAAACAATGAGGTTCTAAACAGAGTGGTTCCCTGTGAACAAGACTTTGGCTCTGATTATGCAGGAATCTTCCATTTCCGGTTCTGGCAGTATGGAGAGTGGGT
GGATGTGGTGATCGATGACAGGTTGCCCACAAAGGATAATGAGCTGATGTTTGTGCACTCAGCTTCAGGAAATGAGTTCTGGAGTGCTCTGCTGGAGAAAGCCTATTCAAAGTTAAATGGGTCCTATGAAGCATTGTCTGGGGGA
TCTACAACTGAAGGCTTTGAGGATTTTACAGGGGGGATTTCAGAGTGGTATGAATTAAATGAAGCACCAAGCAACCTCTTTAAGATCATACGAAAGGCATTAAAAGCTGGGTCACTACTTGGCTGTTCCATTAATATCACCAGCT
CAGCAGATTCAGAGGCAATTACTTCACAGAAGCTGGTCAAAGGTCATGCTTATTCTTTAACTGGAGCGGAAACAGTGGACTATCAAGGCAACGCGGAGCAATTGGTCCGCGTCCGTAATCCATGGGGTCAAGTGGAGTGGACTGG
AAGCTGGTCCGACAATGCACCAGAGTGGGAATGCGTTCCTGAAGAAGAAAAAGATCGCCTATGTGACCAATCAGAGAATGGCGAATTCTGGATGTCATTTTCCGAATTTCTTAGTAATTTCTCTCGAGTGGAAATCTGCAATCTC
ACCCCCGACACACTTAGCAATGATGAGGTTCACAAGTGGAACATGGCTGTCTACAATGGGTCCTGGAGGAAAGGATCTTCTGCGGGAGGATGTCGAAACTATATAAGAACATTTTGGCAAAATCCTCAATTTAAGATTGATTTGA
GGGAGGCAGATGAAGATCCTGATGATGATGATGAGAAATGCAGTTTTCTGGTCGGGGTTATTCAGAGGAATCGTAGAAATAAAAGGAAAATGGGCGAGGACATGCATACTATTGGCTTTGCTGTCTATGAGGTCCCTGAAGAGTA
CCACGACCACAAGAATATTCAACTCAGGAGAGATTTTTTCACAACTCATGCATCCCAAGCAAAATCAGAAACCTTCATCAATCTGCGAGAAGTCATGAGCAGGCTTGCACTTGAACCTGGACAATATTTTGTAATACCATCCACC
TTTGAGCCGAATAAAGATGGGGATTTTGCAATCCGCTTGTTTACAGAGAAGGCAGCCAATGTAGAAGCGATAGATGTTGTAGTTGATGCAATAATTGAAGACGAGGAGGTTGACGAGGATGACATTAGTGCCAGCTTTGAGAATT
TGTTTGAATATATAGCAGGGGAGGATGCAGAGATTTCTTGCTTTGAGCTGCAAAGAATACTGAACAGAATTGTAGGCAACCGTGAAGACATTAAAACTGATGGTTTTAGTTTGGAGACCTGTCGAAATGTAATCAGTCTAATGGA
TAAGGATGGATCTGGAAAAATTGGATTAGTGGAATTCAAGCTATTTTGGAACAAGTTGCAGAAGATGCTGAAAATCTTCAAGAGGGTAGATGCTGATGAATCTGGTACCATGGGTTCATGTGAAATACAAGTGGCACTCGAAGAA
GCAGGTTTCACTCTGAACACTGCATTGATTCAGAACATTGTTGCCCGTTATGCAAACTCCGACCTTCTCATCGACTTTGATAACTTTGTTGGCTGTTTGATCCGATTGGAAACCATGTTCAGAATATTTAAAGCACTAGAAGTCG
ATGATGAGGGATCGGCAGAGTTGGATATATTTCAGTGGTTGGGTCTGGTACTGGGCTAAAATATTGCTCTGCTGCAAGCCAAGCTTTGACCAATCAACAGATGCAGAAAAGAACATCTCAAAACTACGAAAGGAACATGAACCCT
AATATTTAATTTAATGGGCATCATGAATGGGTATAGAGTGAGGGGAGTAGGGTTTGTTTTTAATGCTTCGCTGTACAATCTTTTATACAGAAATTTGGGTGATATTTGATCAGAACTGTGTCTCAATCAGTATCATACTGTGCAC
CGATATTACCAAAAGACATCTTAAATCATAATTACCATGTTATACAATTTATATGGCCCACATTGAAGATAATAAATGGGACACATTTGTTCTGCAATGTTAAAAGATACAAAATTTAACAAATTTGTTATTTTACCTCCGTCTA
CAAATGTGGGCACAGGTAAATCACAAAAATTATACCAGGAGAATCATGATTTGGAATTTAAAGGGCCTGTCCCACTTAGGCAATTTGGACTGCCGAAAAATTTTCAACAT

>Leucoraja erinacea_TranscriptomeContig91257_CAPN9 Complete Coding Sequence
AGGAAACCCGGCGCAGTCTTGCTCCAGCAGGTGAATTGGCAACTTGCTCAGGATCTCTCGCCCCCACCGTGTCTCAAACATGTCCTATGTGTTCACCTCGGCGAGCGGGGGCTCCCAGAAGAAGACGGCGGTGCAAGCCCGGGGG
ACGGTCGGAGTCAAGGCCAGGAAACTCACCTTCCAGGAGCTGAAGCAACACTGTCTGAAGGAGCAAGTCCTGTACGAGGACCCGGAGTTCCCCGCCGTCGAGTCGTCGCTCTTCTACAGCGAGAAGCCTCCAGTTGCCTTTGAGT
GGAAACGGCCGAAGGAGATTTCCAAAGACCCGAAGTTCTTTGTTGGTGGAGCATCAAGGACTGATATTTGTCAGGGAGATCTGGGAGACTGTTGGTTGCTCGCTGCTGTGGCCTCACTAACTCTGAATGAAAAGCTGCTCCACCG
AGTTATCCCTCCCAATCAGACCTTCGGCCGGGATTACGCTGGAATCTTTCACTTTCAGTTCTGGCAATCTAACAAGTGGGTGGATATTGTGATTGATGATCGTCTGCCAACCTTCAAAAACCGCCTTGTGTTTCTACACTCTGCT
AGTAGCGATGAATTCTGGAGCGCCTTACTGGAGAAAGCCTACGCCAAGATGTACGGAAGCTTTGAGTCGCTGAAAGGAGGGAGCACCTTGGAGGCCATGGAAGACTTCACTGGTGGAATGGGAGAGATATTTGATTTGAAGAATG
CACCCCCAAATCAGTTTTCCGTCATTAAAAAGGCACTGCACAAGGGGTGCATGTTGGGATGCTCCATTGACATCACCAATCCAGCTGAAACAGAAGCTCGGACTTCGACAGGACTCGTCAAAGGACATGCATATTCCATTACTGG
TTTGGACCAGGTGAACTACAGAGGTCGACCGCTGCAGATGATCCGTATAAGGAACCCTTGGGGTCAAGTGGAATGGAATGGTCCCTGGAGCGATGACTCTCCTGAATGGAAGGCAGTGGATGCAACGGATTTTCGAAGGCTAAAC
ATGACCTCCAAAGACGATGGCGAATTTTGGATGTCATTCGATGACTTTAAGAGACATTACAGCAAAGTGGAAATTTGTAATCTTACACCGGACTCCCTGGATGATGATTCCAGTCACAAGTGGGAGGTGACCTGTTTTGAAGGGA
TGTGGACCACAGGCTGCACAGCAGGTGGATGCAGGAACTTCCTAGACACTTTCTGGACCAACCCCCAGTACAAGATCAAGCTGCTGGAAGAGGATGAGGAGCAGGAGGATGGCAAAAAGGAATGCACCATGGTGGTGGCTCTCAT
GCAAAAGAATCGGCGCAAATTGCGGAAGGAAGGAGCGGTGCTGCAGACCATTGGATTCACTATTTATCAGGCTGAAAGTGGAGCTGACCATCTGCCGAAAGAGTTCTTCCGATTTCATGCTTCCCGTGCAAAGAGTAAGACATAC
GTTAATTTAAGAGAAATCTGTGAAAGATTTAAACTTCCACCAGGAGAATACATCGTTGTTCCCACTACATTTAAACCCAACGATGAGGCTGAATTTCTTGTTCGAATCTTCTCGGAGAAGAAAGCTCAAACAATTGAATTGGGAG
ATAAAATTGGCGCTGATCTACCAGAGCCGCCCAAACCGACGCAAAAACTGCAGGAGAATGAAGACGAAAAGCAGTTCCGCGCTGTGTTTAAGCACATCTCGGGGGAGGATCTAGAAATCAATGCTTATGAACTGCAAGGTATTTT
AGACAACGTGTTTGGAAAACAAAAAGAAGTGGGAATCGAGAGTTTTGACATTGAGACATGTCGCAGTATTGTGTCCCTCTACAATAAAGAGACTGGTAAAGGACTTGGGTTTGACGAATTTAAGATCCTCTGGCAGCGATTAAGC
GTATGGAAGAATGCTTTCCTGAGATGTGATCATGACAAATCAGGGACTATATCAGCTCACGAGCTACGATCAGCTGTCAAGGATGTTGGGTTTTTCCTCAACAATCAGTTGCTACAACTGTTGGTCGTCCGATATGCCGATGCAA
ACCGACAAATCGATTTTGATAACTACCTGCGTTGCATGGTTCGATTGGAGACTTCGTTCAGATCATTCAAGTCTTTTGATACCCGGCAGACTGGTGAAGTGACTTTGAACTTGATGCAGTGGCTCTTCCTGACTATGAACATATG
AAAATTGAACAGAAGACTGAATGAAATATTGTTGTGTGTCAGTGTATACTACTTACAACTGATCTTAGTTTCCACATTAGGCTGGGTGCCATATGTAACTGAATGAGGCCTTATTCTCTGCATTTGATTTTTTATTGAATAATAC
TTTTATATACGAGCATTTTTGTCTGAACTATTTTGGCAAATATACTTGCTACCAAAGGTACAATTCTAGGAAAGTTATCTGCTCCGGGGCTGTCTCATATTCCTCCATTTTGTAAGTAAACGTCATTTCCTGCTACCTTCCAGCA
TTGGATCCTATTGCTCTTTTCATTAATTCCCCCATTGAAGCTCATGTACCTGGATCATTTAGCATTCTCTTTCTCTGGTGACTTTAGGCCACTCAACAATACCCGTCTCCAATATATTATCTTTACTCTCGAACTCCAAGTACGT
AGATTGTCCCATTATACAACTCCTTTCATGACCTTAACTCCCCCCATTAAAGCTATTTACAGAACTTGACGCATACACTTCCAGGGTTAATAATTGTATCAGAAAGTTTCTAAATTCATGATTTGTTTAAATAGGTTACTAATA
TAGGTTACTAAATTTACTAAGACATTAGTAAAGGGTTGTG

>Leucoraja erinacea_TranscriptomeContig63790_CAPN11 Partial Coding Sequence
AGCTGAAGAAATCCCCCCGAAACCTCTACTCAGTCATGCGCAAGGCCCTGGAGCGAGGCTCCCTGCTCGGCTGCTCCATTGACATCACCAGCGCCTTCGACATGGAGGCCGTGACCTTTAAGAAACTGGTCAAAGGTCATGCCTA
CTCAGTGACAGGGGTCAAGGAGGTCAGCTACCGGGGCAGGGTGGAGCGGCTGATCCGGATCAGGAACCCATGGGGTCAGGTGGAGTGGACGGGGGCCTGGAGCGACAGTTCATCGGAATGGGATGCGATCGACCCGGAGGAGAGG
GAGGGAATGGTGAAGATGGAGGACGGGGAGTTCTGGATGTCGTGGGGAGAGTTCCTGCGGCAGTTCTCGCGGCTGGAGATCTGTAACCTGGGCCCAGAGACGCTGCAGGACGATCGGCACCTCAAGTGGCAGAGCACCACCTTCC
GCGGCTCCTGGAGGCGGGGCAGCACCGCAGGGGGCTGTCGCAACCACCCCGCCACGTTCTGGATCAACCCGCAGTTCCGGCTGACCCTGTGCGAGGAGGACGATGACCCTGACGACCCCGAGCAGTCGTGCAGCTTCCTGTGCGC
CCTGATGCAGAAGGACCGGCGCAGGGCCCGGCGCAAGGGCGGGGACATGCACACCATCGGCTTCGCCCTCTACCCCCTGCCCGAGGAGATGAGTGGGGGTCCGCAGGTCCACCTGCGCAAGGACTTCTTCCTGAGCCGGTCGTCG

TGCGCTCGCTCCGACTCCTTCATCAACATGCGTGAGGTGAGCTCCCGGCTCAGCCTGCCCCCCGGCGACTACATCATCGTCCCCTCCACCTTCGAGCCCAGCCACGAGGCCGACTTTGTGCTCAGGGTCTTCACCGAGAAACACA
CCGACTCACAGGAGATGGACGACCCCATCGATGCTGAGTTGGAGGATGAGGAGGAGATGAGCGAGGACGATGTGGAGGATGGATTCCGAGCCATGTTCAAGCAGCTGTCCGGGGAGGACATGGAGATCAGTGTGTTTGAGCTGCG
CACCATCCTCAACCGCGTGCTGGCCAAACACCAGGACCTGCAGACAGACGGCTTCACCCTGGACTCGTGCCGAGGGATGATCAACCTCCTGGACAGAGACGGCAGCGGGCGGCTGGGTCTGCTTGAGTTCCAGCGGCTGTGGAAC
AAGATCCGGGCCTGGCTGGGTATTTTCAGAAACTTTGATCTGGATAAGTCGGGCACCATGAACTCCTACGAGATGCGCCTCGCCCTCGAAACAGCAGGGTTTAAGATCAACAACCACATACACCAGGCCATCGCCTCTCGTTATG
CAGACGGTGAGGTGATCGACTTCGACAACTTCATCTCCTGTCTGGTCCGGCTCGAGGCTATGATCAGAGCATTCCGGTCTCTACAGCACGATGATGGGACCATGCATATGGATCTGTCGGAGTGGCTCAGTTTGACCATGTCGGG
TTAGGACGGAGAGGCCGCTGTGCCAGAGCCTTGCTGCCGCCGCTGCACCCTCGCCCGCTGCTCCGGATCCTTCCGCCGGCGATCCGCCGGCCCTCCGGAACTTTCTGCAAGCATGCAGATCCCTCCCTACATGGTGTACGGCTCA
GAGGACCTGCCTCCCCCACAACCCCCCTAACCCTGACCCCCCACCCCTGACCCTCGCCCTGGTCGTGCCTGTTCTAGAGATGCTACTGGTGCTCTAGCCCCCCCTCCCCCCACCCCAATAGTAACCCCTCCCCCAGATGTGCCTG
TCTCTGGATTATAACCACTAGGCCGCTACAGGTCATGACCCTGGGGCAAATGCCCCCCCCCCCTCCCCCCAGGTTTGCTGCTGTCACTCTCCCCCCACCCCCTCTACAGTGCCGATCATACATCGGCTGCCTGAAGCTCCCCCTC
ACCCCCACCCTTCCCCCTCACCCCCACCCTTCCCTCTCACCCCCACCCTTCCCCCTCACCC

>Leucoraja erinacea_TranscriptomeContig21198_CAPN2 (cartilagenous fish specific paralog 1) Partial Coding Sequence
CTTCACGGGCGGGGTGGCCGAGTGGTTTGAGCTGAAGACGCCCCCCCCCGACCTGTTCTCCATTATCCGCCGGGCTCTGGAGCGAGGGTCACTTATGGGCTGTTCCATCGACATCACCAACGCATCGGAGACGGAGGCCGTGACC
TCGCAGAAGCTGGTCAAAGGTCACGCCTACTCCATGACCGGTGCCGAGGAGGTGATGTTCCGCGGGGGGCCGGCACAGCTGGTGAGGATCCGAAACCCCTGGGGTGAGGTGGAGTGGACCGGGGCCTGGAGTGACAGCTCGGCCC
AGTGGTCGGGCATCGAGGCCGAGGACCGGGACCGACTGCGGCAGCGGACCGACGATGGGGAATTCTGGATGTCGTTCAATGACTTTCTTCGCCACTACTCGCGGCTGGAGATCTGCAACTTGACGCCGGACACGCTGAGCAGCGG
AGACGTTCTGCGCTGGAACCACAGCCTCTACAGCGAGAGCTGGCGGAGAGGTTCCACCGCTGGGGGCTGCCGCAACTACCCAGGGTCGTTCTGGACCAACCCCCAGTTCAAGGTCGTGCTGGAGGAGGGGGACGAGGATGGGTCG
GGTGAATGCAGCATGGTGTTGGCGCTGATCCAGAAGAACCGGAGGCAGAAGCGGAAGATGGGCGAGGACATGAGGACTATCGGCTTTGCCATCTACGATGTGCCGGCCCAGTACGAGTCTGTGAACAACGTGCATCTGCCGCGCT
CCTACTTCACCACGCACGGCTCGCGGGCTCGCTCGGAGAGCTTCATCAACCTGCGGGAGGTGTGTGGCCGATACAGCCTCCCTCCCGGGCAATACCTGGTCGTCCCCTCCACCTTCGAACCCAACCAGGACGCCGACTTTGCACT
CCGGGTCTTCACCCAGTCCAACTGCCTCACACAACCCATGGACATCGACACTAACGGGGTTTCAGCAACGTTTGAGGATGAGGGGGAGGTGAGTGAAGATGATGTCCCCGACAACTTCAAGGCGCTGTTCAACAAACTGGCCGGA
CCTAGTGGGGAGATCACTGTGTTTGGTCTGCAGAGGATCCTCAACAAGGTGGTGTCCAGCCGCTCGGACATAAAAACGGACGGATTTGGTTTGGAGACCTGTCGTAACATGGTCAACCTGCTCGACCGTGATGGAAACGGGCAAC
TGGGATTGCTGGAGTTTAAGAAACTGTGGGACAAGATTCAGAAATTCCTGAAAGTCTACAAGCGGAACGACTTGGACGGCTCGGGCACCATGAGCTCCAGCGAGATGAGAGTGGCCGTGGAGGAGTCTGGGTTCCAGCTGAACAA
CCAGCTCACCCAGATCCTGGTGGCCCGATATTCAGACAGCGAGCAGCTGGTGCTGGACTTTGACAACTTTGTGTCCTGTCTCATCCGCCTCGAGGCCGTCTTCAAAATGTTCAACGCGCTCCCCAAAGATGAGAATGGAAACATT
GAACTGGGAATGTTCCAGTGGCTGACCTTGGTGCTGGGCTGATGGTCAGTCTGGGCCTCGACCAGATGCATCTCTGCCGCTCTGCAAGAGGGTCCTCTCCGGCCACTCTCGCCTCCACACGGACCCTCGACTGCTGGGGGGGGGG
GAAAGGGGGGACAGTGGGAGAGGGCGACTCTCACACCCGGCTGTTCATGCTGTTACCTGTAACTGGGGGTTGGGGAGGGCTGGGTGGGGGTG

(*b* and *c*). Aligned sequence data used in phylogenetic analyses in Newick format.

(*b*) The 210 sequence amino acid alignment used for the main phylogenetic analysis.

```
>Xenopus_tropicalis_ENSXETG00000022058
QDFHALRDLHLRKGVLFNDEEFPADMRVIG-
KIHNMEWRRPPHLIVDGASYFDIVQSGDCWVLSTIGSVTQKQTLLRNIIPADQGFTRNYAGIFHFRFWYSGKWVDVVIDDRLPFLNGDFFSVRASCAKEYWPCLLEKAYANLYQGYWYIIWTTGAFQCNNFTNVTTTDFKAVPPP
---IVQKPN----LFLCGLVDRHAYTVTDTAQYREGCVNLIRLWNPWGRGEWIGRWSDSWWNDIDPEDRKRL-EDGEFWMSWETFVQQFSRVIICSPTLDFL-WHKTSYENIW----
KAFCKNPQYLIRISPYNVIISLMPIGFLVSFHINKRDVTKGLKLASGRYVITPFNADRSQESSFLLQVFLRKMNSQDLQKFLNDVISASRSMLASMDNGKLELEFFMRLWRYLNHFKIFTDVDVDQNGFIGLSELRKAAKSAGAV
SSDQLTILLLRYGDMKLNFEDYLCCMVRLKSAMSC
>Xenopus_tropicalis_ENSXETG00000023299
QDYDRILHECLQNRTLFKDDTFPPSAYSLGSKTYGVKWVRPPQFIVDGATRTDICQGGDCWLLAAIASLTLNDTILHRVVPHGQSFQDNYAGVFHFQLWQFGEWVDVVIDDYLPVKDGKLVFVHSASGNEFWSALLEKAYAKANG
CYEALSGGSTSEGFEDFTGGVTEWYEMKKAPLFSIIMKAVERGSLMGCKLVKGHAYSVTGAKEFRGRSQKLIRMRNPWGEVEWTGAWSDSEWNSVDAADSQDL-
EDGEFWMAFEDFLREFSRLEICNLTPDALKWNTTVFNGNWRKGSTAFWINPQFKIKLDECTFLLALMTIGFAVYFFINLREVSTRHKLPPGEYIVVPSTFEPNVEGDFVVRFFSEEISATELQSILNKIMSSCRSMVNLMDNGKL
GLVEFNVLWNKIKNYLVFRKFDMDKSGSMSAYEMRLAVESAGKLTNSLHQLIITRYSEMAVNFDSFTCCLIRLETMMFA
>Sus_scrofa_ENSSSCG00000012999
QDYEQLRAHCLQSGSLFRDEAFPPVPQSLGSKTYGVKWKRPPQFIVDGATRTDICQGGDCWLLAAIASLTLNDTLLHRVVPHGQSFQNGYAGIFHFQLWQFGEWVDVVVDDLLPTKDGKLVFVHSAQGNEFWSALLEKAYAKVNG
SYEALSGGSTSEGFEDFTGGVTEWYELRKAPLYSIILKALERGSLLGCKLVKGHAYSVTGAKQYQGQMVNLIRMRNPWGEVEWTGAWSDSEWNGVDPYQRDQL-
EDGEFWMSFRDFLREFTRLEICNLTPDALNWNTTLYEGTWRRGSTAFWVNPQFKIRLEECSFVLALMTIGFAVYFFINLREVSTRFRLPPGEYVVVPSTFEPNKEGDFVLRFFSEEISVRELRTILNRIISSCRSMVNLMDNGKL
GLVEFNILWNRIRNYLIFRKFDLDKSGSMSAYEMRMAIESAGKLNKKLFELIITRYSELAVDFDNFVCCLVRLETMMFA
>Homo_sapiens_NM_001198869
QDYEQLRVRCLQSGTLFRDEAFPPVPQSLGSKTYGIKWKRPPQFIVDGATRTDICQGGDCWLLAAIASLTLNDTLLHRVVPHGQSFQNGYAGIFHFQLWQFGEWVDVVVDDLLPIKDGKLVFVHSAEGNEFWSALLEKAYAKVNG
SYEALSGGSTSEGFEDFTGGVTEWYELRKAPLYQIILKALERGSLLGCKLVKGHAYSVTGAKQYRGQVVSLIRMRNPWGEVEWTGAWSDSEWNNVDPYERDQL-
EDGEFWMSFRDFMREFTRLEICNLTPDALKWNTTLYEGTWRRGSTAFWVNPQFKIRLDECSFVLALMTIGFAVYFFINLREVSTRFRLPPGEYVVVPSTFEPNKEGDFVLRFFSEEISVKELRTILNRIISSCRSMVNLMDNGKL
GLVEFNILWNRIRNYLIFRKFDLDKSGSMSAYEMRMAIESAGKLNKKLYELIITRYSELAVDFDNFVCCLVRLETMMFA
>Mus_musculus_ENSMUSG00000024942
QDYETLRARCLQSGVLFQDEAFPPVSHSLGSKTYGIKWKRPPQFIVDGATRTDICQGGDCWLLAAIASLTLNETILHRVVPYGQSFQDGYAGIFHFQLWQFGEWVDVVIDDLLPTKDGKLVFVHSAQGNEFWSALLEKAYAKVNG
SYEALSGGCTSEAFEDFTGGVTEWYDLQKAPLYQIILKALERGSLLGCNLVRGHAYSVTGAKQYQGQRVNLIRMRNPWGEVEWKGPWSDYEWNKVDPYEREQL-
EDGEFWMSFRDFIREFTKLEICNLTPDALNWNTTFYEGTWRRGSTAFWVNPQFKIRLEECSFLLALMTIGFAVYFFINLREVSNRIRLPPGEYIVVPSTFEPNKEGDFLLRFFSEEISVKELQTILNRIISSCRSMVNLMDNGKL
GLVEFNILWNRIRNYLIFRKFDLDKSGSMSAYEMRMAIEAAGKLNKKLHELIITRYSELAVDFDNFVCCLVRLETMMFA
>Anolis_carolinensis_ENSACAG00000016579
QDYERLRNECLQSGGLFRDETFPASAASLGSKTYGVKWKRPPLFIIDGATRTDVCQGGDCWLLAAIASLTLNDPLLHRVVPHGQSFQNGYAGIFHFQIWQFGEWVDVVVDDLLPTKDGKLVFVHSAEGNEFWSALLEKAYAKLNG
CYEALSGGSTSEGFEDFTGGVTEWYDLRKPPLFQIILKALERGSLMGCKLVKGHAYSVTGGEQYRGQRVSLIRIRNPWGEVEWTGAWSDAEWNTVDPSVGQQL-
EDGEFWMSFQDFLREFSRLEICNLTPDALKWNTTLYDGTWRRGSTAFWINPQFKVRLEECSFVMALMTIGFAVYFFINLREVSTRFKLPPGEYIVVPSTFDPNKEGDFVLRVFSEEISVTELQTILNRIIGSCRSMVNLMDNGKL
GLVEFNILWTKIRGYLVFRKFDLDKSGTMSAYEMRMALESAGKLNKKLYELIITRYAELAIDFDNFVCCLVRLETMMFA
>Pelodiscus_sinensis_ENSPSIG00000008053
QDFETLRNACLQRGSLFQDDTFPPTASSLGSKTYGIKWKRPPQFIVDGATRTDICQGGDCWLLAAIASLTLNDTILHRVVPHGQSFQSGYAGIFHFQIWQFGEWVDVVVDDLLPTKDGKLTFVHSAEGNEFWSALLEKAYAKVNG
CYEALSGGSTSEGFEDFTGGVTEWYELRKPPLYQIILKALERGSLLGCKLVKGHAYSVTGAKQFRGQSVNLIRMRNPWGEVEWTGSWSDSEWNVVDPSVGQQL-
EDGEFWMSFQDFLREFSRLEICNLTPDTLKWNTTLYDGTWRRGSTAFWINPQFKIRLEECSFVMALMTIGFAVYFFINLREVSTRFKLPPGEYIVVPSTFEPNKEGDFVLRVFSEEISVSELQTILNKIISSCRSMVNLMDNGKL
GLVEFNILWNKIRNYLVFRKFDLDKSGCMSAYEMRMALESAGKLNKKLYELIITRYSELAVDFDNFVCCLVRLETMMFA
>Sarcophilus_harrisii_ENSSHAG00000003383
QDYEKLRSQCLQSGVLFEDDTFPASAHSLGSKTYGIKWKRPPRFIVDGATRTDICQGGDCWLLAAIASITLNDTILHRVVPHGQSFQEGYAGIFHFQLWQFGEWIDVVVDDLLPTKDGKLLFVHSAQGNEFWSALLEKAYAKVNG
SYEALAGGSTSEGFEDFTGGVTEWFDLKKPPLYYIILKALERGSMLGCKLVKGHAYSVTGAQQHRGRSVNLIRMRNPWGEVEWTGPWSDSEWSQVAPSDRQQL-
```

EDGEFWMSFSDFLREFTRLEICNLTPDTLKWNTTLYDGTWRRGSTAFWVNPQFKIKLEECSFLLALMTIGFAIYFFVNLREVSSRICLPPGEYIVVPSTFEPNKEGDFVLRFFSEEISVKELQTILNKIISSCRSLLTLVQNGKL
GLVEFNILWNRIKNYLIFRKYDLDKSGSMSSYEMRMALEAAGKLNKKLHGLIITRYSELNLNFDSFVCCLVRLETMMFA
>Taeniopygia_guttata_ENSTGUG00000014528
QDFGQLAEECRRSGTLFQDPSFPPAATSLGSKTRGVQWKRPPQFIVDGATRTDICQGGDCWLLAAIASLTLNETFLHRVVPHGQSFQQGYAGIFHFQIWQFGEWLDVVVDDFLPTKDGKLLFVHSAEGSEFWSALLEKAYAKVNG
CYEALSGGSTSEGFEDFTGGVTEWFDLRRPPLYQIILKALERGSLLGCKLVKGHAYSVTGAKQYRGQALELIRMRNPWGEVEWTGAWSDAEWHAVEPALRQQL-
EDGEFWMSFRDFLREFTRLEICNLTPDALKWNTRLYDGTWRRGSTAFWINPQFKIQLEECSFLLALMTIGFAVYFFINLREVSTRFRLPPGEYIVVPSTFEPNKEGDFVLRVFSELMSVIGCDIICCDIIGNGRHPAKLLQNGKL
GLVEFNVLWNRIRNYLIFRKFDLDKSGS---------------------------------------------------
>Latimeria_chalumnae_ENSLACG00000018300
QDYEALQAACLQNRVLFDDEAFPTCATALGSKTRGIQWKRPPQFIVDGATRTDICQGGKCWLPLHLAAITLSRRYLHNNVIGGKKTGLEKSGEFHVQFWQFGEWVDVVIDDRLPMKDGKLMFVHSAEGNEFWSALLEKAYAKVNG
CYEALSGGSTSEGFEDFTGGVTEMYELKKAPLYEIIQKALERGSLLGCKLVKGHAYSVTGANEYRGQQVKLVRIRNPWGEVEWTGAWSDGEWSGVNPSVREEL-
EDGEFWMSFNDFLREFSRLEICNLTPDALKWNTANYDGLWRRGSTAFWINPQFKIRLDECSFLVALMTIGFAIYFFINLREVSSRCKLPPGEYIIVPSTFEPNKEGDFVLRVFSEEISVRELQTILNRIVSSCRCMVNLMDNGKL
GLVEFNILWGKIRKYLVFRKFDLDKSGTMNAYEMRLALESAG-------------------------------------
>Scyliorhinus_canicula_contig00627
QDYETLRDDCLDRGVQFVDPHFPVGPSILGTKTYGIRWKRPPQFILDGATRTDICQGGDCWLLAAIASLTLNEKILHRVVPHGQSFQRQYCGIFHFQFWQFGEWVDVVIDDQLPVKDGKLVFVHSAARNEFWSALLEKAYAKLSG
CYEALSGGSTSEGFEDFTGGVTEMYDLGKSPLYRIIQKALERGSLMGCKLVKGHAYSLTGAQECRGSLVKLVRLRNPWGEVEWTGAWSDREWNQVDGAQCSQL-
EDGEFWMSFADFQREFNRLEICNLTPDTLKWSTSLYEGAWRRGSTAYWINPQFKIRLRECSFLVALMTIGFAVYFFINLREVSSRFKLPAAVH----------------------------------------------------
-------------------------------------------------------------------------------
>Gadus_morhua_ENSGMOG00000012126
QDYESLKANCAQRRGLFEDPLFPCAPSSLGSKTSGVRWMRPPKFIMDGATRTDICQGGDCWLLAAIASLTLNDQLLHRVVPHGQSFDSGYVGIFHFQFWQFGEWVEVVIDDRLPVKEGKLLFVHSAEGTEFWSALIEKAYAKLNG
CYEALSGGSTSEGFEDFTGGVTEMYDLPKAPLFSIVGRAIERGSLLGCKLVKGHAYSVTGVNEYRGTPTKLVRIRNPWGEVEWTGSWSDREWDSVDRNVRSKL-
EDGEFWMSFSDFLREFSRLEICNLTPDALKWSTSLYQGEWRRGSTAYWINPQFKVALEHCSFLVALMTIGFAIYYFINLREVSSRFSLPPGEYIIVPSTFEPQKEGDFVLRVFSEEISVKELQTILNRIIGACRSMINLMDSGKL
GLAEFHVLWEKIKRYLIFRQFDMDKSGTMNSYEMRRALDSAGKLTNHLFQLIILRYTELTVDFDNFVTCLVRLETMMFA
>Takifugu_rubripes_ENSTRUG00000008157
QDYERLKTQSLQSGRLFEDNLFPCSASSLGSKTSGVRWIRPPEFIVDGATRTDICQGGDCWLLAAIASLTLNDNLLHRVVPHGQSFAHGYIGIFHFQFWQFGEWVDVVIDDRLPVKDGKLLFVHSAEGTEFWSALLEKAYAKLNG
CYEALSGGSTSEGFEDFTGGVTEMYELRKAPLYSIISRALERGSLLGCKLVKGHAYSVTAADEYRGSPTKLVRIRNPWGEVEWTGPWSDREWDNVDRSVRSRL-
EDGEFWMGFNDFLREFTTLEICNLTPDALKWSTSLYQGEWRRGSTAFWLNPQFKLVLKNCSFLVGLMTIGFAIYFFINLREVSSRLQLPAGEYVIVPSTFEPHKEANFVLRVFSEEISITELQTILNRIISACRSMINLMDSGKL
GLTEFHVLWEKIKRYLIFRTFDLDKSGTMSSYEMRMALESAGKLNNNLFQLIILRYTEMSVDFDNFVTCLVRLETMMFA
>Danio_rerio_ENSDARG00000045199
QDYETLRAQSQQSRRLFEDPMFTASSSSLGSKTQGVRWMRPPQFIVDGATRTDICQGGDCWLLAAIASLTLNDNLLHRVVPHGQDFDSRYAGIFHFQFWQFGEWVDVVIDDRLPTKDGKLLFVHSAEGGEFWSALLEKAYAKLNG
CYEALSGGSTCEGFEDFTGGVTEMYELKKAPLFSIIGRAIERGSLLGCKLVKGHAYSVTGAEEYRGNMTKLVRIRNPWGEVEWTGAWSDREWDNVDRSVRGRL-
EDGEFWMSFSDFLREFTRLEICNLTADALKWSTANYNGEWRRGSTAFWINPQFKVALKHCSFLVALMTIGFAIYFFINLREVSSRFRLPAGEYIIVPSTFEPNKEADFVLRVFSEEISVTELQTILNRIIASCRSMINLMDSGKL
GLVEFHVLWEKIKRYLIFRDHDVDKSGTMSSYEMRKALETAGKLNNHLFQLIILRYTELSVDFDNFVSCLVRLETMMFA
>Xiphophorus_maculatus_ENSXMAG00000008475
QDYEFLKAQCLQSGTLFEDNLFPCATSSLGSKTSGVRWMRPPEFIVDGATRTDICQGGDCWLLAAIASLTLNDNLLHRVVPHGQTFQQGYAGIFHFQFWQFGEWVDVVIDDRLPAKNGKLLFVHSAEGTEFWSALLEKAYAKLNG
CYEALSGGSTSEGFEDFTGGVTEMFELKNAPLFSIISRAVERGSLLGCKLVKGHAYSLTAVNEYRGNPTKLVRIRNPWGEVEWTGAWSDREWDSVDSSARSRL-
EDGEFWMSFTDFLREFTRLEICNLTADALKWSTSLFGGEWRRGSTAFWLNPQYKIMLQHCSFLVALMTIGFALYFFINLREVSSRLKLPVGEYIIVPSTFEPNNEGDFVLRVFSEEISMTELQTILNRIIGACRSMINLMDSGKL
GLTEFHVLWEKIKRYLIFRQFDLDKSGTMNSYEMRMALESAGKLNNHLFQLIILRYTEMSIDFDNFVTCLVRLETMMFA
>Oreochromis_niloticus_ENSONIG00000013071
QDYDSLKAQCLQSGGLFEDSLFPAVASSLGSKTSGVRWMRPPEFIVDGATRTDICQGGDCWLLAAIASLTLNDNLLHRVVPHGQGFQQGYAGIFHFQFWQFGEWVDVVIDDRLPVKDGKLLFVHSAEGTEFWSALMEKAYAKLNG
CYEALSGGSTCEGFEDFTGGVTEMYDLNKAPLFSIIRRAVERGSLLGCKLVKGHAYSLTGVEEYRGNMTRLVRIRNPWGEVEWTGAWSDREWDSIDASSRSRL-
EDGEFWMSFNDFLREFSRLEICNLTADALKWSSSLFQGEWRRGSTAFWLNPQFKIMLQNCSFVVALMTIGFALYFFINLREVSSRLKLPKGEYIIVPSTFEPNKESDFVLRVFSEEISVTELQTILNRIISACRSMINLMDTGKL
GLTEFHVLWEKIKRYLVFRQFDVDKSGTMSSYEMRMALESAGKLTNHLFQLIILRYTEMTVDFDNFVTCLVRLETMMFA
>Gasterosteus_aculeatus_ENSGACG00000018991
QDYEGLKAQSLQSGRAFEDHLFPCCASSLGSKTSGVRWMRPPEFIVDGATRTDICQGGDCWLLAAIASLTLNDNLLHRVVPHGQGFQQGYAGIFHFQFWQFGEWVEVVIDDRLPVKDGKLLFVHSAEGTEFWSALLEKAYAKLNG
SYEALSGGSTSEGFEDFTGGVTEMFELNKAPLFSIISRAIERGSLLGCKLVKGHAYSVTGVEEYRGNTTKLVRIRNPWGEVEWTGAWSDREWDNVDRSVRARL-
EDGEFWMSYADFLREFSRLELCNLTADTLKWSSSLYQGEWRRGSTAFWLNPQFKIVLQHCSFLVGLMTIGFALYFFINLREVSSRLRLPPGEYVIVPSTFEPHKEGDFVLRVFSEEISLTELQTILNRIMSSCRSMISLMDSGKL
GLSEFHVLWEKIKRYLIFRQFDLDKSGTMSSYEMRMALDSAGKLTNQLFQLIILRYTEMSVDFDNFVTCLVRLETMMFA
>Oryzias_latipes_ENSORLG00000008280

-----------------------------------------
PEFIVDGATRTDICQGGDCWLLAAIASLTLNDNLLHRVVPHGQCFKQGYAGIFHFQFWQFGEWVDVVIDDRLPAKDGKLLFVHSVEGTEFWSALLEKAYAKLNGCYEALSGGSTSEGFEDFTGGVTEMYDLIKPPLFGIISRAVE
RGSLLGCKLVKGHAYSLTGVDEVMGQETQLGRLENFFGKTPWGGLKPLKEWSYVDSSVRDRL-
EDGEFWMAFSDFLREFSRVEICNLTADALKWSSSLFQGEWRRGSTAFWLNPQFKISLQHCSFLVGLMTIGFALYFFINLREVSSRLRLPAGEYIVVPSTFEPNKEADFVLRVFSEEISVTELQNILNKIISACRSMINLMDSGKL
GLKEFHVLWEKIKRYLIFRQFDLDKSGTMSSYEMRIALESAGKLTNHLFQLIILRYTEMSVDFDNFVTCLVRLETM---
>Danio_rerio_ENSDARG00000055338
QDFESLRTRCLQSGHLFEDDIFPAQQSSLGSKTRGVRWMRPPHFIVDGATRTDICQGGDCWLLAAIACLTLNEPLLRRVVPHGQSFHQQYAGIFHFQFWQFGDWVDVVIDDRLPVRDGKLLFVHSAEGSEFWSALVEKAYAKLNG
CYEALSGGSTSEGFEDFTGGVTEMYELKNAPLFSIITRAVERGSLMGCKLVKGHAYSITGVDEYRRSQTKLLRIRNPWGEVEWTGPWSDKEWREIDASVRSRL-
EDGEFWMAFSDFKREFSRLEICNLTADALKWNSSLYPGEWRRGSTAFWINPQFKIVLKECTFLVALMTIGFAIYFFINLREVSSRFSLPAGEYIIVPSTFEPQKEGDFVLRVFSEEINVAKLEMILNRVVSTCRGMINLMDTGKL
GLTDFHVLWEKFKRYLVFREFDIDKSGTMSSYEMRLALESAGKLTNNLFQLIILRYAKLNVDFDNFVACLIRLETMMFT
>Chiloscyllium_punctatum_contig39834
---------------------------------------------------------------------------------------------
FHFQFWQFGEWVEVVIDDRLPVKDGKLVFVHSAARNEFWSALLEKAYAKLNGCYEALSGGSTSEGFEDFTGGVTEMYDLRKSPLYQIIEKALQRGSLMGCKLVKGHAYSLTGAREARGGLVKLVRLRNPWGEVEWTGAWSDSEWN
SVDSSDCSSM-EDGEFWMSFGDFLREFNRLEICNLTPDTLNWSTSLYEGSWRRGSTAFWINPQFKIELKECSFLVALMTIGFAIYFFINLREVSTRFKLPAGEYIIVPSTFEPNKEADFCLRVFSE----------------
TCRSQ----------------------------------------------------------------------------------------
>Gallus_gallus_ENSGALG00000010186
QDYEALKQECIESGTLFRDPQFPAGPTALGSKTRGVEWKRPPQFIVGGATRTDICQGGDCWLLAAIGSLTLNEELLHRVVPHGQSFQEDYAGIFHFQIWQFGEWVDVVVDDLLPTKDGELLFVHSAECTEFWSALLEKAYAKLNG
CYESLSGGSTTEGFEDFTGGVAEMYDLKRAPMGHIIRKALERGSLLGCKLVKGHAYSVTAFKDYRGQQEQLIRIRNPWGQVEWTGAWSDSEWDNIDPSDREEL-
EDGEFWMSFRDFMREFSRLEICNLTPDALRWHTQVFEGTWRRGSTAFWINPQFKIKLLECSFLVALMTIGFAVYFFINLREVSNQIRLPPGEYIVVPSTFEPHKEADFILRVFTEEISVFELKTILNRVIASCRNMVNLMDSARL
GLVEFQILWNKIRSWLIFRQYDLDKSGTMSSYEMRMALESAGKLNNKLHQVVVARYADTGVDFDNFVCCLVKLETMMCG
>Meleagris_gallopavo_ENSMGAG00000010826
QDYEALKQECIESGVLFRDPQFPAGPTALGSKTRGVEWKRPPQFIVGGATRTDICQGGDCWLLAAIGSLTLNEELLHRVVPHGQSFQEDYAGIFHFQIWQFGEWVDVVVDDLLPTKDGELLFVHSAECTEFWSALLEKAYAKLNG
CYESLSGGSTTEGFEDFTGGVAEMYDLKRAPMGHIIRKALERGSLLGCKLVKGHAYSVTAFKDYRGQQEQLIRIRNPWGQVEWTGAWSDSEWDNIDPSDREEL-
EDGEFWMSFRDFMREFSRLEICNLTPDALRWHTQVFEGTWRRGSTAFWINPQFKIKLLECSFLVALMTIGFAVYFFINLREVSNQIRLPPGEYIVVPSTFEPHKEADFILRVFTEEISVFELKTILNRVIASCRNMVNLMDSARL
GLVEFQILWNKIRSWLIFRQYDLDKSGTMSSYEMRMALESAGKLNNKLHQVVVARYADTGVDFDNFVCCLVKLETMMCG
>Taeniopygia_guttata_ENSTGUG00000008009
----------------------------
LGSKTRGVEWKRPPQFIVGGATRTDICQGGDCWLLAAIGSLTLNEELLHRVVPHGQSFQEDYAGIFHFQIWQFGEWVDVVVDDQLPTKDGELLFVHSAECTEFWSAVLEKAYAKLNGCYESLSGGSTTEGFEDFTGGVAEMYDLK
RPPMGHIIRKALERGSLLGCKLVKGHAYSVTAFRDYRGQQEQLIRIRNPWGQVEWTGAWSDAEWNNIDPDEREEL-
EDGEFWMSFRDFMREFSRLEICNLTPDALRWHTQVFEGTWRRGSTAFWINPQFKIKLLECSFLVALMTIGFAVYFFINLREVSNQIRLPPGEYIIVPSTFEPHKEADFVLRREREEISVFELRTILNRVISSCRNMVNLMDSARL
GLVEFQILWNKIRSWLIFRQYDLDKSGTMSSYEMRMALESAGKLNNKLHQVVVARYADMGVDFDNFVCCLLKLETMMCG
>Anolis_carolinensis_ENSACAG00000000755
QDYETLKQECLESGRLFEDPQFPAIPSVLGSKTQGVRWKRPPQFIVGGATRTDICQGGDCWLLAAIGSLTLNEDLLHRVVPHGQSFQEDYAGIFHFQIWQFGEWVDVVVDDRLPTKDGELVFVHSAECQEFWSALLEKAYAKLNG
SYEALSGGSTTEGFEDFTGGVAEMYDLKKPPIAQIICKALERGSLLGCKLVKGHAYSVTGFKNYRGQQESLIRIRNPWGQVEWTGAWSDSEWNEVDPDQREEL-
EDGEFWMSFREFMRQFSRLEICNLTPDALRWHTTLFEGTWRRGSTAFWINPQFKIKLLECTFLVALMTIGFAVYFFINLREVSNHIRLPPGEYIIVPSTFEPNKEADFVLRVFTEEISVFELRTVLNRVIASCRNMVNLMDSARL
GLVEFQILWNKIRSWLIFRQHDLDKSGTMSAYEMRLALETAGKLDNKLHQVLVARYADLGVDFDNFVSCLVKLETMMCG
>Pelodiscus_sinensis_ENSPSIG00000016903
QDYEALKQQCLENGTLFEDPQFPAVPSVLGSKTQGLRWKRPPQFIVGGATRTDICQGGDCWLLAAIGSLTLNEDLLHRVVPHGQSFQEDYAGIFHFQIWQFGEWVDVVVDDRLPTKDGELVFVHSAECTEFWSALLEKAYAKLNG
SYEALSGGSTTEGFEDFTGGVSEMYDLKQPPLSRIIHKALERGSLLGCKLVKGHAYSVTGFSEYRGRQEELIRIRNPWGQVEWTGAWCDSEWNNVDPSQREEL-
EDGEFWMAFRDFMREFNRLEICNLTPDALKWHTTLFEGSWRRGSTAFWINPQFKIKLLECSFLVALMTIGFAVYFFINLREVSNKIRLPPGEYLIVPSTFEPHKESDFVLRVFTEEISVFELRTVLNRVITSCRNMVNLMDSARL
GLVEFQILWNKIRSWLIFREHDLDKSGTMSAYEMRMALESAGKLNNKLHQVVVARYADLGVDFDNFVCCLVKLEAMMWG
>Takifugu_rubripes_ENSTRUG00000008487
QDYEALKQQCVESGCLFEDPCFPAEPPSLGSKTQGVEWQRPPQFIVGGATRTDICQGGDCWLLAGIASLNLNERLLHRVVPHGQSFQDDYAGIFHFQFWQFGEWVDVVIDDRLPVKDGEVMFVHSAEGNEFWSALLEKAYAKLNG
SYEALSGGSTTEGFEDFTGGVSEMYELRSAPLPKIISKALDRGSLLGCKLVKGHAYSVTGLKEFRGRMERLIRVRNPWGQVEWTGAWSDSEWNEIDPSEREDL-
EDGEFWMSFNEFKKQFSRIEICNLTPDALHWNTMTYSGMWRRGSTAFWINPQFKITLLECTFLVALMTIGFAVYFFINLREVSTRLRLPPGEYLIVPSTFEPHKEADFVLRVFTEEISVHELKTILNRVVTSCRTMVNLMDTAHL
GLVEFQILWNKIRKWLIFRQFDLDKSGAMSSYEMRLAVEAAGKLNNKLNQILVARYAEM-INFDNFICCLVKLEAMMCG
>Oryzias_latipes_ENSORLG00000011738
QDFESLKRDCLESGCLFEDPCFPAEPPSLGSKTRGVEWIRPPQFIVGGATRTDICQGGDCWLLAAIASLTLNERLLHRVVPHGQSFQEDYAGIFHFQFWQFGEWVDVVIDDRLPVKDGELMFVHSAEGNEFWSALMEKAYAKLNG
SYEALSGGSTTEGFEDFTGGVSEMYELRKAPLHRIISKALERGSLLGCKLVKGHAYSVTGLKEYRGNMVRLIRIRNPWGQVEWTGAWSDHEWNDVDPSDREDL-

EDGEFWMSFSDFLRQFSRLEICNLTPDALHWNTMKFYGTWRRGSTAFWINPQYKITLLECSFLVALMTIGFALYFFINLREVSARLRLPPGEYLVVPSTFEPSKEADFVLRVFTEEISIRELKTILNRVISSCRTMVNLMDSARL
GLLEFQILWNKIRKWLIFREFDLDKSGAMSSYEMRLAVEGAGKLNNQLNQILVARYADM-VDFDNFICCLVKLEAMMCG
>Xiphophorus_maculatus_ENSXMAG00000005878
QDYEALKQECVEAGCLFEDPCFPAEPPSLGSKTRDVEWMRPPQFIVGGATRTDICQGGDCWLLAAIGSLTLNERLLHRVVPHGQSFQDDYAGIFHFQFWQFGEWVDVVIDDRLPVKDGELMFVHSAEGNEFWSALLEKAYAKLNG
SYEALSGGSTTEGFEDFTGGVSEMYELRKAPLYRIISKALERGSLLGCKLVKGHAYSVTALKEYAGNMVRLIRIRNPWGQVEWTGAWSDSEWDEIDPSEREDL-
EDGEFWMSFSDFLRQFSRLEICNLTPDVLHWNTMKFYGTWRRGSTAFWINPQYKITLLECSFLVALMTIGFAVYFFINLREVSTRLRLPPGEYLIVPSTFEPGKEADFVLRVFTEEISIRELRTILNRVVTSCRTMVNLMDSARL
GLVEFQILWNKIRKWLTFREFDLDKSGAMSSYEMRLAVEAAGKLNNRLHQILVARYAEL-IDFDNFICCLVKLEAMMCG
>Oreochromis_niloticus_ENSONIG00000009965
QDYEALKQECLESGCLFEDPYFPAEPPSLGSKTKDVEWMRPPQFIVGGATRTDICQGGDCWLLAAIASLTLNEKLLHRVVPHGQSFQDDYAGIFHFQFWQFGEWVDVVIDDRLPVKDGELMFVHSAEGNEFWSALLEKAYAKLNG
SYEALSGGSTTEGFEDFTGGVSEMYELRRAPLYRIIGKALERGSLLGCKLVKGHAYSVTGLKEFHGNNERLIRIRNPWGQVEWTGAWSDPEWDQIDPSEREDL-
EDGEFWMSFSDFIRQFSRLEICNLTPDALHWNTIKFYGTWRRGSTAFWINPQYKITLLECSFLVALMTIGFALYFFINLREVSTRLRLPPGEYLIVPSTFEPSKEADFVLRVFTEEISIRELRTILNRVVTSCRTMVNLMDSARL
GLVEFQILWNKIRKWLIFRQFDLDKSGAMSSYEMRLAVEAAGKLNNRLNQILVARYAEM-IDFDNFICCLVKLEAMMCG
>Gasterosteus_aculeatus_ENSGACG00000004833
QDYETLKQECLESGCLFEDSCFPAEPPSLGSKTKDVVWMRPPQFIVGGATRTDICQGGDCWLLAAIGSLTLNERLLHRVVPHGQSFQDDYAGIFHFQFWQFGDWVDVVIDDRLPVKDGELMFVHSAEGNEYWSALLEKAYAKLSG
SYEALSGGSTTEGFEDFTGGVSEMYELRSAPLHRIIGKALERDSLLGCKLVKGHAYSVTGMKEFRGNMERLIRIRNPWGQVEWTGAWSDPEWNEIDPSEREDL-
EDGEFWMSFSEFKRQFSRLEICNLTPDTLKWHTMKFYGAWRRGSTAFWINPQYKITLLECSFLVALMTIGFALYFFINLREVSTRLRLPPGEYLIVPSTFEPSQEADFVLRVFTEEISVRELRTILNRVVCSCRTMVNLMDSARL
GLVEFQILWNKIRKWLIFRDFDLDKSGAMSSYEMRLAVEAAGKLNNRLNQILVARYAEM-VDFDNFICCLVKLEAMMCG
>Danio_rerio_ENSDARG00000052702
QDYEALRQECLEGGYLFEDPCFPAEPPSLGSKTRDVEWMRPPQFIVGGATRTDICQGGDCWLLAAIGSLTLNERLLHRVVPHGQSFQDDYAGIFHFQFWQFGEWVDVVIDDRLPVKDGELMFVHSAEGNEFWSALVEKAYAKLNG
SYEALSGGSTTEGFEDFTGGVSEMYELRKAPLYRIISKALDRGSLLGCKLVKGHAYSVTALKQYRGRMERLIRIRNPWGQVEWTGAWSDPEWDEIDPSEKDDL-
EDGEFWMSFGEFLRQFSRLEICNLTPDALHWNTIKFHGAWRRGSTAFWINPQYKITLLECSFLVALMTIGFAIYFFINLREVSTRLRLPPGEYIIVPSTFEPSKEADFVLRVFTEEISVHELKTILNRVVSSCRTMVNLLDSARL
GLVEFQILWNKIRKLLIFREFDIDKSGTMSSYEMRLAVESAGKLNNRLNQILVARYAEA-IDFDNFVCCLIKLEAMMCG
>Xiphophorus_maculatus_ENSXMAG00000008745
---------------------------------------------------------
GDCWLLAAIGSLTLNQQLLHRVVPHGQDFSHLYAGIFHFQFWQFGKWVDVVIDDRLPVKDGELLFVHSAEGSEFWSALLEKAYAKLNGSYEALSGGSTTEGFEDFTGGVAEMYELKKAPLYRIIGKALERGSLLGCKLVKGHAYS
VTGVRQYRGRQERLIRIRNPWGQVEWTGAWSDSEWNAIDSAEKDEM-
EDGEFWMSFQEFLHQFSRLEICNLTPDALFWTTVTYEGSWRKGSTAFWINPQYKITLLECSFLVALMTIGFAIYFFINLREVSSRFRLPPGEYLIVPSTFEPSKEADFVLRVFTEEISVRELRTVLNRVVSSCRAMVSLMDSARL
GLLEFQILWNKIRKWLIFREFDLDKSGCMNSYEMRLALENGGKLNNKLYQMLIARYADI-IDFDNFTCCLVKLETMMCG
>Oreochromis_niloticus_ENSONIG00000007243
QDFEALRQDCLQNRTLFEDPMFPAEPGSLGTKTRGVEWKRPPQFIVGGATRTDICQGGDCWLLAAIGSLTLNEQLLHRVVPHGQSFSHQYAGIFHFQFWQFGQWVDVVIDDRLPVKDGELLFVHSAEGTEFWSALLEKAYAKLNG
SYEALSGGSTTEGFEDFTGGVSEMYELKKAPLHRIISKALERGSLLGCKLVKGHAYSVTGLRQYRRQRELLIRIRNPWGQVEWTGAWSDSEWNAIDSAEKDEM-
EDGEFWMSFQEFLRQFSRLEICNLTPDALFWNTATYEGSWRRGSTAFWINPQYKISLLECSFLVALMTIGFAIYFFINLREVSGRFRLPPGEYLIVPSTFEPSKEADFVLRVFTEEISVRELRTILNRVVSSCRAMVSLMDSARL
GLLEFQILWNKIRKWLIFREFDLDKSGCMNSYEMRLALENGGKLNNKLHQMLVARYADI-IDFDNFTCCLVKLEAMMCG
>Gasterosteus_aculeatus_ENSGACG00000019940
QNFQDLKEDCQQNGFLFQDPVFPAEPASLGTKTRGVEWKRPPQFIVGGATRTDICQGGDCWLLAAIGSLTLNERLLHRVVPHGQNFSQEYAGIFHFQFWQFGEWVDVVIDDRLPVKDGELMFVHSAEGSEYWSALLEKAYAKLNG
SYEALSGGSTTEGFEDFTGGVSEMYELRKAPLHRIISKALERGSLLGCKLVKGHAYSVTALRQYHGNMEKLIRIRNPWGQVEWTGAWSDSEWTSVDPAEKDEL-
EDGEFWMSFQDFLRQFSRLEICNMTPDALFWTTTMFEGGWRRGSTAFWINPQYKVSLLECSLLVALMTIGFAIYFFINLREVSARLRLPPGXXXXXXXXXXPSKEMNFDL-----
EISVQELRTVLNRVMSSCRAMVSLMDSARLGLLEFQVLWNKIRKWLIFREFDLDKSRCMNAYEMRLALGEGGKLNNKLYQMLVARYADI-IDFDNFTCCLVKLEAMMCG
>Scyliorhinus_canicula_TranscriptomeContig69801
------------------------------------------------------------------------------------------------------------------------
MFVHSAENNEFWSALLEKAYAKLSGSYEALSGGSTTEGFEDFTGGVAEMYDLGSAPLYTIIQKAVDRGSLLGCKLVKGHAYSVTGVKEYRGRKERLIRIRNPWGQVEWTGAWSDSEWNQIDEDERDGM-
EDGEFWMAFQEFLKQFSRLEICNLTPDTLKWNMSAFNGSWRRGSTAFWINPQFKITLEECSFLCALMTIGFAVYFFINLREVSNRIKLPPGEYVIVPSTFDPSQEADFVLRVFTEEISVFELRTILNRVLSSCRGMVNLLDSGRL
GVLEFQIMWNKIRKWLIFRQYDLDKSGTMSSYEMRLALESAGKINNRIHQLIVGRYAEA-LDFDNFISCLVRLEAMMSG
>Chiloscyllium_punctatum_contig12303
QDYEALKQECLENGTLFEDPQFPAVPTSIGSKTRGIIWKRPPQFILAGASRTDVCQGGDCWLLAAIASLTLNEKVLHRVVPHGQNFQEEYAGIFHFQFWQFGEWVDVVIDDRLPTKDGELVFVHSAENNEFWSGLLEKAYAKLNG
SYEALSGGSTTEGFEDFTGGVAEMYELRNAPLYTIIRKALDRGSLLGCKLVKGHAYSVTGVKEYRSRMERLIRIRNPWGQVEWTGAWSDSEWNYIDQEEREGM-
EDGEFWMAFPEFLRQFSRLEVCNLTPDTLKWNMAVFNGSWRRGSTAFWINPQFKIKLEECSFLCALMTIGFAIYFFINLREVSNRIKLPPGEYVIVPSTFDPSKEADFVLRVFAEEISVFELRTILNRVLSSCRGMVNLLDSGRL
GVVEFQIMWNKIRKWLIFRQYDLDKSGTMSSYEMRLALESAGKINNRIHQVIVGRYSEV-LDFDNFISCLVKLEAMMSG
>Callorhinchus_milii_TranscriptomeContig20333

QDYGVLRGECLENCCLFEDPQFPAIASSIGTKTRGIVWQRPPQFITDGASRTDVCQGGDCWLLAAIASLTLNNEVLHRVVPHGQSFQEEYAGIFHFQFWQYGEWVDVVIDDQLPTKDGELVFVHSAENNEFWSALVEKAYAKLNG
SYEALSGGSTTEGFEDFTGGVAEMYQLNKAPLYSVIRKAVERGSLLGCKLVKGHAYSVTGVKEYRGRQVRLIRIRNPWGQVEWTGSWSDSEWDHIGDDERDGM-
EDGEFWMDFSEFLRQFSRLEICNLSPDALKWHTAVYDGCWRRGSTAFWINPQFKITLSECSFLCALMTIGFAIYFFINLREVSSRIRLPPGEYIIVPSTFEPRQEADFVLRVFTEEISVFELKTILNRVLSSCRTMVNLMDSARL
GILEFQVLWNKIRKWLIFRQFDLDKSGTMSTYEMRLALEASGKLNNRILQSIVGRYADT-IDFDNFIGALVKLEVMMSS
>Leucoraja_erinacea_TranscriptomeContig63790
-------------------------------------------------------------------------------------------------------------------------------------------------
-----------------------------------MRKALERGSLLGCKLVKGHAYSVTGVKEYRGRVERLIRIRNPWGQVEWTGAWSDSEWDAIDPEEREGM-
EDGEFWMSWGEFLRQFSRLEICNLGPETLKWQSTTFRGSWRRGSTAFWINPQFRLTLCECSFLCALMTIGFALYFFINMREVSSRLSLPPGDYIIVPSTFEPSHEADFVLRVFTEEISVFELRTILNRVLASCRGMINLLDSGRL
GLLEFQRLWNKIRAWLIFRNFDLDKSGTMNSYEMRLALETAGKINNHIHQAIASRYADV-IDFDNFISCLVRLEAMMSG
>Ornithorhynchus_anatinus_ENSOANG00000009824
---------------------------------------------------------
GDCWLLAAIGSLTLNEELLHRVVPHGQSFQEEYAGIFHFQIWQFGEWVDVVIDDRLPTKDGELVFVHSAEGSEFWSALLEKAYAKLNGSYEALSGGSTTEGFEDFTGGVAEMYELRRPPLLHIIRKALDRGSLLGCKLVKGHAYS
VTGLREYQNRQEPLIRIRNPWGQVEWTGAWSDNEWNEIDPDERDQL-
EDGEFWMSYQSLIDALELVDIVSISFYLLSYFYVGTDPPRRHPRTSFWINPQFRLRLLECSCLVALMTIGFAIYFFINLREVSAHVRLPPGEYVVVPSTFEPHQEADFVLRVFTEEISVFELRKILNRVVTSCRNMVNLMDSARL
GLVEFQILWNKIRKWLIFRQHDLDKSGTMSSYEMRLAVESAGKLNNKLQQVLVARYADMGIDFDNFVSCLVKLEAMMCG
>Lepisosteus_oculatus_GENSCAN00000007636
QDYDALKQECIESGCLFEDPLFPAEPPSLGSKTRGVEWMRPPQFIVGGATRTDICQGGDCWLLAAIASLTLNEKLLHRVVPHGQSFQEDYAGIFHFQFWQFGEWVDIVVDDRLPVKDGELLFVHSAEGNEFWSALVEKAYAKLNG
SYEALSGGSTTEGFEDFTGGVSEMYELRKAPLYRIISKALERGSLLGCKLVKGHAYSVTGLREYRGRSERLIRIRNPWGQVEWTGAWSDSEWNEIDPSERDDM-
EDGEFWMAFQEFLRQFSRLEICNLTPDALFWNTIKFEGSWRRGSTAFWINPQYKITLLECSFLVALITIGFAIYFFINLREVSSRLRLPPGEYLIVPSTFEPSKEADFVLRVFTEEISVRELQTILNRV----------------
----------------IFRQYDLDKSGTMSSYEMRLALEPAGKLNNKLNQVLVARYADT-IDFDNFICCLVKLEAM---
>Xenopus_tropicalis_ENSXETG00000031732
QDFDKLRAQCLASGTLFSDEEFPACPSSLGSKTNGIVWKRPPQFIVDGATRGDIRQGGDCWLLAAIASLTLDPDLVAQVVPENQSFQKGYAGIFHFQFWQYGEWVDVVVDDRLPMKDGNLVFVHSAEGDEFWSALLEKAYAKLNG
SYEALTGGLTIEGFEDFTGGIAEVYELNAAPLFQIIQKALRAESLLGCKLVKGHAYSVTGAEEYRGRREKLIRVRNPWGEVEWTGPWSDPEWNYIDPKVKAAL-
DDGEFWMAFSDFLREYSRLEICNLSPDTLKWNITLYNGSWARGSTAFWTNPQFRIKLDTCTVIVGLMSIGFSIFFYINIREVSQRFYLPVGDYLIVPTTFEPFQNGDFCLRIFSEELDATDLQSILNVLLSTCREMISLQDTETL
NLREFRVLWMKIQKYMIYLRADSDRSGTIDALELRTALPQAGTLNNKIQQRIVKRYASLAINLDAFIACMMRLETLVTP
>Xenopus_tropicalis_ENSXETG00000018607
QDFEKLRAQCLASGTLFKDEEFPACPSALGSKTQGVIWKRPPQFIVDGATRGDIRQGGDCWLLAAIASLTLDADLVAQVVPENQSFQKDYAGIFHFRFWQYGEWVDVVVDDRLPTKNGNLVFVHSAEGDEFWSALLEKAYAKLNG
SYEALSGGSTIEGFEDFTGGIAEVYELRKAPLFQIIQKALRAESLLGCKLVKGHAYSVTGAEEYRGRQEKLIRVRNPWGEVEWTGPWSDPEWNYIDPKVKAAL-
DDGEFWMAFSDFLREYSRLEICNLSPDTLKWNITLYNGSWARGSTAFWTNPQFRIKLDECTVIVGLMSIGYSLFYYINVREVSNRF-
LPVGDYLIVPSTFEPFKNGDFCLRVFSEEIDAKELQTILNKLISTCREMISLQDTATLSLLEFRILWLKIQKYLIYLKADSDRSGTMDAHELRAALQEAGTLNNKIQQSIVLRYASLTINFDGFIACMMRLETLVYI
>Meleagris_gallopavo_ENSMGAG00000008330
QDYEALRQKCLQAGTLFKDEEFPACPSALGSKTQGIVWKRPPQFIVGGATRTDVCQGGDCWLLAAIASLTLNPDVLHRVVPEAQSFQEDYAGIFHFQFWQYGEWVDVVVDDRLPTKNGELLFVHSEEGNEFWSALLEKAYAKLNG
SYEALAGGSTIEGFEDFTGGISESYDLRRAPLYQIVQKALRAGSLLGCKLVKGHAYSVTGAEEYQGQPEKLVRLRNPWGEVEWTGAWSDREWNYIDPKQKQAL-
DDGEFWMAFSDFQRQFTRLEICNLTPDTLKWDLTVFNGQWIRGSTAYWINPQFKIRLDECTILVGLMSIGYSLYFYINLREVSSRIKLPKGEYLIVPSTFEPYKNGEFCLRVFSEEVTATELQTILNRVLATCREMISLLDTGSL
GLIEFKTLWMKIQMYLIYRKVDRDYSGTIDSHEMRNALSEAGVLNNQVQQSIAIRYACMTIDFDGFVACMIRLETLLV-
>Pelodiscus_sinensis_ENSPSIG00000014086
QDYEVLRQQCLQSGSLFKDEEFPASPSSLGSKTQGIVWKRPPQFIVGGATRTDIRQGGDCWLLAAIASLTLDQEILQRIVPEDQSFQKNYAGIFHFQFWQFGEWVDVVVDDRLPTKNGQLLFLHSEEGNEFWSALLEKAYAKLNG
SYEALAGGSTVEGFEDFTGGIAESYDLRKAPLYQIIRKALQSGSLLGCKLVKGHAYSLTGAEEYRGRLEKLVRIRNPWGEVEWTGAWSDPEWNYVDPKQKQAL-
DDGEFWMAFSDFQRQFTRLEICNLTPDTLKWGLTLFSGQWRRGSTAYWINPQFKIRLDECTVLVGLMTIGYSLYFYVNLREVSGRIKLPQGEYLVVPSTFEPFKDGEFCLRVFSEEMSADELQTVLNRVLTTCREMISLLDTGTL
GLVEFKILWLKIQKYLIYKKVDTDYSGTIDAHEMRDALREAGTLNNKVQHIIATRYVNLTIDFDSFVACMIRLETLMV-
>Anolis_carolinensis_ENSACAG00000002512
QDFEELRRQCLKSGTLFKDEEFPACPSALGSKTQGIIWKRPPQFIVGGATRTDIRQGGDCWLLAAIASLTLDQEILDRVVPKDQSFQKDYAGIFHFQIWQYGEWVDVVVDDRLPTKNGQLLFLHSEEGNEFWSALLEKAYAKLNG
SYEALTGGSTIEGFEDFTGGIAESYDLRKAPLYQIIQKALRSGSLLGCKLVKGHAYSVTGAEEYRGQPVKLLRIRNPWGEVEWTGAWSDPEWNYIDPKEKNAL-
DDGEFWMSLADFQRQFTRLEICNLTPDTLKWGMTLFNGHWRRGSTAYWTNPQFKISLDECTVLIGLMSIGYSIYFYVNLREASNRFHLPRGEYLVVPSTFEPFKDGEFCLRVFSEEMTANELQTILNRVITTCREMISLLDTGSL
GVAEFKILWMKIQRYLIYKKVDTDHSGTIDAHEMRDALKEAGTLNNKVQHTIAARYACLTIDFDGFLACMIRLETLLYA
>Ornithorhynchus_anatinus_ENSOANG00000001449
QDFEALRKQCLKSGTLFKDVEFPACPSALGSHTRGIVWKRPPQFIVGGATRTDIRQGGDCWLLAAIASLTLNEELLYRVVPRNQSFQKDYAGIFHFQFWQYGEWVDVVIDDRLPTRNGNLLFLHSEEGSEFWSALLEKAYAKLNG
SYEALTGGSTVEGFEDFTGGISEFYELKKAPLYQIIQKALRSGSLLGCKLVKGHAYSVTGAEEHHGRPEKLIRLRNPWGEVEWTGAWSDPEWNFIDPKQKEEL-

EDGEFWMSFTDFQRQFSRLEICNLSPDSLKWGLTLFNGRWIRGSTAYWTNPQFKICLDECTVLLGLMRIGYSIYFYVNLREVSGRVQLPQGEYLVVPSTLEPFKDGEFCLRVFSERIQVHELRDILNETFSTCREMISLLDLGDL
GLLEFKTLWLKILKYQIFLEVDTNHSGTIRAHEMRTALKEAG-------------------------------------
>Homo_sapiens_NM_001143962
QDFKTLRQQCLDSGVLFKDPEFPACPSALGSQTQGIIWKRPPQFIVGGATRTDICQGGDCWLLAAIASLTLNEELLYRVVPRDQDFQENYAGIFHFQFWQYGEWVEVVIDDRLPTKNGQLLFLHSEQGNEFWSALLEKAYAKLNG
CYEALAGGSTVEGFEDFTGGISEFYDLKKPPLYQIIRKALCAGSLLGCKLVKSHAYSVTGVEEFQGHPEKLIRLRNPWGEVEWSGAWSDPEWNHIDPRRKEEL-
EDGEFWMSLSDFVRQFSRLEICNLSPDSLKWNLVLFNGHWTRGSTAYWTNPQFKIRLDECTVLLGLMSIGYAVYFYVNLREVSGRARLPPGEYLVVPSTFEPFKDGEFCLRVFSEEITANALKILLNEAFSTCREMISLLDTGTL
GAVEFKTLWLKIQKYLIYWETDYNHSGTIDAHEMRTALRKAGTLNSQVQQTIALRYACLGINFDSFVACMIRLETLLV-
>Mus_musculus_ENSMUSG00000038599
QDFETLRKQCLNSGVLFKDPEFPACPSALGSETQGIIWKRPPQFIVGGATRTDIRQGGDCWLLAAIASLTLNEKLLYRVVPRDQSFQKNYAGIFHFQFWQYGEWVEVVIDDRLPTKNGQLLFLHSEEGNEFWSALLEKAYAKLNG
SYEALAGGSTIEGFEDFTGGISEFYDLRKPPLYYTIQKALRKGSLLGCKLVKGHAYSVTGVEEFRGLPEKLIRLRNPWGEVEWTGAWSDPEWNYIDPQKKGEL-
EDGEFWMSFSDFLKQFSRLEICNLSPDSLKWNLVLFNGRWTRGSTAYWTNPQFKIHLDECTVLLGLMSIGYAVYFYMNLREVSSRVQLPPGQYLVVPSTFEPFKDGDFCLRVFSEEISAHQLKRVLNGLLSTCREMISLLDTGSL
RPVEFKTLWLKICKYLIYQEMDHSRAGTIDAHEMRTALKKAGTLNNQVQQTIATRYACLGVDFDGFVACMIRLEILLV-
>Sarcophilus_harrisii_ENSSHAG00000014684
QDYETLRKQCLETGTLFKDPEFPACPSALGSQTQGIVWKRPPQFIVGGATRTDVRQGGDCWLLAAIASLTLNEGLLYRVVPKGQSFQKNYAGIFHFQFWQYGEWVDVVIDDRLPTRNGNLLFLHSEEGREFWSALLEKAYAKLNG
SYEALTGGSTVEGFEDFTGGISEFYDLKKAPLYQIIRKALRSGSLLGCKLVKGHAYSVTGVEEFHGRLEKLIRLRNPWGGTEWKGAWSDPEWNYIDPRQKEEL-
EDGEFWMSFSDFQRQFSRLEICNLSPDSLKWNTALFNGRWIRGSTAYWINPQFKIRLDECTVLVGLMSIGYSLYFYFNLREVSNRFHLPLGEYLVVPSTFEPFKDGEFCLRIFSEDISAKALQTILNGFFFTCREMIGLLDTGTL
GLVELKILWLKIRKYLIYQEVDANHSGTINTHEMRTAFKKTGNLNNQVQQAIAVHYGDLSIPFDRFISCMIRLESLFVV
>Monodelphis_domestica_ENSMODG00000005003
QDFETLRKQCLESGTLFKDPEFPACPSALGSQTQGIIWKRPPQFIIGGATRTDVRQGGDCWLLAAIASLTLNEELLYRVVPKDQSFQKNYAGIFHFQFWQYGEWVDVVIDDRLPTRNGNLLFLHSEEGREFWSALMEKAYAKLNG
SYEALTGGSTVEGFEDFTGGISEFYDLKKPPLYQIIRKALRSGSLLGCKLVKGHAYSVTGVEEYRGCLEKLIRLRNPWGGTEWTGAWSDPEWNYIDPRQKKEL-
EDGEFWMSFSDFLRQFSRLEICNLSPDSLKWNMTLFNGRWIRGSTAYWTNPQFKIRLDECTVLLGLMSIGYSLYFYINRREVSTRIHLPVGEYLVVPSTFEPFKDGEFFLRIFSEEISANGLQTILNDFFSTCREMIGLLDTGTL
GLAELKILWLKIQKYLIYQEVDINHSGTINAHEMRMAFKKAGTLNNQVQQAIAVRYGGLNIHFDSFIACMMRLESLLV-
>Gallus_gallus_ENSGALG00000009360
QDFGALRRECLQGGRLFHDPSFPAGPAALGSKTKGVVWCRPPRFIAGGATRTDICQGGDCWLLAAIASLTLNEEILARVVPRDQSFQDEYAGIFHFQFWQYGEWVDVVVDDRLPTKNGELLFVHSAEGSEFWSALLEKAYAKLNG
SYEALSGGTTTEGFEDFTGGIAEWYELQKAPLFKIIQKALQKGSLLGCKLVKGHAYSVTGAEEFRGSIQKLIRIRNPWGEVEWTGKWNDPNWSGVDPEVRERL-
EDGEFWMAFNDFLRHYSRLEICNLTPDTLKWSLLKLDGNWRRGATAFWTNPQYLIKLEECTFLIGLITIGFAIYFFINLREVLNRFKLPAGEYIIVPSTFEPNLNGDFCLRVFSEEISAFELRNILNKILATCKIMVDLLDSGKL
GLKEFHTLWTKIQKYQIYREIDVDRSGTMNSYEMRRALEAAGKLNCQLHQIIVARFADLIIDFDNFVRCLIRLETLVI-
>Meleagris_gallopavo_ENSMGAG00000008632
------------------------------
SKTKGVVWCRPPRFIAGGATRTDICQGGDCWLLAAIASLTLNEEILARVVPRDQSFQDEYAGIFHFQFWQYGEWVDVVVDDRLPTKNGELLFVHSAEGSEFWSALLEKAYAKLNGSYEALSGGTTTEGFEDFTGGIAEWYELQKA
PLFKIIQKALQKGSLLGCKLVKGHAYSVTGAEEFRGSIQKLIRIRNPWGEVEWTGKWNDPNWSGVDPEVRERL-
EDGEFWMAFNDFLRHYSRLEICNLTPDTLKWSLLKLDGNWRRGATAFWTNPQYLIKLEECTFLIGLITIGFAIYFFINLREVLNRFKLPAGEYIIVPSTFEPNLNGDFCLRVFSEEISAFELRSILNKILATCKIMVDLLDSGKL
GLKEFHTLWTKIQKYQIYREIDVDRSGTMNSYEMRRALEAAGKLNGQLHQIIVARFADLIIDFDNFVRCLIRLETLII-
>Anolis_carolinensis_ENSACAG00000003199
QDFGALRAECLEAGGLFQDPCFPAAAPALGAKTKGIVWKRPPQFIVGGATRTDICQGGDCWLLAAIASLTLNEEILARVVPSDQSFQDRYAGIFHFQFWQYGEWTDVVVDDRLPTKNGELLFVHSAEGTEFWSALLEKAYAKVNG
SYEALSGGSTTEGFEDFTGGIAEWYELRKAPLFRIIQKALQKGSLLGCKLVKGHAYSVTGAEEFRGSLQKLIRIRNPWGEVEWTGKWNDPSWSNVDPEVRDRL-
EDGEFWMSFDDFLRHYSRLEICNLTPDTLKWSLSLMDGNWRKGSTAFWMNPQYLIKLEECTFLIGLITIGFAIYFFINLREVLNRFKLPSGEYIIVPSTFEPNKNGDFCLRVFSEEISAFELCGILKKVMATCKIMVDLLDSGKL
GLKEFHILWTKIQKYQIYREMDVDSSGTMNAYEMRKALEQAGKLDCQLHQVIVARFADLIIDFDNFVRCLIRLETLII-
>Pelodiscus_sinensis_ENSPSIG00000012616
QDFEALRDEC-------
QDPAFPAVPSSLGSKTQGITWKRPPQFIVGGATRTDICQGGDCWLLAAIASLTLNEEILARVVPKDQSFQDRYAGIFHFQFWQYGEWVDVVVDDKLPTKNGELLFVHSAEGSEFWSALLEKAYAKVNGSYEALSGGSTTEGFEDF
TGGIAEWYELQKAPLFKIIQKALLKGSLLGCKLVKGHAYSVTGAEECRGSMQKLIRIRNPWGEVEWTGKWNDSNWNSVAPEVRERL-
EDGEFWMSFSDFLRHYSRLEICNLTPDTLKWSLTKLDGNWRRGSTAFWMNPQYLIKLEECTFLVGLMTIGFGIYFFINLREVLNRFKLPPGDYVIVPSTFEPNKDGDFCLRVFSEEISAFELCNILRKIMATCKIMVDLLDSGKL
GLKEFHILWTKIQKYQIYREIDVDRSGTMNSYEMRRALEEAGKLTCQLHQVIVARFADLIIDFDNFVRCLIRLETL---
>Homo_sapiens_BC021303
QDYEALRNECLEAGTLFQDPSFPAIPSALGSKTRGIEWKRPPQFIIGGATRTDICQGGDCWLLAAIASLTLNEEILARVVPLNQSFQENYAGIFHFQFWQYGEWVEVVVDDRLPTKDGELLFVHSAEGSEFWSALLEKAYAKING
CYEALSGGATTEGFEDFTGGIAEWYELKKPPLFKIIQKALQKGSLLGCKLVKGHAYSVTGAEESNGSLQKLIRIRNPWGEVEWTGRWNDPSWNTIDPEERERL-
EDGEFWMSFSDFLRHYSRLEICNLTPDTLKWKLTKMDGNWRRGSTAFWMNPQYLIKLEECTFLVGLITIGFGIYFFINLREVLNRFKLPPGEYILVPSTFEPNKDGDFCIRVFSEEISAFELQTILRRVLATCKIMVDMLDSGKL
GLKEFYILWTKIQKYQIYREIDVDRSGTMNSYEMRKALEEAGKMPCQLHQVIVARFADLIIDFDNFVRCLVRLETLVL-

>Mus_musculus_ENSMUSG00000026509
QDYETLRNECLEAGALFQDPSFPALPSSLGSKTRGIEWKRPPQFIIGGATRTDICQGGDCWLLAAIASLTLNEEILARVVPPDQSFQENYAGIFHFQFWQYGEWVEVVVDDRLPTKDGELLFVHSAEGSEFWSALLEKAYAKING
CYEALSGGATTEGFEDFTGGIAEWYELRKPPLFKIIQKALEKGSLLGCKLVKGHAYSVTGAEESSGSLQKLIRIRNPWGQVEWTGKWNDPSWNTVDPEVRANL-
EDGEFWMSFSDFLRHYSRLEICNLTPDTLKWKLTKMDGNWRRGSTAFWMNPQYLIKLEECTFLVGLITIGFGIYFFINLREVLNRFKLPPGEYVLVPSTFEPHKDGDFCIRVFSEEISAFELQTILRRVLATCKIMVDMLDSGKL
GLKEFYILWTKIQKYQIYREIDVDRSGTMNSYEMRKALEEAGKLPCQLHQVIVARFADLIIDFDNFVRCLVRLETLVL-
>Ornithorhynchus_anatinus_ENSOANG00000001450
QDYESLKSDCLQSGKLFQDPSFPAITSSLGSKTQGIVWKRPPQFIVGGATRTDICQGGDCWLLAAIASLTLNEEVLSRVVPENQSFQDNYAGIFHFQFWQYGEWVEVVVDDRLPTKNGELLFVHSAEGSEFWSALLEKAYAKVNG
CYEALSGGATTEGFEDFTGGIAEWYELKKAPLFKIIQKALQKGSLLGCKLVKGHAYSVTGAEECRGSMEKLIRIRNPWGEVEWTGKWNDPNWNSINPEVKERL-
EDGEFWMSFRDFLRHYSRLEICNLTPDTLKWSLSKLDGNWRRGSTAFWMNPQYLIKLEECTFLVGLITIGFSIYFFINLREVLNRFKLPPGEYIIVPSTFEPNKDGDFCLRVFSEEISAFELQTILKKVLATCKIMVDLLDSGKL
GLKEFFILWTKIQNYQIYREIDADNSGTMNAYEMRKALEEAGKLPRQLHQVIIARFADLIIDFDNFVRCLVRLETLVL-
>Monodelphis_domestica_ENSMODG00000005094
QDYETLLSECLEEGKLFQDPHFPAIGSSLGSKTQGIVWKRPPQFIVEGATRTDICQGGDCWLLAAIASLTLNEEILARVVPKNQSFQDNYAGIFHFQFWQYGEWVDVVVDDRLPTKNDELLFVHSAEGSEFWSALLEKAYAKVNG
CYEALSGGATTEGFEDFTGGIAEWYELKKAPLFIIIQKALQKGSLLGCKLVKGHAYSVTGAEEFRGSLQKLIRIRNPWGEVEWTGKWSDPNWNSIDPDERERL-
EDGEFWMSFSDFLRHYSRLEICNLTPDTLKWRLTKMDGTWRRGSTAFWMNPQYMIKLEECTFLVGLITIGFSIYFFINLREVLNRFKLPAGEYVIVPSTFEPHKNGDFCLRVFSEEISAFELQTILRRVLATCKIMVDMLDSGKL
GLKEFYLLWSKIQKYQIYKEMDVDRSGTMNAYEMRKALEEAGKLPYQLHQVIVARFADLIIDFDNFVRCLVRLETLVL-
>Gadus_morhua_ENSGMOG00000018340
------------------------------------------------------------------------------------------------
FWQFGKWVDVVVDDRLPTKDGELLFVHSADSSEFWSALLEKAYAKLNGCYEALSGGSTIEGFEDFTGGIAEKHDLLKAGLFKIIRKALDRGSLLGCKLVKGHAYSVTGAEQYRGDQVQLIRVRNPWGEVEWTGRWSDMEWRYVGD
ADRDRL-
EDGEFWMSFSDFLCQYSMLEICNLTPDALKWALSEFEEGWRRGSTAFWKNPQFVIKLEECTVIVGLMFMGFAIYFFTNMREVCNRFCLPPGEYLIIPSTYEPNHNADFYLRVFTEEMSMFELQKVLNKVVMTCRNMITLLDTRKL
GLLEFKILWTKIEKLLLYKGKDADGNGFMSTTEMRLAVEEAGSLSSPLHQIIVARYSELTIDFDNFVCCVIRLEIL---
>Gasterosteus_aculeatus_ENSGACG00000004767
QDYETLRQECLKRGRLFEDDCFPAEPKSLGSKTRGVVWKRPPQFIKDGATTTDICQGGDCWLLAAIASLTLDQRILARVVPPGQSFSDDYAGIFHFQFWQFGEWVDVVVDDRLPTKDGELLFVHSAEGSEFWSALLEKAYAKING
CYEALSGGSTTEGFEDFTGGIAERHELKNADLFKIIKKALERGSLLGCKLVKGHAYSVTGAEQYRGDQVQLIRIRNPWGQVEWNGAWSDSEWRYVSDGDRKRL-
EDGEFWMSFSDFLRQYSRLEICNLTPDALKWAESEFEETWRRGVSAFWMNPQFVIKLEECTFIVGLMTIGFAIYFFINLREVSNHFCLPPGEYLIVPSTFEPNKNGDFYVRVFSEEISAFELQKILNRVVVTCHHMVNLLDSGKL
GLVEFKILWTKIEKFLVYKEKDANQSGCMNSSEMRMAVEEIGSLNTALHQIILARYSELTIDFDSFVCSMIRLESLLCG
>Gasterosteus_aculeatus_ENSGACG00000019985
QDYEVLRRRCSESGRLFQDETFPAHGSSLGSKTRGVSWERPPEFIVSGASRTDICQGGDCWLLAAIASLTLNEEVLARVVPHGQSFKGEYAGIFHFQFWQFGEWVDVVIDDRLPVKDGELMFVHSAEGREFWSALLEKAYAKMNG
CYEALSGGSTTEGFEDFTGGIAEVHDLNRPDLFHIIHMGQNRGSLMGCKLVKGHAYSVTGTAQYRGNMEKLIRIRNPWGQVEWTGAWSDAQWRQISDEDRERL-
EDGEFWMSFNDFLRHYSRLEICNLTPDALRWALSKFDGSWRRGSTAFWTNPQFVIRLDECSFVVGLITVGFAIYFFINLREVCSRFCLPPGEYLIVPSTFEANKDGDFCVRVFSEEISTHELQRILNKVVTTCRNMINMLDSGKL
GLLEFKVLWTKIENYLIYRQKDVDNSGTMSSSEMRTAVEEAGSLNNPLHQVLVARYSDLTIDFDNFVSCLVRLETMLL-
>Danio_rerio_ENSDARG00000034211
QDYEALKRECLESGRLFQDGMFEANVSALGSKVRGVEWLRPPTFISGGATRTDICQGGDCWLLAAIASLTLNQDVFARVVPAGQSFDGDYAGIFHFQFWQFGEWVDVVIDDRLPARKGELLFVHSAEGSEFWSALLEKAYAKLNG
CYEALSGGTTTEGFEDFTGGIAEVHELPKAGLFKTIQKALSWGSLLGCKLVKGHAYSVTGAEEYRGDLTKLIRIRNPWGQVEWTGPWSDSEWRQISDSDRERL-
EDGEFWMSFSDFMRHYSRVEICNLTPDALKWALSKFDGNWRNGSTAFWMNPQFLIKLEECSFVVGLITIGFAIYFFINLREVCSRFCLPPGEYLIVPSTFEPNKDGDFCVRVFSEEISAFELQKILNNVIATCRNMVNLLDTGKL
GLLEFKILWTKIELFVVYSKNDKDQSGTMSSMEMREAVEKAGSLNNALHQILVARYSELTIDFDNFVACLIRLECMML-
>Danio_rerio_ENSDARG00000090014
QDYEALKRECLESGRLFHDDMFEANVSALGSKVRGVEWLRPPTFISGGATRTDICQGGDCWLLAAIASLTLNQDVFARVVPAGQSFDGDYAGIFHFQFWQFGEWVDVVIDDRLPARNGELLFVHSAEGSEFWSALLEKAYAKLNG
CYEALSGGTTTEGFEDFTGGIAEVHELAKAGLFKTIQKALSWGSLLGCKLVKGHAYSVTGAEEYRGDLTKLIRIRNPWGQVEWTGPWSDSEWRQISDNDRERL-
EDGEFWMSFSDFMRHYSRVEICNLTPDALKWALSKFDGNWRNGSTAFWMNPQFLIKLEECSFVVGLITIGFAIYFFINLREVCSRFCLPPGEYLIVPSTFEPNKDGDFCVRVFSEEISAFELQKILNNVIATCRNMVNLLDTGKL
GLLEFKILWTKIELFVVYSKNDKDQSGTMSSMEMREAVEKAGSLNNALHQILVARYSELTIDFDNFVACLIRLECMML-
>Takifugu_rubripes_ENSTRUG00000012768
QNFAALRAECLSARKLFCDPAFPAAPEALGSKVRGVTWKRPPEFIMGGATRTDICQGGDCWLLAAIASLTLNEYVMARVVPSDQGFGDDYAGIFHFQFWQFGEWVDVVIDDRLPVKDNELMFVHSAEGREFWSALLEKAYAKVNG
CYEALSGGSTTEGFEDFTGGIAENYDLRQPPLFQIVKKALEAGALLGCKLVKGHAYSLTGAVEYRGRQEKLVRMRNPWGQVEWTGAWSDSEWNYMQ-GDCPHA-
EDGEFWMSFSDFLRHYSRIEVCTLTPDTIHWSVSKFDGTWRKGSTAFWMNPQFVINLQECSFVVGLITIGFAIYFFINLREVCSRFKLPPGEYLIVPSTFEPHQNGDFCIRVFSEEISAVELQTIMNKIVGTCRVMVNLMDNGKL
GLGEFATLWKKIQRYLIYKKNDIDNSGTMSTPEMRVAFKDAGTLNNTIYQLLVARYSDMTIDFDNFVGCLMRLEMMMV-
>Xiphophorus_maculatus_ENSXMAG00000008802
QNFEALRAQCLSSGKLFCDPTFPAEPESLGSKTRGVTWKRPPEFIVGGATRTDICQGGDCWLLAAIASLTMNEFVMARVVPTDQGFGDNYAGIFHFQFWQFGEWVDVVIDDRLPVKDGKLLFVHSAEGREFWSALLEKAYAKANG
CYEALSGGSTTEGFEDFTGGIAENYDLNRPPLFQIIKKALEAGALLGCKLVKGHAYSLTGAVEYRGHQEKLVRMRNPWGQVEWTGAWSDSEWSHVQ-GDCPHA-

```
EDGEFWMSFSDFQRHYSRIEVCTLTPDTIHWSVSKFDGTWRRGSTAFWTNPQFVIKLDECSFVVGLITIGFAIYFFINLREVSSRFKLPPGEYLVVPSTFDPHQNGDFCIRVFSE------------------------------
---------------------------------------------------------D---------------------
>Oreochromis_niloticus_ENSONIG00000007266
QNFNALRAQCRSAGKLFCDPTFPAAPESLGSKVRGVSWKRPPEFIVGGATRTDICQGGDCWLLAAIASLTLNEYVMARVVPTDQDFGDSYAGIFHFQFWQFGEWVDVVIDDRLPVKDGELMFVHSAEGREFWSALLEKAYAKVNG
CYEALSGGSTTEGFEDFTGGIAENYDLKKPPLFQIIKKALEAGSLLGCKLVKGHAYSLTGAVEYRGRQEKLVRMRNPWGQVEWTGPWSDSEWNYVQ-GECPHA-
EDGEFWMSFSDFQRHYSRIEVCTLTPDAIHWSVSKFDGGWRRGSTAFWTNPQFVIKLAECSFVVGLITIGFAIYFFINLREVSSRFKLPPGEYLIVPSTFEPHLNGDFCIRVFSEEISAVELRTIMNKIVSTCRVMVNLMDNGKL
GLGEFATLWKKVQRYLFYKKNDSDNSGTMSTPEMRVALKDAGTLNNNIYQLLVARYSDMTIDFDNFVGCLMRLEMMMI-
>Gasterosteus_aculeatus_ENSGACG00000019944
QDFASLRAQCRSAGKLFCDVTFPAEPEALGSKVRGVTWKRPPEFILGGATRTDICQGGDCWLLAAIASLTLNEYVMARVVPTDQGFGDNYAGIFHFQFWQFGEWVDVVIDDRLPVKDGELLFVHSAEGREFWSALLEKAYAKVNG
CYEALSGGSTTEGFEDFTGGIAENFDLQRPPLFYIIKKALEAGALLGCKLVKGHAYSLTGAVEYRGRQEKLVRVRNPWGQVEWTGAWSDSEWNYVE-GDCPHA-
EDGEFWMSFSEFSRNYNRVEVCTLTPDAIHWSVSKFDGTWRRGSTAFWTNPQFVVRLEECSFVVGLITVGFAIYFFINLREVSTRFKLPPGEYLVVPSTFEPHLNGDFCIRVFSEEISAGELRTILNKIVATSRIMVNLMDNGKL
GLGEFATLWKKVQRYLIYKKNDADNSGTMSTPEMRVAFKDAGTLNNNIYQLLVARYSDMTIDFDNFVGCLMRLEMMIL-
>Danio_rerio_ENSDARG00000091699
QSFETLRSECLSRGELFCDPAFPAAPEALGSKTRGVEWKRPPEFIVGGATRTDICQGGDCWLLAAIASLTLNEDVLARVVPSDQGFGQDYAGIFHFQFWQFGEWVDVVVDDRLPTRDGELLFVHSVTGSEFWSALLEKAYAKVNG
CYEALSGGSTTEGFEDFTGGIAEMYELKSAPLFQIIKKALDSGALLGCKLVKGHAYSLTGATEYRSRKEKLVRVRNPWGQVEWTGAWSDSEWNAVDPSERENV-
EDGEFWMSFSDFVRQYSRLEICTLTPDTLHWSVCKFDGTWRKGSTAFWMNPQFKIKLEECSVVIGLITIGYAIYYFINLREVSTRFKLPPGEYLIVPSTFDPHKNGDFCVRVFSEEISASELRTIFNKIVATCRVMVNLMDNGKL
GLTEFATLWKKIQKYLIYKKNDMDGSGCMSTPEMRMALKEAGSLNDCIHQSLAARYGDMTIDFDNFVSCVMRLEMMMI-
>Oryzias_latipes_ENSORLG00000020892
QNFEALRAQSLSSGQLFCDPAFPAAPESLGSKTRGVTWKRPPEFILGGATRTDICQGGDCWLLAAIASLTLNEFVMARVVPMDQGFGKDYAGIFHFQLWILGVLKLLVV-FKLDVKKGPEQLVKLSNGPEL-----------
VNGCYEALSGGSTTEGFEDFTGGIAENFDLREPPLFQILKKALEAGALLGCKLVKGHAYSLTGATEYRGRQEKLVRMRNPWGQVEWTGAWSDSEWNDVQ-GDCPHA-
EDGEFWMSFNEFLRNYSRVEVCTLTPDTIHWSVSKFDGTWRRGSTAFWTNPQFVIKLDECSFVVGLITIGFAIYFFINLREVSTRFKLPPGEYLIVPSTFDPHLNADFCIRVFSEEISAVELKTIMNKIVSTCRIMVNLMDNGKL
GLGEFATLWKKVQNYLIYKKSDLDNTGTMSTPEMRLAFKDAGTLNNTIYQLLVARYSDMTIDFDNFVACLMKLEMMMML
>Danio_rerio_ENSDARG00000055592
QDFEALRSECRARGTLFSDPTFPAAPESLGSKTRGLVWKRPPRFIVGGATKTDICQGGDCWLLAAIASLTQNEDVLARVVPNGQEFDGTYAGIFHFQFWQFGEWVDVVIDDRLPVKDGELLFVHSAEKNEFWSALLEKAYAKVNG
CYEALSGGSTSEGFEDFTGGIAESYEIRKAPLFQIIQKALEAGALLGCKLVKGHAYSLTGATEYRGRKEKLVRMRNPWGQVEWTGAWSDSEWNSVAASERPDA-
EDGEFWMAFSEFLTNYSRIEICTLTPDAIHWAVTNHDGTWRRGSTAFWMNPQFVVKLDECSFVVGLITIGFAIYFFINLREVCTRFHLPPGEYLIVPSTFEANKDGDFCLRVFSEEISPLELMTIFNKVIATCRVMVNLMDNGKL
GLGEFATLWKKVQRYLIYKHNDLDSSGTISTTELRMALKEAGCLNNTLFQLMVVRYAEMTLLFDNFVSCLMRLEMMMI-
>Lepisosteus_oculatus_GENSCAN00000007636
QDFEELRRQCLTAGKLFSDSTFPAAPESLGSKTRGVQWKRPPEFIVGGATRTDICQGGDCWLLAAIASLTLNEDVLARVVPSGQGFGNDYAGIFHFQFWQFGEWVDVVIDDRLPTRDGELLFVHSAEGSEFWSALLEKAYAKVNG
SYEALSGGSTTEGFEDFTGGIAETYELKQAPLFQIIKKALEAGSLLGCKLVKGHAYSLTGAVEYRGRLEKLVRIRNPWGQVEWTGAWSDSEWNYVDASERQNV-
DDGEFWMSFTEFMKQYSRLEICNLTPDTLRWSVSKFDGAWRRGSTAFWMNPQFVIKLEECSFVVGLITIGFAIYYFVNLREVSNHFKLPPGEYLIVPSTFEPHKDGDFCLRVFSEEISATELRTIFNNIVS--RQQVDLNPNG--
----------------VRQPVEED-------PEV------PGTLTNNIHQVLVARYAEMTIDFDNFVACLIRLETM---
>Scyliorhinus_canicula_TranscriptomeContig67723
QDYEALRQQCLETGSLFCDESFPACPSALGSKTRDVEWRRPPKFIVGEASRTDICQGGDCWLLAAIASLTLNNEILSRVVPRDQNFDSDYAGIFHFQFWQYGEWVDVVIDDRLPTKDDQLMFVHSASGNEFWSALLEKAYSKLNG
SYEALSGGSTTEGFEDFTGGISEWYELEKAPLFKIIRKALRSGSLLGCKLVKGHAYSVTGAEIYQGDEEKLIRIRNPWGQVEWTGSWSDAEWDCIPDEERERL-
DDGEFWMSFTDFLQNFSRVEICNLTPDSLKWSMVMYNGSWRTGSTAFWTNPQFKIQLDECSFLAGVITIGFAIYFFINLREVMSRLALEPGQYFIIPSTFEPNQSCDFLIRIFSEEISAFELQRILNRVVATCRNVISLLDSGKI
GLVEFKIFWIKLQKILIFKKVDADDSGTMSSHEMRMALEEAGTLNNTLFQILVARYADLLIDFDNFVGSLIRLETMQN-
>Leucoraja_erinacea_TranscriptomeContig62832
QDYEAIRQHCLDTGNLFCDESFPACPTSLGSKTRDVEWIRPPEFIIGGASRTDICQGGDCWLLAAIASLTLNNEVLNRVVPCEQDFGSDYAGIFHFRFWQYGEWVDVVIDDRLPTKDNELMFVHSASGNEFWSALLEKAYSKLNG
SYEALSGGSTTEGFEDFTGGISEWYELNEAPLFKIIRKALKAGSLLGCKLVKGHAYSLTGAETYQGNAEQLVRVRNPWGQVEWTGSWSDPEWECVPEEEKDRL-
ENGEFWMSFSEFLSNFSRVEICNLTPDTLKWNMAVYNGSWRKGSSAFWQNPQFKIDLRECSFLVGVITIGFAVYFFINLREVMSRLALEPGQYFVIPSTFEPNKDGDFAIRLFTEEISCFELQRILNRIVGTCRNVISLMDSGKI
GLVEFKLFWNKLQKMLIFKRVDADESGTMGSCEIQVALEEAGTLNTALIQNIVARYANLLIDFDNFVGCLIRLETMLG-
>Callorhinchus_milii_TranscriptomeContig60065
-------------------------------------------------------------------------------------------------------------------
MNDSLMFVHSASGNEFWSALLEKAYSKLNGSYESLSGGTTTEGFEDFTGGIAECYDLDSAPIFKIIQKAISRGSLLGCKLVKGHAYSITGAESYNDEQEKLIRIRNPWGQVEWTGPWSDDEWCCVPDEEKEAL-
EDGEFWMSFSDFMFHYSRVEICNLTPDVLKWNLKLFEGTWRNGSTAFWVNPQFKIELRDCSFLIGLITIGFAIYFFTNLREVSNRLSLEPGDYLIVPSTFEPNQNGNFAIRLFTEEISAFELQRILNKILSTCRNVVSLLDSGKI
GLVEFRIFWSKIQKYVVFKQMDADESGTMSAYELRLSLQEIGNLNNHLVQILIARYADLEIDFDNFVGCLVRLETMFN-
>Callorhinchus_milii_TranscriptomeContig17290
```

QSYEELRDRCLQTGVLFQDHSFPAAPDSLGSKTRGVEWQRPPRFIIEGATKTDICQGGDCWLLAAIASLTLNKEVLDRVVPDGQSFDSGYAGIFHFQFWQYGEWVDVVIDDQLPTKDGELMFVHSDEQNEFWSALLEKAYAKLNG
SYEALSGGSTTEGFEDFTGGVSEWYELNKAPLFHIIEKALKRGSLLGCKLVKGHAYSITGAQEFRGSQVQLIRIRNPWGQVEWTGAWSDSHWNSVSPQDAADL-
EDGEFWMSFSDFKRHYSRVEICNLTPDTLKWNCALFSESWRRGSTAFWMNPQFKMVLEECSVLVALITVGFAIYYFINLREVSARFCLPPGEYLVVPSTFEPNQNGDFALRVYTEEISVFELQKILNKIMSSCRNMVHLVDNGKM
GLVEFQKLWNKVQKFLIYKNNDLDQSGTMNSSEMRVAVEEAGHLNNKLCQIIVSRYYDLTLDFDNFVSCLVRLELVLC-
>Leucoraja_erinacea_TranscriptomeContig21198
------------------------------------------------------------------------------------------------------------------------------------------------
----------------FTGGVAEWFELKTPPLFSIIRRALERGSLMGCKLVKGHAYSMTGAEEFRGGPAQLVRIRNPWGEVEWTGAWSDAQWSGIEAEDRDRL-
DDGEFWMSFNDFLRHYSRLEICNLTPDTLRWNHSLYSESWRRGSTAFWTNPQFKVVLEECSMVLALITIGFAIYYFINLREVCGRYSLPPGQYLVVPSTFEPNQDADFALRVFTQEITVFGLQRILNKVVSTCRNMVNLLDNGQL
GLLEFKKLWDKIQKFLVYKRNDLDGSGTMSSSEMRVAVEESGQLNNQLTQILVARYSDLVLDFDNFVSCLIRLEAVLG-
>Chiloscyllium_punctatum_contig44567
QEYAVLRDHCLQTGSLFQDHSFPANASCLGSKTRDVSWLRPPQFLIEGATRTDICQGGDCWLLAAIASLTLNEEILSRVVPPGQSFGPGYAGIFHFQFWQFGEWVDVVVDDRLPTKDGKLMFVHSAERNEFWSALLEKAYAKLNG
SYEALSGGSTTEGFEDFTGGVSEWFELKSAPLFHIIEKALQRGSLMGCKLVKGHAYSLTGAQQFRGTQVQLVRIRNPWGEVEWTGAWSDPHWNGISAEDRNRL-
EDGEFWMAFPEFLRHYSRLEICNLTPDALKWNSTLFSESWRRGSTAFWMNPQFKITLEGCHFLVALITIGYAIYYFINLREVSTRFQLPPGEYLVVPSTFEPHQNGDFVVRVFSEEISAFGLQRILNKVVSTCRNMVNLLDNGKL
GLVEFKKLWDKIQKFLIYKKNDLDQSGTMSSSEMRVAVEEAGHLNNQLTQIIVARYSELTLDFDNFVSCLVRLEAVMG-
>Latimeria_chalumnae_ENSLACG00000001902
---------------------------------------------------------
GDCWLLAAIASLTLNKEVLERVVPGEQSFEDNYAGIFHFQFWQYGEWVDVVIDDRLPTKDGKLLFVHSAEGNEFWSALLEKAYSKLNTSYEALSGGSTTEGFEDFTGGIAERYELAKPPLFKILKKALQRGSLLGCKLVKGHAYS
LTGADEFRGQQVKLIRVRNPWGQVEWTGAWSDDHPSPLPRHLKLQCTNQTHFWMSYDDFRCHFSRVEVCNLTPDTLKWSLTKYYGTWRRGSSAFWMNPQFVIKLNECTFLIGLITIGFAIYFFINLREISSRFKLPSGEYLIVPS
TFEPNKEGDFCIRFFSEEISAFELQRILNKVVSTCKGMIDLMENDKLGLVEFKKLWGKIQGYLIYKKFDIDGSGTMTSYEMRNALDDAGTLNNCLQELLVTRYSDHIIDFDNFVRCLFRLELM---
>Oryzias_latipes_ENSORLG00000011856
QDFETLRQECLSSGRLFEDGCFPAQSKSLGSKTKGIVWKRPPKFIDDGATRTDICQGGDCWLLAAIASLTLDQQILAQVVPPGQSFSEDYAGIFHFQFWQYGEWVDVVIDDRLPTRDGKLLFVHSEEGSEFWSALLEKAYAKVNG
CYEALSGGNTIEGFEDFTGGIGEIFSLNKAPLFKIIQKALCLGSLIGCKLVKGHAYSVTAAEEVRGQQVQLVRIRNPWGQVEWTGAWSDREWNHISQEEKSKL-
EDGEFWMSYSDFIRNFSDLEICNLTPDLLCWNNYQFEGTWRVGSTAFPSNPQFLVHLEDCTFLVGLMTIGFAIYVFINRREVCGRFTLPPGDYAIIPSTFQPHKNGSFVLRVFSEEVSAIELQRILNKIFSTCRLIVSLMDSGKL
GPMEFRLLLTKIPKYLIFKSLDTDNSGTMSSHEMRNAAFKAGQINGSVLQAIVNRYADYAIDFDSFVSCLVKLEMLIY-
>Xiphophorus_maculatus_ENSXMAG00000006017
---
EALRQQCLSRGRLFEDGSFGAESKSLGSKTRGIVWKRPPKFIDDGATRTDICQGGDCWLLAAIASLTLDQRILGRVVPTEQSFTEDYAGIFHFQFWQYGEWVDVVIDDRLPTRDGKLLFVHSAEGSEFWSALLEKAYAKVNGCYE
ALSGGNTIEGFEDFTGGIAEIYTLSKAPLFQIMRKALSLGSLLGCKLVKGHAYSVTGAEEYRGREVQLVRVRNPWGEVEWTGPWSDYEWKYVSEADKSKL-
EDGEFWMSYSDFITHFSKLEICNLTPDTLHWNNYQFEGMWRVGSTAFCSNPQFVMRLEDCTFLVGLMTIGFAIYVFINTREVCERFRLPPGEYAIIPSTFHPHKNGKFVLRVFSEEVSAFELVEILNNIVSTARLIVGLLDNAKL
GLKEFHVLWNKLQKYLIFKSHDTDNSGTMSSLEMRAAATKAGQVNSAVLQAIVNRYADFAIDFDSFVSCLIKLEMLMY-
>Oreochromis_niloticus_ENSONIG00000009988
QDYEALRQQCLETGRLFQDDCFPPEPKSLGSKTKGIVWKRPPQFIDDGATRTDIRQGGDCWLLAAIASLTLEQDILARVVPPDQSFTEGYAGIFHFQFWQFGEWVDVVVDDRLPTRDGKLLFVHSAEGSEFWSALLEKAYAKVYG
SYEALTGGNTIEGFEDFTGGIAETYNLKEAPLFHVIQRALSLGSLMGCKLVKGHAYSVTGAEEFKGQQVQLVRIRNPWGEVEWTGPWSDSEWSHVTEDEKLKL-
EDGEFWMSYSDFIRNFSKLEICNLTPDTLRWNYCQFEGMWRVGSTAFPSNPQFLLRLEDCTFLVGLMTIGFAIYVFINTREVCGRFNLPPGEYAIIPSTFQPHKNGSFILRVFTEEVSAFELIKILNNAVSTGRLMVSLLDSGKL
GLMEFQLLWKKIQKYLIFKSHDTDKSGTMSSHEMRDAASKAGQINSAVLRAVVNRYADYAIHFDSFVGCLIKLEMLIY-
>Danio_rerio_ENSDARG00000055715
QDYQSLKQECLAKRALFCDPTFPAESDSLGSKTKGVQWKRPPEFIMDGANRTDICQGGDCWLLAAIASLTLDKEILERVVPSGQSFTEDYAGIFHFQFWQYGEWVDVVIDDRLPTRDGKLLFVHSAEGSEFWSALLEKAYAKVNG
SYEALTGGSTTEGFEDFTGGITENYELSKAPLFKLVQKALALGSLLGCKLVKGHAYSVTAAEEFRGSLVQLVRIRNPWGEVEWTGAWSDKEWDSVRPEEKAKL-
EDGEFWMAYSDFIQQFSKLEICNLTPDTLRWSYSQFEGNWRVGSTAFCSNPQFMIKLEECTFLVGLITIGFAIYVFINLREVSERFKLPPGEYIIIPSTFEPHRKGSFILRVFAEEVSVLELQQILNTVVSTCRHIISLLDSGKL
GLLEFHTLWMKIQKYLIFKHRDTDNSGTMSSLEMRDAVKEAGQLNNDVLEVLIARYANYAIDFDSFVSCLIRLELLLS-
>Oreochromis_niloticus_ENSONIG00000007284
QDFEQLRSECLQIGSLFCDPTFPADWNSLGSKTGGVEWKRPPCFLADGARRTDVCQGGDCWLLAAIASLTLDPQILNRVVPPGQSFTSQYAGIFHFQLWQYGEWVDVVVDDRLPTRDGKLLFVHSAEGVEFWSALLEKAYAKMSS
SYEALSGGSSIEGFEDFTGGIAESYDLKEAPLFHIVRKALKLGSLLDCKLVKGHAYSITAAEQHCGSLVELIRIRNPWGQVEWTGAWSDKEWDGVWAEEKRRL-
EDGEFWISYHDFLSHFSRLDICNLTPDTLRWNFAEFEGTWRVGSTAFCSNPQFFIRLDDCTVLIGLMTIGFAIYIFINLREVCDRFKLPPGEYAIVPSTFEPHCKGSFILRVFTEEISAFELQQILNKAVTTCRDIISLLDSSRL
GLLEFHSLWMKMQTYLIFKSHDADGSGTMSSHEMRAALAEAGQLNSTVIQEIVARYADYAIDFDRFIGCLIRLEILQSA
>Lepisosteus_oculatus_GENSCAN00000007654
QDFESLQRQFLAKGILFSDETFPAEPESLGSKTRGVVWKRPPQFIIGGANRTDICQGGDCWLLAAIASLTLDEEVLARVVPSGQSFDENYAGIFHFQFWQYGEWVDVVIDDRLPTRDGKLLFVHSAEGSEFWSALLEKAYAKVNG
SYEALSGGSTIEGFEDFTGGIAETYELSKAPLFQIIRKALGLGSLLGCKLVKGHAYSLTGAEEFRGSTVQLVRVRNPWGQVEWTGPWSDREWDYIDPKEKVRL-

EDGEFWMSFSDFVSHFSRLEICNLTPDTLKWNHYQFSGIWSVGSTAFCSNPQFAIKLEECTFLVGLMTVGFAIYIFINLREVCNRMKLPPGEYIIVPSTFEPHKKGSFVLRFFSEEISVFELQEILDRVVS--------------
--------------------------------------------RQLNTTVLQLLVNQYANYAIAFDSFVGCLTQLEMLAHG
>Xenopus_tropicalis_ENSXETG00000018599
-------------------------------------------------------------
GDCWLLAAIASLTLNEQILSRVVPKDQSFQENYAGIFHFQFWQYGEWVDVVIDDRLPTKDGKLLFVHSAEGSEFWSALMEKAYAKLNGSYEALSGGTTTEGFEDFTGGLAEWYELRKAPLFKIIQKALRTGSLLGCKLVKGHAYS
VTAAEEYRGNLEKLIRIRNPWGEVEWTGAWSDSEWNSVDPDVREKL-
DDGEFWMSFNDFLRNYSRLEICNLTPDTLKWSLTKMDGTWRRGSTAFWMNPQYVIKLEECTFIVGLITIGFAIYYYINLREVLNRFRLPPGEYVILPSTFEPHKVGDFCLRVFSEEISPHELFNILQKVISTCRTIVDLLDTGKL
GLKEFKILWTKILKYQIYSSVDRDHSGTINSYEMRGALEAAGKVNSTINELLVARFADHTIDFDNFVRCLLRLEIM---
>Lepisosteus_oculatus_GENSCAN00000007654
QDFEALRQQCLASGVLFEDETFPPVPSALGSKIRGVTWKRPPQFIVGGATRTDICQGGDCWLLAAIASLTLNEEVLARVVPSGQSFQESYAGIFHFQFWQFGEWVDVVIDDRLPTRDGELLFVHSAEGSEFWSALLEKAYAKLNG
SYESLSGGSTTEGFEDFTGGCAEVYELKKNPLFKIIQKAIERGSLLGCKLVKGHAYSVTGADEYRGDKEKLIRIRNPWGQVEWTGPWSD--------------------
MSFSDFLRHYSRVEICNLTPDALKWALSKFDESWRRGSTAFWMNPQFVIKLEECSFVVGLITIGFAIYFFINLREVCNRFTLPPGEYLIVPSTFEPQKNGDFCLRVFSEEISAFELRSILNKVVTSCSQFLRLKD---
LSLCSLQ----------IYRGKDADGSGTMSSPEMRVAVEEAGKLNNALHQILVARYSDLTIDFDNFVGCLIRLESLQAV
>Sus_scrofa_ENSSSCG00000001698
QSFEELRAICLRKGLLFEDPLFPAEPSSLGSNVQNISWQRPPQFIVNGVSPTDICQGGDCWLLAAIGSLTVYPKLFHRVVPKGQSFKKNYAGIFHFQIWQFGQWMDVVVDDRLPTKNGKLVFVHSAERTEFWSALLEKAYAKLNG
SYEALSGGNTMEGFEDFTGGVTQSFQLQTPPLLRMLRKGIERSSLMKKMLVKGHAYSVTGLQDYHGKTVTLIRVRNPWGRIEWNGAWSDKEWEEVSPDFQRQM-
EDGEFWMSYQDFLDNFTLLEICNLMPDTLCWHTTFYEGSWRRGSTAFWTNPQFKLSLPECTCLVALMTIGFVIYFFTNSREVSNHLRLPPGEYIIIPSTFEPHKDADFLLRVFSEEIGVYELQKLLNKVASVCRCMINLLDSGKL
GLQEFQILWRKIKKWTIFQECDQDHSGTLNSYEMRLAIERAGKLSNKVTQVLVARYANMILDFDSFISCFLRLKAMMWG
>Homo_sapiens_JF432283
QSFEELRAACLRKGELFEDPLFPAEPSSLGSNVQNISWQRPPLFIMDGISPTDICQGGDCWLLAAIGSLTTCPKLLYRVVPRGQSFKKNYAGIFHFQIWQFGQWVNVVVDDRLPTKNDKLVFVHSTERSEFWSALLEKAYAKLSG
SYEALSGGSTMEGLEDFTGGVAQSFQLQRPPLLRLLRKAVERSSLMGCMLVRGHAYSVTGLQDYRGKMETLIRVRNPWGRIEWNGAWSDREWEEVASDIQMQL-
EDGEFWMSYQDFLNNFTLLEICNLTPDTLYWHTTFYEGSWRRGSSAFWTNPQFKISLPECTCLVALMTIGFVLYFFTNSREVSSQLRLPPGEYIIIPSTFEPHRDADFLLRVFTEEIGVYELQRLLNRMAIACRCMINLMDSGKL
GLLEFKILWKKLKKWMIFRECDQDHSGTLNSYEMRLVIEKAGKLNNKVMQVLVARYADLIIDFDSFISCFLRLKTMMWG
>Mus_musculus_ENSMUSG00000058626
QNYEDLRAECLRKGELFEDPFFPAEPRSIGSHMQNIYWQRPPQFITNDFSPTDICQGGDCWLLAAIGSLTTCPKLLYRVVPRNQSFKKNYAGIFHFQLWQFGHWLNVVVDDRLPTRNNKLVFVHASHRQDFWSALLEKAYAKLIG
SYGALSGGSTLEGLEDFTGGVAQCIPLQKPPMLRLLKKALEKSSLMGCMLVRGHAYAVTGLEDYRDKLETLIRIQNPWGRVEWNGAWSDTEWEEVSPDVRVQL-
DDGDFWMSYEDFMSNFTLLEICNLTPDALRWHSTFYEGSWRRGSTAFWSNPQFKISLPECTCLVALMTIGFVIFFFINSREVNSHLRLPPGEYVIIPSTYEPHKDADFLLRVFTEEVDMYALHKLLNRMTAVCRRMINLLDSGKL
ELHEFQVLWKKIKKWTIFKECDEDRSGNLNSYEMRLAIEKAGKMNNRVTEVVVARYS-MIVDFDSFLNCFLRLKAMMWG
>Xenopus_tropicalis_ENSXETG00000012304
--------------------------------------------
PQFILGGANRTDICQGGDCWFLAAIACLTLNEKVLFRVIPPDQNFTDNYAGIFHFQFWRYGDWVDVIIDDYLPTYNNELVFTKSSQRNEFWSALLEKAYAKLHGSYEALKGGNTTEAMEDFTGGVTEFYELKEAPMYNIMKKAFE
RGSLIGCGLVKGHAYSVTGVEELKGKPIKLVRLRNPWGQVEWNGAWGDKEWTMVDKSEKTRLQEDGEFWVERDTYLY----LKHYRITSQTLCRTVTDELGQ--
RCYSRYWTNPQYRLKLLECSFVVALMTIGFAIYFYINMREVCQRFRLPPSEYVIIPSTYEPHQEGEFILRVFSEEISADELQSVLNNVVNSCRSMIALMDCGRLNLQEFYHLWQKIKQWQIFLRFDSDQSGTISSFEMRNAINEA
GHLNNQLYDIITMRYANMDLDFDSFICCFVRLEGMMYA
>Gallus_gallus_ENSGALG00000009050
KTYEELHKKCLEENILYEDPDFPPNETSLF-
VPIKFEWKRPPRFIIGGANRTDICQGGDCWFLAAIACLTLNKKLLCRVIPHDQSFIQNYAGIFHFQFWRYGDWVDVIIDDCLPTYNNQLVFTKSSQRNEFWSALLEKAYAKLHGSYEALKGGNTTEAMEDFTGGVTEFYEIKDAP
IYKIMKHAIARGSLMASGLVKGHAYSVTAVEEYKGEKMRLVRLRNPWGQVEWNGPWSDEEWNFIDEEEKTRLKEDGEFWISFEDFMRHFTKLEICNLTPDTLTWTVSVNEGRWVRGCSAFWTNPQYRLKLLECSFLVALMTIGFA
IYFYINMREISERFRLPPSEYVIIPSTYEPHQEGEFILRVFSEEINAEELRNVLNNVVKSCRSMIALMDSGKINFDEFRHLWDKIKSWQIFKHYDADHSGTINSYEMRNAVKDAGRLNNQLYDIITMRYADMNIDFDSFICCFVR
LDAMMYA
>Meleagris_gallopavo_ENSMGAG00000011468
KTYEELHKKCLEKNILYEDPDFPPNETSLF-
VPIKFEWKRPPRFIIGGANRTDICQGGDCWFLAAIACLTLNKKLLCRVIPHDQSFIQNYAGIFHFQFWRYGDWVDVIIDDCLPTYNNQLVFTKSSQRNEFWSALLEKAYAKLHGSYEALKGGNTTEAMEDFTGGVTEFYEIKDAP
IYKIMKHAIARGSLMASGLVKGHAYSVTAVEEFKGEKIRLIRLRNPWGQVEWNGPWSDEEWNFIKEEEKIRLKEDGEFWISFEDFMRHFTKLEICNLTPDTLTWTVSVNEGRWVRGCSAFWTNPQYRLKLLECSFLVALMTIGFA
IYFYINMREISERFRLPPSEYVIIPSTYEPHQEGEFILRVFSEEINAEELRNVLNNVVKSCRSMIALMDSGKINFDEFRHLWDKIKSWQIFKHYDADHSGTINSYEMRNAVKDAGRLNNQLYDIITMRYADMNIDFDSFICCFVR
LDAMMYA
>Taeniopygia_guttata_ENSTGUG00000011081
KTYEELHKKCLEKNILYEDPDFPPNESSLF-
SPVKFEWKRPPRFIIGGANRTDICQGGNCWFLAAIACLTLNKKLLCRVIPHDQSFIQNYAGIFHFQFWRYGNWVDVVIDDCLPTYNNQLVFTKSSQRNEFWSALLEKAYAKLHGSYEALKGGNTTEAMEDFTGGVTEFYEIKDAP

```
IYKIMKHAIDRGSLMASGLVKGHAYSVTAVEEFKGEKIRLVRLRNPWGQVEWNGAWSDEEWDSVNEAEKIRLKEDGEFWISFQDFMRHFTKLEICNLTPDTLTWTVSVNEGRWVRGCSAFWTNPQYRLKLLECSFLVALMTIGFA
IYFYINMREISERFRLPPSEYVIIPSTYDPHQEGEFILRVFSEEINAEELRNVLNNVVKSCRSMIALMDSGKINFDEFRHLWDKIKSWQIFKRYDTDHSGTINSYEMRNAVKDAGRLNNQLYDIITMRYADMNIDFDSFICCFVR
LDAMLYA
>Pelodiscus_sinensis_ENSPSIG00000011344
KTFEELHKKCLEKKILYEDPDFPPNESSLF-
LPVKFEWKRPPRFIIGGANRTDICQGGDCWFLAAIACLTLNEKLLCRVIPHDQTFIKNYAGIFHFQFWRYGDWVDVVVDDFLPTYNNQLVFTKSSQRNEFWSALLEKAYAKLHGSYEALKGGNTTEAMEDFTGGVTEFYEIKDAP
IYKIMKHASDRGSLMACGLVKGHAYSVTAVEEFKAGKIKLVRLRNPWGQVEWKGAWSDDEWTLIDKAEKIRLNEDGEFWMSFEDFMRYFTKLEICNITPDTLTWTVSVNEGRWVRGCSGFWTNPQYRLKLLECSFLVALMTIGFA
IYFYINIREVSERFRLPPSEYVIIPSTYEPHQEGEFILRVFSEEISADELRSVLNNVLKSCRSMIALMDSGKINFQEFQHLWNKLKSWQIFKHYDTDHSGTINSYEMRNAVKDAGSLNKQLYDIITMRYADMNIEFDSFICCFVR
LEGMMYA
>Sus_scrofa_ENSSSCG00000004728
KTFEQLHKKCLEKKVLYLDPEFPPDETSLF-
FPIQFVWKRPPRFIIGGANRTDICQGGDCWFLAAIACLTLNKRLLFRVIPHDQSFTENYAGIFHFQFWRYGDWVDVVIDDCLPTYNNQLVFTKSNHRNEFWSALLEKAYAKLHGSYEALKGGNTTEAMEDFTGGVTEFFEIKDAP
MYKIMKKAIERGSLMGCGLVKGHAYSVTGLEEFKGEKVKLVRLRNPWGQVEWNGSWSDKDWSFVDKDEKARLQEDGEFWMSYDDFIYHFTKLEICNLTADALTWTVSVNEGRWVRGCSAFWTNPQYRLKLLECSFLVALMTIGFA
IYFYINMREVSERFRLPPSEYVIVPSTYEPHQEGEFILRVFSEEICADELKNVLNRVVNSCRSMIALMDSGRLNLQEFHHLWKKIKSWQIFKHYDTDQSGTINSYEMRNAVNDAGHLNNQLYDIITMRYADMNIDFDSFICCFVR
LEGMMYA
>Homo_sapiens_AF127765
KTFEQLHKKCLEKKVLYVDPEFPPDETSLF-
FPIQFVWKRPPRFIIDGANRTDICQGGDCWFLAAIACLTLNQHLLFRVIPHDQSFIENYAGIFHFQFWRYGEWVDVVIDDCLPTYNNQLVFTKSNHRNEFWSALLEKAYAKLHGSYEALKGGNTTEAMEDFTGGVAEFFEIRDAP
MYKIMKKAIERGSLMGCGLVRGHAYSVTGLDEFKGEKVKLVRLRNPWGQVEWNGSWSDKDWSFVDKDEKARLQEDGEFWMSYEDFIYHFTKLEICNLTADALTWTVSVNEGRWVRGCSAFWTNPQYRLKLLECSFLVALMTIGFA
IYFYINMREVSQRFRLPPSEYVIVPSTYEPHQEGEFILRVFSEEICADELKKVLNTVVNSCRSMIALMDSGKLNLQEFHHLWNKIKAWQIFKHYDTDQSGTINSYEMRNAVNDAGHLNNQLYDIITMRYADMNIDFDSFICCFVR
LEGMMYA
>Mus_musculus_ENSMUSG00000079110
KTFEQLRRKCLEKKVLYLDPEFPPDETSLF-
FPIQFVWKRPPRFIIGGANRTDICQGGDCWFLAAIACLTLNERLLFRVIPHDQSFTENYAGIFHFQFWRYGDWVDVVIDDCLPTYNNQLVFTKSNHRNEFWSALLEKAYAKLHGSYEALKGGNTTEAMEDFTGGVTEFFEIKDAP
MYKIMRKAIERGSLMGCGLVKGHAYSVTGLEEFKGEKVKLVRLRNPWGQVEWNGSWSDKDWSFVDKDEKARLQEDGEFWMSYDDFVYHFTKLEICNLTADALTWTVSVNEGRWVRGCSAFWTNPQYRLKLLECSFLVALMTIGFA
IYFYINMREVSQRFRLPPSEYVIVPSTYEPHQEGEFILRVFSEEICADELKNVLNTVVNSCRSMIALMDSGRLNLQEFHHLWKKIKAWQIFKHYDTDHSGTINSYEMRNAVNDAGHLNSQLYDIITMRYADMNIDFDSFICCFVR
LEGMMYA
>Sarcophilus_harrisii_ENSSHAG00000007516
KTFEQLHKKCLEKKVLYVDPDFPPNETSLF-
FPIQFVWKRPPRFIIGGANRTDICQGGDCWFLAAIACLTLNERLLFRVIPHDQTFTENYAGIFHFQFWRYGDWVDVVIDDCLPTYNNQLVFTKSNHRNEFWSALLEKAYAKLHGSYEALKGGNTTEAMEDFTGGVTEFFEIKDAP
MYKIMKKAIERGSLMGCGLVKGHAYSVTGLEEFKGEKVKLVRLRNPWGQVEWNGSWSDKDWVLIEKEEKSRLQEDGEFWMSYEDFMYHFTKLEICNLTADALTWTVSVNEGRWVRGCSAFWTNPQYRLKLLECSFLVALMTIGFA
IYFYINMREVSERFRLPPSEYVIVPSTYDPHQEGEFILRVFSEEICADELKNVLNTVVNSCRSMIALMDSGRLNLQEFHHLWKKIKSWQIFKHYDTDHSGTINSYEMRNAVNDAGHLNSQLYDIITMRYADMNINFDSFICCFVR
LEGMMYA
>Monodelphis_domestica_ENSMODG00000017909
KTFEQLHKKCLEKKVLYLDPDFPPDETSLF-
FPIQFVWKRPPRFIIGGANRTDICQGGDCWFLAAIACLTLNERLLFRVIPHDQTFTENYAGIFHFQFWRYGDWVDVVIDDCLPTYNNQLVFTKSNHRNEFWSALLEKAYAKLHGSYEALKGGNTTEAMEDFTGGVTEFFEIRDAP
MYKIMKKAIERGSLMGCGHFINNCSSICFLSKFKGEKVKLVRLRNPWGQVEWNGSWSDKDWVLIDKEEKSRLQEDGEFWMSYEDFMYNFTKLEICNLTADALTWTVSVNEGRWVRGCSAFWTNPQYRLKLLECSFLVALMTIGFA
IYFYINMREVSERFRLPPSEYVIVPSTYDPHQEGEFILRVFSEEICADELKNVLNTVVNSCRSMIALMDSGRLNLQEFHHLWKKIKSWQIFKHYDTDHSGTINSYEMRNAVNDAGHLNSQLYDIITMRYADMNINFDSFICCFVR
LEGMMYA
>Anolis_carolinensis_ENSACAG00000015493
KTFHELHKKCLEKKILYEDPDFPANESSLF-
LPVKFEWKRPPRFIIGGANRTDICQGGDCWFLAAIACLTLNEKLLFRVIPHDQTFIKDYAGIFHFQFWRYGSWVEVIIDDRLPTYGKQLVFTKSSQQNEFWSALLEKAYAKLHGSYEALKGGNTTEAMEDFTGGVTEFYEIKDAP
IYKIMKHAIERGSLMASGLVLSHAYSVTGVEEFKREKLKLIRLRNPWGQVEWNGAWSDDEWNVIDGAEKTRLKEDGEFWISLQDFLRYFTKLEICNITPDALTWTVSVTEGRWVRGCTAFWTNPQYRLRLLECSFLVALMTIGFA
IYFYINMREVAERFRLPPNEYVIIPSTFEPHQEGEFLLRVFSEEISADELRNVLNNVLKSCRSMIALMDSGKINLEEFQHLWDKIKSWQIFKHYDTDHSGTINSYEMRNAVKDAGQLNNQLYDIITMRYADMNIEFDSFICCFVR
LEGMMYA
>Gadus_morhua_ENSGMOG00000013176
QDFTALKQECLQKKSLFEDDTFPATVESLGSKVKNIVWKRPPEFIVGGASRTDICQGGDCWLLAAIACLTLYEKLLYRVVPQEQSFSEGYAGVFHFQFWRYGDWVDVVIDDRIPTFNNQLVFTKSAERNEFWSALLEKAYAKLHG
SYEALKGGNTTEAMEDFTGGVTEFYEMKEAPLNKIMKKALERGSLMGCGLVKGHAYSVTAVQERKDSKVRLVRLRNPWGQVEWTGPWSDKEWTSLSKDEKEKLQEDGEFWMSFEDFKKNFTKIEICNLTPDALKWTVSVNEGRWV
```

RGCSAFWTNPQYRLRLLECSFVVSLMTIGFAIYFYINLREVTQRFRLSPGEYVIVPSTYEPHQEGEFLLRVFSEEITANELKNLLNKVVSTCRSMIALMDTGRLNLQEFRHLWNKVKQWQIFKHYSVEQSGSISSYEMRNAVNDA
GRLNNQLYDIITMRYANMNIDFDSFISCLVRLEAMMYA
>Gasterosteus_aculeatus_ENSGACG00000005212
QDFNALRQECLQRKSPFEDDSFPATVESLGSKVKNIVWKRPPQFIVGGASRTDICQGGDCWLLAAIACLTLNEKLLYRVVPQEQSFSEGYGGIFHFQFWRYGDWVDVVIDDRIPTFNNQLVFTKSAERNEFWSALLEKAYAKLHG
SYEALKGGNTTEAMEDFTGGVTEFYELKEAPLYKIMKKALLRGSLMGCGLVKGHAYSVTAVDEQKDSKVRLVRLRNPWGQVEWNGPWSDKEWANLSAAEKEKLQEDGEFWMSFEDFKKNYTKIEICNLTPDALKWTVSVNEGRWV
KGCSAFWTNPQYRLRLLECTFVVALMTIGFAIYFYINLREVTQRFRLSPGEYVIVPSTYEPHQEGEFILRVFSEEITANELKNVLNRVITSCRSMIALMDTGRLNLQEFRHLWNKIKQWQIFKHYNADQSGSINSYEMRNAVNDA
GRLNNQLYDIITMRYANMNIDFDSFISCLVRLEAMMYA
>Xiphophorus_maculatus_ENSXMAG00000009351
QDYNTLRQDYLQRKTLFEDEAFPATVESLGSKVKNIVWKRPPEFIVGGASRTDICQGGDCWLLAAIACLTLNEKLLYRVVPQEQSFSENYAGIFHFQFWRYGDWVDVVIDDRVPTFNNQLVFTKSAERNEFWSALLEKAYAKLHG
SYEALKGGNTTEAMEDFTGGVTEFYEMKDAPLYKIMKKALERGSLMGCGLVKGHAYSVTAVDEHKDNKVRLVRLRNPWGQVEWNGPWSDKEWANLSKADKDKLQEDGEFWMSFEDFKKNYTKIEICNMTPDALKWTVSVNEGRWV
RGCSAFWTNPQYRLRLLECTFVVSLMTIGFAIYFYINLREVTQRFRLSPGEYVIVPSTYEPHQEGEFILRVFSEEITANELRNVLNRVITSCRSMIALMDTGRLNLQEFIHLWNKIKQWQIFKHYNADQTGSINSYEMRNAVNDA
GRLNNQLYDIITMRYANMNIDFDSFISCLVRLEAMMYA
>Oryzias_latipes_ENSORLG00000018135
QDFSTLRQECLQRKGLFEDDSFPATVESLGSKVKNIVWKRPPQFIVGGASRTDICQGGDCWLLAAIACLTLNEKLLFRVVPQEQSFSESYAGIFHFQFWRYGDWVDVVVDDRIPTLNNQLVFTKSAERNEFWSALLEKAYAKLHG
SYEALKGGNTTEAMEDFTGGVTEFYEMKEAPLYKTMKKALERGSLMGCGLVKGHAYSVTAVDEQKESKVRLVRLRNPWGQVEWNGPWSDKEWATLSKAEKEKLQEDGEFWMSFEDFKKNYTKIEICNLTPDTLKWTVSVNEGRWL
RGCSAFWTNPQYRLRLLECTFVVSLMTIGFSIYFYINLREVTQRFRLSPGEYVIVPSTYEPHQEGEFILRVFSEEITANKLKNVLNRVITSCRSMIALMDTGRLNLQEFRHLWNKIKQWEIFKHYNADQSGIINSYEMRNAVNDA
GRLNNQLYHIITLRYANMNIDFDSFISCLVRLEAMMYA
>Danio_rerio_ENSDARG00000041864
QDYTLLKQEYLQKKTLFEDETFPATVDSLGSKVKNIVWKRPPQFIVGGASRTDICQGGDCWLLAAIACLTLNDKLLYRVIPQEQSFSEQYAGIFHFQFWRYGDWVDVVVDDRIPTFNNQLVFTKSAERNEFWSALLEKAYAKLHG
SYEALKGGNTAEGMEDFTGGVTEFYEMKEAPLYKIMQKALERGSLMGCGLVKGHAYSVTAVEEQKESRVRLVRLRNPWGQVEWNGPWSDKEWESLSKAEKEKLQEDGEFWMSFEDFKKNYTKIEICNLTPDALKWTVSVNEGRWV
RGCSAFWTNPQYRLRLLECTFVVALMTIGFSIYFYINLREVTQRFRLSPGEYVIIPSSYEPHQEGEFILRVFSEEVSANELKDVLNKVVSCCRSMIALMDTGRLNLQEFRQLWNKIKQWQIFKRYDFDHNDTISSYEMRNAINDA
GRLNNQLYDIITMRYANMNVDFESFISCLVRLEGMMYA
>Takifugu_rubripes_ENSTRUG00000006900
QDYSVLRQECVHRKSLFEDDTFPATVESLGSKVKNIVWKRPPQFIVGGASRTDICQGGDCWLLAAIACLTLNEKLLYRVVPSEQSFSEGYVGVFHFQFWRYGDWVDVVIDDRIPTFNNQLVFTKSAERNEFWSALLEKAYAKLHG
SYEALKGGNTTEAMEDFTGGVTEFYEMKEAPLYKIMKKALDRGSLMGCGLVKGHAYSVTAVEEHKDAKVRLVRLRNPWGQVEWNGPWSDKEWSTISKAEKEKLQEDGEFWMSFEDFKKNYTKIEICNLTPDALKWTVSVNEGRWV
RGCSAFWTNPQYRLRLLECTFVVALMTIGFAIYFYINLREVTQRFRLSPGEYVIVPSTYEPHQEGEFILRVFSEEITANELKNVLNRVIISCRSMIALMDTGRLNLQEFRHLWNKLKQWQTFKHYDADQSGFINSYEMRNAVNDA
GRLNNQLYDIITMRYANMNIDFDSFISCLVRLEAMMYA
>Scyliorhinus_canicula_TranscriptomeContig17411
QNFVTLKQECLQKKILFEDDTFPATVESLGSKVKNILWKRPPKFIIGGASRTDICQGGDCWFLAAIACLTLNEKLLYRVIPRDQYFTENYAGIFHFQFWRFGDWVDVTVDDRIPTFNNQLVFTKSAERNEFWSALLEKAYAKLNG
SYEALKGGNTTEAMEDFTGGVTEFFEMKEAPLYKILKKATDRGSLMGCGLVKGHAYSVTGVDEYKSQKVKLLRLRNPWGAVEWNGSWSDREWSEIEKIQKQRLQEDGEFWMSFDDFKRNFTKLEICNLTPDALKWTVSINEGRWV
KGCSAFWTNPQYRLKLCECTFVVALMTIGFAIYFYINLREVSQRFSLSPGEYVIVPSTYDPHQEGEFVLRVFSEQISASEMRNILNRVVKSCRSMVALMDSGKLNLMEFRHLWNKIKKWQIFMEYDKDASGLIDSYEMRTAVNDA
GQLNSQLYEIISMRYADMNLDFDSFICCIVRLEGMLYA
>Ginglymostoma_cirratum_contig59126
KDFQQLKDECLREGVLFEDEEFPANDSSLFVMPFKLEWKRPPKFIIGGASRTDICQGGDCWFLAAIACLTLNEKLLYRVIPRDQYFTENYAGIFHFQFWRFGDWVDVIVDDRIPTFNNQLVFTKSAERNEFWSALLEKAYAKLHG
SYEALKGGNTTEAMEDFTGGVTEFFEIKEAPLFKILKKATERGSLMGCGLVRGHAYSVTSVDEYKGQTVKLVRLRNPWGSVEWNGSWSDREWSEIEKTQKQRLQEDGEFWMSFDDFKRNFTKLEICNLTPDALRWTVSINEGRWV
KGCSAFWTNPQYRLKLCECTFVVALMTIGFAIY----------------------------------------------------------------------------------------------------------
--------------------------------------
>Latimeria_chalumnae_ENSLACG00000003659
QDFATLKREWLGKKVLYEDEVFPATVESLGAKVKSIVWKRPPQFIIGGANRTDICQGGDCWLLAAIACLTLNNKLLCRVVPPDQSFTENYIGIFHFQFWRYGDWVDVVIDDRIPTYNNQLVFTKSAQRNEFWSALFEKAYAKVHG
QESACLPELSIECMHTYSGESMGFFYGLAPPLGKMIARVLSSSGNSDSGLVKGHAYSITGVEELKGQKVKLVRLRNPWGQVEWNGPWSDKEWTTLEKSEKMRLHEDGEFWMSFDDFKKNFTKLEICNLTPDALKWTVSVNEGRWV
RGCSAFWTNPQYRLKLLECTFLVALMTIGFAIYFYINLREVSHRFRLSPGEYVIIPSTYEPHQEGEFILRVFSEEISSCELRSVLNRVLASCRSMIALMDSGRLNLQEFRLLWNKIKQWQIFKQYDMDKTGYINSYEMRNAVNDA
GRLNNQLYDIITMRYADMNIDFDSFILCFVRLEGMMYA
>Lepisosteus_oculatus_GENSCAN00000022004
QDFSLLKQESLRKKVLFEDDCFPATVESLGSKVKNIIWKRPPSMMDTAPVKAQVQKLGDCWLLAAIACLTLNEKLLYRVIPPDQSFTENYAGIFHFQFWRYGDWVDVVVDDRIPTFNNQLVFTKSAERNEFWSALLEKAYAKLHG
SYEALKGGNTTEAMEDFTGGVTEFFEMKEAPLFKIMSKALERGSLMGCGLVKGHAYSVTAVEE--------------------------
EWVTIPKSEKDKLQEDGEFWMSFEDFKKNFTKIEICNLTPDALKWTVSVNEGRWVRGCSAFWTNPQYRLRLMECTFVVALMTIGFAIYFYINLREVSQRFRLCPGEYVIVPSTYEPHQEGEFILRVFSEEITANELKNVLNRVVA
VCHQ--EKIPVKEVNANEV----NVVLCFQIFKHYDADHSGTINSYEMRNAVNDAGRLNNQLYDIITMRYANMNMDFDSFICCLVRLEGM---
>Gadus_morhua_ENSGMOG00000000192

KTFLELRDKYVHKKVLFEDPLFPADDSSLY-
SPMKFEWKRPPHFIIDGANRSDICQGGDCWLLAAIACLTLNEKLLYRVIPPDQSFTENYAGIFHFQFWRYGEWIDVIIDDRIPTFNDKLVFTKSFRKNEYWSALLEKAYAKLHGSYEALKGGNTLEAMEDFTGGVTEYFDLLEAP
LYSIMKKALERGSLMGCGLVQGHAYSVIGLAEGTDSQIRLVRLRNPWGWVLWKGRWCAKEWSTISTADKENLQQESEFWISFEDFKKCYSKLEMCNLTPDTLSWSVAVNEGRWVRGSSAYWTNPQYRMLLYECTVVVALMTIGFS
IYFYINLREITERFRLPPGEYVVIPTTFKPHNEGEFILRVFSEQICASELKVVMKRVLETCRSMIALMDTGKLNLQEFKHLWNKIKQWQIFRMYDKDKSCTISSFEMRNAVNDPGHLNKQLYDILAMRYADNNIDFDSFICCFVR
LEGMMYS
>Oryzias_latipes_ENSORLG00000012402
KTFLELRDKYVKKKVLFEDPLFPANDSSLF-
PSMKIEWKRPPQFIIDGANRTDICQGGDCWLLAAIACLTLSEKLLYRVIPPEQSFTENYAGIFHFQFWRYGEWIDIVVDDRIPTCNNLLVFTKSFRENEFWSALLEKAYAKLHGSYEALKGGNTLDAMEDFTGGVTELFELSEAP
LFIIMKKALERGSLMGCGLVRGHAYSIIALEESEDGKIRLIRLRNPWGWVLWKGPWSVKEWSTVSTADRDNLQEMSEFWMSFDDFKKNFTKLEMCNLTPDALSWTVSINEGRWVRGSSAFWTNPQYRLRLYECTVVVALMTIGFS
IYFYINLREVTERFRLPPGEYVILPTTFKAHEEGEFLLRVFSEEICASELMAIMKNVLSTCRSMIALMDSGKLNLQEFKHLWKKIKEWQIFKRYDKDGKCSISSFEMRNAVNDAGNLNKQLYDIIAMRYADLNIDFDSYICCFVR
LEGMMYS
>Xiphophorus_maculatus_ENSXMAG00000017636
KTFLELRDKYVTKHILFEDPLFPANDSSLF-
PEMKFEWKRPPQFIVDGANRTDICQGGDCWLLAAIACLTLNEKLLYRVIPPEQSFTENYAGIFHFQFWRYGEWIDVVVDDRIPTCDNQLVFTKSFRKNEFWSALLEKAYAKLHGSYEALKGGNTLEAMEDFTGGVTEFFELSEAP
LFTIMRKALERGSLMGSGLVRGHAYSIIGLEESKDSRIRLIRLRNPWGFVLWKGRWSADEWSTISTADRENLQETSEFWMSFDEFKKTFTKLEMCNLTPDGLTWTVSVNEGRWVRGSSAFWTNPQYRLQLYECTVVVALMTVGFS
IYFYINLREVSERFRLPPGEYAIIPTTFEPHEEGEFILRVFSEEICANELKTVMKNVLSTCRSMIALMDSGKLNLQEFKHLWKKIKAWQIFKQYNQNKSCSISSFEMRNAVNDAGHLNKQLYDIIAMRYADLNIDFDSYICCFVR
LEGMMYS
>Oreochromis_niloticus_ENSONIG00000006073
KTFLELRDKYVEKNVMFEDPLFPADDSSLF-
PSMKIEWKRPPQFIIDGANRTDICQGGDCWLLAAIACLTLNEKLLYRVIPPDQSFTENYAGIFHFQFWRYGEWIDVVVDDRIPTCNNQLVFTKSFRKNEFWSALLEKAYAKLHGSYEALKGGNTLEAMEDFTGGVTEYFELSDDP
LYKIMKNALERGSLMGCGLVRGHAYSIISLEESKDTRIRLIRLRNPWGFVLWKGPWSAKEWSTISAADRENLQEASEFWMSFADFKKNFTKLEMCNLTPDTLTWTVSVHEGRWVRGSSAFWTNPQYRLQLYECTVVVALMTIGFS
IYFYINLREVTERFCLPPGEYVIIPTTFKPHEEGEFILRVFSEQICANELKTILKNVLSTCRSMIALMDTGKLNLQEFKHLWKKIKDWQIFKRYDKDKNWSISSFEMRNAVNDAGQLNRQLYDIIAMRYADLNIDFDSYICCFVR
LEGMMYS
>Gasterosteus_aculeatus_ENSGACG00000009830
KTFLELRDKYVKKNVVFEDPLFPANDSSLF-
PAMKFEWKRPPQFIIDGAKRTDICQGGDCWLLAAIACLTVNEKLLYRVIPPDQSFTDNYAGIFHFQFWRYGEWIDVIVDDRIPTCNNHLVFTKSFRKNEFWSALLEKAYAKLHGSYEALKGGNTLEAMEDFTGGVTEFFDLPEAP
LYSIMRKALERGSLMGCGLVRGHAYSIIGLAEEKDTTIRLIRLRNPWGWVLWKGPWSATEWSTISIADKDNLQQESEFWMSFADFTRNFTKLEMCNLTPDALSWTVSVNEGRWVRGSSAFWTNPQYRLQLYECTVVVALMTIGFS
IYFYINLREVTERVHLPQGEYAIIPTTFEPHQDGEFILRVFSEQICANELRTIMRNVLATCRSMIALMDTGKLNLQEFKHLWKKIKEWQIFRRYDENKTGCVSSFEMRNAVNDAGHLNKQLYDIIAMRYADLNIDFDSYICCFVR
LEGMVYS
>Takifugu_rubripes_ENSTRUG00000018064
KTFLELRDKYVKKKVIFEDPLFPANDSSLF-
PPMKIEWKRPPQFIIDGANRTDICQGGDCWLLAAIACLTVNEKLLYRVIPPDQSFTQNYAGIFHFQFWRYGEWVDVLVDDRLPTCRGQLVFTKSFRKNEFWSALLEKAYAKLHGSYEALKGGNTLEAMEDFTGGVTEFFELSEPP
LYSIMRKALERGSLMGCGLVKGHAYSIIGLTEANKAKVRLIRLRNPWGIVLWKGPWCPKEWSTISIADQENLQESSEFWMSFDDFQRNFTKLEMCNLTPDALSWTVSVNQGRWVRGSSAFWTNPQYRMKLYECTVVVALMTIGFS
IYFYINLREVTERFHLPPGEYVIIPTTFEPHQEGEFILRVFSEQICANELKGIMKNVLDTCRSMIALMDTGKLNLQEFKHMWRKIKAWQIFRRYTKDKTCSISSFEMRNAVNDAGHLNNQLYDIIAMRYADLNINFDGYICCFVR
LEGMIDV
>Danio_rerio_ENSDARG00000043035
KDFQELRDKYVRKKVLFEDPLFPAQDSSLF-
FPLKLEWKRPPQFILGGANRTDICQGGDCWLLAAIACLTLNDVILKRVVPHDQSFTENYAGIFHFQFWRYGEWVDVVIDDRLPTYKNKLVFTRSGQNNEFWSALMEKAYAKLHGSYEALKGGNSLEAMEDFTGGVTEFYEITEAP
LYNIMRKALKRGSLMGCGLVRGHAYSVTGVEQGKDLMIRLVRVRDPWGVA---
PPPACNDWVELAKTEQDKQQEQGEFWMCFEEFQKNFTKLEICNLTPDTLKWNVTVHEGRWVKGCSAYWTNPQFRLVLLECTVVVALMNIGFAIYFYVNIREVTERFCLKPGEYVIIPSTFDPHKESEFLLRVFSEEINANELRTV
LNRVVASCRSMIALLDTGHLNLQEFKHLWNKIKQWKVFTRFDTDKSSTISSFEMRNALTEAGQLNNQLYDIICMRYANMELDFDSYISCLVRLEGMMYA
>Lamprey_ENSPMAG00000007435
KSFEELRTLCLSHDVLFEDPDFPAHNNSIY-
APMQFQWMRPPRSIVIEANHTHICEGGDCWVLAAIACMTMNEELLARVIPAGQSFEENYAGIFHFQFWRFGEWVDVVVDDRLPTFNNQLVFVQSAERNEFWSALLEKAYAKVHGSYEALKGGNTTEAMEDFTGGVTEYFEMKNPP
LYKIMVKATSRRSLMGCGLVRNHAYSITGLDEHRSKIVQLVRLRNPWGHVEWIGPWSDKEWNEITPSEKQRLIEDGEFWISFQDFCRNFTKLEICNLSPDALKWTVSVSEGRWVRGCSAFWTNPQYRLQLYDCSFIVALMTIGFA
IYFYINLREVTERFRLPPGEYVIVPSTFEAHQEGEFLLRVFAEEVGAADLHNILNKVITSCRTMVALMDSGKLGYNDFKYLWDKLKFWQIFRKYDSDKSGSMTSYEMRLAINEAGQLNNQLYQIITLRHADLNIGFDNFIACLIR
LEAMMYA
>Xenopus_tropicalis_ENSXETG00000023310

KTYEQLKNDCLRKGVLFEDVDFPANDSSLF-
PSVPFVWKRPPEFILGGASRTDVCQGGDCWLLAAIASLTLNDKILYRVVPPDQSFSTGYAGIFHFQFWQHNEWLDVVIDDRLPTFRNRLVFVHSADLNEFWSALLEKASCLLNGSYEALKGGSTLEAMEDFTGGVTETYEIRSAP
LFDILDKAIKKGSMLGCGLVKGHAYSITGADVFKGQKVKLIRIRNPWGQVEWNGAWSDSEWNIIGAAEKNRLADDGEFWMDFEDFKRHFDKLEICNLTPDSLKWEVTVHEGSWIRGSTAFWSNPQIKLSLTECTFIAALMTIGYT
IYFYVNLREISQRFKLPPGDYMFIPTTFEPHQEADFCLRIFSEEVSAEELQYVLNAVLRSCNNIISLMDNGKLGFDEFKIFWDKLKTWIIFMQYDLDKSGTMSSYELRLALKSAGHLNNIVLQLIVLRYADFQIEFDDFLNCLVR
LENSMNI
>Gallus_gallus_ENSGALG00000011136
KGYQELKQECLRSGCLFEDPDFPANNASLF-
PPIPFLWKRPPKFILEGATRTDICQGGDCWLLAAIASLTLNEKTLARVVPLDQNFGPGYAGIFHFQFWQHNEWLDVVIDDRLPTFKDRLVFVHSAEHNEFWSALLEKAYAKLNGSYEALKGGSTIEAMEDFTGGVGEMYEVKKAP
FYEILEKALKRSSMVGCGLIKGHAYSVTGIDEYQGQKVQLIRIRNPWGQVEWNGPWSDLEWRLVSPSEQKRLTDDGEFWMKFEDFKVHFDKVEICNLTPDSLKWEVTIHEGSWVRGSTAFWTNPQIKLHLTECTFIAALMSIGYS
IYFYINLREVSDRFKLPPGDYILIPTTFEPHQEADFCLRIFSEEVAAEELEYVLNAVLKSCRNIISLMDNGKLEFSEFKVFWEKLKKWIIFLRFDFDKSGSMSSYELRSALKAAGQLNNYLLQLIVLRYSDFQIDFDDFLNCLIR
LENAMNI
>Meleagris_gallopavo_ENSMGAG00000012108
KGYQELKQECLRSGCLFEDPDFPANNASLF-
PPIPFLWKRPPKFILGGATRTDICQGGDCWLLAAIASLTLNEKTLARVVPLEQNFGPGYAGIFHFQFWQHNEWLDVVIDDRLPTFKDRLVFVHSAEHNEFWSALLEKAYAKLNGSYEALKGGSTIEAMEDFTGGVGEMYEVKKAP
FYEILEKALKRSSMVGCGLIKGHAYSVTGIDEYQGQKVQLIRIRNPWGQVEWNGPWSDLEWRLVSPSEQKRLTDDGEFWMKFEDFTVHFDKVEICNLTPDALKWEVTIHEGSWVRGSTAFWTNPQIKLHLTECTFIAALMSIGYS
IYFYINLREVSDRFKLPPGDYILVPTTFEPHQEADFCLRIFSEEVAAEELEYVLNAVLKSCRNIISLMDNGKLEFSEFKVFWEKLKKWIIFLRFDFDKSGSMSSYELRSALKAAGQLNNYLLQLIVLRYSDFQIDFDDFLNCLIR
LENAMNI
>Taeniopygia_guttata_ENSTGUG00000009921
KGYEQLKQECLRRGVLFEDPDFPACNSSLF-
PPIPFVWKRPPKFILGGATRTDICQGGDCWLLAAIASLTLNEKTLARVVPLDQNFGPDYAGIFHFQFWQHNEWLDVVIDDRLPTFKGRLVFLHSAELNEFWSALLEKAYAKLNGSYESLKGGSTIEAMEDFTGGIGEMYDVKAAP
FYEILEKALKRCSMVGCGLVKGHAYSVTGIEEYRGRQVQLIRIRNPWGEVEWNGPWSDAEWRSVSPSEQRRLADDGEFWMKFEDFKVHFDKVEICNLTPDALKWEVTIHQGSWVRGATAFWTNPQIKLHLTECTFIAALMTIGYS
IYFYINLREVSHRFKLPPGDYILIPTTFEPHQEADFCLRIFSEEISAEELEYVLNAVLKSCRNIISLMDNGKLEFSEFKVFWEKLKKWIIFLQFDFDKSGCMSSYELRGALKAAGQLNNYLLQLIVLRYSDFQIEFDDFLNCLIR
LENAMNI
>Pelodiscus_sinensis_ENSPSIG00000016067
QTYEELKRQCQQRGTLFEDCDFPANCSSLF-
PSIPFVWKRPPEFVVGGATRTDICQGGDCWLLAAIASLTLNENTLARVVPQDQNFGPGYAGIFHFQFWQHNEWLDIVIDDRLPTFKDRLVFLHSAEHNEFWSALLEKAYAKLNGSYEALKGGSTLEAMEDFTGGVGEMYDVKQAP
FYEILGKALKRGSMVGCGLIKGHAYSVTGIDEYQGRKVQLIRPRNPWGQVEWNGRWSDPEWKAVSPSEQKRLTDDGEFWMTFEDFKTHFDKVEICNLTPDALKWEVTVHQGSWVRGSTAFWTNPQIKLRLTECTFIAALMTIGYA
IYFYINLREVSDQFRLPPGEYVLVPTTFEPHQEADFCLRIFSEEITAEELEYVLNAVLKTCRNIISLMDNGKLEFNEFKIFWDKLKKWLIYLHFDSDKTGTMSSYELRSALKAAGQLNNYLLQLIVLRYSNYEIDFDDFLNCLIR
LENAMNI
>Anolis_carolinensis_ENSACAG00000001768
QTYKELKLECLQKGILFEDCDFPPNDASLF-
PPVPFVWKRPPQFIIGGATRTDICQGGDCWLLAAIASLTLNEKTLARVVPQDQDFGPGYAGIFHFQFWQHNEWLDVVIDDRLPTFKDRLVFLHSADHNEFWSALLEKAYAKLNGSYEALKGGSTIEAMEDFTGGVAEMYEVKKAP
FYEILEKALNRGSMVGCGLIKGHAYSVTGINEYQGRKVKLIRIRNPWGQVEWNGPWSDPEWNSISQSEKKRLTDDGEFWMKFEDFQTHFDKVEICNLTPDALKWAVTVHQGSWVRGATAFWTNPQFKIQLTECTFLAALMTIGYA
IYFYINLREVSDRFTLPPGNYIIVPTTFEPHQEADFCLRIFSEEISPEELEYVLNAVLKSCKNIISLMDNGKLDFSEFKIFWDKLKKWIIYLHFDADHSGTMSAYELRLALKAAGQLNNYLLQLIVLRYADYQIEFDDFLNCLIR
LENAMNI
>Sus_scrofa_ENSSSCG00000010182
QSFEELRQACLQKGVLFEDTDFPADSSSLF-
PQIPFVWKRPPEFILGGATRTDICQGGDCWLLAAIASLTLNEKALARVVPHNQSFGPGYAGIFHFQFWQHSEWLDVVIDDRLPTFRDRLVFLHSADHNEFWSALLEKAYAKLNGSYEALKGGSAIEAMEDFTGGVAETFTTKEAP
FYEILEKALTRGSLVGCGLIKGHAYTVTGVDQIRGQKVELIRVRNPWGQVEWTGSWSDSEWRSVGPAEQKRLTDDGEFWMAFRDFRAHFDKVEVCNLTPDALKWEVTVHQGSWVRGSTAFWTNPQIKLSLTECTFLVALMTIGYA
IYFFINLREVSDRFRLPPGDYILIPSTFEPHQEADFCLRIFSEEVTAEELKYVLNAVLQSCKNIISLMDNGKLEFSEFKVFWDKLKKWMLFLQFDADKSGTMSSYELRSALKAAGQLSSPLLQLIVLRYADLQLGFDDFLNCLVR
LENAMNI
>Homo_sapiens_NM_006615
QSFEQMRQECLQRGTLFEDADFPASNSSLF-
PQIPFVWKRPPEFILGGATRTDICQGGDCWLLAAIASLTLNQKALARVIPQDQSFGPGYAGIFHFQFWQHSEWLDVVIDDRLPTFRDRLVFLHSADHNEFWSALLEKAYAKLNGSYEALKGGSAIEAMEDFTGGVAETFQTKEAP
FYEILEKALKRGSLLGCGLIKGHAYSVTGIDQFRGQRIELIRIRNPWGQVEWNGSWSDPEWRSVGPAEQKRLTDDGEFWMAFKDFKAHFDKVEICNLTPDALKWEVTVHQGSWVRGSTAFWTNPQIKLSLTECSFLVALMTIGYA
IYFFINLREVSDRFKLPPGEYILIPSTFEPHQEADFCLRIFSEEVTAEELEYVLNAVLQSCKNIISLMDNGKLEFDEFKVFWDKLKQWILFLRFDADKSGTMSTYELRTALKAAGQLSSHLLQLIVLRYADLQLDFDDFLNCLVR
LENAMNI
>Mus_musculus_ENSMUSG00000031981

```
QSFEQLRQGCLQSGTLFEDADFPASNVSLF-
PQVPFVWKRPPEFILGGATRTDICQGGDCWLLAAIASLTLNQKALTRVVPQDQGFGSGYAGIFHFQFWQHSEWLDVVIDDRLPTFKDRLVFLHSADHNEFWSALLEKAYAKLNGSYEALKGGSAIEAMEDFTGGVAENFQIREAP
FFEILEKALKRGSLLGCGLIKGHAYTVTGLDQFHGQRIKLIRVRNPWGQVEWNGPWSDPEWRSVDLEEQKRLTDDGEFWMAFKDFKIHFDKVEICNLTPDALRWEVTIHQGSWVRGSTAFWTNPQIKLSLTECTFLAALMTIGYA
IYFFINLREVSERFQLPPGDYILIPSTFEPHQEADFCLRIFSEEVSAEELEYVLNAVLQSCRNIISLMDNGKLEFEEFRVFWDKLKHWMLFLQFDVDKSGTMSSYELRTALKAAGQLGGHLLQLIVLRYADLQLDFDDYLNCLVR
LENAMNI
>Sarcophilus_harrisii_ENSSHAG00000017756
QTYEQLRQDCLQRGILFEDGDFPANNSSLF-
PSIPFVWKRPPEFILGGATRTDICQGGDCWLLAAIASLTLNEKTLARVVPQDQNFGSGYAGIFHFQIWQHSEWLDVVIDDRLPTFRDRLVFLHSADHNEFWSALLEKAYAKLNGSYEALKGGSTIEAMEDFTGGVAETFEVKKAP
FYEILEKALKRSSMVGCGLIKGHAYTLTGIDQYRGQKVELIRVRNPWGQVEWNGPWSDGEWSSVESSEQKRLSDDGEFWMTFKDFKIHFDKVEICNLTPDALKWEVTIHQGSWVRGSTAFWTNPQIKLSLTECTFIVALMTIGYA
IYFYINLREVSERFKLPPGEYILIPTTFEPHQEANFCLRIFSEEVTAEELEYVLNTVLQSCKNIISLMDNGKLEFNEFKVFWDKLKTWIIFLQFDADRSGTMSSYELRLALKAAGQLNNYLLQLIVLRYADYQLDFDDFLNCMIR
LENAMNI
>Monodelphis_domestica_ENSMODG00000009241
KTYEELRQECLQRGILFEDRDFPANDSSLF-
PSIPFVWKRPPEFILGGATRTDICQGGDCWLLAAIASLTLNEKTLARVVPPDQNFGPGYAGIFHFQIWQHGEWLDVVIDDRLPTFRDRLVFLHSADHNEFWSALLEKAYAKLSGSYEALKGGSTIEAMEDFTGGVAETFIIKAAP
LYEILEKAVKRGSLVGCGLIKGHAYTVTGINQYKGQKVELIRVRNPWGQVEWNGPWSDPEWNFVNSSEQKRLSDDGEFWMTFKDFKVHFDKVEICNLTPDALKWEVTIYQGSWVRGSTAFWTNPQIKLSLTECTFIVALMTIGYA
IYFYINLREVSNRFKLPPGEYILIPSTFEPHEEANFCLRIFSEEVTAEELRHVLNTMLQSCKNIISLMDSGKMEFNEFKMFWIRLKQWIIFLQFDADKSGTMSSYELRLALKAAGQLGNYLLQLIVLRYADYQLDFDDFLNCMIR
LENAMNI
>Ornithorhynchus_anatinus_ENSOANG00000013738
RTFEELRDRCLRRGVLFEDPDFPADDSSLF-
PPIPFAWKRPPEFVSDGATRTDICQGGDCWLLAAIASLTLNPRVLARVVPPQQSFGPDYAGIFHFQLWQHGQWLDVVIDDRLPTFKDRLVFLHSADHNEFWSALLEKAYVKLNGSYEALKGGSTIEAMEDFTGGVAETLDVKAIS
LSEILRKALDRGSLVACGLVKGHAYSVTGTDQFRGRRVELVRVRNPWGQVEWNGPWSDSEWRSVDPSEKKRLTDDGEFWMPFADFEAHFDKVEICNLTPDALQWEVAVHHGSWVKGATAFWTNPQIKLSLSKCTLVVALMTIGYA
IYFYVNLREVSGRFKLPPGEYVLVPSTFEPHQEADFCLRIFSEQISAEELQEILNAVLRSCRNIISLMDSGKLELDEFKAFWARLKAWTIFFQFDGDKSGTMSSYELRGALKAAGQLNNNLLQLLVLRYADLQVDFDDFLNCLVR
LENTMNI
>Gadus_morhua_ENSGMOG00000005292
RSFKEIRQDCLQRQVLFEDPDFPADDSALF-
PPLAFEWKRPPRFVVGDANRTDICQGGDCWLLAATASLTLQKKNLARVVPRDQEFDNSYAGVFHFQFWQHNKWLDVVVDDRLPTVRNKLVMLHSASNNEFWSALLEKAYAKLHGSYESLKGGSTMEAMEDFTGGVGEMYETKSAP
LFTIMKKALDRSSMMGCGLVKGHAYSITGLEECRGRKVQLIRIRNPWGTVEWNGPWSDKEWSQVDKADKDRIHDDGEFWMEFEDFKSNYDKVEICNLTPDSLHWEVSWFEGNWIRGSTAFWTNPQFKLTLEDCSVVIALMTIGFA
VY-----------------------------------------------------------------------------------------------------------------------------------------------
-------
>Takifugu_rubripes_ENSTRUG00000002630
KSFEQLRQECLQKGILFEDPDFPATDSSLY-
VPVAIEWKRPPKFILDDADRTDICQGGDCWLLAAIASLTLKKDALTRVLPHDQEFDHRYAGIFHFQFWQHNKWLDIVVDDRLPTVRNKLIMLHSASNNEFWSALLEKAYAKLHGSYESLKGGSTLEAMEDFTGGVGELYETKKSP
LFSIMKKALDRGSMMGCGLVKGHAYSITGLEEYRGRTVQLIRIRNPWGQVEWNGPWSDREWSYIDAAEKRRINEDGEFWMEFDDFKANYDRVELCNMTPDALHWEVNTFEGSWIRGSTAFWTNPQFKLRLEDCSVVIALMTIGFA
VYFYINTREVSERFTLPPGEYLLVPTTFQPHHEADFLVRMFSEAISVRELQQMLNGVLSTCHSIINLMDTGMLEFQEFKVFWEKMKKWILFLSFDTDRSGKMSSYELRIALKAAGQLNNSLLQLIGLRFADYDIDFDDYLTCIVR
LENMMNV
>Oryzias_latipes_ENSORLG00000019722
KTFEQLRQECLQKGVLFEDPDFPAVDSSLF-
VPVQIQWKRPPKFIVGGADRTDICQGGDCWLLAAIASLTLKKDAMARVVPPDQDFDHNYAGIFHFQFWQHNKWLDVVVDDRLPTVRNRLIMLHSASNDEFWSALLEKAYAKLNGSYESLKGGSTMEAMEDFTGGVGEMYETKSAP
LFSIMKKALDRGSMMGCGLVKGHAYSITGMEEFRGQTVKLIRVRNPWGQVEWNGAWSDREWNYIDKKEKDRINEDGEFWMEFEDFKKNYDKVEICNMTPDALHWAVNVFEGNWIRGSTAFWTNPQFRLQLEDCSVVIALMTIGFA
VYFYINMREVSERFTLPPGKYLLVPTTYQPHQEADFIIRIFSEAISVRELQQMLNGVLSTCHSIINLMDTGMIEFQEFKVFWEKMKNWILFLAFDTDRSGKMSSYELRSALKAAGQLNNQLLQLVGLRFGDFNIDFDDYLTCIVR
LENMMNV
>Xiphophorus_maculatus_ENSXMAG00000016015
KSFEQLRQECLQKGVLFEDPDFPAADASLF-
VPVQIEWKRPPKFIVGGADRTDICQGGDCWLLAAIASLTIKKEALARVVPHDQDFDRGYAGIFHFQFWQHNKWLDVVVDDRLPTVRNRLIMLHSASSDEFWSALLEKAYAKVHGSYESLKGGSTMEAMEDFTGGVGEMYETKSAP
LFTIMKKALDRGSMMGCGLVKGHAYSITGLEEFRGQTVKLIRIRNPWGQVEWNGRWSDREWDYVDKAEKTRINDDGEFWMEFEDFKRNYDKVEICNMSPDALHWEVNVFEGNWIRGSTAFWTNPQFKLQLEDCSVVIALMTIGFA
VYFYINMREVSERFTLPPGKYLLVPTTFQPHQEADFIIRIFSEAICVMELQEMLNGVLSTCHSIINLMDTGKMEFQEFKVFWDKMKKWILFLSFDTDRSGKMSSYELRTALTAAGNLNNQLLQLIGLRFADYEIDFDDYLTCIVR
LENMMNV
>Oreochromis_niloticus_ENSONIG00000000043
```

KSFEQLRHECLMKGVLFEDPDFPAKDSSLF-
VPVQIEWKRPPKFILDGADRTDICQGGDCWLLAAVASLTMKKEALARVVPPDQEFDQRYAGIFHFQFWNHNKWLDVVVDDRLPTVRNQLIMLHSASSNEFWSALLEKAYAKLNGSYEALKGGSTMEAMEDFTGGVGEMYETKAAP
LFNIMKKALDRGSMMGCGLVKGHAYSITGVEEARGQKVKLVRIRNPWGQVEWNGAWSDREWNYVDSAEKTRINDDGEFWMEFEDFKRNYDKVEICNMSPDSLQWAVNVFEGNWIRGSTAFWTNPQFKLLLNDCSVVIALMTIGFA
VYFYINMREVAERFTLPPGHYLLVPTTFQPHHEADFLIRVFSEAISVRELQEMLNGVLSTCHSIINLMDTGKIEFQEFKVFWEKMKKWILFLSFDTDRSGKMSSYELRGALKAAGQLNNQLLQLVGLRFADYEIDFDDYLTCIVR
LENMMNV
>Gasterosteus_aculeatus_ENSGACG00000019761
KSFEELRHECLQKGVLFEDQDFPAADSSLY-
VPVNIEWKRPPKFIVGGADRTDICQGGDCWLLAAIASLTLKKEALARVVPPDQDFDRRYAGIFHFQFWSHNRWLDVVVDDRLPVVRNKLIMLHSASNDEFWSALLEKAYAKLHGSYESLKGGSTMEAMEDFTGGVGENYETKNCP
LFSIMKKALDRGSMMGCGLVKGHAYSITSLEEFRGKTVQLIRVRNPWGQVEWNGPWSDREWNYVDSGDKNRLLDDGEFWMEFSDFKKNYDKVEICNMTPDDLQWAVSMFEGNWIRGSTAFWTNPQFKLKLEDCSVVIALMTIGFA
VYFYINVREISERFTLPPGSYLLVPTTFQPHHEADFIVRVFSERVSVCKIIEILNKKKHTCHSIINLMDTGQLEFQEFKVFWEKMKKWILFLAFDTDRSGKMSSYELRSALKAAGQLNNRLLQLIGLRFADYDIDFDDYLTCIVR
LENMIQS
>Danio_rerio_ENSDARG00000012341
KTYEQLRRECLEKGKLFEDPDFPAVDGSLF-
VPVNFEWKRPPQFITGGATRTDICQGGDCWLLAAIASLTLKEETLNRVVPNDQTFDRGYAGIFHFQFWQHNKWMDVVVDDRLPCVRDKLVFLHSADNNEFWSALLEKAYAKLNGSYEALKGGSTMEAMEDFTGGVGEMYETKNGP
LFLILKKAIERGSMLGCGLVKGHAYSVTGVEEYRGAKVQLIRVRNPWGQVEWNGPWSDREWTVIDSSEKKRLNDDGEFWMEFGDFKSNYDKVEICNLSPDALQWAVNLFEGSWIRGSTAFWTNPQFKLHLENCSVIVALMTIGFA
IYFYINVREVSERFRLPPGNYLLVPTTFQPHKEADFLIRLFSEVISARELQHVLNNVLGTCISIINLMDSGMMEFSEFKVFWDKLKKWILFLSYDVDRSGTMSSYELRSALNAAGKLNNRILQLLGLRFADLEIDFDDYLTCIVR
LENMMNV
>Latimeria_chalumnae_ENSLACG00000001048
KTYEELKRECLRRGQLFEDPDFPADDSSLF-
PSVPFTWKRPPQFIVGGATRTDICQGGDCWLLAAIASLTLNEKTLSRVIYPAQNFGDDYAGIFRFQFWQNNEWLDVIVDDRLPTFRNRLVFVHSAELNEFWSALLEKAYAKLYGSYEALKGGSTLEAMEDFTGGVGEMYNTKESP
LYQIINKALKRGSMLGCGLVKGHAYSITGLEEYRGDTVQLIRVRNPWGQVEWNGPWSDAEWSRIDPSVRKRLIDDGEFWMSFEDFKKNFDKIEICNLTPDSLKWDVTVFQGSWVRATTAFWTNPQFKLKLMECTFIAALMTVGFS
IYFYINMREVSDRFSLKCGEYVIIPTTFKPEEEADFFLRIFSEKTNIPSVDH-
LKTVSLNVILFSLITPSMKLGFPEFKVFWGKLKHWLIFLKYDHDKSGSMSGHEFRLALNNAGQLDNYLLQLVVLRYMNLQIELDDFISCLVRLEQSMNI
>Scyliorhinus_canicula_TranscriptomeContig20672
TLYQELKNQCLKQGVLYEDPEFPAEESSLF-
PAVAFEWKRPPKFIVDGASRTDICQGGDCWFLAAVASLTLNQKIMGQVVPLEQTFDRNYAGIFYFRFWQYNEWVEVIIDDRLPTFRNRLVFLHSASNDEFWSALLEKAYAKLYGGYESLKGGNTLEAMEDFTGGMGETFKLKEPP
MYTLFHKALKRGSMLGCGLVKGHAYSVTGLDQSRGRPVQLIRVRNPWGQVEWNGSWSDTEWKTVDAADNRRLVDDGEFWMSFDDFKKHFDTVEICNLTPDSLKWEVTVFEGAWTKGCTAFWINPQYKVQLLECTFIVALMTVGFA
IYFYINVRENSQRFKLPPGQYVIVPTTFQPEAEAEFIVRFISEEINAYELQDVLNTVFSTCRSIVSLYHAEGLGFDEFKIFWNRMKQWKAFLYCDHDRSGTMSAHELRTAVKNSGQINNQLLQLLVLRYTDLQIDFDHFLRCMVR
LETSMDI
>Leucoraja_erinacea_TranscriptomeContig91257
TVFQELKQHCLKEQVLYEDPEFPAVESSLF-
PPVAFEWKRPPKFFVGGASRTDICQGGDCWLLAAVASLTLNEKLLHRVIPPNQTFGRDYAGIFHFQFWQSNKWVDIVIDDRLPTFKNRLVFLHSASSDEFWSALLEKAYAKMYGSFESLKGGSTLEAMEDFTGGMGEIFDLKNAP
QFSVIKKALHKGCMLGCGLVKGHAYSITGLDQYRGRPLQMIRIRNPWGQVEWNGPWSDPEWKAVDATDFRRLTDDGEFWMSFDDFKRHYSKVEICNLTPDSLKWEVTCFEGMWTTGCTAFWTNPQYKIKLLECTMVVALMTIGFT
IYFYVNLREICERFKLPPGEYIVVPTTFKPNDEAEFLVRIFSEEINAYELQGILDNVFGTCRSIVSLYNGKGLGFDEFKILWQRLSVWKAFLRCDHDKSGTISAHELRSAVKDVGFLNNQLLQLLVVRYADRQIDFDNYLRCMVR
LETSMNI
>Callorhinchus_milii_TranscriptomeContig81212
----------------------------------------------------------------------------------
PPKQNFQENYAGIFHFWFWQYNEWVEVVVDDRLPTFRDRLVYLHSAEQNEFWSALLEKAYAKLNGNYESLKGGNTLEAMEDFTGGLGEMFDLNESSMYATIQKALQRRSMVGCGLIKGHAYSVTGLQEYKGKPVRLIRVRNPWGQ
VEWNGAWSDAEWKMIDTSEHRTLVDDGEFWMAFEDFCKHFGKLEVCNLTPDSLKWNVSVYEGKWQKGSTAFWTNPQYKLRLHDSTLMIAILTIGFAIYFYINVREICQRFQLPPGDYILVPTTFQPHHEADFVIRIFSEEIDAYE
LQKILSTVFATCRSIVSLYARRMLGFEEFKYLWTRMKMWKAFLKCDNDNSGTISSYELRSAIEEAGQVNNQLIQLLVLRYANMEIYFDNFIRCLVRLETSMNI
>Lamprey_ENSPMAG00000003440
---------------------------------------
RAPPQFIVNGATRTDIKQGGDCWLLAAIASLTLHPRLLARVVPEGQSFSDGYAGIFHFQFWQFGEWVDVVIDDLLPTQNNKLAFLHSTDRNEFWSALLEKAYAKLNGSYEALKGGSIAEAMEDFTGGVFEFYNLKKAPLYRIMRK
GLEKASMMGCGLVKGHAYSITGLDQFEGQDVRLVRIRNPWGEVEWNGAWSDWEWRVVDVSERRRLVEDGEFWMSFSDFKRNFSKMEICNLSPDTLHWTVTAHEGRWLRGCTAFWTNPQFHLRLHECSFIVALMTIGYAIYFFINL
REVSDRFRLPPGHYVIVPSTYEPHQEADFLLRVYCEEISPRELQDLLNKVTAACQSMVALMDSGKLGLPEFKLLWEKIKMWQIFRKYDEDRSGTMSSYEMRAALNAAGHLNNQMYQLITMRYADMTIKFNDYITCMVRLEAMMYA
>Lamprey_ENSPMAG00000007039
----------------------------------------------------------------------------------------------------------------------------------------------R----
LQGSYEALSGGSTTEGFEDFTGGVAESIELNKPPLWRMLQQATLRGSLMGCKLVKGHAYSLTGTTEYQGQMVRLVRIRNPWGQVEWTGAWSDPQWGYVSDEDRGRL-

EDGEFWMSFEDFLRQFSRLEICNLTPDALHWSANNFEGSWVRGCSALWEKWKFHLKHTECTFIVALMTVGYAIYYFINLREVSQRFTLPAGEYIVVPSTFEPNQEADFILRIFSEEISAKELQSILNRVVSACRSMVSLMDTAKL
GVKEFNFLWKKIKNWQIFLEYDTDSTGTMNSMEMRLALEAAGRLNEKLNQIVTTRYADITIDFDNYVCCLVRLETMMFA
>Xenopus_tropicalis_ENSXETG00000030338
QDYGQLKAQCLASNSLFEDEPFPASQASLGSKTKGIVWLRPPEFIISGATRSDIRQGGDCWFLSSIASLTLNEEYLSQVVPGDQSFQTNYAGIFHFKFWQYGEWVDVVVDDRLPTKKGKLVFVKSAEGNEFWSALLEKAYAKKAG
SY-----------ISPVC----------PPPLAKVMPKAISPNPLCAANVVKNHAYTITRAEEYRGEKVQLIRLRNPWGKTEWNGAWSDPEWDDIDSETRAAL-
DDGEVWMPFSDFISEFYRLDICNLSLDCVRWCLTQFYGSWKSGCTAFWINPQFRIKLEECTVIVALIAVGYYLYFYKKHRETCCRHKLPVGEYVLLPHTYYPCQEADFCLRVFSEEMSAEELRINLSRILSTCKEIINLFDTGKL
SYKEFRPLWIKLDKYTIFKSADNDRSESIEAHEMRNALQQAGNLNNKIQEAIVQRYITLSISFDSFIACLIRLETLMM-
>Xenopus_tropicalis_ENSXETG00000002784
QDYHKLVQECLQQGRLFEDPHFPADAKSLGSVTRGIQWRRPPKFITENMKWTDVCQGGNCWFLAAAASLTQYPLLMARVVPPQQGFKDRYVGIFHFQFWQYGEWVDVVVDDRLPVKNGQLVFVSSAQKSEFWAALLEKAYAKLNG
SYEALNGGFMNEAFVDFTGGLDETVDLKVPPLYHLIEKAVKKRSLMGAGLVKGHAYSIIATWKQNGRTIHLLRLRNPWGKVEWNGRWSDPLWSQVTFELREKM-
EDGEFWMQMEDFLRFFDILEVCNLTPESMLWNTNTFSGRWMMGHNAFWTNPQYIVTLTECTLLVSLMVVGFEIYWFVAKRDVTERYQLPPGRYLLIPSTFQPHQESDFILRVFTEEISPEELQRILVQTTSTCRLLIKITDNGKL
QLEEFRQLWFKIKEWEIFTKYDKDRSGTMDVQELRLALEAAGTLNNQLVESLCQKYGDRQVDFDSFLSCLAYLVCVHFN
>Sus_scrofa_ENSSSCG00000002967
QSYRAIQAACLDEGILFRDPYFPAGPDALGSKAKGVEWKRPPQFICEDMSRTDVCQGGNCWFLAAAASLTLYPRLLCRVVPPGQGFQDGYAGVFHFQLWQFGHWVDVVVDDRLPVREGKLVFVHSAQRNEFWAPLLEKAYAKLHG
SYEVMRGGHMNEAFVDFTGGVGEVLYLRQNALFAALRHALAKESLVGAGLVKGHAYSVTGTHKLGFTKLRLLRLRNPWGRVEWTGAWSDPRWDELPTEWRDAL-
EDGEFWMELQDFLCHFDTVQICSLSPEVLGWHIHTFQGRWVRGFSSFWTNPQFRLTLLECTVLLSLITVGFHVF-
FCARRDVSRRCRLRPGHYLVVPSTARAGDEADFTLRVFSEELRAPQLQTLLSIALETCEQLLRCFGGRSLALYHFQQLWGHLLEWQTFDKFDEDASGTMNSYELRLALNAAGHLNNQLTQALTSRYRDLRVDFERFVSCMAQLLC
LTFS
>Homo_sapiens_AK127398
QSYEAIRAACLDSGILFRDPYFPAGPDALGSKAKGVKWMRPPKFICEDMSRTDVCQGGNCWFLAAAASLTLYPRLLRRVVPPGQDFQHGYAGVFHFQLWQFGRWMDVVVDDRLPVREGKLMFVRSEQRNEFWAPLLEKAYAKLHG
SYEVMRGGHMNEAFVDFTGGVGEVLYLRQNSLFSALRHALAKESLVGAGLVKGHAYSITGTHKLGFTKVRLLRLRNPWGCVEWTGAWSDPRWDTLPTECRDAL-
EDGEFWMELRDFLLHFDTVQICSLSPEVLGWHVHTFQGRWVRGFNSFWTNPQFRLTLLECTVLLSLITVGFHVF-
LSARRDVTRRCCLRPGHYLVVPSTAHAGDEADFTLRVFSEELNASQLQALLSIALETCEQLLQCFGGQSLALHHFQQLWGYLLEWQIFNKFDEDTSGTMNSYELRLALNAAGHLNNQLTQTLTSRYRDLRVDFERFVSCVAHLTC
ITFS
>Mus_musculus_ENSMUSG00000054083
QNYEAIRRACLDSGILFRDPCFPAGPDALGSKAKGVEWKRPPQFICEDMSRTDVCQGGNCWLLAAAASLTLYPRLLYRVVPPGQGFQDGYAGVFHFQLWQFGRWVDVVVDDKLPVREGKLMFVRSEQRNEFWAPLLEKAYAKLHG
SYEVMRGGHMNEAFVDFTGGVGEVLYLRQNTVFAALRHALAKESLVGAGLVKGHAYSVTGTHKLGFTKVRLLRLRNPWGRVEWSGPWSDPRWDMLPSEWRDAL-
EDGEFWMELQDFLTHFNTVQICSLSPEVLGWHIHIFQGRWVRGFNSFWTNPQFRLTLLECTVLLSLITVGFHVF-
FCARRDVSRRCRLPPGHYLVVPSASRVGDEADFTLRIFSEELNALQLQTLISIALETCEQLVQCFGGQRLSLHHFQELWGHLMSWQTFDKFDEDASGTMNSCELRLALTAAGHLNNQLTQSLTSRYRDLRVDFERFVCCAARLTC
ITFS
>Monodelphis_domestica_ENSMODG00000013388
QCYHAIQDCCLQAGTLFLDPCFPAGPSALGSKAKGVEWLRPPQFICENMDRTDVCQGGNCWFLAAAASLTLYPRLLHRVVPPGQSFQWGYAGVFHFQLWQFGHWVDVVVDDRLPVRDGKLLFVRSAQRAEFWASLLEKAYAKLHG
SYEVMRGGHMNEAFVDFTGGVGEVLYLQPNTLFSALRHALAKESLVGAGLVRGHAYSVTGTHKLGFAKVRLLRLRNPWGHVEWSGAWSDPRWAALPEAWREAL-
EDGEFWMGLEDFLQHFNTVQICSLSPEVLGWHVHTFQGRWVRGFNAFWTNPQFQLTLQDCTVLLSLITVGFHVF-
FSIRRDVSRRCRLAPGHYLIVPSTAHVGEESDFTFRIYTEAISTAQLQALLSLALETCEQLIHCFGGHCLTLPEFQQLWYRLQGWQTFNKFDADKSGTMNSHELRLALNTAGQLNNQLTQLLTSRYRDLRVDLDRFVSCVTQLTC
LTFS
>Anolis_carolinensis_ENSACAG00000011727
QNYQELKRQCLQQQRLFLDPEFKPCAESLGSNIQGVVWKRPPHFICKGMNRTDVCQGGDCWFLAAAASLTLYPELLYRVVPQDQHFGTEYAGIFHFQFWQYGQWVDVVVDDLLPTINNELLFVRSPEHDEFWMPLLEKAYAKLNG
SYEAMNGGYMNEAFVDFTGGIGETLSLKIPNLFKTIRAALSRNSLMGAGLVKGHAYSITGIHKFGEKVVRLLRLRNPWGYQEWTGRWSDPLWSSLDPELLKKLRKDGEFWMQLVDFIRHFDVLEICHFSGDHTCWNVNCFQGCWI
KGYTAFWMNPQFHVSLLECTFLVSLMLICFQIFVNGYSRDITRYLQLPPGDYLIIPNTQSPLEEANFTLRIFTEEVDANQFQSILNQTMTECEKIIQHFSTNKLTLEDFQQFSCRMQEWQIFLAYDVDRSGSMNTHEIQLALDAA
GHLNNRTTEALVKKYGNLQIDFDSFVSLMVHLESVM--
>Xenopus_tropicalis_ENSXETG00000022056
QDFDSIRESCYSRRQLFEDETFPASVSSIG-
QLLSIKWERPARLLVDGASIFDMVQGGDCWVLAAVGALTLHQKFLDIVIPKDQEFNYKYAGVFHFRFWQFGEWVDVVIDDRLPTLNGKYLSVHPRSDNEFWPTLLEKAYAKLRGSYQNLHWGYISEALVDFSGGVLVEFDLTKPP
LRDIVIAAAKSGSLMNCGLVQGHAYTVTDATQYKNGTEDLVRVWNPWGKGEWNGRWSDPQWDRVRADVRQKL-NDGEFWMSCQDFLQNFSCASICNHTPAYFTWQTLTYFSRWVRGSTALWRNPQFVISVSD-NVTVALM-
IGFALCERAHYREVTRSFMAPPGTYVVIPFTESKGQESEFLLRIFLKELYAEQLQRLLNEVIIACRGILMLMDNGRLSLQEFGRLWKRLNMCKMFRSIDGNQTGFIDASGLKKAVQLA-
ELDNALINVMVLRYANEKLSFADFVCCMIRLETVMSS
>Gallus_gallus_ENSGALG00000020084

```
QDFEHLRSLCLSQGLLFEDDIFPADTSSIG-
KLQQIEWKRPPCLIMDGVSRFDIIQGGDCWMLAALGSLTMQKQFLEKVLPKDQGFQSDYAGIFHFRFWQFGDWVDVVIDDRLPFLNGNYLSVHARTSNEFWPSLLEKAYAKLQGSYQNLHGGYISDALVDFTGGVQMQFSLKDPP
LEDVLKAASKSRCLMGCGIVQGHAYTVTGAVKFKNQWEHIIRVWNPWGHGEWRGPWSDPEWDYVEPEIKEEL-NDGEFWMSYENFREQFSWLCVCNCTPTFLGWSVERHINLWSPLLATVSKNPQYFFKVTK-NVVISLI-
IGFFIT-FS-LRDVSSCFNLSPGTYAVTPATTED-REFEFVLRIFVKAIDALQLQRLLNDMVLSCRAILALMDNGQLTLQEFGSLWRSVTKYMLFRREDRNCSGFLDVYELKSAIQTA-
PVNEQILHLMALRYGNKRMGFADFVSCMLRLETMMYI
>Meleagris_gallopavo_ENSMGAG00000005407
QDFEHLRSLCLSQGLLFEDDTFPADISSIG-
ELQQIVWKRPPFLIMDGVSRFDIIQGGDCWMLAALGSLTMQKRFLENVLPKDQGFQSDYAGIFHFRFWQFGDWVDVVIDDRLPFLNGNYLSVHPRTSNEFWPSLLEKAYAKLQGSYQNLHGGYISDALVDFTGGVQMQFSLKDPP
LEDILKAASKSQCLMGCGIVQGHAYTVTGAVKFKNHWEHIIRVWNPWGHGEWKGPWSDPEWDYVEPEIKEEL-NDGEFWMSCESFREQFSWLCVCNCTPTFLGWSVDRHINLWSPLLTTVSKNPQYFFKVTN-NVVISLI-
IGFFITNFS-LRDVSSCFNLSPGTYAVIPATTED-REFEFVLRIFVKAIDALQLQQLLNDMVLSCRAILALMDNGQLTLQEFGSLWRSVTNLCLFKREDRNRSGFLDVSELKSAIQTA-
PVNEQILRLMALRYGDRRMGFADFVSCMLRLETMMYI
>Pelodiscus_sinensis_ENSPSIG00000005500
QDFTQLRDYCLRRRLLFEDETFPAHVSSIG-
KLSHIQWKRPPHLIMDGASRFDILQGGDCWVLAALGSLTLQRQFLENVLPKDQGFHHSYAGIFHFRFWHFGDWVDVVIDDRLPFLKGKYLSVRPRCKNEFWPSLLEKAYAKLRGSYQNLHLGYISEALVDLTGGVQVQFSLQSPP
LQEILKAAVKSQCLMGCGLVRGHAYTVTGAEEYQYSQEEIIRVWNPWGHREWKGPWSDPEWDQIPAEYKKAL-DDGEFWISRRDFVKQFFSLCICNVVPSFLTWAMTAYVNQWVRGLTAFSRNPQYFIQIEE-NVVVSLM-
IGFFIFFFNGQRDVTDCFFLSPGIYVVVPVTSEEGQEAEFLLRIFLKDMNASQLQRLLNEVFLSCRGILALMDNGRLSLKEFKHLWKLLVKYKIFRMEERTHAGFLDVSDFRPHLIGSKAVNDQLFHLMALRYSDMRVSFPDFAC
CMIRFETMMYC
>Anolis_carolinensis_ENSACAG00000007376
QDFVYLTDYCLKHGILFEDDTFPAHYSSIG-
KLRRIKWLRPPRLFVDGVNRADILQGGDCWVLAALGSLTRQQRFLKNVIPKDQGFEDSYAGIFHFRFWQFGDWVDVVIDDRLPFLDDNFLFVHPRSKNEFWPPLLEKAYAKLRGSYTNLDQGYLTEALVDFTGGVEMPFNLKNPP
LFEMLKTAAESGCLMGCGIVQLHAYTVIWATEYMDGKERLVRLWNPYGNTEWKGAWSDVEWQHVPIPYRKKL-DDGEFWISYKDFRDNFSFLIICNDVPTCLTWSVDKHVNKF-SGASLLSRNPQYFIQVPE-NLVVALS-
IGFVIV-LQPQRDVVNCFYLSPGTYIIIPTTTQEGEGTEFLLRTFLKFLDASQLQRILNEVLLSSKSLLALMDNGRLSLDEFGELWRELNIYKIFIKEDEQNSGFLNASNLKRIIQER-
SVSEKLLGLMTVRYGDMRMNYPGFVCCMIRLETMMYC
>Latimeria_chalumnae_ENSLACG00000011037
QDFENLREECLSQGVLFEDKTFPPGVESIG-
QLRNLTWKRPPQLYVKGASRFDIKQGGDCWFLAALGSLTCHDHHLEWILPKNQGFSKDYAGIFHFRFWHFGQWADVVIDDRLPMNGERYLFVSPRQSNEFWPALLEKAYAKLCGSYQHIDGGNIADALVDFTGGIKKDIYLKKKP
VWEMMKRSDKLKCLMAAGLVEGHAYSVTGIAQYKGRTERLIRLWNPWGQEEWKGRWSDVQWERISKEEQDNL-EDGEFWMALQDFRDYFNGLVISDANPEFMKWMVRTYENGWVAGVSAFLKNPQYKLQLKK-
NVTVSLIGISIVLHLLNQPREISCKFILEPGTYVLIPCTNKAEQEAGFILRVFYR-------------------------------------------------------------------------------------------------
--------------------
>Gadus_morhua_ENSGMOG00000009688
QDFQQLKKYCIERQLRYIDDMFPPNQNSIG-RLARVEWLRPPSFVVDGVSRFDFCQGGNCWFLASVGALTFQSQILQRVLPSELI----
QCWLFHFRFWRFGYWVDVVIDDKLPTIDGKLIFVRSKTPNEFWPALLEKAYAKVCGSYADMNAGTPSEALVDFTGGVHICFQLDSAVLWNTMHRAAQSNSLMGCGLVEGHAYAVTGVTQSNGQPINLVRLLNPWGNGEWIGDWSD
VLWQSISAEDREMC-DDGEFWMAMEDFCRFFSDLDICCLCPDFL-WTPLVYESRWVAGTTAFRMNPQYRFRIEG-NILVSLM-
IGFSIFFHLNSREVMDMFKLAPAEYLIVPSSFKPDETASFILRILSKEVHAEQLQNILNEKILACRSMVALMDTGKLKRDEFLRLWRKVTTYNIFSQIDPTRKGTLSLHELRKAIKAA---
KDGMLNLMAVRYGTGRISLESYINLLLRLECM---
>Takifugu_rubripes_ENSTRUG00000002373
--------------------------------------------
PSFSVDGMSRFDFGQGGNCWFLASIGALTFQRPILEQVVPLDQSFDEEYCGLFHFRFWRFGKWIDVIIDDKLPTINGRLLFVQSKTPNEFWPALLEKAYAKVCGSYSDMNAGSPAEALVDFTGGVHTVLQLSDPPLWELMFRAGK
SKSLMGCGLVQGHAYTVTGVKQSQGRQVNLVRLWNPWGQGEWNGDWSDRLWNTVSSEDREMC-NDGEFWMALEDFCRFFADLDICCLCPDFL-WRSSCYEGRWVAGITAFWTNPQYRVKVDK-TMLVSLM-
IGFSVFFYVNAREVMELMALKPGEYLIVPSTFNPNETASYLLTILSKEVDAEQLQKLLNERILACRSMVALMDTGKLNQREFLRLWRKILALKIFVRTDDSNTGTLPLNKLRNAVEAT-
RLNDDLLNLMALRYGSGSITLENFISLMLRWESMSFS
>Oryzias_latipes_ENSORLG00000019014
QDYKQLKDFCLISGMRYIDDMFPPDSRSIG-
NMAQVKWLRPASFVVDGVSRFDFGQGGNCWFLASIGSLTFQTNILNQVVPLEQTFDDEYCGLFHFRFWRFGRWIDVIIDDKLPTINGKLIFVRSKDPTEFWPALMEKAYAKVCGSYADMNAGTPAEALVDFTGGVHMCVQLSDPP
LWELMCRAGRSRSLMSCGLVQGHAYTVTGVKHSRGETVQLVRLWNPWGKREWNGDWSDPLWKTVSTEDREVC-DDGEFWMTLKDFCKNYSDLDICSSNPDFL-WKTSFYEGRWVAGTTAFWTNPQYRVQVEK-NMLVSLM-
IGFSIFFFLNAREVMEFVTLKPGEYLIVPSTYNPNETASFLLTIISKEVDAEKLQMILNDTILACRSMVALMD-----------------------------------------------------------------------------
------------
>Gasterosteus_aculeatus_ENSGACG00000005683
```

QDYEQLKQYCLIQGVRFIDKMFPPDQRSIG-
DLSRVEWLRPPSFEVDGISRFDFGQGGNCWFLASIGSLTFQQQIFQQVVPLEQRFDEKYCGLFHFRFWRFGKWVDVVIDDKLPTINGQLIFVQSSDPTEFWPALLEKAYAKVCGSYSDMNSGTPDEALVDFTGGVHMCIELQDPP
LWELMCRAGQSKSLMGCGLVQGHAYAVTGVKQSNGTPVQLVRLWNPWGATEWNGAWSDSLWQTVSPQNREMC-DDGEFWMNLKDFCKFYSSLDICSLCPDFL-WKTAFYEGRWVAGTTAFWTNPQYCFKIDE-NMLVSLM-
IGFSVFFYKNAREVMAFFALKPGEYLIVPSTFSPNETASFILTIVSKEVDAEKLQRLLNEHVLACRSMVALMDTGKLNDEEFVHLWNKVVGYKVFFRSDVSQTGTLSLIELRNAITAS-
TINDEILNLMALRYGAGHITLESFISLILRFDRVMYT
>Xiphophorus_maculatus_ENSXMAG00000000065
QDFQQLKEYCVIRRVRFIDDMFPPDHRSIG-
DLVRVEWVRPPSFIVDGVSRFDFGQGGNCWFLASIGALTFQDLALKQVVPLEQTFDEDYCGLFHFRFWRFGKWVDVVIDDKLPTINGQLIFVHSKNPTEFWPALMEKAYAKVCGSYADMNAGTPVEALVDFTGGVHMCVQLSEPP
LWELMCRASQAKSLMGCGLVQGHAYAVTGVKESRAKAERLVRLWNPWGQGEWSGDWSDPLWQTVSPQDRETC-EDGEFWMTLQDFCKFYSDLDICCSCPDFL-WKSSFYEGRWVAGISAFWTNPQYRVEVAG-NMLVSLM-
IGFCVFFYPNAREVMQLLSLKPGEFLIVPSTFGPNETASFILTVLSKEVDAEQLQSLLNGKILACRSMVALMDTGKLNSEEFVSLWRKIVSYKIFSHCDVSQTGTLSLSELRKAIMAS-
TISDGMLNIMALRYGAGFITLENFIALVLRFDRMMYS
>Oreochromis_niloticus_ENSONIG00000008267
QDFHQLKQYCLIRHLRYIDEMFPPDRNSIG-
DLARVVWLRPPSFVVDGVSRFDFGQGKNCWFIASIGALTFQNHILGQVVPLEQNFDEDYCGLFHFRFWRFGKWVDVIIDDKLPTIDGRLIFVHSRDPTEFWPALLEKAYAKVCGSYTDMNAGTPAEALVDFTGGVHIYIDLSKPP
LWEMMCRAGQSKSLMGCGLVQSHAYAVTGVKQSRGQTVNLVRLWNPWGHGEWNGDWSDPLWQTVSPQDRDMC-DDGEFWMSLKDFCKTYSELDICCMCPDFL-WNTSIYQGRWVAGTTAFWTNPQYRVKIDE-NMLVSLM-
IGFSVFFYINAREVMELMMLKPGEYLIVPSTFSPNETASFILTILSKEVDAEQLQRLLNGKILACRSMVALMDTGKLNGEEFVRLWKKVVTYQR-----------------------------------------------------
-----------
>Oryzias_latipes_ENSORLG00000019002
QDFQQLAKYCLSQRVKFLDETFPPERSSIG-
DLAKVEWLRPPSFVLRGVSRFDFGQGGNCWFLASIGALTFQKEIFNQVVPLEQTFDQNYCGLFHFRFWRFGKWVDVVIDDKLPTLNGRLIFVRSQSQNEFWPALLEKAYAKVCGSYTDMTAGTPSEAMMDFTGGVHIYIQLSEPP
LWELMCRAGKSGVLMCCGLVLGHAYTVTGVIQSRGKPVNLVRLWNPWRTGEWRGDWCDPLWQTVSPKDREKC-DDGEFWMTLEDFCKLYSDLDICGMDPNFL-WKTSVHEGRWVPGTTAFWTNPQFRVQISG-NTFVSLI-
IGFSIFFFMNAREVMEFIRLKPGEYLIVPSTFKPNESASFLLTIHSKEVDAEQLQKILNDQILACRSMVALMDTGSLNSKEFVRLWNKVIAFKIFFQTDVSRTGTLSLSELRNAIIAS-
KVNDELLNLMALRYSAGHVTLESFISLILRLECMMY-
>Xiphophorus_maculatus_ENSXMAG00000000132
QDFRQLKEFCVGRRLKFIDDMFPPDSKSIG-
DLARVVWLRPPSFVLQGFSRFDFGQGGNCWFLASIGALTFQDQILQQVVPQEQDFDENYCGLFHFRFWRFGKWVDVVIDDKLPTINGSLIFVHSKKQNEFWPALLEKAYAKVCGSYTDMNAGTPAEAMMDFTGGVHMSIQLSDPP
LWGLMSRAGNFSALLSCGLVQGHAYTVTGVVQSRGKPVKMLRLWNPWGKGEWNGDWSDSLWQTVSSQDREKC-DDGEFWMTLEDFSKFFTDLDICGLNPNFL-WTTSVAEGRWVAGTTAFWTNPQYRVKLCG-NILVSLM-
IGFSIFFFMNAREVMELLTLKPGDYMIVPSTFHPNETASFLLTILSKEMDAEQLQRILNENLLACRSMVALMDTGRLNSSEFIRLWNKVIIYKIFFQTDVSRTGTLSLTELRNALVAS-
RVSDDMLNLMALRYGAGHMTLESFISLILRLDCMMYT
>Oreochromis_niloticus_ENSONIG00000008269
QDYEQLKQYCLTRGVSFTDETFLPDRRSIG-
ELACVEWRRPPAFVLDGVSRFDFGQGGNCWFLASIGALTFQKSIFAEVVPLEQRFDENYCGIFHFRFWRFGQWEDVVIDDKLPTINGRLIFVHSKDQNEFWPALLEKAYAKVCGSYSDMIAGTLSEAMMDFTGGVHINIELSRPS
LWGLICRAGKSNTLMGCGLVAGHAYTVTGVKESRGKIVYLVRLWNPWGNGEWKGDWSDPLWQTVSPEDREMC-DDGEFWITFEDFCKFYTDIDICGLSPDFL-WKTSVYDGRWVAGTTAFWTNPQCRFKICG-NIMVSLM-
IGFFIFFYLNAREVMEFFMLKHGEYLIVPSTFNPNETASFILTIHAKEVDAEQLQRLLNENILACCSMVALMDCGKLNSEEFLRLWNKVVTFKIFFRTDISRTGTLSLRELRNAFLVS-
RVSDDMLNLMALRYGAGHMTLESFISLILRFDCMMYT
>Xiphophorus_maculatus_ENSXMAG00000000188
QDYLQLKRFLLTQNKLFRDETFPPDQRSIG-
ELAQVQWLRPPFFILDGVSRFDFGQGGNCWFLASLGALTFHKEIFKLIVPLDQTVGKDYCGLFHFRFWRFGKWVDVVIDDKLPTIKRKPIFARSKDEREFWPALLEKAYAKVCGSYADMTSGTPAEAMRDFTGGVHMCIQLSDPS
LWKLLCRAGRSRTFMSCGLVPGHAYTVTGLKQSQETEVNLVRLWNPWGHGEWNGDWSDPLWRSVSTKDREKC-NDGEFWMSLEDCCRYYTNIEICGMRPDFL-WKTSMYENRWVAGTTAFWTNPQYRIKVCG-NTLVSLM-
IGFIIFFLMKSREVTEFLTLKPGEYVIVPCTNEPNQTASFLLTIFSREVDAELLQQLLK----ACRSMVALMDDGKLDSQEFGYLWHKVMKYKVFAKMDVSQTGTLSLTELRNALRDS-
SISDELLNLMVVRHGAGHMTLENFISLSLRLSRMMYT
>Takifugu_rubripes_ENSTRUG00000001713
QDYEQLKQYCNIRGLRYIDERFPPDRNSIG-
DLDRVVWLRPPCLIIDGISRFDFGQGGDCWFLASLGSLTFQSDILKQVVPVEQTFQESYCGLFHFRFWRFGGWVDVVIDDKLPTINGRLIFVHSKDPNEFWPALLEKAYAKVCGSYADMNAGSPAEALMDFTGGVHVTVQLSDPP
LWELLHPLTQ--TLILTGLVEGHAYTITGVKEHQGKVVRLVRLWNPWGKGEWKGDWSDPLWRTVSAEDRKLC-EDGEFWMPFKDFCSFFTDLDICCLCPSFL-WRTSMFEGRWVSGVTAFWINPQFRLKLHG-NIFVSVT-
IGFSVFFFLDAREVMEVFRLKPAEYLIVPSTFGPNETASFLLTIVYKEVNAEQLQRLLNDQILACRSMVALMDHSKLNSEEFVALWKNVIKYRSWT-----------------------------------------------------
-----------
>Danio_rerio_ENSDARG00000008553

QDFQQLKQFCLKERLRFRDNLFPPELKSIG-
DLWKVVWKRPPVYIAQGTSRFDFIQGGNCWFLASVGALTFQKRIMKQVIQDDQTFSVDYAGIFHFRFWRFGSWVDVVIDDKLPTIDNQLVFVQSKTPNEFWPALLEKAYAKVCGSYADMDAGNISEALMDFTGGPHMTIKLSQAS
LWDIMRRAGKSESLMGCGLVEMHAYTVTGVTECKGRPVKLVRIFNPWGSGEWNRDWSDPLWELVRPEDQKY--DNGEFWMSMEDFCRNFSEMDICCSDVNVL-WKTEVHKGQWVMGTTAFSKNPQYRVTLKE-NLLVSLI-
IGFNIYFFLNAREVMEFFRFKAGDYLIVPSTFQPNEASSFLLTVYSKEVDAERLQKLLNENLHLCKSLVALMDTGRLSEFECLRLWKRAVFLKIFYDMDVSHTGSLSVNELRNALKVA-
VLSDGMLNLMALRYADGEISLENFIVLVLRMDCMLYS
>Danio_rerio_ENSDARG00000077217
QDYQELLKSCITNRMRFVDDRFPPDSSSID-
DMSQIQWLRPPQFIVKGVSRFDYSQGGNCWFLASVGTLTFQKDVLYQVMPEGQSFGKDYAGIFHFRFWRFGKWIDVVIDDKLPTIKGDLIFVHSKTSNEFWPALLEKAYAKVCGSYSDMDAGVVSEALLDFTGGIHVRFELEKPP
LWSLMDRAAKCKALMACGIVQGHAYSVTGIFKWQGAPVRLVRVLNPWGKREWTGAWCDSMWNKVSESERTKC-NDGEFWMSMEDFSKNFEEVDICCFTPDFL-WKTVRYNGSWEAGKTAFWTNPQFRVTIEK-NVLVSLL-
IGFCVFFFVNARHVMKFFKLEPGEYLIVPTTFKPNECAKFMLSIFSKHRKSRKPE------------------------------------YV-----------------------------------------------------
-----------
>Danio_rerio_ENSDARG00000091723
QNYQELQQKCLTESTLYSDELFPPDNSSIG-
DMSQIVWKRPPNLIVDDVSRFDYAQGGNCWFLASIGALTFEKDIMKQVLPAEQSFTKDYAGIFHFRFWRFEKWIDVVIDDQLPTIDDELLFVCSKTSNEFWPALLEKAYAKVCGSYADLNAGFISEALMDFTGGTHMYYTLSSAP
LWKIMECAFISKTLIGCGIIADHAYTVTGVFESEENPVQLVRLLNPWGEEEWQGDWSDPLWETVCEEARKCC-NDGEFWMSMEDFTRLFESIDICCLCPDFL-WTSKRHFGRWNAEKTAFWKNPQFRVTIDE-NILVSLM-
IGFYVFFLVWLREVMKFFRLEPGEYLIVPCTKNPGETASFVLSVFSKQIQ-ETTE-------------------------------------------------------------------------------------------
-----------
>Danio_rerio_ENSDARG00000052917
QDYNFLQDYCLKTRQRFVDEFFPPDLRSIG-
VMARVEWIRPAEFVVDTVSRFDYAQGGNCWFLASVGALTFQSKLLQKVVPDGQSLRHNYTGLFHFRFWRFGKWFDVVIDDKLPTINRQLIFVKSKTYNEFWPALLEKAYAKVCGSYADMHTGRVSEALLDFTGGVHMHYDLKTAP
LWEIMYRASQSEVLMGCGIVLGHAYTVTKVYQSGRNPVQLVRLFNPWGDSEWNGDWSDPLWNTVDDEDRKQL-DNGEFWMSMKDFLRTFDNMDICCNCPDFL-WTSKSHNGSWVPGSTAFCTNPQFWLRINE-NVLVSLI-
IGFCVFFFQDARQVMKFFRLEPGEYLIVPSTYLPNKNADFILSILCKVVDAEKLQQILHENLLSCRSMIAMSDTGRLQGSEFVRLWDRITTYRIFYNMDSSKDGVLSLNELQNALEKT-
HLNEDILNLMVVRYGGEQISLEGFICLVMRLNCMMYS
>Homo_sapiens_NM_14457
QDFTTLRDHCLSMGRTFKDETFPAADSSIG-RLSNVIWKRPPHFILDDISRFDIQQGADCWFLAALGSLTQNPQYRQKIL-
MVQSFSHQYAGIFRFRFWQCGQWVEVVIDDRLPVQGDKCLFVRPRQNQEFWPCLLEKAYAKLLGSYSDLHYGFLEDALVDLTGGVITNIHLHSSPLVKAVKTATKAGSLITCGLVSLHAYTVTGAEQYRRGWEEIISLWNPWGEA
EWRGRWSDQEWEETCDPRKSQL-EDGEFWMSCQDFQQKFIAMFICSEIPITLGWSQIMFRKQVILGNTAPRNDAQFNFSVQE-NVVVCVTPLDFQVIFNKFRRNFTMTYHLSPGNYVVVAQTRR--
KSAEFLLRIFLKDIDATQLQGLLNQELLECRSLVALMENGRLDQEEFARLWKRLVHYQVFQKV-QTSPGVLLSSDLWKAIENTGFISRELLHLVTLRYSDGRVSFPSLVCFLMRLEAMMYN
>Mus_musculus_ENSMUSG00000043705
QDFRTLRDHCLSRGQLFIDDTFPAAASSIG-HLSKLEWKRPPHFILEGASRFDIHQGGDCWFLAALGSLTQNPQCLQKIL-
MDQSYSHQYAGIFQFRFWQCGQWVEVVIDDQLPVIGDNFLFVHPRGNKEFWPCLMEKAYAKLLGSYSQLHYGYLPDALVDLTGGVVTIINLHSSPLLTTVKTAIQAGSMVACGLVSQHAYTVTGAEKYQGRWEDIIRLWNPWGKT
EWKGRWKDKEWRETHDPRKSQL-EDGEFWMSCQDFQENFSCLFICNQIPITMSWRQMRFTNQVISRNRAHGRDMQYVFSVQE-NVVVAFTPLQFQVFFYVSKCNFTKSFHLNPGTYVVVTTANG--
KEVEFLLRIFLKDIDATQLQSLLNQEFLQCQSIMALMDNGRLDQEEFARLRSRLIHCQIFQSI-QRRPGVLLSSDLWKVIENTGFISSELLSLMALRYSDGRVSFPTLVCFLIRLETMMYS
>Ornithorhynchus_anatinus_ENSOANG00000008244
-----------------------------------------------------------------------------------------------
FQFWQCGQWVDVVVDDRLPVQGQDYLFVRPRHSNEFWPCLLEKAYAKLHGSYFSLHSGHLSEALVDFTGGVAMWFNVQETPLVRRVQEASASGSLMACGLVSGHAYTVTGAKRYGNGWEAIIRVWNPWGSCEWKGRWSDEEWRKC
QDPRKNKL-EDGEFWISYQDFREHFSTLYICNETPIPLKWNLTKYENQRIQGNPIGRKNPLYFISVTE-
NVVVTFTPLSFQLFFFKSRRHVTKYFHLPPGTYVVVTFVNQEEREVEFFLRIFQKSIDPSQLQSFLNGEFLECRSIVALLDNGRLDRSEFDQLWKHLVTYQIFQKVDKKKSGFLLGSDLWKAVHESGSISDELLARMVLRYGDGR
ISFPDLVCFLLRLKIMMYS
>Sarcophilus_harrisii_ENSSHAG00000007666
-----------------------------------------
PHFILDGISKFDIRQGGDCWFLAALGSLTHKPKLLAKIIPINQSFTAGYAGIFHFQFWQCGQWVDVVVDDRLPVESRKYLFVHPRGNNEFWPCLLEKAYAKLHGSYSQLHYGYTIDSLVELTGWVVTKVNLKEVHLFKNLQVAEQ
SGSLITCGLVNNHAYTVTGSAKLDTCQEELIRLWNPWGEKEWTGCWCNREWQKVQESERTRL-EDGEFWMSFLDFQKTFASMLICFQYPMVMVWSQILNKVQKIQRNIILGNTLQYFFSVME-
NVVVSINASLIFNIYVTSKWNYTESFHLDSGTYVVVP---
TIEEGLNFYLQIFLKDIDASQLQSLLNKEFLECRSILALMDNGRLELKEFEQLWKKLIRYQIFRRVEKNGPGFLMSCDLWKVIRESGSINSELLDLMAIRYGDGKIYFADVICFLIRLEIMMYC
>Sarcophilus_harrisii_ENSSHAG00000003193
---------------------------------------------------------
GDCWFLAALGSLTHKPELLAKIIPTNQSFTHEYAGIFHFKFWQCDQWVDVVVDDRLPTSNEDCLFVHPRGNNEFWPCLLEKAYAKLHGSYSQLQSGYLIDSLVELTGWVATVITPK---
LFKNLQLAEQSGALITCGLVNGHAYSVTGSAELDSYQEKLIRLWNPWGEREWKGAWSDKKWQKIKESERTRL-EDGEFWFSLLDFQEIFTSMVICYQDPIIMAWCQSPYKVQKIKKYIMSGNNMQYFFNVTE-NVVVSIN-

```
MPLFIMHIRSKWNYTEYFHLDCGTYVVVP---
TNDRRLNFYLRIFLKDIDASQLQSLLNKELLECRSIVALMDNGRLDLEEFEQLWKKLLQYQIFRKVEKNGSGYLMGSDLMQVIKESGPINNELLNLMAIRFGDGKIYFADMICFLIRLQIKMYC
>Monodelphis_domestica_ENSMODG00000015519
QDFTSLRDHYLRNGMLFEDDMFPADDSAIG-
NLSSPIWYRPPHFILDGISKFDIQQGGDCWFLAALGSLTQRPKLLAKIIPPNQSFKQGYAGIFHFQFWQCGQWVDVVVDDRLPVKPNKYLFVHPRGNNEFWPCLLEKAYAKLHGSYSNLHSGSLADSLVELTGWIVTAIDLKKAD
MVQNLKVAEQCGSIITCGLVNNHAYAVTGTAELGTGPEELIQLWNPWGWGEWLGRWSDEEWWKIQDSERTRL-ADGEFWMSFQDFQMKFSHIYICYQEPMELTWIQIPCKVQKIQENINSGKNTQCPFSVTE-NVVVSMN-
ISMMIYYIKSKWNYTESIHLVPGNYIVVPTTNA--
RELRFFLRIFLKDIGASQLQSLLNKELLECRSILALMDNGRLDLEEFEQLWKKLIRCQIFQKVEKNASGFLLGSDLWKVIQEADSNNSELLDLMAIRYGDGKIYFSDLVCLLIRLEIVMYC
>Sus_scrofa_ENSSSCG00000020986
QDFYSLRDHCLKRGVLFEDETFPADTYSIG-NLSSLSWIRPPHFILEGASRFDIQQGGDCWFLAALGSLTQNPQHLQKIL-TDQSFSYHYAGIFLFRFWQCGQWVEVVIX-------
KEFLFVHPRNKREFWPCLLEKAYAKFHGSYANLHYGYLSDALMDLSGGVVTNIRLHSSSLMMMVKTAAEAGSLITCGLVSRHAYTVTGAEKYRKSWEYLIRLWNPWGRMEWRGPWSDGFWKPLSPSWRRGVTREGSIWGSH---
CRSVQPTPLCD------YFPIQTHTYILVSGL--FHHNY--------CALLLGALIIAVKIFLENFRKGFKKRHEPQPEKYRVTPYTPPPQV----
HMHVYTHFLDATQLQSLLNQEFLECRGIVALMDNGRLNEQEFSRLWRRLVHCQVFWNARKN-SGVFLSSDLQKAIQDTGSVTSELLDLSTNPHSS---------SSALRISKVMYN
>Takifugu_rubripes_ENSTRUG00000000168
QDYAQLRDTCLHTGSLFVDSTFPPNSQSLG-REAQVEWLRPAVFILDGASRFDFSQGGNCWFLAAISALTFHKSVLGQVVPTEQSF-
ENYAGIFHFRFWRFGEWVDVVVDDYLPVLDGTLLSVRSKGGIEFWVALLEKAYAKVCGSYADMNAGLPSEACKDFCGGINMIYELKDAHLWLTLERATTSHAMICCGLVDGHAYTITGVTKCFGCDVKLVRLMNPWGKQEWSGKW
SDSEWNRVSAEDQKKR-EDGEFWMDLEDFCYYFQMLFICGENPSFIRWKYQIYQGSWVAGKSSFDQNPQYRIQVAE-NVLFSLM-
IGLSIFFYYYEREMTEFYSLEPGEYVVVPSTMRAYMNADFVLTVYFKKLNARQLQKLLNENFPSCRSLIALMDKMKMNISEFSALWEKIDKFKLFQSSDTNRNRYLNKYELKKALSAA-
ELSDETVDLLMYRYSDMPLSLNGFIPFMMRMDRMLN-
>Gasterosteus_aculeatus_ENSGACG00000020515
QDYAQLRDNYRRRRTQFVDNTFPPNNQSLG-QEAQVEWLRPPIFCSKGASRFDFDQGGNCWFLAAISSLTFHKGLLAQVVPMDQNF-
SDNAGIFHFRFWRFGVWVDVVIDDHLPTLNNGLMFVSSKDGTEFWAPLLEKAYAKVCGSYADMSSGLPSEACKDFSGGLNMDYKLHEAHLWNALSRATRCQSMICCGIVDAHAYSVTAVTEYYGSKVKLVRLMNPWGRTEWKGKW
SDDMWSKVRPEDRKNF-NDGEFWMELEDFCHYFQNLFICCENPNFI-WKSMTYDGSWVAGITAFEANPQYRLQVTK-NIFLSLM-
IGLTVFFYTNERDLIESYSLEPAEYLIVPSTLKPNMSGDFVLTVYTKRLAAGQLQKLLNDNFPTCMSMMAMVDTMRMTFTEFSTLWEKITKYKRFHLADVNENGSLSERELQKAMEDE-DTDDFMVRLMTARYSGNT-
SMENFITLMLRLEKIMYN
>Oreochromis_niloticus_ENSONIG00000005520
QDFAQIKADCLQKGELFVDNEFPPNGRSLG-Q----DLLRKPAFSKDGMSRFDFGQGGDCWFLSAISALTFQENLMAQVVPMDQSF-
ENYAGIFHFRFWRFGKWVDVVIDDNLPTIKKRLLFVSSKCGNEFWAPLLEKAYAKLCGSYADMHAGFPSEACKDFTGGVNQTYKLKEMNLWLTLNRATRCQSLICCGLVNGHAYSITGVTKLNGSKVRLVRVMNPWGEREWNGKW
SDDLWDRVSPEVKKKC-DDGEFWMQMEDFCSYFAYVSICCETPNFL-WNCMIYDGSWVAGKSAFATNPQYRIRVTV-NIVLSLM-
TRLTIYFYSSERDHIELHSLEPGEYVIIPSTYKPNITADFALAVYTKELLTGQLQKLLNDRFPTCRSMIALVDRMTMSFIEFLILWNKIQEYKLFHQSDLNQSGSLTDLELQKTVEAA-NVNDKTVRLMMFRYSRVT-
MLEDFITLMLRLDKMMYN
>Oreochromis_niloticus_ENSONIG00000005532
QDFAQIKADCLHSGRLFVDNTFPPNSGSLG-QENDVEWLRPPAFCIDGASRFDFGQGGNCWFLSAISALTFKKKLLAQVVPLDQSF-
KNYAGIFHFRFWRFGKWVDVIIDDYLPTINKQLLLAHSKCGNEFWVPLLEKAYAKICGSYADMHWGSPSESFKDFTGGVTMTYKLREAHLWLTLKRAIQCKSLICCGLVDAHAYSVTAVTELYSSKVRLVRLINPWGKQEWNGKW
SDDLWYKVRIEDRKKC-EDGEFWMQLEDVCYYFSYLSICCENPNFI-WKCMTYDGSWVAGRSAFATNPQYRIQVSV-NVLLSLM-
IGLTIYFYSFNSDLTELHSLEPGEYVIIPSTMKPNMTADFVLTVYTNELLAGQLQKLLNDRFPTCRSMIALVDRMTMSFTEFLILWNKIQEYKLFHQSDLNQSGSLTDLELQRAVEAA-NVNDKTVRLMMFRYSRVT-
MLEDFITLMLRLDKMMYN
>Oreochromis_niloticus_ENSONIG00000005524
QDFAQIKADCLRKGGLFVDNAFPPNSRSLG-QESEVKWLRPPAFCTDGTSRFDFGQGGNCWFLSAISALTFQKGLMAQVVPMDQSF-
EDYAGIFHFRFWRFGKWIDVVIDDYLPTINKRLLSVSSKDGNEFWAPLLEKAYAKVCGSYADMHGGSSSEACKDFTGGVNQIYHLTEANLWLTLSRATECKSLICCGLVDGHAYSITEITELNGSKVRLVRIMNPWGKREWSGKW
SDDLWNKVRPDVRKKC-DDGEFWMELEDFFRYFSAVFICCETPNFL-WKCMIYD-----GRVEFATNPQYRIQVSV-NILLSLM-
IGLTIYFYTGFREVVELLSLEPGEYVIIPFTNKPNVTADFVLTVYTKELIPRQLQKLLNDRFPTCRSMIALVDRMTMTFAEFSILWKKIQEYKLFHRCDVNENGSLSSPELQKAMEAA-DVNDGIVGLMMFRYSGST-
SLEEFITLMLRLDKML--
>Xenopus_tropicalis_ENSXETG00000033183
QDYQALLEKSLGGKQLFVDETFPASLASIG-
LPDCVEWKRPPQFCTENASEFGLHQGENCWFLAALSSLTFHPDILTNVVPQNQSFNKSYGGIFHFRFWRFGEWVDVVVDDRLPVNGGKLLFVSSVRKNLFWGPLLEKAYAKLCGSYEDMQIGQVSEALVDFTGGVNMSIKMAQAP
LFQIMMRAQNSGSLMGCGLVTGHAYTVMDVRQVKSVTENLVKLRNPWGKIEWTGKWSDPKWEQLSYKERLFL-EDGEFWMSIEDFEAHFVELVICKLTPDLMEWTLSMQCGRWSAGSTAYWLNPQYRLKILKCNVLVSLM-
IGMSVFY-INSKGLTSPFLLSPRCHLRVPYTCIVHCGSLFLFRTVPREIKVAQLQQLLNKITWDCKLIMALLDSGTLNMQEFRGLWKRLLSYQIFQRRDVDKTGYLKLNDLQAAIKPKGL-
HRQFYNLVALRYGDLKMSFENFACLMLRIEINLYS
```

>Gallus_gallus_ENSGALG00000009069
QNYQALLEMCLKNKQLFTDESFPADISSIG-
LPRNVQWKRPPVFYAANRKQLDLCQGGNCWFLAALQALTFHQDILAAVVPQNQSFERKYAGIFHFRFWHFGEWVDVVVDDRLPVNEGELVFVSSVYKNVFWGALLEKAYAKLYGSYEDLQIGQVSEALVDFTGGVNIRIKLPAAP
LWDILTRATYSRSLMCCGLVAGHAYTVTGIRKCQYGPENLVRLRNPWGKIEWNGDWSDYKWELLSPKEKILL-EDGEFWMSLQDFKIHFVDVVICKLTPDLVKWMYSLKNGRWVKGSTAFWMNPQYWLNILACSVVISLM-
IGFSLYFFLDEREVTCDFHLEPGVYVIVPSTLEPHQESEFILRVFSREINAVQLQRILNNVSWACQSILALLDSGTLSIQEFRVLWKRLLFYLVFQKRDTSRSGKLDLVELRAAVQETGL-
SNEVCNLMAIRYGDLKISFESFMCFMLRVEIMLYS
>Meleagris_gallopavo_ENSMGAG00000005468
QNYQALLEMCLRNKQLFSDESFPADISSIG-
LPRNVQWKRPPVFYAANRKQLDLCQGGNCWFLAALQALTFHQDILAAVVPQNQSFERKYAGIFHFRFWHFGEWVDVVVDDRLPVNEGELVFVSSVYKNVFWGALLEKAYAKLYGSYEDLQIGQVSEALVDFTGGVNIRIKLPAAP
LWDILTRATYSRSLMCCGLVAGHAYTVTGIRKCQYGPENLVRLRNPWGKIEWNGDWSDYKWELLSPKEKILL-EDGEFWMSLQDFKIHFVDVVICKLTPDLVKWMFSLKNGRWVKGSTAFWMNPQYWLNVLACNVVISLM-
IGFSLYFFLDEREVTCDFHLEPGVYVIVPSTLEPQQESEFILRVFSREINAVQLQRILNNVSWACQNILALLDTGTLSIQEFRVLWKRLLFYLVFQKRDTNRSGKLDLVELRAAVQETGL-
SNEVCNLMAIRYGDLKISFESFMCFMLRVEIMYF-
>Taeniopygia_guttata_ENSTGUG00000004597
QNYQALLESCLKNKCLFTDDTFPAHISSIG-
LPRNLQWKRPPVFYAANRKQLDLCQGENCWFLAALGALTFHQDILAAVVPQNQSFERKYAGIFHFRFWHFGEWIDVVVDDRLPVNEGELLFVSSVYKNVFWGALLEKAYAKLYGSYEDLQIGQVSEALVDFTGGVNTRIKLAEAP
LWDILTRATYSTSLMGCGLVAGHAYTVTGIRKCQYGPENLLRLRNPWGKIEWKGDWSDFRHELMLRLAKIML----TFWMSLQDFKIHFVDLVICKLTPDLMKWMYSLKSGRWVKGSTAFWMNPQYWLNVLPCSVVISLM-
IGFLLYFFLDEREVTYDFHLEPCVYVIVPSTLKPQQESEFILRVFSR--------------------------------------------------------------------------------------------------
-----------
>Pelodiscus_sinensis_ENSPSIG00000017963
QNYETLLETCLKNKCLFKDENFPADLSSIG-
LPPKLQWKRPPVFYPANTRQLDLCQGENCWFLAALQALTFHRDILTVIVPQYQSFDRKYAGIFHFRFWHFGEWVDVVVDDRLPVNEGQLIFLSSVCKNIFWGALLEKAYAKLYGSYEDLQIGHVSEALVDFTGGVNMTIKLAEAP
LWNILTRAAYSRSLMGCGLVAGHAYTITGIRKCKYGPEYLVRLRNPWGKIEWKGAWSHGNWELLSLKEKILL-DDGEFWMSLQDFKTHFEDLIICKLSPDLMKWMYSLQTGSWVKGSTAFWMNPQYWLDVYQCSMLVSLI-
IGLSIFFFMSKREVTQDFRLQPGSYVIVPSTAEPQQESEFVLRVFSREINAVQLQRILNNMVWACQSILAVLDTGTLSIQEFRLLWKRLLFYMVFLKKDVARSGNLNLMELHAAVQETGLNSNQVCNLMVIKYGKMKISFESFFC
FMLRVEIM---
>Anolis_carolinensis_ENSACAG00000007149
QNYQTLLEDCRKKGYLFEDELFPADLSSIG-
LPAKIQWKRPPVFFSSKKISCMLCQGDDCWFLAAVEALTFHQDILFKVVPQNQSFKKKYAGIFHFKFWHFGEWVDVVVDDRLPVNEGQFIFLSCIYKNIFWGALLEKAYAKLCGSYGDLQIGYVSEALVDFTGGVNITIKLAEAP
LWNILTRAIYSRSFIGCGLATCHAYTMTAIQKCKHGTENLVRLRNPWGKTEWKGDWSDEKWELLNPKEKILL-EDGEFWMSLQDFQIHFVDLIICKLTPDLMKWMCSMQFGKWVKGWTSFWMNPQYLLKVLRHSVLISLI-
IGLTVYFFFNQREVTQDFRLPPGIYVIVPSTAEPHQESVFLLRVFSRNEGCRAEHRHVN----VCRSILALLDTGDLSIQEFRLLWKKLLLYQVFQKKDIHHTGCLEQAEVQAALEEMGI-
SSELCKMMAYRYGDLKVTFENFACFVLRVELMLYS
>Sus_scrofa_ENSSSCG00000024781
QDYEALQERCLIDGCLFEDQSFPATLRSIG-
LPERVYWRRPPQFYSAKAKRLDLCQGGDCWFLAALQALTLHQDILSRVVPLNQSFTEKYAGIFRFWFWHFGKWIPVVVDDRLPVNEGQLVFVSSTYKNLFWGALLEKAYAKLSGSYEDLQRGQVSEALVDFTGGVTMTINLVEAP
LWDILTRAIYSRTLIGCGLVDGHAYTLTGIRKSKHGPEYLVKLRNPWGKVEWKGDWSDSTWELLSPKERILL-NDGEFWMTLRDFKTHFTLLVICKLSPGLLTWLYTMLEGRWEKGTTAFWKNPQFLLSVWRCSVLVSLL-
IGFYLFFFLTEKEVSRELWLEPGTYLIVPCTSEPRQESEFVLRVFSREINAIQLQKILNRMPWACQGILALLDSGTVSIQEFRHLWKQLMFYQVFHKQDTHRSGSLNWAQLQAAMREA-RGERSSC--
WLPRGWGLEIKCFQFTHTHTHTHTHLYS
>Sus_scrofa_ENSSSCG00000027391
QDYEALQERCLIDGCLFEDQSFPATLRSIG-
ILEGQFRLFPPQFYSAKAKRLDLCQGGDCWFLAALQALTLHQDILSRVVPLNQSFTEKYAGIFRFWFWHFGKWIPVVVDDRLPVNEGQLVFVSSTYKNLFWGALLEKAYAKLSGSYEDLQRGQVSEALVDFTGGVTMTINLVEAP
LWDILTRAIYSRTLIGCGLVDGHAYTLTGIRKSKHGPEYLVKLRNPWGKVEWKGDWSDSTWELLSPKERILL-NDGEFWMTLRDFKTHFTLLVICKLSPGLLTWLYTMLEGRWEKGTTAFWKNPQFLLSVWRCSVLVSLL-
IGFYLFL-LTSFSVYKNLCLAPSWYLGLVLFFLLREHWAFLLINLKREINAIQLQKILNRMPW----------------------------------------------------------------------------------
-----------
>Homo_sapiens_NM_001145122
QDFEALLAECLRNGCLFEDTSFPATLSSIG-
LPPRLQWKRPPQFYFAKAKRLDLCQGGDCWFLAALQALALHQDILSRVVPLNQSFTEKYAGIFRFWFWHYGNWVPVVIDDRLPVNEGQLVFVSSTYKNLFWGALLEKAYAKLSGSYEDLQSGQVSEALVDFTGGVTMTINLAEAH
LWDILIEATYNRTLIGCGLVEGHAYTLTGIRKCKHRPEYLVKLRNPWGKVEWKGDWSDSKWELLSPKEKILL-NDGEFWMTLQDFKTHFVLLVICKLTPGLLKWTYTMREGRWEKRSTAFWKNPQFLLSVWRCSVLVSLL-
IGFYLYFFLKEKEVSQELCLEPGTYLIVPCILEAHQKSEFVLRVFSREINAVQLQNLLNQMTWACQGILALLDSGTMSIQEFRDLWKQLKLSQVFHKQD-RGSGYLNWEQLHAAMREA-
MLSDDVCQLMLIRYGGLQMDFVSFIHLMLRVENMLYS
>Sarcophilus_harrisii_ENSSHAG00000012346

QDYETLLETCLRNNCLFEDPNFPADMNSIG-
LPPTLHWKRPPVFYSTKAERLGLCQGGNCWFLAALEALTFHGDILNRVVPLNQSFTKRYAGIFLFRFWHFGEWIPVVIDDRLPVNEGHLVFVSSTCKNLFWAALLEKAYAKLSGSYEDLQIGHVSEAFVDFTGGVTATIQLSKAP
LWDILTRVASKRSLIGCGLVDGHAYTVTGIRKCHNRPEYLVRLRNPWGKVEWKGDWSDKKWDLLNPKEKILL-EDGEFWMSFHDFKFHFVSLVICQLTPDLMKWMCSLSTGRWVKGSTAFWMNPQFQLTVLSCSVLVSLM-
IGIFLFFFQTEREVSYEFSLEPDTYLIVPCTSEPWQESEFILRVFSREINAIQLQKILNNMTWACLGILALLDTGTVGIQEFRYLWKRLLIYQVFHKQDKHKSGFLESGQLRAAAQEAELLSDEVCNLMAIRYGNRKIGFENFVY
FMLRAENVLYC
>Monodelphis_domestica_ENSMODG00000015513
QDYQTLLEMCLRNNCLFEDDTFPADMSSIG-
LPPNLQWKRPPVFYSAMAERLCLCQGGNCWFLAALEALTFQWDILSWVVPVNQSFTRKYAGIFFFQFWHFGEWVPVVIDDRLPVNEGQLVFVSSTCKNLFWGALLEKAYAKLSGSYEDLQIGHISEAFVDFTGGVTATIELANAP
LWELLTRATCKRSLIGCGLVDGHAYTLTGLRKCHNVPEYLVRLRNPWGKVEWKGDWSDKKWDLLNPKEKILL-EDGEFWMSLHDFKFHFVSLVICQLTPSLMKWICSLSTGRWVKGSTAFWMNPQFQLTVLRCRVLVSLM-
IGIFLF------------------------------------------------------------CVGV--------------------------------------------------------------------------------
-----------
>Lamprey_ENSPMAG00000009490
KTFQEIKKECLQRKILFEDPDFPASLSSLHTSNNFIEWKRPPEFIVSGATRADINQGPDCWLLSAIASLTLYPDLLAKVVPMGQSFHEDYAGIFLFRFCQYGEWVDVHVDDRLPTKKGELVFVQSGTGNEFWSALLEKAYAKLNG
CYEALAYGFPVEALCDFTGGVGETFTMNNAPYWDGISRAIKKGAIICCGLVEGHAYSITGVIKFPGKTVQLIRVRNPYGKVEYTGPWSDTTWNEVPEEDQKRL-
EDGEFWISDKDFRKNFHLVEICHQGSEVELWICELHNSMWLKGLNAFPLNYQFFLTLLECHFLLSLTPIGIYIYQFIARREVTMRMTLPPGNYIIIPCTESAGQEAQFLLRIFMELIKADKLQALITLEIQSCRVLVALFDSGQL
GYEEFLLVWEFIKMTLTYKMCDVDKSGTLSSTELPMALEHSRKVGKLMQRLIMMRFSELTISFDSFVCLLAKMYTAFNI
>Takifugu_rubripes_ENSTRUG00000010051
QDFKKLLEACVKSGELFDDPAFPAEQKSIGPPKNAIKWLRPAVFVEGTTGTTDICQGGNCWLLAALSCLTMHPTLFVKVVPPGQSLSQSYAGIFYFRFWQYGEWVEVVVDDRLPVRGGRLLFSYSHTCNEYWSALVEKAYAKLIG
CYGSLKGGNISEGMEDFTGGIAYSTRVSRTPLWRSLTAALSRGSLLSSGLIKGHAYAITDTVKNASVETLLLRLRNPWGFVEYRGPWSDKEWDDVDKAEKEKIIEDGEFWISVEDLSRLFDVVELCSVNPDTLTWTISEHEGFWV
PGSSAFWKNPQFQLVLRECTVLVELLYIAFHVYFYRAQRGVWRKLLLDPGRYIVVASTYRPNQSGEFFVRIFSKRVNAKELMGLFNSAL-
TCRQLIFGEDRASLSRKQAETLLTSLRQLQIFFQFDEDSSGTMSPFELSAALEAV-QCDRKIVELLSERFASLHAPFHSFVSCVTRLRRLL--
>Oreochromis_niloticus_ENSONIG00000003718
QDFETLQAECLKSGVLFSDPTFPAEQKSIGPPKKAIQWKRPAAFVEDTTATTDICQGGDCWLLAALSCLTAHPSLFVKVVPPNQSLSDRYAGIFHFKFWQYGEWVEVVVDDRLPVREGRLLFSYSRSRNEFWSALVEKAYAKLIG
SYGSLKGGNISEGMEDFTGGIARSLEISRTPLWRSLAAALSRGSLLSCGLIRGHAYAITDADKKTSDEILLLRLRNPWGFIEYCGPWSDKEWENVDPTEKERIKEDGEFWISAEDFCNLFDVVELCSVNPDSYSWAITEHEGCWL
PGSSAFCKNPQYTLVLRECTVLVELLYIAFHVYFYKAQRGVWRKMRLDPGTYVIVVSTYRPNLPGEFFVRTFSKRLNVKDIMKLFNSVLETCRELIFGEDRCALSREQAGTLLASVRNL-
IFSQFDEDSSGSMSRFELSSALHFP-QCDNKVVELLSERFTSLDMPFHGFVSCVTRLRKL---
>Xiphophorus_maculatus_ENSXMAG00000018623
QDFDALLQECLKDKKLFADPTFPAEQKSIGPPAKEIKWKRPALFVEDTIGTTDICQGGDCWLLAALSALTVHSKLFAKVVPPNQSLSDSYAGIFHFRFWQYGEWVEVVVDDRLPVREGRLLFSYSRTRNEFWSALVEKAYAKLVG
SYGSLKGGNISEGMEDFTGGIAYSLPVARTPLWRILTASLSRGSLLSCGLVKGHAYAITDTNKKGPEEVLLLKLRNPWGFVEFCGSWSDKEWADTEQSEKQRIKEDGEFWISASDFSAMFNVVELCSVSPESLTWTISEHKGSWV
SGSSAFWKNPQFELVLRECTVLVELLYIAFHVYFYKAQRGIWRKLRLDPGHYVIVGSTYRPNQPGEFFIRIFSKRLNAQEIMELINSVLSTCRQIIFGEDRATLSREQAEIVLADLLTLQVFVQFDEDSSGSISPFELSLALEAV
-TCDGKVVQLLSERFVGPHLSFHGFVSCVSRLRKLLA-
>Danio_rerio_ENSDARG00000010758
QDYEVLLQTCLKSAVLFSDSVFAANQSSLGPPKKAVKWLRPAVFVEGTMGTTDICQGGNCWLLAALSCLTMHPTLFVKVVPAGQSLSESYAGIFRFKFWQYGEWVEVVVDDRLPVREGRLLFSYSRTSNEFWSALVEKAYAKLIG
SYGSLKGGIISEGMEDFTGGIAYSLPVSRPPLWKAITAALARNSLLSCGLIKGHAYAITETGKKASEEVFLLRLRNPWGFVEYCGPWSDKDWDFVDNTEKTKLAEDGEFWISIEDFCRFYNTVEMCSVDPDSMSWTLSLHQGTWV
PMCSAFWKNPQYQMILSECTVLVELLYIAFHIYFYRSIRSVWRKVHLEPGSYVILASTYRPNQQGEFFLRIYTKRVNAVQFMNLVNSVLETCRQLIFGEDRGRLDRAQAEKLLSSLRNLQIFFQFDEDSSGTMSPFELSLALNAA
-ECDSVVVQMLWERFGAQYLPFYGFVSCVARLQVLLAV
>Gasterosteus_aculeatus_ENSGACG00000009801
QDFQTLLEKCLKSRTLFADPCFPPQQTSIGPPKKAIKWRRPAVFVEDTIGTTDICQGGNCWLLAALSCLTMHPTLFEKVVPPNQSLSESYAGIFHFRFWQYGEWVEVVVDDRLPVREGRLLFSYSHTRNEYWSALVEKAYAKLVG
CYGSLKGGNISEGMEDFTGGIAYSLQVSRAPLWRSLTAALSRGSLLSCGLVKGHAYAITDSDKKASDETLLLRLRNPWGFVEYRGPWSDKEWDDVVKEEKERIKEDGEFWISAEDFSRLFNVVELCSVNPDNLTWSISEYKGCWV
SGSSAFWKNPQFQLDLTQCTVFVELLHIAFHIYFYEAQRGSWREMQLDPGNYILVPSTFQPNQPGEFFVRIFSKRLNPKELMKLFNTGLLATR-------------------
WEQGDNEMIFLQFDEDSSGTMSPFELSAALQAVGQCDGKVLELLCERFASLHMPFHSFVSCVTRMQKLVM-
>Lepisosteus_oculatus_GENSCAN00000002892
QEFASLRAQCLRSGSLFEDPEFPAQQDSLGPPAKAVVWRRPPKFIEGTASTTDICQGGDCWLLAALSCLTLHQPLFNNVVPGDQSLSEEYAGIFHFKFWQYGQWVEVVVDDRLPVQRGRLLFSYSRTQNEFWSALLEKAYAKVNG
CYASLKGGNISEAMEDFTGGIARSLPVKRTPLWRAVGESLSRGTLLSC---------------RCALRCSLCRL-----------
PVLPEDWDRVDNAEKQRIIEDGEFWIKADDLSHLFTTVEMCSVNPDALLWRITAHEGAWVPGCSAFCKNPQFRLILSQGSVVLELLYIAFHVYFYRQLRGVRKKVKLEPGHYVIVTSSYKPNVEGSFFLRIYAK-----------
------------------------------------IFSQFDEDSSGTMSPFELSLALQAA-QLDAPVLQLLWLRHGTLSLTFDGLVACVGKLRKL---
>Xenopus_tropicalis_ENSXETG00000034127
--------------------------------------
WRRPPRFILDGATRMDVCQGSNCWFLSAVACLSLYPQLLEKVVLPGQDFVGAYDGKFRFQFPGQGAAVDGDIHGCLRTGDGQXIRTKPAHKPEVWQSFAEMVYSGLKGGYSALQLGFAGEALVDMTGGVAQTCYTNGPLLWVSLS

```
HLLQQGALTCCGILSHHMYSVTGAKQALHGSVSLLRVRNPWGHTEWSGPWRDSEWLSVINIKEIEV-EDGEFWMDVEDFQKNFQVLEICHLGPESLPWEYVTYEGCWLKDISSFWMNPQYTLSLVTCSIIVSVMYI-
FQLYICDKKREAVLSTSLPPGRYIIIPSLETTSDEGEFLIRILTEKWNRNSKKNFLNDNISCCR-
LIASLDEGALCWEQFDRLWKNIITGSIFSNLQSSKDRNLDKDQIGAALQSAGITDNFLVRLVQLRYADGSLSYSAFICCLLKIKAVLYS
>Gadus_morhua_ENSGMOG00000007927
--------------GLFIDYTFPVGQELLA----
SVEWKRPPQFIVDGATRMDVCQGGDCWFLSALASLSMHRSLLKRVVPPGQGFQNGYDGSFYFRFWQYGKWEEVRVDDLLPTQNGQLYYLRSPNRNEFWSCLLEKAYAKLKGGYQALEMGFPHEAMXDMTGGVTEVLSVASFSLPR
ALQHLLRKGALINCGILFRHAYSLTAVERTA-GPVTLVRLHNPWGGTEWKGRWSDPEWNTVSEDEQRRLVEDGEFWMSVSDFRRNLEIIELCHMS-DSLPWHCQMHQGHWVPNLSAVLPDP------
VRCSFLVALMSIGMNVYDHVMRRETVLRGALAPGHYVIIPYASLPNQEGRFILRVLTEVCKPLNLHRLLTEAIQHCKSMVVLVDLAVLDWPGFKTLWYKIQSWTAFLIHDKNGSKKLEYVEVSSAMKSAGNMDSSVMQFVGLRYT
EMTVSYPGFLYLMMKLDSM---
>Takifugu_rubripes_ENSTRUG00000009564
MSLERAEGPPVGWDGLFVDRHFPLGELEMQ----
EVKWKRPPNFIVSGATRLDIRQGNDCWLLSAIASLSLHPSLLERVVPLQQSFQDGYNGSFTFRFWMYGQWEEVRIDDLLPTLDNRLIYLSSPDKCEFWSPLLEKAYAKLKGGYRALNMGFPHEAMVDMTGGVTEVLNIAALPLPP
LLSYLLSKGALINCGIMFRHAYSLTAVEQTAHGTECLVRLLNPWGNTEWEGAWSDPEWNSVSIQEQQRLVEDGEFWMSVSDFRQNFEIMEVCHQT-
EAFPWSCSMHHGSWVSSITAFWQNPQFSFTLSECSFVLALMSIALHVYDYSSRREVVLRSSLPPGRYLIIPSTSEPNQQGEFLLRVLTEFCKPVHLYNLLTEAIQQCKSMVVLVDIARLNWIEFQDLWDKIRKWTIFLVFDKNKT
KRLEYQEVGPALKAAGVVDDLIMQLVGLRYTEMTISYPGFLYLLLKLENM---
>Oryzias_latipes_ENSORLG00000000963
LAAPAADGVTLGRDGLFVDYQFPVGELEMQ----
GLKWKRPPQFIIDGASRLDIVQGSDCWLLAAIASLAMHRSLLKKVVPLHQSFEEDYNGSFVFRFWQYGQWEEVRIDDLLPTENDKLVYLSSPERREFWSSLLEKAYAKLKGGYRALDMGFPHEAMVDMTGGVTEVFSIASLPLLE
FLDYLLSKGALINCGILFRHAYALTAVEKTTNGPVDLVRILNPWGRTEWKGPWSDPEWQTVSLEEQRRLVEDGEFWMSISDFRQNFEVMEVCHLT-ECLPWSCVMHHGNWVP-------
NPQFLLVLSECSFVLALMSIGMHVYEHSSRREVVLRGSLPPGRYIIVPSTLEPNQEGAFLLRVLTEFCKPLHLLNLLTEAIQHCKSLVVLMDVREERSVTGGLLWSLVDSWTIFLMFDKNKTKHLEYQEVKPALMEAGKVDDLVM
QLVGLRYTEMTVSFPGFLYLMMKMESMMYN
>Gasterosteus_aculeatus_ENSGACG00000013147
--------------GLFVDHHFPVGELEMQ----
GVKWKRPPQFIVNGATRLDVCQGNDCWLLSAIASLSVNRFLLNKVMPLEQSFKDGYKGCFTFSFWQYGQWEEVKIDDFLPTKDNKLIYLSSPERDEFWSALLEKAYAKLKGGYRALDMGFPHEAMVDMTGGVTEVFNISLLPLPA
FLRHLLARGALINCGIMFRHAYSLTAVEQTAHGIVDLVRILNPWGNTEWSGPWSDPEWEAVNKEEQKRLVEDGEFWMSVSDFRRHFETMEVCHLT-
DSLPWCCVMHHGNWVPSISAAQGEPSVLLHLVRCSFVLALMAIALHIYDYSMRREVVLRGSLPPGRYIIVPSTAEPNQPGAFLLRVLTELCKPVHLLNLLTEALQHCKSLVVLTDNARLNWLDFRCLWDKIRRWTIFLVFDKNKT
NHLEYKEVAPALQKAGMVDDLIMQLVGLRYTELTISYPGFLYLLMKLESMMYN
>Oreochromis_niloticus_ENSONIG00000001284
MPPESGDVTPVGRDGLFVDFHFPVGELEMQ----
GVKWKRPPQFIVDGATRLDVHQGSDCWLLSAIASLSMYQSLLKKVVPLSQSFQDGYNGCFTFRFWQYGQWEDVQIDDLLPTVDNNLIFLSSPEKQEFWSALLEKAYAKLKGGYRALDMGFPHEAMVDMTGGVTEVLQVASLPLPT
FLRHLLVRGALINCGIMFRHAYALTAVEKTTHGTEYLVRILNPWGNTEWEGPWSDSEWNTVSVEEQKRLVEDGEFWMSVSDFRQNFEVMEVCHLT-
EALLWSCIMHHGNWVPNITAFYQNPQFYFVLSKCSFVLALMAIALHVYDYVTRREIVLRGSLPPGSYIIIPSTAETNQQGAFLLRVLMELCKPLHLYNLLTEAIQHCKSLVVLMDIAQLNWPEFQSLWDKIRRWTIFLMHDKNKT
QRLEYQEVMPSLKAAGRVDELVMQLVGLRYTEMTISYPGFLYLLMKLESMMYN
>Danio_rerio_ENSDARG00000055094
RTGLGAPAPPVPATGLYVDTRFPVQNMPVQ----
DLVWRRPPQFIVDGATRMDICQGGDCWFLSAMASLSLYPALLDRVVPAGQGFQQGYNGCFCFQFWQYGEWISVKIDDLLPTCNNQLIYLHSASRDEFWSALLEKAYAKLKGGYSALNMGFPHEAMVDMTGGITEVFMVASLPLGS
FLRSLLEKGALINCGILFRHAYSVTALETSTIGPVQLVRIRNPWGKAEWEGPWSDIEWHMVTAEEQRRVIEDGEFWMSLADFRQNFELMEVCHLSEDTLPWKCTSHHGQW----TP----PQYNLTLLECSFLLALMSIALHIY-
FQQRPELVLRGRLAPGHYIIIPAIQETNQSGEFLLRVLTEYCRPLDLYNLLTEAISHCKSFVVLMDMGRLDLTEFQALWDKLRKWTIFITFDKNKNQALDYLEISPALSAAGKVDEFILQLISLRYTEMTLSFPGFLFLLMKLDC
MLYN
>Lepisosteus_oculatus_GENSCAN00000011023
RT--------GSEVALFVDKSFPVHYNP------
KLVWKRPPKFIVDGATRTDVCQGSDCWFLSAVASLSQHRALLERVVPEGQGFDREYTGCFRFQFWQYGEWKEVEVDDFLPTEGGQLVYLHSAERDEFWSALLEKAYAKLKGGYQALQLGFPHEALVDMTGGVTEIFTIPLLPLAG
FLRPLLEKGALINCGILFKHAYSVTGLEKCYLGEADLVRVRNPWGHTEWTGPWSDLEWAKVSAEEQARVVIDGEFWMEVSDFQRNFNMMEVCHLSDSTLPWECATHHGSWVPGISAYWLNPQFRMTLLECSFLVALMDIGLHIYD
YNQQREVVIRSALAPGHYIIIPSTSMANQEGEFVLRVYTERCGAVELQGLLKEVIVQIVSFTLVHDRILLEMEEFEALWEKFRKWTIFVHFDKNRSQSLDYPEIIPALQAAGQVDDFVLQLIGLRYTDLTISYPGFLHLLLKLDI
M---
>Xiphophorus_maculatus_ENSXMAG00000003111
------------------------------------
TSRRLPKFIVDGASRLDICQGSDCWVLSAIASLANYPELLKKVVPLNQSFQDGYNGRFSFRFWQYGQWEEVRIDDFLPTYNNKLIYLSSPERHEFWSSLLEKAYAKLKGGYRALELGYPHEAMVDMTGGVTEVFSLPLVSLPELL
RNLLAKGALINCGIMYRHAYSLTAVEKTTDGFKDLVRILNPWGHTEWLGAWSDNEWTKVSAEEQKRLVEDGEFCLTVIDTRSKIETLFACLTSDRGLRRTVRTYVTRSGNGGTVYERNTRRRRYVRPCLFYLPASACSDE--
```

DHSTRREVVLRSSLPPGRYIIIPSTFEPNQQGEFLLRVLTELIFPAALASSRQEAFPHCKSLVVLMDKARLNWSDFQLLWDKIRKWTIFLVYDTNKTHHLEYKEVEPALKAAGMVNDLVMQLVGLRYTELTISYPGFLFLLMKME
SMLYS

(*c*). The 22 amino acid sequence alignment used for the sub-phylogenetic analysis (i.e. CAPN12/17 analysis).

>Anolis_carolinensis_ENSACAG00000011727
LFLDPEFKCAESLGYKELGPGQGVVWKRPKDICKTPHFICKGMNRTDVCQGQLGDCWFLAAAASLTLYPELLYRVVPQDQHFGTEYAGIFHFQFWQYGQWVDVVVDDLLPTINNELLFVRSPEHDEFWMPLLEKAYAKLNGSYEA
MNGGYMNEAFVDFTGGIGETLSLKILFKTIRAALSRNSLMGAHIEVKRPEKPTPEGLVKGHAYSITGIHKIDEKVVRLLRLRNPWGYQEWTGRWSD-
LWSSLDPDRKDGEFWMQLVDFIRHFDVLEICHFSHTAGFCTFLVSLMQRERRRSRRSPTHATASNGYSRDITRYLQLPPGDYLIIPNTQSPLEEANFTLRIFTEKKHQFDGRLEDFQQFSCRMQEWQHIFLAYDVDRSGSMNTHE
IQLALDAA-GFHLNNRTTEALVKKYGNPWLQIDFDSFVSLMVHLESVFIHILFGLLVS
>Xenopus_tropicalis_ENSXETG00000002784
LFEDPHFPDAKSLGYNELGPWRGIQWRRPQEICQSPKFITENMKWTDVCQGQLGNCWFLAAAASLTQYPLLMARVVPPQQGFKDRYVGIFHFQFWQYGEWVDVVVDDRLPVKNGQLVFVSSAQKSEFWAALLEKAYAKLNGSYEA
LNGGFMNEAFVDFTGGLDETVDLKVLYHLIEKAVKKRSLMGASIQIQSQELTTPEGLVKGHAYSIIATWKMEGRTIHLLRLRNPWGKVEWNGRWSD-LWSQVTFG-
EDGEFWMQMEDFLRFFDILEVCNLTSNAGYCTLLVSLMQKNRRQYRSRPNQKSLSFVAKRDVTERYQLPPGRYLLIPSTFQPHQESDFILRVFTEKQHTLDYLLEEFRQLWFKIKEWEKIFTKYDKDRSGTMDVQELRLALEAA-
GFTLNNQLVESLCQKYGDDVRQVDFDSFLSCLAYLVCVFGQCQDSLHFN
>Homo_sapiens_CAPN12_AK127398
LFRDPYFPGPDALGYDQLGPDKGVKWMRPHEFCAEPKFICEDMSRTDVCQGSLGNCWFLAAAASLTLYPRLLRRVVPPGQDFQHGYAGVFHFQLWQFGRWMDVVVDDRLPVREGKLMFVRSEQRNEFWAPLLEKAYAKLHGSYEV
MRGGHMNEAFVDFTGGVGEVLYLRQLFSALRHALAKESLVGATALSDRGEYRTEEGLVKGHAYSITGTHKVFFTKVRLLRLRNPWGCVEWTGAWSD-RWDTLPTK-
EDGEFWMELRDFLLHFDTVQICSLSVNSGNCTVLLSLIQRNRRRLRAKPRLLRARLSARRDVTRRCCLRPGHYLVVPSTAHAGDEADFTLRVFSERRHTADDLLHHFQQLWGYLLEWQAIFNKFDEDTSGTMNSYELRLALNAA-
GFHLNNQLTQTLTSRYRDSRLRVDFERFVSCVAHLTCIFCHCS-TATFS
>Sus_scrofa_ENSSSCG00000002967
LFRDPYFPGPDALGYDQLGPDKGVEWKRPHEFCAEPQFICEDMSRTDVCQGSLGNCWFLAAAASLTLYPRLLCRVVPPGQGFQDGYAGVFHFQLWQFGHWVDVVVDDRLPVREGKLVFVHSAQRNEFWAPLLEKAYAKLHGSYEV
MRGGHMNEAFVDFTGGVGEVLYLRQLFAALRHALAKESLVGATALSDRGEYRTGDGLVKGHAYSVTGTHKVSFTKLRLLRLRNPWGRVEWTGAWSD-RWDELPTK-
EDGEFWMELQDFLCHFDTVQICSLSVSSGGCTVLLSLIQRNRRRLRAQPSLLRARFCARRDVSRRCRLRPGHYLVVPSTARAGDEADFTLRVFSERRHTADDLLYHFQQLWGHLLEWQATFDKFDEDASGTMNSYELRLALNAA-
GFHLNNQLTQALTSRYRDSRLRVDFERFVSCMAQLLCLFRHCS-TATFS
>Mus_musculus_ENSMUSG00000054083
LFRDPCFPGPDALGYDKLGPDKGVEWKRPHEFCAEPQFICEDMSRTDVCQGSLGNCWLLAAAASLTLYPRLLYRVVPPGQGFQDGYAGVFHFQLWQFGRWVDVVVDDKLPVREGKLMFVRSEQRNEFWAPLLEKAYAKLHGSYEV
MRGGHMNEAFVDFTGGVGEVLYLRQVFAALRHALAKESLVGATALSDRGEIRTDEGLVKGHAYSVTGTHKMSFTKVRLLRLRNPWGRVEWSGPWSD-RWDMLPSK-
EDGEFWMELQDFLTHFNTVQICSLSVNSGSCTVLLSLIQRNRRCLRAKPGLLRARFCARRDVSRRCRLPPGHYLVVPSASRVGDEADFTLRIFSERSHTADDLLHHFQELWGHLMSWQATFDKFDEDASGTMNSCELRLALTAA-
GFHLNNQLTQSLTSRYRDSRLRVDFERFVCCAARLTCIFRHCC-TATFS
>Monodelphis_domestica_ENSMODG00000013388
LFLDPCFPGPSALGYDQLGPGKGVEWLRPHEFCENPQFICENMDRTDVCQGSLGNCWFLAAAASLTLYPRLLHRVVPPGQSFQWGYAGVFHFQLWQFGHWVDVVVDDRLPVRDGKLLFVRSAQRAEFWASLLEKAYAKLHGSYEV
MRGGHMNEAFVDFTGGVGEVLYLQPLFSALRHALAKESLVGATALSDRGEYRTEDGLVRGHAYSVTGTHKITFAKVRLLRLRNPWGHVEWSGAWSD-RWAALPEK-
EDGEFWMGLEDFLQHFNTVQICSLSVNAGGCTVLLSLIQRNRRRLRARAGLLRARFSIRRDVSRRCRLAPGHYLIVPSTAHVGEESDFTFRIYTECQHTADNFLPEFQQLWYRLQGWQATFNKFDADKSGTMNSHELRLALNTA-
GFQLNNQLTQLLTSRYRDSRLRVDLDRFVSCVTQLTCLFRHCS-TATFS
>Takifugu_rubripes_ENSTRUG00000010051
LFDDPAFPEQKSIG---
MPEDNAIKWLRPKEIGKNAVFVEGTTGTTDICQGQLGNCWLLAALSCLTMHPTLFVKVVPPGQSLSQSYAGIFYFRFWQYGEWVEVVVDDRLPVRGGRLLFSYSHTCNEYWSALVEKAYAKLIGCYGSLKGGNISEGMEDFTGGI
AYSTRVSSLWRSLTAALSRGSLLSSLIEAGSYGKVTGEGLIKGHAYAITDTVKVLSVETLLLRLRNPWGFVEYRGPWSD-EWDDVDKKKEDGEFWISVEDLSRLFDVVELCSVNTSAGYCTVLVELLQKNRRQ--
RDKNSPVGSYRAQRGVWRKLLLDPGRYIVVASTYRPNQSGEFFVRIFSKTGNTLDFLRKQAETLLTSLRQLQAIFFQFDEDSSGTMSPFELSAALEAV-
GMQCDRKIVELLSERFASGALHAPFHSFVSCVTRLRRLFVIFSHLIL--
>Oreochromis_niloticus_ENSONIG00000003718
LFSDPTFPEQKSIG---
TPEDKAIQWKRPKEISKNAAFVEDTTATTDICQGQLGDCWLLAALSCLTAHPSLFVKVVPPNQSLSDRYAGIFHFKFWQYGEWVEVVVDDRLPVREGRLLFSYSRSRNEFWSALVEKAYAKLIGSYGSLKGGNISEGMEDFTGGI
ARSLEISSLWRSLAAALSRGSLLSCFIQAKSTGKVTGEGLIRGHAYAITDADKLTSDEILLLRLRNPWGFIEYCGPWSD-EWENVDPCVEDGEFWISAEDFCNLFDVVELCSVNSSAGYCTVLVELLQKNRRQ--

RDKNRPAGSYKAQRGVWRKMRLDPGTYVIVVSTYRPNLPGEFFVRTFSKIGNTL--LREQAGTLLASVRNL-SIFSQFDEDSSGSMSRFELSSALHFP-EFQCDNKVVELLSERFTSGELDMPFHGFVSCVTRLRKLF-------
---
>Xiphophorus_maculatus_ENSXMAG00000018623
LFADPTFPEQKSIG---
MPKDKEIKWKRPKEISADALFVEDTIGTTDICQGQLGDCWLLAALSALTVHSKLFAKVVPPNQSLSDSYAGIFHFRFWQYGEWVEVVVDDRLPVREGRLLFSYSRTRNEFWSALVEKAYAKLVGSYGSLKGGNISEGMEDFTGGI
AYSLPVASLWRILTASLSRGSLLSCFIQASSYGKVTADGLVKGHAYAITDTNKVAPEEVLLLKLRNPWGFVEFCGSWSD-EWADTEQLEEDGEFWISASDFSAMFNVVELCSVSSSAGYCTVLVELLQRNRRQ--
NDNTRPVGSYKAQRGIWRKLRLDPGHYVIVGSTYRPNQPGEFFIRIFSKTRNTLDFLREQAEIVLADLLTLQSVFVQFDEDSSGSISPFELSLALEAV-GMTCDGKVVQLLSERFVGGEPHLSFHGFVSCVSRLRKLFALFE-
LFLA-
>Gasterosteus_aculeatus_ENSGACG00000009801
LFADPCFPQQTSIG---
MPEDKAIKWRRPKEIGVNAVFVEDTIGTTDICQGQLGNCWLLAALSCLTMHPTLFEKVVPPNQSLSESYAGIFHFRFWQYGEWVEVVVDDRLPVREGRLLFSYSHTRNEYWSALVEKAYAKLVGCYGSLKGGNISEGMEDFTGGI
AYSLQVSSLWRSLTAALSRGSLLSCFIQASSNGKVTGDGLVKGHAYAITDSDKVTSDETLLLRLRNPWGFVEYRGPWSD-EWDDVVKQKEDGEFWISAEDFSRLFNVVELCSVNNSAGYCTVFVELLQKNRRK--
KTTNFPVGSYEAQRGSWREMQLDPGNYILVPSTFQPNQPGEFFVRIFSKTGNTLDFYWEQGDNEM-------
TIFLQFDEDSSGTMSPFELSAALQAVGGMQCDGKVLELLCERFASGELHMPFHSFVSCVTRMQKLFVFLETLVM--
>Danio_rerio_ENSDARG00000010758
LFSDSVFANQSSLG---
LPADKAVKWLRPKEITSNAVFVEGTMGTTDICQGQLGNCWLLAALSCLTMHPTLFVKVVPAGQSLSESYAGIFRFKFWQYGEWVEVVVDDRLPVREGRLLFSYSRTSNEFWSALVEKAYAKLIGSYGSLKGGIISEGMEDFTGGI
AYSLPVSSLWKAITAALARNSLLSCFIHATTVGTVTPEGLIKGHAYAITETGKVKSEEVFLLRLRNPWGFVEYCGPWSD-DWDFVDNKVEDGEFWISIEDFCRFYNTVEMCSVDSSAGYCTVLVELLQKYRRQ--
KDSNRAISSYRSIRSVWRKVHLEPGSYVILASTYRPNQQGEFFLRIYTKTGNIQDFLRAQAEKLLSSLRNLQSIFFQFDEDSSGTMSPFELSLALNAA-GVECDSVVVQMLWERFGAGEQYLPFYGFVSCVARLQVLFDLYE-
GLLAV
>Lepisosteus oculatus_GENSCAN00000002892_LG3
LFEDPEFPQQDSLG---
LPSDKAVVWRRPKEISKEPKFIEGTASTTDICQGQLGDCWLLAALSCLTLHQPLFNNVVPGDQSLSEEYAGIFHFKFWQYGQWVEVVVDDRLPVQRGRLLFSYSRTQNEFWSALLEKAYAKVNGCYASLKGGNISEAMEDFTGGI
ARSLPVKSLWRAVGESLSRGTLLSCFIQALA-----------------------DALRCSLCRL-----------PVLP-DWDRVDNRAEDGEFWIKADDLSHLFTTVEMCSVNASAGFGSVVLELLQKHRRQ--
KDSNKPVASYRQLRGVRKKVKLEPGHYVIVTSSYKPNVEGSFFLRIYAKTGNELGF----------------AIFSQFDEDSSGTMSPFELSLALQAA-GFQLDAPVLQLLWLRHGTADLSLTFDGLVACVGKLRKLFDLYE-G-
---
>Petromyzon_marinus_ENSPMAG00000009490
LFEDPDFPSLSSLHIRVQPTVNFIEWKRPWELVRHPEFIVSGATRADINQGTLPDCWLLSAIASLTLYPDLLAKVVPMGQSFHEDYAGIFLFRFCQYGEWVDVHVDDRLPTKKGELVFVQSGTGNEFWSALLEKAYAKLNGCYEA
LAYGFPVEALCDFTGGVGETFTMN-YWDGISRAIKKGAIICCGIETLAAEKVTPMGLVEGHAYSITGVIKLKGKTVQLIRVRNPYGKVEYTGPWSD-TWNEVPEA-
EDGEFWISDKDFRKNFHLVEICHQGVNAGHCHFLLSLTQKHRRKFRKIKEKPLVSFIARREVTMRMTLPPGNYIIIPCTESAGQEAQFLLRIFMEKAQQHSQMYEEFLLVWEFIKMTLKTYKMCDVDKSGTLSSTELPMALEHS-
RFKVGKLMQRLIMMRFSEPNLTISFDSFVCLLAKMYTAFNIFI-NFFNI
>Gadus_morhua_ENSGMOG00000007927
LFIDYTFPGQ-----------
KSVEWKRPMELCKPPQFIVDGATRMDVCQGNLGDCWFLSALASLSMHRSLLKRVVPPGQGFQNGYDGSFYFRFWQYGKWEEVRVDDLLPTQNGQLYYLRSPNRNEFWSCLLEKAYAKLKGGYQALEMGFPHEAMXDMTGGVTEVL
SVA-LPRALQHLLRKGALINCANCQ---EQKNEQGILFRHAYSLTAVERVE-GPVTLVRLHNPWGGTEWKGRWSDLEWNTVSESKEDGEFWMSVSDFRRNLEIIELCHMS-SAG-CSFLVALMQKHQKL----GLAPVL-
HVMRRETVLRGALAPGHYVIIPYASLPNQEGRFILRVLTEKGND---LWPGFKTLWYKIQSWTDAFLIHDKNGSKKLEYVEVSSAMKSA-GINMDSSVMQFVGLRYTEPDMTVSYPGFLYLMMKLDSMI----------
>Takifugu_rubripes_ENSTRUG00000009564
LFVDRHFPGE-----------PEVKWKRPKELCS-
PNFIVSGATRLDIRQGELNDCWLLSAIASLSLHPSLLERVVPLQQSFQDGYNGSFTFRFWMYGQWEEVRIDDLLPTLDNRLIYLSSPDKCEFWSPLLEKAYAKLKGGYRALNMGFPHEAMVDMTGGVTEVLNIA-
LPPLLSYLLSKGALINCATSQVKTEQKNELGIMFRHAYSLTAVEQVKHGTECLVRLLNPWGNTEWEGAWSD-EWNSVSISREDGEFWMSVSDFRQNFEIMEVCHQT-TAGFCSFVLALMQKHQRR----
KLRPVLSYSSRREVVLRSSLPPGRYLIIPSTSEPNQQGEFLLRVLTENENIS--LWIEFQDLWDKIRKWTDIFLVFDKNKTKRLEYQEVGPALKAA-GIVVDDLIMQLVGLRYTEPDMTISYPGFLYLLLKLENMIYKFQ-----
-
>Oryzias_latipes_ENSORLG00000000963
LFVDYQFPGE-----------AGLKWKRPKELCS-
PQFIIDGASRLDIVQGKLSDCWLLAAIASLAMHRSLLKKVVPLHQSFEEDYNGSFVFRFWQYGQWEEVRIDDLLPTENDKLVYLSSPERREFWSSLLEKAYAKLKGGYRALDMGFPHEAMVDMTGGVTEVFSIA-
LLEFLDYLLSKGALINCANSH---ETMNDLGILFRHAYALTAVEKVKNGPVDLVRILNPWGRTEWKGPWSDLEWQTVSLSREDGEFWMSISDFRQNFEVMEVCHLT-----CSFVLALMQKHQRR----MLQPVL-
HSSRREVVLRGSLPPGRYIIVPSTLEPNQEGAFLLRVLTERGNAA--LSVTGGLLWSLVDSWTDIFLMFDKNKTKHLEYQEVKPALMEA-GIKVDDLVMQLVGLRYTEPDMTVSFPGFLYLMMKMESMIHKFQ-STMYN
>Gasterosteus aculeatus_ENSGACG00000013147

```
LFVDHHFPGE-----------AGVKWKRPTELCS-
PQFIVNGATRLDVCQGALNDCWLLSAIASLSVNRFLLNKVMPLEQSFKDGYKGCFTFSFWQYGQWEEVKIDDFLPTKDNKLIYLSSPERDEFWSALLEKAYAKLKGGYRALDMGFPHEAMVDMTGGVTEVFNIS-
LPAFLRHLLARGALINCANCQ---EQSNGLGIMFRHAYSLTAVEQVKHGIVDLVRILNPWGNTEWSGPWSDLEWEAVNKKREDGEFWMSVSDFRRHFETMEVCHLT-SAG-CSFVLALMQKHQRR----AFRPVL-
YSMRREVVLRGSLPPGRYIIVPSTAEPNQPGAFLLRVLTEQGNAA--LWLDFRCLWDKIRRWTDIFLVFDKNKTNHLEYKEVAPALQKA-GIMVDDLIMQLVGLRYTEPDLTISYPGFLYLLMKLESMAHKFQ-STMYN
>Tilapia_CAPN12_ENSONIG00000001284
LFVDFHFPGE-----------PGVKWKRPKELCP-
PQFIVDGATRLDVHQGKLSDCWLLSAIASLSMYQSLLKKVVPLSQSFQDGYNGCFTFRFWQYGQWEDVQIDDLLPTVDNNLIFLSSPEKQEFWSALLEKAYAKLKGGYRALDMGFPHEAMVDMTGGVTEVLQVA-
LPTFLRHLLVRGALINCANCQ---ERRNELGIMFRHAYALTAVEKVRHGTEYLVRILNPWGNTEWEGPWSDFEWNTVSVRQEDGEFWMSVSDFRQNFEVMEVCHLT-TAG-CSFVLALMQKHQRR----
ASRPVLSYVTRREIVLRGSLPPGSYIIIPSTAETNQQGAFLLRVLMEQGNNA--LWPEFQSLWDKIRRWTDIFLMHDKNKTQRLEYQEVMPSLKAA-GIRVDELVMQLVGLRYTEPDMTISYPGFLYLLMKLESMIHKFQ-
STMYN
>Danio_rerio_ENSDARG00000055094
LYVDTRFPQN-----------NDLVWRRPKEICL-
PQFIVDGATRMDICQGVLGDCWFLSAMASLSLYPALLDRVVPAGQGFQQGYNGCFCFQFWQYGEWISVKIDDLLPTCNNQLIYLHSASRDEFWSALLEKAYAKLKGGYSALNMGFPHEAMVDMTGGITEVFMVA-
LGSFLRSLLEKGALINCANSQ---EKSNEFGILFRHAYSVTALETVKIGPVQLVRIRNPWGKAEWEGPWSDNEWHMVTAQQEDGEFWMSLADFRQNFELMEVCHLS-----CSFLLALMQKHTRQ------
SPLLSFQQRPELVLRGRLAPGHYIIIPAIQETNQSGEFLLRVLTEKGNTT--LLTEFQALWDKLRKWTSIFITFDKNKNQALDYLEISPALSAA-GIKVDEFILQLISLRYTEPDMTLSFPGFLFLLMKLDCMMRKFQ-NTLYN
>Lepisosteus_oculatus_GENSCAN00000011023_LG2
LFVDKSFP-------------PKLVWKRPKDICV-
PKFIVDGATRTDVCQGILSDCWFLSAVASLSQHRALLERVVPEGQGFDREYTGCFRFQFWQYGEWKEVEVDDFLPTEGGQLVYLHSAERDEFWSALLEKAYAKLKGGYQALQLGFPHEALVDMTGGVTEIFTIP-
LAGFLRPLLEKGALINCVNTQ---EKKNEQGILFKHAYSVTGLEKVRLGEADLVRVRNPWGHTEWTGPWSDNEWAKVSAQQIDGEFWMEVSDFQRNFNMMEVCHLSTSAGICSFLVALMQKHQRQ----
RAQPLITYNQQREVVIRSALAPGHYIIIPSTSMANQEGEFVLRVYTEKGNKA--LMEEFEALWEKFRKWTDIFVHFDKNRSQSLDYPEIIPALQAA-GLQVDDFVLQLIGLRYTDPDLTISYPGFLHLLLKLDIMI---------
-
>Xiphophorus_maculatus_ENSXMAG00000003111
------------------------TSRRLQELFP-
PKFIVDGASRLDICQGKLSDCWVLSAIASLANYPELLKKVVPLNQSFQDGYNGRFSFRFWQYGQWEEVRIDDFLPTYNNKLIYLSSPERHEFWSSLLEKAYAKLKGGYRALELGYPHEAMVDMTGGVTEVFSLP-
LPELLRNLLAKGALINCANGKVRREKKNEMGIMYRHAYSLTAVEKVKDGFKDLVRILNPWGHTEWLGAWSDNEWTKVSARQEDGEFCLTVIDTRSKIETLFACLTSG----CLFYLPASVSEEAG------
KGVLWHSTRREVVLRSSLPPGRYIIIPSTFEPNQQGEFLLRVLTEPGSDA--LWSDFQLLWDKIRKWTDIFLVYDTNKTHHLEYKEVEPALKAA-GIMVNDLVMQLVGLRYTESDLTISYPGFLFLLMKMESMIHKFQ-STLYS
>Xenopus_tropicalis_ENSXETG00000034127
-------------------------WRRPQEICA-
PRFILDGATRMDVCQGKLSNCWFLSAVACLSLYPQLLEKVVLPGQDFVGAYDGKFRFQFPGQGAAVDGDIHGCLRTGDGQXIRTKPAHKPEVWQSFAEMVYSGLKGGYSALQLGFAGEALVDMTGGVAQTCYTN-
LWVSLSHLLQQGALTCCGNTEVSYEMANSLGILSHHMYSVTGAKQVQHGSVSLLRVRNPWGHTEWSGPWRD-EWLSVIN--EDGEFWMDVEDFQKNFQVLEICHLGSSSGCCSIIVSVMLKHKRL----
CSQPVLVCDKKREAVLSTSLPPGRYIIIPSLETTSDEGEFLIRILTEKGSSA--MWEQFDRLWKNIITGSLIFSNLQSSKDRNLDKDQIGAALQSA-GLITDNFLVRLVQLRYADKDGSLSYSAFICCLLKIKAVTGMFE-
NALYS
```

(*d*-*l*) Codon alignment and tree files for 9 ancestral vertebrate classical calpain family members provided in nexus format (*d*) *CAPN1*, (*e*), *CAPN2*, (*f*) *CAPN3*, (*g*) *CAPN8*, (*h*) *CAPN9*, (*i*) *CAPN11*, (*j*) *CAPN12*, (*k*) *CAPN13* and (*i*) *CAPN17*.

(*d*) *CAPN1*

#NEXUS

```
BEGIN TAXA;

        DIMENSIONS NTAX = 23;
```

```
        TAXLABELS

                'XENOPUS_LAEVIS_NM_001093915'  'XENOPUS_TROPICALIS_ENSXETG00000023299'  'XENOPUS_LAEVIS_NM_001087016'  'GALLUS_GALLUS_NM_001044672'
'SUS_SCROFA_ENSSSCG00000012999'          'OVIS_ARIES_NM_001127267'            'HOMO_SAPIENS_NM_001198869'            'EQUUS_CABALLUS_XM_001917043'
'CERATOTHERIUM_SIMUM_XM_004437595'                   'MUS_MUSCULUS_ENSMUSG00000024942'                    'ANOLIS_CAROLINENSIS_ENSACAG00000016579'
'PELODISCUS_SINENSIS_ENSPSIG00000008053'             'SARCOPHILUS_HARRISII_ENSSHAG00000003383'             'LATIMERIA_CHALUMNAE_ENSLACG00000018300'
'CHILOSCYLLIUM_PUNCTATUM_CONTIG39834'                     'SCYLIORHINUS_CANICULA_CONTIG00627'                     'GADUS_MORHUA_ENSGMOG00000012126'
'TAKIFUGU_RUBRIPES_ENSTRUG00000008157'            'XIPHOPHORUS_MACULATUS_ENSXMAG00000008475'            'OREOCHROMIS_NILOTICUS_ENSONIG00000013071'
'GASTEROSTEUS_ACULEATUS_ENSGACG00000018991' 'DANIO_RERIO_ENSDARG00000045199' 'DANIO_RERIO_ENSDARG00000055338' ;

END;

BEGIN CHARACTERS;
        DIMENSIONS NCHAR = 2085;
        FORMAT
                DATATYPE = DNA
                GAP=-
                MISSING=?
                NOLABELS

        ;

MATRIX


ATGGCCGAGGTGCCAGTTTACGCCACCGGGATTTCGGCGCAGCTGCAGAAGCAACGCGACCACGAACTGGGGGTCGGGAAACACGAGAATGCGGTGAAATTTCTGGGACAGGATTATGACCGGATTCGCCACGAGTGCCTGCAAA
ACGGAAGCCTCTTTAAGGATGACACTTTCCCACCGTCGGCTTATTCTCTGGGCTTTAAGGAGCTCGGCCCAAACTCATCCAAAACCTATGGAGTGAAGTGGGTTCGGCCTTCGCAACTCGTCTCCAGCCCACAGTTTATTGTCGA
TGGTGCCACAAGAACTGACATCTGTCAGGGAGCGTTGGGCGACTGTTGGCTCCTCGCTGCTATTGCCTCCCTCACATTAAATGATACAGTCCTGCACCGGGTTGTACCTCATGGTCAGAGCTTCCAGGATAACTATGCCGGAGTC
TTTCATTTTCAGCTGTGGCAGTTTGGCGAGTGGATTGATGTGGTTATCGATGATTACCTCCCTGTTAAAGATGGAAAACTGGTATTCGTGCACTCGGCTTCTGGGAATGAATTCTGGAGTGCTTTACTGGAGAAGGCCTATGCCA
AGGCCAATGGCTGCTATGAGGCTCTCTCTGGAGGCAGCACATCAGAAGGATTTGAGGATTTTACTGGAGGGGTAACAGAGTGGTATGAGATGAAGAAAGCACCTAAAGATATATTTAACATTATTCTGAAGGCGTTGGAGAGAGG
TTCCCTCATGGGCTGTTCCATTGATATCACTAGTGCCACAGATATGGAGGCCGTAACATTCAAGAAGCTTGTCAAAGGTCACGCATACTCTGTTACAGGGGCCAAAGAGATCAACTTTAGGGGTCAGAACACAAAGCTGATTCGG
ATGAGAAATCCATGGGGTGAGGTGGAGTGGACAGGAGCATGGAGTGATGGTTCATCTGAATGGAACTCAGTGGATGCAGCAGACAGAGAAGAGTTGAGAATAAAGATGGAGGATGGAGAATTCTGGATGGCTTTTCAGGACTTCC
TTCGCGAATTTTCTCGCCTCGAGATCTGTAACCTCACGCCAGATGCGCTCAGCGCCCGCAGATACCGTAAGTGGAACACAACACTCTACAATGGGTCTTGGAGGAAAGGCAGCACGGCTGGCGGATGCAGAAATTACCCTGCCAC
ATTCTGGATCAACCCACAGTTCAAGATCAAACTGGAGGAGGAGGATGACGATGACTACGGCTCTGAAAAAGGCTGCACCTTCCTCCTTGCACTTATGCAAAAGAACAAGCGCAAAGAGAGGCGCGTTGGGAAAGATATGGAGACC
ATTGGATTTGCAGTCTATGAGGTTCCCAGAGAGTTTTACGGCCAGTCAGGTGTGCACCTAAAGCGGGATTTCTTCCTTCAAAACGCCTCCAGCGCTCGCTCCGAGCAGTTCATCAACCTGCGAGAGGTCAGCACCCGGCACAAAC
TGCGCCCCGGAGAATACATTGTGGTGCCTTCTACCTTTGAACCTAATGTGGAAGGAGACTTTGTCATCAGAGTCTTCTCTGAAAACAAACATGGCTCTGTTGAGATGGATGATCCTTTATCTGCTGACCTACCCCCTGAGGAAGT
TGAAAAGAATCTTTTCAAGCAACTGGCTATCAGTGCATCGGAGTTACAGTCCATTCTAAATAAAATTGTATCAAAGCATAAAGATCTTCGCACATCGGGATTTAGTTTGGAGTCGTGCCGCAGCATGGTGAACCTGATGGATAAA
GACGGGAATGGAAAATTAGGACTGGCGGAGTTCAATATTCTGTGGAATAAAATTAAGAATTACTTGACAATATTCCGGAAGTATGACGTGGATAAATCGGGAAGTATGAGCGCCTATGAGATGCGTCTGGCGGTGGAGTCTGCAG
GTTTCAAGTTGACCAACAGTCTGCATCAGCTCATCATCACCAGATACTCAGAGCCTGATATGGCCATGAATTTCGATAGCTTCACCTGCTGTCTCATCAGACTGGAGACTATGTTCCGATTTTTCCAGGGTATGGACCCACAGAA
ACAAGGAGTGATCAATTTTGACCTGTTTAGTTGGCTCCAAATGACAATGTTTGCA


ATGGCTGAGGTGCCCGTTTATGCCTCCGGGATTTCGGCGCAGCTGCAGAAGCAACGTGACCAAGAGCTGGGTGTTGGGAAACACGAAAATGCAGTTAAATTTCTGGGACAAGATTATGACCGGATTCTCCACGAGTGCCTGCAAA
ACAGGACCCTCTTTAAGGACGACACTTTCCCACCCTCTGCTTACTCTCTGGGCTTTAAGGAGCTCGGCCCAAACTCATCCAAAACCTATGGAGTAAAATGGGTTCGGCCTTCGCAACTCGTCTCCAACCCACAGTTTATTGTCGA
TGGTGCCACAAGAACAGACATCTGTCAGGGAGCCTTGGGTGACTGTTGGCTCCTCGCTGCTATCGCCTCCCTCACATTAAATGATACAATCCTGCACCGTGTAGTACCACATGGCCAGAGCTTTCAGGATAATTACGCTGGTGTC
TTTCATTTTCAGCTGTGGCAGTTTGGCGAGTGGGTCGATGTGGTTATCGATGATTACCTCCCTGTTAAAGATGGAAAACTTGTGTTTGTGCATTCGGCTTCCGGGAATGAATTCTGGAGTGCCTTGCTGGAGAAGGCCTATGCCA
AGGCCAATGGTTGCTATGAGGCTCTTTCTGGAGGCAGCACATCAGAAGGTTTTGAGGATTTTACAGGAGGTGTTACTGAGTGGTATGAGATGAAGAAAGCACCAAAAGATTTATTTAGCATTATCATGAAGGCTGTGGAGAGAGG
TTCCCTCATGGGCTGTTCCATTGATATCACTAGTGCCACTGATATGGAAGCTGTAACATTCAAGAAGCTTGTAAAAGGCCATGCATACTCTGTTACTGGAGCCAAAGAGATCAACTTCAGGGGTCGGAGCCAAAAGCTGATTCGA
```

ATGAGAAATCCATGGGGTGAGGTGGAGTGGACAGGTGCTTGGAGTGATAATTCATCTGAATGGAATTCTGTGGATGCAGCAGACAGTCAAGACTTGAGAATAAAAATGGAGGATGGAGAATTTTGGATGGCTTTTGAGGACTTCC
TTCGTGAATTTTCTCGCCTTGAGATCTGTAACCTCACACCCGATGCACTCACTGCCCGCAAATACCGCAAGTGGAACACAACCGTCTTTAATGGGAACTGGAGGAAAGGTAGCACAGCTGGCGGATGCAGAAATTTCCCTGCCAC
ATTCTGGATTAACCCACAGTTCAAGATAAAATTGGACGAGGAGGAT------------
GGCTCAGAAAAAGGTTGCACATTCCTGCTTGCACTTATGCAAAAGGACAAACGCAAAGAGAGGCGCTTTGGGAAAGATATGGAGACCATTGGATTTGCAGTCTATGAGGTTCCTTCTCAGTTTCATGGACAGTCTGGTGTGCACC
TAAAGCGGGACTTCTTCTTACAAAACGCCTCCCGTGCTCGCTCCGAGCAGTTCATAAACCTGCGAGAAGTCAGCACCCGCCACAAACTGCCCCCCGGAGAATACATTGTGGTCCCTTCTACCTTCGAACCTAATGTGGAAGGAGA
CTTTGTTGTTCGATTCTTTTCTGAAAACAAGCATGGCTCTGTTGAGATGGATGATCCATTATCTGCTGACCTACCTCCT---GAGGTAGAA------------------------
ATCAGTGCTACGGAGTTACAGTCCATTTTAAATAAAATTATGTCAAAACAT---------------------------------
TCGTGCCGCAGCATGGTGAACCTGATGGATAAAGATGGGAATGGGAAATTGGGACTGGTGGAGTTCAATGTTCTGTGGAATAAAATTAAAAATTACTTGACAGTATTCCGAAAGTTTGACATGGATAAATCTGGAAGTATGAGTG
CCTATGAGATGCGTTTAGCTGTGGAGTCTGCAGGTTTCAAGTTAACCAACAGTCTGCATCAGCTCATCATCACCAGATACTCAGAGCCTGATATGGCTGTGAATTTTGATAGCTTCACATGCTGTCTCATCAGACTGGAGACTAT
GTTCCGTTTTTTCCAGGGTATGGACACAGAGAAGAAAGGAGTGATTCATTTTGACATTTTTAAATGGCTCCAAATGACAATGTTCGCA

ATGGCTGAAGTGCCAGTTTATGCCACTGGGATTTCGGCGCAGCTGCAGAAGCAACGTGATCAAGATCTGGGTGTAGGGAAACATGAGAATGCAGTCAAATTTCTGGGACAGGATTATGACAGGATTCGCCACGAGTGCCTGCAAA
ACGGAACCCTCTTTAAGGACGACACATTCCCAGCCTCTGCTTACTCTCTGGGTTTTAAGGAGCTCGGCCCAAACTCATCCAAAACCTATGGTGTAAAATGGGTTCGGCCTTCGCAACTCGTCTCCAACCCACAGTTTATTGTCGA
TGGTGCCACAAGAACAGACATCTGTCAGGGAGCATTGGGTGACTGTTGGCTCCTCGCTGCTATCGCCTCCCTCACATTAAACGAAACAGTCCTGCACCGTGTAGTACCTCATGGCCAGAGCTTCCAGGAGAACTATGCTGGCGTC
TTTCATTTTCAGCTGTGGCAGTTTGGCGAGTGGGTCGATGTGGTTATCGATGATTTGCTCCCTGTTAAAGATGGAAAACTTGTGTTCGTGCACTCGGCTTCTGGGAATGAGTTCTGGAGTGCTTTGCTGGAGAAGGCCTATGCCA
AGGCCAACGGTTGTTATGAGGCTCTTTCTGGAGGCAGCACATCAGAAGGTTTTGAGGATTTTACTGGAGGTGTTACTGAGTGGTATGAGATGAAGAAAGCACCCAAAGATCTGTTTAACATTATTCTGAAGGCACTGGAGCGAGG
TTCCCTCATGGGCTGTTCCATTGATATCACTAGTGCCACTGATATGGAAGCCGTAACATTCAAGAAGCTTGTAAAAGGTCACGCATACTCTGTTACAGGGGCCAAAGAGATCAACTTTAGGGGTCAGAACACCAAGCTGATTCGG
ATGCGAAACCCATGGGGTGAGGTGGAATGGACAGGCGCATGGAGCGATAATTCATCTGAATGGAATTATGTGGGTCAAGCAGACAGTAACGAGTTGAGAATAAAGATGGAAGATGGAGAATTCTGGATGTCTTTTGAGGACTTCC
TTCGTGAATTTTCCCGCCTTGAGATTTGTAACCTCACTCCAGATGCACTCACTGCCCGCAGATACCGCAAGTGGAACACAACAGTGTACAATGGTTCTTGGAGGAAAGGAAGCACAGCTGGCGGATGCAGAAATTTCCCTGCCAC
ATTCTGGATTAACCCACAGTTCAAGATAAAACTGGAGGAGGAGGAT------------
GGCTCTGAAACCGGTTGCACCTTCCTCCTTGCACTTATGCAAAAGAACAAAAGAAAAGAGAGGCGCTTTGGGAAAGATATGGAGACCATTGGATTCGCAGTCTATGAGGTTCCCAGAGAGTTTCTTGGACAGTCTGGTGTGCATC
TAAAGCGGGACTTCTTCCTCCAAAACGCCTCCCGTGCTCGCTCAGAGCAGTTCATCAACCTGCGAGAGGTCAGCACCCGCCACAAACTGCCCCCCGGAGAATACATTGTGGTGCCTTCTACCTTTGAACCTAATGTGGAAGGAGA
CTTTGTCATTAGAGTCTTCTCTGAAAACAAGCATGGCTCTGTTGAGATGGATGATCCTTTATCTGCTGACCTACCTCCT---GAGGTTGAA------------------------
ATCAGTTATACGGAGTTACAGTCCATTTTAAATAAAATTGTATCAAAACAT---------------------------------
TCGTGCCGCAGCATGGTGAACCTGATGGATAAAGATGGAAATGGGAAATTAGGACTGGTGGAGTTCAATATTCTGTGGAATAAAATTAAGAATTACTTGACCATATTCCGAAAGTTTGACATGGATAAATCTGGAAGCATGAGCG
CATATGAGATGCGTCTAGCTTTGGAGTCTGCAGGTTTCAAGTTGACCAACAGTCTGCACCAGCTCATCATCACCAGATACTCGGAGCCTGATATGGCCGTGAATTTCGATAGCTTCACATGCTGTCTCATCAGGCTGGAGACTAT
GTTCCGATTTTTCCAGGGTATGGACACACAGAAACAAGGAGTGATCAATTTTGACCTGTTTAGTTGGCTCCAAATGACAATGTTTGCA

ATGGCGGAGCCCCCCGTGTTCTGCACCGGGGTGTCGGCGCAGGTGCAGCGGCAGCGGGCGAAGGCGTTGGGTTTGGGGCAGCACCAGAACGCCGTGCGGTTCCGCGGCCAGGACTACGCGGCGCTGCGCGACGACTGCCTGCGCT
CGGGGAGCCTCTTCAGGGACGAAACCTTTCCCCCTTCTGCTTCATCTCTGGGCTTCCGGGAGCTCGGGCCCGGCTCTTCCAAAACCCGCGGCGTGACGTGGAAGAGGCCGACGGAGCTGTGTCGGCACCCTCAGTTCATCGTAGA
CGGGGCGACGCGCACCGACATCTGCCAGGGAGCTCTGGGTGACTGCTGGTTGCTGGCAGCCATCGCGTCCCTAACGCTGAATGAGACCATCCTGCACCGCGTGGTGCCACATGGGCAGAGCTTCCAGAACGGCTACGCCGGGATC
TTCCACTTCCAGATCTGGCAGTTTGGTGAGTGGCAGGACGTGGTGGTGGACGATTACCTGCCCACCAAGGATGGCAAACTGCTCTTCGTCCACTCCGCCGAGGGCACCGAATTCTGGAGTGCTTTGCTGGAGAAAGCCTACGCCA
AGGTGAACGGGTGCTATGAAGCGCTGTCGGGCGGCAGCACCTCGGAGGGCTTCGAGGATTTCACCGGGGGGGTCACAGAGTGGTACGACCTGCGCAAGCCCCCCGCAGACCTCTACCAGATCATCCTGAAGGCGTTGGAGAGGGG
ATCCCTCCTGGGGTGCTCCATCGATATAACGAGTGCCTTCGACATGGAGGCGGTGACATTCAAGAAGCTGGTGAAGGGCCATGCCTACTCGGTGACGGGAGCCAAGCAGATCAGCTACCGCGGGCAGTCGCTGGGTCTGATCCGC
ATGCGCAACCCGTGGGGCGAGGTGGAGTGGACCGGAGCCTGGAGCGATAACTCCTCCGAATGGAACGCGGTGGAGCCGGCGCTGAGGCAGCAGCTGATGGTGAGAATGGAGGACGGGGAGTTCTGGATGTCTTTCCGGGACTTCC
TGCGGGAATTCACCCGCCTGGAGATCTGCAACCTCACCCCCGACGCCCTCCAGTCCCGGAAGTTCCGCAAGTGGAACACGCGGCTCTACGACGGCTCGTGGCGGCGCGGCAGCACGGCCGGCGGCTGCAGGAACTACCCCGCCAC
GTTTTGGATCAACCCTCAATTCAAGATCTGCCTGGAGGAGGTGGAC------------
GGCCGTGAGCCGGGCTGCAGCTTCCTGCTGGCCCTCATGCAGAAGCACCGACGCCGCGAGCGCCGCTACGGGAAGGACATGGAGACCATCGGCTTTGCTGTCTACGAGGTTCCCCCCGAGCACGTGGGGCACTCGGGGGTCCACC
TGCAGCGCGACTTCTTCCTGTCCAACGCGTCCCGCGCGCGCTCCGAGCAGTTCATCAACCTGCGGGAGGTCAGCACGCGCCTGCGGCTGCCGCCCGGGGAGTACATCGTGGTGCCCTCCACCTTCGAGCCCAACCGCGAGGGGGA
CTTCGTGCTGCGGGTCTTCTCCGAGAAGAGAGCCGACACCGAAGAAATCGATGACAAAATAGAGGCGAAGCTCCCGGAT---GAGGTGGAA------------------------
ATCAGCGTCACGGAGCTGCAGACGATCCTCAACCGCATCATCGCCAAACAT---------------------------------
TCGTGTCGCAGTATGGTCAACCTGATGGACAAAGATGGGAACGGGAAGTTGGGCCTGGTGGAGTTCAACGTCCTATGGAATAGGATCCGCAATTACCTGTCAGTGTTCCGCAAATTCGACCTGGACAAGTCTGGCAGTATGAGCG
CCTATGAGATGCGGATGGCACTGGAGGCATCGGGCTACAAACTGACCCAAAAGCTGCACCAGCTCCTCATCACGCGCTACGCCGAGCCCGACCTCGCCATCGACTTCGACTCCTTCGTCTGCTGCCTCGTCCGCCTGGAGACCAT
GTTCCGCTTCTTCCAGGCGATGGATGGGGACAATGACGGCGTCGTCACCTTTGGCTTGCTGCAGTGGCTGCAGCTCACCATGTTTGCC

ATGGCCGAGGTCCCGGTGTACTGCACTGGGGTGTCTGCACAAGTGCAGAAGCTGCGGGCCAAGGAGCTGGGCCTGGGCCGCCATGAAAATGCCATCAAGTACCTGGGCCAGGATTACGAGCAGCTCCGGGCTCACTGCCTGCAGA

GTGGGAGCCTCTTCCGTGATGAGGCTTTCCCTCCAGTGCCGCAGAGCCTGGGCTTCAAGGAACTGGGCCCCAACTCCTCCAAAACCTATGGCGTCAAGTGGAAGCGTCCCACGGAGCTGTTCTCAAACCCCCAGTTCATCGTGGA
TGGAGCCACCCGAACAGACATCTGCCAGGGAGCACTGGGGGACTGCTGGCTCCTGGCTGCCATCGCCTCCCTTACCCTCAACGACACCCTCCTGCACCGAGTAGTTCCACACGGCCAAAGCTTCCAGAATGGCTATGCTGGCATC
TTCCATTTCCAGCTGTGGCAGTTTGGGGAGTGGGTGGACGTGGTGGTGGATGACCTGCTGCCCACCAAAGACGGGAAGCTGGTGTTCGTGCACTCTGCCCAAGGCAACGAGTTCTGGAGCGCCCTGCTCGAGAAGGCTTATGCCA
AGGTGAATGGCAGCTACGAGGCCCTGTCCGGGGGCAGCACCTCCGAGGGCTTTGAGGACTTCACGGGCGGGGTCACCGAGTGGTACGAGCTGCGGAAGGCACCCAGCGACCTCTACAGCATCATCCTCAAGGCGCTGGAGCGAGG
CTCCCTGCTGGGCTGCTCCATTGACATCTCCAGCGTTCTGGACATGGAGGCTGTCACCTTCAAGAAGCTAGTGAAGGGCCACGCCTACTCCGTGACTGGGGCCAAGCAGGTGAACTACCAGGGCCAGATGGTGAACCTGATCCGG
ATGCGGAACCCCTGGGGCGAGGTGGAGTGGACGGGAGCCTGGAGTGATGGCTCCTCGGAGTGGAACGGTGTGGACCCCTACCAGCGGGATCAGCTCCGGGTCCGGATGGAGGATGGGGAGTTCTGGATGTCCTTCCGAGACTTCT
TGCGTGAGTTCACCCGCCTGGAGATCTGCAACCTGACGCCCGACGCGCTCAAGAGCCAGAGGGTCCGCAACTGGAACACCACCCTCTACGAGGGCACCTGGCGGCGGGGGAGCACCGCGGGGGGCTGCCGCAACTACCCAGCCAC
CTTCTGGGTGAACCCCCAGTTCAAGATCCGGCTGGAGGAGACGGAT------------
GGCCGTGAGTCAGGCTGCAGCTTCGTGCTCGCCCTCATGCAGAAACACCGCCGCCGGGAGCGCCGATTCGGCCGGGACATGGAGACCATCGGCTTTGCCGTCTATGAGGTCCCTCCGGAGCTGGTGGGCCAGCCG---
GTGCACCTGAAGCGGGACTTCTTCCTGGCCAACGCCTCTCGGGCCCGGTCCGAGCAGTTCATCAACCTGCGGGAGGTCAGTACCCGCTTCCGCCTGCCGCCCGGCGAGTACGTGGTGGTGCCTTCCACCTTCGAGCCCAACAAGG
AGGGCGACTTTGTGCTGCGTTTCTTCTCAGAGAAGAAAGCCGGGACCCAAGAGCTGGACGACCAGGTCCAGGCCATTCTCCCCGAC---GAGGTGGAG------------------------
ATCAGCGTCAGGGAGCTGCGCACCATCCTCAACAGGATCATCAGCAAACAC---------------------------------
TCCTGCCGCAGCATGGTCAACCTCATGGACCGTGATGGCAACGGGAAGCTGGGCCTGGTGGAGTTCAACATCCTGTGGAACCGCATCCGGAATTACCTGTCCATCTTCCGGAAGTTCGACTTGGACAAGTCAGGCAGCATGAGCG
CCTACGAGATGCGGATGGCCATCGAGTCTGCAGGGTTCAAGCTCAACAAGAAGCTGTTTGAGCTCATCATCACCCGCTACTCGGAGCCCGACCTGGCCGTGGACTTCGACAACTTCGTGTGCTGCCTGGTGCGGCTGGAGACCAT
GTTCCGATTTTTCAAGACTCTGGACACAGATCTGGATGGAGTTGTGACCTTTGACTTGTTTAAGTGGTTGCAGCTGACCATGTTTGCA

ATGGCCGAGTTCCCGGTGTACTGCACCGGGGTGTCTGCACAAGTGCAGAAGCAGCGGGCCAAGGAGCTGGGCCTGGGCCGCCATGAAAATGCCATCAAGTACCTGGGCCAGGATTACGAGCAGCTGCGGGTTCACTGCCTGCAAA
GGGGGGCCCTTTTCCGTGATGAGGCTTTCCCCCCAGTGCCCCAGAGCCTGGGCTTCAAGGAGCTGGGCCCCAACTCCTCCAAAACCTATGGCATCAAGTGGAAGCGTCCCACGGAGCTGTTCTCAAACCCCCAGTTCATCGTGGA
TGGAGCCACCCGCACAGACATCTGCCAGGGCGCACTGGGGGCATGTTGGCTTCTGGCTGCCATCGCCTCCCTTACCCTCAATGACACGCTCCTGCACCGAGTAGTTCCACATGGACAAAGCTTCCAGGATGGCTACGCGGGCATC
TTCCATTTCCAGCTGTGGCAGTTTGGTGAGTGGGTGGATGTGGTGGTGGATGACCTGCTGCCCACCAAGGACGGGAAGCTGGTGTTCGTGCACTCTGCCCAAGGCAACGAGTTCTGGAGCGCCCTGCTGGAGAAGGCCTATGCCA
AGGTGAACGGCAGCTACGAGGCCCTCTCAGGAGGCAGCACATCTGAGGGCTTTGAGGACTTCACCGGCGGAGTCACCGAGTGGTATGAACTGCGGAAGGCGCCCAGCGACCTCTACAACATCATCCTCAAGGCCCTGGAGCGCGG
CTCCCTGCTGGGCTGCTCCATCGATATCTCCAGCATTCTGGACATGGAGGCTGTCACCTTCAAGAAGCTGGTGAAGGGTCACGCCTACTCTGTGACTGGGGCCAAGCAGGTGAACTACCAGGGCCAGATGGTGAACCTGATCCGG
ATGCGGAACCCCTGGGGCGAGGTGGAATGGACGGGAGCCTGGAGTGACGGCTCCTCGGAGTGGAACGGCGTGGACCCCTTACGTGCGGGAGCAGCTCCGGATCAAGATGGAGGATGGGGAGTTCTGGATGTCATTCCGAGACTTCA
TGCGTGAATTCACCCGCCTGGAGATCTGCAACCTGACGCCTGACGCACTCAAGAGCCAGAGGTTCCGCAACTGGAACACCACCCTGTACGAGGGCACCTGGCGGCGGGGGAGCACCGCGGGGGGCTGCCGCAACTACCCAGCCAC
TTTCTGGGTGAACCCCCAGTTCAAGATCCGGCTGGAGGAGACGGAT------------
GGTCGCGAGTCAGGCTGCAGCTTCTTGCTCGCCCTCATGCAGAAGCACCGCCGTCGAGAGCGCCGATTCGGCCGTGACATGGAGACCATAGGTTTCGCTGTCTACGAGGTCCCTCCGGAGCTGGTGGGCCAGCCGGCCGTGCATC
TGAAGCGAGACTTCTTCCTGGCCAATGCCTCCCGGGCCCGGTCTGAGCAGTTCATCAACCTGCGGGAGGTCAGCACCCGCTTCCGCCTGCCGCCCGGGGAGTACGTGGTGGTGCCCTCTACCTTCGAGCCCAACAAGGAAGGCGA
CTTTGTGCTGCGTTTCTTCTCAGAGAAGAGCGCAGGGACCCAAGAGCTGGATGACCAGGTCCAGGCCAATCTCCCTGAT---GAGGTGGAG------------------------
ATCAGCGTCAAGGAGCTGCGGACCATCCTCAACAGGATCATCAGCAAACAC---------------------------------
TCCTGCCGCAGCATGGTCAACCTCATGGATCGCGACGGCAATGGCAAACTGGGCCTGGTGGAGTTCAACATCCTATGGAACCGGATCCGGAATTACCTGTCCATCTTCCGGAAGTTTGACCTGGACAAGTCGGGCAGCATGAGTG
CCTACGAGATGCGGATGGCCATTGAGTTTGCAGGCTTCAAGCTCAACAAGAAGCTGTACGAGCTCATTATCACCCGCTACTCGGAGCCAGACCTGGCCGTGGACTTCGACAACTTTGTGTGCTGCCTGGTGCGGCTGGAGACCAT
GTTCCGGTTTTTCAAAACTCTGGACACCGATCTGGATGGAGTGGTGACCTTTGACTTGTTTAAGTGGCTACAGCTGACCATGTTTGCA

ATGTCGGAGATCCCGGTGTACTGCACTGGGGTGTCAGCCCAAGTGCAGAAGCAGCGGGCCAGGGAGCTGGGCCTGGGCCGCCATGAGAATGCCATCAAGTACCTGGGCCAGGATTATGAGCAGCTGCGGGTGCGATGCCTGCAGA
GTGGGACCCTCTTCCGTGATGAGGCCTTCCCCCCGGTACCCCAGAGCCTGGGTTACAAGGACCTGGGTCCCAATTCCTCCAAGACCTATGGCATCAAGTGGAAGCGTCCCACGGAACTGCTGTCAAACCCCCAGTTCATTGTGGA
TGGAGCTACCCGCACAGACATCTGCCAGGGAGCACTGGGGGACTGCTGGCTCTTGGCGGCCATCGCCTCCCTCACTCTCAACGACACCCTCCTGCACCGAGTGGTTCCGCACGGCCAGAGCTTCCAGAATGGCTATGCCGGCATC
TTCCATTTCCAGCTGTGGCAATTTGGGGAGTGGGTGGACGTGGTCGTGGATGACCTGCTGCCCATCAAGGACGGGAAGCTAGTGTTCGTGCACTCTGCCGAAGGCAACGAGTTCTGGAGCGCCCTGCTTGAGAAGGCCTATGCCA
AGGTAAATGGCAGCTACGAGGCCCTGTCAGGGGGCAGCACCTCAGAGGGCTTTGAGGACTTCACAGGCGGGGTTACCGAGTGGTACGAGTTGCGCAAGGCTCCCAGTGACCTCTACCAGATCATCCTCAAGGCGCTGGAGCGGGG
CTCCCTGCTGGGCTGCTCCATAGACATCTCCAGCGTTCTAGACATGGAGGCCATCACTTTCAAGAAGTTGGTGAAGGGCCATGCCTACTCTGTGACCGGGGCCAAGCAGGTGAACTACCGAGGCCAGGTGGTGAGCCTGATCCGG
ATGCGGAACCCCTGGGGCGAGGTGGAGTGGACGGGAGCCTGGAGCGACAGCTCCTCAGAGTGGAACAACGTGGACCCATATGAACGGGACCAGCTCCGGGTCAAGATGGAGGACGGGGAGTTCTGGATGTCATTCCGAGACTTCA
TGCGGGAGTTCACCCGCCTGGAGATCTGCAACCTCACACCCGACGCCCTCAAGAGCCGGACCATCCGCAAATGGAACACCACACTCTACGAAGGCACCTGGCGGCGGGGGAGCACCGCGGGGGGCTGCCGAAACTACCCAGCCAC
CTTCTGGGTGAACCCTCAGTTCAAGATCCGGCTGGATGAGACGGAT------------
GACCGCGAGTCAGGCTGCAGCTTCGTGCTCGCCCTTATGCAGAAGCACCGTCGCCGCGAGCGCCGCTTCGGCCGCGACATGGAGACTATTGGCTTCGCGGTCTACGAGGTCCCTCCGGAGCTGGTGGGCCAGCCGGCCGTACACT
TGAAGCGTGACTTCTTCCTGGCCAATGCGTCTCGGGCGCGCTCAGAGCAGTTCATCAACCTGCGAGAGGTCAGCACCCGCTTCCGCCTGCCACCCGGGGAGTATGTGGTGGTGCCCTCCACCTTCGAGCCCAACAAGGAGGGCGA
CTTCGTGCTGCGCTTCTTCTCAGAGAAGAGTGCTGGGACTGTGGAGCTGGATGACCAGATCCAGGCCAATCTCCCCGAT---GAGGTGGAG------------------------
ATCAGCGTGAAGGAGTTGCGGACAATCCTCAATAGGATCATCAGCAAACAC---------------------------------
TCGTGCCGCAGCATGGTGAACCTCATGGATCGTGATGGCAATGGGAAGCTGGGCCTGGTGGAGTTCAACATCCTGTGGAACCGCATCCGGAATTACCTGTCCATCTTCCGGAAGTTTGACCTGGACAAGTCGGGCAGCATGAGTG

CCTACGAGATGCGGATGGCCATTGAGTCGGCAGGCTTCAAGCTCAACAAGAAGCTGTACGAGCTCATCATCACCCGCTACTCGGAGCCCGACCTGGCGGTCGACTTTGACAATTTCGTTTGCTGCCTGGTGCGGCTAGAGACCAT
GTTCCGATTTTTCAAAACTCTGGACACAGATCTGGATGGAGTTGTGACCTTTGACTTGTTTAAGTGGTTGCAGCTGACCATGTTTGCA

ATGGCTGAGATCCCAATATACTGCACCGGGGTGTCTGCACAAGTGCAGAAGCACCGGGCCAAGGAGCTGGGCCTGGGCCGCCATGAAAACGCCATCAAGTACTTGGGCCAGAATTATGACCAGCTGCAGGCTCAGTGCCTGCAGA
GTGGGGTCCTCTTTCGTGATGAGGCCTTCCCCCCAGTGCCCCAAAGCTTGGGCTTTAAGGAACTGGGCCCCAACTCCTCCAAAACCTATGGCATCAAGTGGAAGCGTCCCACGGAGCTGTTCTCAAACCCCCAGTTCATCGTGGA
TGGAGCTACCCGCACAGACATCTGCCAGGGAGCCCTGGGGGACTGTTGGCTGCTGGCTGCCATCGCCTGCCTCACCCTCAACGACACCCTCCTGCACCGAGTGGTTCCTCATGGCCAAAGCTTCCAGAATGGCTATGCTGGCATC
TTCCATTTCCAGCTGTGGCAGTTTGGGGAGTGGGTGGACGTGGTCGTGGATGACCTGCTGCCCACCAAGGACGAGAAGCTGGTGTTCGTGCACTCCGCCCAAGGCAACGAGTTCTGGAGCGCCCTGCTCGAGAAGGCCTATGCCA
AGGTGAACGGCAGCTATGAGNGCCTCTCGGGGGGCAGCACCTCAGAGGGCTTTGAGGACTTTACAGGCGGGGTCACCGAGTGGTACGAGNCGCGCAAGGCGCCCAGCGACCTCTACCACATCATCCTCAAGGCGCTGGAACGAGG
CTCCCTGCTGGGCTGCTCCATTGACATCTCCAGTGTTCTGGACATGGAGGCCGTCACCTTCAAGAAGCTGGTGAAGGGCCATGCCTACTCTGTGACTGGGGCCAAGCAGGTTAACTACCAGGGCCAGATGGTGAGCCTGATCCGG
ATGCGGAACCCCTGGGGCGAGGTGGAGTGGACAGGAGCCTGGAGTGATGGCTCCTCGGAGTGGAACAACGTGGACCCTTACGAACGGGAGCAGCTCCGGGTCAAGATGGAGGACGGAGAGTTCTGGATGTCATTCCGAGACTTCA
TGCGTGAGTTCACCCGCCTGGAGATCTGCAACCTGACGCCTGACGCGCTCCAGAGCCGGAAGTTCCGCAAATGGAACACCACGCTCTACGAGGGCACCTGGCGGCGGGGGAGCACGGCGGGGGGCTGCCGGAACTACCCAGCCAC
CTTCTGGGTGAACCCTCAGTTCAAGATCCAGCTGGAGGAGCCAGAT------------
GGCCGCGAGTCAGGCTGCAGCTTCGTGCTCGCCCTCATGCAGAAGAACCGCCGCCGCGAGCGCCGCTTCGGCCGCGATATGGAGACCATTGGCTTCGCGGTCTACGAGCCCCCGCCCCAGCTGGTGGGCCAGCCGGCCGTGCACC
TGAAGCGGGACTTCTTCCTGGCCAACTCGTCGCGGGCCCGATCGGAGCAGTTTATCAACCTGCGCGAGGTCAGCACCCGCTTCCGCCTGCCGCCCGGGGAGTACGTGGTGGTGCCGTCCACCTTCGAGCCCAACAAGGAGGGCGA
CTTCGTGCTACGCTTCTTCTCGGAGAAGAAAGCCGGGACCCAGGAGCTGGACGACCAGATCCAGGCCAATCTCCCTGAT---GAGGTGGAG-----------------------
ATCAGCGTCAGGGAGCTGCAGACCATTCTCAACAGGATCATCAGCAAACAC--------------------------------
TCGTGCCGCAGCATGGTGAACCTCATGGATCGGGACGGCAACGGGAAGCTGGGCCTGGTGGAGTTCAACATCCTGTGGAACCGCATCCGGAATTACCTGTCCATCTTCCGGAAGTTTGACTTGGACAAGTCGGGCAGCATGAGCG
CCTACGAGATGCGGATGGCCATCGAGTCTGCAGGCTTCAAGCTCAACAAGAAGCTGTATGAGCTCATTATCACCCGCTACTCGGAGCCCGACCTGGCCGTGGACTTTGACAACTTCGTGTGCTGCCTGGTGCGGCTGGAGACCAT
GTTCCGGTTTTTCAAAACTCTGGACACAGATCTGGATGGAGTCGTGACCTTTGACTTGTTTAAGTGGTTACAGCTGACCATGTTTGCC

ATGGCTGAGATCCCGGTATACTGCACTGGGGTGTCTGCACAAGTGCAGAAGCTTCGGGCCAAGGAGCTGGGCCTGGGCAGCCATGAAAACGCCATCAAGTACCTGGGCCAGAATTATGAGCAGCTGCAAACTCAATGCCTGCAGA
AGGGGGTCCTCTTTCGTGATGAGGCCTTCCCCCCAGTGCCCCAGAGCTTGGGCTATAAGGACCTGGGCCCCAACTCCTCCAAAACCTATGGCATCAAGTGGAAGCGTCCCACGGAGCTGTTCTCAAACCCCCAGTTCATCGTGGA
TGGAGCCACCCGCACAGACATCTGCCAGGGAGCCCTGGGGGACTGCTGGCTCCTGGCTGCCATCGCCTCCCTCACCCTCAATGACACCCTCCTGCACCGGGTGGTTCCTCACGGCCAAAGCTTCCAGAATGGCTATGCCGGCATC
TTCCATTTCCAGCTGTGGCAGTTTGGGGAGTGGGTGGATGTGGTCGTGGATGACCTGCTGCCCACCAAGGACGGGAAGCTGCTGTTCGTGCACTCTGCCCAAGGCAACGAGTTCTGGAGCGCCCTGCTCGAGAAGGCCTATGCCA
AGGTGAACGGCAGCTATGAGGCCCTGTCGGGGGGCAGCACCTCAGAGGGCTTTGAGGACTTCACAGGCGGGGTCACCGAGTGGTACGAGCTGCGCAAGGCGCCCAGCGACCTCTACCAAATCATCCTCAAGGCGCTGGAGCGAGG
CTCCCTGCTGGGCTGCTCCATTGACATCTCCAGCGTTCTGGACATGGAGGCGGTTACCTTCAAAAAGCTGGTGAAGGGCCACGCCTACTCTGTGACTGGGGCCAAGCAGGTGAACTACCGGGGCCAGATGGTGAACCTGATCCGG
ATGCGGAACCCCTGGGGTGAGGTGGAGTGGACGGGAGCCTGGAGTGACAGCTCCTCGGAGTGGAACAATGTAGACCCTTATGAACGGGAGCAGCTCCGGGTCAAGATGGAGGACGGGGAGTTCTGGATGTCATTCCGAGACTTCT
TGCGTGAGTTCACCCGCCTGGAGATCTGCAACCTGACGCCTGACGCGCTCCAGAGCAGGAGGTTCCGAAAATGGAACACCATGCTCTATGAGGGCACCTGGCGGCGGGGGAGCACCGCAGGGGGCTGCCGGAACTACCCAGCCAC
TTTCTGGGTGAACCCTCAGTTCAAGATCCGGCTGGAGGAGACGGAT------------
GGTCGCGAATCAGGCTGCAGCTTCGTGCTCGCCCTCATGCAGAAGAACCGCCGCCGCGAGCGCCGCTATGGCCGCGATATGGAGACCATTGGCTTCGCCGTCTATGAG---
GTCCCTGAGCTGGTGGGCCAGCCGGCCGTGCACCTGAAGCGTGAATTCTTCCTGGCCAACTCGTCCCGGGCCCGGTCGGAGCAGTTCATCAACCTGCGGGAGGTCAGCACGCGCTTCCGCCTGCCGCCCGGGGAGTACGTGGTGG
TGCCCTCCACCTTCGAGCCCAACAAGGAGGGCGACTTCGTGCTGCGCTTCTTCTCAGAGAAGAAAGCCGGGACCCAGGAGCTGGACGACCAGATCCAGGCCAATCTCCCTGAC---GAGGTGAAG--------------------
----ATCAGCGTCAAGGAGCTGCGGACCATTCTCAACAGGATCATCAGCAAACAC--------------------------------
TCGTGCCGCAGCATGGTGAACCTCATGGACCGGGACGGCAACGGGAAGCTGGGCCTGGTGGAGTTCAACATCCTGTGGAACCGCATCCGGAACTACCTGGCCATCTTCCGGAAGTTTGACTTGGACAAGTCAGGCAGCATGAGCG
CCTATGAGATGCGGATGGCCATCGAGTCTGCAGGCTTCAAGCTCAACAAGAAACTGTATGAGCTCATTATCACCCGCTACTCGGAGCCCGACCTGGCCGTGGACTTCGACAACTTCGTGTGCTGCCTGGTGCGGCTGGAGACCAT
GTTCCGGTTTTTCAAAACTCTGGACACAGATCTGGATGGAGTCGTGACCTTTGACTTGTTTAAGTGGTTACAGCTGACCATGTTTGCA

ATGACAGAGTTACCTGTGTACTGCACCGGGGTGTCAGCCCAAGTACAGAAGAAGCGGGACAAGGAGCTGGGCTTGGGCCGCCATGAAAACGCCATCAAGTACCTGGGCCAGGATTATGAAACGCTTCGGGCAAGATGCCTGCAGA
GTGGGGTCCTCTTCCAAGACGAGGCCTTCCCTCCGGTTTCTCATAGCCTGGGCTTCAAGGAACTGGGTCCTCATTCCTCTAAAACCTATGGCATCAAATGGAAGCGGCCTACGGAACTGATGTCAAACCCCCAGTTCATCGTGGA
TGGAGCCACCCGGACAGACATCTGCCAGGGAGCACTGGGGGACTGTTGGCTCCTGGCTGCCATTGCCTCCCTCACCCTCAACGAGACTATTCTGCACCGAGTGGTTCCCTACGGCCAGAGCTTTCAGGATGGCTACGCTGGCATC
TTTCATTTCCAGCTGTGGCAGTTCGGGGAGTGGGTCGACGTGGTCATAGATGACTTGCTGCCCACCAAGGACGGGAAGCTGGTGTTCGTGCACTCTGCCCAGGGCAACGAATTCTGGAGCGCACTGCTGGAGAAAGCCTATGCTA
AAGTGAATGGCAGCTATGAGGCTCTTTCGGGAGGCTGCACCTCAGAGGCCTTCGAGGACTTTACCGGTGGGGTCACTGAGTGGTACGACCTGCAGAAGGCCCCCAGCGACCTCTACCAGATCATTCTAAAGGCCCTGGAACGAGG
CTCCTTGCTGGGCTGCTCCATTAATATCTCCGATATCCGTGATTTAGAGGCTATTACTTTTAAGAACCTGGTGAGGGGCCATGCGTACTCTGTGACGGGCGCCAAGCAGGTAACTTACCAGGGCCAGCGGGTGAACCTAATCCGG
ATGCGGAACCCCTGGGGTGAAGTGGAGTGGAAAGGACCCTGGAGTGACAGCTCCTATGAGTGGAACAAAGTGGACCCCTATGAACGAGAGCAGCTGAGGGTCAAGATGGAGGATGGGGAGTTCTGGATGTCGTTCCGAGACTTCA
TCCGTGAATTCACCAAACTGGAAATCTGCAACCTTACACCGGACGCCCTTAAGAGCAGGACCCTCCGGAATTGGAATACCACATTTACGAGGGCACCTGGCGTCGGGGAAGCACCGCTGGAGGCTGCAGGAACTACCCAGCTAC
CTTCTGGGTAAACCCCCAGTTCAAGATCCGGTTGGAGGAGGTGGAT------------

AACCGGGAGTCGGGCTGCAGCTTCTTGCTGGCCCTCATGCAGAAACACCGCCGCAGGGAGCGTCGCTTTGGCCGGGACATGGAGACCATCGGTTTTGCAGTGTACCAGGTCCCTCGGGAGCTGGCGGGTCAGCCT---
GTGCACTTGAAGCGTGATTTCTTCCTGGCCAACGCTTCTCGGGCGCAGTCAGAGCACTTCATCAACCTTCGGGAAGTCAGCAACCGTATCCGCCTGCCGCCCGGGGAGTACATAGTGGTGCCCTCCACCTTCGAGCCCAACAAAG
AAGGCGACTTCCTGCTGCGCTTCTTCTCAGAGAAGAAGGCTGGGACCCAGGAACTAGATGACCAGATCCAGGCCAACCTCCCTGAT---GAGGTTGAG------------------------
ATCAGCGTCAAGGAGCTACAGACCATCTTGAACAGGATCATTAGCAAACAC---------------------------------
TCGTGCCGCAGCATGGTGAACCTCATGGATCGAGATGGCAACGGGAAGCTGGGTCTGGTGGAGTTCAACATCCTGTGGAACCGCATCCGAAATTACCTGACCATCTTCCGGAAGTTTGACCTGGACAAGTCTGGCAGCATGAGTG
CCTATGAGATGAGGATGGCCATCGAGGCTGCAGGCTTCAAGCTTAACAAGAAGCTGCATGAACTCATAATCACCCGCTACTCGGAGCCCGACCTGGCCGTGGACTTTGACAACTTTGTGTGCTGTCTTGTGCGGCTGGAGACCAT
GTTCCGGTTTTTCAAACTTCTGGACACAGACCTGGATGGTGTTGTGACCTTTGATCTATTTAAGTGGCTCCAGCTGACTATGTTTGCC

ATGTCGGAGGTCCCCGTTTACTGCACCGGAGTCTCGGCACAAGTCCAGAAGCAGCGAGCCAAAGACCTGGGTTTGGGGAAACACGAGAACGCCGTCAAGTACTTAGGCCAGGATTACGAAAGGCTCCGGAACGAGTGCTTGCAGA
GCGGAGGCCTCTTCCGAGACGAGACCTTCCCGGCCTCCGCCGCCTCCTTGGGATTTAAGGAGCTGGGGCCGAATTCCTCCAAGACGTACGGCGTCAAATGGAAACGGCCCACGGAACTGTGCCGCAACCCGCTTTTCATTATCGA
CGGAGCCACCCGCACGGACGTCTGCCAAGGAGCTCTGGGCGACTGCTGGCTCCTCGCCGCCATTGCCTCCCTCACCCTGAACGACCCCCTTCTCCATCGTGTCGTCCCTCATGGGCAAAGCTTCCAAAACGGCTATGCGGGGATC
TTCCATTTCCAGATCTGGCAGTTCGGCGAGTGGGTGGACGTGGTGGTGGATGACCTGCTGCCAACAAAAGACGGCAAGCTGGTGTTTGTGCATTCCGCAGAAGGCAATGAGTTTTGGAGCGCTCTGCTTGAGAAGGCCTATGCCA
AGCTGAATGGCTGCTACGAGGCTCTTTCCGGGGGAAGCACCTCTGAAGGCTTTGAAGACTTCACTGGAGGTGTGACGGAGTGGTACGATCTCCGGAAGCCACCCAGCGACCTCTTCCAGATCATCCTGAAGGCCCTGGAGAGGGG
ATCTCTCATGGGTTGCTCCATTGACATCACAAGTGCTTTCGACATGGAAGCGGTCACTTTCAAGAAGCTGGTGAAAGGCCACGCGTATTCGGTCACCGGCGGCGAGCAGATCACCTACCGCGGGCAGAGGGTGAGCCTGATACGG
ATACGAAACCCCTGGGGGGAAGTGGAATGGACCGGCGCTTGGAGCGACAATTCCGCGGAGTGGAACACGGTGGACCCATCGGTGGGGCAGCAACTGAGGATTAAAATGGAAGATGGAGAATTCTGGATGTCCTTCCAAGACTTCC
TCCGGGAGTTCTCTCGCCTTGAGATCTGCAACCTCACGCCGGATGCGCTGAAGTCGCGCAAGTTCCGGAAGTGGAACACGACGCTTTACGACGGCACCTGGCGGAGAGGGAGCACGGCAGGCGGCTGCAGGAACTACCCAGCCAC
ATTCTGGATCAATCCCCAATTCAAAGTCCGTCTCGAGGAGGTCGAC------------
AGCCGGGAGCCTGGCTGCAGTTTTGTCATGGCGCTGATGCAGAAGCACCGGCGCCGAGAGAGGCGCCTCGGCAAGGACATGGAGACCATTGGCTTTGCTGTCTATGAGGTACCACCGGAGTATGTAGGGCAACCCTCGGTCCACC
TGAAGCGCGACTTCTTCCTCTCCAACTCCTCGCGGGCCCGTTCTGAGCAGTTCATCAACCTGCGCGAGGTCAGCACCCGCTTCAAGCTGCCTCCCGGAGAGTATATCGTGGTGCCTTCCACCTTTGATCCCAACAAGGAAGGGGA
CTTTGTGCTGAGGGTCTTCTCTGAGAACAAAGCAGGGACCGTGGAGATGGACGACGACATCCAGGCCAATCTCCCAGAT---GAGGTGGAG------------------------
ATCAGCGTTACCGAACTGCAGACAATCCTCAACAGGATCATCGGAAAACAC---------------------------------
TCCTGCCGCAGCATGGTGAACCTCATGGACAGGGATGGAAATGGGAAACTCGGTTTGGTGGAGTTCAACATCCTTTGGACCAAAATCAGAGGCTATTTGGTGGTCTTTAGGAAGTTCGATTTGGACAAGTCGGGAACCATGAGTG
CCTATGAGATGCGGATGGCTCTGGAATCTGCAGGTTTCAAGCTGAACAAGAAGCTCTACGAGTTGATCATCACCCGCTACGCCGAGCCTGACTTGGCGATCGACTTTGACAACTTCGTCTGCTGTTTAGTGCGACTGGAAACCAT
GTTCCGGTTTTTCCAGGCCCTGGACTCTGACAAAGATGGCATCGTCACCTTTGACTTGGTTAAGTGGATCCAGCTCACCATGTTTGCC

ATGGCAGAAATTCCAGTGTACTGCACAGGCGTCTCGGCTCAGGTCCAGAAGCAGAGAGCTAAGGATCTAGGTCTGGGAAAGCACGAGAATGCAGTGAAGTACTTGGGCCAGGACTTTGAGACGCTTCGGAATGCATGCTTACAGA
GAGGCAGCCTCTTCCAAGATGACACCTTCCCTCCTACAGCATCTTCCCTGGGCTTCAAAGAGTTGGGTCCAAACTCCTCTAAAACCTATGGAATCAAGTGGAAGAGACCCACGGAAATGTGTTCCAACCCGCAGTTCATTGTTGA
CGGAGCCACCCGCACAGACATCTGCCAGGGAGCCCTCGGTGACTGCTGGCTGCTAGCTGCTATTGCTTCCCTCACCTTGAATGATACCATCCTCCATCGAGTGGTCCCTCATGGGCAGAGCTTCCAGAGTGGATATGCTGGCATC
TTCCACTTCCAGATCTGGCAGTTTGGGGAGTGGGTGGATGTTGTAGTGGACGATCTTCTCCCGACGAAAGATGGCAAGCTGACCTTTGTACACTCAGCTGAAGGCAATGAGTTCTGGAGTGCTCTACTCGAGAAGGCTTATGCCA
AGGTGAATGGTTGCTATGAAGCTCTGTCTGGAGGAAGCACCTCTGAGGGCTTTGAAGACTTCACTGGAGGGGTCACTGAATGGTACGAGCTTCGGAAACCACCCAACGACCTTTACCAAATTATTTTGAAAGCTTTGGAGAGGGG
ATCTCTGCTTGGCTGCTCCATTGATATTACCAGTGCTTTTGACATGGAGGCTGTAACATTTAAAAAGCTGGTGAAGGGTCACGCCTATTCGGTCACAGGAGCCAAACAGATTAACTTCCGAGGACAGTCTGTTAATCTAATTCGA
ATGCGAAACCCATGGGGAGAGGTGGAATGGACTGGAAGCTGGAGTGATAACTCCTCTGAGTGGAACGTGGTGGATCCATCTGTGGGACAGCAGCTGAGGATTAAAATGGAAGATGGGGAATTCTGGATGTCCTTCCAAGACTTTT
TGCGAGAGTTCTCACGGCTGGAGATCTGTAACCTTACCCCAGATACGCTGAAATCGCGCAAGCTCCGCAAGTGGAACACAACACTTTATGATGGCACCTGGAGAAGGGGGAGCACAGCTGGAGGCTGCAGGAATTATCCAGCTAC
ATTCTGGATAAACCCTCAGTTTAAAATTCGACTGGAAGAGGTGGAT------------
AGCAGAGAATCGGGCTGTAGCTTCGTCATGGCACTCATGCAGAAACACCGTCGCAGGGAAAGGCGATTTGGGAAGGACATGGAGACCATTGGCTTTGCTGTTTATGAGGTTCCTCGTGAGTTTGTAGGTCAGTCTGCAGTTCACC
TGAAACGAGATTTCTTCCTGGCCAATTCCTCACGAGCTCGCTCGGAGCAGTTCATAAACCTTCGTGAAGTCAGCACACGTTTCAAGTTGCCTCCAGGGGAGTATATTGTTGTGCCTTCCACCTTCGAACCTAACAAGGAAGGAGA
TTTTGTGCTCAGGGTCTTCTCAGAGAAAAAAGCTGGAACTGAGGAGATGGATGATCAGATCCAGGCTAATCTTCCAGAT---GAGGTGGAG------------------------
ATCAGTGTGTCAGAACTGCAGACTATCCTCAACAAAATCATAAGTAAACAT---------------------------------
TCGTGCCGAAGTATGGTGAACCTCATGGATAGGGATGGAAATGGGAAATTAGGACTGGTGGAATTCAACATCCTCTGGAACAAAATTAGGAATTACCTGGCTGTCTTTAGAAAGTTTGATTTAGACAAGTCTGGATGCATGAGTG
CCTATGAAATGCGAATGGCGCTGGAATCTGCAGGCTTTAAACTGAACAAGAAACTGTATGAATTGATCATCACCCGTTATTCTGAGCCTGACTTGGCTGTTGACTTTGATAACTTTGTGTGCTGTTTAGTGCGACTTGAAACCAT
GTTCAGATTTTTTCAAGCTATGGACACAGATAAAGATGGAATTATCACTTTCGATTTATTTAAGTGGTTACAGCTAACTATGTTTGCT

ATGACAGAAGTCCCTGTGTACTGCACGGGGGTGTCTGCCCAAGTGCAAAAACAGAGAGCAAAGGACCTAGGACTGGGCCTCCATGAAAACTCCATCAAGTACCTGGGCCAGGATTACGAGAAGCTACGGTCCCAGTGCCTGCAGT
CTGGGGTCCTTTTTGAGGATGATACTTTCCCCGCTAGTGCCCATTCCCTGGGCTTCAAGGACCTGGGACCCAAATCCTCCAAAACCTATGGCATTAAATGGAAAAGGCCCACGGAACTTTTACCAAACCCTCGGTTTATTGTGGA
TGGGGCCACCCGAACAGACATTTGTCAGGGAGCTCTGGGGGACTGCTGGCTCCTGGCAGCCATCGCCTCCATCACCCTGAACGACACCATCCTGCACCGGGTGGTGCCTCATGGGCAGAGTTTTCAGGAAGGCTATGCTGGCATC
TTTCACTTCCAGTTGTGGCAGTTTGGGGAGTGGATCGATGTGGTTGTGGACGACCTGCTGCCAACCAAGGATGGAAAGCTGCTCTTTGTGCACTCGGCCCAAGGCAATGAGTTCTGGAGTGCCCTGCTTGAGAAAGCCTACGCCA

AGGTGAATGGTAGCTATGAAGCCCTTGCTGGGGGTAGCACCTCAGAGGGATTTGAAGACTTTACCGGAGGTGTTACAGAATGGTTTGACCTCAAGAAACCACCTAGTGATCTCTACTACATCATTCTGAAGGCCCTGGAACGGGG
GTCCATGCTGGGCTGTTCCATCGATCCTGCCCTTTCTTTTCAGCTGGAAGCCGTCACCTTCAAGAAACTGGTGAAGGGCCACGCCTACTCGGTGACCGGAGCCCAGCAGGTGACCCATCGGGGCCGCTCGGTGAACCTAATTCGA
ATGCGGAACCCGTGGGGTGAGGTGGAGTGGACGGGGCCTTGGAGTGACAATTCCTCTGAGTGGAGCCAGGTGGCCCCCTCTGATAGGCAACAGCTCCAGGTCAAGATGGAGGATGGGGAGTTCTGGATGTCCTTCAGTGACTTTC
TTCGGGAGTTCACCCGCCTGGAGATCTGCAACCTGACCCCAGACACCCTCAAGTCCCAAAAGTTCCGCAAATGGAACACAACCCTGTATGATGGCACCTGGAGACGTGGAAGCACAGCGGGTGGCTGCAGAAACTACCCAGCCAC
CTTCTGGGTGAACCCCCAGTTTAAAATCAAGCTGGAGGAGGTGGAT------------
GGTCGAGATGACGGCTGCAGCTTCCTGCTGGCGCTCATGCAGAAGAACCGGCGCCGAGAGCGGCGCTTTGGCCGGGACATGGAGACCATTGGCTTTGCGATCTATGAAGTCCCTGATCACTTGGTGGGCCAGCCTTCTGTGCACC
TGAAGCGGGACTTCTTCCTGTCGCACTCGTCCCTGGCCCGCTCGGAGCAGTTCGTCAACCTGCGGGAGGTCAGCTCGCGCATCTGCCTGCCTCCCGGGGAGTACATCGTGGTGCCCTCCACCTTTGAGCCAAACAAGGAGGGGGA
CTTTGTGCTGCGCTTCTTCTCTGAGAAGAAGGCCGGGACGGAGGAGCTGGATGACAAGATTGAAGCTGACCTGCCCGAT---GAGGTGGAG-----------------------
ATCAGCGTCAAGGAACTGCAGACCATCCTGAACAAAATCATCAGCAAACGA---------------------------------
TCGTGCCGATCTCTCTTAACTCTGGTCCAGCGGGACGGCAATGGGAAACTGGGGCTGGTGGAGTTTAACATCCTCTGGAACCGAATCAAAAATTACCTGAGCATCTTTCGAAAGTATGACCTAGACAAGTCGGGCAGCATGAGTT
CCTACGAGATGCGGATGGCTCTTGAGGCAGCAGGGTACAAGTTGAATAAGAAGCTGCATGGACTAATCATAACTCGCTACTCAGAGGCTGACCTGAACTTGAACTTTGACAGCTTTGTGTGCTGTCTCGTGCGGCTGGAGACCAT
GTTCCGATTTTTCCAGGCCCTGGACACAGACAAAGATGGAATTGTGAATTTTGACTTATTTAGGTGGTTACAGTTGACCATGTTTGCG

 ---------
ATGCCGTGCTTTTTCTCCGGGGTGTCTGCGCAGGTACAGAAGCAGCGGGACAAGGACCTGGGCGTAGGGAAGCACGAGAACGCGGTGAAGTTCCAGGGTCAGGACTACGAGGCACTGCAGGCGGCCTGTCTGCAGAACAGGGTCC
TCTTCGATGACGAAGCCTTTCCCACTTGCGCCACTGCCCTGGGATTCAAGGAACTGGGGCCGGGCTCCCAGAAAACCCGCGGGATCCAGTGGAAAAGACCTATGGAAATTTGTCGCAATCCCCAGTTTATTGTTGACGGAGCGAC
TCGTACGGACATCTGTCAAGGAGCTTTGGGTAAGTGTTGGCTCCCCCTCCACCTTGCAGCAATTACATTATCTCGCAGGTATTTACACAATAATGTTATTGGGGGCAAAAAAACAGGTTTGGAAAAATCTGGAGAGTTTCATGTA
CAATTCTGGCAGTTCGGAGAGTGGGTGGACGTGGTGATTGACGATCGTCTCCCCATGAAAGATGGGAAGCTGATGTTCGTTCACTCGGCCGAGGGGAACGAATTTTGGAGTGCGCTGCTGGAGAAGGCCTACGCAAAGGTAAACG
GTTGCTATGAAGCGCTGTCCGGCGGAAGCACCTCTGAGGGCTTTGAGGATTTCACTGGTGGGGTGACTGAAATGTATGAACTGAAGAAGGCCCCCAAGGACCTGTATGAGATTATCCAGAAAGCCCTGGAAAGGGGCTCCTTGCT
CGGCTGTTCTATCGATATCACAAGTTCTTGCGACATGGAGGCTGTGACATTTAAGAAGTTGGTCAAAGGTCACGCCTACTCCGTCACTGGTGCCAACGAGGTGCAGTACCGAGGACAGCAGGTGAAACTGGTTCGGATCCGAAAC
CCCTGGGGAGAGGTGGAGTGGACCGGCGCCTGGAGCGACGGCTCCGGAGAGTGGAGCGGTGTGAACCCCTCAGTCCGGGAAGAGCTGCGCATTCGAATGGAGGACGGAGAGTTCTGGATGTCCTTCAACGATTTCTTGCGTGAGT
TTAGCCGGCTGGAGATCTGTAACTTGACGCCAGACGCGCTGAAAGACCAGAAGCTGCGCAAATGGAACACGGCGAACTACGACGGACTGTGGAGGCGCGGCAGCACTGCGGGCGGCTGCAGGAATTTCCCAGCCACGTTCTGGAT
TAATCCGCAATTTAAAATCCGATTGGACGAGCTGGAC------------
AGTGGTGAATCGGGGTGCAGCTTCCTGGTTGCGCTGATGCAGAAGGACCGGCGTAAGCAGAGGAAGCTGGGCAAGGACATGGAGACCATCGGCTTTGCGATCTACGAGGTTCCTGAAGAGTGTGCAGGACAGTCCGCGGTCCACC
TGAAGCGGGATTTCTTCTTGACCCACAGCTCCCGCGCCCGTTCGGAGCTCTTCATAAACCTGCGTGAGGTCAGCTCGCGATGTAAGCTCCCGCCGGGCGAGTACATCATCGTGCCCTCCACCTTCGAGCCCAACAAGGAAGGAGA
TTTTGTCCTGAGGGTCTTCTCCGAGAAAACCGCGGATTCTGAGGAGGTGAGCGGGACAATTCATTCCCCCCTCCCCGAA---------AAC-----------------------
ATCAGCGTCCGGGAACTTCAGACCATCCTCAACAGAATAGTCTCCAAACAT---------------------------------
TCCTGCCGCTGTATGGTGAACCTCATGGACAAAGACGGAAATGGAAAACTGGGGCTCGTTGAGTTTAATATCCTTTGGGGAAAAATCCGCAAGTATCTGGGTGTTTTCAGGAAGTTTGATTTGGACAAATCTGGAACAATGAATG
CTTATGAAATGCGTCTTGCTTTGGAGTCTGCAGGT--------------------------------------------------------------------------------------------------------------
----------------------------------------------------------------------------------------

 ------------------------------------------------------------------------------------------------------------------------------------------------
-------------------------------------------------------------------------------------------------------------------------------------------------
-------------------------------------------------------------------------------------------------------------------------------------------------
-
TTTCATTTCCAGTTCTGGCAGTTTGGTGAATGGGTGGAGGTGGTGATTGATGACCGACTCCCGGTGAAAGATGGAAAGCTGGTGTTTGTCCACTCGGCGGCTAGGAACGAGTTCTGGAGCGCCCTCCTGGAGAAAGCCTACGCCA
AATTAAATGGTTGTTATGAAGCCCTCTCGGGAGGTAGTACATCTGAAGGTTTTGAGGATTTCACGGGAGGAGTGACGGAAATGTATGACCTTCGTAAATCGCCCCATGACCTTTACCAAATTATCGAAAAGGCTCTGCAACGTGG
TTCACTCATGGGCTGCTCCATTGACATCACCAGCCAGTTCGACATGGAGGCTGTGACGTTCAAGAAACTGGTCAAAGGTCATGCTTACTCCCTCACTGGTGCCCGGGAGGTACGTGCTCGGGGAGGTTTGGTTAAACTGGTGCGA
TTGCGAAATCCCTGGGGGGAGGTGGAGTGGACTGGAGCCTGGAGTGACAGGTCGAGTGAATGGAACAGTGTGGACTCCTCGGACTGCAGCTCGATGAGAATAAAGCTGGAGGACGGTGAGTTCTGGATGTCCTTTGGTGACTTCC
TCAGAGAATTCAACCGCTTGGAAATTTGCAACCTGACGCCGGATACTCTGAAATCGGAAACGATCTGCAATTGGAGCACCTCTCTGTACGAGGGCTCCTGGAGGCGTGGGAGCACCGCTGGAGGCTGCCGGAATTATCCAGCCAC
GTTCTGGATAAACCCCCAGTTTAAGATTGAACTGAAGGAAGAGGAT------------
GGAATGGAACCCGGCTGCAGCTTCCTGGTCGCCCTGATGCAGAAAGATCGTCGCAAACTCCGCACCGCGGGCAGGGATATGGAGACCATCGGCTTTGCCATCTATGAGGTCCCCCCTGAGTACGCCGGACGCTCAGCTGTTCACC
TGAAGCGAGATTTTTTCCTGACACACGGCTCGCAAGCTCGTTCCGAAATGTTCATCAACCTCCGGGAAGTCAGTACCCGCTTCAAGCTGCCTGCCGGAGAGTACATCATCGTCCCCTCGACCTTTGAACCCAACAAAGAGGCCGA
CTTCTGCCTGAGGGTGTTCTCTGAGAAGCGCTCACACTCTGAGGAA---------------------------------------------------------------------------------------------------
---------------AGGAGG---------------------------------ACATGCAGATCACAG----------------------------------------------------------------------------
-------------------------------------------------------------------------------------------------------------------------------------------------
----------------------------------------------------------------------------------------------------------------------------------------------

------------------
ATGTATTCTGGGGTGGCGGCTCGAGTCTACAAGCAAAGGCAGAAGCAGCTCGGGGCGGGCAACCACGATCAGGCCTTCAAGTACCTCGGGCAGGACTACGAGACGCTACGTGACGACTGCCTTGACCGAGGCGTTCAGTTTGTGG
ACCCCCACTTTCCCGTCGGGCCCTCCATCCTTGGGTTCAAGGAGCTGGGGCCCGGCACCTCAAAAACCTACGGCATCAGGTGGAAGAGGCCGAAGGACATATGTTCAAATCCCCAGTTCATCCTGGATGGGGCAACGCGCACTGA
CATCTGTCAGGGAGGTCTGGGTGACTGCTGGCTTCTTGCGGCCATCGCTTCGCTGACCCTGAACGAGAAGATCCTCCACCGGGTGGTGCCCCACGGGCAGAGCTTCCAGCGGCAGTACTGCGGAATATTCCACTTCCAGTTCTGG
CAGTTTGGTGAGTGGGTGGACGTGGTGATTGACGACCAGCTCCCGGTGAAGGACGGCAAGCTGGTCTTCGTCCATTCCGCCGCGCGCAACGAGTTCTGGAGCGCGCTGCTGGAGAAAGCCTACGCCAAGTTAAGCGGTTGCTATG
AAGCGTTGTCGGGCGGAAGCACGTCGGAAGGCTTCGAAGACTTCACGGGAGGGGTCACGGAAATGTACGACCTCGGCAAATCGCCGCCGGACCTTTACAGAATAATCCAGAAGGCTCTGGAACGTGGCTCTCTCATGGGATGCTC
AATCGATATAACCAGTCAGTTCGACATGGAGGCCGTAACGTTCAAGAAGCTGGTCAAAGGTCACGCCTACTCCCTCACCGGCGCCCAGGAGGTGCCGTGTCGCGGATCGCTGGTCAAACTGGTGCGACTGCGAAACCCCTGGGGG
GAGGTGGAATGGACCGGAGCCTGGAGCGACAGCTCCCGTGAATGGAATCAAGTGGATGGGGCCCAGTGCAGCCAATTGCGGATCCAGCTGGAGGATGGCGAGTTTTGGATGTCCTTCGCTGACTTCCAAAGGGAATTTAACCGTC
TGGAAATCTGCAACCTGACGCCCGACACCCTGAAATCGGACCAGGTCCGCAAATGGAGCACCTCGCTCTACGAGGGCGCCTGGAGGCGCGGGAGCACGGCGGGAGGCTGTCGCAACTACGCAGCTACCTATTGGATAAACCCGCA
GTTCAAGATCCGCCTGAGGGAGGAAGAC---------------
GTCGAGTCGGGCTGCAGTTTCCTGGTGGCGCTGATGCAGAAAGACCGGCGCAAGCTGCGCACCGCGGGCAAGGACATGGAGACCATCGGCTTCGCTGTCTACGAGGTCCCGGATGAGTGCGCCGGACAGTCTGCGGTCCACCTGA
AGCGGGACTTCTTCCTGACGCACGGCTCGAGGGCCCGCTCCGAGATGTTCATCAACCTGCGAGAGGTCAGCAGCCGCTTCAAGCTGCCCGCGGCAGTACAT--------------------------------------------
--------------------------------------------------------------------------------------------------------------------------------------------------
--------------------------------------------------------------------------------------------------------------------------------------------------
--------------------------------------------------------------------------------------------------------------------------------------------------
--------------------------------------------------------------------------------------------------------------------------------------------

---------
ATGCCCATCTACGCCACGGGCATCGCCGCCAAGCTCAGGAGCCAATGGGATCGTGACGACGGCCTGGGTCAGAACCACAAGGCGGTGAAGTTCCTGGGGCAGGACTACGAGTCGCTGAAGGCCAACTGCGCCCAGCGCCGGGGCC
TGTTTGAGGACCCCCTGTTCCCCTGCGCCCCCTCCTCGCTGGGCTTCGACGAGCTGGGGCCCCGCTCAGCCAAGACCTCGGGTGTCCGCTGGATGAGGCCCACGGAGGTTTGCCGGAATCCCAAGTTCATTATGGATGGAGCCAC
CCGCACAGATATCTGCCAGGGGGGTCTTGGTGACTGCTGGCTCCTGGCGGCCATCGCCTCCCTGACGCTGAATGACCAGCTCCTTCACCGGGTGGTGCCTCACGGCCAGAGCTTCGACAGCGGCTACGTCGGCATCTTCCACTTT
CAGTTCTGGCAGTTTGGAGAGTGGGTGGAGGTGGTGATAGACGACCGCCTCCCGGTGAAGGAGGGCAAGCTGCTGTTCGTCCACTCGGCCGAGGGCACCGAATTCTGGAGCGCCCTGATCGAGAAGGCCTACGCCAAGCTGAACG
GCTGCTACGAGGCCCTGTCGGGGGGCAGCACGTCGGAGGGCTTCGAGGACTTCACGGGGGGCGTGACGGAGATGTACGACCTGCCCAAGGCCCCCCAGGACCTCTTCAGCATCGTGGGCCGCGCCATCGAGAGGGGCTCCCTGCT
GGGCTGCTCCATCGACATATCCAGTAACAAGGACATGGAGGCGGTCACCTTTAAAAAGCTGGTGAAGGGACACGCCTACTCTGTGACCGGCGTGAACGAGGTGGTGTACCGAGGGACCCCCACCAAGCTAGTCCGCATCAGGAAC
CCCTGGGGGGAGGTGGAGTGGACCGGCTCCTGGAGCGACAACTCCAGGGAGTGGGACAGTGTGGACCGCAACGTCCGCAGCAAACTGCAGAACCGCAGCGAGGACGGAGAGTTCTGGATGTCGTTCAGCGACTTCCTGCGGGAGT
TCAGCCGGCTGGAGATCTGCAACCTGACCCCTGACGCGCTGCAGAACTGCCAGCTGAAGAAGTGGAGCACCTCGCTCTACCAGGGCGAGTGGCGCCGCGGCAGCACCGCCGGGGGCTGCCGCAACTTCCCAGCGACCTACTGGAT
CAACCCACAGTTCAAGGTAGCTCTGGAGCACCCGGAC------------------
CAATCAGGGTGCAGCTTCCTGGTGGCGCTGATGCAGAAGGACCGGAGGAAGAAGCGGCGGGAGGGGAAGGACATGGAGACCATCGGGTTCGCCATCTATGAGNNNNNNNNNNNNNTTTGCGGGCAAGTCGGCGGTCCACCTGAAGC
GGGACTACTTCCTGACACACAGCTCCAGTGCGCGCTCCGAGCTCTTCATCAACCTGAGGGAGGTGAGCTCGCGCTTCAGCCTGCCCCCGGGGGAGTACATCATCGTGCCCTCCACCTTCGAGCCCCAGAAGGAGGGGGACTTTGT
CCTGCGGGTGTTCTCGGAGAAGCCCACCGACTCCGAGGAGCTTGATGATGAGGTCACAGCAGAACTTCCCCCA---GAGCAGCAG------------------------
ATCAGTGTTAAAGAACTGCAAACAATTCTGAACAGGATCATCGGCAAACAC---------------------------------
GCCTGTCGCAGCATGATAAACCTCATGGACACGGACGGCAGCGGCAAGCTTGGGCTGGCAGAGTTCCACGTGCTGTGGGAGAAGATCAAGAGATACCTGACCATATTCCGCCAGTTTGACATGGACAAGTCGGGCACCATGAACT
CCTACGAGATGCGACGGGCACTGGACTCTGCAGGAATCAAGCTGACCAATCACCTGTTCCAGCTGATCATCCTGCGCTACACGGAGGAAGACCTCACCGTCGACTTTGACAACTTCGTCACGTGCCTGGTTCGGCTGGAGACCAT
GTTCAAGACCTTCAACACAATGGACACCAAGAGAGAAAAATGCATTTCCCTGAACTTCATGCAGTGGATTTCTCTGACCATGTTCGCC

GTCACCCCAATGCGCATCTACGCCACGGGCATGGCCTACAGGCTGAGGAGCCAGTGGGACCGCGAAGAAGGTCTGGGGCAGAACCACAAGGCCGTCAAGTTTCTGGGCCAGGACTACGAGCGCCTGAAAACCCAGAGCCTCCAGA
GTGGGAGGCTGTTCGAGGACAACCTTTTCCCCTGCAGCGCGTCCTCGCTGGGATTCAACGAGCTCGGCCCGAGGTCCTCTAAGACCTCCGGGGTGCGCTGGATAAGGCCTACGGAGATCTGCAAACGTCCCGAGTTCATTGTGGA
CGGAGCCACTCGCACAGACATCTGTCAGGGAGCCCTGGGGGACTGCTGGCTGCTGGCGGCCATCGCTTCGCTCACCTTGAACGACAACCTCCTCCACCGAGTGGTCCCGCACGGCCAGAGCTTTGCGCACGGATACATCGGCATC
TTTCACTTCCAGTTTTGGCAGTTTGGCGAGTGGGTGGATGTGGTGATTGATGACAGGCTGCCGGTGAAGGACGGGAAGTTGCTGTTTGTCCACTCGGCGGAGGGAACCGAGTTCTGGAGCGCGCTGCTGGAAAAGGCTTATGCTA
AGCTGAACGGCTGTTACGAGGCGCTGTCAGGCGGCAGCACGTCGGAGGGCTTCGAGGATTTCACGGGCGGCGTGACGGAGATGTACGAGCTGAGGAAAGCGCCCTCTGACCTCTACAGCATCATCAGCAGGGCGCTGGAGAGAGG
GTCGCTGCTGGGCTGCTCCATCAACATCACCAGCAAATTTGACATGGAGGCTGTGACGTTCAAGAAGCTGGTGAAGGGTCACGCGTACTCTGTCACCGCCGCAGACGAGGTGGTGTACAGAGGAAGTCCAACCAAGCTGGTGCGG
ATCAGAAACCCCTGGGGGGAAGTGGAGTGGACCGGACCCTGGAGTGACAACTCCAGAGAGTGGGACAACGTGGATCGCTCCGTGAGAAGCCGCCTACAAAACCGCAGCGAGGACGGGGAGTTCTGGATGGGATTCAACGACTTCC
TGCGCGAATTCACCACCCTGGAGATCTGCAACCTGACCCCTGATGCCCTGCAGAACGCCCAGCTAAAGAAGTGGAGCACCTCGCTCTACCAGGGCGAGTGGAGGAGGGGGAGCACCGCTGGGGGCTGCAGGAACTATCCAGCCAC
CTTTTGGCTCAACCCACAGTTCAAGCTGGTGCTGAAGAACCCTGAC------------------
CAATCGGAATGCAGCTTCCTGGTCGGCCTCATGCAGAAGGACCGTAGGAAGAAACGGCGAGACGGCGAAGACATGGAGACCATCGGGTTTGCCATCTATGAGGTTCCCCGAGAGATGGCCGGCAGGTCGGGGATCCACCTGAAGC
GAGACTTTTTCCTCACCCATGCTTCTAGCGCTCGCTCCGAGCTCTTCATCAACCTGAGGGAGGTTAGCTCGCGGCTCCAGCTGCCTGCTGGAGAATACGTCATTGTCCCGTCCACGTTCGAGCCACACAAAGAGGCCAACTTCGT
CCTTAGGGTTTTCTCGGAAAAGCCCGCCCAGTCGGAAGAGCTGGATGATGAAGTTGTAGCAGATATTCCGAAG---GAGCGTCAG------------------------
ATCAGCATCACAGAGCTGCAGACCATACTGAACAGGATCATCAGCAAACAT---------------------------------

GCTTGTCGCAGCATGATTAACCTCATGGACGAGGACGGGAGTGGAAAATTGGGTCTGACAGAGTTCCATGTCCTCTGGGAGAAGATTAAACGATATCTGACAATCTTCAGGACCTTCGATTTGGACAAATCGGGCACCATGAGCT
CCTACGAAATGAGGATGGCCCTTGAATCTGCAGGGTTCAAGCTAAACAACAACCTGTTCCAGCTCATCATCCTGCGCTACACGGAGGCCGACATGTCCGTGGACTTTGACAACTTTGTCACATGTCTGGTCAGATTGGAGACCAT
GTACAAGACCTTTAACAGTTTGGACACAGACAAAGACAAAGTCATCAGCCTCAACTTCTTCCAGTGGATCACCCTGACCATGTTTGCC

---------
GTGCCCATTTTTGCCACAGGCATGGCCGCCAAGCTCAGGAATCAGTGGGATCGCGACGGCGGCCTCGGACAGAACGACAACGCGGTGAAGTTTCTGGGTCAGGACTATGAGTTTCTCAAAGCGCAGTGCCTCCAGAGCGGGACGC
TGTTCGAGGACAATCTGTTCCCCTGCGCCACATCGTCTCTGGGATACAACGAGCTCGGCCCCAGATCCTCCAAGACCAGCGGCGTCCGCTGGATGAGGCCCTCGGAGTTGTGCAAGCGGCCGGAGTTCATTGTGGACGGAGCGAC
TCGCACAGACATCTGTCAGGGATCCCTGGGAGACTGCTGGCTGCTGGCGGCCATCGCCTCACTGACGCTGAACGATAACCTCCTCCACAGGGTGGTTCCTCATGGACAGACCTTTCAGCAGGGTTATGCTGGCATCTTCCACTTT
CAGTTCTGGCAGTTTGGAGAGTGGGTGGATGTGGTGATTGATGACCGTCTCCCAGCGAAGAATGGGAAGTTACTGTTTGTCCACTCCGCAGAGGGGACTGAATTCTGGAGCGCTCTGCTGGAAAAGGCCTACGCCAAGCTGAACG
GCTGTTACGAGGCTCTGTCAGGCGGCAGCACATCAGAGGGCTTTGAGGACTTCACCGGAGGCGTGACGGAGATGTTTGAGCTGAAGAACGCCCCGCCGGACCTCTTCAGCATCATCAGCCGAGCCGTCGAGAGAGGATCCCTGCT
CGGCTGCTCTATCGACATCACCAGCTCAGTAGACATGGAGGCCGTCACATTCAAGAAGCTGGTGAAGGGCCACGCCTACTCTCTGACTGCTGTGAACGAGGTGGTGTACAGAGGAAACCCGACCAAGCTGGTCCGCATCAGGAAC
CCCTGGGGGGAGGTGGAATGGACCGGGGCCTGGAGTGACAACTCCAGAGAATGGGACTCCGTGGATAGCTCCGCCAGAAGTCGACTTCAGAACTGCAGCGAAGACGGAGAGTTCTGGATGTCCTTCACTGACTTCCTGCGGGAGT
TCACCCGTCTGGAGATCTGCAACCTGACGGCGGACGCCCTGCAGGACGGCCGGATGAAGAAGTGGAGCACGTCGCTCTTCGGAGGCGAGTGGAGGAGAGGCAGCACAGCTGGAGGCTGCAGGAACTACCCAGCGACGTTTTGGCT
GAATCCTCAGTATAAGATCATGCTGCAGCACCCCGAC------------------
AAATCGGACTGCAGTTTTTTGGTGGCGCTCATGCAAAAGGACCGCAGGAAGAAAAGACAAGAGGGCAAAGACATGGAGACCATTGGGTTTGCTCTATATGAGGTTCCAAAAGAGTTCGTGGGGAGCTCGGGGGTCCACCTGAAGC
GAGACTTCTTCCTCACACACGCCTCCAGCGCTCGCTCGGAGCAGTTCATCAACCTGAGGGAGGTCAGCTCACGCCTGAAGTTGCCCGTCGGGGAGTACATCATCGTCCCCTCCACCTTTGAGCCAAACAATGAGGGCGACTTTGT
CCTGAGAGTCTTCTCCGAGAAACCTGCCGGCTCTGAGGAGCTGGATGACAAAGTGGTTGCAGATCTTCCACCA---GAGAAACAG------------------------
ATCAGCATGACTGAGCTTCAGACTATACTGAACAGGATCATTGGCAAACAC---------------------------------
GCTTGCCGCAGTATGATAAACCTCATGGACGCGGACGGGAGCGGGAAGCTGGGCCTGACAGAGTTCCACGTTCTCTGGGAAAAGATCAAACGATACCTGACTATATTCAGACAGTTTGATTTGGACAAATCGGGCACCATGAACT
CCTATGAGATGCGGATGGCCCTTGAATCTGCAGGCTTCAAACTGAACAACCATCTGTTCCAGCTGATCATCCTGCGGTACACAGAGGAAGACATGTCCATCGACTTTGACAACTTTGTCACCTGTTTGGTCCGGCTGGAGACGAT
GTTCAAAACATTTAAAAACATGGACACGGATGGAGACGGTCAAATATCCCTCAACTTCTTTCAGTGGATTACTCTGACCATGTTTGCC

---------
GCACCCATTTATGCCACCGGCATGGCCTACAAGCTGAGGAGCCAGTGGGATCGTAATGAGGGCCTGGGGCAGAACCACAATGCGGTGAAGTTCTTGGGTCAGGACTATGATTCTCTGAAAGCCCAGTGCCTTCAAAGCGGGGGAT
TGTTTGAGGATAGCCTGTTTCCTGCTGTAGCGTCATCTTTGGGATTCAACGAACTCGGCCCCAGATCGGCAAAGACCAGCGGCGTCCGCTGGATGAGGCCCACGGAGCTCTGCAAGCGGCCCGAATTCATCGTAGATGGAGCTAC
TCGCACTGACATCTGTCAGGGCGCTTTGGGGGACTGCTGGTTGCTGGCAGCCATCGCATCGCTGACCCTAAATGACAACCTCCTCCACAGAGTGGTTCCTCACGGACAGGGTTTCCAGCAGGGATATGCTGGCATCTTTCACTTC
CAGTTCTGGCAGTTTGGTGAGTGGGTGGACGTCGTGATTGACGATCGGCTGCCGGTGAAAGATGGGAAGCTGCTGTTTGTCCACTCAGCAGAGGGAACTGAGTTTTGGAGCGCTCTGATGGAAAAGGCTTACGCCAAGTTGAATG
GCTGTTATGAGGCTCTGTCAGGGGGCAGCACATGTGAGGGTTTTGAAGACTTTACCGGAGGTGTGACGGAGATGTACGATCTGAACAAAGCCCCCTCGGACCTCTTCAGCATCATCCGCAGAGCCGTTGAAAGAGGATCCCTTCT
TGGCTGCTCCATTGACATCACCAGTGCAAATGACATGGAGGCCGTCACCTTCAAAAAGCTGGTGAAGGGACACGCCTACTCCCTGACTGGTGTGGAAGAGGTGTCGTACAGAGGAAACATGACAAGGCTGGTTCGCATCAGGAAC
CCCTGGGGAGAGGTGGAATGGACCGGAGCCTGGAGTGATAACTCCAGAGAGTGGGACTCGATTGATGCGTCTTCCAGAAGTCGTCTACAAAACCGCAGCGAGGATGGTGAATTCTGGATGTCATTTAACGATTTTCTGCGTGAGT
TCTCTCGTCTGGAGATCTGCAACCTGACGGCGGACGCCCTGGAGAGCAGCCAGGTGAAGAAATGGAGCTCCTCGCTTTTTCAGGGGGAGTGGAGGAGAGGCAGCACAGCCGGAGGCTGCAGGAACTACCCAGCAAGTTTTTGGCT
GAATCCTCAGTTCAAGATCATGCTGCAGAACCCAGAC------------------
AGTCCAGACTGCAGCTTTGTGGTTGCCCTCATGCAAAAGGACCGCAGGAAGAAACGGCGAGAGGGCCAAGACATGGAGACCATCGGTTTCGCTCTGTACGAGATTCCAGATGAGTTCAAAGGGAGTTCAGGGGTTCACCTCAAGC
GAGACTTTTTCCTCAGTCATGCCTCCAGCGCTCGCTCAGAAACCTTCATCAACTTGAGGGAGGTCAGCTCACGTCTGAAGCTGCCGAAAGGGGAGTACATAATCGTCCCCTCCACCTTTGAGCCAAACAAAGAGAGCGACTTCGT
CCTCAGGGTGTTCTCCGAGAAGCCTGCAGACTCTGAGGAGCTTGATGATGAAATTAAAGCAGATCTTCCAGCA---GAGCAACAG------------------------
ATCAGTGTCACAGAGCTGCAAACCATACTGAACAGGATCATCAGCAAACAT---------------------------------
GCTTGTCGCAGCATGATTAACCTCATGGATACTGATGGCACTGGGAAGTTGGGCCTGACAGAGTTTCACGTACTCTGGGAAAAGATTAAACGATATCTGACTGTATTCAGGCAGTTTGACGTGGACAAATCGGGCACCATGAGCT
CCTATGAGATGAGAATGGCTCTTGAATCTGCAGGTATCAAGTTGACTAACCACCTGTTCCAGCTGATCATCCTGCGATACACAGAGGCAGACATGACTGTCGACTTTGACAACTTTGTCACCTGTTTGGTCAGACTGGAGACAAT
GTTCAAAACCTTTAAGAACCTGGACACTGATGGAGACGGTCAGATATCTCTAAACTTCTTCCAGTGGATCACCCTGACCATGTTTGCC

---------GCG---
ATTTGTGCGACCGGCATGGCCGCCAAGCTGAGGAGCCAGTGGGATCGCAACGAGGGCCTGGGACAGAACCACATGGCGGTGAAGTACCTGGGTCAGGACTACGAGGGTCTGAAGGCCCAGAGCCTCCAGAGCGGCAGGGCGTTTG
AGGATCACCTGTTCCCTTGTTGCGCGTCGTCTCTGGGATTCAACGAGCTCGGCCCGAGGTCCGCCAAGACGTCTGGAGTGCGCTGGATGAGGCCCGCGGAGATTTGCAAGCGGCCCGAGTTCATCGTGGACGGAGCTACACGCAC
AGACATCTGTCAGGGAGCTCTGGGGGACTGCTGGCTGCTGGCCGCCATCGCCTCGCTGACCTTGAACGACAACCTCCTCCACCGAGTGGTTCCACACGGACAGGGCTTCCAGCAGGGCTACGCCGGCATCTTCCACTTCCAGTTC
TGGCAGTTCGGCGAGTGGGTGGAGGTGGTGATCGACGACCGGCTGCCGGTGAAGGACGGGAAGCTGCTGTTCGTGCACTCGGCCGAGGGCACCGAGTTCTGGAGCGCGCTGCTGGAGAAGGCCTACGCAAAGCTGAACGGCAGTT
ACGAGGCCCTGTCGGGCGGCAGCACGTCCGAGGGCTTCGAGGACTTCACGGGCGGCGTGACGGAGATGTTCGAGCTGAACAAAGCCCCCCCCGACCTCTTCAGCATCATCAGCAGGGCCATAGAGAGGGGGTCCCTGCTGGGCTG
CTCCATAGAGACCACCAGCTCCAGTGACCGGGAGGCCGTCACGTTCAAGAAGCTGGTGAAGGGACACGCTTACTCCGTGACCGGGGTGGAGGAGGTGGTGTACAGAGGGAACACCACCAAGCTGGTTCGCATCAGGAACCCGTGG
GGGGAGGTGGAGTGGACCGGCGCCTGGAGCGACGACTCCAGGGAGTGGGACAATGTGGATCGCTCCGTCCGAGCTCGCCTGCAGAACCGCAGCGAGGACGGCGAGTTCTGGATGTCCTACGCCGACTTCCTGCGCGAGTTCAGCC
GCCTCGAGCTCTGCAACCTGACGGCGGACACCCTGCAGAGCAGCCAGCCCAAGAAGTGGAGCTCCTCGCTCTATCAGGGCGAGTGGAGGAGGGGCAGCACCGCGGGGGGCTGCAGGAACTTCCCAGCGACCTTCTGGCTGAACCC

```
TCAGTTCAAGATCGTGCTGCAGCACCCGGAC------------------
CAGTCCGAATGCAGCTTCCTGGTCGGCCTCATGCAGAAGGACCGCAGGAAGAAGCGGCGGGAGGGACAAGACATGGAGACCATCGGGTTCGCCCTCTACGAGGTTCCAAATGAGTTTGTTGGCAGGTCGGGCGTCCACCTGAAGA
AGGACTTCTTCCTCACCCACGCCTCCAGCGCTCGCTCCGAGCTCTTCATCAACCTGAGGGAGGTCAGCTCGCGGCTGCGGCTGCCGCCCGGCGAGTACGTCATCGTCCCCTCCACCTTCGAGCCGCACAAAGAGGGCGACTTCGT
CCTGAGGGTCTTCTCCGAGAAGCCCGCCGACTCTGAGGAGCTCGATGACGACGTGGCAGCGGAGCTTCCCACT---GAGCAGCAG------------------------
ATCAGCCTCACGGAGCTGCAGACCATACTGAATCGGATCATGAGCAAACAC---------------------------------
TCCTGTCGCAGCATGATCAGCCTCATGGACACGGACGGCAGCGGGAAGTTGGGCCTGAGCGAGTTCCACGTCCTCTGGGAGAAAATCAAACGCTACCTGACCATTTTCAGGCAGTTTGACTTGGACAAGTCGGGCACCATGAGCT
CCTACGAGATGAGGATGGCTCTGGATTCTGCAGGTTTCAAGCTGACCAACCAACTGTTCCAGCTGATCATCCTGCGCTACACGGAGGCCGACATGTCCGTGGACTTCGACAACTTCGTCACCTGCCTGGTCCGGCTGGAGACCAT
GTTCAAAACCTTCCACACCATGGACACGGACAAAGACGGCTTCATGTCCCTCAACTTCAACCAGTGGATCACCCTGACCATGTTTGCC

 ------------
CCCATCTGCGCCACTGGAATGGCTGCGAGGCTGCGGAGTCAGTGGGATCGAGACGCGGGTTTGGGTCAAAACCACAACGCTGTCAAGTTCTTGGGTCAAGATTACGAGACGCTGAGAGCTCAGAGCCAGCAGAGCAGACGACTGT
TTGAGGATCCAATGTTCACCGCCAGCAGCTCTTCTCTGGGCTTCAATGAACTCGGCCCGCGATCCTCCAAAACACAAGGAGTGCGCTGGATGAGACCCAAGGAAATGTGTGCGCGTCCTCAGTTTATCGTGGATGGAGCCACTCG
CACAGACATCTGTCAGGGAGCGCTGGGTGATTGTTGGCTGCTGGCTGCTATCGCGTCCCTGACTCTGAATGATAATCTGCTGCATCGCGTGGTGCCGCACGGACAGGACTTTGACAGCAGATATGCAGGAATATTCCACTTCCAG
TTCTGGCAGTTTGGTGAATGGGTGGACGTGGTGATCGACGACCGGCTGCCGACGAAGGATGGCAAACTGTTATTCGTGCACTCGGCGGAGGGTGGAGAGTTCTGGAGTGCTCTGCTGGAGAAGGCCTACGCCAAGCTGAACGGCT
GTTACGAGGCTCTGTCTGGAGGCAGCACGTGTGAAGGCTTCGAGGATTTCACCGGTGGAGTGACAGAGATGTACGAGTTAAAGAAAGCTCCCGCTGACCTCTTCAGCATTATTGGCCGCGCCATCGAGAGAGGCTCGCTGCTGGG
CTGCTCTATTGATATCACCAGTAAGTTTGACATGGAGGCGGTGACCTTTAAGAAGCTGGTCAAAGGCCACGCATACTCTGTGACCGGAGCAGAAGAGGTGGTCTACAGGGGGAACATGACCAAACTGGTGCGCATCAGGAACCCG
TGGGGGGAAGTGGAGTGGACTGGAGCCTGGAGCGACAACTCTCGCGAATGGGACAATGTGGACCGCTCCGTCCGGGGCCGATTACAGAACCGCAGCGAGGACGGAGAGTTCTGGATGTCGTTCAGTGATTTTCTGCGCGAGTTCA
CCCGTTTGGAAATCTGTAACCTGACGGCGGACGCTCTCCAGGCCAGTCAGGTGAAGAAGTGGAGCACCGCCAACTATAACGGCGAGTGGAGGAGAGGAAGCACTGCAGGTGGCTGCAGGAACTATCCAGCAACATTCTGGATCAA
CCCGCAGTTCAAGGTGGCCCTGAAACACCCGGAT------------------
CAATCAGAGTGCAGCTTCCTGGTGGCGCTGATGCAGAAGGACCGCAGGAAGAAGAGAAGAGAAGGGCAGGACATGGAGACTATCGGATTCGCCATTTATGAGGTGCCAAGAGAGTTTCTAGGTCAGTCTGGTGTTCATCTGAAGC
GGGATTTCTTCCTCACACACGCGTCCAGCGCTCGATCGGAGCTCTTCATTAACCTGCGGGAGGTGAGCTCCCGTTTCCGGCTGCCCGCGGGCGAGTACATCATCGTCCCGTCCACCTTCGAGCCAAACAAAGAGGCCGATTTCGT
GCTCAGAGTTTTCTCAGAGAAGCCCGCCAACTCAGAAGAAATGGATGACAAAGTCATGGCTGAAATTCCTGAG---GAGCGTCAG------------------------
ATCAGTGTGACGGAGCTACAGACGATCCTGAACAGAATCATCGCCAAACAT---------------------------------
TCGTGTCGGAGTATGATCAACCTCATGGACACGGATGGCAGTGGGAAACTCGGACTCGTAGAGTTTCATGTGTTGTGGGAGAAGATTAAACGCTACCTGCAAATCTTCCGCGATCACGATGTGGATAAATCAGGCACTATGAGCT
CGTATGAGATGAGGAAAGCTCTGGAAACTGCAGGATTTAAGCTGAACAATCATTTATTCCAGCTGATCATCCTGCGCTACACTGAGGAGGATCTTTCCGTGGATTTTGACAACTTTGTGTCCTGTTTAGTGCGACTGGAGACCAT
GTTCAAGACGTTCAAGAGTCTGGACACAGACGCTGATGGAGTCATTTCACTCACATTCTTCCAGTGGATCACCCTGACCATGTTTGCC

 ------------
CCAATTTGTGCCATGGGAGTTGCTGCTCGCTTGAGGAGTGAGTGGGACCGAAACGAGGGATTTGGACAGAACCACATGGCCCTGAAATTCTTGGGCCAAGATTTTGAGTCCTTGCGTACTCGCTGCCTTCAGAGTGGACATCTGT
TTGAGGATGATATTTTCCCAGCCCAGCAGTCATCCTTAGGATTCAAGGAGCTGGGCCCCAACTCTAGCAAAACCAGAGGTGTGCGCTGGATGAGGCCAACGGAGTTCTGCTCTGACCCACACTTTATCGTAGATGGGGCTACACG
CACTGACATCTGCCAAGGGGCTCTAGGTGATTGTTGGCTGCTGGCGGCCATTGCGTGCCTTACACTAAACGAACCCTTGCTGCGACGGGTGGTTCCTCATGGCCAGAGCTTCCATCAGCAATATGCCGGAATATTTCACTTCCAG
TTTTGGCAGTTTGGGGACTGGGTGGATGTGGTGATTGATGACCGGCTGCCTGTCAGAGACGGAAAGCTGCTTTTCGTCCACTCAGCTGAAGGATCAGAGTTCTGGAGCGCCCTGGTGGAGAAGGCTTATGCAAAGTTGAATGGCT
GCTATGAGGCTTTATCCGGAGGCAGCACATCTGAAGGCTTTGAGGACTTCACCGGTGGAGTGACTGAGATGTATGAGCTGAAAAACGCTCCACCAAACCTTTTCAGCATCATCACACGAGCTGTGGAGAGAGGCTCCCTCATGGG
CTGCTCTATAGATATTACAAGTTTTTTTGACATGGAGGCCATCACCTTTAAAAAACTAGTGAAAGGCCATGCCTACTCCATTACCGGAGTGGACGAGGTGGAGTACAGACGAAGCCAGACCAAGCTGCTGCGCATCAGAAACCCT
TGGGGAGAGGTGGAGTGGACGGGACCCTGGAGTGATGAGTCAAAGGAATGGCGTGAAATCGATGCATCTGTGAGGTCTCGTCTGCACAACTGTCAAGAAGATGGCGAATTTTGGATGGCGTTCAGTGACTTCAAGCGAGAGTTCA
GCAGACTGGAGATCTGTAACCTGACAGCGGATGCGCTCCAGAGCCGAGAGGTGAAAAAGTGGAACTCGTCGCTGTATCCTGGTGAATGGAGAAGAGGATCCACTGCTGGCGGATGCAGGAATTATCCAGCAACATTTTGGATCAA
TCCCCAGTTTAAAATAGTGCTGAAGGAACCAGAT------------------
CAAGAAGGCTGCACTTTTCTAGTGGCTCTGATGCAGAAGAATCGCAGACAGTTACGGAGGGAGGGCAAAGACATGGAGACCATTGGATTTGCCATTTATGAGGTCCCTAAAGAGTATCTTGGTCGGGTTGGTGTGCATCTGAAGC
GGGATTTTTTCCTCAAGCACACGTCCAGCGCTCGGTCAGAGCTCTTCATCAATCTTCGAGAAGTGAGCTCACGCTTCTCACTGCCAGCAGGAGAGTATATCATCGTCCCGTCCACTTTTGAGCCCCAGAAAGAAGGAGACTTTGT
GCTCAGAGTGTTCTCGGAGAAGGCGACCGACTCGCAGGAGTTAGATGATGAAATTTCAGCAGACGTCCCGGAG---GAGGTGGAC------------------------
ATCAATGTGGCAAAACTTGAGATGATTCTGAATCGGGTTGTCAGCAAACAT---------------------------------
ACTTGCAGAGGCATGATAAATCTCATGGATACTACTGGGACTGGAAAGCTGGGACTGACAGACTTCCATGTGCTCTGGGAGAAGTTCAAACGATATCTTGCAGTCTTTAGGGAGTTTGACATAGACAAGTCTGGCACAATGAGCT
CTTATGAAATGCGTCTCGCTTTAGAATCAGCAGGCTTTAAGTTGACCAACAACCTGTTCCAGCTGATAATCCTGCGCTATGCAAAGTCAGACCTCAATGTGGACTTTGATAACTTTGTGGCCTGTCTGATCCGCCTTGAGACTAT
GTTCAAAACGTTTAAAACATTGGACGCTGATGAAGATGGACTTGTATCCTTCAGCTTTGCCCAGTGGATCACCCTCACTATGTTCACA;

END;
```

```
BEGIN TREES;

	TREE tree = (((((XENOPUS_TROPICALIS_ENSXETG00000023299,XENOPUS_LAEVIS_NM_001093915),XENOPUS_LAEVIS_NM_001087016),((((OVIS_ARIES_NM_001127267,SUS_SCROFA_ENSSSCG00000012999),((CERATOTHERIUM_SIMUM_XM_004437595,EQUUS_CABALLUS_XM_001917043),(HOMO_SAPIENS_NM_001198869,MUS_MUSCULUS_ENSMUSG00000024942))),SARCOPHILUS_HARRISII_ENSSHAG00000003383),((PELODISCUS_SINENSIS_ENSPSIG00000008053,ANOLIS_CAROLINENSIS_ENSACAG00000016579),GALLUS_GALLUS_NM_001044672))),LATIMERIA_CHALUMNAE_ENSLACG00000018300),(((GASTEROSTEUS_ACULEATUS_ENSGACG00000018991,((OREOCHROMIS_NILOTICUS_ENSONIG00000013071,XIPHOPHORUS_MACULATUS_ENSXMAG00000008475),TAKIFUGU_RUBRIPES_ENSTRUG00000008157)),(DANIO_RERIO_ENSDARG00000045199,DANIO_RERIO_ENSDARG00000055338)),GADUS_MORHUA_ENSGMOG00000012126)),(SCYLIORHINUS_CANICULA_CONTIG00627,CHILOSCYLLIUM_PUNCTATUM_CONTIG39834));

END;
```

## (*e*), *CAPN2*

```
#NEXUS
BEGIN TAXA;

	DIMENSIONS NTAX = 25;

	TAXLABELS

		'ANOLIS_CAROLINENSIS_ENSACAG00000003199' 'GALLUS_GALLUS_ENSGALG00000009360' 'PELODISCUS_SINENSIS_ENSPSIG00000012616' 'HOMO_SAPIENS_BC021303' 'CERATOTHERIUM_SIMUM_XP_004439596' 'OVIS_ARIES_NP_001106288' 'MUS_MUSCULUS_ENSMUSG00000026509' 'ORNITHORHYNCHUS_ANATINUS_ENSOANG00000001450' 'MONODELPHIS_DOMESTICA_ENSMODG00000005094' 'XENOPUS_LAEVIS_NP_001083713' 'XENOPUS_TROPICALIS_NM_001005446' 'GASTEROSTEUS_ACULEATUS_ENSGACG00000004767' 'GASTEROSTEUS_ACULEATUS_ENSGACG00000019985' 'DANIO_RERIO_ENSDARG00000034211' 'DANIO_RERIO_ENSDARG00000090014' 'TAKIFUGU_RUBRIPES_ENSTRUG00000012768' 'OREOCHROMIS_NILOTICUS_ENSONIG00000007266' 'GASTEROSTEUS_ACULEATUS_ENSGACG00000019944' 'DANIO_RERIO_ENSDARG00000091699' 'LEPISOSTEUS_OCULATUS_GENSCAN00000007636' 'DANIO_RERIO_ENSDARG00000055592' 'RAJA_ERINACEA_TRANSCRIPTOMECONTIG62832' 'SCYLIORHINUS_CANICULA_TRANSCRIPTOMECONTIG67723' 'CHILOSCYLLIUM_PUNCTATUM_CONTIG44567' 'CALLORHINCHUS_MILII_TRANSCRIPTOMECONTIG17290' ;

END;



BEGIN CHARACTERS;

	DIMENSIONS NCHAR = 2040;
	FORMAT
	DATATYPE = DNA
GAP=-

		MISSING=?

		NOLABELS

	;
```

MATRIX

ATGTCTGGGATCGCAGCGAAAGTGGCCAAAGAGCGGGAGGTGGCCCAAGGGCTGGGCTCCAACCAGAAGGCCGTGAAGTACCTCCAGCAGGACTTCGGGGCGCTGCGGGCGGAGTGCCTGGAGGCCGGGGGGCTCTTCCAGGACC
CCTGCTTCCCCGCCGCCGCCCCCGCGCTGGGCTTCAAGGAGCTGGGACCCCACGCCGCCAAGACCAAGGGCATCGTCTGGAAGAGGCCCACGGAGTTATGTTCCAATCCTCAGTTTATTGTTGGAGGGGCCACCCGGACTGATAT
CTGCCAGGGAGCTTTAGGAGACTGTTGGCTTCTGGCTGCCATTGCTTCTCTCACGCTAAATGAAGAAATTCTGGCTAGAGTTGTTCCCAGTGATCAAAGCTTTCAAGACAGATATGCAGGGATTTTCCATTTCCAGTTCTGGCAG
TATGGAGAATGGACAGATGTGGTGGTTGATGATCGGCTGCCAACCAAAAATGGGGAGCTCCTTTTTGTTCACTCAGCAGAAGGGACTGAGTTCTGGAGTGCTCTGTTGGAGAAGGCCTATGCCAAGGTGAATGGGTCGTATGAGG
CTCTCTCTGGAGGAAGCACTACTGAGGGCTTTGAGGACTTCACTGGTGGAATTGCAGAGTGGTATGAACTGAGAAAAGCTCCACCAAATCTGTTCAGAATCATCCAGAAAGCCCTTCAAAAAGGTTCTCTCCTTGGATGTTCTAT
CGATATCACAAGTGCTGCTGAGACGGAGGCAGTCACAACACAGAAGCTGGTTAAAGGGCATGCATACTCTGTAACGGGTGCAGAAGAGGTCAGTTTCCGAGGCAGTCTGCAAAAATTGATCAGAATCCGAAATCCCTGGGGAGAA
GTAGAATGGACTGGGAAATGGAACGATAATTGCCCAAGCTGGAGTAATGTT---
CCTGAAAAACATGAAGATGGAGAATTTTGGATGTCATTTGACGATTTTCTGAGACATTACTCACGCCTTGAGATCTGTAACTTGACACCAGACACCCTGACCAATGACAACTACAAGAAATGGAGTCTCAGTTTGATGGATGGGA
ACTGGAGAAAAGGATCCACTGCTGGTGGATGCAGAAATTATCCTGATTCATTTTGGATGAACCCACAATATTTGATTAAACTTGAGGAAGAAGATGAAGATCCGGATGATCCTGAGAAGGGCTGCACCTTCTTGATTGGACTGAT
TCAGAAACACCGCCGGAAGCAGCGGAAAATGGGGGAGGATATGCATACCATTGGCTTTGCCATCTATGAGGTACCTCCACAGTTTTCAGGCCAAACCAACGTCCATCTGAGCAAAAACTTCTTTCTAACAAATCGAGCGAAAGAA
CGATCAAACACCTTCATCAACCTCCGAGAAGTGTTAAATCGCTTCAAGCTTCCCTCAGGGGAGTATATTATTGTGCCATCAACATTCGAACCCAACAAGAATGGGGATTTCTGCCTCCGAGTCTTCTCCGAAAAGAGTGCGGATT
CACAG---------------------------------------
GTCACTGAAGATGAAATTGAACCCAGTTTTAAGAAACTATTTGGACAATTAGCTGGAGGGGATGCTGAGATCTCAGCCTTTGAACTGTGTGGTATTCTGAAAAAAGTGATGGCAAAA---------
ATTGGCTTCAGCATTGAAACATGTAAAATCATGGTTGATCTATTAGATACAGATGGAAGTGGTAAACTGGGGCTGAAGGAGTTTCATATACTT---ACAAAGATTCAG---------
AAAATTTATCGGGAAATGGATGTTAGTTCTGGAACC------------------------------------GCAGGCTTTAAGCTGGATTGTCAGTTGCACCAAGTCATTGTGGCTCGTTTTGCTGATCTGATC------
ATTGATTTTGACAATTTTGTTCGATGCTTGATTCGGCTGGAAACCTTGTTCAAGATATTTAAGAAATTGGACACTGAGAAAACGGGAACAGTACAGATGAATATCATC

ATGGCGGGGATGGCGGCGGCGCTGGCGAAGGAGCGGGCGGCGGCGGCGGGCGCGGGGCGGCACGGGCAGGCCGTGCCCTACGTGGGGCAGGACTTCGGGGCGCTGCGGCGGGAGTGCCTGCAGGGAGGCCGCCTCTTCCACGACC
CGTCCTTCCCCGCCGGCCCCGCCGCTCTCGGGTACCGGGAGCTGGGGCCCAACTCCTACAAAACGAAGGGCGTCGTCTGGTGTAGACCTACGGAGCTGTGTTCCTGTCCGCGGTTCATCGCCGGCGGAGCCACGCGCACCGACAT
CTGCCAAGGAGCCCTGGGGGACTGCTGGCTCTTGGCAGCTATTGCCTCCCTCACTCTGAATGAAGAAATTCTGGCCCGTGTTGTTCCCAGAGACCAGAGCTTCCAGGATGAATATGCAGGAATCTTCCACTTCCAGTTCTGGCAG
TACGGAGAGTGGGTGGACGTCGTGGTGGATGACCGGCTGCCCACCAAGAACGGGGAGCTGCTCTTCGTGCACTCGGCAGAGGGCAGCGAGTTCTGGAGCGCACTGCTGGAGAAGGCCTACGCCAAGCTGAATGGATCATACGAGG
CTCTCTCTGGTGGCACCACCACTGAAGGCTTTGAGGACTTCACTGGTGGGATTGCAGAATGGTATGAGTTGCAGAAGGCACCACCCAACCTCTTCAAAATCATTCAGAAAGCACTGCAGAAAGGCTCTCTCCTTGGCTGCTCCAT
CGATATCACCAGTGCAGCTGAGACAGAGGCAGTCACGTCGCAGAAGCTGGTGAAGGGACACGCGTACTCCGTCACCGGGGCAGAGGAGGTGAACTTCCGTGGAAGTATCCAGAAGCTCATCAGAATCCGAAATCCCTGGGGGGAA
GTGGAGTGGACAGGGAAATGGAATGACAACTGCCCAAACTGGAGTGGTGTT---
CCAGAGAGACATGAGGATGGGGAGTTTTGGATGGCCTTCAATGACTTCCTGAGGCACTACTCCCGCCTGGAAATCTGCAATCTGACTCCAGACACTCTGGCAAGTGACAGGTACAAGAAGTGGAGCCTGCTGAAGCTGGATGGGA
ACTGGCGGCGAGGAGCTACTGCGGGGGGCTGCAGGAATTACCCAAACACTTTCTGGACGAATCCACAGTATTTAATCAAGCTGGAGGAAGAAGATGAGGATCCTGATGATCCTGAGGGGGGCTGCACATTTCTCATTGGCCTGAT
TCAGAAGCATCGGAGGAAGCAGAGGAAGATGGGAGAGGACATGCATACCATTGGCTTTGCAATTTATGAGGCAACTCCTTCTTTCTCTGGCCAGACAAATATTCATCTGAGCAAAAATTTTTTCCTGACTAACAAAGCAAGAGAA
AAATCCAACACCTTCATCAACCTCCGGGAGGTGCTGAACCGGTTCAAGCTCCCTGCAGGAGAATACATCATCGTGCCCTCTACCTTTGAGCCCAACTTGAATGGAGACTTCTGCCTCCGAGTCTTCTCGGAAAAAAATGCAAACT
CAACG---------------------------------------
ATCGATGAAGATGACATTGAACCTAGCTTCAAAAAGCTTTTTGGGCAGTTGGCAGGAAGCGATGCAGAGATCTCTGCCTTCGAGCTGCGCAACATCCTGAATAAAATCCTGGCTAAG---------
ATTGGCTTTAGCATTGAGACGTGCAAAATAATGGTTGACCTGCTAGATAACGATGGGAGTGGAAAACTGGGGCTGAAGGAGTTCCACACGCTC---ACAAAGATTCAG---------
AAAATTTACAGGGAAATTGACGTGCGATCTGGAACC------------------------------------GCAGGGTTCAAGTTGAACTGCCAGTTACATCAGATCATTGTGGCTCGGTTTGCTGATCTCATC------
ATTGACTTTGATAATTTTGTCCGGTGCTTGATTCGGCTGGAAACCTTGTTCAAAATGTTTAGAAAACTGGACACCGAGAAAACTGGAACAATAGAGTTGAACGTCATT

 ATGTCAGGGATGGCAGCGAAGTTGGCGAAGGAGCGGGAAACAGCCCGGGGCTTGGGCTCCAACGCGAAGGCGTTTAAATACCTCAGCCAGGACTTCGAGGCGCTGCGGGACGAGTGC---------------------
CAGGACCCCGCCTTCCCCGCCGTCCCCTCTTCCCTGGGCTACCAGGAGCTGGGGCCCACCTCGTACAAAACTCAGGGCATCACGTGGAAGAGACCAACGGAGCTGTGTTCTAATCCCCAGTTTATTGTTGGAGGTGCCACCCGAA
CAGATATCTGCCAAGGAGCTTTGGGTGACTGCTGGTTGCTGGCAGCCATTGCGTCTCTCACCCTGAATGAAGAGATTCTGGCCAGAGTTGTTCCCAAAGATCAGAGCTTCCAGGACAGATATGCAGGAATCTTCCACTTCCAGTT
CTGGCAGTATGGGGAGTGGGTGGATGTTGTGGTTGATGACAAGCTCCCTACCAAAAATGGAGAGCTGCTCTTCGTTCACTCAGCAGAAGGGAGCGAGTTTTGGAGTGCCCTGCTGGAGAAGGCCTATGCCAAGGTAAATGGCTCA
TACGAGGCACTGTCTGGTGGAAGCACCACTGAAGGCTTTGAGGATTTCACCGGTGGAATTGCAGAATGGTATGAGCTGCAAAAGGCACCAGCTGACCTATTCAAAATCATCCAGAAAGCTCTTCTAAAAGGCTCCCTTCTTGGAT
GCTCTATTGATATCACAAGTGCCGCTGAGACGGAGGCAGTCACTTCCCAGAAGCTGGTCAAAGGCCACGCATACTCCGTTACAGGAGCTGAAGAGGTGAATTGCCGAGGAAGCATGCAAAAGCTGATCCGAATCCGAAATCCCTG
GGGGGAAGTGGAGTGGACTGGAAAATGGAATGATAACTGTTCAAACTGGAATAGTGTT---
CCCGAGAGACATGAAGATGGAGAATTTTGGATGTCATTCAGTGATTTCCTGAGACATTATTCCCGCCTTGAGATCTGCAACCTGACTCCGGACACACTGGCAAATGAAAAATACAAGAAGTGGAGCCTGACCAAGCTAGACGGGA

ATTGGAGACGTGGCTCCACTGCTGGGGGTTGCCGGAATTACCCAAATACTTTCTGGATGAATCCACAGTATTTGATTAAGCTGGAAGAAGAAGATGGGGATCTGGATGATCCTGAGAAGGGCTGCACCTTCTTGGTTGGCCTGAT
GCAGAAGCACCGGAGGAGGCAGAGGAAGATGGGGGAGGATATGCACACCATTGGCTTTGGAATCTATGAGCTCCCCTCACAGTTTTCTGGACAGACAAACGTTCATCTGAGCAAAAACTTCTTCCTGACGAACAGAGCCAAAGAA
AAATCAAACACTTTCATCAATCTCCGAGAGGTACTAAACCGATTCAAACTCCCTCCAGGAGACTATGTCATTGTGCCATCCACTTTTGAACCCAACAAGGATGGGGATTTCTGCCTCCGTGTCTTCTCTGAAAAGAATGCAGATT
CACAG----------------------------------------------------
ATCAGTGAAGATGACATTGAACCCAGTTTCAAAAAGCTTTTTGGGCAGTTGGCTGGCAAGGATGCAGAGATCTCTGCTTTTGAATTATGCAATATCCTGAGAAAAATCATGGCTAAA---------
ATTGGCTTCAGTATTGAGACATGTAAAATAATGGTCGATCTGTTAGATTCTGATGGAAGTGGTAAACTGGGACTGAAGGAATTCCACATACTT---ACAAAGATACAG---------
AAAATCTACAGGGAAATAGACGTTCGCTCTGGTACC-----------------------------------------------GCAGGGTTCAAACTGACTTGCCAGCTGCACCAAGTCATTGTGGCTCGTTTTGCAGATCTTATC------
ATCGACTTCGATAATTTTGTCAGGTGCTTGATTCGGCTGGAAACTTTGTTCAAAATATTCAAGAAACTGGATGTTGAGAAAAGTGGAACAGTGGAGTTGAAT------

ATGGCGGGCATCGCGGCCAAGCTGGCGAAGGACCGGGAGGCGGCCGAGGGGCTGGGCTCCCACGAGAGGGCCATCAAGTACCTCAACCAGGACTACGAGGCGCTGCGGAACGAGTGCCTGGAGGCCGGGACGCTCTTCCAGGACC
CGTCCTTCCCGGCCATCCCCTCGGCCCTGGGCTTCAAGGAGTTGGGGCCCTACTCCAGCAAAACCCGGGGCATCGAGTGGAAGCGCCCCACGGAGATCTGCGCTGACCCCCAGTTTATCATTGGAGGAGCCACCCGCACAGACAT
CTGCCAAGGAGCCCTAGGTGACTGCTGGCTGCTGGCAGCCATTGCCTCCCTCACCTTGAATGAAGAAATCCTGGCTCGAGTCGTCCCCCTAAACCAGAGCTTCCAGGAAAACTATGCAGGGATCTTTCACTTCCAGTTCTGGCAA
TACGGCGAGTGGGTGGAGGTGGTGGTGGATGACAGGCTGCCCACCAAGGACGGGGAGCTGCTCTTTGTGCATTCAGCCGAAGGGAGCGAGTTCTGGAGCGCCCTGCTGGAGAAGGCATACGCCAAGATCAACGGATGCTATGAAG
CACTATCAGGGGGTGCCACCACTGAGGGCTTCGAAGACTTCACCGGAGGCATTGCTGAGTGGTATGAGTTGAAGAAGCCCCCTCCCAACCTGTTCAAGATCATCCAGAAAGCTCTGCAAAAAGGCTCTCTCCTTGGCTGCTCCAT
CGACATCACCAGCGCCGCGGACTCGGAGGCCATCACGTTTCAGAAGCTGGTGAAGGGGCACGCGTACTCGGTCACCGGAGCCGAGGAGGTTGAAAGTAACGGAAGCCTACAGAAACTGATCCGCATCCGAAATCCCTGGGGAGAA
GTGGAGTGGACAGGGCGGTGGAATGACAACTGCCCAAGCTGGAACACTATA---
CCAGAGCGGCATGAAGATGGAGAATTCTGGATGTCTTTCAGTGACTTCCTGAGGCACTATTCCCGCCTGGAGATCTGTAACCTGACCCCAGACACTCTCACCAGCGATACCTACAAGAAGTGGAAACTCACCAAAATGGATGGGA
ACTGGAGGCGGGGCTCCACCGCGGGAGGTTGCAGGAACTACCCGAACACATTCTGGATGAACCCTCAGTACCTGATCAAGCTGGAGGAGGAGGATGAGGACGAGGAGGATGGGGAGAGCGGCTGCACCTTCCTGGTGGGGCTCAT
TCAGAAGCACCGACGGCGGCAGAGGAAGATGGGCGAGGACATGCACACCATCGGCTTTGGCATCTATGAGGTTCCAGAGGAGTTAAGTGGGCAGACCAACATCCACCTCAGCAAAAACTTCTTCCTGACGAATCGCGCCAGGGAG
CGCTCAGACACCTTCATCAACCTCCGGGAGGTGCTCAACCGCTTCAAGCTGCCGCCAGGAGAGTACATTCTCGTGCCTTCCACCTTCGAACCCAACAAGGATGGGGATTTCTGCATCCGGGTCTTTTCTGAAAAGAAAGCTGACT
ACCAA----------------------------------------------------
ATCAGCGAGGATGACATTGATGATGGATTCAGGAGACTGTTTGCCCAGTTGGCAGGAGAGGATGCGGAGATCTCTGCCTTTGAGCTGCAGACCATCCTGAGAAGGGTTCTAGCAAAG---------
ATCGGCTTCAGCATCGAGACATGCAAAATTATGGTTGACATGCTAGATTCGGACGGGAGTGGCAAGCTGGGGCTGAAGGAGTTCTACATTCTC---ACGAAGATTCAA---------
AAAATTTACCGAGAAATCGACGTTAGGTCTGGTACC-----------------------------------------------GCAGGTTTCAAGATGCCCTGTCAACTCCACCAAGTCATCGTTGCTCGGTTTGCAGATCTCATC------
ATCGATTTTGATAATTTTGTTCGGTGTTTGGTTCGGCTGGAAACGCTATTCAAGATATTTAAGCAGCTGGATCCCGAGAATACTGGAACAATAGAGCTCGACGTACTT

ATGGCGGGCATCGCGGCCAAGCTGGCGAAGGACCGGGAGGCGGCCGAGGGGCTGGGCTCGCACGAGAGGGCCATCAAGTACCTCAACCAGGACTACGAGGCGCTGCGGAACGAGTGCCTGGAGGCCGGGGCGCTCTTCCAGGACC
CCTCCTTCCCGGCCGGCCCTTCCTCCCTGGGCTTCAAGGAGTTGGGACCCTACTCCAGCAAGACGCAGGGCATCCAGTGGAAGCGGCCCACGGAGATCTGCGATGACCCCCAATTTATTGTTGGAGGAGCCACCCGCACAGATAT
CTGCCAAGGAGCCCTGGGTGACTGCTGGCTGCTGGCAGCCATCGCCTCCCTCACCTTGAATGAGGAGATCCTGGCTCGAGTCGTCCCCCTGGACCAGAGCTTCCAGGAGAACTATGCAGGGATCTTCCGCTTCCAGTTCTGGCAG
TACGGCGAGTGGGTGGAGGTGGTCGTGGACGACAGGCTGCCCACCAAGGACGGGGAGCTGCTCTTCGTGCATTCGGCAGAGGGGAGCGAGTTCTGGAGCGCGCTGCTGGAGAAGGCATACGCCAAGATCAATGGGTGTTATGAAG
CACTCTCGGGGGGCGCCACCACCGAGGGCTTCGAGGACTTCACCGGAGGCATCGCTGAGTGGTACGAGTTGAGGAAGGCCCCTTCCAACCTGTTCAAGATCATCCAGAAGGCACTGCAGAAAGGCTCTCTCCTTGGCTGCTCCAT
CGATATCACCAGCGCTGCAGACTCGGAGGCCATCACATTCCAGAAGCTGGTGAAGGGGCATGCGTACTCCGTCACCGGAGCCGAGGAGGTTGAAAGCAACGGAAGCCTGCAGAAGCTGATCCGCATCCGAAATCCCTGGGGAGAG
GTGGAGTGGACCGGGAGGTGGAATGACAACTGCCCAAACTGGAATACTGTC---
CCAGAGCGGCATGAAGATGGAGAGTTCTGGATGTCTTTCAGCGACTTCCTGAGGCACTATTCTCGCCTGGAGATCTGCAACCTGACCCCCGACACCCTCACCTGCGATTCCTACAAGAAGTGGAAACTCACCAAGATGGATGGGA
ACTGGAGGCGGGGCTCCACGGCCGGAGGCTGCAGGAACTACCCGAATACCTTCTGGATGAACCCTCAGTACCTGATCAAGCTGGAGGAGGAGGATGAGGACCAGGAGGACGGGGAGAGCGGCTGCACCTTCCTGGTGGGCCTCAT
CCAGAAGCACCGGCGGCGCCAGAGGAAGATGGGCGAGGACATGCACACCATTGGCTTTGGCATCTATGAGGTTCCGGAGGAGTTAAATGGACAGACCAACGTCCACCTCAGCAAAAACTTCTTCCTGACGCACAGAGCCAGGGAG
CGGTCGGACACCTTCATCAACCTGCGAGAGGTGCTCAACCGCTTCAAGCTGCCCCCGGGCGAGTACATCCTCGTGCCATCCACCTTTGAGCCCCACAAGGATGGGGACTTCTGCATCCGGGTCTTTTCTGAAAAGAAAGCTGACT
ACCAA----------------------------------------------------
GTCAGCGAGGAAGACATTGATGATGGATTCAGGAGGCTGTTTGCCCAGCTGGCGGGGGAGGATGCAGAGATTTCTGCCTTCGAGTTGCAGACCATCTTGAGACGAGTTCTAGCAAAG---------
ATCGGCTTCAGCATCGAGACCTGCAAAATCATGGTTGACATGCTAGATTCGGATGGGACTGGTAAGCTGGGGCTGAAGGAGTTCTACATTCTT---ACGAAGATTCAG---------
AAAATTTACCGGGAAATCGACGTGAGGTCTGGTACC-----------------------------------------------GCAGGTTTCAAGCTGCCCTGTCAACTCCACCAAGTCATCGTGGCTCGGTTCGCAGATCTCATC------
ATCGATTTTGATAATTTCGTTCGGTGTTTGGTTCGACTGGAGACGCTATTCAAGATATTTAAGCAGCTGGACCCCGAGAATACTGGCACGATAGAGCTCGACGTCCTT

ATGGCGGGCATCGCGGCCAAGCTGGCCAAGGACCGGGAGGCGGCCGAGGGGCTGGGCTCGCACGAGAGGGCGGTCAAGTACCTCAACCAGGACTACGCGGCGCTGCGGGACGAGTGCCTGGAGGCCGGGGCGCTGTTCCAGGACC
CCTCCTTCCCGGCCCTGCCTTCCTCCCTGGGCTTCAAGGAGTTGGGACCCTACTCCAGCAAGACCCGGGGCATCGAGTGGAAGCGGCCCACGGAGATCTGCGATGATCCCCAATTTATTACTGGAGGAGCCACTCGCACAGACAT
CTGCCAAGGAGCCCTAGGTGACTGCTGGCTGCTGGCCGCCATTGCCTCCCTCACTTTGAATGAAGAAATTCTGGCTCGAGTCGTCCCCCTGGACCAGAGCTTCCAGGAGAACTATGCAGGGATCTTCCACTTCCAGTTCTGGCAG

TACGGCGAGTGGGTGGAGGTGGTGGTGGACGACAGGCTGCCCACCAAGGACGGGGAGCTGCTCTTCGTGCACTCAGCAGAGGGCAGCGAGTTCTGGAGTGCGCTGCTGGAGAAGGCCTACGCCAAGATCAATGGGTGTTATGAAG
CACTTTCGGGGGGCGCCACCACCGAGGGCTTCGAGGACTTCACTGGAGGCATCGCCGAGTGGTATGAGTTGAGGAAGGCCCCTCCCAACCTGTTCAGGATCATCCAGAAGGCTCTGCAGAAAGGCTCTCTCCTGGGCTGCTCCAT
CGATATCACCAGCGCGGCGGACTCGGAGGCCATCACGTTCCAGAAGCTGGTGAAGGGGCACGCGTACTCGGTGACCGGCGCCGAGGAGGTGGAAAGCAGAGGAAGCCTGCAGAAGCTGATCCGCATCCGGAATCCCTGGGGAGAG
GTGGAGTGGACCGGGCAGTGGAATGACAACTGCCCAAACTGGAACACAGTT---
CCCGAGCGGCACGAGGATGGGGAATTCTGGATGTCTTTCAGCGACTTCCTGAGACACTATTCCCGCCTGGAGATCTGCAACCTAACCCCTGATACACTCACCAGTGACACCTACAAGAAGTGGAAACTCACCAAGATGGATGGGA
ACTGGAGGCGAGGCTCGACCGCGGGCGGCTGCAGGAACTACCCAAACACGTTCTGGATGAACCCTCAGTACCTGATCAAGCTGGAGGAAGAGGACGAGGATCAGGAGGACGGGGAGAGCGGCTGCACCTTCCTGGTGGGCCTCAT
CCAGAAGCACCGCCGGCGGCAGAGAAAGATGGGCGAGGACATGCACACCATTGGCTTTGGCATCTATGAGGTTCCAGAGGAGTTTACTGGGCAGACCAACATCCACCTCAGCAAAAAATTCTTCCTGACAACCAGAGCGCGGGAG
CGGTCGGACACCTTCATCAACCTGCGGGAGGTGCTCAACCGCTTCAAGCTGCCCCCCGGGGAGTACATCGTGGTGCCCTCCACCTTCGAGCCCAACAAGGACGGCGACTTCTGCATCCGGGTCTTTTCTGAGAAGAAAGCCGACT
ACCAA---------------------------------------
ATCAGCGAGGACGACATCGATGATGGATTCAGGAGACTGTTTGCCCAGCTAGCTGGAGAGGATGCAGAGATTTCTGCCTTTGAGTTGCAGACAATCCTGAGACGAGTGCTAGCAAAG---------
ATCGGCTTCAGTATTGAGACCTGCAAGATCATGGTGGACATGCTCGACTCGGATGGGAGTGGCAAACTGGGTCTGAAGGAGTTCTACATTCTC---ACGAAGATTCAG---------
AAAATTTACCGGGAAATCGATGTGCGGTCTGGAACC------------------------------------GCAGGTTTCAAGATGCCCTGTCAGCTCCACCAAGTGATCGTCGCCCGGTTTGCAGATCTCATC------
ATCGATTTTGACAATTTTGTCCGGTGTTTGATTCGACTGGAGACACTATTCAGGATATTTAAGCAGCTGGACCCGGAGAATACTGGAATGATCCAGCTCGATGTACTT

ATGGCGGGCATCGCGATAAAGCTGGCCAAGGACCGCGAAGCGGCCGAGGGGCTGGGGTCGCATGAGAGAGCCATCAAGTACCTCAACCAGGACTACGAGACGCTGCGGAACGAGTGCCTGGAGGCCGGGGCGCTCTTCCAGGATC
CTTCCTTCCCCGCCCTGCCGTCATCCTTGGGCTATAAGGAGTTGGGGCCCTACTCCAGCAAAACTCGGGGCATCGAATGGAAGCGGCCCACGGAGATCTGCGCTGACCCCCAGTTCATTATTGGAGGAGCCACCCGCACAGACAT
CTGCCAAGGAGCCCTGGGGGACTGCTGGCTGCTGGCTGCCATCGCCTCCCTCACCTTGAATGAGGAAATCCTGGCTCGGGTTGTGCCTCCAGATCAGAGCTTCCAGGAAAACTATGCAGGCATCTTCCACTTCCAGTTCTGGCAA
TATGGCGAGTGGGTGGAGGTGGTGGTGGACGACAGGCTGCCCACTAAGGACGGGGAGCTGCTCTTTGTGCATTCAGCTGAAGGGAGTGAGTTCTGGAGTGCCCTTCTGGAGAAAGCCTATGCCAAGATCAATGGGTGCTATGAAG
CACTCTCAGGGGGTGCCACCACGGAAGGCTTTGAAGACTTCACAGGAGGCATTGCTGAGTGGTATGAGCTGAGGAAGCCTCCTCCCAACTTGTTCAAGATCATCCAGAAGGCTTTGGAGAAAGGTTCTCTGCTTGGCTGCTCTAT
CGATATCACCAGCGCTGCAGATTCTGAGGCCGTTACGTACCAGAAGTTGGTGAAAGGACATGCGTACTCTGTCACCGGAGCCGAGGAGGTTGAAAGTTCAGGAAGCCTGCAGAAATTGATCCGCATCAGGAATCCCTGGGGACAA
GTAGAGTGGACCGGGAAGTGGAATGACAATTGCCCCAGCTGGAACACGGTT---
CCAGAACGGCAGGAGGACGGAGAATTCTGGATGTCCTTCAGTGACTTCCTGAGACACTACTCTCGCCTGGAGATCTGCAACCTGACCCCGGACACCCTCACCTGTGACTCCTATAAGAAGTGGAAACTCACCAAGATGGATGGGA
ACTGGAGGCGAGGCTCCACCGCAGGGGGCTGCAGGAATTACCCAAATACCTTCTGGATGAACCCTCAGTACCTAATTAAGCTGGAGGAAGAAGACGAAGATGAGGAGGATGGGGAGAGAGGCTGTACCTTCCTGGTGGGTCTCAT
CCAGAAGCACCGGCGACGGCAGAGGAAGATGGGCGAGGACATGCACACCATTGGCTTCGGCATCTATGAGGTTCCAGAGGAGCTAACAGGGCAGACCAACATCCACCTCGGCAAAAACTTTTTCCTCACAACCCGAGCCAGGGAG
CGGTCAGATACCTTCATTAACCTCCGCGAGGTCCTCAACCGCTTCAAGCTGCCCCCGGGAGAGTATGTCCTCGTTCCCTCCACCTTCGAACCCCACAAGGATGGCGATTTCTGCATCCGAGTCTTCTCGGAGAAGAAGGCTGACT
ACCAA---------------------------------------
GCCAATGAGGAGGACATTGACGATGGATTCCGAAGGCTCTTTGTTCAGCTGGCTGGAGAGGATGCAGAGATCTCTGCCTTTGAGCTGCAGACCATCTTAAGAAGAGTTCTAGCCAAA---------
ATCGGCTTCAGCATCGAGACCTGTAAGATCATGGTGGACATGCTGGATGAAGATGGGAGTGGCAAGCTTGGCCTGAAGGAGTTCTACATCCTC---ACGAAGATTCAG---------
AAAATCTACCGGGAAATCGATGTGAGGTCTGGAACC------------------------------------GCAGGTTTCAAGCTGCCCTGTCAACTTCATCAAGTCATCGTTGCCCGGTTTGCAGACCTAATC------
ATCGACTTTGACAATTTTGTGCGGTGTTTGGTCCGTCTGGAAACGCTATTCAAGATATTCAAGCAGCTAGACCCTGAGAACACTGGAACGATACAGCTCAACGTACTC

ATGTCAGGAATTGCAGCCAAAGTAGCCAAGGACAGAGAAGCGGCGCAAGGGCTCGGTACCCACCAGAAGGCCATCAAGTACCTCAACCAGGACTATGAATCTCTGAAGAGCGACTGCCTGCAGTCGGGGAAGCTCTTCCAAGATC
CCTCTTTTCCTGCCATCACCTCTTCCCTGGGATTCAAGGAACTGGGACCGAACTCCAGCAAAACTCAAGGCATCGTCTGGAAGAGACCGGCGGAGATTTGTTCCAATCCCCAGTTTATCGTTGGAGGCGCTACCCGCACAGACAT
CTGCCAAGGGGCCCTGGGTGACTGCTGGCTGCTGGCAGCTATTGCCTCCCTCACCCTAAACGAAGAAGTCTTAAGTCGGGTGGTCCCCGAGAATCAGAGTTTCCAAGACAACTACGCTGGGATCTTCCACTTTCAGTTCTGGCAG
TATGGAGAGTGGGTGGAAGTGGTTGTGGACGACCGGCTGCCGACCAAGAATGGGGAGCTGCTGTTTGTGCATTCGGCTGAGGGGAGCGAGTTCTGGAGCGCCTTGCTGGAGAAGGCCTATGCCAAGGTCAACGGCTGCTACGAAG
CTCTGTCCGGGGGTGCCACCACAGAAGGCTTTGAGGACTTCACGGGTGGGATTGCGGAGTGGTATGAGTTGAAGAAAGCCCCAGACAACCTGTTCAAGATCATCCAGAAGGCTCTGCAGAAAGGGTCCCTCCTCGGCTGCTCCAT
TGATATTACAAGTGCTTCAGAGACAGAGGCAGTTACATTCCAGAAGTTGGTGAAAGGACATGCATATTCTGTCACAGGAGCAGAAGAGGTGAGCTGCAGAGGGAGCATGGAGAAGCTGATCCGCATCCGGAACCCCTGGGGGGAA
GTGGAGTGGACCGGGAAGTGGAATGACAACTGCCCAAACTGGAATAGCATC---
CCTGAACGACATGAAGATGGAGAATTCTGGATGTCATTCAGGGACTTCTTGAGGCACTATTCCCGCTTGGAGATCTGCAACCTGACTCCTGACACTCTAACCAGCGAGACCTTCAAGAAGTGGAGCCTTTCAAAGCTGGATGGGA
ACTGGAGGCGGGGTTCCACTGCCGGGGGCTGCAGAAACTATCCGAACACTTTCTGGATGAACCCTCAGTACCTGATCAAGCTGGAGGAAGAGGATGAAGATCCTGAGGACGTGGAGAAGGGATGCACTTTCCTAGTGGGCCTCAT
CCAGAAGCACCGACGGCGGCAGAGGAAGATGGGACAGGACATGCACACCATCGGCTTCAGCATCTATGAGGTTCCTAAGCAGTTTTCCGGGCAGACTAACATTCACCTCAACAAAAACTTCTTCTTGACGAATAAAGCGAGAGAA
CGGTCGGACACTTTCATCAACCTCCGTGAGGTGCTCAACCGCTTCAAGCTGCCGCCGGGAGAATACATTATTGTCCCATCCACTTTCGAACCCAACAAGGATGGAGACTTCTGCCTTCGGGTCTTCTCTGAAAAGAATGCCAACT
CCCAG---------------------------------------
GTTGATGAAGACAGCATCGACGCAGGCTTCAAGAGACTGTTTGGGCAACTGGCGGGAGAGGATGCGGAGATCTCTGCCTTTGAGCTGCAGACCATCCTGAAAAAAGTCCTAGCCAAG---------
ATCGGCTTCAGCATTGAAACTTGTAAAATCATGGTTGATCTGCTAGACACTGATGGGAGTGGCAAACTGGGTCTGAAGGAGTTCTTCATTCTC---ACAAAAATTCAG---------
AAAATTTACCGGGAAATAGATGCTAACTCAGGAACC------------------------------------GCAGGCTACAAGCTACCCCGCCAGCTCCATCAAGTCATCATTGCGCGATTTGCAGACCTCATC------
ATAGATTTTGATAACTTCGTTCGGTGTTTGGTTCGCCTGGAAACTTTGTTCAAAGTATTTAAGCAGCTGGACACCCAGAAAACAGGAACAATAGAGCTGAATGTCCTT

ATGTCGGGCATCGCAGCCAAAGTGGTGAGAGATCGAGAGGCGGCGGCCGGCCTGGGCACTCATGAGCAGGCGGTCAAGTACCTCAACCAGGACTATGAGACACTTCTCAGCGAGTGCCTTGAGGAGGGCAAACTCTTCCAGGACC
CTCACTTCCCAGCAATTGGATCTTCCCTGGGGTTCAAGGAACTGGGGCCGTACTCGAGCAAAACTCAAGGCATCGTTTGGAAGAGACCCACGGAGATCTGCTCTGATCCTCAGTTTATTGTTGAAGGAGCAACTCGCACAGACAT
CTGCCAAGGAGCCCTGGGTGACTGCTGGTTGCTGGCAGCCATCGCCTCTCTCACCCTGAATGAAGAAATCCTGGCCAGGGTGGTCCCTAAAAACCAAAGCTTCCAGGACAACTATGCTGGGATCTTCCATTTTCAGTTCTGGCAG
TATGGCGAATGGGTTGATGTGGTGGTGGATGACAGGCTGCCCACAAAGAACGATGAGCTGCTTTTTGTCCACTCGGCAGAGGGAAGTGAATTCTGGAGTGCCCTCCTGGAGAAAGCCTATGCCAAGGTCAATGGATGTTATGAAG
CTCTCTCAGGAGGTGCCACCACCGAGGGCTTTGAGGACTTCACTGGTGGGATAGCCGAGTGGTACGAGCTGAAGAAAGCACCTGACAACTTGTTTATCATCATCCAGAAGGCCCTGCAGAAGGGTTCTCTCCTGGGCTGTTCCAT
TGATATCACCAGTGCCGCCGATTCCGAGGCTGTGACTTTCCAGAAGCTGGTGAAGGGGCACGCGTACTCGGTCACCGGAGCTGAAGAGGTGGACTTCAGAGGAAGCCTGCAGAAGCTGATTCGGATACGCAACCCCTGGGGAGAA
GTAGAATGGACCGGAAAGTGGAGTGACAGCTGCCCTAACTGGAACAGCATT---
CCAGATCGGTCTGAAGATGGAGAATTCTGGATGTCTTTCAGCGACTTCCTGAGGCACTATTCCCGCCTCGAGATCTGCAACCTAACCCCCGACACGCTCATCAGTGATGACTACAAAAAGTGGAGACTCACCAAGATGGATGGGA
CCTGGCGGCGGGGGTCTACTGCAGGGGGCTGCAGGAACTACCCAAACACTTTCTGGATGAACCCTCAATACATGATCAAGCTGGAGGAGGAAGATGAAGATGACGATGGCTCCGAGCCCGGCTGCACCTTCCTCGTGGGCCTTAT
CCAGAAACACCGGCGACGCCAAAGGAAAATGGGCGAGGATATGCACACTATCGGCTTTAGCATCTATGAGGTTCCCGAGGAGTTTTCTGGTCGAACCAACCTTCACCTCAACAAAAACTTCTTTCTGACAACCAGAGCAAGAGAG
AGATCAGACACATTCATCAATCTCCGGGAAGTGCTGAACCGCTTCAAGCTGCCTGCTGGAGAATATGTCATCGTGCCTTCTACTTTTGAACCCCACAAGAATGGGGATTTCTGCCTCCGTGTCTTTTCAGAGACGAAAACTGACT
CTCAA---------------------------------------
GTTGATGAAGATGGCATTGAGGACAGCTTCAGGAGACTGTTTGCTCAACTGGCAGGGGAGGACGCAGAGATTTCTGCCTTTGAGCTTCAGACTATCCTGAGAAGAGTCCTGGCAAAG---------
ATCGGCTTCAGCATTGAGACTTGCAAGATCATGGTTGACATGCTGGATTTTGACGGGAGTGGAAAACTAGGTCTGAAGGAGTTCTACCTTCTT---TCCAAAATACAG---------
AAAATTTACAAGGAGATGGACGTTAGGTCCGGCACC------------------------------------GCAGGGTTCAAGCTGCCCTATCAGCTCCATCAGGTCATCGTGGCCCGGTTCGCGGATCTCATC------
ATCGACTTCGACAACTTTGTCCGGTGTTTGGTCCGACTGGAGACGCTGTTCAAAGTGTTTAAGCAGCTGGATCCCGAGAACACCGGGACGATAGAGCTCGATGTCCTT

ATGAGCGGGGTCGCCGACAGACTTGCTAAGGAGCGGGCAAGGGCCAGTGGCTTTGGTACCAATGAAAAGGCTCTGCCCTACCTCAACCAGGACTTCGAGGCTCTGCGCAATGAGTGTCTCCAGTCCGGCTCCCTGTTTAAGGACC
CCAGCTTCCCCGCCATCCACTCCTCCTTGGGATTCAAAGAGCTGGGTCAGTACTCAAGCAAAACCCAAGGGGTGGTGTGGAAAAGACCCACGGACCTGTGCTCAAGCCCTCAGTTCATCGCAGGTGGAGCAACTCGGACTGATAT
CTGCCAGGGAGCCCTGGGTGACTGCTGGCTTCTTGCAGCCATCGCCTCCCTCACCCTCAATGAACAGATTCTTTCCCGGGTCGTTCCAATGGACCAGAGCTTTCAGGAGAAATATGCAGGAATATTCCACTTCCAGTTCTGGCAA
TATGGCGAGTGGGTGGACGTTGTCATTGATGACCGTTTGCCAACCAAAGATGGGAAGCTGCTTTTTGTACACTCAGCCGAGGGGACTGAATTCTGGAGCGCGCTCATGGAGAAGGCTTATGCCAAACTGAACGGATCCTATGAAG
CTCTCTCAGGTGGGACCACCACTGAAGGATTTGAAGATTTCACAGGTGGACTTGCAGAGTGGTATGAACTGAAGAAGGCCCCGTCTAATCTGTTCAAGATTATCCAGAAAGCTCTGAAATCTGGATCTCTCCTGGGCTGCTCTAT
AGATATCACCAGTGCAGCCGAGACAGAGGCCATCACTTTCCAGAAGCTTGTGAAGGGTCACGCCTACTCTGTGACCGCTGCAGAAGAGGTGCAATATCGAGGGAGCCTGGAAAAGCTGATCCGAATCAGAAATCCTTGGGGGGAG
GTGGAATGGACTGGAGCCTGGAGTGACAATTCCAGTGAGTGGAATTCTGTG---
CCGGAGAGATGTGACGATGGAGAGTTTTGGATGTCCTTTAATGACTTCTTGAGAAACTATTCAAGACTGGAGATCTGCAATCTGACCCCGGACACTCTGGCCTCTGACAAATACAGTAAATGGAGCCTGACCAAGATGGACGGCT
CATGGAGGCGAGGCTCTACAGCCGGTGGATGCAGGAATTTCCCAGAAACCTTCTGGATGAACCCCCAGTACATGATTAAGCTGGATGAGGCAGATGATGATCCCGATGATAATGAGGAAGGCTGCACCTTCATTGTTGGTCTGAT
ACAAAAGAACCGCAGGAATCAGAGAAAGATGGGAGAAGACATGCACACCATTGGCTTTGCCATGTACGAGGTGCCGCCACAGCTTACTGGCCAGAAAGACGTTCGCCTGAGCCGAGATTACATTCTAAGGAACAAGATTCGGGAA
AAATCAGAGACCTACATAAACCTGCGTGAGGTTCTGAACAGATTTCGGCTGCCCGCAGGGGAGTATGTCATTGTGCCGTCCACGTTCGATCCCCACAAAGTAGGCGACTTTTGTCTGCGGGTGTTCTCTGAAAAGAATGCCGAGA
CAAAA---------------------------------------
GTTAATGAGAATGCCATCGATCCCACCTTCAAAAGAATGTTCCTGGCGTTGGCAGGAGATGACAAAGAGATCTCTCCGCATGAACTCTTTAACATCCTACAGAAAGTCATATCAAAA---------
ATAGGGTTCAGCATGGAGACCTGTAGGACCATTGTTGACCTGCTGGATTCTGATGGTTCAGGGAAACTTGGCCTCAAGGAGTTCAATATCCTC---ACAAAAATACTC---------
AAAATATACAGTAGCGTGGATAAGCGTTCGGGTACC------------------------------------GCAGGGTTTAAGGTGAACGCCAAGATTATTGAGCTGCTGGTGGCTCGCTTCGCAGATCAAAAC------
ATTGACTTTGATAACTTTGTGAGATGCCTCCTGCGCCTGGAGACCATGTTCAAGATCTTCAATAAGCTGGACACGGAGAAGACTGGAGTCGTGCCGCTCAAGGTGATC

ATGAGCGGGGTGGCTGATAGGCTGGCCAAGGAGCGGGCACTGGCAAGTGGCCTTGGCACCAATGAGAAGGCGCTGCCCTACCTGAGCCAGGACTATGAGGCGCTGCGCAATGAGTGTCTCCAGTCTGGGACCCTGTTTAAGGACC
TCAGCTTCCCGGCCATCCACTCCTCCCTGGGGTTCAAAGAGCTGGGGCAGCATTCGGCCAAATGCCAAGGGGTGGTATGGAAGAGACCCAAGGACCTGTGCCCAGACCCTCAGTTCATCGCAGGAGGAGCGACTCGCACTGATAT
CTGCCAAGGAGCCCTGGGTGACTGCTGGTTACTTGCGGCCATTGCCTCCCTCACCCTGAATGAGCAGATTCTGTCCCGGGTTGTTCCAAAGGATCAGAGCTTCCAAGAGAACTATGCCGGAATATTCCACTTCCAGTTCTGGCAG
TACGGTGAGTGGGTGGATGTTGTCATTGATGATCGTCTGCCAACCAAGGATGGGAAGCTGCTTTTTGTGCATTCAGCGGAGGGGAGTGAGTTCTGGAGCGCACTCATGGAGAAGGCTTATGCCAAACTGAACGGATCCTATGAAG
CTCTCTCGGGTGGGACCACCACCGAAGGGTTTGAAGATTTCACAGGTGGACTTGCAGAGTGGTATGAACTGAGGAAGGCCCCACCCAATCTGTTCAAGATTATCCAGAAAGCTCTGAGGACTGGATCTCTGCTGGGCTGCTCTAT
AGATATCACAAGTGCAGCGGAGACAGAAGCCATCACATTCCAGAAGCTCGTGAAGGGCCATGCCTACTCTGTGACTGCAGCAGAAGAGGTGCAATATCGAGGGAACCTGGAAAAGCTGATCAGAATCAGAAATCCCTGGGGGGAA
GTGGAATGGACCGGGGCCTGGAGTGACAATTCCAGCGAGTGGAATTCTGTGGATCCGGATAGATGTGACGATGGAGAGTTTTGGATGTCCTTCAATGACTTCTTGAGAAACTATTCAAGACTGGAGATCTGCAATCTGACCCCGG
ACACTCTGGCCTCTGACAAATACAGTAAGTGGAGTCTGACCAAGATGGACGGCACGTGGAGGAGAGGCTCGACAGCCGGCGGATGCAGGAATTTCCCAGAAACCTTCTGGATGAACCCCCAGTATGTGATTAAGCTGGAAGAAGC
AGATGATGATCCTGATGATAATGAGGAAGGCTGCACCTTTATTGTCGGTCTGATACAGAAGAACCGCAGGAAGCAGAGGAAGATGGGAGAGGACATGCACACCATTGGCTTTGCCATCTATGAGGTGCCGCCACAGCTCACTGGC
CAGAAGGACATTCGCCTGAGCCGAGATTACATTCTAAGGAACAAGATTCGGGAAAAATCCGACACCTATATAAACCTGCGCGAGGTGCTGAACAGATTCCGGCTGCCCCCAGGGGAGTACGTCATTCTGCCTTCCACATTCGAAC
CCCACAAAGTCGGAGACTTCTGCCTGCGGGTCTTCTCTGAAAAGAACGCCGAGTCCAAAGTTCCTGATGATGAAATCAAGGCTGACATTGATGAGGACGTCAATGAAGATGCCATCGACCCCAACTTCAAAAGGATGTTCCTGGC

GCTGGCAGGAGATGACAAAGAGATCTCTCCGCATGAACTCTTTAACATCCTACAGAAGGTGATATCCAAGCGAGAAGACATAGGGTTCAGCATAGAGACCTGTAGGACAATTGTTGACCTGCTGGATTCTGATGGTACAGGGAAA
CTTGGCCTTAAGGAGTTCAAAATCCTATGGACAAAAATACTGAAGTACCAGAAAATATACAGCAGCGTGGATAGGCACTCCGGCACTATTAATTCCTATGAGATGCGGGGAGCCCTGGAAGCTGCAGGGATTAAGGTGAACTCCA
CCATTAATGAGCTGCTGGTGGCTCGCTTCGCGGATGAAGACCATACCATTGACTTTGATAACTTTGTGAGATGCCTTCTGCGCCTGGAGATCATGTTCAAGATCTTCAACCAGATGGACACTGAGAAGACCGGAGTGGTGACTCT
GAAGATGGAC

ATGACGTCCACTGCAGAGCGACTGGCCCGTCAGAAGGAGAGGGCAGAAGGCATCGGCACCAACCAGCAAGCGGTGAAGTTCTCCCAACAGGATTATGAGACGCTGCGCCAGGAGTGCCTGAAGCGCGGACGTCTATTTGAGGATG
ACTGCTTTCCAGCTGAGCCTAAATCCCTGGGCTTCAACGAGCTGGGACCGTACTCATCCAAAACCAGGGGCGTTGTTTGGAAAAGGCCAACGGACCTGTGCTCTGAGCCACAGTTCATTAAGGACGGGGCGACGACCACAGACAT
TTGCCAAGGAGCTCTGGGTGACTGCTGGCTACTGGCTGCAATAGCCTCTCTGACTCTTGACCAGCGGATCCTGGCTCGCGTGGTGCCCCCCGGACAGAGCTTTTCCGATGATTATGCCGGGATATTTCACTTCCAGTTCTGGCAG
TTTGGCGAGTGGGTGGATGTGGTGGTTGACGACCGCTTGCCCACCAAAGATGGAGAGCTGCTGTTTGTTCACTCAGCGGAGGGCTCGGAGTTTTGGAGCGCGCTGTTGGAGAAGGCCTATGCCAAAATAAATGGATGCTATGAGG
CTCTTTCCGGAGGCAGCACCACTGAGGGATTTGAGGACTTCACGGGAGGCATTGCCGAGAGACATGAGCTGAAGAACGCAGATCCTCGCCTCTTCAAAATCATTAAGAAGGCCTTGGAGAGAGGCTCCCTTCTGGGATGCTCCAT
TGATATCACCAGCGCATCTGACTCAGAAGCTGTCACATATCGCAAGTTGGTGAAAGGCCACGCCTACTCGGTGACGGGAGCGGAGCAGGTGGATTACAGAGGAGACCAGGTGCAGCTGATCAGGATTAGGAACCCGTGGGGTCAG
GTGGAGTGGAATGGAGCTTGGAGTGACCGGTCGTCCGAGTGGAGATACGTG---
GACGGGCGCTGTGAGGATGGGGAGTTCTGGATGTCGTTTTCCGACTTCCTGCGGCAATATTCTCGCCTTGAGATCTGCAACCTCACCCCTGATGCGCTCACGGGCGATGAGTTCAAGAAATGGGCAGAGTCAGAGTTTGAAGAAA
CGTGGAGACGAGGCGTTTCAGCTGGTGGCTGCAGAAACTTTCCAGATTCCTTCTGGATGAATCCTCAGTTTGTCATAAAGCTGGAGGAGGTGGACGATGATCCTGAGGACGGTGAGGAGGGCTGCACCTTCATCGTGGGCTTGAT
GCAGAAGAACCGGCGGCGTATGAGGAAAATGGGGCAGGACATGGAGACCATCGGCTTTGCCATATATGAGCTGCCTGATGAGTACTCCGGCCAAAGGCAAGTGCACTTGAAGAGAAACTTCTTTGTGCGCAACTGTTCAGCAGCG
CGCTCCGAGACCTTCATCAACCTGAGGGAAGTGAGCAACCACTTCTGTCTCCCCCCGGGAGAATACCTCATCGTCCCCTCCACCTTTGAGCCCAACAAGAACGGGGACTTTTATGTGCGGGTGTTCTCTGAGAAACAGACAGCTT
TCCAA---------------------------------------
ATCGATGAAGATGACATCAGTGACAGATTCAAGAGACTGTTTGGACAGCTCGCTGGACATGATGTGGAGATTTCTGCATTCGAGCTGCAGAAAATCCTCAACCGAGTGGTGGTGAAG---------
ATCGGGTTTAGCCTGACAACCTGCCACCACATGGTCAATCTGCTTGACAAAGATGGAAGCGGAAAGCTGGGACTGGTGGAGTTTAAGATTCTG---ACAAAGATTGAG---------
GATGTGTACAAAGAGAAAGACGCACAGAGTGGCTGT------------------------------------ATTGGTTTCTCTCTCAACACCGCTCTGCATCAGATAATTCTGGCCCGCTACAGCGAGCTCACC------
ATTGACTTTGACAGCTTTGTGTGCAGCATGATCCGCTTGGAGTCACTCTTCAACATCTTCAACACCCTGGAAAAGAACGGGTCCGGTGAGATAGAATTGGGTCTGTGC

TTAAATGGTAAAATAATAAAAACAAAACACCGCAGAGACAAGGACCGGGGCATCGGCTCCTTCGGCCGGGCGCTGAAGTACCTGAACCAGGATTACGAGGTTTTGCGGCGGCGCTGCTCGGAGAGTGGCCGCCTGTTCCAGGATG
AGACCTTCCCGGCTCACGGTTCCTCTCTGGGCTTCAGGGAGCTGGGCCCCGGATCCCCCAAGACCCGCGGGGTGTCCTGGGAGAGGCCGACGGAGTTGACCTCTGACCCTGAGTTCATTGTCTCCGGAGCCTCAAGGACGGACAT
CTGTCAGGGAGCTTTGGGGGACTGCTGGCTGCTCGCCGCCATCGCCTCTCTGACCCTGAATGAGGAAGTCCTGGCTCGAGTCGTTCCGCATGGGCAAAGCTTCAAAGGAGAGTACGCAGGCATCTTCCACTTCCAGTTCTGGCAG
TTTGGGGAGTGGGTGGACGTTGTCATTGACGACCGTCTGCCAGTCAAAGACGGAGAGCTGATGTTCGTGCACTCAGCGGAGGGGAGGGAGTTCTGGAGCGCCCTGCTGGAGAAGGCCTACGCCAAGATGAACGGGTGCTACGAGG
CTCTGTCTGGAGGATCCACCACGGAGGGCTTTGAGGACTTCACTGGCGGGATCGCTGAGGTCCATGACCTCAACAGACCGGATCCACATCTGTTCCACATCATCCACATGGGTCAGAACCGTGGGTCTCTGATGGGCTGCTCCAT
AGACATCACAAGCTCATCAGACTCTGAGGCAGTCACCTCTCAGAAGCTGGTGAAAGGCCACGCCTACTCTGTGACGGGGACGGCTCAGGTTGAGTACCGAGGAAACATGGAGAAGCTGATTCGGATCAGGAACCCTTGGGGTCAG
GTGGAGTGGACGGGAGCCTGGAGTGACGATTCAGCTCAGTGGCGTCAGATC---
GACGAGCGCTCAGAGGACGGAGAGTTCTGGATGTCCTTCAATGACTTCCTGCGTCACTACTCACGGCTGGAGATCTGTAATCTGACGCCAGACGCCCTGAGCGACGACTCCGTCTCCAGGTGGGCTCTGTCCAAGTTCGACGGCA
GCTGGAGGAGAGGGTCCACCGCCGGGGGCTGCAGGAACTTTCCCAACACGTTCTGGACAAACCCCCAGTTCGTGATCCGGCTGGACGAGGAGGACGACGACCCCGACGACGGCGAGAGCGGCTGCAGCTTTGTAGTCGGTCTGAT
CCAGAAGAACCGCAGATCCATGAGGAAGATGGGAGAGGACATGCACACGGTGGGATTCGCCATCTACGAGGTCCCTGAGGAGTTCTGTGGCCAGAGGAACGTCCACCTGAACAGGAATTTCTTCCTGAGCAACGCATCCGCTGCT
CGCTCCGAGACTTTCATCAACCTGCGAGAGGTCTGCAGCCGCTTCTGCCTCCCGCCGGGGGAGTACCTGATCGTCCCGTCCACCTTCGAGGCCAACAAGGACGGGGATTTCTGCGTCCGCGTCTTCTCAGAGAAACAAGTTAATT
CTCAG---------------------------------------
ATTGATGAAGGCGACGTAGACGACCGGTTTCGGAGTCTGTTTGGACAACTGGCGGGCGAGGACTGTGAGATCTCAACCCATGAGCTTCAGAGGATCCTCAACAAAGTGGTGACCAGA---------
ATCGGCTTCAGCATCTCGACGTGTCGCAACATGATCAACATGCTGGACAAGGACGGCAGCGGTAAACTGGGTCTGCTGGAGTTTAAAGTGCTG---ACCAAAATCGAG---------
AAAATCTACCGTCAGAAGGACGTCAACTCTGGGACG------------------------------------GCTGGCTTTAGTCTGAACAACCCGCTCCATCAGGTCCTGGTGGCTCGTTACAGTGACCTCACC------
ATTGACTTCGACAACTTTGTTAGCTGTTTGGTTCGTCTGGAGACAATGTTCAACACTTTCAATGTGCTGGACAAAGATAATTCGGGAACTGTCGAGTTCAACCTGCTC

ATGTCAGGAATCGCTACAAAACTCCAAAAGAACCGGGCTCGAGCGGCGGGCATCGGTACCAACGCTCAAGCTGCCAAATTCCTCAACCAGGACTACGAAGCCCTCAAACGAGAGTGTCTGGAGTCCGGACGCCTGTTTCAGGATG
GCATGTTTGAAGCCAATGTGTCTGCCCTTGGGTTTAAGGAACTGGGACCAAACTCGTCTAAAGTGCGAGGGGTTGAGTGGCTGAGACCCAAACAACTGACGTCCAACCCAACATTCATTAGTGGGGGAGCGACTAGAACAGACAT
CTGCCAGGGTGCTTTGGGTGACTGTTGGCTTCTAGCGGCCATCGCTTCTCTTACTCTCAACCAGGATGTGTTTGCTCGTGTCGTGCCGGCTGGTCAGAGTTTTGATGGCGACTATGCTGGGATATTTCATTTTCAGTTCTGGCAG
TTTGGTGAATGGGTGGATGTGGTCATTGACGACCGACTGCCGGCCAGAAAAGGAGAGCTGCTATTCGTCCACTCGGCCGAGGGCTCTGAGTTTTGGAGCGCTCTATTAGAAAAAGCCTATGCAAAGCTGAATGGCTGCTATGAAG
CTCTTTCTGGCGGCACCACCACTGAAGGTTTTGAGGATTTCACTGGAGGCATCGCTGAGGTTCACGAACTGCCCAAAGCGGGTCCAAACCTCTTCAAAACTATCCAGAAGGCTTTGAGTTGGGGCTCATTGCTGGGCTGCTCCAT
AGATATCACAAGCTCATCTGACTCAGAGGCCATTACTAGTCAGAAACTGGTGAAAGGACACGCCTACTCGGTTACAGGAGCTGAGGAGGTTGAATACAGGGGTGACTTAACCAAACTGATTCGCATCAGGAACCCCTGGGGGCAG
GTGGAGTGGACGGGACCCTGGAGTGATGGGTCATCAGAATGGCGTCAGATC---

GACAGCAAAGCCGAAGATGGAGAATTCTGGATGTCTTTCTCGGACTTCATGCGTCATTACTCGCGGGTGGAGATCTGCAACCTGACACCCGATGCTCTGACAGACGAAAGTGTTAATAAATGGGCTCTGTCCAAGTTTGATGGCA
ACTGGAGGAATGGATCAACTGCTGGAGGCTGCAGGAATTACCCCAACTCGTTCTGGATGAATCCTCAGTTTTTGATCAAGCTGGAGGAGCAGGACGATGACCCGACTGATAATGAAGCAGGATGCAGCTTCGTTGTCGGATTGAT
CCAGAAGAACCGCCGCAAAATGAGGAAGGTCGGAGAGGACATGAACACCATCGGCTTCGCAATCTATGAGGTTCCTGACGAGTTTGTGGGACAGAGGAACGTTCACCTGGATCGTAACTTCTTTGTGCGACATGCTTCAGCTGCT
CGCTCCGAGACCTTCATCAACCTGCGGGAGGTTTGCTCTCGCTTCTGTCTGCCGCCCGGCGAGTACCTCATCGTACCCTCCACCTTTGAGCCCAACAAAGATGGCGACTTCTGTGTGCGCGTTTTCTCCGAAAAACAAGCAGAGT
TCCAA---------------------------------------
ATAAAAGAGGGAGATATCGATAGCCGGTTCAGGAATCTGTTCAAGCAGCTTGCTGGAGCTGACAGTGAGATCTCTGCATTTGAGCTGCAAAAAATCCTGAACAATGTGATAGCTAAA---------
ATTGGATTTAGCTTGGAGACCTGCCGCAACATGGTGAACCTACTGGACAAAGACGGCACTGGAAAACTAGGCCTGCTTGAATTTAAGATTCTG---ACTAAAATTGAG---------
GACGTCTACAGTAAAAATGACAAACAGTCCGGCACA------------------------------------GCAGGGTTTTCTCTGAATAACGCTCTGCACCAGATCCTTGTTGCACGATACAGCGAACTCACC------
ATCGACTTTGACAACTTTGTGGCCTGTCTTATCCGCCTCGAATGCATGTTCAAGGCATTTAAGGTGCTGGACAAGGACAAGAATGGCACAGTCGAGCTGAACATGCTC

ATGTCAGGAATCGCGATTAAACTCCAACACAAGCGGGCACAAGCGGCGGGCATCGGTACCAACGCTCAAGCGGTCAAATTCCTTAACCAGGACTATGAAGCCCTCAAACGAGAGTGTCTGGAGTCCGGACGTCTGTTTCACGATG
ACATGTTTGAAGCTAATGTGTCTGCCCTCGGGTTTAAGGAACTAGGACCAAACTCGTCTAAAGTGCGCGGGGTTGAGTGGCTGAGACCCAAACAACTGACGTCCAACCCAACATTCATTAGTGGAGGAGCGACCAGAACAGACAT
CTGCCAGGGCGCTTTGGGTGACTGTTGGCTATTAGCAGCCATCGCTTCTCTTACCCTGAACCAGGACGTGTTTGCTCGTGTTGTGCCAGCTGGTCAGAGTTTTGATGGCGACTATGCTGGAATATTTCATTTTCAGTTTTGGCAG
TTTGGTGAATGGGTGGATGTAGTCATCGATGACCGGCTGCCGGCTAGAAACGGAGAGCTGCTGTTTGTCCACTCGGCCGAGGGCTCTGAGTTCTGGAGTGCTCTTTTGGAGAAAGCCTATGCAAAGTTGAACGGCTGCTATGAAG
CTCTTTCTGGAGGCACCACCACTGAAGGTTTTGAGGATTTCACTGGAGGCATTGCCGAGGTCCACGAACTGGCCAAAGCAGGTCCAAACCTCTTCAAAACCATCCAGAAGGCTCTTAGCTGGGGCTCGTTGCTGGGCTGCTCCAT
AGATATCACAAGCTCTTCTGACTCAGAGGCCATTACTAGTCAGAAACTGGTGAAAGGACACGCCTACTCGGTTACAGGAGCTGAAGAGGTGGAGTACAGGGGTGACTTAACTAAACTGATTCGAATCAGGAACCCCTGGGGGCAG
GTCGAGTGGACGGGACCCTGGAGTGATGGGTCATCAGAATGGCGTCAGATC---
GACAACAAAGCTGAAGATGGAGAATTCTGGATGTCTTTCTCTGACTTCATGCGTCATTACTCACGGGTGGAGATCTGCAATCTGACGCCCGATGCGCTCACAGATGAAAGTGTTAATAAATGGGCTCTGTCCAAGTTTGATGGCA
ACTGGAGGAATGGATCAACTGCTGGAGGCTGCAGGAATTACCCCAACTCGTTCTGGATGAATCCTCAGTTTCTGATTAAACTGGAGGAGCAGGATGACGACCCGACTGATAATGAAGCAGGATGCAGCTTTGTTGTGGGACTGAT
CCAGAAGAACCGCCGCAAAATGAGGAAGGTCGGAGAGGACATGAACACCATCGGCTTTGCAATCTATGAGGTTCCTGACCAGTTTGTGGGACAGCGGAACGTCCATCTGGATCGTAACTTTTTCGTGCGACATGCATCAGCTGCT
CGGTCCGAGACCTTCATCAACCTGCGGGAGGTTTGCTCTCGCTTCTGTCTGCCCCCCGGCGAGTACCTCATCGTGCCCTCTACTTTTGAGCCCAACAAAGATGGTGACTTCTGTGTCCGCGTTTTCTCAGAAAAACAAGCAGAGT
TCCAA---------------------------------------
ATAACAGAGGGTGATATTGATAGCCGCTTCAAGAAACTGTTTGGACAGCTTGCTGGAGCTGACTGTGAGATCTCTGCATTTGAGCTGCAAAAAATCCTGAACAATGTGATAGCTAAA---------
ATTGGATTTAGTTTGGAGACTTGCCGCAACATGGTGAACCTACTGGATAAAGACGGCACTGGAAAACTAGGCCTGCTTGAATTTAAGATTCTG---ACTAAAATTGAG---------
GACGTCTACAGTAAAAATGACAAACAGTCTGGCACA------------------------------------GCAGGGTTTTCTCTGAATAATGCTCTACACCAGATCCTTGTTGCACGATACAGCGAACTCACC------
ATCGACTTTGACAACTTTGTGGCCTGTCTTATACGCCTCGAATGCATGTTCAAGGCATTTAAGGTGCTGGACAAGGACAAGAATGGCACCGTCGAGCTGAACATGCTC

ATGTCAGGTGTGGCGTCCACTCTGGCCAAGAAGCGGGCCCTGGCAGCAGGCTTCGGTACCAACGCCAACGCGGTTCCGTACCTGAATCAGAACTTCGCGGCTCTGCGTGCCGAGTGTCTGTCTGCGAGGAAACTGTTCTGCGACC
CGGCCTTCCCCGCCGCCCCAGAGGCCCTGGGCTTCAAGGAGCTGGGCCGGAGTTCATACAAAGTCCGTGGAGTTACCTGGAAGAGACCCGCGGAGCTGGTCTCTAACCCCGAGTTCATCATGGGCGGAGCCACCAGGACCGACAT
CTGTCAGGGGGCTCTGGGTGACTGCTGGTTGCTGGCCGCCATCGCCTCACTGACTCTCAATGAGTATGTGATGGCCAGAGTGGTTCCCAGTGACCAGGGCTTCGGTGATGACTACGCAGGCATCTTCCACTTCCAGTTCTGGCAG
TTTGGCGAGTGGGTGGACGTGGTGATCGACGACCGTCTGCCAGTCAAAGACAATGAGCTGATGTTTGTCCACTCTGCAGAAGGACGAGAATTCTGGAGTGCTCTTCTGGAGAAGGCCTATGCCAAAGTCAATGGCTGCTACGAAG
CTCTGTCTGGTGGCTCCACCACCGAAGGTTTCGAAGACTTCACCGGAGGCATCGCTGAGAACTACGACCTGCGCCAGCCTCCCTCCAACCTGTTCCAGATCGTGAAGAAGGCCCTAGAGGCTGGAGCTCTACTGGGCTGCTCTAT
CGACATCACCAGTGCGGCGGACTCTGAGGCGGTCACCCGTCAGAAACTGGTGAAAGGCCATGCCTACTCCCTGACAGGAGCTGTGGAGGTGAACTACCGGGGCCGTCAGGAGAAGCTAGTGCGGATGAGGAACCCGTGGGGTCAG
GTGGAGTGGACCGGTGCGTGGAGCGACGGGTCGTCTGAGTGGAACTACATG---------
AATGCCGAGGACGGAGAGTTCTGGATGTCCTTCAGCGACTTCTTACGCCATTATTCTCGTATAGAAGTGTGCACTCTGACCCCTGACACCATTTTAGACGACTCTGTCAAACATTGGAGCGTCAGTAAATTTGATGGCACCTGGA
GAAAAGGTTCCACTGCCGGAGGCTGCAGGAACCACCCCTACACCTTCTGGATGAATCCTCAGTTTGTGATAAACCTGCAGGAGGAGGATGATGACCCAGATGATGGAGAAGAAGGATGCAGCTTTGTGGTTGGCTTGATTCAGAA
GAACCGCAGGAAGCTTAGAAAACAAGGCGAGGACATGCACACCATCGGATTCGCCATTTATGAGGTTCCAGAACAGTTCAAAGGTCAGCGTGAAGTACACCTGGACAAGAACTTCTTCCTGAGTCACGCTCAGACAGCCAGGTCC
GAAACCTTCATCAATCTGCGGGAGGTGTGCTCTCGCTTCAAGCTCCCCCCTGGAGAGTACCTTATCGTCCCTTCCACCTTTGAGCCGCACCAGAATGGAGACTTCTGCATCCGAGTGTTCTCCGAGAAGCAGACAGACACCCAG-
--------------------------------------
GTATCAGATGAGGATGTGGATGCAGGATTCAGAGGTCTCTTCACAAAACTGGCAGGAAACGACATGGAGATCTCTGCAGTGGAGCTCCAAACCATCATGAACAAGATTGTTGGCAAA---------
ATCGGCTTCAGCATGGAGACCTGCAGGGTCATGGTCAACCTGATGGATGACAGTGGAAATGGAAAGCTGGGCCTGGGAGAGTTTGCCACCCTG---AAGAAAATCCAG---------
TCCATCTACAAGAAGAACGACATTAATTCAGGGACG------------------------------------GCAGGTTTCACCCTCAACAATACCATCTATCAGCTGCTGGTGGCCAGATACTCTGATATGACC------
ATTGACTTTGACAACTTTGTGGGATGTCTGATGAGGCTGGAGATGATGTTCAGAATTTTCAAGAAGTTGGATGTTCAGAACAGCGGCTTGATAGAGCTGAACATGGTT

ATGTCCGGCGTGGCGTCCACTCTGGCCAAGAAGCGCGCTCTGGCCGCGGGCTTTGGCACCAACGCGAACGCGGTCCCGTACCTGAACCAGAACTTCAACGCGCTGCGCGCCCAGTGCCGCTCCGCGGGCAAGCTCTTCTGCGACC
CGACGTTCCCCGCCGCGCCCGAGTCTTTGGGCTTCAACGAGCTGGGCCCGAGCTCCTACAAGGTCCGCGGAGTCAGCTGGAAGAGGCCCACGGAGCTCGTCTCTAACCCGGAGTTCATCGTGGGCGGAGCCACCAGGACTGACAT

CTGCCAGGGCGCACTGGGTGACTGCTGGCTGCTGGCGGCCATCGCCTCTCTGACCCTGAATGAATATGTGATGGCCAGAGTTGTTCCCACGGACCAGGACTTTGGGGACAGCTACGCTGGCATCTTCCACTTCCAGTTCTGGCAG
TTCGGGGAGTGGGTGGACGTGGTGATCGACGACCGGCTGCCGGTCAAAGACGGCGAGCTGATGTTCGTGCACTCGGCTGAGGGGAGGGAGTTTTGGAGCGCCTTGTTGGAGAAAGCCTACGCCAAAGTGAACGGCTGCTACGAGG
CGCTGTCTGGCGGGTCCACCACTGAGGGCTTCGAGGACTTCACCGGCGGCATCGCAGAGAACTACGACCTCAAAAAGCCCCCCTCCAACTTGTTCCAGATCATCAAGAAGGCGCTGGAAGCGGGATCACTGCTGGGCTGCTCCAT
CGATATTACGAGCGCCGCAGACTCTGAAGCTGTGACTCGTCAGAAGCTGGTGAAAGGCCACGCCTACTCGCTGACGGGCGCCGTGGAGGTGAACTACCGAGGGCGTCAGGAGAAGCTGGTGAGGATGAGGAACCCGTGGGGCCAG
GTGGAGTGGACGGGACCGTGGAGCGACGGGTCGTCTGAGTGGAACTACGTG---------
AACGCAGAAGATGGAGAGTTCTGGATGTCCTTCAGCGACTTCCAGCGTCACTACTCTCGTATTGAGGTGTGCACTCTGACCCCGGATGCCATCGAGGACGACTCCGTCAAACACTGGAGCGTCAGCAAGTTTGACGGCGGCTGGA
GGAGAGGCTCCACCGCCGGAGGCTGCAGGAATAACCCGTACACGTTCTGGACGAATCCACAGTTCGTGATTAAGCTTGCCGAGGAGGATGATGACCCTGACGACGGCGAGGTGGGCTGCAGCTTCGTGGTGGGTCTGATCCAGAA
GAACCGCAGGAAGCTGCGCAAACAGGGCGAGGACATGCACACCATCGGCTTCGCCATCTACGAGCTTCCACAACAGTTTCACGGCCAGAGGGACGTACACCTGGATAAGAACTTCTTCCAGACCCCACGCTCAGACGGCGAGGTCG
GAAACCTTCATCAACCTGCGTGAGGTCAGCTCTCGCTTCAAGCTGCCGCCGGGCGAGTACCTGATTGTCCCGTCCACCTTCGAGCCGCACCTGAACGGAGACTTCTGCATCCGGGTGTTCTCCGAGAAGCAGACGGAGACCGTG-
-------------------------------------
GTGTCAGACAACGAGGTGGACGCCGGGTTCAGAGGCCTCTTCACTAAACTCGCCGGAAGTGACATGGAGATCTCTGCAGTGGAGCTGAGGACCATCATGAACAAAATCGTCTCCAAA---------
ATCGGCTTTGGCATGGAGACGTGCAGGGTGATGGTGAACCTGATGGACGACAGCGGCAACGGGAAGCTCGGCCTCGGGGAGTTTGCCACGCTG---AAGAAGGTGCAG---------
GAATTTTATAAGAAGAACGACTCTAACTCAGGGACG-----------------------------------GCAGGTTCACTCTCAATAACAACATCTACCAGCTGCTGGTGGCCCGATACTCCGACATGACC------
ATCGACTTCGACAACTTCGTGGGATGTCTGATGAGGCTGGAGATGATGTTCAAGGTCTTTAAGAAGCTCGACACTAATGGCAGCGGTTCCATTGAGCTCGACATGATC

ATGTCCGGCGTGGCCTCCACTCTGGCCAAGAGGCGCGCGGTGGCCGCGGGCTTCGGCACCAACGCCAACGCGTCGCGGTACCTGAACCAGGACTTCGCGTCTCTGCGCGCGCAGTGCCGCTCGGCCGGTAAACTCTTCTGTGACG
TCACGTTCCCTGCGGAGCCCGAAGCTCTGGGCTTCAAAGAGCTGGGCCGCGGCTCCTCAAAGGTCCGGGGAGTCACCTGGAAGAGGCCCACGGAACTTGTGTCAAATCCTGAGTTCATCTTGGGGGGAGCAACTAGAACCGACAT
CTGCCAGGGAGCTCTAGGTGACTGCTGGCTGTTGGCGGCCATTGCCTCGCTCACGCTGAACGAGTATGTGATGGCCAGAGTTGTCCCCACCGACCAGGGCTTCGGAGACAACTACGCTGGCATCTTTCACTTCCAGTTCTGGCAG
TTTGGGGAGTGGGTGGACGTTGTCATTGACGACCGTCTGCCGGTGAAAGACGGAGAGCTGTTGTTCGTCCACTCAGCGGAGGGGAGGGAGTTCTGGAGCGCCCTGCTGGAGAAGGCCTACGCCAAAGTCAACGGCTGCTACGAGG
CCCTCTCTGGGGGATCCACTACCGAGGGCTTCGAGGACTTCACAGGAGGCATCGCCGAGAACTTCGACCTCCAGCGCCCCCCCCGAACCTCTTCTACATCATCAAGAAGGCGCTGGAGGCCGGAGCGCTGTTGGGCTGCTCCAT
TGACATCACCAGCGCCGCCGACTCGGAGGCCGTGACCCGTCAGAAGCTGGTGAAAGGCCACGCCTACTCGCTGACCGGCGCCGTGGAGGTGAACTACCGTGGCCGGCAGGAGAAGTTGGTGAGGGTCAGGAACCCCTGGGGTCAA
GTGGAGTGGACGGGAGCCTGGAGCGACGGATCGTCTGAGTGGAACTATGTA---------
AAGGCCGAGGACGGAGAGTTCTGGATGTCCTTCAGCGAGTTCTCACGTAACTACAACCGCGTGGAGGTGTGCACCCTGACCCCCGACGCCATCGACGACGACTCCGTCAAACATTGGAGCGTCAGCAAGTTCGACGGCACCTGGA
GGAGAGGCTCCACGGCTGGGGGCTGCAGGAACCACCCGTACACCTTCTGGACGAACCCCCAGTTCGTGGTCCGCCTGGAGGAGGAGGACGACGACCCCGATGACGGAGATGTGGGCTGCAGCTTTGTGGTCGGCCTGATCCAGAA
GAACCGCAGAAAGCTACGTCAGCAGGGCGAGGACATGCACACCGTGGGCTTTGCCATCTACGAGGTTCCAAAGCAATTCTACGGGCAGCGGGAGGTGCACCTGGACAAGAACTTCTTCCTGACTCACGCTCAGACGGCCAAGTCG
GAGACATTCATCAACCTCCGTGAGGTGAGCACGCGCTTCAAGCTGCCGCCAGGGGAGTACCTGGTGGTCCCGTCCACCTTCGAGCCGCACCTCAACGGAGACTTCTGCATCCGTGTGTTCTCCGAGAAGCAGACCGAGACCCTA-
-------------------------------------
GTGTCAGATGAAGGTGTTGATGCAGGATTCAGAGGCCTCTTCACCAAACTCGCCGGAGACGACATGGAGATCTCTGCGGGGGAGCTGAGGACCATCCTGAACAAGATTGTTGCCAAA---------
ATAGGCTTCAGTATGGAGACCTCCAGGATCATGGTGAACCTGATGGACGACAGCGGGAACGGGAAGCTCGGCCTCGGAGAGTTTGCCACTCTG---AAGAAGGTGCAG---------
ACCATTTACAAGAAAAACGACGCTAACTCCGGGACG-----------------------------------GCAGGTTTCACTCTCAACAACAACATCTACCAGCTGCTGGTGGCTCGATACTCTGACATGACC------
ATTGACTTCGACAACTTCGTTGGCTGTCTGATGAGGCTGGAGATGATGTTCAAGATCTTCAAGAGGCTCGACCCTCACGACAGTGGCTCCATCGAGCTCGACATTTTA

ATGTCCGGCGTGGCATCCACACTCGCCAAGAAGAGAGCCCTGGCGGCTGGCTTTGGGACGAACTCCAATGCCGTGAAATACCTGAACCAGAGCTTCGAGACTTTGAGGAGTGAATGCCTGAGCCCGCGGAGAGCTTTTCTGTGATC
CCGCTTTCCCAGCCGCTCCCGAGGCCCTGGGCTTTAATGAACTCGGGCCACGGTCGTCAAAAACCAGAGGTGTTGAGTGGAAAAGACCAGGGCAATTGACTTCCAGTCCGGAGTTCATAGTCGGAGGAGCTACAAGAACAGATAT
TTGCCAAGGAGCTTTAGGTGATTGCTGGCTCTTAGCAGCCATCGCCTCTCTGACCCTTAATGAAGACGTTTTGGCTCGTGTCGTACCATCCGATCAGGGATTTGGGCAAGACTATGCTGGAATTTTCCACTTTCAGTTTTGGCAG
TTTGGAGAATGGGTGGATGTGGTGGTGGATGATCGATTGCCCACTAGAGATGGGGAACTGCTCTTTGTTCACTCCGTCACAGGCTCTGAGTTCTGGAGCGCTCTGCTGGAGAAGGCCTATGCAAAAGTGAATGGATGTTATGAAG
CCCTTTCTGGAGGTTCCACCACAGAAGGTTTTGAGGATTTCACTGGTGGGATTGCAGAGATGTACGAGCTGAAAAGTGCACCGACCAACCTGTTTCAGATCATCAAGAAGGCCCTGGACTCTGGAGCTCTGTTGGGATGCTCCAT
AGATATCACCAGTGCTGCTGATTCTGAGGCCGTCACTTATCAGAAGCTTGTAAAGGGACATGCATACTCTCTTACTGGAGCTACTGAGGTAAATTACAGAAGTCGAAAAGAAAAGCTGGTCCGAGTGCGTAATCCTTGGGGTCAG
GTGGAATGGACTGGGGCTTGGAGTGATAACTCTTCTGAGTGGAACGCTGTC---------
AAATCAGAAGATGGAGAGTTCTGGATGTCATTCTCAGATTTTGTGAGGCAGTATTCCCGCCTTGAGATCTGCACTCTGACCCCAGACACGCTGACGTCTGACTCGCTCAAACACTGGAGTGTGTGTAAATTTGATGGCACCTGGA
GAAAAGGCTCTACGGCTGGTGGTTGCAGGAACCATCCATACACGTTCTGGATGAATCCTCAGTTTAAGATCAAGCTGGAGGAGGAAGATGATGATCCGGATGATAATGAAGTGGGCTGCAGTGTGGTGATCGGCTTGATTCAGAA
GAACCGCCGCAAGATGAGGAAGGCTGGAGAAGACATGCACACTATCGGCTATGCCATCTACGAGGTGCCTTCTCAGTTTCATGGGCAAAAGGAGGTTCATCTGGATAAGAACTATTTCCTGACTCACGCTCAGAAAGCTCGATCT
GAAACCTTTATCAACCTCAGAGAGGTCAGCACCCGCTTCAAACTTCCTCCTGGAGAATATCTCATCGTCCCTTCTACATTCGATCCGCACAAGAACGGGGATTTTTGTGTGCGTGTGTTCTCCGAGAAACAGTCTGAGATGCAA-
-------------------------------------
GTGTCTGAAGAAGAGGTTGATTCTGGCTTTAGGGGCCTGTTTACTAAACTTGCAGGAGATGACATGGAGATCTCTGCTTCTGAGCTGCGAACGATTTTCAACAAAATAGTGGCAAAG---------
ATCGGTTTCAGTCTGGATACATGTAGGGTGATGGTCAACCTCATGGATGAAAGTGGAAATGGAAAACTAGGATTAACTGAGTTTGCTACACTT---AAGAAGATTCAA---------

GGCATTTATAAGAAGAATGACATGGGATCCGGCTGT------------------------------------GCAGGATTCTCTCTGAATGACTGCATTCACCAGAGTCTGGCTGCACGCTATGGAGACATGACC------
ATCGACTTTGACAACTTTGTGTCCTGTGTGATGCGCCTGGAGATGATGTTCAAAGTGTTCAAGAGGATGGATATAGACCACAGCGGGTTCATTGAGCTGGACATGATT

ATGACCGGCATGGCATCTACACTGGCCAAAAAGCGGGCACTGGCGGCGGGGTTCGGCACCAACTCCAACGCGGTGAAGTACCTCAACCAGGACTTCGAGGAGCTGAGACGGCAGTGTCTGACCGCGGGCAAGCTCTTCTCGGACT
CGACCTTTCCAGCCGCGCCCGAGTCCCTGGGATTCAAAGAGCTCGGTCCGTACTCCTCCAAAACCAGAGGAGTGCAGTGGAAAAGACCAAGTGAACTGTGCTCCAGGCCTGAGTTCATTGTGGGCGGAGCCACGAGGACAGATAT
CTGCCAGGGGGCGCTCGGTGACTGCTGGCTCCTGGCGGCCATTGCCTCCTTGACCCTTAATGAGGACGTCTTGGCCAGAGTTGTGCCAAGTGGACAGGGCTTCGGAAATGACTATGCTGGGATCTTTCACTTCCAGTTCTGGCAG
TTTGGAGAATGGGTAGATGTTGTCATCGACGATCGGCTGCCCACCAGAGACGGGGAGCTGCTGTTTGTCCACTCAGCGGAGGGCTCGGAGTTCTGGAGCGCCCTGCTGGAGAAGGCCTATGCCAAGGTCAATGGCTCCTATGAGG
CTCTGTCTGGGGGCTCCACCACTGAAGGCTTCGAGGACTTCACCGGGGGGATAGCTGAGACGTACGAGCTGAAGCAGGCCCCCCCAAACCTCTTCCAAATCATCAAGAAGGCCCTGGAGGCTGGCTCTCTGCTGGGCTGCTCCAT
TGATATCACCAGTGCTGCCGAGACGGAGGCCATCACCTATCAGAAACTGGTGAAGGGACACGCCTACTCCCTCACTGGTGCTGTGGAGGTGACGTACCGGGGACGCCTGGAGAAGCTGGTCAGAATCCGTAACCCCTGGGGTCAG
GTGGAGTGGACAGGAGCCTGGAGTGACAGCTCGTCTGAGTGGAACTACGTT---------
AAAGCAGATGATGGAGAGTTCTGGATGTCGTTCACAGAATTCATGAAGCAGTACTCACGGCTGGAGATCTGCAACCTGACCCCAGACACCCTGTCCAGCGACTCGTACAAGCGCTGGAGCGTTTCCAAGTTTGACGGCGCCTGGA
GGCGGGGCTCCACCGCAGGCGGCTGCAGGAACAACCCCTACACCTTCTGGATGAACCCGCAGTTTGTGATCAAGCTGGAGGAGGAGGATGACGATCCTGAAGACAACGAGGTGGGCTGCAGCTTCGTGGTGGGTCTGATTCAGAA
GAACCGCCGGCGGCTGAGGAAGGCGGGCGAGGACATGCACACCATCGGCTTCGCCATCTACGAGGTGAACAGACCTTTCCGTGGTCAGACAGAGGTGCACCTGGACAAGAACTACTTCCTGACACACGCACAGACCGCTCGCTCT
GAGACCTTCGTCAACCTGAGGGAGGTCTCCAATCACTTCAAGCTGCCCCCGGGGGAGTACCTCATCGTTCCTTCCACCTTCGAGCCGCACAAGGACGGCGACTTCTGCCTGCGAGTCTTCTCAGAGAAGCAAGCCGAAACACAA-
--------------------------------------
GTTTCTGAGGGTGAAATTGACGCAGGCTTCAAGAACCTGTTTGTCAGGCTTGCTGGATCGGACAATGAGATTTCTGCAACAGAACTGAGGACTATTTTTAATAATATTGTCTCCAAA---------GTG----------------
--------------------------------------------------------------------------------------------------------------------------CAGGCAGGACAG-----------
-------------------------CCTGGTTTCACCTTAACCAATAACATCCACCAAGTCCTTGTGGCCCGCTATGCTGAAATGACT------
ATTGACTTTGACAACTTCGTGGCCTGCCTGATTCGTCTGGAGACGATGTTCCAAGTGTGCCGGTGCCTGTCAGCCGAACACCCGAGCGCAGGTGAAGACGGT------

ATGGCAGGAATTGCGTCTACTGTGGCCAAGAAGAGAGCGCTGGCTGCAGGGTTTGGAACCAACGCCAATGCCACCAAGTACCTGAACCAGGACTTTGAGGCGCTCAGGAGTGAGTGCAGGGCCAGAGGGACCCTGTTCTCAGACC
CGACTTTCCCCGCCGCTCCTGAATCACTGGGCTTTAAAGAACTCGGACCCAACTCGTCCAAAACCAGAGGATTAGTATGGAAAAGGCCCGGGGAATTATGCCAGAAACCACGCTTCATCGTTGGTGGGGCCACAAAAACGGATAT
CTGTCAAGGAGCTTTGGGTGACTGTTGGCTGCTGGCTGCTATTGCCTCCCTGACACAGAATGAAGATGTTTTGGCAAGAGTTGTGCCCAATGGACAGGAGTTTGATGGCACTTATGCAGGAATCTTCCATTTCCAGTTCTGGCAG
TTTGGCGAGTGGGTGGATGTGGTCATTGACGACCGGCTGCCGGTGAAAGATGGCGAGCTGTTGTTTGTTCACTCCGCTGAGAAAAACGAGTTCTGGAGCGCCCTGCTGGAGAAGGCCTATGCTAAGGTGAACGGCTGTTATGAAG
CCCTGTCTGGAGGATCCACTAGCGAGGGTTTTGAGGACTTCACTGGAGGCATCGCTGAGAGCTACGAGATCAGAAAAGCACCGACCAATTTGTTTCAGATCATCCAGAAAGCCCTGGAGGCTGGAGCTTTGCTGGGCTGCTCCAT
CGACATCACAAGTGCTGCTGATTCAGAAGCCATCACTCGTCAGAAGCTGGTCAAAGGCCACGCCTACTCACTCACAGGGGCAACAGAGGTGAACTATCGTGGTCGCAAGGAAAAGCTGGTTCGTATGCGCAACCCTTGGGGACAG
GTGGAGTGGACCGGAGCCTGGAGCGACAATTCATCTGAATGGAACAGTGTT---------
AGCGCTGAGGATGGTGAATTTTGGATGGCATTCTCCGAATTCCTCACCAATTACTCTCGTATAGAGATCTGCACTCTGACGCCGGATGCCATCACAGATGATTCAGTAAAACATTGGGCCGTGACAAATCATGACGGCACATGGA
GGAGAGGCTCCACCGCTGGAGGCTGCAGAAACAATCCATACACATTTTGGATGAATCCACAATTTGTGGTGAAACTGGATGAAGAA------
AATGTCGAAGATAAGGAGAAGGGCTGCAGTTTTGTAGTGGGTCTGATTCAGAAGAACCGCCGGCGCCTGAGGAAAACTGGAGAGGATATGAACACTATTGGCTTTGCTATTTATGAGCTCCCCCAGGAGTTTCACGGTCAGCGGG
AAGTGCACCTGGATAAAAACTTCTTCCTGACGCACGGACAGAAAGCTCGCTCTGAGACTTTCATTAACCTCAGGGAGGTCTGCACGCGCTTCCATCTGCCTCCTGGAGAGTACCTCATCGTACCATCTACCTTTGAAGCCAACAA
GGACGGAGACTTCTGCCTGCGTGTCTTCTCTGAAAGCCAAAGTGAAACACAA---------------------------------------
GTCTCAGATGATCAAGTTGATTCAAAGTTTAGGGGGATGTTCGTAAAACTTGCCGGACCTGACATGGAAATCTCACCCCTGGAGCTCATGACCATCTTTAACAAAGTCATTGCAAAA---------
ATTACCTTCACTTTGGATACGTGTCGTGTCATGGTGAACCTCATGGACGACAGTGGAAATGGAAAACTAGGGCTGGGAGAGTTTGCTACTCTG---AAGAAAGTGCAG---------
GAAATTTACAAGCATAATGACTTGAGCTCGGGAACC------------------------------------GCCGGTTTCTGTCTCAATAACACTTTATTTCAGCTGATGGTTGTACGTTATGCTGAGATGACT------
CTCCTCTTTGACAACTTCGTGTCCTGCCTGATGCGACTTGAAATGATGTTCAGGGCATTCAAAAGGTTGGATCCTCACAAAAGTGGCTTCATTGAGCTGAATATGATA

ATGTCAGGAATGGCATCTAATATCAAGAAGCAGAGGGAGAGAGCGGCCGGGATCGGCTCCTGGCAGCAACCGGTGAAGTTTCTCAATCAAGATTATGAAGCGATCCGGCAGCATTGCTTGGATACTGGAAACTTGTTCTGTGACG
AGTCTTTCCCTGCTTGTCCTACATCCCTGGGATTCAAAGAACTCGGACCCGATTCGTACAAAACCAGAGACGTCGAGTGGATTCGCCCGAAGGATGTGTGTGGCAACCCTGAATTTATAATTGGTGGAGCATCACGTACTGATAT
CTGCCAGGGAGCGCTAGGTGACTGCTGGCTATTGGCTGCTATTGCATCACTAACCCTAAACAATGAGGTTCTAAACAGAGTGGTTCCCTGTGAACAAGACTTTGGCTCTGATTATGCAGGAATCTTCCATTTCCGGTTCTGGCAG
TATGGAGAGTGGGTGGATGTGGTGATCGATGACAGGTTGCCCACAAAGGATAATGAGCTGATGTTTGTGCACTCAGCTTCAGGAAATGAGTTCTGGAGTGCTCTGCTGGAGAAAGCCTATTCAAAGTTAAATGGGTCCTATGAAG
CATTGTCTGGGGGATCTACAACTGAAGGCTTTGAGGATTTTACAGGGGGGATTTCAGAGTGGTATGAATTAAATGAAGCACCAAGCAACCTCTTTAAGATCATACGAAAGGCATTAAAAGCTGGGTCACTACTTGGCTGTTCCAT
TAATATCACCAGCTCAGCAGATTCAGAGGCAATTACTTCACAGAAGCTGGTCAAAGGTCATGCTTATTCTTTAACTGGAGCGGAAACAGTGGACTATCAAGGCAACGCGGAGCAATTGGTCCGCGTCCGTAATCCATGGGGTCAA
GTGGAGTGGACTGGAAGCTGGTCCGACAATGCACCAGAGTGGGAATGCGTT---
GAAGAACAATCAGAGAATGGCGAATTCTGGATGTCATTTTCCGAATTTCTTAGTAATTTCTCTCGAGTGGAAATCTGCAATCTCACCCCCGACACACTTAGCAATGATGAGGTTCACAAGTGGAACATGGCTGTCTACAATGGGT
CCTGGAGGAAAGGATCTTCTGCGGGAGGATGTCGAAACTATATAAGAACATTTTGGCAAAATCCTCAATTTAAGATTGATTTGAGGGAGGCAGATGAAGATCCTGATGATGATGATGAGAAATGCAGTTTTCTGGTCGGGGTTAT

TCAGAGGAATCGTAGAAATAAAAGGAAAATGGGCGAGGACATGCATACTATTGGCTTTGCTGTCTATGAGGTCCCTGAAGAGTACCACGACCACAAGAATATTCAACTCAGGAGAGATTTTTTCACAACTCATGCATCCCAAGCA
AAATCAGAAACCTTCATCAATCTGCGAGAAGTCATGAGCAGGCTTGCACTTGAACCTGGACAATATTTTGTAATACCATCCACCTTTGAGCCGAATAAAGATGGGGATTTTGCAATCCGCTTGTTTACAGAGAAGGCAGCCAATG
TAGAA---------------------------------------
GTTGACGAGGATGACATTAGTGCCAGCTTTGAGAATTTGTTTGAATATATAGCAGGGGAGGATGCAGAGATTTCTTGCTTTGAGCTGCAAAGAATACTGAACAGAATTGTAGGCAAC---------
ATTGGTTTTAGTTTGGAGACCTGTCGAAATGTAATCAGTCTAATGGATAAGGATGGATCTGGAAAAATTGGATTAGTGGAATTCAAGCTATTT---AACAAGTTGCAG---------
AAAATCTTCAAGAGGGTAGATGCTGAATCTGGTACC------------------------------------GCAGGTTTCACTCTGAACACTGCATTGATTCAGAACATTGTTGCCCGTTATGCAAACCTTCTC------
ATCGACTTTGATAACTTTGTTGGCTGTTTGATCCGATTGGAAACCATGTTCAGAATATTTAAAGCACTAGAAGTCGATGATGAGGGATCGGCAGAGTTGGATCTGGGC

ATGTCAGGGATGGCTTCGAGGATCAAGAAGCTGAGGGACCAGGCGGCGGGTATCGGCTCCTACGGGCAGCCCGTCAAGTTCCTCCGGCAGGATTACGAGGCTCTCCGGCAGCAGTGCCTGGAGACCGGGAGCCTGTTCTGTGACG
AGTCCTTCCCCGCCTGTCCTTCGGCGCTGGGCCACAATGAACTGGGACCGGGCTCCTTCAAAACCCGGGATGTCGAGTGGAGGCGCCCAAAGGACCTGTGTGCGGACCCCAAATTTATTGTTGGAGAAGCTTCACGCACGGACAT
CTGCCAGGGAGCATTGGGTGACTGCTGGCTCTTGGCTGCCATTGCGTCGTTGACCCTGAATAATGAGATTCTGTCCAGGGTTGTTCCCAGGGATCAAAACTTTGATTCTGATTATGCAGGAATCTTTCATTTCCAGTTCTGGCAG
TACGGAGAATGGGTGGATGTGGTAATTGATGATAGGTTGCCCACAAAGGATGATCAGCTGATGTTTGTGCACTCTGCATCTGGGAATGAGTTCTGGAGTGCTCTGCTGGAGAAAGCCTATTCAAAGTTAAATGGATCGTATGAAG
CGCTTTCTGGAGGATCCACCACTGAAGGGTTTGAAGATTTTACGGGAGGAATTTCGGAGTGGTACGAATTAGAAAAAGCACCAAGCAACCTCTTTAAGATCATCCGAAAGGCATTAAGAAGTGGGTCACTGCTTGGCTGCTCTAT
CAATATCACCAGTTCAGCAGAGACGGAGGCAATCACTTCACAGAAGTTGGTCAAAGGTCACGCTTACTCCGTTACTGGAGCAGAAATAGTGAATTATCAAGGTGATGAGGAGAAACTGATCCGCATCCGGAACCCGTGGGGTCAA
GTGGAGTGGACAGGAAGCTGGAGTGACAAAGGGGCTGAGTGGGATTGCATC---
GATGAAGAATCAGATGATGGAGAATTCTGGATGTCATTTACCGATTTTCTCCAAAATTTCTCGCGAGTGGAAATCTGCAACCTCACTCCTGACAGCCTTAGCAGTGATGAAGTTGGCAAGTGGAGCATGGTGATGTACAATGGGA
GCTGGAGAACTGGATCTACTGCAGGGGGGTGCCGAAACTATCCAAGGACTTTTTGGACAAATCCTCAATTTAAGATCCAGTTGGATGAAGCAGATGATGATCCTACTGATGACGATGATAAATGTAGCTTCCTGGCTGGTGTGAT
ACAAAAGAATCGTAGATGTAAACGGAAAATGGGCGAGGACATGCGTACCATAGGCTTCGCTATCTATGAGGTCCCTGAAGAGTACCAAAACCAGACCAATGTTCAGCTGAAGAGAGATTTTTTCATGACGAATGCATCCAGCGCA
AGATCGGAAACCTTCATCAACCTGCGGGAGGTCATGAGCCGGCTTGCTCTTGAACCTGGGCAATATTTCATTATACCATCCACTTTCGAGCCAAATCAAAGTTGCGACTTTTTGATTCGTATCTTTTCAGAAAAACAAAGTAACG
TACAA---------------------------------------
GTCGAAGAAGAAGACATTAGTGACAGTTTCGAGAATATGTTTCAACATTTAGCAGGGCAGGATTCAGAGATATCTGCCTTTGAGCTGCAAAGAATACTGAACAGAGTTGTAGCTAAC---------
ATTGGTTTTAGTTTGGAAACCTGCCGAAATGTAATAAGCCTATTGGATAAAGATCGATCTGGGAAAATTGGACTAGTGGAATTCAAGATATTT---ATAAAATTACAG---------
AAAATCTTCAAGAAGGTGGATGCTGATTCTGGTACC------------------------------------GCAGGTTTCACTCTGAACAATACATTGTTTCAGATCCTTGTTGCCCGATATGCAGATCTTCTC------
ATTGACTTTGATAACTTTGTTGGCTCTTTGATCCGTCTGGAAACCATGTTCAGAATATTCAAACAATTAGACAAGGATGATGAGGGATATGCAGAGCTCAATCAAAAC

ATGTCCGGGATGGCCCTGACCATTTCCAAGGCTCGGCAGAGACGGGCCGGGGTCGGCTCTAATGCAGCAGCCAGCCCCTTCCTGAAGCAGGAATACGCGGTTCTCCGGGATCACTGTCTCCAAACAGGCTCCTTGTTCCAGGATC
ACAGCTTCCCCGGCCAACGCCAGCTGCCTGGGATACAAGGAACTCGCCCCCAACTCCTACAAAACCCGGGATGTCAGCTGGCTGAGACCCCGGGAGATCTGCTCCAACCCCTCAATTTCTCATTGAAGGAGCCACTCGGACAGACAT
CTGTCAGGGAGCCCTGGGTGACTGTTGGCTCCTAGCTGCGATCGCCTCTCTGACCCTGAATGAAGAGATCCTCAGTCGGGTGGTACCCCCGGGACAATCCTTCGGGCCCGGCTATGCTGGAATCTTCCATTTCCAGTTCTGGCAG
TTTGGGGAGTGGGTGGACGTGGTGGTTGATGACCGACTGCCCACCAAGGATGGAAAGCTGATGTTTGTCCACTCGGCCGAGAGGAATGAGTTCTGGAGCGCTCTCCTGGAGAAGGCCTACGCCAAGTTGAATGGGAGTTATGAAG
CTCTGTCGGGAGGCTCAACCACGGAGGGTTTTGAAGATTTTACTGGTGGGGTTTCGGAATGGTTTGAGCTGAAGTCTGCTCCTCGGGACCTGTTCCACATCATCGAGAAAGCTCTGCAACGCGGATCATTGATGGGCTGCTCCAT
CGACATCACCAGTGCTGCTGAGACCGAGGCTGTTACCGGGCAGAAGTTGGTGAAGGGACACGCGTACTCACTGACTGGGGCCCAGCAGGTTGCGTTCCGAGGTACCCAGGTCCAGTTGGTCCGGATTCGGAATCCCTGGGGAGAG
GTGGAATGGACCGGAGCCTGGAGTGACAATTCACCACATTGGAATGGGATA---
GCGGAGCGGAACGAAGATGGGGAGTTTTGGATGGCTTTCCCTGAATTTCTGAGACATTATTCCCGCCTGGAGATTTGTAACCTGACCCCCGACGCCCTGAGCAGTCAGCAGGTCCTCAAGTGGAATTCGACTCTATTCAGTGAGA
GCTGGAGGAGGGGGTCCACTGCAGGGGGCTGTCGCAACTACCCAGGGACGTTCTGGATGAACCCTCAGTTTAAGATTACCTTGGAGGGCAGTGACCCTGAC------GACAGTGAG---
CAGTGCCACTTCCTGGTTGCTCTCATTCAGAAGAATCGGAGGCAACAGAGGAGGATGGGGGAAGATATGCACACCATTGGATACGCCATCTACGATGTTCCCGATGAGTATAAGGGGGTGTCCAATATCCATCTGCCAAAGAGTT
ACTTCCTGACCCACGGCTCCCGTGTACGTTCAGAGACTTTTATTAACCTGCGTGAAGTCTCGACTCGATTCCAACTGCCCCCTGGAGAATATCTCGTTGTGCCCTCCACCTTTGAACCTCACCAAAATGGAGATTTCGTTGTGAG
GGTCTTTTCGGAGAATCGCTCGCAGACCGAG---------------------------------------
GTCAACGAGAGAGATATCCCAGACAACTTCCGGACACTCTTCGAGAAACTGGCAGGACCGGGGAAGGAGATTTCTGCCTTTGGTTTGCAGAGAATCTTAAACAAAGTGGTGTCCAGC---------
ATTGGATTTGGCCTGGAGACTTGCCGGAATATGGTCAACCTCCTGGATATGGATGGGAACGGGAAGCTGGGTTTGGTGGAGTTTAAGAAACTG---GACAAGATTCAG---------
AAAATCTACAAGAAGAATGATCTGCAGTCTGGTACC------------------------------------GCTGGTTTCCACTTGAATAACCAGCTGACCCAGATCATTGTGGCCCGCTATTCCGAGCTCACC------
CTTGATTTCGATAACTTTGTTTCCTGTCTCGTTCGCCTGGAAGCTGTTTTCAAAATGTTTAATTCTCTGCCCAAGGATGGGGATGGTCTTGTCGAGCTCGGAATGGGC

ATGAGCGGAATGGCACTGGCGATCCAGCGCTCTCGGCAGCGGGACGCGGGGTTGGGCTCTCGGGGCAATGCGCGGCGGTTCCTGGGGCAGAGCTACGAGGAGCTGCGGGACCGCTGTCTGCAGACGGGGGTCCTCTTCCAGGACC
ACAGCTTCCCCGCCGCCCCGGACAGTCTGGGCTACCAGGAGCTGGGCGGCCACTCACACAAAACCCGGGGGGTGGAGTGGCAGCGCCCACCGGATCTGTGCTCAAGACCCCGGTTTATTATTGAAGGAGCCACCAAGACTGACAT
TTGCCAGGGCTCCTTGGGGGATTGCTGGCTCCTGGCTGCCATCGCGTCCCTGACTTTGAACAAGGAAGTGTTGGACAGAGTGGTTCCCGATGGACAGAGCTTTGATTCGGGCTACGCCGGAATCTTCCATTTCCAGTTCTGGCAG
TACGGGGAGTGGGTGGATGTGGTGATCGATGACCAGCTGCCCACCAAGGATGGAGAGCTAATGTTTGTCCACTCGGACGAGCAGAATGAGTTCTGGAGCGCGCTGCTGGAGAAAGCTTACGCCAAGCTGAATGGATCGTATGAGG

```
CTCTGTCGGGTGGATCGACGACCGAGGGGTTTGAGGACTTCACGGGGGGCGTGTCAGAGTGGTACGAGCTGAACAAGGCCCCTGCGGACCTTTTTCACATCATCGAGAAGGCGCTGAAGAGGGGCTCACTGCTCGGCTGCTCCAT
CAATATCACGAGTGCATCAGAGACCGAGGCGATCACCCCCGAGAAGCTGGTGAAAGGTCACGCCTATTCCATCACTGGAGCACAGGAGGTTCGGTTCCGCGGGTCCCAGGTGCAGTTGATCCGGATCAGGAACCCCTGGGGGCAG
GTGGAGTGGACGGGAGCCTGGAGTGACAGCTCCTCGCATTGGAACTCAGTG---
CCACAGCGCGCCGAGGATGGAGAATTCTGGATGTCGTTCTCGGACTTTAAGAGGCACTATTCTCGGGTTGAGATCTGTAACCTGACGCCTGACACCCTCACCTCCGACAAGATCCTCAAATGGAACTGTGCCTTGTTCAGTGAGA
GCTGGAGACGTGGCTCCACAGCTGGAGGCTGCCGGAACTTCCCAGCCACCTTCTGGATGAACCCCCAGTTTAAGATGGTGCTGGAGGAAATCGACGATGATGGACGAGGGGAGGAC---
GGCTGCAGCGTCCTGGTGGCTCTGATCCAGAAAAACCGAAGGAACCTGAGGAAGATGGGCGAGGACATGCACACTGTGGGCTTCGCCATCTATGAGGTCCCTGATGAGTACAAAGGGGTCACAAACGTGCACCTGGAGAAAAGCT
ACTTCATAACACATGGATCAAAAGCACGATCCGAGACCTTCATTAACCTACGGGAGGTCTCAGCCCGGTTCTGCCTGCCCCCCGGAGAGTACCTGGTTGTCCCCTCCACTTTCGAACCCAACCAAAATGGGGACTTTGCTCTGCG
CGTCTACACAGAGAAACGGGCTGACACTCAC----------------------------------------
GTTCAGGAGTCCGATGTGAGCAGCAACTTCAGGAGTATGTTTGAAAAACTCGCTGGAGAGGACAAGGAAATCTCGGTATTCGAGCTCCAGAAAATACTGAACAAAATCATGTCCAAA---------
ATCGGATTCGGGCTGGAGTCGTGCCGCAACATGGTGCATCTGGTGGACAAAGACGGGAACGGAAAGATGGGATTGGTGGAATTCCAGAAACTG---AACAAGGTGCAG---------
AAAATCTATAAAAATAATGACCTGCAGTCGGGAACA-------------------------------------GCTGGTTTTCACCTGAATAATAAACTCTGTCAGATCATCGTTTCTCGTTACTATGACCTCACC------
CTGGACTTTGATAACTTTGTTTCCTGTCTTGTCCGCCTGGAGCTGGTGTTCAAGTTGTTCAATTCTCTCCCCAAGGATGAGGAAGGATTCGCTCAACTCAGCTTGTGC;

END;



BEGIN TREES;

       TREE tree = ((((GASTEROSTEUS_ACULEATUS_ENSGACG00000019985,((((CALLORHINCHUS_MILII_TRANSCRIPTOMECONTIG17290,CHILOSCYLLIUM_PUNCTATUM_CONTIG44567),(SCYLIORHINUS_CANICULA_TRANSCRIPTOMECONTIG67723,RAJA_ERINACEA_TRANSCRIPTOMECONTIG62832)),((XENOPUS_TROPICALIS_NM_001005446,XENOPUS_LAEVIS_NP_001083713),(((MONODELPHIS_DOMESTICA_ENSMODG00000005094,((CERATOTHERIUM_SIMUM_XP_004439596,OVIS_ARIES_NP_001106288),(HOMO_SAPIENS_BC021303,MUS_MUSCULUS_ENSMUSG00000026509))),ORNITHORHYNCHUS_ANATINUS_ENSOANG00000001450),((PELODISCUS_SINENSIS_ENSPSIG00000012616,ANOLIS_CAROLINENSIS_ENSACAG00000003199),GALLUS_GALLUS_ENSGALG00000009360)))),(LEPISOSTEUS_OCULATUS_GENSCAN00000007636,((DANIO_RERIO_ENSDARG00000055592,((GASTEROSTEUS_ACULEATUS_ENSGACG00000019944,OREOCHROMIS_NILOTICUS_ENSONIG00000007266),TAKIFUGU_RUBRIPES_ENSTRUG00000012768)),DANIO_RERIO_ENSDARG00000091699)))),GASTEROSTEUS_ACULEATUS_ENSGACG00000004767),DANIO_RERIO_ENSDARG00000090014),DANIO_RERIO_ENSDARG00000034211);

END;
```

## (*f*) *CAPN3*

```
#NEXUS

BEGIN TAXA;

       DIMENSIONS NTAX = 26;

       TAXLABELS

              'XENOPUS_TROPICALIS_XM_004917239' 'GALLUS_GALLUS_ENSGALG00000009050' 'MELEAGRIS_GALLOPAVO_ENSMGAG00000011468' 'TAENIOPYGIA_GUTTATA_ENSTGUG00000011081' 'PELODISCUS_SINENSIS_ENSPSIG00000011344' 'ANOLIS_CAROLINENSIS_ENSACAG00000015493' 'SUS_SCROFA_ENSSSCG00000004728' 'MUS_MUSCULUS_ENSMUSG00000079110' 'HOMO_SAPIENS_AF127765' 'SARCOPHILUS_HARRISII_ENSSHAG00000007516' 'MONODELPHIS_DOMESTICA_ENSMODG00000017909' 'OVIS_ARIES_EU846598' 'CERATOTHERIUM_SIMUM_XM_004421467' 'GADUS_MORHUA_ENSGMOG00000013176' 'GASTEROSTEUS_ACULEATUS_ENSGACG00000005212' 'TAKIFUGU_RUBRIPES_ENSTRUG00000006900' 'ORYZIAS_LATIPES_ENSORLG00000018135' 'XIPHOPHORUS_MACULATUS_ENSXMAG00000009351' 'DANIO_RERIO_ENSDARG00000041864' 'SCYLIORHINUS_CANICULA_TRANSCRIPTOMECONTIG17411' 'LEPISOSTEUS_OCULATUS_GENSCAN00000022004' 'LATIMERIA_CHALUMNAE_ENSLACG00000003659' 'GADUS_MORHUA_ENSGMOG00000000192' 'ORYZIAS_LATIPES_ENSORLG00000012402' 'GASTEROSTEUS_ACULEATUS_ENSGACG00000009830' 'OREOCHROMIS_NILOTICUS_ENSONIG00000006073' ;
```

```
END;

BEGIN CHARACTERS;

        DIMENSIONS NCHAR = 2220;

        FORMAT

                DATATYPE = DNA


                GAP=-

                MISSING=?

                NOLABELS

        ;


MATRIX

 GGAGGAGGCATTTATTCTGCCATTATCAGCAGGAATCTTCCAATTATT---AAAACCTTTGAGGAACTCAGAAAAAAATGCTTGCAGAGGAACTTTCATTTTCAAGATCGTGAGTTTCCACCAGATGAAAGCTCTCTG------
---
GAATGGAAAAGGCCTCCTGAAATTTGTGAGAATCCCCAGTTTATCCTTGGAGGAGCAAACAGAACTGACATCTGCCAAGGAGATTTAGGGGATTGCTGGTTCCTGGCTGCCATAGCTTGTCTGACACTCAATGAAAAGTCCTCT
TTCGTGTCATTCCTCCAGACCAGAATTTCACAGACAACTATGCTGGAATATTTCATTTTCAGTTCTGGCGTTATGGAGACTGGGTCGATGTGATCATAGATGACTATTTGCCAACCTATAACAATGAACTGGTGTTCACCAAATC
TTCCCAGCGGAATGAGTTTTGGAGTGCTTTGCTGGAAAAGGCGTACGCAAAACTGCATGGCTCCTATGAGGCTCTCAAGGGTGGAAATACCACAGAAGCTATGGAGGACTTCACTGGTGGAGTGACAGAATTTTATGAGCTTAAA
GAAGCACCAAAGGACATGTACAACATAATGAAGAAAGCTTTTGAGAGAGGCTCTCTCATTGGATGTTCAATTGATGGGTCTTCCTGTGGATTTGCACCTCCTAGTAATATGGAGGATGCAATAATGAAAATGATAGAGAGTGTGG
GTGACACACGTCCTGACACAAATGGCAGAGGCCAGCCACCAAAGCCAGTAGCGACCCTTGTCCCTGTTCAGCTGGAGACCCGCATGGCCAATGGACTGGTTAAAGGCCACGCATACTCTGTTACAGGTGTTGAAGAGACAAAGAA
AGGAAAGCCAATCAAACTTGTTCGTCTCAGGAACCCTTGGGGACAAGTGGAATGGAATGGAGCATGGGGTGACAATGCAAAGGAATGGACTATGGTTGATAAGTCGGAAAAGACTCGCCTCCAGCACCAGGTACAGGAAGATGGC
GAGTTTTGGATGTCTTATGATGATTTTATGAAGAATTTCACCAAGGCTGAAATCTGCAATCTGACACCAGATGCACTGGATTCGGACTCACTCCAAGTATGGACAGTGTCTGTAAATGAAGGGAGATGGGTCAGGGGTTGCTCAG
CAGGAGGCTGTCGCAATTATCCAGACACCTACTGGACCAACCCTCAGTATCGTCTTAAGCTGCTGGAGGAGGATGATGACCCTGCTGACAATGAGGTTGTCTGTAGTTTTGTTGTTGCTTTAATGCAGAAGAATCGAAGGAAAGA
TCGAAAGGCAGGAGCCAATCTTTTCACCATTGGTTTTGCAATATATGAGGTTCCAAAAGAGATGCATGGCAACAATCAGCACCTGCAAAAGGATTTCTTCCTCTACAATGCCTCCAAAGCCAAGTGCAAATCATATATCAACATG
AGAGAAGTCTGTCAGCGTTTCCGATTGCCCCCAAGTGAATACGTCATTATCCCGTCTACCTATGAGCCCCACCAGGAAGGAGAGTTCATTCTGAGAGTTTTCTCTGAGAAAAGAAACATTTCAGAAGAAGTTGAAAACAAGATAG
AAGCAGAAAAACCAATTCCTATCATCTTTGTATCGGACAGATCAAACAGCAATAAGGAGCTGAATGTGGATGGCGCCACTGATGAAGATCAGAAAAAAATGGACGCAGAGAAAAAAGATAAGACCAGTACAGACACAGAGACTGA
GGAAGAAAAACAGTTCAGGAATATCTTCCAACAAATTGCAGGAGATGACATGGAAATAAGTGCTGATGAGCTTCAAAGTGTCCTGAACAATGTAGTTAACAAACATAAAACTCTGAAATCCAGTGGATTCATGATTGGCTGCGGG
AGACTCAACCTGCAAGAATTCTATCACCTTTGGCAAAAGATCAAGCAGTGGCAGAAAATCTTTTTGCGTTTTGATTCTGACCAGTCGGGGACCATCAGTAGCTTTGAGATGCGCAATGCCATCAATGAAGCTGGTTTCCATCTGA
ACAATCAGCTGTATGACATTATCACAATGCGCTATGCCAATAAGAGAATGGACCTTGACTTTGACAGTTTCATCTGCTGCTTTGTGCGCCTGGAAGGAATGTTCAGGGCATTCCATGCATTTGACAAAGATGGAGATGGTATCAT
TAAGCTGAATGTGCTGGAGTGGCTGCAGCTTACCATGTATGCT

 ACAGGAGGGATTTATTCTGCCATTATAAGTCGCAACCAGCCCATTATC---AAGACCTATGAAGAGCTTCACAAGAAGTGCCTGGAGGAGAACATTCTCTATGAGGATCCTGATTTTCCACCTAATGAGACTTCCCTC------
------
TGGAAAAGACCACGTGAAATCTGTGAGAATCCACGATTTATTATCGGCGGAGCCAACAGAACAGATATCTGCCAAGGAGAATTAGGCGATTGCTGGTTCCTGGCTGCCATTGCTTGCCTGACACTGAATAAAAAACTGCTCTGCA
GAGTCATACCTCATGACCAGTCCTTCATACAGAACTATGCTGGCATCTTTCACTTCCAGTTTTGGCGCTACGGAGACTGGGTGGATGTCATCATTGATGACTGCCTACCCACATACAACAACCAGCTGGTCTTCACCAAATCCTC
CCAGCGCAATGAGTTCTGGAGTGCTCTCCTGGAAAAGGCCTATGCAAAACTCCATGGATCCTATGAAGCTTTGAAAGGAGGCAACACCACAGAGGCCATGGAGGATTTCACTGGAGGGGTGACAGAGTTCTATGAGATAAAGGAT
GCTCCTAAAGATATCTATAAAATCATGAAACATGCCATTGCCAGAGGATCCCTCATGGCCAGCTCCATTGAT------------------------------------------------------------------------
```

-----------------------------------------------------------------------GAAACCCGGATGTCTTGTGGGCTGGTCAAAGGTCACGCATACTCTGTCACAGCTGTG------------
AAAGGGGAAAAGATGCGTCTGGTAAGGCTGAGAAACCCGTGGGGACAGGTGGAATGGAATGGACCTTGGAGTGATAAATCAGAAGAGTGGAACTTCATTGATGAAGAAGAGAAAACCCGCCTGCAACACAAGATTGCAGAAGATG
GGGAATTCTGGATATCATTTGAAGATTTCATGAGGCATTTCACAAAACTTGAGATCTGTAACCTCACACCTGATACTCTGGAAGCTGATAAGCTTCAGACCTGGACTGTGTCAGTCAACGAAGGACGCTGGGTGAGAGGCTGCTC
AGCTGGAGGGTGCCGCAATTATCCAGATACATTTTGGACCAATCCCCAGTATCGCCTGAAGCTCCTAGAGGAAGATGATGATCCTGAAGATGAAGAGGTTATCTGCAGCTTCCTGGTAGCACTGATGCAGAAAAACAGGAGGAAA
GAACGCAAGCTGGGAGCCAACCTCTACACCATTGGCTTTGCCATCTATGAGGTACCCAAAGAGATGCACGGTACTAAGCACCACTTGCAAAAGGATTTTTTCCTCTACAATGCTTCCAAAGCTAGAAGTAAGACCTATATAAACA
TGCGAGAAATCTCTGAGCGCTTCCGGCTGCCTCCCAGCGAGTACGTTATCATCCCATCCACATACGAACCCCACCAGGAGGGAGAATTCATCCTGAGGGTTTTCTCAGAGAAAAGAAGTCTTTCAGAGGAGGTTGAAAACATGAT
TGAGGCAGATCGCCGT---------------------------------------------------------------------------------------------------------------
GCAAAACGTGAGAAGAGTGAAGAGGAAACTCAGTTCAGGAATATTTTCCGACAGATTGCAGGGGATGACATGGAGATCAATGCCGAAGAACTCAGGAATGTTCTCAACAACGTTGTTAAAAAACATAAG---
CTAAAGACAGAAGGATTTATGATCGGCTCAGGGAAGATCAACTTTGATGAGTTCCGACACCTCTGGGACAAGATCAAAAGCTGGCAGAAAATCTTCAAGCATTACGATGCAGACCATTCAGGAACCATTAACAGCTATGAGATGC
GCAATGCAGTCAAAGATGCAGGGTTTCGGCTGAACAACCAGCTCTACGACATCATCACGATGCGCTATGCTGACAAGAACATGAACATCGACTTTGACAGCTTCATCTGCTGCTTCGTGAGACTGGATGCTATGTTCAGGGCATT
CCATGCCTTTGATAAAGATGGAGATGGAATCATAAAGCTCAACGTCCTGGAGTGGCTGCAGCTCACCATGTACGCC

ACAGGAGGGATTTATTCTGCCATTATAAGTCGCAACCAGCCCATTATC---AAGACCTATGAAGAGCTTCACAAGAAGTGTCTGGAGAAAAACATTCTCTATGAGGATCCTGATTTCCCACCTAATGAGACTTCCCTC------
------
TGGAAGAGACCACGTGAAATTTGTGAGAATCCACGGTTTATTATCGGCGGAGCCAACAGAACAGATATCTGCCAAGGAGAATTAGGCGATTGCTGGTTCCTGGCTGCCATTGCTTGCCTGACACTGAATAAAAAACTGCTCTGCA
GAGTCATACCTCATGACCAGTCCTTCATACAGAACTATGCTGGCATCTTTCACTTCCAGTTTTGGCGCTATGGAGACTGGGTGGATGTCATCATTGATGACTGCCTACCCACATACAACAACCAGCTGGTCTTCACCAAATCCTC
CCAGCGGAATGAGTTCTGGAGTGCTCTCCTGGAAAAGGCCTATGCAAAACTCCATGGATCCTATGAAGCCTTGAAAGGAGGCAACACCACAGAGGCGATGGAGGACTTCACTGGAGGAGTGACAGAGTTCTATGAGATAAAGGAT
GCTCCTAAAGATATCTATAAAATCATGAAACATGCCATTGCCAGAGGATCCCTCATGGCCAGCTCCATTGAT-------------------------------------------------------------------------
-----------------------------------------------------------------------GAAACTCGGATGTCTTGTGGACTGGTCAAAGGTCACGCATACTCTGTTACAGCCGTG------------
AAAGGGGAAAAGATCCGTCTGATAAGGCTGAGAAACCCGTGGGGACAGGTGGAGTGGAATGGACCTTGGAGCGATAAATCAGAAGAGTGGAACTTCATTAAAGAAGAAGAGAAAATCCGTCTGCAACACAAGATTTTGGAAGATG
GGGAATTCTGGATATCATTTGAAGATTTCATGAGGCATTTCACAAAACTTGAGATCTGTAACCTCACACCTGATACTCTAGAAGCTGATAAGCTTCAGACCTGGACTGTGTCAGTCAATGAAGGACGCTGGGTGAGAGGCTGCTC
AGCTGGAGGGTGCCGCAATTATCCAGATACATTTTGGACCAATCCCCAGTATCGCTTGAAGCTCCTAGAGGAAGATGATGATCCCGAAGATGAAGAGGTTATCTGCAGCTTCCTGGTAGCACTGATGCAGAAAAACAGGAGGAAA
GAACGCAAGCTGGGAGCCAACCTCTACACTATTGGCTTTGCCATCTATGAGGTACCCAAAGAGATGCACGGTACTAAGCACCACTTGCAAAAGGATTTTTTCCTCTACAATGCTTCCAAAGCTAGAAGTAAGACCTATATAAACA
TGCGAGAAATCTCTGAGCGCTTCCGGCTGCCTCCCAGCGAGTATGTTATCATCCCATCCACATACGAACCCCACCAGGAGGGAGAATTCATCCTGAGGGTTTTCTCAGAGAAAAGAAGTCTTTCAGAGGAAGTTGAAAACATGAT
TGAGGCAGATCGCCGT---------------------------------------------------------------------------------------------------------------
GCAAAACGTGAGAAGAGTGAAGAGGAAACACAGTTCAGGAATATTTTCCGACAGATTGCAGGGGATGACATGGAGATCAATGCTGAAGAACTCAGGAATGTTCTCAACAATGTTGTTAAAAAACATAAG---
CTAAAGACAGAAGGATTTATGATTGGCTCAGGGAAAATCAACTTTGATGAGTTCCGACACCTCTGGGACAAGATCAAAAGCTGGCAGAAAATCTTCAAGCATTACGATGCTGACCATTCAGGAACCATTAACAGCTATGAGATGC
GCAATGCAGTCAAAGATGCAGGGTTTCGGCTGAACAACCAGCTCTATGACATCATCACGATGCGCTACGCTGACAAGAACATGAACATCGACTTTGACAGCTTCATCTGCTGCTTTGTGAGACTGGATGCTATGTTCAGGGCATT
CCATGCCTTCGATAAAGATGGAGATGGAATCATAAAACTCAATGTCCTGGAGTGGCTGCAGCTCACCATGTACGCC

ACAGGGGGAATTTATTCTGCCATTATAAGTCGCAATCAGCCCATTATC---AAGACCTATGAAGAGCTTCATAAGAAGTGCCTGGAGAAAAACATTCTCTATGAAGATCCTGATTTCCCACCAAATGAGTCGTCCCTT------
------
TGGAAAAGACCACGTGAAATCTGTGAGAATCCACGGTTTATTATTGGTGGAGCCAATAGAACAGATATCTGCCAAGGAGAATTAGGTAACTGCTGGTTTCTGGCTGCCATTGCTTGCCTGACACTGAATAAAAAACTACTCTGTA
GAGTCATACCTCACGACCAGTCCTTTATACAAAACTATGCTGGCATCTTTCACTTCCAGTTCTGGCGCTATGGAAACTGGGTGGATGTCGTCATCGATGACTGCTTACCCACCTACAACAACCAGCTGGTCTTTACCAAGTCCTC
CCAGCGCAACGAGTTCTGGAGCGCCCTCCTGGAGAAGGCCTATGCAAAACTCCATGGGTCATATGAAGCTTTGAAGGGAGGCAACACCACTGAAGCCATGGAGGACTTCACTGGAGGAGTCACAGAGTTCTATGAGATAAAGGAT
GCACCTAAAGATATCTATAAAATCATGAAACATGCTATTGACAGAGGATCGCTCATGGCCAGTTCCATTGAT-------------------------------------------------------------------------
-----------------------------------------------------------------------GAGACACGGATGTCCTGCGGGCTGGTCAAGGGGCACGCCTACTCCGTGACGGCCGTG------------
AAAGGTGAAAAAATACGTCTGGTAAGGCTGAGAAACCCCTGGGGACAGGTGGAATGGAATGGAGCCTGGAGTGACAAGTCAGAGGAGTGGGACTCTGTTAATGAAGCGGAGAAAATCCGCCTGCAGCACAAGGTCGTGGAGGATG
GGGAGTTCTGGATATCGTTTCAAGATTTCATGAGGCACTTCACAAAGCTTGAGATCTGTAACCTCACACCTGATACTCTGGAAGTGGATAAATTCCAGACCTGGACTGTGTCAGTCAATGAAGGACGCTGGGTGAGAGGCTGCTC
AGCTGGAGGTTGCCGCAATTTTCCAGATACATTTTGGACCAATCCCCAGTATCGCCTGAAGCTCCTGGAGGAAGATGATGATCCTGAGGATGAAGAGGTTATCTGCAGCTTCCTTGTTGCACTGATGCAGAAAAACAGGAGGAAA
GAGCGCAAGCTGGGAGCCAACCTGTACACCATTGGCTTTGCCATCTATGAGGTGACAGACTCTATGCATGGTACTAAGCACCACTTGCAGAAGGATTTTTTCCTCTACAATGCCTCCAAAGCTAGAAGTAAGACCTACATAAACA
TGCGAGAAATCTCTGAGCGCTTCCGGTTACCTCCCAGCGAGTACGTCATCATCCCATCAACATATGACCCCCACCAGGAGGGAGAATTCATCCTCAGGGTCTTCTCAGAGAAAAGAAGTCTTTCAGAAGAGGAGGAAAACAGAGT
AGCAAATTCAGAACGT---------------------------------------------------------------------------------------------------------------
ACAAAACATGAGGAGACTGAAGAGCAAAAACAGTTCAGGAATATTTTCCGACAGATTGCAGGGGATGACATGGAGATCAATGCTGAGGAACTTAGGAATGTTCTCAATAATGTCGTAAAAAAACATAAG---
CTAAGGTCAGAAGGGTTTATGATTGGCTCAGGGAAGATAAACTTTGATGAATTTCGACATCTCTGGGACAAGATTAAAAGCTGGCAGAAAATTTTCAAGCGTTATGACACGGATCATTCAGGAACCATTAACAGCTACGAGATGC
GCAATGCAGTCAAAGATGCAGGGTTTCGGCTGAACAACCAGCTCTACGACATCATCACCATGCGCTACGCCGACAAGAACATGAACATCGACTTCGACAGCTTCATCTGCTGCTTCGTGCGCCTGGACGCCATGTTCCGGGCATT
CCACGCATTTGACAAAGATGGAGATGGCATCATTAAGCTCAATGTCCTGGAGTGGCTGCAGCTCACCCTGTACGCC

CCTGGGGGAATCTATTCTGCTATCATCAGTCGCAACCAACCCATTATT---AAGACCTTTGAAGAACTTCACAAGAAATGCCTGGAGAAGAAGATTCTTTATGAGGATCCTGATTTCCCACCTAATGAGAGCTCCCTC------
------
TGGAAAAGACCCCCAGAAATCTGTGAGAACCCACGCTTTATAATTGGTGGAGCCAACCGAACAGATATTTGTCAAGGAGAATTAGGTGACTGCTGGTTCCTGGCTGCCATTGCTTGCCTGACATTGAATGAAAAGCTGCTCTGCC
GAGTCATACCTCACGATCAGACCTTCATAAAAAACTATGCTGGGATCTTTCACTTCCAGTTCTGGCGCTATGGAGACTGGGTGGACGTTGTTGTTGACGACTTTTTACCAACATATAACAACCAGCTGGTCTTCACCAAATCCTC
CCAGCGCAATGAATTCTGGAGTGCTCTGCTGGAAAAGGCTTATGCAAAACTCCACGGATCTTATGAGGCGTTGAAAGGAGGCAATACTACAGAGGCTATGGAAGATTTCACAGGTGGAGTGACTGAGTTCTATGAGATAAAAGAT
GCACCGAAAGATATATACAAAATCATGAAACATGCCAGTGACAGAGGATCACTCATGGCCTGCTCCATTGAT-------------------------------------------------------------------------
-----------------------------------------------------------------------GAGACACGCATGACTTGTGGACTAGTCAAAGGACATGCCTATTCAGTTACTGCAGTG------------
AAAGCAGGAAAAATAAAGCTAGTGCGGCTGAGGAACCCGTGGGGACAGGTGGAATGGAAAGGAGCCTGGAGCGACCGTTCAGATGAATGGACCTTAATTGACAAAGCAGAGAAAATCCGACTACAGCACAATATTGAAGAGGATG
GGGAGTTCTGGATGTCATTTGAAGATTTCATGAGGTACTTCACCAAGCTTGAGATCTGTAACATCACACCTGATACTCTGGAGGCTGATAAGCTCCACACTTGGACTGTTTCAGTCAATGAAGGGCGTTGGGTGAGAGGCTGCTC
TGGAGGAGGTTGTCGTAATTATCCAGATACATTTTGGACCAATCCTCAGTATCGCTTGAAGCTCCTGGAAGAGGATGACGATCCTGAAGATAATGAGGTTGTCTGCAGCTTCCTGGTTGCACTGATGCAGAAAAACAGGAGAAAA
GAGCGCAAACTCGGAGCCAACCTCCTCACTATTGGCTTCGCCATTTATGAGGTGCCAAAAGAGATGCATGGTAACAAGCAGCATTTACAAAAGGATTTTTTCCTTTACAATGCCTCCAAAGCTAGAAGTAAGTCATATATAAACA
TACGAGAAGTCTCCGAACGCTTCCGGCTGCCTCCCAGTGAGTATGTCATCATCCCATCAACATATGAACCTCACCAGGAGGGGGAGTTCATCCTGAGAGTCTTCTCAGAAAAAAGAAACCTTTCAGAGGAAGTTGAAAACAAGAT
AGAAGTAGAACGTCCC---------------------------------------------------------------------------------------------------------------
ACGAAAGACAAGGACAGTGAAGAAGAGGAGCAGTTCAGGAATATATTCCAGCAGATTGCTGGAGATGACATGGAGATCAGTGCTGATGAACTCAGAAGTGTTCTGAATAATGTTCTGAAAAAACACAAA---
CTGAAGACAGAAGGGTTTATGATTGGTTCAGGGAAGATAAATTTCCAAGAGTTTCAACATCTCTGGAATAAGCTGAAAAGCTGGCAGAAAATTTTCAAGCATTATGACACAGATCATTCTGGCACCATTAACAGTTATGAGATGC
GTAATGCAGTCAAAGATGCAGGATTCTCCCTGAACAAACAGCTCTATGACATCATTACAATGCGCTATGCTGACAAAAACATGAACATTGAGTTTGACAGCTTTATCTGCTGCTTTGTGCGACTGGAAGGAATGTTCAGAGCATT
CCATGCCTTTGATAAAGATGGAGATGGTATAATTAAACTCAACGTCTTGGAGTGGCTGCAACTCACCATGTATGCT

GCAGGAGGGATCTACTCCGCCATCCTCAGCCGCAACCAGCCCATCATC---AAGACTTTTCACGAGCTCCACAAGAAATGTCTCGAAAAGAAGATTCTTTATGAGGATCCAGATTTTCCAGCAAATGAGTCCTCCCTT------
------
TGGAAGAGGCCACCAGAAATTTGTGAGAATCCACGCTTCATAATTGGTGGAGCCAACAGGACTGACATTTGTCAAGGGGAACTAGGCGATTGCTGGTTCCTAGCTGCCATTGCTTGCCTAACCTTGAATGAAAAACTGCTCTTCC
GGGTCATACCACACGATCAGACCTTCATAAAAGATTATGCTGGGATCTTCCATTTCCAGTTTTGGCGATATGGAAGTTGGGTGGAAGTTATCATTGATGATCGATTACCAACATATGGCAAACAACTGGTCTTTACCAAATCCTC
CCAGCAAAATGAGTTCTGGAGTGCTTTACTAGAAAAAGCCTATGCAAAGCTTCATGGGTCTTATGAGGCATTGAAAGGAGGCAACACCACAGAAGCCATGGAGGACTTCACCGGAGGTGTAACCGAGTTCTATGAGATAAAGGAC
GCTCCTAAAGATATCTATAAAATCATGAAGCATGCCATCGAGAGAGGGTCACTCATGGCCTCCTCCATTGAG-------------------------------------------------------------------------
-----------------------------------------------------------------------GAGAGACGCATGGCTAATGGATTGGTTCTAAGCCATGCCTATTCAGTGACAGGAGTG------------
AAACGGGAGAAGCTAAAACTGATACGGTTAAGAAATCCTTGGGGACAAGTAGAATGGAATGGAGCTTGGAGTGATAGTTCAGATGAATGGAATGTTATTGATGGTGCAGAGAAGACCCGACTACAGCATAAAGTTTCAGAGGATG
GAGAGTTCTGGATATCTTTGCAAGATTTCCTGAGATACTTCACAAAGCTTGAAATATGTAATATCACACCTGATGCTCTAGAGGGTGATAAGCTTCAAACATGGACAGTTTCTGTCACTGAAGGGCGATGGGTGCGAGGCTGCAC
AGCTGGAGGCTGCCGTAACTATCCAGACACATTTTGGACCAATCCTCAGTATCGCTTGAGACTTCTGGAAGAAGACGATGATCCAGAAGACAGTGAAGTTGTGTGCAGTTTCTTAGTTGCACTGATGCAGAAGAATAGGAGGAAA
GAACGCAGACTAGGAGCCAATCTTCTCACAATTGGCTTTGCCATCTATGAG------------
ATGCAGGGTAACAATATGCACTTGCAAAGGGATTTTTTCCTCTACAATGCTTCAAAAGTAAAATGCAAGATGTATATAAATATGAGGGAAGTTGCTGAACGCTTCCGGTTGCCTCCCAATGAGTATGTCATCATCCCATCTACAT
TCGAACCTCACCAAGAAGGGGAGTTTCTCCTTCGAGTTTTCTCAGAAAAAAATAAGTCTTTCAGAGGAAGTTGAAAACAAGATTGCAGTAGGACAACCT-----------------------------------------------
----------------------------------------------------------------
AGAGATAATAAGGAAACTGAGGAAGACAAACAGTTCAGAAATATTTTCCGGCAGATTGCTGGAGATGACATGGAGATCAGTGCTGACGAACTCAGGAATGTCCTTAACAATGTTCTGAAAAAGCATAAG---
TTGAAGACAGAAGGGTTCATGATCGGTTCTGGAAAAATAAACCTTGAAGAGTTTCAACATCTTTGGGATAAGATTAAAAGCTGGCAGAAAATTTTCAAACACTATGACACCGATCACTCAGGAACCATTAACAGCTATGAAATGC
GTAATGCAGTGAAAGATGCAGGATTTCAGTTGAATAACCAATTGTATGACATCATTACAATGCGCTATGCTGACAGAAATATGAACATTGAGTTTGACAGCTTCATCTGTTGTTTTGTGAGGTTAGAGGGAATGTTCCGTGCATT
TAATGCCTTCGACAAAGATGGAGATGGTATTATTAAACTGAATGTCTTAGAGTGGCTACAGCTCACCATGTATGCC

CCAGGTGGTAAATACTCAGCCATCATCAGCCGCAATTTTCCCATTATT---AAGACATTTGAGCAGCTTCACAAGAAATGCCTAGAAAAGAAGGTTCTTTATCTGGATCCTGAGTTCCCACCGGACGAGACCTCGCTC------
------
TGGAAGAGACCTCCGGAAATTTGTGAGAATCCCCGATTTATCATTGGTGGAGCCAACAGAACTGACATCTGTCAAGGAGACCTAGGGGACTGCTGGTTTCTGGCAGCCATCGCTTGCCTGACCCTCAACAAGCGTCTGCTTTTCC
GAGTCATACCCCATGATCAGAGTTTCACCGAAAACTACGCAGGGATCTTTCACTTCCAGTTCTGGCGCTACGGAGACTGGGTGGACGTGGTTATTGATGACTGCCTGCCAACTTACAACAATCAACTGGTTTTCACCAAATCCAA
CCACCGCAATGAGTTCTGGAGTGCTCTGCTGGAGAAGGCTTATGCTAAGCTCCATGGTTCATACGAAGCCCTGAAAGGTGGAAACACTACAGAGGCCATGGAGGACTTCACAGGAGGAGTGACAGAGTTTTTTGAAATCAAAGAT
GCTCCCAGAGACATGTACAAGATCATGAAGAAAGCCATCGAGAGGGGCTCCCTCATGGGCTGCTCCATTGAT-------------------------------------------------------------------------
-----------------------------------------------------------------------GAGACAAGAATGGCCTGTGGGCTGGTCAAAGGCCACGCCTATTCGGTTACTGGGCTG------------
AAGGGTGAAAAGGTGAAGCTTGTGCGGCTGCGGAACCCCTGGGGCCAGGTGGAGTGGAATGGCTCCTGGAGTGACAGTTGGAAGGACTGGAGCTTTGTGGACAAAGATGAGAAGGCCCGTCTGCAGCACCAGGTCACTGAGGATG
GAGAGTTCTGGATGTCTTATGATGATTTCATCTACCATTTCACAAAGCTGGAGATCTGCAACCTCACAGCTGATGCCCTGGAGTCCGACAAGCTTCAGACTTGGACAGTGTCTGTGAACGAGGGCCGCTGGGTGAGGGGCTGCTC
TGCCGGAGGCTGTCGCAACTTCCCAGACACTTTCTGGACCAACCCACAGTACCGTCTGAAGCTCCTGGAGGAGGATGATGACCCTGACGACTCCGAGGTGATCTGCAGCTTCCTGGTAGCACTGATGCAGAAGAACCGGCGGAAG
GACCGGAAGCTGGGGGCCAACCTCTTCACCATCGGCTTCGCCATCTACGAGGTCCCCAAAGAGATGCATGGAAACAAGCAGCACTTGCAGAAGGACTTCTTCCTGTACAATGCCTCCAAGGCTAGGAGCAGAACCTACATTAACA
TGCGGGAGGTGTCCGAGCGCTTCCGCCTGCCCCCCAGCGAGTACGTCATTGTGCCCTCCACCTATGAGCCCCACCAGGAGGGGGAATTCATCCTCCGGGTCTTCTCCGAAAAGAGGAACCTCTCTGAGGAAGTTGAAAATACCAT

```
CTCTGTGGATCGACCA---------------------------------------------------------------------------------------------------------------
AGCAACGACCAGGAAAGTGAGGAACAGCAGCAATTCCGGAACATTTTCAGGCAGATAGCAGGCGATGACATGGAGATCTGTGCAGATGAGCTCAAGAATGTCCTTAACAGAGTGGTGAACAAACATAAG---
CTGAAGACTGAAGGGTTCATGATTGGCTCTGGAAGACTGAACCTGCAAGAGTTCCACCACCTCTGGAAGAAGATTAAATCCTGGCAGAAAATTTTCAAGCATTATGACACCGACCAATCCGGCACCATCAACAGCTATGAGATGC
GAAATGCAGTCAATGACGCAGGCTTCCACCTCAACAACCAGCTCTATGACATCATCACCATGCGGTACGCAGACAAGTACATGAACATCGACTTTGACAGTTTTATCTGCTGCTTCGTCAGGCTGGAGGGCATGTTCAGAGCTTT
TAATGCCTTTGACAAGGATGGAGATGGTATCATCAAACTCAATGTTCTAGAGTGGCTGCAGCTCACCATGTATGCC

 CCAAGTGGCATCTATTCGGCCATCATTAGCCGCAATTTTCCGATCATC---AAGACATTTGAGCAGCTCCGCAGGAAGTGCCTAGAGAAGAAAGTTCTTTATCTAGACCCCGAGTTCCCACCGGATGAGACCTCTCTC------
------
TGGAAGAGACCTCCGGAAATTTGTGAGAATCCCCGGTTTATCATTGGTGGAGCCAACAGGACTGACATCTGCCAAGGAGATCTAGGGGACTGCTGGTTTCTTGCAGCCATCGCCTGCCTGACCCTGAATGAGCGGCTGCTTTTCC
GAGTTATACCCCATGATCAAAGTTTCACTGAAAACTACGCAGGGATCTTCCACTTCCAGTTCTGGCGCTATGGAGACTGGGTAGATGTGGTCATTGACGACTGTCTGCCAACATACAACAATCAGCTGGTTTTCACCAAATCCAA
CCACCGCAATGAGTTCTGGAGTGCTCTCCTTGAGAAGGCTTATGCCAAGCTCCATGGCTCCTATGAAGCTCTGAAAGGTGGGAATACCACAGAAGCCATGGAGGACTTCACAGGAGGGGTGACAGAGTTTTTTGAGATCAAGGAT
GCTCCGAGTGACATGTACAAGATTATGAGGAAAGCTATCGAGAGAGGCTCCCTCATGGGCTGCTCCATTGAC-------------------------------------------------------------------------
-----------------------------------------------------------------------GAAACAAGAATGGCCTGTGGGTTGGTGAAAGGGCATGCCTATTCAGTCACTGGGCTG------------
AAAGGTGAGAAGGTGAAGTTGGTGCGGCTGCGGAACCCCTGGGGCCAGGTGGAGTGGAACGGCTCTTGGAGTGATGGTTGGAAGGACTGGAGCTTTGTAGACAAAGATGAGAAGGCCCGTCTACAGCACCAGGTTACTGAGGATG
GAGAGTTCTGGATGTCATATGATGACTTCGTCTACCATTTCACGAAGCTGGAGATCTGCAACCTCACAGCTGACGCCCTGGAGTCCGATAAGCTTCAGACCTGGACGGTGTCTGTAAACGAGGGCCGCTGGGTGAGGGGCTGTTC
TGCTGGAGGCTGCCGAAACTTCCCAGACACTTTCTGGACCAACCCGCAGTACCGTCTCAAGCTTCTGGAGGAAGACGATGACCCTGAGGACTCTGAGGTAATCTGCAGCTTCCTCGTGGCTCTGATGCAGAAGAACCGGCGCAAG
GACCGGAAGCTGGGGGCCAACCTCTTCACTATTGGCTTCGCCATCTACGAGGTTCCCAAAGAGATGCACGGGAATAAGCAACACCTGCAGAAGGACTTCTTCTTGTACAACGCCTCCAAGGCCAGAAGCAAAACCTACATCAACA
TGCGGGAGGTGTCCCAGCGCTTCCGCCTGCCACCCAGCGAGTATGTCATCGTACCCTCCACCTACGAGCCCCACCAGGAGGGGGAATTCATCCTCCGAGTCTTCTCCGAAAAGAGGAATCTCTCTGAGGAAGCTGAAAATACAAT
CTCTGTGGATCGGCCA---------------------------------------------------------------------------------------------------------------
GGCCACGACCAGGAAAGTGAGGAGCAGCAGCAATTCCGGAACATCTTCAGGCAGATCGCAGGCGACGACATGGAGATCTGTGCAGATGAACTCAAGAATGTCCTCAACACAGTGGTGAACAAACACAAG---
CTGAAGACACAAGGGTTCATGATAGGCTCTGGAAGACTGAATCTTCAAGAGTTCCATCACCTCTGGAAAAAGATCAAGGCCTGGCAGAAAATCTTCAAGCACTATGACACAGACCATTCCGGTACCATCAATAGCTATGAGATGC
GAAATGCAGTCAATGATGCAGGCTTCCATCTCAACAGCCAACTCTATGACATCATCACCATGCGCTATGCGGACAAACACATGAACATCGACTTTGACAGCTTCATCTGCTGCTTCGTCAGGCTGGAAGGGATGTTCAGAGCTTT
TAACGCATTTGACAAGGATGGCGATGGTATCATCAAACTGAATGTACTTGAGTGGCTGCAGCTTACCATGTATGCC

 CCAAGTGGCATCTATTCAGCCATCATCAGCCGCAATTTTCCTATTATC---AAGACATTCGAGCAACTTCACAAGAAATGTCTAGAAAAGAAAGTTCTTTATGTGGACCCTGAGTTCCCACCGGATGAGACCTCTCTC------
------
TGGAAGAGACCTCCGGAAATTTGCGAGAATCCCCGATTTATCATTGATGGAGCCAACAGAACTGACATCTGTCAAGGAGAGCTAGGGGACTGCTGGTTTCTCGCAGCCATTGCCTGCCTGACCCTGAACCAGCACCTTCTTTTCC
GAGTCATACCCCATGATCAAAGTTTCATCGAAAACTACGCAGGGATCTTCCACTTCCAGTTCTGGCGCTATGGAGAGTGGGTGGACGTGGTTATAGATGACTGCCTGCCAACGTACAACAATCAACTGGTTTTCACCAAGTCCAA
CCACCGCAATGAGTTCTGGAGTGCTCTGCTGGAGAAGGCTTATGCTAAGCTCCATGGTTCCTACGAAGCTCTGAAAGGTGGGAACACCACAGAGGCCATGGAGGACTTCACAGGAGGGGTGGCAGAGTTTTTTGAGATCAGGGAT
GCTCCTAGTGACATGTACAAGATCATGAAGAAAGCCATCGAGAGAGGCTCCCTCATGGGCTGCTCCATT----------------------------------------------------------------------------
-----------------------------------------------------------------------GAGACAAGAATGGCCTGCGGGCTGGTCAGAGGTCACGCCTACTCTGTCACGGGGCTG------------
AAAGGTGAGAAAGTGAAGCTGGTGCGGCTGCGGAATCCGTGGGGCCAGGTGGAGTGGAACGGTTCTTGGAGTGATAGATGGAAGGACTGGAGCTTTGTGGACAAAGATGAGAAGGCCCGTCTGCAGCACCAGGTCACTGAGGATG
GAGAGTTCTGGATGTCCTATGAGGATTTCATCTACCATTTCACAAAGTTGGAGATCTGCAACCTCACGGCCGATGCTCTGCAGTCTGACAAGCTTCAGACCTGGACAGTGTCTGTGAACGAGGGCCGCTGGGTACGGGGTTGCTC
TGCCGGAGGCTGCCGCAACTTCCCAGATACTTTCTGGACCAACCCTCAGTACCGTCTGAAGCTCCTGGAGGAGGACGATGACCCTGATGACTCGGAGGTGATTTGCAGCTTCCTGGTGGCCCTGATGCAGAAGAACCGGCGGAAG
GACCGGAAGCTAGGGGCCAGTCTCTTCACCATTGGCTTCGCCATCTACGAGGTTCCCAAAGAGATGCACGGGAACAAGCAGCACCTGCAGAAGGACTTCTTCCTGTACAACGCCTCCAAGGCCAGGAGCAAAACCTACATCAACA
TGCGGGAGGTGTCCCAGCGCTTCCGCCTGCCTCCCAGCGAGTACGTCATCGTGCCCTCCACCTACGAGCCCCACCAGGAGGGGGAATTCATCCTCCGGGTCTTCTCTGAAAAGAGGAACCTCTCTGAGGAAGTTGAAAATACCAT
CTCCGTGGATCGGCCA---------------------------------------------------------------------------------------------------------------
GGCAGCGATCAGGAAAGTGAGGAACAGCAACAATTCCGGAACATTTTCAAGCAGATAGCAGGAGATGACATGGAGATCTGTGCAGATGAGCTCAAGAAGGTCCTTAACACAGTCGTGAACAAACACAAG---
CTGAAGACACACGGGTTCATGATTGGCTCTGGAAAGCTCAACCTGCAGGAGTTCCACCACCTCTGGAACAAGATTAAGGCCTGGCAGAAAATTTTCAAACACTATGACACAGACCAGTCCGGCACCATCAACAGCTACGAGATGC
GAAATGCAGTCAACGACGCAGGATTCCACCTCAACAACCAGCTCTATGACATCATTACCATGCGGTACGCAGACAAACACATGAACATCGACTTTGACAGTTTCATCTGCTGCTTCGTTAGGCTGGAGGGCATGTTCAGAGCTTT
TCATGCATTTGACAAGGATGGAGATGGTATCATCAAGCTCAACGTTCTGGAGTGGCTGCAGCTCACCATGTATGCC

 TCTGGGGGTATCTATTCAGCTATTATCAGCCGAAATTTCCCTATTATT---AAGACTTTTGAACAACTACACAAGAAATGTCTAGAAAAGAAGGTTCTGTATGTGGATCCTGATTTTCCTCCCAATGAGACTTCTCTA------
------
TGGAAGAGACCACCGGAAATCTGCGAGAATCCCAGGTTTATCATTGGTGGAGCCAACAGAACTGACATCTGCCAAGGAGATTTAGGGGACTGCTGGTTCCTGGCTGCCATTGCCTGCTTGACCCTGAATGAGCGACTACTTTTCC
GCGTTATCCCCCATGATCAAACTTTCACTGAAAACTATGCTGGGATTTTTCATTTCCAGTTCTGGCGTTATGGAGACTGGGTGGATGTGGTTATTGATGACTGCCTCCCAACCTATAACAACCAGCTGGTCTTCACCAAATCCAA
CCATCGCAATGAGTTTTGGAGTGCTCTGTTGGAAAAGGCCTACGCAAAGCTCCATGGCTCTTATGAAGCCCTGAAAGGTGGTAATACCACAGAAGCTATGGAGGACTTCACTGGAGGAGTGACAGAATTCTTTGAGATCAAGGAT
GCTCCCAGAGACATGTACAAGATCATGAAGAAAGCTATTGAGAGGGGTTCCCTCATGGGCTGCTCCATTGAT-------------------------------------------------------------------------
-----------------------------------------------------------------------GAGACACGAATGACCTGCGGGCTGGTCAAAGGTCATGCATATTCAGTCACTGGACTA------------
```

AAAGGAGAGAAGGTGAAGTTGGTGCGGCTGAGGAACCCGTGGGGTCAGGTGGAATGGAATGGCTCCTGGAGTGATGGCTGGAAGGACTGGGTCCTGATTGAAAAAGAGGAGAAATCTCGCTTGCAGCACCAGGTGACAGAAGATG
GAGAGTTCTGGATGTCCTATGAAGATTTCATGTATCATTTCACAAAGCTTGAAATCTGCAACCTCACAGCTGATGCCCTGGAATCTGACAAGCTCCAGACCTGGACTGTCTCTGTGAATGAGGGCCGCTGGGTGCGGGGATGCTC
TGCTGGAGGCTGTCGCAACTTTCCAGACACTTTTTGGACCAACCCTCAGTATCGCCTGAAACTTCTGGAAGAAGATGATGACCCTGAAGACACTGAGGTTGTCTGCAGCTTCCTAGTGGCCCTGATGCAAAAGAATAGGAGGAAG
GATCGGAAACTGGGAGCCAACCTTTTCACCATTGGTTTTGCTATCTATGAGGTGCCAAAAGAGATGCATGGGAACAAACAACATCTACAGAAGGATTTTTTTCTGTACAACGCCTCCAAAGCCCGGAGTAAAACCTACATCAACA
TGCGCGAAGTGTCTGAGCGCTTCCGCCTGCCGCCCAGCGAGTATGTCATCGTCCCCTCCACTTACGATCCACACCAGGAGGGAGAATTCATCCTTCGCGTCTTTTCTGAAAAAAGGAACCTCTCTGAAGATGTAGAAAATACAAT
TGCTGTGGATCATCCA----------------------------------------------------------------------------------------------------------------
GGTAACGACCGGGAGAGCGAGGAGCAGGTGCAATTCAGGAACATTTTTCGGCAGATTGCGGGTGATGACATGGAAATTTGTGCTGATGAACTCAAGAATGTTCTCAACACAGTGGTGAATAAACATCAA---
TTGAAGATAGATGGATTCATGATTGGTTCTGGACGGCTGAACCTGCAGGAATTTCACCACCTCTGGAAGAAGATCAAGAGCTGGCAGAAAATCTTCAAACACTATGACACAGACCACTCCGGTACCATCAACAGCTATGAGATGC
GCAATGCTGTGAATGATGCGGGCTTCCACCTCAACAGTCAGCTCTACGATATTATCACCATGCGTTATGCAGACAAGCATATGAACATCAACTTTGACAGCTTCATCTGCTGCTTTGTACGGCTGGAGGGCATGTTCAGAGCTTT
TCAAGCTTTTGACAAGGATGGAGACGGCATCATTAAGCTCAATGTTCTAGAGTGGCTGCAGCTTACAATGTATGCC

ACTGGGGGCATCTATTCAGCTATTATCAGTCGGAATTTCCCTATTATT---AAGACTTTTGAGCAGCTACACAAGAAATGTCTAGAAAAGAAGGTTCTGTATCTGGATCCTGATTTCCCCCCCGATGAGACCTCTCTG------
------
TGGAAGAGACCACCGGAAATCTGCGAGAATCCCAGGTTTATCATTGGTGGAGCCAACAGAACTGACATCTGCCAAGGCGATTTAGGGGATTGCTGGTTCCTGGCTGCCATTGCCTGTCTGACCCTGAATGAGCGACTACTTTTCC
GAGTTATCCCCCATGATCAGACTTTCACTGAAAACTATGCTGGGATCTTCCATTTCCAGTTCTGGCGCTATGGAGACTGGGTAGATGTGGTTATTGATGACTGCCTCCCAACCTATAACAACCAGCTGGTCTTCACCAAATCCAA
CCATCGCAATGAATTTTGGAGTGCTCTGCTGGAGAAGGCCTATGCAAAGCTCCATGGCTCTTACGAAGCCCTGAAAGGTGGTAACACCACAGAAGCGATGGAAGACTTCACTGGAGGAGTGACAGAGTTCTTCGAGATCAGGGAT
GCTCCCAGAGACATGTACAAGATCATGAAGAAAGCTATTGAGAGGGGTTCCCTCATGGGCTGCTCCATTGAT--------------------------------------------------------------------------
-----------------------------------------------------------------------AGGTACCTCGCTCCATTTGGGCATTTCATTAACAATTGTTCATCGATTTGCTTTTTG------------
AAAGGAGAGAAGGTGAAGCTGGTACGACTGCGGAACCCCTGGGGTCAGGTGGAATGGAATGGCTCCTGGAGTGATGGCTGGAAGGACTGGGTCCTGATTGACAAAGAGGAGAAATCTCGCTTGCAGCATCAGGTGACAGAAGATG
GGGAGTTCTGGATGTCCTATGAAGATTTCATGTATAATTTCACAAAGCTTGAGATCTGCAACCTCACAGCTGATGCCCTGGAATCTGACAAGCTCCAGACCTGGACTGTGTCTGTGAATGAGGGACGATGGGTGCGGGGATGTTC
TGCAGGAGGTTGCCGCAACTTTCCAGACACTTTTGGACCAATCCTCAGTATCGCTTGAAACTTCTGGAGGAAGATGATGACCCTGAAGACAATGAGGTTGTCTGCAGCTTCCTGGTGGCCCTGATGCAAAAGAATAGGAGGAAA
GACAGAAAATTGGGAGCCAACCTCTTCACCATTGGCTTCGCCATCTATGAGGTGCCAAAAGAGATGCATGGGAACAAACAGCACCTTCAAAAGGATTTCTTCTTGTACAACGCCTCCAAAGCTCGGAGTAAAACCTATATCAACA
TGCGTGAAGTATCCGAGCGCTTCCGCCTGCCCCCCAGTGAATATGTCATCGTCCCCTCCACCTACGATCCCCACCAGGAAGGAGAATTCATCCTTCGGGTCTTCTCTGAAAAGAGGAACCTCTCTGAAGATGTCGAAAATACAAT
TGCTGTGGACCATCCA----------------------------------------------------------------------------------------------------------------
GGCAACGACCGGGAGACCGAGGAGCAGGTGCAATTCAAGAATATTTTTCGACAGATTGCGGGTGATGATATGGAAATTTGTGCTGATGAACTCAAGAATGTTCTCAACACAGTGGTGAACAAACATAAA---
TTGAAGATAGAGGGATTCATGATTGGTTCTGGGCGGCTGAACCTGCAGGAATTCCACCACCTCTGGAAGAAGATCAAGAGCTGGCAGAAAATCTTCAAACACTATGACACAGACCACTCCGGCACCATCAACAGCTATGAAATGC
GTAATGCCGTGAATGACGCAGGCTTCCACCTCAACAGTCAGCTCTATGACATTATCACCATGCGCTATGCAGACAAGCACATGAACATCAATTTTGACAGCTTCATCTGCTGCTTTGTACGGCTGGAGGGCATGTTCAGGGCTTT
CCAAGCCTTTGACAAGGACGGAGACGGCATCATCAAGCTCAACGTTCTCGAGTGGCTGCAGCTTACAATGTATGCC

----------------------------------------------------------
TTTGTTGTTCTCAGACAACGGTGCCTGGCTCAGAAGTGCCTCTTTGAAGACTGTGTGTTCCCAGCAGGCATCCAATCCCTGGGCTCCCAT---
TGGAAAAGGCCAAAGGAAATTTGTGAGAATCCCCGATTTATCATTGGTGGAGCCAATAGAACTGACATCTGCCAAGGAGATCTAGGGGACTGCTGGTTTCTTGCAGCCATCGCTTGCCTGACCTTGAACGAGCGTCTGCTTTTCC
GGGTCATACCCCATGATCAGAGTTTCACCGAAAACTACGCGGGGATTTTTCACTTCCAGTTCTGGCGCTATGGAGACTGGGTGGACGTGGTTATTGATGACTGCCTGCCAACTTACAACAATCAACTGGTTTTCACCAAATCCAA
CCATCGCAATGAGTTCTGGAGTGCTCTGCTGGAGAAGGCTTATGCTAAGCTCCATGGTTCGTATGAAGCCCTGAAAGGCGGGAACACTACAGAGGCCATGGAGGACTTCACGGGAGGAGTGACAGAGTTTTTTGAAATCAAGGAT
GCTCCCAGAGACATGTACAAGATCATGAAGAAAGCCATCGAGAGGGGTTCCCTCATGGGCTGCTCCATT-----------------------------------------------------------------------------
-----------------------------------------------------------------------GAGACAAGAATGGCCTGTGGGCTGGTCAAAGGCCATGCCTACTCAGTCACTGGGCTG------------
AAGGGTGAGAAAGTGAAGCTGGTGCGGCTGCGGAACCCCTGGGGCCAGGTGGAGTGGAATGGCTCCTGGAGTGACAGCTGGAAGGACTGGAGCTATGTGGACAAGGACGAGAAGGCCCGTTTGCAGCACCAGGTCACTGAGGATG
GAGAGTTCTGGATGTCCTACGATGATTTTATCTACCATTTCACAAAGCTGGAGATCTGCAACCTCACAGCTGATGCCCTGGAGTCCGACAAGCTTCAGACGTGGACAGTGTCCGTGAACGAGGGCCGCTGGGTGAGGGGCTGCTC
TGCCGGAGGCTGCCGCAACTTCCCAGACACTTTCTGGACCAACCCACAGTACCGTCTGAAGCTCCTAGAGGAGGACGACGACCCCGATGATTCCGAGGTGATCTGTAGTTTCCTCGTGGCTCTGATGCAGAAGAACCGGAGGAAG
GACCGGAAGCTGGGGGCTAACCTCTTCACCATCGGTTTCGCCATCTACGAGGTTCCCAAAGAGATGCACGGCAACAAGCAGCACCTGCAGAAGGACTTCTTCCTGTACAACGCCTCCAAGGCTAGGAGCAGAACCTATATCAACA
TGCGCGAGGTGTCTGAGCGCTTCCGCCTGCCTCCCAGCGAGTACGTCATTGTGCCCTCCACTTACGAGCCCCACCAGGAGGGCGAGTTCATCCTCCGGGTCTTCTCAGAAAAGAGGAACCTCTCTGAGGAAGTTGAGAATACAAT
CTCCGTGGATCGGCTA----------------------------------------------------------------------------------------------------------------
GGCAACGACCAGGAAAGTGAGGAACAGCGGCAATTCCGGAATATTTTCAGGCAGATAGCAGGCGATGACATGGAGATCTGTGCAGATGAGCTCAAGAATGTCCTTAACAGAGTTGTGAACAAACATAAG---
CTGAAGACACAAGGCTTCATGATCGGCTCTGGGAGACTGAACCTGCAAGAGTTTCATCACCTCTGGAAGAAGATTAAGACGTGGCAGAAAATTTTCAAACACTACGACACAGACCAATCCGGCACCATAAATAGCTACGAGATGC
GCAATGCAGTCAATGATGCAGGCTTCCACCTCAACAACCAACTCTACGATATCATTACCATGCGCTATGCAGACAAGTACATGAACATCGACTTCGACAGTTTCATCTGCTGCTTTGTCAGGCTGGAGGGCATGTTCAGGGCTTT
CAATGCATTTGACAAGGATGGGGACGGTATCATCAAACTCAACGTTCTCGAGTGGCTGCAGCTCACCATGTATGCC

----------------------------------------------------------
TTTGTTGTTCTCAAACAATGGTGCCTGGCTCAGAAGTGCCTCTTCGAAGACCATGTGTTCCCAGCAGGTGTTCAGGCCCTGGGTTCCCGT---

TGGAAAAGGCCAAAGGAAATTTGCAAGAATCCCCGATTTATTATTGGTGGAGCCAACAGAACCGACATCTGTCAAGGAGATCTAGGGGACTGCTGGTTTCTCGCGGCCATTGCTTGCCTGACCCTGAACGAGCGCCTGCTTTTCC
GAGTCATACCCCACGATCAGAGTTTCACGGAAAACTACGCTGGGATCTTCCACTTCCAGTTCTGGCGCTATGGAGACTGGGTGGACGTGGTTATTGATGACTGCCTGCCAACTTACAACAATCAACTGGTTTTCACCAAATCCAA
CCACCGCAATGAGTTCTGGAGTGCTCTGCTGGAGAAGGCTTATGCTAAGCTCCACGGTTCCTATGAAGCCCTGAAAGGTGGGAACACTACAGAGGCCATGGAGGACTTCACAGGAGGGGTGACAGAGTTCTTTGAGATCAAGGAA
GCTCCCAGTGACATGTACAAGATCATGAAGAAAGCCATCGAGAGAGGCTCCCTCATGGGCTGCTCCATT----------------------------------------------------------------------------
-----------------------------------------------------------------------GAGACAAGAATGCCCTGTGGGCTAGTCAGAGGCCATGCCTACTCGGTCACTGGGCTG------------
AAGAATGAGAAAGTGAAGCTGGTGCGGCTGCGGAACCCCTGGGGCCAGGTGGAGTGGAATGGCTCCTGGAGTGATAGTTGGAAGGACTGGAGCTTTGTGGACAAAGAAGAGAAGGCCCGTCTGCAGCACCAGGTCACTGAGGACG
GAGAGTTCTGGATGTCATATGACGATTTCATCTACCATTTCACAAAGCTAGAGATCTGCAACCTCACAGCCGACGCCCTGGAGTCTGACAAGCTTCAGACCTGGACCGTGTCCGTGAACGAGGGCCGCTGGGTGAGGGGCTGCTC
TGCCGGAGGCTGCCGCAACTTCCCAGACACTTTCTGGACCAACCCGCAGTACCGTCTGAAGCTCCTGGAGGAGGACGATGACCCCGACGACTCGGAGGTGATCTGTAGCTTCCTGGTGGCCCTGATGCAGAAGAACCGGCGGAAG
AACCGGAAACTGGGGGCCAACCTCTTCACCATCGGCTTCGCCATCTACGAGGTTCCCAAAGAGATGCACGGGAACAGGCAGCACCTGCAGAAGGACTTCTTCCTGTACAATGCCTCCAAGGCCAGGAGCAAGACCTACATCAACA
TGCGGGAGGTGTCCCAGCGCTTCCGCCTGCCTCCCAGCGAGTACGTCATCGTCCCCTCCACCTATGAGCCCCACCAGGAGGGAGAATTCATCCTCCGGGTCTTCTCGGAAAAGAGGAACCTCTCTGAGGAAGTTGAAAACACAAT
CTCCGTGGATCGGCCA---------------------------------------------------------------------------------------------------------------
GGCAACGATCAGGAAAGTGAGGAACAGCAGCAATTTCAGAACATTTTCAGGCAGATAGCGGGTGACGACATGGAGATCAGTGCAGATGAGCTCAAGAATATCCTTAACACAGTTGTGAACAGACATAAG---
CTGAAGACACAAGGGTTCATGATTGGCTCTGGGAGGCTGAACCTGCAAGAGTTCCATCACCTCTGGAAGAAGATTAAGGACTGGCAGAAAATTTTCAAACACTACGACACAGACCAATCTGGTACCATCAACAGCTATGAGATGC
GGAATGCAGTCAATGACGCAGGATTCCACCTCAACAGACAGCTGTACGACATCATTACCATGCGGTATGCAGACAAACACATGAACATTGACTTCGACAGTTTCATCTGCTGCTTTGTCAGGCTGGAGGGCATGTTCAGAGCTTT
TAAAGCATTTGACAAGGATGGAGACGGTATCATCAAACTCAATGTTCTAGAGTGGCTGCAGCTCACCATGTATGCC

---------------------------------------------------------
TTCACCGCGCTCAAGCAGGAGTGCCTGCAGAAGAAGAGCCTGTTCGAGGACGACACCTTCCCCGCCACCGTGGAGTCCCTGGGCTACAAG---
TGGAAGAGGCCCAAGGAAATCTGTGAGAACCCTGAGTTCATTGTGGGCGGAGCCAGCAGGACAGACATCTGCCAGGGAGACCTGGGGGACTGCTGGCTGCTGGCTGCCATTGCCTGCCTCACGCTGTATGAGAAGCTGCTGTACC
GCGTGGTTCCCCAGGAGCAGAGCTTCTCCGAAGGATACGCCGGCGTCTTCCACTTCCAGTTCTGGCGCTATGGCGACTGGGTGGATGTTGTCATTGACGACCGCATCCCCACCTTCAACAACCAGCTGGTGTTCACCAAGTCTGC
CGAGAGGAATGAGTTCTGGAGCGCCCTTCTGGAGAAGGCCTACGCCAAGCTCCACGGCTCGTACGAGGCCCTGAAGGGAGGCAACACCACGGAGGCCATGGAGGACTTCACCGGGGGGGTGACCGAGTTCTACGAGATGAAGGAG
GCCCCGAAGGAGCTCAACAAGATCATGAAGAAGGCCCTGGAGAGGGGCTCGCTCATGGGCTGCTCCATCGAT-------------------------------------------------------------------------
-----------------------------------------------------------------------GAGACCCGGACCACCACTGGCCTGGTGAAGGGCCACGCCTACTCTGTGACCGCTGTG------------
AAGGACTCCAAGGTGCGCCTGGTGCGCCTCAGGAACCCCTGGGGCCAGGTGGAGTGGACCGGCCCCTGGAGCGACAACTCCAAGGAGTGGACCTCCCTCTCCAAGGATGAGAAGGAGAAGCTCCAGCACCAGAATGCCGAGGACG
GAGAGTTCTGGATGTCCTTCGAGGACTTCAAGAAGAACTTCACCAAGATCGAGATCTGCAACCTGACCCCTGACGCCCTGGAGGACGACAAGATCCACAAGTGGACCGTGTCGGTGAACGAGGGCCGCTGGGTGAGGGGCTGCTC
CGCCGGGGGCTGCCGGAACTACCCAGACACCTTCTGGACCAACCCGCAGTACCGCCTGCGTCTGCTGGAAGAGGACGACGACCCCGAGGACAACGAGGTGGCCTGCTCCTTCGTGGTGTCCCTGATGCAGAAGAACCGCAGGAAG
GAGCGCAAGATGGGCGCCAACCTCTTCACCATCGGCTTCGCCATCTACGAGGTGCCAAAGGAGATGCACGGCAACAAGCAGCACATGCAGAAGGACTTCTTCCTGGCAAACTCGTCCAAGGCCCGCTGCAAGTCCTACATCAACC
TGCGCGAGGTGACCCAGCGCTTCCGCCTGAGCCCCGGGGAGTACGTCATCGTGCCCTCCACCTACGAGCCCCACCAGGAGGGCGAGTTCCTCCTGCGCGTCTTCTCCGAGAAGAGGAACACCTCAGAGGAGATAGAGAACAGGAT
CGAAGCGGACCATCCG---------------------------------------------------------------------------------------------------------------
GCCTCTGGGGAAGAGAGCGAAGAGGACCAGAAATTCCGGACGATTTTTCAGGAGATAGCCGGAGACGAGATGGAGATCACAGCCAACGAGCTGAAGAACCTGCTCAACAAAGTCGTCTCCAAGAACCAG---
CTGAACACGGAGGGCTTCATGATCGGCACCGGTCGGCTAAACCTCCAGGAGTTCAGACACCTGTGGAACAAGGTCAAACAGTGGCAGGGGATCTTTAAGCACTACAGCGTGGAGCAGTCTGGGAGCATCAGCAGCTACGAGATGA
GGAATGCCGTCAACGATGCCGGCTACCGTCTCAATAACCAGCTTTACGACATCATCACCATGCGTTACGCCAACGAGAGCATGAACATCGACTTCGACAGTTTCATCAGCTGCCTCGTGCGCCTGGAGGCAATGTTCAGAGCTTT
CCAGGCCTTCGATCAGGACGGAGATGGATCCATCCGGCTCAGCGCTCTGGAGTGGCTCCAGCTGACCATGTACGCC

---------------------------------------------------------
TTCAACGCGCTGAGACAGGAGTGTCTCCAGAGGAAGTCACCGTTCGAGGATGACTCTTTCCCGGCCACCGTGGAGTCTCTGGGCTTCAAG---
TGGAAGAGGCCCAAGGAAATCTGTGAGAACCCTCAGTTCATTGTTGGAGGAGCCAGTAGGACAGACATCTGCCAGGGAGATCTGGGTGACTGCTGGCTGCTGGCTGCCATCGCCTGTCTCACCTTGAATGAGAAGCTTCTCTACC
GCGTCGTTCCCCAGGAGCAGAGCTTCTCTGAGGGCTACGGAGGCATCTTCCACTTCCAGTTCTGGCGCTATGGCGACTGGGTCGACGTCGTCATTGACGACCGCATCCCAACCTTCAACAACCAGCTGGTGTTCACCAAGTCCGC
GGAGAGGAACGAGTTCTGGAGCGCCCTGCTGGAGAAAGCCTACGCCAAGCTGCACGGCTCCTACGAGGCCCTGAAGGGGGGAAACACCACCGAGGCCATGGAGGACTTCACCGGTGGAGTCACTGAATTCTACGAGTTGAAGGAA
GCGCCCAAAGAGCTCTACAAGATCATGAAGAAAGCTCTGCTGAGGGGCTCGCTCATGGGCTGCTCCATCGAC-------------------------------------------------------------------------
-----------------------------------------------------------------------GAGACTCGCACCGCGACGGGACTCGTGAAGGGTCACGCCTACTCCGTGACGGCAGTG------------
AAGGACTCGAAGGTCCGCCTGGTGCGCCTCAGGAACCCGTGGGGTCAAGTGGAGTGGAACGGCCCCTGGAGTGACAATTCCAAGGAGTGGGCCAATCTCTCTGCGGCGGAAAAGGAGAAGCTGCAGCACCAGAGTGCCGAGGATG
GAGAGTTCTGGATGTCGTTCGAGGACTTTAAGAAGAACTACACCAAGATCGAGATCTGTAACCTCACCCCCGACGCCCTGGAGGACGACAAGATCCACAAGTGGACGGTTTCTGTGAACGAGGGTCGCTGGGTGAAGGGCTGCTC
CGCGGGGGGCTGCAGGAACTACCCAGACACCTTCTGGACCAACCCGCAGTACCGCCTTCGTTTGCTGGAGGAGGACGACGATCCCGAGGACAACGAGGTGGGCTGCACCTTCGTGGTGGCTCTGATGCAGAAGAACCGGCGGAAA
GAGCGCAAGATGGGCGCCAACCTCTTCACCATCGGATTCGCCATTTACGAGGTGCCAAAGGAGATGCACGGGAACAAGCAGCACATGCAGAAGGACTTCTTCGTCTTCAGCTCCTCCAAAGCTCGCTGCAAGTCCTACATCAACC
TGCGCGAGGTGACGCAGCGCTTCCGCCTGAGCCCCGGCGAGTACGTCATCGTGCCCTCCACCTACGAGCCGCACCAGGAGGGCGAGTTCATCCTGCGGGTCTTCTCTGAGAAGAAGAACACCTCAGAGGAGATAGAGAACAGGAT
CGAGGCCGACCATCCG---------------------------------------------------------------------------------------------------------------
GCCTCAGGGGAAGAGAGCGAAGAGGACCAGCAGTTCAGGACTATTTTTCAGGAGATAGCCGGTGATGACATGGAGATCACAGCCAACGAGCTGAAGAACGTCCTCAACAGAGTGATCACCAAACATAAG---
CTGAACACCAAGGGCTTCATGATAGGGACGGGTAGACTCAACCTCCAGGAGTTCAGACACCTGTGGAATAAGATCAAGCAGTGGCAGGGAATCTTCAAGCACTACAACGCCGATCAGTCCGGTAGCATCAACAGCTACGAGATGA

GGAACGCTGTCAATGATGCAGGCTTCCGTCTCAACAACCAGCTGTATGACATCATCACCATGCGCTACGCCAACGAGGGCATGAACATCGACTTCGACAGCTTCATCAGCTGCCTGGTTCGGCTCGAGGCCATGTTCAGAGCGTT
CCAGGCCTTCGATCAGGACGGAGACGGAACGATCCGACTCAGCGTCCTGGAGTGGCTCCAGTTGACCATGTACGCC

---------------------------------------------------------
TACAGCGTCCTGAGGCAGGAGTGCGTCCACAGGAAGTCCCTGTTCGAGGATGACACCTTCCCAGCCACCGTAGAGTCTTTGGGCTACAAG---
TGGAAACGGCCCAAGGAGATCTGTGAGAATCCTCAGTTCATAGTTGGAGGAGCCAGCAGGACCGACATCTGCCAGGGGGACCTGGGTGACTGCTGGCTGCTGGCCGCCATCGCTTGTCTCACCCTGAACGAGAAGCTGCTGTATC
GGGTCGTTCCGTCCGAGCAGAGCTTCTCAGAGGGCTACGTTGGCGTCTTCCACTTCCAGTTCTGGCGTTACGGAGACTGGGTGGACGTGGTCATCGACGACCGCATCCCGACCTTCAACAACCAGCTGGTCTTCACAAAGTCGGC
GGAGAGGAACGAGTTCTGGAGCGCCCTGCTGGAGAAGGCCTACGCCAAGCTACATGGTTCCTACGAGGCCCTGAAGGGTGGCAACACCACCGAGGCCATGGAGGACTTCACCGGCGGCGTCACCGAGTTCTACGAGATGAAGGAG
GCCCCTAAAGAGCTCTATAAGATCATGAAGAAAGCTCTGGACAGAGGCTCGCTCATGGGCTGCTCCATCGAC--------------------------------------------------------------------------
------------------------------------------------------------------------GAGACTCGGACGGCGACGGGACTGGTGAAGGGCCACGCCTACTCGGTGACAGCGGTG------------
AAGGACGCGAAGGTCCGCCTGGTGCGCCTCAGGAATCCCTGGGGTCAGGTGGAATGGAACGGTCCCTGGAGCGACAACTCCAAAGAGTGGTCCACCATCTCCAAGGCTGAAAAAGAGAAGCTGCACCACCAGAGCGCCGAGGATG
GAGAGTTCTGGATGTCCTTCGAGGACTTCAAGAAGAACTACACCAAGATCGAGATCTGCAACCTGACGCCCGACGCTCTGGAGGACGACAAGATCCACAAGTGGACCGTTTCTGTGAACGAAGGCCGCTGGGTGAGGGGCTGCTC
CGCTGGGGGCTGCAGGAACTACCCAGACACTTTCTGGACAAACCCTCAGTACCGCCTCCGTCTGCTGGAGGAGGACGACGACCCCGAGGAAAACGAGGTGGGCTGCACCTTCGTGGTGGCTCTCATGCAGAAGAACAGGCGGAAA
GAGCGCAAGATGGGCGCCAACCTCTTCACCATCGGATTCGCCATCTACGAGGTTCCAAAGGAGATGCACGGCAACAAGCAGCACATGCAGAAGGACTTCTTCCTGTTCAGCTCCTCCAAAGCTCGCTGCAAGTCCTACATCAACC
TGCGGGAGGTGACGCAGCGCTTCCGCCTGAGCCCCGGGGAGTACGTCATCGTGCCCTCCACGTACGAGCCGCACCAGGAGGGCGAGTTCATCCTGAGGGTCTTCTCCGAAAAGAAGAACACGTCAGAGGAGATAGAGAACAGGAT
CGAGGCCGACCACCCG----------------------------------------------------------------------------------------------------------------
GCGTCCGGCGAGGAGAGCGAGGAGGACCAGCAGTTCAGGACTATTTTTCAGGAGATAGCCGGAGATGACATGGAAATCACCGCCAACGAACTGAAGAACGTCCTCAACAGAGTGATCATCAAACATAAG---
ATGAACACGGAGGGCTTCATGATTGGGACAGGTCGGCTCAACCTGCAGGAGTTCAGACACCTGTGGAACAAGCTGAAGCAGTGGCAGGGAACCTTCAAGCACTACGACGCCGACCAGTCCGGCTTCATCAACAGTTACGAGATGA
GGAACGCCGTCAACGATGCAGGCTTCCGTCTCAACAACCAGCTGTATGACATCATCACCATGCGCTACGCCAACGAGAACATGAACATCGACTTCGACAGCTTCATCAGCTGTCTGGTTCGCCTCGAAGCCATGTTCAGGGCGTT
TCAGGCCTTCGACCAGGACGGAGATGGGACCATCAGACTCAGCGTCCTGGAGTGGCTCCAGCTGACCATGTACGCC

---------------------------------------------------------
TTCAGCACCTTGCGGCAGGAGTGCCTGCAGAGGAAGGGCCTGTTCGAGGACGACTCCTTCCCTGCCACCGTCGAGTCTCTGGGCTTCAAG---
TGGAAGAGGCCCAAGGAAATCTGTGACAACCCCCAGTTCATAGTTGGGGGGGCCAGCCGGACTGACATCTGCCAGGGAGATCTGGGTGACTGCTGGCTGTTGGCCGCCATCGCTTGCCTCACGCTGAACGAGAAGCTGCTTTTCC
GAGTGGTTCCTCAGGAGCAGAGCTTTTCCGAGAGCTACGCCGGCATCTTCCACTTCCAGTTCTGGAGGTATGGCGACTGGGTGGACGTCGTCGTTGACGACCGCATCCCAACCTTAAACAACCAGCTGGTTTTCACCAAGTCAGC
GGAGAGAAATGAATTCTGGAGTGCCCTGCTGGAGAAGGCCTATGCCAAGCTTCACGGCTCCTATGAGGCCCTGAAGGGGGGAAACACCACAGAGGCCATGGAGGATTTCACCGGGGGGGTCACTGAGTTTTATGAGATGAAGGAA
GCTCCCAAAGAGCTATACAAGACCATGAAGAAAGCTCTGGAGAGAGGCTCCCTGATGGGGTGCTCCATCGAT--------------------------------------------------------------------------
------------------------------------------------------------------------GAGACGCGCACGACGACGGGGCTGGTGAAGGGACACGCCTACTCTGTGACGGCTGTG------------
AAGGAGTCCAAGGTTCGCCTGGTGCGCCTCAGGAACCCGTGGGGTCAGGTGGAGTGGAACGGTCCCTGGAGTGACAACTCCAAGGAGTGGGCCACGCTCTCTAAGGCAGAGAAGGAGAAACTGCAGCACCAGAGCGCAGAGGACG
GGGAGTTCTGGATGTCCTTTGAGGACTTCAAGAAGAACTACACCAAGATTGAGATCTGTAACCTGACTCCAGACACGCTGGAGGACGATAAGATCCATAAGTGGACCGTTTCTGTGAACGAGGGCCGCTGGTTGCGGGGCTGCTC
CGCCGGTGGCTGCAGGAACTACCCAGACACTTTCTGGACCAACCCTCAGTACCGCCTGCGCCTGCTGGAGGAGGACGACGACCCGGATGACAACGAGGTGGCCTGCACCTTCGTGGTCTCACTGATGCAGAAGAACAGACGGAGG
GAGCGCAAGATGGGAGCCAACCTCTTCACCATTGGGTTCTCCATTTATGAGGTCCCCAAAGAGATGCATGGGAACAAGCAACACTTGCAGAAGGACTTCTTCCTCCTCAACTCCTCCAAGGCACGCTCCAAATCCTACATCAACC
TCCGGGAGGTGACGCAGCGCTTCCGGCTGAGCCCTGGCGAGTACGTCATCGTCCCCTCCACCTATGAGCCGCACCAGGAGGGGGAGTTCATCCTGCGCGTCTTCTCTGAGAAGAGGAACACCTCAGAGGAAATAGAGAACAGGAT
CGAAGCCGACCATCCA----------------------------------------------------------------------------------------------------------------
GCCTCAGGGGAGGAGAGCGAGGAGGACCACCATTTCCGGACGATTTTTCAGGAAATAGCTGGTGAGGACATGGAGATCACAGCCAACAAACTGAAAAACGTTCTGAACAGAGTGATCACTGAGCGTAAG---
CTGAACACGGTGGGCTTCATGATTGGGACGGGCCGACTCAACCTGCAGGAGTTCAGACATCTGTGGAATAAGATCAAGCAGTGGGAGGGAATCTTCAAACATTATAACGCCGACCAGTCCGGCATCATCAACAGCTATGAGATGA
GGAACGCTGTGAATGATGCAGGCTTTCGCCTCAACAACCAGCTGTACCACATCATCACGCTGCGCTACGCCAACGAGAACATGAACATCGACTTCGACAGCTTCATCAGCTGCCTGGTTCGCCTGGAGGCCATGTTCCGAGCATT
CCAAGCCTTTGATCAGGATGGAGATGGAACCATCAGACTCAGCGTGCTGGAGTGGCTCCAGCTGACCATGTACGCC

---------------------------------------------------------
TACAACACCCTGAGACAGGACTACCTGCAGAGGAAGACTCTGTTTGAGGATGAAGCTTTCCCCGCCACAGTGGAGTCTCTGGGCTTCAAG---
TGGAAGAGGCCTAAGGAAATCAGTGAAAACCCTGAGTTCATTGTCGGAGGAGCGAGTAGGACAGACATCTGTCAGGGAGATCTTGGTGACTGCTGGTTGTTAGCCGCTATTGCTTGTCTCACCCTGAATGAGAAACTCCTCTACC
GAGTTGTTCCCCAGGAGCAAAGCTTCTCTGAGAATTATGCCGGCATCTTTCACTTCCAGTTCTGGCGTTACGGTGACTGGGTCGACGTCGTCATTGACGACCGCGTCCCGACTTTCAACAACCAGCTGGTCTTCACTAAGTCCGC
CGAGAGAAACGAGTTCTGGAGTGCCCTGCTGGAGAAGGCCTACGCAAAGCTTCACGGCTCTTACGAGGCCCTGAAAGGCGGGAACACCACGGAAGCCATGGAGGATTTCACGGGAGGCGTCACCGAGTTTTACGAGATGAAGGAT
GCTCCAAAAGAGCTTTATAAGATCATGAAGAAAGCCCTGGAGAGAGGCTCACTCATGGGCTGCTCCATTGAT--------------------------------------------------------------------------
------------------------------------------------------------------------GAGACCCGCACAGTGACGGGACTAGTGAAGGGTCACGCCTACTCCGTGACAGCGGTG------------
AAGGACAACAAAGTTCGTCTGGTGCGTCTCAGGAACCCATGGGGTCAGGTGGAATGGAACGGCCCCTGGAGTGACAACTCCAAAGAGTGGGCGAATCTCTCTAAGGCTGACAAAGACAAACTGCAGCACCAAAGCGCAGAGGATG
GAGAGTTCTGGATGTCATTTGAGGACTTCAAGAAGAATTACACTAAGATTGAGATCTGTAACATGACACCCGACGCCCTCGAGGACGACAAGATCCACAAGTGGACGGTTTCTGTGAACGAGGGCCGCTGGGTGAGAGGCTGCTC
CGCCGGAGGCTGCAGAAACTATCCAGACACCTTCTGGACCAACCCTCAGTACCGCCTCCGCCTGCTGGAGGAGGACGACGACCCGGACGACAACGAGGTGGTCTGCACCTTCGTGGTGTCCCTGATGCAGAAGAACAGACGAAAA

GAGCGAAAGATGGGGGCCAACCTCTTCACCATCGGATTCGCCATTTACGAGGTGCCAAAGGAGATGCATGGAAACAAGCAGCACATGCAGAAGGACTTCTTCTTGGTCAACTCCTCCAAGGCCCGCTCCAGGTCCTACATCAACC
TGCGGGAGGTGACGCAGCGCTTCCGCCTGAGCCCCGGCGAGTACGTCATCGTGCCTTCCACGTACGAGCCGCACCAGGAGGGGGAGTTCATCCTGAGAGTCTTCTCTGAAAAGAGGAACACCTCAGAGGAGATAGAGAACAGGAT
CGAAGCGGACCGTCCG---------------------------------------------------------------------------------------------------------------
GCCTCGGGGGAAGAGAGCGAGGAGGACCAGCAGTTCCGGACTATTTTTCAGGAGATAGCCGGCGACGACATGGAGATCACAGCTAATGAACTGAGAAACGTTCTCAACAGGGTGATCACCACGCATAAG---
CTGAACACGGAGGGCTTCATGATTGGGACAGGTAGACTCAACCTGCAGGAGTTTATACATCTGTGGAATAAGATCAAGCAGTGGCAGGGAATCTTCAAACATTATAACGCCGATCAAACCGGCAGCATCAACAGCTACGAGATGA
GGAACGCCGTCAACGATGCAGGCTTCCGTCTCAACAACCAGCTGTATGACATCATCACTATGCGCTACGCGAACGAGAACATGAACATCGACTTCGACAGCTTCATCAGCTGCCTGGTTCGGCTCGAAGCCATGTTCAGGGCCTT
CCAAGCATTTGACCAGGATGGAGATGGAACCATCAGACTCAGCGTCCTGGAGTGGCTCCAGTTGACCATGTACGCC

 ---------------------------------------------------------
TACACCCTCCTCAAGCAGGAGTATCTCCAGAAGAAGACCCTGTTCGAGGACGAGACCTTCCCTGCTACTGTGGACTCTCTGGGCTATAAA---
TGGAAACGTCCCAAGGAGATCTGCGATAACCCACAGTTCATTGTCGGAGGCGCTAGCAGGACTGATATCTGCCAGGGAGATCTGGGTGACTGCTGGTTGCTTGCAGCCATTGCCTGTCTGACTCTGAATGACAAGCTGCTGTATC
GTGTTATTCCTCAAGAGCAGAGCTTCTCCGAACAATATGCAGGAATTTTCCATTTCCAGTTCTGGCGATATGGGGATTGGGTGGATGTTGTGGTTGATGACCGCATTCCCACCTTCAACAACCAACTGGTGTTCACCAAGTCTGC
TGAGAGGAACGAGTTCTGGAGCGCTCTTCTGGAGAAGGCTTACGCTAAGCTTCATGGGTCCTATGAGGCCCTGAAGGGTGGAAACACAGCCGAGGGAATGGAGGACTTCACTGGTGGAGTGACTGAATTCTACGAGATGAAGGAG
GCGCCCAAAGAGCTGTATAAGATCATGCAGAAAGCTCTAGAGAGAGGCTCACTCATGGGCTGCTCCATTGAT-------------------------------------------------------------------------
-----------------------------------------------------------------------GAAACTCGTACTGCGACAGGTCTTGTGAAGGGTCATGCCTACTCTGTGACCGCTGTT------------
AAGGAGTCTAGAGTGCGTCTGGTGCGTTTGCGTAACCCCTGGGGTCAAGTAGAGTGGAATGGACCATGGAGTGACAATTCTAAAGAATGGGAAAGTCTTTCTAAAGCGGAAAAAGAGAAACTCCAGCAACAGAACGCTGAAGATG
GAGAGTTTTGGATGTCGTTTGAAGATTTTAAGAAAAATTACACCAAAATTGAGATTTGTAACTTGACACCTGATGCCCTGGAGGACGATAAGCTACATAAGTGGACAGTGTCTGTCAATGAGGGCCGCTGGGTGAGAGGCTGCTC
TGCCGGAGGATGCAGAAACTATCCAGACACATTCTGGACAAACCCACAATACCGTCTGCGTCTCCTGGAGGAAGATGACGATCCGGAGGATGATGAAGTGGCCTGCACATTTGTTGTTGCTCTAATGCAGAAAAACAGACGTAAA
GAACGCAAGTTGGGTGCGAATCTCTTGACCATTGGATTTTCCATCTATGAGGTGCCAAAGGAGATGCATGGAAATAAGCAGCACATGCAGAAGGAGTTCTTCATGTCTACCACCGCTAAGGCTCGGTCCAGGGCCTACATTAACC
TGCGCGAGGTCACCCAGCGTTTCCGTCTGAGTCCCGGAGAGTACGTCATCATTCCCTCTTCTTACGAGCCCCATCAGGAAGGCGAGTTCATCCTCCGCGTCTTCTCTGAAAAGAGAAATACTTCTGAGGAGATAGAGAACAGGAT
TGAAGCTGATCATCCT---------------------------------------------------------------------------------------------------------------
GCTTCAGGGGAGGAGAGCGAAGAGGACCAACAGTTCCTGTCCATTTTCCAGCACATCGCGGGGGACAACATGGAGGTCTCTGCCAACGAGCTTAAAGATGTCTTGAACAAGGTGGTGTCTAAACACAAG---
ATTCACACCGAGAGCTTCATGATTGGAACAGGAAGACTTAACCTGCAAGAGTTTAGACAATTGTGGAACAAAATCAAACAGTGGCAGGAAATCTTTAAGCGCTATGACTTTGATCATAATGACACTATCAGCAGCTATGAGATGA
GAAACGCCATCAATGACGCAGGGTTCCGTCTGAACAACCAGCTGTATGACATCATCACCATGCGCTACGCCAACGAAAGCATGAATGTCGACTTTGAGAGTTTCATCAGCTGCCTTGTGCGACTGGAGGGGATGTTCAGAGCATT
CCAGGCCTTTGACCAATGTGGAGATGGCAGCATCAGGCTCAGTGTACTGGAGTGGCTCCAGCTAACCATGTACGCG

 ---------------------------------------------------------
TTTGTGACGCTGAAGCAGGAATGCCTGCAGAAAAAGATTCTGTTTGAAGATGATACGTTTCCAGCCACTGTAGAGTCCCTCGGGTATAAA---
TGGAAAAGACCAAAGGAAATCTGTGACAATCCGAAATTCATCATTGGCGGAGCTCAAGAACTGATATCTGTCAAGGAGATTTGGGTGACTGCTGGTTCCTGGCTGCCATTGCTTGTCTAACTCTTAATGAGAAGCTGCTCTACC
GGGTCATCCCTAGAGATCAATACTTTACAGAAAACTATGCTGGGATCTTCCATTTCCAGTTCTGGCGTTTCGGTGACTGGGTGGATGTCACTGTTGACGACCGAATCCCCCACCTTCAACAACCAGTTGGTTTTCACTAAATCTGC
TGAGAGGAATGAGTTCTGGAGTGCCTTGCTGGAGAAAGCCTATGCCAAGCTGAATGGATCCTATGAAGCGCTGAAGGGTGGCAACACCACGGAAGCCATGGAAGATTTCACCGGTGGTGTTACTGAATTCTTTGAGATGAAGGAG
GCCCCGAAGGATCTGTACAAGATCCTAAAGAAGGCAACAGACAGAGGTTCTCTAATGGGCTGTTCTATTGAT-------------------------------------------------------------------------
-----------------------------------------------------------------------GAGACACGGACTGCCAGTGGCCTGGTCAAGGGTCACGCGTACTCTGTGACCGGTGTG------------
AAAAGTCAGAAGGTGAAATTACTGCGTTTGAGGAACCCATGGGGAGCAGTGGAGTGGAATGGGTCCTGGAGTGACAAGGCCCGAGAATGGTCTGAGATTGAGAAAATACAAAAGCAGCGGCTACAACATCAAACGAAGGAAGATG
GAGAGTTCTGGATGTCTTTTGATGATTTCAAGAGAAATTTCACAAAGCTGGAGATTTGTAACCTGACACCAGATGCACTGGGTGATGATAAGCTCCACAAGTGGACTGTGTCTATCAATGAAGGACGCTGGGTAAAAGGCTGTTC
AGCTGGGGGCTGCCGCAACTATCCAGACACGTTCTGGACAAACCCTCAGTACCGATTGAAGCTCTGTGAGGAGGATGATGATCCTGATGTTTCTGAGGTGCTCTGCACATTTGTCGTCGCTCTGATGCAAAAGAATCGTCGGAAG
GAGCGCAGGGTGGGCATCACATTCCATACCATTGGATTCGCTATTTATGAGGTACCCAAAGAGATGCAAAACAATAAGCAACACCTACCCAAAGATTTCTTCCTCTACCAGGCATCGAAGTGCAGGTCCAAGTCATACATTAACC
TACGGGAGGTATCGCAGCGTTTCAGCCTCTCCCCTGGGGAATATGTCATTGTCCCTTCCACATACGATCCACACCAAGAAGGTGAATTCGTTCTCAGGGTCTTCTCAGAAAAACAGAACCTGTCAGAGGAAACTGAGAACCAGAT
CATAGCTGTCAACCCT---------------------------------------------------------------------------------------------------------------
ACAACAAGGGAGGAGACTGAGGAGGACCTGCAGTTCCGTCGTATTTTTGAACAGATTTCAGGAGATGATATGCAGATCAGTGCATCTGAAATGAGGAATATCTTAAACCGTGTTGTTAAAAAACAC------
ATGAAAACGGAAGGGTTCATGGTTGGGTCCGGAAAGCTTAATTTAATGGAGTTTAGGCATCTCTGGAACAAGATTAAGAAATGGCAGACAATCTTTATGGAATATGACAAAGATGCTTCTGGTTTGATTGACAGCTATGAAATGC
GCACTGCTGTGAATGATGCAGGTTTCCAACTGAACAGTCAGCTCTATGAGATCATCTCCATGCGCTATGCTGATGAAAATATGAACCTGGACTTTGACAGTTTCATCTGTTGTATTGTGAGACTAGAGGGGATGTTCAGAGCTTT
CCATGCCTTTGATAAAGATGGAGATGGAATCATTAAACTCAGTGTTCTGGAGTGGTTGCAACTCACCTTGTATGCT

 ---------------------------------------------------------
TTCTCCCTCCTGAAACAGGAGAGCTTGAGGAAGAAGGTGCTGTTCGAGGATGACTGCTTCCCAGCCACTGTGGAGTCCCTGGGCTACAAA---
TGGAAGAGGCCCAAGGCTGTGGCTAACTGGCCAAAGGTCCAGCTGCAGTCAGCAAAGGACACTGCACCCGTGAAGGCTCAGGTGGGGGACTGTTGGCTGTTGGCTGCCATAGCCTGCCTGACTCTGAATGAGAAGCTACTGTACC
GGGTCATCCCTCCTGATCAGAGCTTCACCGAGAACTACGCCGGCATCTTTCACTTCCAGTTCTGGCGCTACGGTGACTGGGTAGATGTTGTTGTTGATGACCGAATCCCCACTTTCAACAACCAGCTGGTGTTCACCAAGTCAGC
CGAGCGCAATGAATTCTGGAGTGCTCTACTGGAGAAGGCTTATGCCAAGCTTCATGGATCCTATGAGGCCCTCAAGGGAGGGAACACCACGGAGGCAATGGAGGACTTCACAGGGGGGGTGACGGAGTTCTTCGAGATGAAGGAG

GCCCCCAAGGAGCTCTTCAAGATCATGAGCAAGGCCCTGGAGAGGGGCTCTTTGATGGGCTGCTCCATTGAT-------------------------------------------------------------------------
-----------------------------------------------------------------------GAGACTCGTACTGTCACTGGATTAGTAAAGGGTCATGCTTATTCTGTGACAGCAGTA-----------------
-------------------------------------------------------------------------------
GAATGGGTCACAATTCCAAAGTCAGAGAAGGACAAACTACAACATCAGAGTGTGGAGGATGGAGAGTTCTGGATGTCATTTGAGGACTTCAAAAAGAACTTCACCAAGATTGAGATCTGCAACCTGACTCCTGATGCCCTGGAGG
ACGATAAACTCCACAAGTGGACTGTGTCGGTGAACGAGGGGCGCTGGGTGAGGGGCTGCTCTGCTGGAGGCTGTCGCAACTACGCAGACACATTCTGGACAAACCCACAGTACCGTCTGCGGCTTATGGAGGAGGATGATGACCC
CGACGACAATGAGGTCGCCTGCACCTTCGTGGTGGCACTTATGCAGAAGAACCGCAGGAAAGAGCGCAAGATGGGTGCCAACCTGTTCACCATTGGCTTCGCCATCTAT------------
GAGATGCACGGCAACAAGCAGCACATGTCCAAGGACTTCTTCCTGTACAACGCCTCAAAAGCCCGCTGCAAGTCCTACATCAACCTGCGCGAGGTGTCCCAGCGCTTCCGCCTCTGCCCAGGCGAGTACGTGATTGTCCCCTCGA
CCTACGAGCCCCACCAGGAGGGAGAGTTCATCCTCCGCGTCTTCTCTGAGAAGAGGAGCACCTCAGAGGAGATCGAAAACAGGATTGAAGCCGACCATCCA--------------------------------------------
-------------------------------------------------------------------
GCTTCAGGAGAGGAGACTGAGGAGGACAGGCAGTTCAGGACCATCTTCCAGCAGATTGCCGGAGACGATATGGAAATAACAGCTAATGAGCTCAAGAACGTGCTCAACAGGGTGGTTGCAAAACATAAG----------------
-----------ACTGTCAAAGAGGTCAATGCTAATGAAGTG------------
AATGTTGTTCTTTGCTTCCAGGGAATTTTTAAACACTATGACGCTGACCATTCTGGAACTATCAACAGTTATGAGATGCGCAACGCTGTAAATGATGCCGGCTTCCGTCTCAACAACCAGCTGTATGACATCATCACGATGCGCT
ACGCCAACGAAAACATGAATATGGACTTCGACAGCTTCATCTGCTGCTTGGTCAGGCTGGAGGGGATGTTCCGAGCGTTTCATGCCTTTGATAAAGATGGCGATGGAATAATCAAACTCAGTGTGTTGGAG--------------
----------

---------------------------------------------------------
TTTGCCACCCTGAAGCGGGAGTGGCTCGGGAAGAAGGTGCTCTATGAGGATGAAGTGTTCCCAGCAACCGTGGAGTCCCTGGGTTACAAA---
TGGAAAAGGCCAAAGGAGATCTGCGACAATCCACAGTTTATCATCGGAGGGGCCAACAGAACTGATATCTGTCAAGGAGACTTAGGTGACTGTTGGCTCCTTGCTGCCATCGCCTGCCTGACGCTTAACAACAAGCTGCTGTGCC
GCGTTGTGCCCCCAGATCAGAGCTTCACAGAGAACTACATCGGCATCTTCCATTTCCAGTTTTGGCGCTATGGCGACTGGGTCGATGTGGTTATTGATGACCGTATTCCCACCTACAATAACCAGCTTGTCTTCACCAAGTCTGC
TCAGCGCAATGAGTTTTGGAGTGCTCTGTTCGAAAAGGCCTATGCAAAAGTACATGGACAGGAGTCTGCCTGCCTGCCTGAGTTGAGTATAGAGTGTATGCACACTTACAGCCCAGCTATAGAGTTGAGTATAGAGATGCATACT
TACAGCCCAGCTGTACAGAGTGTACAAATGAAAGCTGTCCTGGCTGGCGAAAGTATGGGG-------------------------------------------------------------------------------------
-----------------------------------------------------------------------GAGACCCGCACCTCCTCTGGGCTGGTTAAGGGCCATGCTTACTCGATTACAGGGGTC------------
AAAGGACAGAAAGTGAAGTTAGTGCGGCTGAGAAACCCCTGGGGACAGGTGGAGTGGAACGGCCCCTGGAGTGACAATTCTAAGGAATGGACCACCCTAGAAAAATCTGAGAAAATGCGGCTGCAGCATCATATCGCCGAGGACG
GGGAATTCTGGATGTCCTTTGATGACTTCAAGAAGAACTTCACGAAGCTTGAGATCTGCAACCTGACCCCGGATGCCCTGGTGGAGGACAGAGTCCACAAGTGGACGGTTTCTGTGAATGAGGGCAGATGGGTGCGGGGCTGCTC
TGCCGGAGGCTGCCGCAACTACCCAGACACTTTCTGGACGAACCCTCAGTACCGTCTGAAACTCCTAGAAGAAGATGATGACCCTGATGACAACGAGGTCATTTGCACGTTCCTGGTGGCACTGATGCAGAAGAACCGTCGCAAA
GAGCGGAAGCTGGGAGCAAACCTCTTTACTATAGGATTTGCAATTTATGAGGTGCCGAAGGAGATGTACGGCAACAAGCAGCACTTGCCCAAAGACTTCTTCCTTTATAACGCATCCAAAGCCAGGTGTAAGTCTTACATTAACC
TGCGAGAGGTGTCGCACCGTTTCCGCCTGTCGCCAGGAGAGTACGTGATCATCCCGTCAACCTACGAGCCACACCAAGAGGGCGAGTTCATCCTCCGGGTCTTCTCTGAAAAACGAAACTTATCTGAAGAGGTTGAAAATCGGAT
TGAAGCAGATCACCCT-------------------------------------------------------------------------------------------------------------
GCTTCAGCTGAAGAGAACGAGGAGGACAAGCAGTTCAGGAGCATCTTCCAGCAGATTGCAGGAGACGATATGGAAATCAGCTCCTGTGAACTGAGAAGTGTTTTAAACAGAGTTCTTGCCAAACACAAG---
CTGAAGACAGATGGTTTCATGATCGGATCAGGAAGGCTGAACCTTCAGGAGTTCCGGCTCCTGTGGAATAAGATCAAGCAATGGCAGGGCATCTTTAAGCAATACGACATGGACAAGACTGGATACATCAACAGTTATGAGATGC
GCAATGCAGTTAACGATGCAGGTTTCCGTTTGAACAACCAGCTGTATGACATCATCACCATGAGATACGCTGATAAACAGATGAATATTGACTTTGACAGTTTCATCTTGTGCTTTGTCAGGCTAGAGGGAATGTTCAGAACATT
CCATGCCTTCGACAAAGACGGAGATGGACTCATTAAACTAAATGTCTTGGAGTGGCTGCAGCTCACCATGTATGCT

TCCAACTCAATTTACGCTGCCATCCTCAGCAGGAACGAGACCGTCAAACTGAAGACTTTTCTGGAACTGCGGGACAAGTATGTGCACAAGAAAGTGCTGTTTGAAGACCCCCTGTTCCCTGCAGACGACTCTTCTCTG------
------
TGGAAGCGTCCAACGGAAATCTGTGGAAATCCCCACTTCATCATAGATGGAGCCAACAGATCAGATATCTGTCAAGGAGAATTAGGGGACTGCTGGCTCCTGGCTGCCATCGCGTGTCTGACTCTGAACGAGAAGCTGCTGTACA
GGGTCATCCCTCCGGACCAGAGCTTCACTGAGAACTATGCCGGCATCTTCCACTTCCAGTTCTGGCGCTACGGCGAATGGATTGACGTCATCATTGACGACCGCATCCCCACCTTCAACGACAAGCTGGTGTTCACCAAATCCTT
CCGGAAGAATGAATACTGGAGCGCTCTCCTGGAGAAAGCGTACGCCAAATTGCACGGCTCCTACGAGGCTCTGAAAGGTGGCAACACGCTGGAGGCCATGGAGGACTTCACGGGGGGCGTGACCGAGTACTTTGACCTGTTGGAG
GCCCCCAAGGACCTGTACTCTATCATGAAGAAGGCCCTGGAGAGGGGCTCACTCATGGGCTGCTCCATAGAC-------------------------------------------------------------------------
-----------------------------------------------------------------------GAAAGCCGCACTTCTCAAGGACTAGTGCAAGGGCATGCCTACTCTGTTATAGGTCTG------------
ACCGACTCCCAGATCCGTCTCGTTCGGTTGCGTAACCCCTGGGGCTGGGTGCTCTGGAAGGGACGGTGGTGTGCAGATTCCAAAGAGTGGTCCACCATATCCACTGCTGACAAAGAAAATCTACTGAAGCAGACCATACAGGAAA
GTGAGTTCTGGATATCCTTTGAGGACTTCAAGAAGTGCTACTCCAAGCTGGAGATGTGTAACCTCACCCCCGACACCCTGCGTGGCGACGAGAGGAACAGCTGGAGCGTGGCGGTGAACGAGGGCCGCTGGGTGAGAGGCAGCTC
CGCGGGGGGCTGCAGGAACTTCCCAGACACATACTGGACCAACCCCCAGTACCGCATGCTGCTGTACGAGGAGGACGACGACCCCGAGGACGGGAAGAGGGCGTGCACGGTGGTGGTGGCTCTGATGCAGAAGGGCCGCCGAAAG
GAGCGCAGCAAGGGGGCAAGCCTCTACACCATCGGCTTCTCCATCTACGAGGTGCCAAAGGAGATGCATGGCTGCCAACAACATCTGGGAAAAGAATTCTTCCTCTACACCAAGTCCACGGCCAAGTGCCAGTCCTACATCAACC
TGCGGGAGATCACCGAGCGCTTCCGCCTGCCCCCCGGGGAGTACGTGGTCATCCCCACGACCTTTAAACCCCACAATGAGGGGGAGTTCATCCTCAGAGTGTTCTCTGAGAAGAGGAGCACCTCTGAGGAGGTCGAAGAGGAGAT
TGGATCTGATGAA---------------------------------------------------------------------------------------------------------------------------
GAGGAGACTGAGGAGGACAAAACCTTCAGGGCCATTTACAGACAGATCGCTGGTGAGGACATGCAAATCTGTGCCAGTGAGCTCAAAGTTGTCATGAAGAGGGTGCTGGAAAAACACAAC---
ATAAAGACGGAGGGCTTCATGATCGGCACTGGAAAGCTGAATCTACAAGAATTCAAACACTTGTGGAATAAAATCAAACAGTGGCAGCTCATCTTCAGAATGTACGACAAAGACAATCCTGCACCATCAGTAGCTTCGAAATGA

GGAACGCTGTGAATGATCCTGGTTTCCACCTCAATAAGCAGTTGTACGACATATTAGCCATGCGCTATGCAGACGAACGCAACAACATTGACTTTGACAGCTTCATCTGCTGTTTTGTGAGGCTAGAAGGAATGTTCAGGGCCTT
CCATGCGTTTGACAAAGACGGAGATGGTTTGATCAAGCTCAACGTCCTAGAGTGGCTTCAGCTGACCATGTATTCA

---------ATCTACTCAGCAATTCTCAGCAGAAACGAGGCCGTCAAATTGAAGACGTTTTTGGAACTGCGAGATAAATATGTGAAGAAGAAGGTTTTGTTTGAGGATCCTCTGTTCCCTGCAAATGACTCGAGTCTT------
------

TGGAAACGTCCCACAGAAATCTGTGAAAACCCTCAGTTCATCATAGATGGAGCCAACCGAACTGACATCTGCCAAGGAGAACTGGGTGACTGTTGGCTGCTTGCTGCCATCGCCTGTCTCACCCTGAGTGAAAAGCTGCTGTACA
GAGTCATTCCACCAGAGCAAAGTTTCACAGAAAACTATGCTGGCATCTTTCACTTCCAGTTCTGGCGTTATGGTGAATGGATTGATATAGTTGTGGACGACCGGATCCCCACCTGCAACAACCTGCTAGTTTTCACCAAGTCATT
CAGAGAGAATGAGTTCTGGAGTGCCCTTTTGGAGAAAGCTTATGCCAAGCTGCATGGGTCTTATGAAGCACTGAAAGGGGGGAACACGTTGGACGCCATGGAGGATTTCACAGGAGGAGTCACCGAGCTCTTTGAGTTGTCCGAG
GCGCCCAAAGGCCTGTTCATCATCATGAAGAAAGCACTGGAGAGGGGCTCACTGATGGGCTGCTCCATTGAT-----------------------------------------------------------------------------
---------------------------------------------------------------------------GAGAGCCGGACAAATCAAGGGCTTGTCAGAGGTCACGCCTACTCCATCATAGCCCTG------------
GAGGACGGCAAAATTCGCCTGATTCGTTTGCGCAATCCCTGGGGTTGGGTCCTCTGGAAGGGACCCTGGAGTGTGAATTCAAAGGAGTGGTCGACCGTCTCCACTGCAGACAGGGATAATCTGAAAAAACAGACGTTGGAGATGA
GTGAGTTCTGGATGTCTTTTGACGATTTCAAGAAAAATTTCACTAAACTTGAGATGTGCAACTTAACCCCTGATGCTCTCACGGGGGACGAGAGACAAAGCTGGACGGTGTCAATCAACGAGGGTCGCTGGGTGAGGGGCAGCTC
TGCTGGTGGCTGCAGAAATTTTCCAGACACATTTTGGACCAACCCTCAATACCGGCTGCGGCTGTACGAAGAG---
GACGACCCCGAGGAAGAGCAGGTACTCTGCACAGTTGTGGTGGCTCTGATGCAGAAAGGTCGGAGGATGCAGCGCCATGCTGGAGCCAAATTCCTCACCATTGGATTTTCCATTTATGAGGTGCCAAAAGAGTTGCAGGGGCAGA
ATCAGCATCTGCAGAAGGACTTTTTCCTCTACAATGCCTCCAAAGCAAAGTGCAAGGCCTACATTAACCTGCGGGAGGTAACAGAGCGTTTCCGTCTCCCTCCGGGGGAGTACGTCATCCTGCCTACAACCTTTAAAGCTCATGA
AGAAGGAGAGTTCCTCCTGCGGGTTTTCTCTGAGAACAAAAGCACATCAGAGGAGGCAGAGACCAGCATTGACAAAATTGAGCGT---------------------------------------------------------------
------------------------------------------------------

CTACAAGTGGAAGAGACTGAGGAGGAAAAGCAGTTCAGAGTCATTTACCAAAAGATTGCTGGTGAGGACATGGAGATCTGCGCCAGCGAGCTGATGGCGATCATGAAGAACGTACTCTCCAAGCATAGC---
ATAAAGTCAGACGGCTTTATGATTGGGTCTGGAAAGTTGAACCTGCAGGAGTTCAAACATTTGTGGAAAAAGATCAAGGAGTGGCAGCTCATCTTTAAGCGCTACGATAAAGATGGAAAGTGCTCCATCAGCAGTTTTGAGATGA
GGAATGCTGTAAATGACGCAGGGTTTAATCTCAACAAACAGTTATATGACATCATCGCCATGCGCTATGCAGACAAGCACCTCAACATCGACTTTGACAGTTACATCTGCTGTTTCGTAAGACTGGAGGGCATGTTCAGAGCGTT
CAATGCGTTTGATAAAGATGGAGATGGAATAATCAAACTCAACGTGCTCGAGTGGCTTCAGCTGACCATGTACTCT

ACCAACTCCATCTACTCGGCGATTCTGAGCAGAAACGAGGCCGTCAAGCTCAAGACGTTTTTGGAGCTGCGGGACAAGTACGTGAAGAAGAACGTGGTGTTTGAAGACCCCCTGTTCCCCGCAAATGACTCCTCACTC------
------

TGGAAGCGTCCCTCGGAAATTTGTGAAAACCCCCAGTTCATCATAGACGGAGCCAAAAGGACAGACATCTGTCAAGGAGAATTGGGTGACTGCTGGCTGCTGGCTGCCATCGCCTGTCTGACCGTCAACGAGAAGCTGCTGTACC
GAGTGATTCCCCCGGACCAGAGCTTCACCGACAACTACGCCGGCATTTTCCATTTCCAGTTCTGGCGCTATGGCGAATGGATCGATGTTATCGTGGACGATCGCATCCCCACCTGCAACAACCATCTGGTGTTCACCAAATCTTT
CAGAAAGAACGAGTTCTGGAGCGCTCTTCTGGAAAAAGCTTATGCCAAGTTGCACGGCTCTTACGAAGCCCTGAAAGGGGGCAACACTTTGGAGGCCATGGAGGATTTCACTGGCGGGGTTACTGAGTTCTTCGACTTGCCCGAG
GCGCCCAAAGAGCTCTACAGCATCATGAGGAAGGCGCTGGAGAGAGGCTCGCTCATGGGCTGCTCCATAGAT-----------------------------------------------------------------------------
---------------------------------------------------------------------------GAGACCCGGACGGATCAGGGGCTGGTCAGGGGTCACGCCTACTCCATCATAGGTTTG------------
AAGGACACCACAATTCGCCTGATTCGCCTGCGTAATCCCTGGGGTTGGGTGCTGTGGAAAGGCCCATGGAGCGCAAACTCGACAGAGTGGTCGACCATTTCCATCGCAGATAAGGACAACCTGATTAAACAGACTGTACAGGAGA
GCGAGTTCTGGATGTCATTCGCTGATTTCACGAGGAACTTCACCAAGCTGGAGATGTGTAACTTGACCCCCGACGCCCTGGGCTGCGATGAAAGACAAAGCTGGACGGTGTCGGTCAACGAGGGTCGGTGGGTGAGAGGCAGCTC
CGCCGGCGGCTGCAGGAACTTCCCACAAACCTTCTGGACGAACCCTCAGTACCGGCTGCAACTGTACGAGGACGACGACGACCCGGAGGACGGGCAGGCGGCCTGTACCGTTGTCGTGGCTCTGATGCAGAAAGGCCGGAGGATG
CAGCGCCACCAAGGGGCTAAATTCCTCACCATCGGGTTTTCAATCTACGAGGTGCCGAAGGAGATGTGCGGACAAAATCAACACCTGCAGAAGGACTTCTTCCTGTACACCGCCTCCAAAGCCAAATGCAAGACCTACATTAACC
TGCGGGAGGTGACGGAGCGTGTGCACCTGCCTCAGGGAGAGTACGCCATCATCCCCACCACCTTTGAGCCCCACCAAGACGGAGAGTTCATCCTCAGGGTGTTCTCGGAGAAGAAGAGCACGTCTGAGGAAGTGGAGGACACAAT
TGGGTCTGAGAAACGA---------------------------------------------------------------------------------------------------------------------
CTCCAAGAGGAGGAGACCGAAGAGGAAAAACATTTCAGAGCCATTTACCAAAAGGTTGCCGGCGAGGACATGCAGATCTGCGCCAACGAGCTCAGAACGATCATGAGAAACGTGCTCGCCAAACATAAC---
ATGAAGACGGAAGGCTTCATGATCGGGACGGGAAAGCTGAACCTGCAGGAGTTCAAACACTTGTGGAAAAAGATCAAGGAGTGGCAGCTGATCTTCAGACGATACGACGAAAACAAGACCGGCTGCGTCAGCAGCTTCGAGATGA
GGAACGCCGTCAATGACGCAGGGTTTCACCTCAACAAGCAGCTGTACGACATCATCGCCATGCGCTACGCAGACGAACATCTCAACATCGACTTTGACAGTTATATCTGCTGCTTTGTGAGACTGGAGGGCATGTTCAGAGCTTT
TAATGCCTTCGACAAAGATGGAGATGGAATAATAAAGCTCAATGTCCTGGAGTGGCTTCAGCTGACGGTGTATTCT

TCAAACTCCATCTACTCAGCAATTCTCAGCAGGAATGAGGCCGTCAAACTTAAGACCTTTCTGGAGCTGCGAGACAAATATGTGGAGAAGAATGTTATGTTTGAAGACCCTCTGTTCCCCGCAGATGACTCCTCGCTT------
------

TGGAAACGTCCATCGGAAATTTGTGAAAACCCCCAGTTCATCATAGATGGAGCCAACAGAACAGACATCTGTCAGGGAGAATTAGGTGACTGCTGGCTGCTGGCTGCCATCGCCTGTCTCACGCTGAACGAGAAGCTGCTGTACA
GAGTTATTCCCCCGGATCAGAGCTTCACAGAAAACTACGCCGGCATCTTCCACTTCCAGTTCTGGCGTTATGGCGAGTGGATCGATGTCGTCGTGGACGACCGCATTCCCACCTGCAACAACCAGCTGGTTTTCACAAAGTCTTT
CAGAAAGAACGAGTTCTGGAGCGCTCTTTTGGAAAAAGCTTATGCCAAGTTGCACGGGTCTTATGAGGCACTGAAAGGAGGGAACACTTTGGAAGCCATGGAGGATTTCACAGGTGGAGTTACCGAGTACTTTGAATTGTCTGAT
GATCCCACAGAGCTCTACAAGATCATGAAGAATGCGCTGGAGAGAGGCTCACTGATGGGCTGCTCCATAGAT-----------------------------------------------------------------------------
---------------------------------------------------------------------------GAGAGTCGGACTGAGCAGGGGCTGGTCAGGGGTCATGCCTATTCCATCATAAGTCTG------------
AAGGACACAAGAATTCGCCTGATTCGTTTGCGCAATCCCTGGGGTTTTGTGCTCTGGAAAGGACCCTGGAGTGCAAATTCAAAGGAGTGGTCGACCATTTCCGCTGCAGACAGAGAAAACCTGAAAAAACAGACCATAGAGGCAA
GTGAGTTCTGGATGTCCTTTGCTGATTTCAAGAAGAATTTCACCAAGCTGGAGATGTGCAACCTGACCCCCGACACGCTGCAGGGGGATGAAAGAAACACCTGGACAGTGTCGGTCCACGAGGGTCGCTGGGTGAGAGGAAGCTC

```
TGCTGGTGGCTGTAGGAACTTCCCAGACACGTTTTGGACAAACCCTCAGTATCGTCTGCAGCTGTATGAGGAGGACGATGACCCGGATGACGAGCAGGTGGCCTGCACTGTTGTCGTGGCACTGATGCAGAAAGGTCGAAGGATG
CAGCGCCATCAAGGGGCCAAATTTCTCACCATAGGCTTTTCCATCTACGAGGTGCCGAAGGAGATGTGTGGGCAGAATCAGCATCTGCAGAAGGACTTCTTCCTCTACACAGCCTCCAAGGCAAAATGCAAGACTTACATTAACC
TGCGGGAGGTAACGGAGCGTTTCTGTCTGCCTCCCGGTGAGTACGTCATCATCCCCACAACCTTTAAACCCCATGAAGAAGGAGAGTTCATCCTCAGGGTCTTCTCTGAGAAGCAGAGCACATCAGAGGAGGCAGAGACTACGAT
CGGATCAGATCAACGG---------------------------------------------------------------------------------------------------------------CTCCAA---
GAGGAAACCGAGGAGGAAAAGCAGTTCAGGGCTATTTACCAACAGATTGCTGGTGATGACATGCAGATCTGTGCCAATGAACTCAAAACGATCTTAAAAAACGTGCTTTCCAAACATAAT---
ATAAAGTCAGAAGGTTTCATGATCGGGACAGGAAAGCTGAATCTGCAGGAGTTCAAACACTTGTGGAAAAAGATCAAGGACTGGCAGCTCATCTTCAAACGTTACGACAAAGATAAAAACTGGTCTATCAGTAGTTTCGAGATGA
GGAATGCTGTTAATGATGCAGGGTTTCAACTCAACAGACAACTGTATGACATCATAGCCATGCGCTATGCAGATGAACGCCTGAACATTGATTTTGATAGTTACATCTGCTGCTTTGTGAGGCTAGAAGGCATGTTCAGAGCATT
CAATGCCTTTGACAAAGATGGAGATGGAATAATCAAGCTCAATGTCCTGGAGTGGCTTCAGCTGACGATGTACTCA;

END;



BEGIN TREES;

        TREE                                                                tree                                                                     =
((((((((GALLUS_GALLUS_ENSGALG00000009050,MELEAGRIS_GALLOPAVO_ENSMGAG00000011468),TAENIOPYGIA_GUTTATA_ENSTGUG00000011081),(ANOLIS_CAROLINENSIS_ENS
ACAG00000015493,PELODISCUS_SINENSIS_ENSPSIG00000011344)),((((CERATOTHERIUM_SIMUM_XM_004421467,OVIS_ARIES_EU846598),SUS_SCROFA_ENSSSCG00000004728)
,(HOMO_SAPIENS_AF127765,MUS_MUSCULUS_ENSMUSG00000079110)),(MONODELPHIS_DOMESTICA_ENSMODG00000017909,SARCOPHILUS_HARRISII_ENSSHAG00000007516))),XE
NOPUS_TROPICALIS_XM_004917239),LATIMERIA_CHALUMNAE_ENSLACG00000003659),((((OREOCHROMIS_NILOTICUS_ENSONIG00000006073,ORYZIAS_LATIPES_ENSORLG000000
12402),GASTEROSTEUS_ACULEATUS_ENSGACG00000009830),GADUS_MORHUA_ENSGMOG00000000192),(LEPISOSTEUS_OCULATUS_GENSCAN00000022004,(DANIO_RERIO_ENSDARG0
0000041864,(GADUS_MORHUA_ENSGMOG00000013176,((TAKIFUGU_RUBRIPES_ENSTRUG00000006900,GASTEROSTEUS_ACULEATUS_ENSGACG00000005212),(XIPHOPHORUS_MACULA
TUS_ENSXMAG00000009351,ORYZIAS_LATIPES_ENSORLG00000018135))))))),SCYLIORHINUS_CANICULA_TRANSCRIPTOMECONTIG17411);

END;
```

## (*g*) *CAPN8*

```
#NEXUS


BEGIN TAXA;

        DIMENSIONS NTAX = 23;

        TAXLABELS

                'ANOLE_CAPN8_ENSACAG00000002512_ENSACAT00000003160'                              'TURTLE_CAPN8_ENSPSIG00000014086_ENSPSIT00000016134'
'TURKEY_CAPN8_ENSMGAG00000008330_ENSMGAT00000009604'      'CHICKEN_CAPN8_XM_426117'      'FALCON_CAPN8_XM_005241871'      'HUMAN_CAPN8_NM_001143962'
'RHINO_CAPN8_XM_004439538'        'SHEEP_CAPN8_XM_004013988'        'MOUSE_CAPN8_ENSMUSG00000038599_ENSMUST00000048941'        'DOG_CAPN8_XM_849757'
'PLATYPUS_CAPN8_ENSOANG00000001449_ENSOANT00000002296'                                   'OPOSSUM_CAPN8_ENSMODG00000005003_ENSMODT00000006284'
'TASMANIAN_DEVIL_CAPN8_ENSSHAG00000014684_ENSSHAT00000017417'                              'XENOPUS_CAPN8L_ENSXETG00000031732_ENSXETT00000062202'
'XENOPUS_CAPN8_ENSXETG00000018607_ENSXETT00000062268'      'X_LAEVIS_CAPN8_NM_001088543'      'MEDAKA_PCAPN2_ENSORLG00000011856_ENSORLT00000014856'
'PLATYFISH_CAPN2OR8_ENSXMAG00000006017_ENSXMAT00000006157'                            'TILAPIA_CAPN2OR8_ENSONIG00000009988_ENSONIT00000012572'
'ZEBRAFISH_CAPN8_ENSDARG00000055715_ENSDART00000078111'                               'TILAPIA_CAPN2OR8_ENSONIG00000007284_ENSONIT00000009192'
'XENOPUS_CAPN8_ENSXETG00000030338_ENSXETT00000061954' 'X_LAEVIS_CAPN8_NM_001085983' ;
```

END;

BEGIN CHARACTERS;

DIMENSIONS NCHAR = 2334;

FORMAT
DATATYPE = DNA

GAP=-

MISSING=?

NOLABELS

;

MATRIX

```
 ---------------------ATGACCAGC---------------------------GGCTTGGGTTCCAATGGGAAGGCAATAAAATACCTAAAACAGGATTTTGAGGAGCTAAGG---------------
AGGCAGTGCTTAAAGTCTGGTACCCTGTTTAAAGATGAAGAATTCCCTGCTTGTCCTTCTGCTCTTGGCTACAGAGATTTGGGGCCGTATTCTCCCAAAACCCAAGGCATTATCTGGAAACGACCCTCGGAGCTAGTTCCAAAT-
--
CCTCAATTCATTGTGGGAGGAGCTACTCGTACAGATATCCGTCAGGGAGCATTGGGAGACTGCTGGCTTTTGGCAGCCATTGCTTCTCTCACATTGGATCAAGAAATATTAGACCGAGTTGTCCCCAAAGACCAAAGCTTCCAGA
AAGACTATGCTGGGATCTTTCATTTCCAGATCTGGCAATATGGAGAATGGGTGGATGTTGTCGTTGATGACCGGCTCCCCACGAAAAATGGTCAGCTTCTTTTTCTGCACTCTGAAGAAGGGAATGAGTTCTGGAGTGCACTGTT
GGAGAAGGCCTATGCCAAGTTGAATGGTTCCTATGAGGCTCTTACAGGAGGATCTACTATA------
GAAGGTTTTGAAGATTTTACTGGTGGCATCGCTGAATCTTATGACTTGAGGAAAGCTCCATCTAATCTGTATCAAATCATACAAAAGGCCTTGCGGTCTGGTTCCCTGTTGGGTTGTTCCAAAATGCTTCCCTCTGTAAGGAAAT
CCTTCCTGAATTTGCAGATGGCACAAAATTGCAAGGAACAGTTTACACTTCAAAAAACCCTGAATAGAGGTATATCAAATGAAAGAAAAATTGCTAGTGCAGCTGAA------
ACAGAAGCAATCACAAGGCTCAAACTGGTCAAAGGTCATGCATACTCTGTTACTGGAGCTGAAGAGGTTAACTACCGCGGGCAGCCAGTGAAACTTCTCAGAATAAGAAATCCATGGGGTGAAGTGGAGTGGACTGGAGCCTGGA
GTGATAATGCTCCAGAATGGAATTATATTGACCCCAAGGAAAAGAATGCTCTGGATAAGCAAGCCGATGATGGGGAATTTTGGATGTCCTTGGCCGACTTTCAAAGGCAGTTTACGAGGCTTGAGATCTGCAACTTGACCCCCGA
CACCCTGACAAGCAATCACCTTCATAAGTGGGGCATGACATTGTTCAATGGACACTGGCGGCGGGGCTCTACAGCCGGGGGATGTCAGAACTATCCAGCAACATACTGGACCAACCCACAGTTTAAAATCAGTCTGGATGAGCCG
GACGATGAC---GAACCATCTTGTACGGTGCTGATTGGCTTGATGCAGAAGAATCGCAGGAGGCAAAAGAGGATGGGGGAAGCGCTGCTCAGTATTGGCTACTCCATTTATCAGGTTCCAAAA---
TTGGAAAATCAAACAGATGTTCATCTAAACAGAGATTTCTTCTCAACGAATAGATCTGTAGCTCGCTCTGACACGTATGTCAACTTGCGTGAAGCCTCAAATCGATTCCACCTGCCTCGGGGAGAATACCTGGTTGTGCCATCCA
CCTTTGAACCTTTTAAAGATGGAGAGTTCTGTCTCAGAGTCTTCTCTGAGAAAAAA------------------------------------------------------------------------------
TTCAAGAATCTGTTTGAAAAGCTTTCTGGG---------------------------------------------------GAGGAT------------
TGTGAAATGACTGCAAATGAGCTTCAGACTATTTTGAACAGGGTGATAACA---------------------AAG---
CGAACAGACATTAAAAGTGATGGATTCAATCTAAACACCTGCCGGGAAATGATCAGCCTCTTAGATAACCATGGCACTGGAAGCTTGGGAGTTGCAGAGTTCAAGATACTATGGATGAAGATTCAGAGATATCTGGAAATCTATA
AGAAAGTAGATACTGACCACTCTGGAACTATAGATGCACATGAAATGAGAGATGCTCTCAAGGAAGCA---GGTTTC---
ACTCTCAACAACAAAGTCGCTGCCCGCTATGCCTGCAGCAAGCTGACCATTGACTTTGATGGCTTCCTGGCCTGCATGATTCGCCTGGAAACCCTGTTTAAAATGTTTCAGATGTTAGACAAGGAAAAACGTGGAACTGTCCAGC
TCTCTCTGGCTGAG------TTT------CCCCAACTGAGTTTATATGCC---

 ---------------------ATGGCAGGA---------------------------GGGCTCGGCTCCAATGAGAAGGCAGTGAAGTATTTAAATCAGGATTATGAGGTTTTGAGG---------------
CAGCAGTGCTTGCAGTCTGGTTCTCTATTTAAGGATGAAGAATTCCCAGCCTCTCCTTCTTCTCTGGGCTATAAGGACCTGGGGCCACATTCCCCCAAAACGCAGGGTATTGTCTGGAAGCGACCCACGGAGTTTTGTGCCAAT-
--
CCTCAGTTTATAGTTGGAGGAGCAACTCGGACTGATATTCGCCAAGGGGACCTGGGTGACTGCTGGCTCCTAGCAGCCATTGCATCTCTTACCTTAGATCAGGAAATCCTACAGCGAATTGTTCCTGAAGATCAGAGCTTCCAGA
AGAACTATGCTGGGATCTTTCATTTCCAGTTCTGGCAGTTTGGGGAGTGGGTGGATGTTGTTGTTGACGACAGGCTGCCCACTAAAAACGGGCAGCTGCTCTTTCTACACTCAGAGGAAGGCAATGAGTTCTGGAGTGCCCTGCT
GGAGAAGGCCTATGCTAAATTGAATGGCTCATATGAAGCTCTTGCAGGAGGTTCCACTGTA------
```

GAAGGCTTTGAAGATTTCACTGGAGGGATCGCTGAGTCGTATGATCTGAGGAAGGCACCACCCAATCTATATCAGATCATTCGGAAGGCCCTGCAATCTGGGTCATTGCTTGGCTGCTCC-------------------------
-------------------------------------------------------------------------------------------ATCACCAGTGCAGCTGAA------
ACGGAGGCAATCACAAGTAAGAAATTGGTAAAGGGACATGCTTACTCTCTCACTGGAGCAGAAGAGGTAAATTATCGCGGGCGCTTAGAGAAGCTAGTCAGGATTAGAAATCCTTGGGGTGAAGTGGAATGGACCGGAGCCTGGA
GCGATAATGCTCCAGAATGGAATTATGTTGATCCCAAACAAAAACAGGCTCTGGATAAGCAATCGGATGATGGAGAATTTTGGATGGCATTCTCAGATTTTCAAAGACAGTTCACACGGCTTGAGATCTGCAACTTGACTCCTGA
CACCCTGACGAGTGACAAAGTCCATAAATGGGGTCTGACCTTGTTCAGTGGTCAGTGGAGACGGGGTTCTACTGCTGGGGGCTGCCAGAACTATCCAGCAACATACTGGATCAATCCCCAGTTTAAAATCAGGCTGGATGAGCCA
GATGATGAT---AAGCCATGCTGCACTGTGCTGGTGGGCTTGATGCAGAAGAACCGCAGGAGAGTGAAGAAGATGGGGGAATCTCTACTCACTATTGGCTATTCCCTCTATCAGGTCCCTAAA---
CTGCAAAATCACACGGATGTCCATCTGAGCCGAGATTTCCTCACAAGGCACCGACCAGTAGCCCGGTCTGACACATATGTTAACCTGCGTGAAGTCTCCGGCCGTATCAAATTGCCCCAAGGAGAGTATCTCGTTGTGCCGTCTA
CATTTGAGCCTTTCAAAGATGGAGAGTTCTGCCTTCGGGTTTTCTCTGAGAAGCAG-------------------------------------------------------------------------------
TTCAGGAGCTTGTTTCAGAAGCTCTCTGGA---------------------------------------------------GAGGAT------------
TATGAAATGAGTGCAGATGAACTTCAGACTGTTCTGAACAGGGTGTTAACA---------------------AAG---
AGGGCAGACATTAAAACTGATGGATTCAACATAAACACCTGCAGAGAGATGATCAGTCTCTTAGACACCAACGGGACTGGCACCTTGGGACTTGTAGAATTCAAGATACTTTGGCTGAAGATTCAAAAATATTTGGAGATCTATA
AGAAAGTGGACACCGACTATTCTGGTACCATAGATGCCCATGAGATGCGAGATGCCCTCAGAGAAGCA---GGTTTC---
ACTCTCAACAACAAGGTGGCCACCCGCTATGTCAACAGCAAGCTGACCATTGACTTTGACAGCTTTGTGGCCTGCATGATCCGCCTGGAGACCCTCTTCAAAATGTTCCAGATTCTAGACAAAGACAAGAATGGTGTTGTCCAGC
TCTCTTTGGCTGAG------TGG------TTATGCTGCACGATGGTT------

---------------------ATGGCTGGG---------------------------GGGCTGGGTTCCTGCAGAAAGGCCCTCAGCTACTTGGGCCAGGACTACGAGGCACTGAGG---------------
CAGAAGTGCCTTCAGGCTGGGACTCTATTCAAGGATGAGGAGTTCCCTGCCTGCCCGTCTGCACTGGGATACCAGGACCTGGGACCTTACTCCTTCAAAACCCAGGGAATAGTCTGGAAGCGGCCTACGGACTTATGTGACAAT-
--
CCTCAGTTCATAGTTGGAGGAGCTACTCGGACAGATGTTTGCCAAGGAGAGCTGGGTGACTGCTGGCTCCTGGCTGCCATTGCATCTCTGACTTTGAATCCAGATGTTCTGCACCGCGTTGTTCCCGAGGCTCAGAGTTTCCAGG
AGGACTATGCTGGGATCTTTCATTTCCAGTTCTGGCAGTACGGAGAGTGGGTGGACGTGGTGGTAGACGACCGGCTACCCACCAAGAATGGGGAGCTGCTCTTCGTGCATTCAGAGGAAGGCAATGAATTCTGGAGCGCACTGCT
GGAGAAAGCCTACGCCAAGCTGAATGGTTCTTATGAAGCTCTAGCTGGAGGGTCCACTATA------
GAGGGGTTTGAGGATTTTACTGGAGGCATCTCTGAATCCTATGACCTGCGGAGGGCTCCAGCAAATCTATACCAAATCGTCCAGAAGGCCCTGAGGGCTGGCTCTCTGCTCGGCTGTTCC-------------------------
-------------------------------------------------------------------------------------------ATAACTTGTGCAGCTGAA------
ACAGAAGCAATCACGAGTCTGAAGCTGGTGAAGGGACATGCTTATTCTGTCACTGGGGCCGAAGAGGTCTATTACCAAGGACAGCCAGAGAAACTTGTGAGGCTTAGAAATCCCTGGGGTGAAGTAGAATGGACTGGAGCGTGGA
GTGATAATGCTCGTGAATGGAATTATATTGATCCCAAACAAAAACAGGCTCTGGATAAGCAGTAGATGATGGAGAATTTTGGATGGCATTTTCTGATTTTCAAAGGCAATTCACCCGCCTGGAGATCTGCAACCTGACTCCTGA
CACACTGACAAGCAACCAGGTCAACAAATGGGACCTGACCGTGTTCAATGGACAGTGGATACGGGGTTCAACAGCTGGAGGTTGCCAGAACTACCCAGCAACATACTGGATCAATCCCCAGTTTAAAATCCGGCTGGATGAACCA
GATGATGAT---GAGCCATGCTGTACTATATTGGTGGGCTTGATGCAGAAGAACCGCAGGAGACAGAAGAGAATGGGAGAAGGTCTGCTTAGTATTGGTTATTCACTCTATCAGATTCCACAG---
CTGGAAAACAACAAAGACATCCATCTGAACCGTGCTTTCTTCACAAGGAACCAACCAATAGCCCGGTCTGAGACTTACATCAACCTGCGTGAAGTCTCCAGCCGCATTAAATTGCCCAAAGGAGAGTACCTCATTGTGCCATCCA
CATTTGAGCCTTACAAAAATGGAGAGTTCTGCCTTCGAGTTTTTTCTGAGAAAAAG-------------------------------------------------------------------------------
TTCAAGACTCTATTTCAAAAGCTCTCTGGA---------------------------------------------------GAGGAC------------
TGTGAAGTGACTGCGACTGAACTTCAAACTATTCTAAATAGGGTTTTGGCA---------------------AAG---
AGAAAAGATATAAGAAGTGATGGATTCAATATTAACACTTGCAGGGAGATGATCAGCCTTTTAGACACTAATGGGACTGGCTCCTTGGGGCTTATAGAATTCAAGACACTTTGGATGAAGATTCAGATGTATTTGGCAATCTATA
GGAAGGTGGACAGAGACTACTCAGGTACCATTGACTCCCATGAGATGCGAAATGCTCTCAGCGAGGCA---GGTTTT---
GTGCTCAACAACCAGGTGGCCATCCGCTATGCCTGCAGCAAGATGACCATTGATTTTGACGGTTTTGTGGCCTGCATGATCCGCTTAGAGACCTTGTTCAAAGTGTTTCATCTCCTAGATAAAGAGAAGAGTGGAGTTGTTCAGC
TTTCCTTAGCAGAG------TGG------CTGTGCTGCACACTGGTT------

---------------------ATGGCTGGG---------------------------GGGCTGGGTTCCTACAGGAAGGCCATCAGCTACTTGGGCCAGGACTACGAGGCACTGAGG---------------
CAGAGGTGCCTTCAGACTGGGACTCTGTTCAAGGATGAGGAGTTCCCTGCCTGCCCATCTGCACTGGGATACCAGGACCTGGGACCCTACTCCTTCAAAACCCAGGGAGTAGTCTGGAAACGGCCCACGGAGTTATGTGACAAT-
--
CCTCAGTTCATAGTCGGAGGAGCTACTCGGACAGATGTTTGCCAAGGAGAGCTGGGTGACTGCTGGCTCCTGGCTGCCATTGCATCTCTGACTTTGAATCCAGATGTTCTTCACCAAGTTGTTCCCAAGGCTCAGAGTTTTCAGG
AGAACTATGCTGGGATCTTTCATTTCCAGTTCTGGCAGTATGGAGAGTGGGTGGATGTGGTGGTAGACGACCGGCTGCCCACCAAGAACGGGGAGCTGCTCTTCGTGCATTCAGAGGAAGGCAATGAGTTCTGGAGCGCACTGCT
GGAGAAAGCCTACGCCAAGCTGAATGGTTCTTATGAAGCTCTAGCTGGAGGGTCCACTATA------
GAGGGGTTTGAGGATTTTACTGGAGGCATCTCTGAATCCTATGACCTGCGGAGGGCTCCAGCAAACCTGTTCCAAATTGTCCAGAAGGCTCTGAGGGCTGGCTCACTGCTCGGCTGTTCC-------------------------
-------------------------------------------------------------------------------------------ATAACTCGTGCATCTGAA------
ACAGAAGCAATCACGAGTCTGAAGCTTGTGAAAGGACATGCTTATTCTGTCACTGGGGCAGAAGAGGTCTATTACCGAGGACGGCCAGAGAAACTTGTGAGGCTTAGAAATCCCTGGGGTGAAATAGAATGGACTGGAGCGTGGA
GTGATAATGCTCCCGAATGGAATTATATTGATCCCAAACAAAAGCAGGCTCTGGATAAGCAAGTAGATGATGGAGAATTTTGGATGGCGTTTTCTGATTTCAAAGGCAGTTCACCCGTCTGGAGATCTGCAACCTGACTCCTGA
CACACTGACAAGCAATCAGGTCAACAAATGGGACCTGACCATGTTCAATGGACAGTGGATACGGGGTTCAACAGCTGGAGGTTGCCAGAACTACCCAGCAACATACTGGATCAATCCCCAGTTTAAAATCCGGCTGGATGAACCA
GATGATGAT---GAGCCATGCTGTACTATATTGGTGGGCTTGATGCAGAAGAACCGCAGGAGACAGAAGAGAATGGGAGAAGGTCTGCTTAGTATTGGTTATTCACTCTATCAGATTCCACAG---
CTGGAAAATAACAAAGACATTCATCTGAACCGTGCTTTCTTCACAAGGAACCAACCAGTAGCCCGGTCTGAGACTTACATCAACCTGCGTGAAGTCTCCAGCCGCATTAAATTGCCCAAAGGAGAGTACCTCATTGTGCCATCCA

```
CATTTGAGCCTTACAAAAATGGAGAGTTCTGCCTTCGAGTTTTTTCTGAGAAAAAG------------------------------------------------------------------------------
TTCAAGACTCTATTTCAAAAACTCTCTGGA---------------------------------------------------GAGGAC------------
TGTGAAGTGACTGCAACTGAACTTCAAACTATTCTAAATAGGGTTTTGGTG---------------------AAG---
AGAAAGGATATAAGAAGTGATGGATTCAACATTAACACTTGCAGGGAGATGATCAGCCTTTTAGATACTAATGGGACTGGCACCTTGGGACTTATAGAATTCAAGACACTCTGGATGAAGATTCAGATGTATTTGGCAATCTACA
GGAAGGTGGACAGAGACTACTCGGGTACCATTGACTCCCATGAAATGCGAAATGCACTCAGCGAGGCA---GGTTTT---
GTGCTCAACAACCAGGTGGCCATCCGCTACGCCTGCAGCAAGATGACCATTGATTTTGATGGCTTTGTGGCCTGCATGATCCGCTTAGAGACCCTGTTCAAAGTGTTTCATCTCCTAGATAAAGAGAAGAGTGGAGTTGTTCGGC
TCTCTTTAGCAGAG------TGG------CTGTGCTGCACACTCGTT------

ATGGAGGCACACGGGAAATCAATGGCACATGGAGAACTGGAAGCAGATCAAGTAATGTGCGTGCACTCAATTACTGCTACGCCATCAGTCAAAGTCTCCAGCATGGCTGTGCTGAAGCTCCAGCAGAACACGCAGCAGTGCCTGC
AGGCTGGCACCCTGTTCAAGGATGAGGAGTTCCCAGCCTGTCCCTCCGTGCTGGGCTACCAGGACCTGGGACCATACTCCTTCAAAACCCAGGGGATAATCTGGAAGCGCCCCACGGAGTTATGTGCCAAC---
CCTCAGTTTATAATTGGAGGAGCTACTCGAACAGATGTCTGCCAAGGAGAGCTGGGTGATTGCTGGCTTCTAGCTGCCATTGCATCTCTCACTTTGAATCCAGATGTTCTGTACCGTGTTGTTCCCAAGGCTCAGAGTTTCCAGA
AGGATTATGCTGGGATCTTTCATTTCCAGTTCTGGCAGTACGGGGAGTGGGTGGATGTTGTAGTGGACGACAGGCTGCCCACCAAGAATGGGAAGCTGCTCTTCGTGCACTCGGAGGAAGGCAACGAGTTCTGGAGCGCGCTGCT
GGAGAAGGCCTACGCCAAGTTGAATGGCTCTTATGAAGCTCTCACAGGAGGGTCCACTATG------
GAGGGCTTTGAGGATTTTACTGGAGGCATCTCTGAGTCCTATGAGCTGCGGAGGGCTCCTTCAAACCTTTACCAAATCATCCAGAAGGCTCTGAGAGCTGGCTCGCTGCTTGGCTGTTCC-------------------------
-----------------------------------------------------------------------------------------ATAACTACTGCAGCTGAA------
ATAGAAGCAATTACAAGTCTGAAGCTGGTAAAGGGACACGCTTACTCTGTCACTGGAGCAGAGGAGGTATATTACCGAGGACAGCCAGAGAAACTAGTGAGGCTTAGAAATCCCTGGGGTGAAGTGGAATGGACTGGAGCTTGGA
GTGATAATGCTCCTGAATGGAATTATGTTGATCCCAAACAAAAATGGGCTTTGGATAAGCAAGTGGATGATGGAGAGTTTTGGATGGCATTTTCGGATTTTCAAAGGCAGTTCACCCGCCTTGAGATCTGCAACTTGACTCCTGA
CACGCTGACAAGCAATGAGGTCAACAAATGGGACCTGACCCTGTTTAATGGACAGTGGAGACGGGGTTCGACTGCTGGGGGCTGCCAGAACTACCAAGCAACGTACTGGATCAATCCCCAGTTTAAAATCCGGTTGGACGAACCA
GACGATGAC---GAGCCATGCTGTACTATACTGGTGGGCTTGATACAGAAGAACCGCAGGAGACAGAAGAAAATGGGAGAAGCTCTGCTTAGTATTGGTTACTCACTCTATCAGATACCTCAG---
CTGGAAAATGACATAGATATTCATATGAACCGTGCTTTCTTCGCAAGGAACCAACCAGCAGCCCAGTCTGGTCCTTATGTCAACCTGCGTGAAGTCTCCAGACGCATGAAGTTGCCCCGAGGAGCATACCTCATTGTGCCATCCA
CTTTTGAGCCTTACAAGAATGGAGAGTTCTGCCTTCGAGTTTTTTCTGAGAAACAG------------------------------------------------------------------------------
TTCAAGATTCTGTTTCAAAAGCTCTCTGGA---------------------------------------------------GAGGAC------------
TGTGAAGTGACTGCAACTGAACTTCAAACTATCCTAAACCGGGTTTTGTCA---------------------AAG---
AGAAAGGATATTAAAAGCAATGGATTCAACATTAACACTTGCAGGGAGATGATCAGCCTTTTAGATACCAATGGGACTGGCACCTTGGGACTTGTAGAATTCAAGACACTTTGGATGAAGATTCAAAAGTATTTGGCAATCTATA
AGAAAGTGGACAGTGACTACTCCGGTACCATCGACTCCCATGAGATGCGAAATGCCCTCAGAGAGGCA---GGTTTC---
ACGCTCAACGATCAGGTGGTCATCCGCTATGCCTGCAGCAAGCTGACCATTGATTTTGACGGCTTTGTGGCCTGTATGATCCACTTGGAGACCCTGTTCAAAGTGTTCCATCTCCTGGACAAAGACAAGCGTGGAGTCATCCAGC
TCTCTCTGGCTGAG------TGG------CTGTGCTGCACAGTGGTT------

 ---------------------ATGGCAGCC---------------------------GGTCTTGGCTCCAACCAAAACGCTTTGAAGTACTTGGGCCAGGATTTCAAGACCCTGAGG---------------
CAACAGTGCTTGGACTCAGGGGTCCTATTTAAGGACCCTGAGTTCCCAGCATGTCCATCAGCTTTGGGCTACAAGGATCTTGGACCAGGCTCTCCGCAAACTCAAGGCATCATCTGGAAGCGGCCCACGGAGTTGTGTCCCAGC-
--
CCTCAGTTTATCGTTGGTGGAGCCACGCGCACAGACATTTGTCAGGGTGGTCTAGGTGACTGCTGGCTTCTGGCTGCCATTGCCTCCCTGACCCTGAATGAAGAGCTGCTTTACCGGGTGGTCCCCAGGGACCAGGACTTCCAGG
AGAACTATGCGGGAATCTTTCACTTTCAGTTCTGGCAGTACGGAGAGTGGGTGGAGGTGGTCATTGACGACAGGCTGCCCACCAAGAATGGACAGCTGCTCTTCCTACACTCGGAACAAGGCAATGAATTCTGGAGTGCCCTGCT
GGAGAAAGCCTATGCCAAGCTTAATGGTTGTTATGAGGCTCTCGCTGGAGGTTCCACAGTG------
GAGGGGTTTGAGGATTTCACAGGTGGCATCTCTGAGTTTTATGACCTGAAGAAACCACCAGCCAATCTATATCAGATCATCCGGAAGGCCCTCTGTGCGGGGTCTCTGCTGGGCTGCTCC-------------------------
-----------------------------------------------------------------------------------------GTCTCCAGTGCAGCCGAA------
GCCGAAGCCATCACCAGCCAGAAGCTGGTTAAGAGTCATGCGTACTCTGTCACTGGAGTCGAAGAGGTGAATTTCCAGGGCCATCCAGAGAAGCTGATCAGACTCAGGAATCCATGGGGTGAAGTGGAGTGGTCGGGAGCCTGGA
GCGATGATGCACCAGAGTGGAATCACATAGACCCCCGGCGGAAGGAAGAACTGGACAAGAAAGTTGAGGATGGAGAATTCTGGATGTCACTTTCAGATTTCGTGAGGCAGTTCTCTCGGTTGGAGATCTGCAACCTGTCCCCGGA
CTCTCTGAGTAGCGAGGAGGTGCACAAATGGAACCTGGTCCTGTTCAACGGCCACTGGACCCGGGGCTCCACAGCTGGGGGCTGCCAGAACTACCCAGCCACGTACTGGACCAATCCCCAGTTCAAAATCCGTTTGGATGAAGTG
GATGAGGAC---GAACCCTGCTGTACAGTGCTGCTGGGCCTGATGCAGAAAAATCGCAGGTGGCGGAAGCGGATAGGACAAGGCATGCTTAGCATCGGCTATGCCGTCTACCAGGTTCCCAAG---
CTGGAGAGTCACACGGACGCACACTTGGGCCGGGATTTCTTCCTGGCCTACCAGCCCTCAGCCCGCACCAGCACCTACGTCAACCTGCGGGAGGTCTCTGGCCGGGCCCGGCTGCCCCCTGGGGAGTACCTGGTGGTGCCATCCA
CATTTGAACCCTTCAAAGACGGCGAGTTCTGCTTGAGAGTGTTCTCAGAGAAGAAG------------------------------------------------------------------------------
TTCAGGAGGCTGTTTGAGAAGTTGGCAGGG---------------------------------------------------AAGGAT------------
TCTGAGATTACTGCCAATGCACTCAAGATACTTTTGAATGAGGCGTTTTCC---------------------AAG---
AGAACAGACATAAAATTCGATGGATTCAACATCAACACTTGCAGGGAAATGATCAGTCTGTTGGATAGCAATGGAACGGGCACTTTGGGGGCGGTGGAATTCAAGACGCTCTGGCTGAAGATTCAGAAGTATCTGGAGATCTATT
GGGAAACTGATTATAACCACTCGGGCACCATCGATGCCCACGAGATGAGGACAGCCCTCAGGAAGGCA---GGTTTC---
ACCCTCAACAGCCAGGTGGCCCTGCGGTATGCGTGCAGCAAGCTCGGCATCAACTTTGACAGCTTCGTGGCTTGTATGATCCGCCTGGAGACCCTCTTCAAACTATTCAGCCTTCTGGACGAAGACAAGGATGGCATGGTTCAGC
TCTCTCTGGCCGAG------TGG------CTGTGCTGCGTGTTGGTC------
```

```
 ---------------------ATGGCAGCC---------------------------GGTCTTGGCTCCAACCAGAAAGCCGTGAAGTACTTGGGCCAGGACTTCGAGACCCTGAGG---------------
CAACAGTGCTTGGACTCAGGGGTCCTGTTCAAGGACCCGGAGTTCCCAGCATGCCCGTCAGCCTTGGGCTACAAGGATCTTGGACCAGGCTCCCCCCAAACTCAAGGCATTGTCTGGAAGCGGCCCACGGAACTGTGTTCCAGC-
--
CCTCAGTTTATCGTTGGTGGAGCCACGCGCACAGACATTTGTCAGGGTGGTCTCGGTGACTGCTGGCTTCTGGCGGCCATTGCCTCTCTCACCTTGAATGAAGAGCTGCTTTACCGAGTGGTCCCCAGGGACCAGAACTTCCAGA
AGAACTACGCGGGAATCTTTCATTTTCAGTTCTGGCAGTTCGGAGAGTGGGTGGAGGTGGTCGTGGACGACAGGCTGCCCACTAAGGACGGGAAGCTGCTCTTCGTGCACTCGGAAGAAGGCAATGAGTTCTGGAGTGCGCTGCT
GGAGAAAGCCTACGCCAAGCTCAATGGTTCTTATGAGGCTCTCGCTGGAGGGTCCACAGTG------
GAGGGGTTTGAGGATTTCACGGGCGGCATCTCTGAGTTTTATGACCTGAAGAAGCCGCCAGCCAGTTTGTATCACATCGTCTGGAAGGCGCTCTGCAGGGGCTCTCTGCTGGCCTGCTCC-------------------------
-----------------------------------------------------------------------------------------GTCTCCAGCGCAGCCGAA------
ACAGAAGCCATCACCAGCCAGAAGCTGGTTAAGAGTCACGCATACTCTGTCACTGGAGTCGAAGAGGTGGATTTCCGGGGCCGTCCAGAGAGGCTGATCCGGCTCAGGAATCCGTGGGGTGAAGTGGAGTGGACGGGAGCCTGGA
GTGACGATGCGCCAGAGTGGAATTACATAGATCCCAGGCAGAAGGCAAAGCTGGACAGGAAAGCCGAGGATGGAGAGTTTTGGATGTCTTTTGCGGATTTCTTGAGGCAGTTCTCGCGGCTGGAGATCTGCAACCTGTCCCCGGA
CTCCCTGAGTAGTGAGGAGGTGCACAAGTGGAACTTGGTCCTGTTCAACGGCCGCTGGACGCGGGGCTCCACCGCGGGGGGCTGCCAGAACTACCCAGCCACTTACTGGACCAACCCCCAGTTCAAAATCCATTTGGACGAGGTG
GATGACCAC---GAGCCATGCTGTACGGTGCTGCTGGGTCTGATGCAGAAGAATCGCAGGCGACAGAAGAGGATAGGACAGGGCATGCTCAGCATCGGTTACGCTGTCTACAAGGTCCCCAAA---
CTGGAGAGTCACACGGACGTGCACCTGGGCCGGGACTTCTTCATGGGCCACCAGCCCTCGGCCCGCTCCAGCACCTATGTCAACCTGCGGGAGGTCTCCAGCCGCGTCCGGCTCCCCCCGGGGCAGTACCTGGTGGTGCCGTCCA
CCTTCGAGCCCTTCAAGGACGGCGACTTCTGCCTGAGGGTGTTCTCAGAGAAGAAG------------------------------------------------------------------------------
TTCAAGAGCCTGTTTGAGAAGCTTGCAGAG-----------------------------------------------------AAGGAT------------
TCTGAGATCAGTGCCAATGAACTCAAGACGGTTCTGAATGAGGTGTTTTCC---------------------AAA---
CGAACGGACATAAAATTCGACGGATTCGACATCAACACTTGCAGGGAGATGATCAGCCTGATGGATAACAATGGGACAGGCACCTTGGGACCAGTGGAATTCAAGATGCTCTGGTTGAAGATTCAGAAGTATCTGGAGATCTATC
GGGAAACTGATGATAAGCACCTGGGGACCATCGATGCCCATGAGATGAGGACAGCCCTCAAGAAAGCA---GGTTTC---
ACCCTCAACAATCAGGTGGCCCTGCGGTATGCATGCAGCAAACTCGGCATCGACTTCGACGGCTTCGTTGCTTGTATGATCCGCCTGGAGACCCTCTTCAAACTGTTCAGGCTTCTGGACAAGGACCAGAGTGGCACTGTCCAGC
TCTCTCTGGCTGAG------TGG------CTGTGCTGTGTGTTGGTC------

 ---------------------ATGGCAGCC---------------------------GGCCTTGGCTCCAGCCAGAAGGCCGTGAGGTACTTGGGCCAGGACTTCGAGACCTTGAGG---------------
CAGCAGTGCTTGGACTCGGGGCTCCTGTTTAAGGACCCAGAGTTCCCAGCGTGCCCGTCAGCTTTGGGCTACAAGGATCTCGGACCTTGCTCCCCTCAAACTCAAGGCGTTGTCTGGAAGCGGCCCACGGAATTGTGCCCCAGC-
--
CCTCAGTTTATCGTTGGTGGAGCCACGCGCACAGACATCTGTCAGGGTGCCCTGGGTGACTGCTGGCTGCTGGCGGCCATTGCCTCCCTCACCTTGAACCGAGAGCTGCTGTACCGCGTGGTTCCTAGGGACCAGAGCTTCCAGG
AGAACTATGCAGGAATCTTTCATTTTCAGTTCTGGCAGTATGGAGAGTGGGTGGAGGTGGTCGTGGACGACAGGCTCCCCACCAAGGATGGAAAGCTGCTCTTCCTGCATTCCGAAGAAGGCAGTGAATTCTGGAGCGCGCTGCT
GGAGAAGGCCTATGCCAAGCTCAACGGTTCTTACGAGGCTCTCACTGGAGGGTCCACGGTA------
GAGGGGTTTGAGGATTTCACAGGTGGGATCTCTGAGTTTTATAACCTGAAGAAGCCACCGGCCGGTCTGTTCCAGATCATCCGGAAGGCCCTCCGCTCGGGGTCTCTGCTGGCCTGCTCC-------------------------
-----------------------------------------------------------------------------------------GTCTCCAGTGCAGCCGAA------
ACGGAAGCCATCACCAGCCAGAAGCTGGTTAAGGGTCATGCATACTCTGTCACTGGAGCCGAAGAGGTGGATTTCCGGGGCTGTCCAGAGAGGCTGATCAGACTGCGGAACCCGTGGGGTGAAGTGGAGTGGAAGGGGGCCTGGA
GCGACGATGCACCGGAGTGGAATTACATAGCCCCCAGGCAGAAAGAACAGCTGGGCAAGAAAGCCGAGGATGGAGAGTTTTGGATGTCTTTTTCGGATTTCTTGAGGCAGTTCTCTCGGCTGGAGATCTGCAACCTGTCCCTGGA
CTCATTGAGCAGTGAGGAGGTGCACAAGTGGAGCCTGGTGCTGTTCAATGGGCGCTGGACTCGGGGCTCCACGGCTGGAGGCTGCCAGAACTACCCAGCCACTTACTGGACGAATCCCCAGTTCAAAATCCATTTGGACGAAGTG
GACGATCAC---GAACCCTGCTGCACGGTACTGCTGGGGCTGATGCAGAAGAATCGCCGGCGACAGAAGAGGATGGGACAGGGCATGCTCAGCATCGGCTATGCTGTCTACAAGATACCCAAA----------------------
--------------------------------AGCCCAGCTCCGCGG------------------------------------------------------------------------
CCCTCATCCTCAAGGATGTGGGCAGAC------------ACGAGGGTGTTCTCGGAGAAAAAG------------------------------------------------------------------------------
CTGAGGAGCCTGTTTGAGAAGTTTGCCGGG-----------------------------------------------------AAGGAT------------
TCTGAGATCAGGGCCAGTGAACTCAGAACAGCTCTGAATGAAGTGTTTTCC---------------------AAA---
CGAACAGACGTCAAATTTGATGGATTTGACATCAACACTTGCAGAGAGATGATCAGCCTGATGGATAGCAATGGGACAGGCTCCCTGGAACTGGTGGAATTCAAGACGCTCTGGTTGAAGATCCAGAAGTATCTGGAGATCTTTC
GGGAGACCGATCATAACCACTCGGGGACTATCGATGCCCATGAGATGAGGACAGCCCTCAAGAAGGCA---GGTTTC---
ACCCTCAGTGACCAGGTCGCCATGCGGTATGCATGCAGCAAGCTCACCATGGACTTTGACAGCTTCATTGCTTGTATGATCCGCCTGGAGACCCTCTTCAAACTGTTCAGACTTCTGGACAAGGACCAGAATGGCATTGTCCAGC
TCTCTCTGGCTGAG------TGG------CTGTGCTGTGTGTTGGTC------

 ---------------------ATGGCAGCC---------------------------GGCCTTGGCTCTAACCAGAATGCTGTGAAGTATCTGGGCCAGGACTTCGAGACCCTGAGA---------------
AAGCAATGCTTGAACTCAGGGGTCCTATTTAAAGACCCGGAATTCCCGGCATGTCCGTCAGCTTTGGGCTACAGGGATCTTGGACCAGGCTCTGCAGAAACTCAAGGCATCATATGGAAGCGACCCACGGAATTGTGTTCCAAC-
--
CCTCAGTTTATCGTTGGTGGAGCCACACGCACAGACATCCGCCAGGGGGGTCTTGGAGACTGTTGGCTTCTGGCAGCCATTGCCTCCCTCACCTTGAATGAAAAGCTGCTTTATCGAGTGGTTCCTAGAGACCAGAGCTTCCAGA
AGAACTATGCTGGCATTTTTCATTTCCAGTTCTGGCAGTATGGAGAGTGGGTGGAGGTGGTCATTGATGACCGGCTCCCCACCAAGAATGGACAGCTGCTCTTCCTACACTCCGAAGAAGGCAATGAGTTCTGGAGTGCTCTGCT
GGAGAAAGCCTATGCCAAGCTCAATGGTTCGTATGAGGCTCTTGCTGGAGGCTCCACAATT------
```

GAGGGGTTTGAGGATTTCACGGGTGGCATCTCTGAGTTTTATGACTTGAGGAAGCCTCCGGGCAATCTGTACTACACCATCCAGAAGGCCCTGCGCAAAGGCTCTCTGCTGGGCTGCTCC--------------------------
--------------------------------------------------------------------------------------------GTCTCAAACGCAGCCGAA------
GCAGAAGCCACCACCAGGCAAAAGCTGGTTAAGGGCCACGCATACTCTGTGACTGGAGTCGAAGAGGTGGATTTCCGTGGCCTCCCAGAGAAGCTGATCAGGTTGAGGAACCCGTGGGGTGAAGTGGAGTGGACGGGAGCCTGGA
GTGATAGTGCACCTGAGTGGAATTACATAGATCCACAGAAGAAGGGAGAACTGGACAAGAGAGCAGAAGATGGGGAGTTCTGGATGTCCTTTTCGGATTTCTTGAAGCAGTTCTCCCGACTAGAGATATGCAACCTGTCCCCAGA
CTCTCTGAGCAGTGAGGAGATACACAAATGGAACCTGGTACTCTTCAACGGCCGCTGGACACGGGGTTCTACAGCCGGGGGCTGCCAGAACTACCCAGCCACTTACTGGACCAACCCCCAGTTTAAAATCCATTTGGACGAGGTG
GATGAGGAC---GAACCCTGCTGTACAGTGCTGCTGGGTCTGATGCAGAAGAATCGCAGACGTCAGAGGAGGATCGGCCAGGGCATGCTCAGCATAGGCTATGCTGTCTACCAGATTCCCAAG---
CTGGAGAATCACACCGATGAGCACCTGGGCCGGGATTTCTTCCAGGGCCGCCAGCCCTCCACCTGCTCCAGCACCTACATGAACCTTCGAGAGGTGTCCAGCAGGGTCCAACTCCCCCCAGGACAGTACCTGGTGGTGCCATCCA
CCTTTGAGCCCTTCAAGGACGGTGACTTCTGCCTGAGGGTGTTCTCGGAGAAGAAG--------------------------------------------------------------------------------
TTCTGGAGCCTGTCTGAGGAGTTTGCGGAC-----------------------------------------------------AAGGAT------------
TCTGAAATCAGTGCTCACCAGCTCAAGAGGGTCCTGAATGGATTGCTTTCT----------------------AAA---
CGAACAGACATGAAATTTGACGGATTCAACATCAACACTTGCAGAGAAATGATCAGCCTGCTGGATGGTGATGGGACAGGAAGCCTGCGACCGGTGGAGTTCAAGACTCTCTGGCTGAAGATCTGCAAGTATCTGGAGATCTACC
AAGAAATGGACCACAGCCGTGCAGGGACCATTGATGCTCACGAGATGAGGACAGCTCTCAAGAAAGCA---GGTTTC---
ACCCTCAACAACCAGGTGGCCACGAGGTATGCATGCAGCAAGCTTGGTGTTGACTTTGACGGCTTTGTGGCTTGTATGATCCGCCTGGAGATCCTGTTCAAATTGTTCAGACTCTTGGATAAGGACCAGAATGGCATTGTACAGC
TCTCCCTGGCTGAG------TGG------CTGTGCTGTGCACTGGTC------

 ---------------------ATGTCAGCC----------------------------GGTCTTGGCTCCCACCAGAAAGCCGTGAAATACTTGGGCCAAGACTTTGAGAAACTGAGG---------------
CAGCAATGCTTGGATGCGGGGGTCCTATTTAAAGACCCAGAGTTCCCGGCATGCCCGTCAGCCTTGGGCCACAAGGACCTTGGACCACAGTCCCCTCAGACTCAAGGCATCGTCTGGAAGCGGCCCCCGGAATTGTGTGCCAAC-
--
CCCCAGTTTATCGTTGGTGGAGCCACGCGCACGGACATCTGCCAGGGTAGTCTAGGTGACTGCTGGCTGCTGGCGGCCCTTGCCTCCCTGACCTTGAATCAGGAGCTGCTTCACCGAGTAGTGCCCAGGGGCCAGGACTTCCAGC
AGAACTACGCGGGGATCTTTCATTTCCAGTTCTGGCAGTACGGCGAGTGGGTGGAGGTGGTGGTGGACGACAGGCTGCCCAGCAAGGATGGGGAGCTGCTCTTCCTGCACTCGGAGACAGGCAATGAGTTCTGGGGAGCTCTGCT
GGAGAAGGCCTATGCCAAGCTCAATGGTTCCTATGAGGCTCTCACTGGAGGGTCCACGGTC------
GAGGGGTTTGAGGACCTCACGGGCGGCATCTCTGAGTATTACGACCTGAAGAAGCCTCCGGCCCGCCTGTTCCAGATCATCCAGAAGGCCCTCCGCGCGGGCTCTCTGCTGGCCTGCTCC--------------------------
--------------------------------------------------------------------------------------------
GTCTCCAGTGCAGCTGAAGCAGGAGCAGAGGTCACCACCCGCCACAAGCTGGTGAAAAGCCATGCATACTCTGTCACTGGAGTCATAGAGGTGGATTTCCGGGGCCGTCCCGAGAAGCTGATCAGACTCCGGAATCCGTGGGGCG
AAGTGGAGTGGACCGGGGCCTGGAGTGACGATGCACCAGAGTGGAACGACATAGACCCCAGGCAGAAGGAGGAGCTGGACCGGAGAGCCGAGGATGGGGAGTTCTGGATGTCATTTTTGGATTTCCTGGGCCAGTTCTCTCGGCT
GGAGATCTGCAACTTGTCCCCAGACTCCCTGAGCAGTGAGGAGCTGCACAAATGGAACCTGGTTCTGTTCAACGGCCGCTGGACACGGGGCTCCAGTGCTGGGGGCTGTGCGAACTTTCCAGCCACTTACTGGACCAATCCCCAG
TTCAAAATCCATCTGGACGAGGTGGACGACAGC---
AAACCCCACTGCACGGTTCTGCTGGGTCTGATGCAGAAGAATTGCAGGCGACAGAAGAGTACAGGACAGGGCATGCTCAGCATTGGCTACGCTGTCTACAAGGTACCTGTG---
CTGGAGAGCCCTGCGGACACACCCCTGGGCCAGAATTTCTTCTTGGGCCGCCAGCCCTTTGCACGCTCCAGCGCCCATGCCAACCTGCGGGAGGTGTCCAGCCGGCTGCTGCTGCCCCCGGGGGAGTACCTGCTGGTGCCATCTA
CCTTCGAGCCCTTCCAGGATGGGGACTTCTGCTTGAGGGTGTTCTCACAGAAGAAG--------------------------------------------------------------------------------
TTTGGGAGTCTGCTGGGGACACTTGCAGGG-----------------------------------------------------AAGGAT------------
TCTGAGATCGGTGCCAATGAACTCAGGACGATTCTGAATGAAGTGTTTTCC----------------------AAA---
GAAACAGACATAAAATTCTGTGGCTTCGACATCGACACTTGCAGGGGGATGGTCAGCTTGATGGACAACAGCGGCACGGGCCTCCTAGGGCTGGCGGAACTCAGGACACTGTGGCTGAAGCTTCGCAAGTACCTGGAGATCTACC
GGGAGACCAATGACAACCACTCGGGGAGCATTGACACCCATGAGATGAGGACGGCCCTTAGGAATGCA---GGTTTC---
ATGCTCAGCACCCAGGTGGCCCTGCGGTATGCGGACAGCAGACTTAGCATCGACTTCGATGACTTCGTGGCCTGCGTGGCCCGCCTGGAGTCCCTCTTCAAGCTGTTTAGGCTCCTGGACAAGGACCAACGTGGCACCGTCCGGC
TCTCCCTGGCTGAG------TGG------CTGTGCTGCGTGTTGGTC------

 ---------------------ATGGCAGCT----------------------------GGGCTTGGCTCCATTTGGAACGCCGTGAAGTACCTGAACCAGGACTTTGAAGCCCTGAGG---------------
AAGCAATGCCTGAAGTCAGGAACTCTGTTTAAGGATGTGGAATTTCCGGCATGCCCTTCGGCTCTAGGGTTTAAGGATCTGGGACCCTACTCACCGCACACGCGAGGCATTGTCTGGAAGCGGCCCACGGAGTTATGCCCCAAC-
--
CCCCAGTTTATTGTTGGTGGAGCGACCCGCACAGACATCCGCCAGGGGGACCTTGGTGACTGCTGGCTCTTGGCAGCCATCGCCTCCCTCACCCTGAATGAAGAGCTGCTATATCGGGTGGTGCCCAGGAACCAGAGCTTCCAGA
AGGACTATGCAGGAATCTTCCACTTCCAGTTCTGGCAGTATGGAGAATGGGTGGATGTTGTCATTGATGACAGACTCCCCACCAGAAATGGAAATTTGCTTTTCCTGCACTCAGAAGAAGGCAGCGAGTTCTGGAGTGCCCTGCT
GGAGAAGGCTTATGCCAAGCTCAATGGATCATATGAAGCTCTCACAGGAGGGTCCACAGTG------
GAGGGGTTTGAGGATTTCACAGGTGGCATCTCAGAGTTTTATGAGCTGAAGAAGGCTCCCTCCAATCTGTATCAGATCATCCAGAAGGCCTTGCGCTCTGGATCCCTGCTCGGCTGCTCC--------------------------
--------------------------------------------------------------------------------------------GTCAGCAGTGCAGCAGAA------
ACAGAAGCAGTCACCAGCCTGAAGCTGGTGAAGGGGCATGCTTACTCCGTCACTGGGGCTGAAGAGGTCCGGCACCATGGCCGCCCAGAGAAGCTGATCAGGCTCCGCAATCCGTGGGGTGAAGTGGAGTGGACCGGAGCCTGGA
GTGACAAGGCTCCAGAATGGAATTTCATTGACCCCAAGCAGAAGGAGGAGCTGGACAAGCAGATGGAAGATGGAGAATTTTGGATGTCTTTTACAGATTTTCAGCGACAATTCTCTCGGCTGGAGATCTGCAACTTGTCCCCCGA
CTCCCTGTCCTGTGAGGAGGTGCACAAGTGGGGCTTGACCTTGTTCAATGGCCGCTGGATCCGGGGCTCCACGGCTGGAGGCTGCCAGAACTACCCAGCCACGTATTGGACCAATCCCCAGTTCAAAATCTGTCTGGATGAGGCA
GATGACGAC---GACCACTGCTGCACTGTGCTGCTGGGCCTGATGCAGAAAAACCGCAGAAGGCAGAAGAGGAAGGGCCAGGGGTTGCTCAGGATCGGCTACTCGATCTACCAG------------

```
CTGGAGAACCACTCCGACAGGCACCTGGGGCAGGATTTCTTCCTGGGCCGCCGCCCCTCAGCAAGCTCGGACTCCTATGTCAATCTGCGGGAAGTCTCCGGCCGTGTGCAGCTGCCCCAGGGAGAGTACCTGGTGGTACCCTCCA
CTTTGGAGCCATTTAAGGATGGAGAGTTCTGCCTGAGAGTGTTCTCAGAGAAGAAA------------------------------------------------------------------------------
TTCAAGAGCCTGTTTGAGAAGCTGGCTGGG---------------------------------------------------GAGGAC------------
TCCAGGATCCAGGTCCATGAGCTCCGGGATATTTTGAATGAGACTTTTTCC---------------------AAA---
CGAGCTGACTTAAAATTTGATGGATTCAGTCTCAACACCTGTAGGGAAATGATCAGCCTTTTGGATAGTGATGGTTTGGGTGATTTGGGACTCCTTGAATTCAAGACACTCTGGCTCAAGATTTTGAAGTACCAGGAAATCTTTT
TGGAAGTGGACACCAACCACTCAGGGACCATCCGCGCCCATGAGATGCGGACGGCTCTGAAGGAAGCA---GGT-----------------------------------------------------------------------
----------------------------------------------CTTCAGGATGAGGAT------------------------------------------------------------------------

 ---------------------ATGGCGGCT---------------------------GGCCTGGGTTCCAGCCAGAATGCTGTGAAATATTTGGACCAGGACTTTGAGACCTTAAGG---------------
AAACAGTGCTTGGAGTCTGGGACTCTATTTAAGGACCCAGAATTCCCAGCTTGTCCCTCAGCTTTGGGATATAAGGATCTGGGACCATACTCTCCTCAAACCCAAGGGATTATCTGGAAAAGACCTACGGAGTTGTGTCCTGAC-
--
CCTCAGTTTATTATTGGTGGAGCTACTCGAACAGATGTTCGTCAGGGTGCCCTTGGTGATTGCTGGCTCCTGGCAGCCATTGCTTCCCTGACCTTGAATGAAGAGCTCCTTTACCGGGTGGTCCCCAAAGATCAGAGCTTCCAGA
AGAACTATGCAGGAATCTTTCATTTTCAGTTCTGGCAGTATGGAGAATGGGTGGATGTTGTCATCGATGACAGGCTCCCCACCAGAAATGGGAATTTGCTATTCCTTCACTCAGAAGAAGGCAGAGAGTTCTGGAGTGCCCTGAT
GGAGAAAGCCTATGCCAAGCTTAATGGGTCTTATGAAGCTCTCACTGGAGGGTCCACAGTG------
GAGGGATTTGAAGATTTCACTGGAGGAATCTCAGAGTTTTATGATTTGAAGAAGCCTCCATCCAATCTGTATCAAATCATCAGGAAGGCTTTGCGTTCTGGATCACTGCTTGGCTGCTCC-------------------------
-----------------------------------------------------------------------------------------ATCACCAATGCATCAGAA------
TCACAGGAAATCACCAGCCTGAAGCTGGTGAAGGGGCATGCTTATTCTGTCACTGGAGTTGAAGAGGTTAATTATCGTGGCTGTCTTGAAAAGCTAATTCGTCTAAGGAATCCGTGGGGTGGGACAGAGTGGACAGGAGCATGGA
GTGACAGTGCCCCAGAATGGAATTACATAGATCCCAGGCAGAAGAAAGAATTGGACAAGAAAGCTGAAGATGGAGAGTTTTGGATGTCATTTTCAGATTTCCTGAGGCAATTCTCTAGGCTGGAGATCTGTAATTTGTCTCCTGA
CTCTTTGACCAGTGAGGAGATCCACAAATGGAACATGACACTGTTCAACGGCCGCTGGATCAGGGGCTCCACAGCTGGGGGCTGCCAGAATTACCCAGCTACATACTGGACCAACCCCCAGTTCAAGATCCGATTGGATGAAGCA
GATGACCAC---
GAGCCCTGCTGCACAGTGCTGCTGGGTCTGATGCAGAAAAACAGCAGGAGAAAGAAGAGAATGGGACAAGGGATGCTCAGCATTGGCTATTCCCTTTACCAGGTACTTCACATCCTGGAGAACCACACTGAAGTACATCTGAGCC
AGGACTTCTTCTTGGGACATCAGCCTGCTGCCAAAACCCCAGCCTACATTAATCGTCGAGAAGTTTCTACCAGGATCCACCTCCCTGTGGGAGAGTACCTGGTGGTGCCATCAACCTTTGAGCCATTCAAGGATGGAGAGTTCTT
TCTGAGGATATTCTCAGAGAAAGAA------------------------------------------------------------------------------AGAAAAATTCAGATCTATAAAATGACAACA------------
---------------------------------------GACAATAGATCAAGGGATTCTGAAATCAGTGCCAATGGACTCCAAACCATTCTGAATGATTTCTTTTCT---------------------AAA---
CGAACAGACGTGAAATCTGATGGATTCAACATCAATACCTGCAGAGAAATGATTGGCCTTTTGGATAATAATGGAACAGGCACTTTGGGACTTGCAGAACTCAAGATATTATGGCTGAAGATTCAGAAATATTTGGAGATCTACC
AGGAAGTGGATATTAACCACTCAGGGACCATCAATGCCCATGAGATGAGAATGGCTTTCAAGAAAGCA---GGTTTC---
ACCTTGAACAACCAGGTAGCTGTGCGCTATGGGGGCAGCTCTCTCAACATTCACTTTGACAGCTTCATTGCCTGCATGATGCGTCTCGAGAGTCTATTCAAAATATTCAGTCTCTTGGACAAGGACCACAGTGGCACTGTCCACC
TCTCTCTGGCAGAG------TGG------CTGTGTTGTGTGCTAGTC------

 ---------------------ATGGCAGCT---------------------------GGTCTGGGTTCCAGCCTGAATGCTGTAAAATACCTGGGCCAGGACTATGAGACCTTAAGG---------------
AAACAGTGCTTGGAGACAGGGACTCTATTTAAGGACCCAGAATTTCCAGCTTGTCCCTCAGCTTTGGGATACAAGGATCTGGGACCATATTCTCCTCAAACCCAAGGAATTGTGTGGAAACGGCCCACGGAATTGTGTCCTGAC-
--
CCTCAGTTTATTGTTGGTGGAGCTACTCGAACAGATGTTCGTCAGGGTGCCCTTGGTGACTGCTGGCTCCTGGCAGCCATTGCTTCCCTGACATTGAATGAAGGGCTCCTTTACAGAGTAGTCCCTAAAGGTCAGAGCTTTCAGA
AGAACTACGCAGGAATCTTTCATTTTCAGTTCTGGCAGTATGGAGAATGGGTGGATGTTGTCATCGATGACAGGTTACCCACAAGAAATGGGAATTTGCTATTCCTTCACTCAGAAGAAGGCAGAGAGTTCTGGAGTGCCCTGCT
GGAAAAGGCCTATGCCAAGCTCAATGGGTCCTATGAAGCTCTCACAGGAGGGTCTACCGTG------
GAGGGATTTGAAGATTTCACCGGAGGAATCTCAGAGTTTTATGATTTGAAAAAGGCCCCAACCAATCTGTATCAAATCATAAGGAAGGCTTTACGCTCTGGATCACTGCTTGGATGCTCT-------------------------
-----------------------------------------------------------------------------------------GTCACCAATGCATTGGAA------
TTGCAGGAAGTCACCAGCCTGAAGCTGGTTAAGGGGCACGCTTACTCTGTCACTGGAGTTGAAGAGGTTAATTTCCATGGTCGCCTTGAAAAACTAATCCGCCTAAGGAATCCATGGGGTGGGACGGAATGGAAAGGAGCATGGA
GTGACAGTGCCCCTGAATGGAATTACATAGACCCAAGGCAGAAGGAAGAACTAGACAAGAAAGCTGAAGATGGAGAATTTTGGATGTCATTTTCAGATTTTCAGAGGCAATTTTCTCGCCTGGAGATCTGTAACTTGTCTCCTGA
CTCCTTGACCAGTGAGGAGATCCACAAATGGAACACAGCATTGTTCAATGGCCGCTGGATCCGGGGCTCCACAGCTGGAGGCTGCCAGAACTACCCAGCTACATACTGGATCAATCCCCAGTTTAAGATCCGATTGGATGAGGCA
GATGATCAC---GAGCCCTGCTGTACAGTTCTGGTGGGTCTGATGCAGAAAAATCGAAGAAAGAAGAAGAGGATTGGACAGGGGATGCTCAGCATTGGCTATTCACTTTACCAGATCCCCAAA---
CTGGAGAACCACACAGAAGTGCATCTGAGCCAGGACTTCTTCTTGGGACATAAGCCTGCTGCTCAAACCCCAGCATACTTTAACCTTCGAGAAGTTTCTAATCGGTTCCACCTTCCTCTGGGAGAGTACCTGGTGGTGCCCTCAA
CTTTTGAGCCATTCAAGGATGGAGAGTTTTGCCTGAGGATATTCTCAGAGAAGGAAGATACGTACAAA------------------------------------------------------------------
TTCAAGATTCTTTTTGAGAAGCATTCTGGG---------------------------------------------------GAGAAT------------
TCTGACATCAGTGCCAAAGCGCTCCAAACTATTCTGAATGGCTTCTTTTTT---------------------AAA---
GGAACAGGCGTGAAATTTGATGGATTCAACATCAATACCTGCAGAGAAATGATTGGCCTTTTGGACAATAATGGGACAGGTACTTTGGGACTTGTAGAACTCAAGATATTATGGCTGAAGATTCGGAAATATTTGGAGATCTACC
AGGAAGTGGATGCTAATCACTCAGGGACCATCAATACCCATGAGATGAGGACAGCCTTCAAGAAAACA---
GGATATAAAAACCTCAACAACCAGGTCGCTGTGCACTATGGGGACAGCACTCTCAGCATTCCCTTCGACAGGTTCATTTCCTGCATGATACGTCTGGAGAGTCTCTTCAAAATATTCCATCTTTTGGACAAGGACCAGAATGGCA
TTGTCCAGCTCTCACTGGCAGAG------GTA------AATGGGATTAGGTTTGTGGTACAG
```

 ------------------------------------------------------------------------------------------------CAAGACTTTGATAAGCTGAGG---------------
GCACAGTGTTTGGCATCTGGGACTCTCTTCAGTGATGAGGAATTCCCAGCATGCCCATCTTCCCTGGGTTACAATGAACTGGGACCCGGCTCATCCAAAACCAATGGAATTGTATGGAAGAGACCCAAGGAGATTTGCCCCAACG
GCCCCCAGTTTATTGTAGATGGAGCAACGCGTGGAGACATCCGTCAGGGGACACTTGGGGACTGCTGGCTCCTGGCGGCCATCGCATCTCTTACACTGGATCCAGACCTTGTAGCCCAGGTGGTTCCAGAGAACCAAAGTTTCCA
GAAGGGCTACGCTGGGATCTTCCACTTCCAGTTCTGGCAGTACGGAGAGTGGGTGGACGTGGTGGTGGATGACCGGCTGCCTATGAAGGATGGGAACCTGGTGTTTGTACACTCGGCTGAAGGGGACGAATTCTGGAGCGCTCTG
CTAGAGAAGGCCTACGCAAAGTTGAACGGCTCCTACGAGGCTCTGACTGGCGGTTTGACCATA------
GAGGGATTTGAAGACTTTACCGGAGGTATCGCCGAGGTGTACGAACTGAACGCGGCTCCGTCTGATTTATTCCAGATCATCCAAAAAGCCCTTAGGGCTGAATCACTGCTTGGCTGCTCT-------------------------
-----------------------------------------------------------------------------------------ATTTCTAATTCAAATGAC------
ACTGAAGCCGAGACAAGCAGAAAGCTGGTTAAGGGGCACGCGTATTCAGTGACGGGCGCCGAGGAGGTGTTATACCGAGGGCGCCGGGAGAAGCTGATCCGAGTGAGAAATCCCTGGGGAGAAGTAGAATGGACCGGACCTTGGA
GTGATTATGCTCCAGAGTGGAATTATATTGATCCTAAGGTGAAGGCAGCTCTGGATAAACAGTCTGACGATGGAGAATTTTGGATGGCGTTTTCTGACTTTCTCCGAGAGTATTCCCGCCTGGAGATCTGTAACCTGTCCCCCGA
CACACTCACCAGCACCGAGCAGCATAAATGGAACATAACCCTGTACAATGGGAGCTGGGCACGGGGGTCTACTGCCGGCGGCTGCCCCAAATACCCAGAAACATTCTGGACAAACCCACAGTTTCGCATTAAACTGGATACGCCG
GATTGGGGC---GAGCCCTGCTGTACAGTCATTGTGGGGCTGATGCAGAAGAACCGCAGACGGAACAGAAAGATGGGGGAGGATTTGCTTAGCATCGGCTTCTCCATATTTAGGATCCCTGAT---
CTTCGAGGCCACACTGATAGCAACTTCAGCAGGGAATTCTTCCAAAGGTCTCGAACAGTGGCTCGATCTGACGCCTATATAAACATACGGGAGGTCTCCCAGCGCTTCTATCTGCCAGTCGGGGATTATCTCATCGTCCCAACCA
CCTTTGAGCCTTTCCAAAATGGCGACTTCTGCCTTCGAATCTTCTCAGAAAAGGAG------------
TTTATTTTCAACTCCACTGAAAAAATCAAGGTAATATACTACATTTATATATATCTACACTATACACTCAAGGAGATGTTTTATGAGTTTGCCGGGAAACCGAGTAATCCCGACACTTGGGGTTACGGCAACAAATTAGCAAATC
CCAAGAAA------------GAAGAACTTGATGCTACAGATCTCCAAAGCATTCTCAATGTGCTCCTATCAATCCCAGTAAAGAATCTCCCCAAA---
CGGCCGGACTTGAGATCGGAGAAATTTACCCTTAGCACTTGCAGGGAGATGATCAGCCTCCAGGATATGGATGGGACAGAAACACTGAACCTCCGGGAGTTCCGAGTTCTCTGGATGAAGATACAGAAATACATGACAATCTATT
TAAGGGCGGACTCGGATCGTTCTGGAACCATAGACGCTCTTGAGCTGAGAACAGCTTTACCACAAGCA---GGTTTT---
ACTCTGAATAATAAGATCGTGAAGCGATACGCCTCTGAGGACCTGGCTATTAACTTAGATGCCTTTATTGCCTGTATGATGCGCCTGGAGACCCTGTTCAAAATGTTCGAGATATTGGACAAGAGCAAGAGAGGGGTCGTTGAGC
TGGATTTACAAGAG------TGG------CTCAGTGCAACAGTGACCCCC---

 ---------------------ATGTCTAAC---------------------------GGTCTTGGTACCAATAAGAACCCAGTGCAATATCTAAACCAAGACTTTGAGAAGCTGAGG---------------
GCACAATGCTTGGCATCTGGTACCCTGTTTAAAGATGAAGAATTCCCAGCATGCCCTTCTGCACTGGGTTTCAACGAACTGGGACCCGGCTCCCCAAAAACACAAGGAGTGATATGGAAGAGACCTTCGGAGATTTGCCCCAAC-
--
CCCCAGTTCATTGTGGATGGAGCAACACGCGGAGACATCCGTCAAGGGGCTCTCGGGGATTGCTGGCTCCTGGCGGCCATCGCTTCTCTTACACTGGACGCGGATCTTGTAGCCCAGGTGGTTCCAGAGAATCAAAGTTTCCAGA
AGGACTACGCTGGGATCTTCCACTTCCGGTTCTGGCAGTACGGAGAGTGGGTGGATGTGGTGGTGGATGACCGGCTGCCTACGAAGAATGGGAACCTGGTGTTTGTACACTCGGCTGAAGGGGACGAGTTCTGGAGCGCTCTGCT
AGAGAAGGCCTACGCAAAGTTGAATGGCTCCTACGAGGCTCTGTCTGGCGGTTCCACCATA------
GAAGGATTTGAAGACTTTACCGGAGGTATCGCCGAGGTGTATGAACTGAGGAAGGCTCCGTCTGATTTATTCCAGATCATCCAGAAAGCCCTTAGGGCTGAATCACTGCTTGGCTGCTCT-------------------------
-----------------------------------------------------------------------------------------ATTACGAATGCCTATGAC------
ACTGAAGCCATCACAAGCCGAAAGCTGGTTAAGGGGCACGCGTATTCAGTAACTGGCGCCGAGGAGGTGTTATACCGAGGTCGCCAGGAGAAGCTGATCCGAGTGAGAAATCCCTGGGGAGAAGTAGAATGGACTGGACCTTGGA
GTGATACGGCTCCAGAGTGGAATTATATCGATCCAAAAGTGAAAGCGGCTCTGGATAAACAGTCTGACGATGGAGAATTTTGGATGGCGTTTTCAGACTTTCTCCGAGAGTATTCCCGCCTGGAGATCTGTAACCTGTCCCCCGA
CACACTCACCAACACCGAGCAGCATAAATGGAACATAACCCTGTACAATGGGAGCTGGGCACGGGGGTCTACTGCCGGCGGCTGCCAAAACTATCCAGCAACATTTTGGACCAACCCGCAGTTTCGCATTAAACTGGATGAACCC
GATCATGAA---GAGCCGTGCTGCACCGTTATTGTGGGATTGATGCAGAAGAACCGTAGAAGAAAGAAGAAGATGGGAGAAGACTTGCTGAGCATTGGCTACTCACTCTTTAAGGTTAGTATT---
TTATCCTCAATAGTTCTTCTGTATATA---------TACATCCAAAAGACTCCAACGGCCGCCCGTTCCGACACCTATATAAACGTACGGGAGGTGTCCAACCGCTTC---
CTTCCAGTCGGGGATTATCTGATTGTGCCGTCCACCTTTGAGCCTTTTAAAAATGGCGACTTCTGTCTTCGTGTCTTCTCAGAAAAGGAG-------------------------------------------------------
-----------------------TTCAAGAATATTTTTGACAAGCTTGCAGGG---------------------------------------------------GATAAA------------
GAAGAAATTGATGCGAAAGAACTTCAAACCATTCTCAATAAACTCATTTCA---------------------AAG---
AGGCCGGACTTGAGATCCAATGGATTTACTGTCAACACTTGCAGAGAGATGATCAGCTTACAAGACATGGATGGAACAGCAACGCTGAGCCTTCTGGAGTTCCGTATTCTGTGGCTGAAGATACAGAAATACTTGGCAATCTATT
TAAAGGCAGACTCTGACCGTTCTGGGACCATGGACGCCCACGAGCTGAGAGCAGCTTTACAAGAAGCA---GGTTTT---
ACTCTGAATAATAAGATCGTGCTGCGATACGCCTCTGATGAGCTGACGATTAACTTCGATGGCTTTATTGCCTGTATGATGCGCCTGGAGACCCTGTTCAAAATGTTCCAGATGTTGGAAAAGAGTAAGAAAGGGGTCGTTGAGC
TGAGTTTACAAGAGGTAACCTGGGCTAACCTGTGTGTGTATATA---------

 ---------------------ATGTCGAGA---------------------------GGTGGTGGAACGAAGAGGAACCCAGAAAAATATTTGGATCAAGAATTTGAGAAGCTGAGG---------------
GCACAATGCTTGGCATCTGGTGCTCTGTATAAAGATGAAGAATTCCCAGCATGCCCATCTGCACTGGGTTACAATGAACTAAGACCCGGCTCATACAAAACCAGTGGGGTCATATGGAAGAGACCTACGGAAATTTGTCCCAAC-
--
CCCCAGTTCATTGTGGATGGAGCAACACGAGGAGACATCCGTCAAGGGGCCCTCGGGGATTGCTGGCTTCTGGCTGCCATCGCATCTCTTACACTGGAGCCAGATCTTGTAGCTCAAGTGGTTCCTGAGAATCAAAGTTTCCAGA
AAAACTACGCTGGAATCTTCCACTTCCGGTTCTGGCAGTATGGAGAGTGGGTGGATGTGGTGGTGGATGACCGGCTGCCGACAAAGAATGGGAAACTGGTATTTGTCCACTCAGCAGAAGGGGATGAGTTCTGGAGTGCTCTGCT
TGAAAAGGCCTACGCAAAGTTGAACGGCTCCTACGAGGCTCTGACTGGCGGTTCTACCATA------
GAGGGATTTGAAGACTTTACCGGAGGTATCGCTGAGGTGTATGAACTGAAGAAGGCTCCACCTAATTTATTCCAGATCATCCAGAAAGCCCTTAAGGCTGAATCACTGCTTGGCTGCTCT-------------------------

```
-----------------------------------------------------------------------------------------ATTACAAACGCCTATGAT------
ACTGAAGCCATCACAAGCAGAAAGCTGGTTAAAGGGCACGCCTATTCTGTCACTGGTGCTGAGGAGGTGTTGTACAGAGGTCGCCAGGAGAAGTTGATCCGAGTGAGAAATCCCTGGGGTGAGGTAGAATGGACTGGACCTTGGA
GTGATGAGGCTCCAGAATGGAATTATGTTGATCCAAAAGTAAAAGCTGTTCTGGATAAAAAATCTGAGGATGGAGAATTTTGGATGGCGTTTTCAGACTTTCTCAGAGAGTATTCCCGTCTGGAGATCTGTAACCTGTCTCCTGA
CACCCTCACCAGCAACCACCAACATAAATGGAACATAACATTGTACACCGGGAGCTGGGCACGGGGGTCTACTGCCGGAGGCTGCCAAAATTACCCAGCAACATTTTGGACCAACCCGCAATTTCGCATTAAACTGGATGAGCCC
GATGATGAT---GAGCCGTGCTGTACCGTCATTGTGGGACTGATGCAGAAAAACCGCAGAAGAAAGAAGAAGATGGGGGAGGACTTGCTCAGCATTGGCTACTCACTCTTTAAGATCCCTGAT---
CTTCAGGACCATACAGATGCCCACCTCGGCAGGGACTTCTTGCAAAAGACTCCAACGGCCGCCCGATCTGACACCTATATCAACGTACGGGAGGTGTCCAACCGCTTCCACCTACCGGTCGGGGATTATCTGATTGTGCCATCCA
CCTTTGAGCCCTTTAAAAATGGCGACTTCTGCCTTCGTGTCTTCTCAGAAAAGGAG------------------------------------------------------------------------------
TTCAAGAATATTTTTGACAAGCTGGCGGGA---------------------------------------------------GATAAA------------
GAAGAAGTTGATGCAAGAGAACTTCAAACCATTCTAAATAAACTCATTTCA---------------------AAG---
AGGCCGGACTTGAGATCCAATGGATTTACTCTTAACACATGCAGAGAGATGATCAGCTTACAAGATATGGATGGAACCGCAACACTGAGCCTTCTGGAGTTCCGTATTCTGTGGATGAAGATACAGAAATATTTGGCAATCTATT
TAAAGGCAGACTCGGATCGTTCTGGAATCATGGACTCTCATGAGCTGAGAACAGCTTTGCAAGAAGCA---GGTTTT---
ACTCTGAATAATAAGATCGTGCAGCGCTACGCATCCAATGACCTGGCTCTTAACTTTGATGGCTTTATCGCTTGTATGATGCGCCTGGAGACCTTGTTCAAAATGTTTCAGATGTTGGACAAGAGTAAGAGAGGGGTCGTTGAGC
TGAGTTTACAAGAG------TGG------CTCTGCGCAACATTGGTC------

 ---------------------ATGACATCC---------------------------GGCGTCGGCACCATTGAGAACGCCGTGAAGTTTTGCCAGCAGGATTTTGAGACTCTTCGG---------------
CAGGAATGTCTGAGCAGCGGCCGCCTCTTTGAGGATGGCTGCTTTCCAGCTCAAAGCAAGTCTCTGGGCTACAAAGAGCTGGGACCGTACTCATCCAAGACCAAGGGGATTGTCTGGAAGAGACCTACGGAGCTCTGTTCCAAC-
--
CCAAAGTTCATCGATGATGGAGCCACCAGGACAGATATCTGTCAAGGAAGCCTTGGCGACTGCTGGCTGTTGGCCGCCATAGCCTCTCTGACTCTAGATCAGCAGATCCTGGCTCAAGTGGTTCCTCCCGGACAGAGTTTTTCTG
AAGACTATGCTGGAATTTTTCACTTCCAGTTTTGGCAGTATGGCGAATGGGTCGACGTGGTCATTGATGACCGTTTACCCACCAGAGATGGAAAGCTGCTGTTTGTCCACTCAGAGGAAGGCTCAGAGTTCTGGAGTGCTCTGCT
GGAAAAGGCTTATGCTAAAGTGAACGGCTGTTATGAGGCCTTGTCCGGAGGAAACACCATA------
GAAGGCTTTGAGGACTTCACTGGGGGCATCGGAGAGATCTTTTCCCTGAACAAGGCTCCGCCTCAGCTCTTTAAGATCATTCAGAAGGCTCTATGTCTGGGTTCACTGATTGGGTGCTCC-------------------------
-----------------------------------------------------------------------------------------GTCACCAGTTCCTACGAG------
ACGGAAGCTGTCACGACTCTAAAGCTCGTGAAAGGTCATGCGTACAGCGTTACCGCAGCAGAAGAGGTAAACGTTCGTGGGCAGCAGGTTCAGCTGGTCCGCATCAGGAACCCATGGGGTCAGGTGGAGTGGACCGGGGCTTGGA
GTGACGGATCCAGGGAATGGAACCACATCAGCCAAGAGGAGAAGTCGAAGCTTAACAAAGTGGCTGAAGATGGGGAGTTCTGGATGTCTTACTCAGACTTCATCAGAAATTTCTCCGACCTGGAAATCTGTAACCTGACCCCGGA
CTTGCTCACGGACGACACAAAAAGCTGCTGGAACAACTACCAGTTTGAGGGGACATGGAGGGTGGGCTCCACCGCCGGCGGCTGCCTCAATCACTCAGCCACATTTCCATCCAACCCTCAGTTCTTGGTGCATCTAGAGGACGTG
GATGACGATCCT---------TGTACTTTTCTGGTGGGACTCATGCAAAAGGATGGACGTCGGAAGAGGGTGCTTCAACAAGATCTGGAGACCATTGGCTTTGCCATCTATAAGGTCCCAGAT---
TACAAAGGCCGCAGCAACGTTCGCCTTGGCCCTGATGTCCTGCTGCGGCAGCGGCCCGTGGCCATGAGCAGCACCTTCATCAACAGACGGGAAGTGTGTGGTCGCTTCACACTCCCTCCTGGAGATTACGCCATTATCCCCTCAA
CCTTCCAGCCTCACAAGAACGGCTCTTTTGTTCTCAGGGTGTTCTCTGAGAAGAAG------------------------------------------------------------------------------
TTCAAGGAAATGTTCAAAAGCGCTGCTGGC---------------------------------------------------GGTCAC------------
ATGGAGGTTTCCGCCATTGAGCTGCAGAGAATTTTGAACAAAATCTTTTCT---------------------ATC---
CAGACGGATGTCAAGACAGATGGATTCAGCCTAGAGACGTGTCGCCTCATCGTCAGCCTCATGGATAAAGATGAAAGTGGCAAGTTGGGCCCGATGGAATTCCGCCTTCTGTTGACCAAAATCCCGAAATATTTGGAGATCTTCA
AGAGCCTTGACACTGACAACTCTGGCACCATGAGTTCCCATGAAATGAGAAATGCTGCATTCAAAGCAACAGGTTTC---
CAAATCAACGGCTCTGTTGTCAACCGCTACGCCGACGCGCAGTACGCCATCGACTTTGACAGCTTCGTGAGCTGCCTGGTCAAACTGGAAATGCTCTTCAAAATGTTCAAAGCGCTGGACAGAGGTGGCTCAGGAAAGATTGAGC
TGAACATGCAACAA------TGG------TTGTGCCTGGCAATTTAC------

 ---------------------ATGACCTCC---------------------------GGCATCGGCACCAACCAGAAGGCAGTGAAGTTT---------GCGNATGAGGCCCTGCGG---------------
CAGCAGTGTCTGAGCAGGGGACGCCTGTTTGAGGATGGCAGCTTTGGTGCCGAGAGCAAGTCACTAGGCTACAATGAGCTGGGGCCGTACTCATCCAAGACGAGAGGCATTGTTTGGAAGAGGCCAACGGAGCTGTGTTCAAAC-
--
CCAAAGTTCATTGACGATGGCGCCACAAGGACGGATATTTGTCAGGGAGCATTAGGGGATTGCTGGCTTCTGGCTGCCATTGCTTCTCTAACTCTGGACCAGCGAATCCTAGGACGGGTGGTGCCCACTGAACAGAGCTTCACTG
AGGATTATGCAGGGATATTTCACTTTCAGTTTTGGCAGTATGGTGAGTGGGTGGATGTCGTAATAGATGATCGTTTACCCACCAGAGATGGGAAGCTTCTGTTTGTTCACTCAGCAGAGGGCTCAGAGTTCTGGAGCGCTCTCCT
GGAGAAAGCTTACGCAAAGGTGAATGGCTGCTACGAAGCTCTGTCTGGAGGAAACACCATC------
GAGGGCTTTGAGGATTTCACAGGAGGGATTGCTGAGATTTACACCTTGAGCAAAGCTCCACCAAAACTCTTCCAAATCATGCGGAAAGCCCTGAGTCTGGGTTCACTCCTGGGCTGCTCT-------------------------
-----------------------------------------------------------------------------------------ATTACCAGCGCCAGTGAG------
ACAGAGGCAGTAACCGCTCTGAAGCTTGTGAAAGGACATGCATACTCTGTTACTGGTGCAGAAGAGGTCAGCTACCGAGGCAGGGAAGTTCAACTGGTCCGTGTCAGAAACCCATGGGGAGAAGTGGAATGGACAGGTCCTTGGA
GTGACGGATCCTATGAGTGGAAGTATGTCAGTGAGGCTGACAAATCAAAGTTGGACCATGTGGCAGAAGATGGAGAGTTCTGGATGTCCTACTCAGACTTCATCACGCACTTCTCCAAGCTGGAGATCTGCAACTTGACCCCAGA
TACGCTGGAAAGCGATGAGGTTGGCCACTGGAACAACTACCAGTTCGAAGGGATGTGGAGGGTGGGATCCACCGCTGGCGGCTGCAGAAACAATGAAGCCACGTTCTGCTCCAACCCTCAGTTCGTCATGCGTCTGGAGGATGTG
GATGACCACCCT---------TGCACCTTCCTGGTGGGGTTGATGCAAAAGGATGGACGGCAGAAGAGGAGGCTTAATTGCGAACTGGAGACCATCGGCTTCGCCATTTACAAGGTTCCAGAT---
TACAAAGGCCGCGGCAACGTTCGCCTCGGCCCAGATGTGTTGCTGCGTCAGAAAGCCGTGGCGATGAGCAGCACCTTCATCAACACCCGCGAAGTGTGCGAACGCTTCAGACTTCCCCCTGGAGAATACGCCATCATTCCCTCAA
CCTTTCACCCTCACAAGAATGGCAAATTTGTTCTCAGGGTATTCTCAGAGAAAGAG------------------------------------------------------------------------------
```

TTCAAGAAAATCTTTAAGGAAATTGCTGGC---------------------------------------------------ACTGAC------------
ATGGAGGTATCTGCCTTTGAGTTAGTTGAAATTCTGAACAACATTGTCTCT---------------------AAC---
AGCTTTGACATCAAGACAGACGGATTCAGCCTCGAGACGGCGCGTCTCATAGTCGGTCTGCTGGATAAAGATGAAAATGCCAAGTTGGGGCTAAAGGAATTCCATGTTCTCTGGAACAAACTTCAGAAATACCTGGAAATCTTCA
AGAGTCACGACACAGACAACTCGGGTACGATGAGCTCCCTCGAAATGAGAGCCGCCGCTACTAAAGCA---GGTTTC---
CAGGTGAACAGCGCTGTGGTCAATCGCTATGCGGATGCTCAGTTTGCAATCGACTTTGACAGCTTTGTGAGCTGTCTCATTAAGCTGGAAATGCTCTTCAAAATGTTTAAGGCCCTGGAGAAAGGAGACTCAGGGAAGATTGAGC
TGAACATACAACAG------TGG------ATGTGCCTGGCAATGTAT------

 ---------------------ATGACATCT---------------------------GGCATTGGCACCAATGCACAGGCAGTGAAGTTCTACCAGCAAGATTATGAGGCTCTGCGG---------------
CAGCAGTGTCTAGAGACCGGCCGCCTGTTTCAGGATGATTGCTTTCCTCCTGAGCCTAAATCACTGGGCTACAACCAGCTGGGACCATACTCATCAAAGACTAAGGGCATTGTTTGGAAGAGGCCAACGGAGCTTTGTTCCAAC-
--
CCACAGTTCATTGATGACGGCGCTACAAGGACAGACATTCGGCAGGGAACTCTGGGTGACTGCTGGCTCTTAGCTGCCATAGCTTCTCTGACTTTGGAGCAGGACATTCTGGCTCGTGTGGTGCCTCCTGACCAAAGCTTTACTG
AAGGCTATGCTGGGATATTTCACTTTCAGTTCTGGCAGTTTGGTGAGTGGGTGGATGTGGTGGTTGATGATCGTTTGCCCACCAGAGATGGAAAGCTGCTGTTTGTTCACTCAGCGGAGGGCTCAGAGTTTTGGAGTGCACTGCT
GGAGAAGGCCTACGCTAAGGTGTATGGCAGCTATGAGGCTTTGACAGGAGGAAACACTATT------
GAGGGTTTTGAGGATTTCACCGGGGGAATTGCAGAAACATACAACTTAAAAGAGGCTCCAGCAAATCTCTTTCACGTTATTCAGAGGGCCCTGAGCCTGGGTTCACTGATGGGTTGCTCT-------------------------
-----------------------------------------------------------------------------------------ATCACCAGTGCCTGTGAG------
ACAGAGGCAGTTACATCTCTAAAGCTTGTAAAAGGACATGCATACTCTGTCACCGGTGCAGAGGAGGTTCATTTTAAAGGGCAGCAAGTGCAGTTGGTTCGCATCAGGAACCCATGGGGTGAGGTGGAATGGACAGGCCCTTGGA
GTGATGGATCCAGTGAATGGAGCCACGTCACTGAAGATGAGAAGTTGAAGTTAAACAATGTGGCTGAAGATGGAGAGTTCTGGATGTCCTACTCAGACTTCATCAGGAATTTCTCCAAGCTGGAGATCTGCAATTTGACCCCAGA
TACACTCACTAGTGATGATGTGGGTCGCTGGAACTACTGCCAGTTTGAAGGGATGTGGAGGGTCGGCTCCACTGCTGGCGGCTGCCGGAATCACTCAGCCACATTCCCATCCAATCCTCAGTTCCTGTTGCGTCTGGAGGATGTG
GATGATAATCCT---------TGCACCTTTCTGGTGGGGTTGATGCAAAAGGATGGACGACGGCTGATGAAGGTTAACCCCAACCTCGAGACAATCGGCTTTGCCATTTATAAGGTTCCAGAT---
TACAAAGGTTGCAAAAATGTCCGCCTCGGGCCTGACGTCCTGCTGCGTCAGAAACATGTAGCCATGAGCCCCACTTTCATCAACACACGGGAAGTGTGTGGCCGCTTCAATCTCCCACCGGGAGAATATGCCATAATTCCCTCAA
CCTTCCAGCCCCACAAGAACGGCAGCTTCATTCTCAGGGTGTTCACAGAAAAAGAG------------------------------------------------------------------------------
TTGAAGCAACTCTTCAAGCAGCTGGCTGGA---------------------------------------------------AATAAT------------
ACGGAGGTGTCCGCCTTTGAACTGATCAAAATTTTAAACAACGCCGTTTCT---------------------CAG---
CGGTCTGACATTAAAACTGGTGGATTCAGCGTTGAGACAGGTCGCCTTATGGTCAGTCTGCTGGATAAAGATGAAAGTGGGAAGTTGGGACTGATGGAATTCCAGTTGCTTTGGAAGAAAATCCAGAAATACTTGGAGATCTTCA
AGAGTCATGATACAGACAAATCTGGCACCATGAGCTCCCATGAGATGAGAGATGCTGCCAGTAAA------GGGTTC---
CAGATCAACAGTGCAGTGGTAAATCGTTACGCTGATGCTCAGTATGCCATACACTTTGACAGCTTTGTAGGCTGTCTCATTAAACTGGAAATGCTCTTTAAAGTGTTTAAGACCCTGGAAAGGGATGACTCGGGCAAGATTGAGC
TGAACATGCAGCAG------TGG------CTGTGCCTGGCAATCTAC------

 ---------------------ATGTCTAAT---------------------------GGGCTGGGGACAAATGAGAAGGCCATTCCCTACAACAAGCAGGACTACCAAAGCCTGAAG---------------
CAGGAATGTCTGGCTAAAAGAGCCTTGTTTTGTGACCCAACTTTCCCCGCTGAATCTGACTCTCTGGGGTACAATGAACTGGGACGATACTCCTTTAAAACCAAAGGAGTGCAGTGGAAGAGACCCAAGGAGCTGTGCTCAAAC-
--
CCTGAGTTCATCATGGACGGGGCCAATCGGACAGACATCTGCCAGGGAGCTTTAGGAGACTGTTGGCTTTTGGCGGCCATTGCGTCTCTGACTCTGGATAAGGAAATCCTGGAACGTGTAGTTCCATCAGGACAGAGTTTTACAG
AGGATTACGCTGGCATCTTTCACTTTCAGTTTTGGCAGTATGGCGAATGGGTGGATGTGGTCATTGATGACCGGCTGCCGACCAGAGATGGGAAGCTGCTGTTTGTGCACTCAGCCGAAGGCTCTGAGTTCTGGAGCGCTCTGTT
GGAAAAAGCCTATGCAAAGGTAAATGGCTCATATGAAGCGCTGACAGGAGGTTCAACCACT------
GAGGGATTTGAGGATTTCACTGGAGGAATCACTGAGAACTATGAGCTGAGCAAAGCTCCTCCCAATCTGTTCAAGCTCGTACAGAAAGCACTGGCGCTGGGATCATTGCTGGGCTGCTCT-------------------------
-----------------------------------------------------------------------------------------ATTACCAATTCATATGAG------
ACTGAGGCAGTGACGAGTCTGAAGCTGGTGAAGGGACATGCATACTCAGTCACGGCTGCAGAGGAGGTTCACTTCAGAGGCAGTCTGGTTCAGCTGGTGCGAATCAGGAACCCGTGGGGTGAGGTGGAGTGGACGGGGGCCTGGA
GCGACAATTCCAAAGAGTGGGACAGTGTCCGACCAGAAGAGAAAGCTAAACTGGATTATTCAGCTGAAGATGGAGAGTTCTGGATGGCGTATTCTGACTTCATACAGCAGTTCTCCAAGTTGGAGATCTGTAATCTGACTCCTGA
CACTCTTTCGAGTGAAGAAGTGAATCGCTGGAGCTACAGTCAGTTTGAAGGCAACTGGAGGGTGGGCTCCACTGCGGGAGGCTGCAGGAACAACCCTGCAACGTTCTGCTCAAACCCTCAGTTTATGATCAAGCTGGAGGAAATA
GATGATGATCCT---------TGCACATTCCTAGTGGGTTTGATACAGAAAGATGGTCGTAAAGACAAGCGCTTTGGACGAGACCTAAACACCATCGGCTTTGCCATCTATAAGGTTCCGGAT---
TTAAAGGGTCGCAGCAATATTCATCTGGGCCCTGATGTCCTGCTGCGTCAGCGTTCAGTTGCTGGGAGCAACACCTTCATCAACCTGCGAGAGGTGAGCGAGCGCTTCAAACTGCCTCCGGGAGAATACATCATCATCCCCTCCA
CCTTTGAACCGCACCGCAAAGGAAGTTTCATTCTGCGGGTGTTCGCAGAAAAAGAG------------------------------------------------------------------------------
TTCAAACGCCTCTTCAGTCAGGTTGCTGGA---------------------------------------------------AGCGAC------------
TCTGAGGTCTCTGTATTGGAGCTCCAGCAGATCCTGAACACCGTTGTCTCG---------------------
AAACGTAGATCTAATGTGAAGACAGACGGATTCAGTTTGGAAACCTGCCGTCACATTATCAGCCTGCTAGATAAAGATGGCAGTGGCAAACTCGGCCTTCTGGAGTTTCACACACTGTGGATGAAGATCCAGAAATATTTGGAAA
TTTTTAAGCACCGCGATACAGACAACTCAGGTACCATGAGTTCTCTTGAGATGCGAGATGCGGTAAAAGAAGCA---GGGTTC---
CAGCTGAACAATGATGTAATCGCACGATATGCCAACCAGGAATACGCCATTGACTTTGATAGCTTCGTCAGCTGCCTGATCCGCCTCGAGCTGCTCTTCAAAATGTTTCAACTGTTCGATAAGAAGAACACAGGGAAAATCGAAC
TGGATATCCTGCAG------TGG------CTCTGCCTGGCCCTCAGT------

```
 ---------------------ATGGCGAGC---------------------------GGCGCTGGCAGCCGGGCCAACGCGATCCTCTTTAACCACCAGGACTTCGAGCAGCTGCGA---------------
TCCGAGTGCTTGCAGATCGGCTCGCTGTTTTGTGACCCCACCTTCCCGGCAGACTGGAACTCTCTGGGATACAACCAGCTGGGGCGCTACTCATCCAAAACCGGTGGTGTGGAGTGGAAACGGCCGTGGGAGCTGTGTTCTGAT-
--
CCTTGCTTCCTCGCTGACGGAGCCAGAAGAACAGACGTCTGTCAGGGAGAGCTGGGTGACTGCTGGCTCCTGGCGGCCATTGCCTCCCTGACTCTGGACCCTCAGATCCTGAACAGAGTGGTCCCTCCTGGGCAGAGCTTCACCT
CACAGTATGCCGGCATCTTCCACTTCCAGTTATGGCAGTACGGGGAGTGGGTGGACGTAGTCGTGGACGACCGGCTGCCCACCAGAGACGGGAAACTGCTGTTCGTCCACTCAGCGGAGGGAGTGGAGTTCTGGAGTGCTCTGCT
GGAGAAAGCCTACGCTAAAATGAGCAGCTCTTACGAGGCCCTGAGCGGCGGCTCGAGCATC------
GAGGGCTTCGAGGATTTTACCGGCGGCATCGCAGAGAGTTACGACCTGAAGGAAGCCCCGCCCTTCCTGTTCCACATCGTCAGGAAAGCCCTGAAGCTTGGATCGCTGCTCGACTGCTGG-------------------------
-----------------------------------------------------------------------------------------ATTTCCAGCTTGTATGAG------
ACGGAGCTCATCACGAGTCAGAAGCTGGTCAAAGGCCACGCCTACTCCATCACAGCGGCCGAGCAGGTGCATCATTGTGGTTCTCTGGTGGAGCTTATCCGGATCAGGAACCCCTGGGGTCAGGTGGAGTGGACGGGCGCCTGGA
GCGACAGCTCGAAGGAGTGGGATGGCGTTTGGGCAGAGGAGAAGAGGCGGCTGGATTGCTGCGCCGAGGACGGCGAGTTCTGGATATCCTATCACGACTTCCTGAGTCACTTCTCCCGCCTCGACATCTGTAACCTGACACCTGA
CACGCTGACCAGCAACGAGGTCGGCCGCTGGAACTTTGCCGAGTTTGAGGGCACGTGGAGGGTGGGATCCACCGCTGGTGGCTGCAGGAACTACCCCGCCACCTTCTGCTCCAACCCACAGTTCTTCATCCGGCTGGATGATGTG
GACGACGATCCT---------TGCACCGTCCTTATCGGTCTGATGCAGAAAGACGCTCGAAGGGAGAGGCGCTTCGGACGCGACCTCAACACCATCGGCTTCGCCATCTACAAGGTGCCCGAT---
TACAAAGGTCGCACTAACGTCCGCCTGAGCCCGGACATCCTCCTGAGAGACCAGTCAGTGGCTGGCAGTCACGCCTTCATCAACCTGAGGGAGGTGTGCGATCGCTTCAAATTGCCACCCGGAGAGTACGCCATCGTCCCGTCCA
CCTTTGAACCGCACTGCAAAGGAAGTTTCATCCTGAGGGTGTTCACAGAGAAGGAG------------------------------------------------------------------------------
CTCAAACACCTCTTCATGCAGATCTGTGGA---------------------------------------------------AACGAC------------
TCGGAGATCTCCGCCTTTGAGCTCCAGCAGATTTTGAACAAAGCGGTGACT---------------------CAG---
AGGTCGAACATTAAAACCGATGGCTTCAGCCTGCAAACCTGCCGTGACATCATCAGCCTGCTGGATACTGACGGCAGCTCCAGACTGGGCCTGCTGGAGTTCCACAGCCTGTGGATGAAGATGCAGACGTACCTGGAGATCTTTA
AGAGTCACGACGCGGACGGGTCTGGCACGATGAGCAGCCACGAGATGAGAGCGGCTCTGGCTGAAGCC---GGGTTC---
CAGCTGAACAGCACTGTGGTCGCTCGCTACGCCGACAGAAGCTACGCCATCGACTTCGACCGTTTCATCGGCTGCTTGATCCGTCTGGAGATACTCTTCAGGATGTTCAGGACTCTGGATAAGCAGCAGCGGGGCTGGATCCAGC
TCGACCTGCAGCAG------TTT------GCGGATGTTACCCAATCAGCA---

 ---------------------ATGTCTGGC---------------------------GGTCTGGGCACAAATAATAAGCCCCTGAAATACTTAAACCAGGATTATGGGCAGCTGAAG---------------
GCGCAGTGTCTGGCGTCTAACTCTCTGTTTGAGGATGAGCCCTTCCCTGCAAGCCAAGCGTCTCTGGGGGTCAAAGAACTCGGACCCGATTCTGACAAAACCAAAGGGATTGTCTGGCTGAGACCGATGCAAATTCATCCCAAA-
--
CCGGAATTTATTATCAGTGGCGCAACCCGCTCTGACATTCGCCAGGGGTCCCTAGGGGACTGCTGGTTCCTTTCCTCCATTGCTTCCCTTACACTGAATGAAGAATATCTGTCCCAGGTCGTTCCGGGGGATCAGAGCTTTCAGA
CCAACTATGCAGGCATCTTTCACTTCAAGTTCTGGCAGTACGGTGAGTGGGTGGATGTAGTTGTGGATGACCGACTCCCAACAAAGAAAGGAAAACTGGTGTTTGTCAAGTCAGCCGAGGGCAACGAGTTCTGGAGCGCGCTGCT
GGAAAAGGCTTATGCCAAGGACAGTCTTCCTTACAAAGCTGGAAGTTATATAAGTCCTGTGTGCCCCCCCCCCCATACACCGTATATCAGTTCCTTAGCTAAAGTA------ATGCCCAAGGGTCCTGCTTCTGTA------
AATGCCAATCAAGCGATTAGCCCAAACCCGCTA------TGTGCA----------------------------------------------------------------------------------------------------
--------------AAATCCGACAGTGGGAAA------
GTGGAAACTGTTGCAAAGAACAACGTAGTGAAGAATCACGCTTACACCATCACCAGGGCGGAAGAGGTTTCCTACCGTGGAGAGAAAGTTCAGCTGATCCGGCTGAGAAATCCCTGGGGTAAAACAGAATGGAACGGCGCATGGA
GTGATAATGCACCTGAATGGGACGACATTGACTCAGAGACAAGAGCGGCTCTAAATACTCAGGGTGACGATGGAGAAGTCTGGATGCCATTCTCGGATTTCATTAGTGAGTTCTACCGACTGGATATCTGTAACCTCAGCCTGGA
CTGTGTGTGCAGCAAGGAGGAGCGGCGCTGGTGCCTGACCCAGTTCTATGGGAGCTGGAAGAGCGGATGCACTGCGGGCGGATGCAAGAAATATCCAGACACATTTTGGATCAACCCGCAGTTCCGGATTAAGCTGGAGGAGCCG
GATGATGAG---GCACAGAGCTGCACGGTGATTGTGGCGCTGATCCAGAAGAACCGCCGGAAGATGAAGCCCACGGGAGGGGAAGAGTTTGCCGTTGGCTATTACTTGTATCCGATCCCTAAA---
TTGCAGGGAAGCCCCGATGTCCCTCTGGGGAAAGATTTTTTCTTGAAAACCAAATACGTGGCTTGGACAGACGTGTACAAGAAGCACCGGGAGACCTGCTGCCGCCACAAGCTGCCCGTCGGCGAGTACGTCCTCCTCCCGCACA
CCTACTACCCCTGCCAGGAGGCCGATTTCTGCCTTCGAGTCTTCTCAGAAAAGAAA------------------------------------------------------------------------------
ATTGGAGACATTCTTACTGAGCTTGAGCAA---------------------------------------------------CAGAAA------------
ATGGAAATGAGTGCAGAGGAACTGAGGATCAATCTGAGCAGGATTCTATCA---------------------AAG---
AGAAAGGACATTAAATCGGACGGGTTCAGCCTGGCGACATGCAAAGAAATTATCAACCTTTTTGACACTGACCTGACAGGGAAGCTGAGTTACAAGGAGTTCCGACCTCTATGGATAAAGTTAGATAAATACACGAAAATATTCA
AATCAGCAGACAATGACAGATCGGAGAGCATCGAAGCCCATGAAATGCGAAATGCGTTGCAACAAGCA---GGGTTT---
AATCTGAATAACAAGATCGTGCAGCGCTACATTACTAACGGCTTATCTATCAGCTTCGACAGCTTCATTGCGTGCCTCATTCGCCTGGAGACCTTGTTCGAAATGTTTGAGCTGCTGAAAACAAATGAGAGTGGAGCCCTGAGTC
TGTCTCTATCTGAG------TGG------CTCTGCACCGCCATGATG------

 ---------------------ATGTCTGGC---------------------------GGTCTGGGCACAAATGATAAGCCCCTGAAATACTTAAACCAGGATTATGAGCAGCTGAAG---------------
GCACGGTGCCTGGAGTCCAACACTCTGTTTGAGGATGAGACCTTCCCTGCAAGCCAAGCCTCAATGGGGGTCAAAGAACTTGGACCGAATTCTGACAAAACCAAAGGGATTGTCTGGCTGAGACCATTGCAAATTCATCCCAAG-
--
CCAGAATTCATTATCAGTGGCGCAACCCGTTCTGACGTTCGCCAAGGTTCTCTAGGGGACTGCTGGTTCCTTTCTTCTATTGCATCTCTTACACTGAATGAAGAATATCTGTCCCGGGTTGTTCCGGGGGATCAAAGCTTTCAGA
CCAACTATGCGGGCATCTTCCATTTCAAGTTCTGGCAGTACGGTGAGTGGGTGGATGTAGTTGTGGACGACAGACTCCCTACAAAAAAAGGAAAACTGGTATTTGTCAAGTCAGCTGAGGGCAACGAGTTCTGGAGTGCACTATT
GGAAAAGGCTTATGCCAAGCTGAATGGATCCTATGAAGCTCTGGTAGGAGGTTCTCCAGTA------
GATGCCCTTGAGGACTTCACTGGTGGTATTGCTGAACTATACTATCTGCAGAAGCCTCCAGCGGATCTGTTCCAGAGAGTCCAGAAAGCTCTGAGAGCGAAATCCCTGCTCACCTGCACC-------------------------
```

```
-----------------------------------------------------------------------------------------AAATCCGACAGCACAAAA------
GTTGAAACAGTTGCAAAGAACAATGTGGTGAAGAATCACGCTTACACAATCATCAGAGCAAAAGAGGTTATCTACCGAGGCGAAAAAGTCCAGCTGATCCGCCTAAGAAATCCCTGGGGTTACAAGGAATGGAATGGCCCGTGGA
GTGATAATGCATGTGAATGGGATGAAATTGATTCAGAGGTGAAAGCTGCTCTGAATACTCAATGTGACGATGGAGAAGTCTGGATGTCATTCTCCGACTTCATTAACGAGTACTACCGACTGGACATCTGTAACCTTAGTCTGGA
CTGTGTGTGCAGCAAGGAAGAGCGGCGCTGGTGCCTGACCCAGTATTCTGGGAGCTGGAAGAGCGGATGCACAGCAGGCGGATGCAAGAAATATCCAAACACATTTTGGATCAACCCGCAGTTCTGGATTAAGCTGGAGGAGCCG
GATGATGAC---GCACAGTGCTGTACAGTTATTGTGGGTCTGATCCAGAAGAACCGCAGGAAGATGAAGCCCATGGGAGAGGAAGAGTTTCCCATTGGCTATTACTTATACCCGATCCCTAAA---
TTCCAGGGCAGCCATGATGTCCCTCTGGGGAAAGATTTTTTCTCGAAAACAAAATATGTGGCTTGGACAGACGTCTACAGAAAACACCGTGAGACCTCCTGCCGCCACAAGCTGCCCATAGGAGAGTACATCATTCTCCCACACA
CCTACTATCCCTGCCAGGATGCCGATTTCTGCCTTCGCGTATTCTCCAAAAAGAAG------------------------------------------------------------------------------
ATTGAAGACATTCTTAATGAGCTTGAGCAA---------------------------------------------------AAGAAA------------
ATGGAAATGAGTGCCGAGGACCTTAGGATCAATCTCAGCAGGATCTTATCA---------------------AAG---
AAAAAGGACATTAAATCAGATGGGTTCAGCCTGACGACATGCAAAGAGATGATCAACCTGTTTGATACTGACCTGACTGGGAAGCTGAACTGCAAGGAGTTCAGACCACTATGGATAAAGTTAGATACATACATGAAAATATTCA
AATCGGTGGACAATGACAGATCCGACAGCATCGAGGCCCACGAAATGCGAAATGCGTTGCAACAAGCA---GGGTTT---
AATCTGAACAACAAGATCGTGCAGCGGTACATTTCTAATGAATTATCTATCAACTTTGACAACTTTATTGCGTGCCTGATTCGCCTGGAGAACTTGTTCAAAATATTTGAAGTGCTGAAAACAACTGAGAGCGGAGTCATGAGTC
TGTCCTTATCAGAG------TGG------CTTTGTACCGCCATGATG------;

END;



BEGIN TREES;

	TREE tree =
((((((RHINO_CAPN8_XM_004439538,(DOG_CAPN8_XM_849757,SHEEP_CAPN8_XM_004013988)),(HUMAN_CAPN8_NM_001143962,MOUSE_CAPN8_ENSMUSG00000038599_ENSMUST0
0000048941)),(TASMANIAN_DEVIL_CAPN8_ENSSHAG00000014684_ENSSHAT00000017417,OPOSSUM_CAPN8_ENSMODG00000005003_ENSMODT00000006284)),PLATYPUS_CAPN8_EN
SOANG00000001449_ENSOANT00000002296),(((FALCON_CAPN8_XM_005241871,(CHICKEN_CAPN8_XM_426117,TURKEY_CAPN8_ENSMGAG00000008330_ENSMGAT00000009604)),T
URTLE_CAPN8_ENSPSIG00000014086_ENSPSIT00000016134),ANOLE_CAPN8_ENSACAG00000002512_ENSACAT00000003160)),((XENOPUS_CAPN8_ENSXETG00000030338_ENSXETT
00000061954,X_LAEVIS_CAPN8_NM_001085983),((XENOPUS_CAPN8L_ENSXETG00000031732_ENSXETT00000062202,XENOPUS_CAPN8_ENSXETG00000018607_ENSXETT000000622
68),X_LAEVIS_CAPN8_NM_001088543))),((TILAPIA_CAPN2OR8_ENSONIG00000007284_ENSONIT00000009192,ZEBRAFISH_CAPN8_ENSDARG00000055715_ENSDART00000078111
),((TILAPIA_CAPN2OR8_ENSONIG00000009988_ENSONIT00000012572,PLATYFISH_CAPN2OR8_ENSXMAG00000006017_ENSXMAT00000006157),MEDAKA_PCAPN2_ENSORLG0000001
1856_ENSORLT00000014856)));

END;
```

## (*h*) *CAPN9*

```
#NEXUS
BEGIN TAXA;

	DIMENSIONS NTAX = 24;

	TAXLABELS

		'XENOPUS_CAPN9_ENSXETG00000023310_ENSXETT00000060247' 'X_LAEVIS_CAPN9_NM_001092528'
'CHICKEN_CAPN9_ENSGALG00000011136_ENSGALT00000018152' 'TURKEY_CAPN9_ENSMGAG00000012108_ENSMGAT00000013646'
'ZEBRA_FINCH_CAPN9_ENSTGUG00000009921_ENSTGUT00000010378' 'TURTLE_CAPN9_ENSPSIG00000016067_ENSPSIT00000018324'
'ANOLE_CAPN9_ENSACAG00000001768_ENSACAT00000002035' 'PIG_CAPN9_ENSSSCG00000010182_ENSSSCT00000011148' 'RHINO_CAPN9_XM_004432359'
'HUMAN_CAPN9_NM_006615' 'CAT_CAPN9_XM_003993929' 'MOUSE_CAPN9_ENSMUSG00000031981_ENSMUST00000093033'
'TASMANIAN_DEVIL_CAPN9_ENSSHAG00000017756_ENSSHAT00000021107' 'OPOSSUM_CAPN9_ENSMODG00000009241_ENSMODT00000011757'
'PLATYPUS_CAPN9_ENSOANG00000013738_ENSOANT00000021670' 'STICKLEBACK_CAPN9_ENSGACG00000019761_ENSGACT00000026171'
'FUGU_CAPN9_ENSTRUG00000002630_ENSTRUT00000006163' 'MEDAKA_CAPN9_ENSORLG00000019722_ENSORLT00000024514'
```

'PLATYFISH_CAPN9_ENSXMAG00000016015_ENSXMAT00000016192' 'TILAPIA_CAPN9_ENSONIG00000000043_ENSONIT00000000052' 'ZEBRAFISH_CAPN9_ENSDARG00000012341_ENSDART00000007732' 'COELOCANTH_CAPN9_ENSLACG00000001048_ENSLACT00000001182' 'SMALLERSPOTTEDCATSHARK_TRANSCRIPTOMECONTIG20672' 'LITTLESKATE_TRANSCRIPTOMECONTIG91257' ;

END;

BEGIN CHARACTERS;

DIMENSIONS NCHAR = 2031;
FORMAT

DATATYPE = DNA

GAP=-

MISSING=?

NOLABELS

;

MATRIX

ATTCATGGGAAAACCTACGAGCAGCTCAAAAATGACTGCCTCAGAAAAGGGGTTTTGTTTGAAGATGTTGATTTCCCTGCAAATGATTCATCCCTATTCTACAGTGAAAAACCCAGTGTTCCTTTTGTGTGGAAAAGACCCAAGG
AAATGGTTGCAGATCCTGAGTTTATTCTAGGCGGTGCATCCAGAACTGATGTATGTCAAGGTGACCTAGGTGACTGTTGGCTTTTAGCAGCTATTGCTTCCCTTACACTTAATGATAAGATATTATACAGAGTGGTACCACCAGA
TCAGTCCTTTAGCACTGGGTATGCTGGTATATTTCATTTCCAGTTTTGGCAACACAATGAATGGCTTGATGTTGTTATTGATGATCGACTGCCAACCTTCAGGAACCGCTTGGTCTTTGTCCATTCGGCTGATCTCAATGAATTT
TGGAGTGCACTATTGGAAAAAGCGTCTTGTTTACTGAATGGAAGCTATGAAGCTTTAAAAGGCGGAAGTACTTTAGAGGCTATGGAAGATTTCACTGGAGGTGTTACAGAAACTTATGAAATTAGAAGTGCCCCTGCCAATCTGT
TTGATATATTGGACAAAGCTATTAAGAAAGGATCGATGTTGGGCTGTTCCATTGATATTACAAGTTCTGCTGAAACAGAAGCTAAAACACCTCAGGGACTTGTGAAAGGCCACGCTTATTCAATAACTGGAGCAGATGTGGTAAA
TTTCAAAGGTCAGAAGGTAAAACTCATTCGAATCAGGAATCCATGGGGGCAAGTTGAGTGGAATGGGGCTTGGAGTGACAACTCTTCAGAGTGGAACATTATTGGTGCTGCTGAAAAAAACCGACTATCTCAGGCATCTCTCGAT
GATGGAGAATTCTGGATGGACTTTGAGGACTTCAAAAGACACTTTGACAAACTTGAGATCTGTAATCTGACTCCCGATTCATTGGATGACGACGCCAAACATAAGTGGGAAGTGACGGTACATGAGGGAAGCTGGATCAGAGGCT
CCACTGCTGGAGGATGCAGGAATTTTTTAGAAACATTTTGGTCAAATCCACAAATCAAGTTGAGTCTCGATGGTCAAAAGGAATGCACGTTTATTGCAGCCTTAATGCAAAAGAATAGAAGAAAGTTAAGAAAAACTGGAGCTGA
CTTGCTTACAATTGGATATACTATTTATCAGTGTCCTGGATCTGAGTCAATCCTGTCAAAAGATTTCTTTAAGTTTAATCCATCTAAGGCAAGAAGCAAAACATACGTTAATCTGAGAGAGATCTCCCAGAGGTTCAAGCTTCCA
CCAGGGGACTACATGTTCATTCCAACAACGTTTGAACCTCATCAGGAAGCTGATTTTTGCCTCAGAATATTTTCAGAAAAGAAAGCAGACATAAAAGAGATGGATGGAGAAGTAAATATTGATCTTCCAGATCCACCTGCCCCGA
CTCCTGAAACCGAAGAAGAAAGACAATTTCGTACTTTGTTTGAACAAATTTCTGGACAAGACTTGGAAGTATCAGCTGAGGAATTGCAATATGTTCTAAATGCTGTTCTTCGGAGAACAAAAAATATTAAATTCAAGGAAATGAG
CCTTCTATCATGTAACAACATCATTTCTCTAATGGATGCCAGCGGCAATGGAAAACTTGGGTTTGATGAATTCAAGATATTTTGGGACAAACTGAAGACCTGGATTGGTATCTTCATGCAGTATGATTTAGACAAGTCAGGCACT
ATGTCTTCATATGAACTCCGACTGGCGTTGAAATCTGCAGGTTTTCATCTTAATAATATTGTATTGCAACTGATTGTCCTGAGATACGCAGATGATCAGTTCCAAATAGAATTTGATGACTTCCTTAACTGTTTGGTTCGTCTGG
AGAACTCTTACAGGGTCTTCAAGCAGCTAAACCGAAGCGATATCCAATTAAACCAAGCAGAGTTTATTGCCCTGACAATGAATATT???????????????????????????????????????????????????????????
?

GTTCACGGGAAAACATATGAGCAGCTCAAAAGCGAATGTCTCAGTAAAGGGGTTTTGTTTGAAGATGTTGATTTCCCTGCAAATGATTCATCCCTCTTCTACAGTGAGAAACCCAGTGTTCCTTTTGTTTGGAAAAGACCTAAGG
AAATGGTTGCAGATCCTGAGTTCATTCTAGGCGGTGCATCCAGAACTGATGTTTGTCAAGGTGACCTTGGTGACTGTTGGCTTTTAGCAGCTATTGCTTCCCTTACACTGAATGATAAGATATTACACAGAGTGGTGCCACCAGA
TCAGCCCTTTAGCACTGGGTATGCTGGTATATTTCATTTCCAGTTTTGGCAACATAATGAATGGCTTGATATTGTTATTGATGACCGACTACCAACCTTTAGGAATAGATTGGTCTTTGTCCATTCAGCTGATCTCAATGAATTT
TGGAGTGCACTGTTGGAAAAAGCATACGCCAAACTGAATGGTAGCTATGAAGCTTTAAAAGGTGGCAGTACTTTAGAGGCTATGGAAGATTTCACTGGAGGTGTTACAGAAACTTATGAAATTAGAAGTGCCCCTGCCAATCTGT
TTGAAATAATGGACAAAGCTCTTAAAAAGGGATCAATGTTGGGCTGTTCCATTGATATCTCAAGTTCATCTGAAACAGAAGCTAAAACACCTCAGGGACTTGTGAAAGGCCACGCCTATTCAATAACTGCAACCGATGTGGTCAA

TTTCCAAGGCCAAAAGGTAAAGCTCATTAGAATTAGGAATCCATGGGGGCAAGTTGAGTGGAATGGTGCTTGGAGTGACAACTCTTCAGAGTGGAATATTATTGGTGCTGCTGAAAAAAACCGACTATCTCAGGCATCTCTTGAT
GATGGAGAATTCTGGATGGACTTTGAGGACTTCAAAAGACACTTTGACAAACTTGAGATCTGTAATCTGACTCCTGATGCTTTGGAGGAAGACACCAAATATAAGTGGGAAGTGACGGTACATGAGGGAAGCTGGATCAGAGGCT
CCACTGCTGGAGGTTGCAGGAATTTTTTAGAAACATTTTGGTCAAATCCACAAATCAAGCTGAGTCTGGAAGGTCAAAAGGATTGCACATTTATTGCAGCCTTAATGCAAAAGAATAGAAGAAAGCTGAGAAAAACTGGAGCCGA
CTTGCTAACGATAGGATATACTATTTATCAGTGTCCTGGATCTGAGTCAATCTTGGAAAAAGATTTCTTCAAATTTAATGCATCTAAGGCAAGAAGCAAAACATATGTAAACCTGAGAGAAGTGTGCCAGAGGTTTAAGCTTCCA
CCAGGGGATTACATGTTCATTCCAACAACGTATGAACCTCATCAGGAAGCTGACTTTTGCCTCAGAATATTTTCGGAGAAGAAAGCGGACATAAAAGAGATGGATGGAGAAATAAATGTTGATCTTCCAGATCCAACTGCGCCGA
CTCCTGAAACTGAAGAAGAAAGACAATTTCGCACTTTGTTTGAGCAAATTTCTGGTCAAGATTTGGAGGTATCAGCTGAGGAATTGCAATATGTTCTGAATTCTGTTCTTCGGAGGACAAAAAATATTAAATTCAAGGAAATGAG
CCTTCTGTCATGTAACAACATCATTTCTCTAATGGATACTAGCGGCAATGGAAAACTTGGGTTTGATGAATTCAAGATATTTTGGGACAAATTAAAGACCTGGATTGGTATCTTCATGCAGTATGACTTAGACAGGTCAGGCACT
ATGTCTTCATATGAACTCCGATTGGCGTTGAAATCTGCAGGTTTTCATCTTAATAATATTGTGTTGCAACTGATTGTCCTGAGATATGCAGATGATCACTTCCAAATAGAATTTGATGACTTCCTAAACTGTTTGATTCGCCTGG
AGAACTCTTACAGGGTCTTCAAGCAATTAAACCAAAGCAATATCCAAATAAACCAAGCTGAGTTTATTGCCCTAACAATGAATATT???????????????????????????????????????????????????????????
?

ACCCACGGGAAGGGCTACCAGGAGCTGAAGCAGGAGTGCCTGCGCAGCGGCTGCCTCTTTGAGGACCCTGACTTCCCCGCCAACAATGCCTCCCTTTTCTTCAGTGAAAAGCCACCGATTCCCTTCCTCTGGAAGAGGCCTGGGG
ACATTGTCAAAGATCCCAAGTTTATCCTCGAAGGAGCCACAAGAACTGACATTTGCCAAGGGGATCTTGGTGACTGCTGGCTATTAGCCGCCATAGCTTCTCTCACACTGAATGAGAAAACACTAGCAAGAGTAGTACCGCTGGA
TCAAAATTTTGGGCCAGGTTATGCTGGGATATTTCACTTCCAGTTCTGGCAGCACAATGAGTGGCTGGATGTTGTGATTGATGATCGATTGCCCACCTTCAAAGACCGGTTGGTATTCGTACATTCAGCAGAACACAATGAATTT
TGGAGTGCTTTACTAGAGAAAGCCTATGCCAAGCTGAATGGCAGCTATGAGGCTCTGAAAGGAGGCAGTACAATAGAAGCCATGGAGGATTTCACTGGGGGAGTGGGAGAAATGTATGAGGTTAAAAAAGCTCCTGACAATTTCT
ATGAAATCCTAGAGAAAGCTCTGAAAAGATCGTCAATGGTGGGCTGCTCTATCGATACCAGCAGTGCTGCAGAGTCAGAAGCCAGGACACCCTTTGGCCTCATAAAAGGCCATGCTTACTCTGTAACCGGCATTGATGAGGTGAG
CTATCAGGGCCAGAAAGTGCAGCTCATAAGAATAAGGAACCCCTGGGGACAAGTGGAGTGGAACGGGCCTTGGAGTGACAACTCTCTAGAGTGGCGTTTGGTCAGCCCATCGGAGCAGAAGCGCTTGGCTCAGACAGCACTGGAT
GATGGAGAATTCTGGATGAAATTTGAAGATTTCAAAGTGCATTTTGATAAGGTTGAGATCTGCAACCTGACTCCAGATTCCCTGGAAGACAACACTACCCAAAAGTGGGAGGTGACCATCCATGAGGGGAGCTGGGTCCGGGGAT
CCACTGCAGGAGGATGCAGAAATTTTCTAGACACTTTCTGGACAAATCCACAAATCAAGCTGCATCTGGATGGTCAAGATGACTGCACGTTCATAGCAGCACTGATGCAAAAGGATCGCCGTAAACTCAGGAAGCTTGGTGCTGA
AATGCTAAGCATCGGCTACTCCATTTATGAGAGCCCTGGTGGAGATGAACACTTGAGCAAAGACTTCTTCAGGTACCACGCCTCCAAGGCCAGGAGCAAAACCTACATCAATTTAAGGGAAGTGTCTGACAGATTCAAGCTGCCA
CCAGGTGACTACATCCTCATTCCAACCACATTTGAGCCACATCAGGAGGCAGATTTCTGCCTGAGAATCTTTTCAGAGAAGAAGGCCATCACTGAGGATCTGGATGAAAATGTTGCCATTGATCTCCCCGAGCCTCCAAACCCAA
CCCCAGAATCTGAGGAAGAAAAGCAGTTCCGGGCACTGTTTGAGCAGATCTCAGGAAAGGACATGGAAGTAGCTGCAGAAGAACTTGAATATGTTCTAAATGCAGTATTAAAAAACACAAAGGATATCAAATTCAAGAAGTTAAG
TCTCATTTCATGCCGAAACATCATTTCTCTTATGGATACCAGTGGCAACGGGAAACTGGAATTCAGTGAGTTCAAAGTTTTCTGGGAAAAACTGAAGAAATGGATAAATATCTTTCTTCGATTTGACTTTGACAAATCTGGCTCC
ATGTCCTCCTATGAGCTTCGCAGTGCTCTCAAAGCTGCAGGATACCAACTCAACAACTACCTGCTGCAGCTGATCGTCCTCCGATACTCTGATGAGCAGTTCCAAATTGATTTTGATGATTTTCTAAACTGCCTAATTCGCTTGG
AGAATGCAAGTCGAGTATTCCAAGCATTGAGTGTGAAAAACATTAACTTGAATATTGGCGAGTTCATCAACCTGGCAATGAACATC???????????????????????????????????????????????????????????
?

ACCCACGGGAAGGGCTACCAGGAGCTGAAGCAGGAGTGCCTGCGCAGTGGCTGCCTCTTTGAGGACCCTGACTTCCCTGCCAATAATGCCTCCCTCTTCTTCAGTGAAAAGCCACCGATTCCCTTCCTCTGGAAGAGGCCTGGGG
ACATTGTCAAAGATCCCAAGTTTATCCTCGGAGGAGCCACAAGAACTGACATTTGCCAAGGGGATCTTGGTGACTGCTGGCTATTAGCAGCCATAGCTTCTCTGACACTGAATGAGAAAACACTAGCAAGAGTAGTGCCATTGGA
GCAAAATTTTGGGCCAGGTTATGCTGGGATATTTCACTTCCAGTTTTGGCAGCACAATGAGTGGCTGGATGTTGTGATTGATGATCGATTGCCCACCTTCAAAGACCGGTTGGTATTCGTACATTCAGCTGAACACAATGAATTT
TGGAGTGCTTTACTAGAGAAAGCCTATGCCAAGCTGAATGGCAGCTATGAGGCTCTGAAAGGAGGCAGTACAATAGAAGCCATGGAAGATTTCACTGGGGGAGTGGGAGAAATGTATGAGGTTAAAAAAGCTCCTGGCAATTTCT
ATGAAATCCTAGAGAAAGCTCTGAAAAGATCATCAATGGTGGGCTGCTCTATTGATACCAGCAGTGCTGCCGAGTCAGAAGCCAGGACACCCTTTGGCCTCATAAAGGGCCATGCTTATTCTGTAACTGGCATTGATGAGGTGAG
CTATCAGGGCCAGAAAGTGCAGCTCATAAGAATAAGGAACCCTTGGGGACAAGTGGAGTGGAACGGGCCTTGGAGTGATAACTCTCTAGAGTGGCGTTTGGTCAGCCCATCGGAGCAGAAGCGCTTGGCTCAGACAGTGCTGGAT
GATGGAGAGTTCTGGATGAAATTTGAAGACTTCACAGTGCATTTTGATAAAGTTGAGATCTGCAACCTGACTCCAGATGCCCTGGAAGACAGCACTGCCCACAAGTGGGAGGTGACCATCCATGAGGGGAGCTGGGTCCGGGGGT
CCACAGCAGGAGGATGCAGAAATTTTCTAGACACTTTTTGGACAAATCCACAAATCAAGCTGCATTTGGATGGTCAAGATGACTGCACGTTCATAGCAGCACTGATGCAAAAGGATCGCCGTAAACTCAGGAAGCTTGGTGCTGA
AATGCTAAGCATCGGCTACTCCATTTATGAGAGCCCTGGTGGAGATGAACACTTGAGCAAAGACTTCTTCAGGTACCATGCCTCCAAGGCCAGGAGCAAAACCTACATCAATTTAAGGGAAGTGTCTGACAGATTCAAGCTGCCA
CCAGGTGACTACATCCTTGTTCCAACCACATTTGAGCCACACCAGGAAGCAGATTTCTGCCTGAGAATCTTTTCAGAGAAGAAGGCCATCACTGAGGATCTGGATGAAAATGTTGCCATTGATCTCCCCGAGCCTCCAAACCCAA
CCCCAGAAACTGAGGAAGAAAAACAGTTCCGGGCACTGTTTGAGCAGATCTCAGGAAAGGACATGGAAGTAGCTGCAGAAGAACTTGAATATGTTCTAAATGCAGTATTAAAAAACACAAAGGACATCAAATTCAAGAAGTTAAG
TCTCATTTCATGCCGAAACATCATTTCTCTTATGGATACCAGTGGCAACGGGAAACTGGAATTCAGTGAGTTCAAAGTTTTCTGGGAAAAACTGAAGAAATGGATAAATATCTTTCTTCGATTTGACTTTGATAAATCTGGCTCG
ATGTCCTCCTATGAGCTTCGCAGTGCTCTCAAAGCTGCAGGATATCAACTCAACAACTACCTGCTGCAGCTGATCGTCCTCCGATACTCTGATGAGCAGTTCCAGATTGATTTTGATGATTTTCTAAACTGCCTAATTCGCTTAG
AGAATGCAAGTCGAGTATTCCAAGCATTGAGTGTGAAAAACATTAAGTTGAATATTGGCGAGTTCATCAACCTGGCAATGAACATC???????????????????????????????????????????????????????????
?

ACGCACGGGAAGGGCTACGAGCAGCTGAAGCAGGAGTGCCTGCGCAGGGGCGTCCTCTTTGAGGACCCCGACTTCCCCGCCTGCAACTCGTCTCTCTTCTTCAGTGAAAACCCACCCATTCCCTTCGTCTGGAAGAGGCCTGGGG
ATATTGTCAAAGACCCCAAGTTTATCCTTGGAGGAGCCACCAGAACTGACATTTGTCAAGGGGATCTTGGTGACTGCTGGCTATTAGCAGCCATTGCTTCTCTCACACTGAATGAAAAAACACTAGCAAGAGTGGTGCCACTGGA
CCAAAATTTTGGGCCGGATTATGCTGGAATATTTCACTTCCAGTTTTGGCAGCACAACGAGTGGCTGGATGTCGTGATTGATGACCGATTACCCACCTTCAAAGGCCGCCTGGTTTTCCTGCACTCAGCTGAACTCAATGAATTT
TGGAGTGCCTTACTAGAGAAAGCCTATGCCAAGTTGAATGGCAGCTACGAGTCTCTGAAGGGTGGCAGTACAATAGAGGCCATGGAGGATTTCACTGGGGGTATAGGAGAAATGTATGATGTTAAGGCGGCTCCTGACAATTTCT
ATGAAATCTTAGAAAAAGCCCTGAAAAGATGCTCAATGGTGGGCTGCTCTATTGATACCAGCAGTGCTGCCGAGTCAGAAGCCCGAACCCCATTTGGTCTTGTAAAAGGCCATGCATACTCTGTGACTGGCATTGAGGAGGTGAG
CTATAGGGGCCGGCAAGTGCAGCTCATAAGGATAAGGAACCCGTGGGGAGAGGTGGAGTGGAACGGCCCTTGGAGTGACAACTCTGCAGAGTGGCGCTCAGTCAGCCCATCGGAGCAGAGACGCCTGTCTCAGGCAGCACGGGAT
GATGGAGAGTTCTGGATGAAGTTTGAAGATTTCAAAGTGCATTTTGACAAAGTTGAGATCTGCAACCTGACTCCAGATGCCCTGGAAGACAGCACTGCCCACAAATGGGAAGTGACCATCCACCAAGGCAGCTGGGTCCGGGGAG
CCACCGCAGGAGGATGTAGAAATTTTCTAGAGACTTTCTGGACAAATCCACAAATCAAGCTGCATTTGGATGGACAAGATGACTGCACATTCATAGCAGCACTGATGCAAAAGGATCGGCGTAAACTCAAGAAGCTTGGAGCTGA
AATGCTGACCATTGGCTACTCTATTTATGAGAGCCCAGGCAGAGATGGACACTTGGGCAAAGACTTCTTCAGGTACCACCCCTCCAAGGCCAGGAGCAAAACCTACATCAATTTAAGGGAAGTATCCCACCGATTCAAGCTGCCA
CCGGGTGATTACATTCTTATTCCGACCACATTTGAGCCACACCAGGAGGCAGATTTCTGCCTGAGGATCTTCTCTGAGAAGAAGGCCATCACTGAGGATCTAGATGAAAGTGTTGCCATTGATCTTCCTGAGCCCCTACATCCAA
CCCCAGAAACTGAAGAAGAAAAGCAATTCCGGGCACTGTTTGAGCAGATTTCAGGAAAGGACATGGAGATATCTGCAGAAGAGCTTGAATATGTTCTGAATGCTGTACTAAAAAAAACAAAGAACATAAAATTCAAGAACTTAAG
TCTCATTTCATGCCGAAATATCATTTCTCTTATGGATACCAGTGGCAACGGGAAATTGGAATTCAGTGAGTTCAAAGTTTTCTGGGAAAAACTGAAGAAATGGATAAGTATCTTTCTTCAATTTGACTTTGATAAATCTGGCTGC
ATGTCCTCTTATGAGCTTCGTGGTGCTCTCAAAGCTGCAGGATACCAGCTCAACAATTACCTGCTGCAACTGATTGTCCTCCGATACTCTGATGAGCAGTTCCAGATTGAATTTGATGATTTTCTGAACTGCCTAATACGCCTGG
AGAATGCGAGTCGAGTATTCCAAGCACTGAGTGTGAAAAACATTAACCTGAATATAGGCGAGTTTATCAACCTGGCAATGAACATC???????????????????????????????????????????????????????????
?

ACACACGGCCAGACGTATGAGGAGCTGAAGCGCCAGTGCCAACAAAGGGGGACCCTATTTGAGGATTGTGACTTCCCAGCCAATTGCTCCTCTCTCTTCTACAGTGAGAAGCCATCCATCCCCTTTGTATGGAAAAGGCCTGGGG
AAATTGTTAAAGACCCAGAGTTTGTAGTTGGAGGAGCCACAAGAACTGACATTTGTCAAGGGGATCTTGGTGACTGCTGGCTGTTAGCAGCTATAGCTTCCCTTACATTGAATGAAAACACATTAGCCAGAGTTGTACCTCAAGA
TCAAAATTTTGGACCAGGTTATGCTGGAATATTTCACTTTCAGTTTTGGCAGCACAATGAGTGGCTGGATATTGTTATTGATGACCGATTACCCACCTTTAAAGACCGCTTGGTTTTTCTGCACTCGGCCGAACACAATGAATTC
TGGAGTGCCTTACTGGAAAAAGCCTATGCCAAACTGAATGGAAGTTACGAAGCCCTAAAAGGAGGCAGTACTCTCGAAGCCATGGAAGATTTCACTGGAGGAGTTGGAGAAATGTATGACGTCAAACAGGCTCCAGACAACTTCT
ATGAAATCTTAGGAAAAGCTCTTAAAAGAGGGTCAATGGTGGGCTGTTCAATTGATACCACCAGCGCTGCTGAGTCAGAAGCTCGAACCCCCTTTGGCCTCATAAAGGGTCATGCTTATTCCGTAACTGGCATTGATGAGGTGAC
CTACCAAGGCCGAAAGGTGCAACTCATAAGGCCAAGAAATCCATGGGGGCAAGTGGAGTGGAATGGCCGTTGGAGTGACAATTCTCCTGAATGGAAGGCAGTCAGCCCGTCGGAGCAAAAACGTCTATGTCAGACAGCACTAGAT
GATGGAGAGTTCTGGATGACCTTTGAAGACTTCAAAACACATTTTGATAAAGTTGAGATTTGCAACCTAACTCCAGATGCTCTTGGGGACAATGCTACCAACAAGTGGGAAGTGACAGTCCATCAAGGGAGCTGGGTCAGGGGGT
CCACTGCTGGAGGGTGCAGAAATTTTCTAGACACTTTTTGGACAAATCCACAGATCAAGCTGCGTTTGGACGGAAAAGACGAATGCACGTTTATAGCAGCTTTGATGCAAAAGAATCGGCGTAAACTCAGGAAACTAGGTGCTGA
AATGCTGACAATTGGCTATGCTATTTATGAGAGCCCTAGCAAAGATGAACACTTGAACAAAGACTTTTTCAGATACCATCCCTCCAAGGCAAGGAGCAAAACCTATATCAACTTAAGAGAAGTGTCTGACCAATTCAGGCTGCCA
CCTGGGGAGTATGTTCTTGTTCCTACTACTTTTGAACCACACCAGGAAGCTGACTTCTGCCTCAGAATCTTCTCTGAGAAAAAAGCCATCACGCAGGATATGGATGGAAATGTTGATATTGATCTACCAGAGCCCCCTAAGCCAA
CCCCAGAAACCGAAGAAGAAAAACAGTTCCGGGCATTGTTTAAACAGGTTGCGGGAGAGGACATGGAGATAACTGCAGAGGAACTTGAATATGTTCTGAATGCCGTATTAAAAAAAGCAAAGGACATCAAATTTGACAAAGTATG
CCTCATGACATGCAGAAACATCATCTCTCTTATGGATTCCACTGGTAACGGGAAACTGGAATTTAATGAGTTCAAGATTTTCTGGGACAAACTGAAGAAGTGGCTAAGTATCTATCTTCATTTTGATTCTGATAAAACCGGCACC
ATGTCTTCCTATGAGCTTCGTAGTGCCCTAAAAGCTGCAGGATTTCAACTCAATAATTACCTGCTACAGCTGATTGTCCTCAGATATTCTAATGACCAGTATGAAATTGATTTTGATGACTTCCTAAACTGCTTAATTCGCCTAG
AGAATGCAAGCCGAGTATTCCTGGAACTAACTATGAAAAACATTAACCTGAATATAAATGAGTTTATCACCCTTGCAATGAACATT???????????????????????????????????????????????????????????
?

ACACACGGCCAAACGTACAAGGAGCTGAAACTCGAATGCCTGCAGAAAGGGATACTCTTTGAGGACTGTGATTTTCCACCCAATGACGCTTCCCTTTTCTTCAGTGAGAAGCCTCCTGTCCCCTTTGTATGGAAGAGGCCTGGGG
AAATCGTTAAGGACCCGCAGTTTATTATTGGAGGTGCCACCAGGACTGACATTTGTCAAGGAGATCTTGGTGACTGTTGGTTATTAGCAGCCATAGCATCACTTACGCTTAATGAAAAGACATTAGCCAGAGTTGTGCCACAAGA
TCAGGATTTTGGACCCGGTTATGCTGGCATATTTCATTTTCAATTCTGGCAACACAATGAGTGGCTGGATGTTGTTATTGATGACCGACTACCCACTTTCAAAGACCGTCTGGTTTTTCTCCACTCTGCTGACCATAATGAGTTC
TGGAGCGCCTTGCTGGAAAAAGCCTATGCCAAGTTGAATGGAAGTTATGAAGCTCTAAAAGGTGGAAGCACAATTGAAGCCATGGAGGATTTTACAGGAGGCGTTGCAGAAATGTATGAAGTTAAAAAGGCACCAGAAAACTTCT
ATGAAATCTTGGAAAAAGCTCTTAATAGGGGGTCAATGGTGGGCTGCTCTATTGATACCAGAAGTGCAGCTGAGACTGAAGCTCGAACCCCCTATGGCCTTATAAAGGGTCACGCATATTCAGTAACAGGTATTAATGAAGTCAA
CTACCAAGGACGGAAAGTGAAACTCATTCGCATTAGGAATCCATGGGGTCAGGTGGAATGGAATGGTCCTTGGAGTGACAATTCCCCAGAATGGAATTCTATCAGTCAATCTGAGAAAAAGCGTTTGTATCAGACAGCATTAGAT
GATGGAGAATTCTGGATGAAGTTTGAGGATTTCCAGACTCATTTTGATAAAGTTGAAATCTGCAACCTCACACCTGATGCCCTTGAGGACAACATCGCTCACAAATGGGCAGTAACAGTCCATCAAGGCAGTTGGGTTAGAGGTG
CCACGGCTGGAGGGTGCAGGAACTTTATCGAAACATTTTGGACAAATCCACAATTCAAGATCCAGTTAGATGGAAAAGATGGCTGTACATTTCTGGCGGCTCTAATGCAAAAAGACCGTCGTAAACTAAAGAAACTGGGAGCAGA

AATGCTAACAATAGGATATGCTATCTACGAGAGCCCAGATGAAGATGAGCAGGTGACAAAGGACTTTTTCCGCTATCATGCATCCAAAGCCAGGAGCAAAACATATATAAACTTGAGAGAAGTCTCTGACCGATTTACATTACCA
CCTGGCAATTACATTATTGTCCCTACCACATTTGAGCCACATCAAGAGGCTGATTTCTGTCTACGAATTTTTTCTGAGAATAAAGCACTTACTAAGGAGATGGATGGAAAAGTTGACATCAACCTGCCAGAGCTTCCAGAACCAA
CACCCGAAACAGAAGAAGAGAAGCAGTTCCGAACATTATTCACACAGATTTCAGGAGAGGACATGGAGATCAGCCCAGAGGAACTTGAATATGTTCTGAATGCTGTATTGAAAAAAACAAACAACATCAAATTCAAGAAAATAAG
TCTTCTTTCATGTAAAAATATCATTTCTCTAATGGATTCTAGTGGAAATGGAAAACTGGATTTCAGTGAGTTCAAAATTTTCTGGGATAAACTGAAAAAGTGGATTGGTATCTACCTCCATTTTGATGCAGATCACTCTGGCACC
ATGTCGGCCTATGAACTTCGTCTTGCTTTAAAAGCAGCAGGGTTTCAACTTAATAATTATCTGCTTCAGCTCATTGTTCTCCGATATGCTGATGATCAGTACCAGATTGAATTTGATGATTTCTTAAACTGCCTAATTCGCCTAG
AAAATGCCAGCAGAGTCTTCCAGGCATTAAGTGTGAAAAATATTAACCTGAATATAGGCGAGTTTATCAACTTAACAATGAACATC???????????????????????????????????????????????????????????
?

ACCCACGGCCAGAGCTTCGAGGAGCTGAGGCAGGCGTGCCTGCAGAAGGGCGTCCTGTTTGAGGACACAGACTTCCCAGCTGATAGCTCGTCTCTCTTCTACAGCGAGAGGCCCCAGATCCCTTTCGTTTGGAAACGGCCAGGGG
AAATAGTGAAAAACCCAGAATTCATCCTTGGAGGGGCCACAAGGACGGATATTTGCCAGGGGGAGCTTGGGGACTGCTGGCTCTTGGCTGCCATTGCCTCCCTTACACTTAATGAAAAAGCACTGGCTCGCGTTGTCCCCCACAA
CCAAAGCTTTGGCCCCGGTTATGCTGGGATATTCCATTTCCAGTTCTGGCAGCACAGCGAGTGGCTGGATGTGGTGATCGACGACCGGCTGCCCACCTTCAGAGACCGCCTTGTCTTCCTGCACTCGGCCGACCACAACGAGTTC
TGGAGCGCCCTGCTGGAGAAAGCCTACGCCAAGCTGAACGGGAGCTACGAGGCCCTGAAGGGGGGCAGTGCCATCGAGGCCATGGAAGACTTCACTGGGGGTGTGGCAGAGACCTTCACGACCAAAGAGGCTCCATGGAATTTCT
ATGAGATTCTAGAGAAGGCCTTGACGAGGGGCTCGCTGGTGGGCTGCTCCATTGATATCAGAAATGCTGCAGAGTCTGAAGCCCGGACGCCTTTTGGCCTCATTAAGGGTCATGCCTACACAGTGACAGGGGTGGACCAGGTAAA
CATCCGAGGCCAGAAAGTCGAGCTCATCAGAGTCCGGAATCCTTGGGGCCAGGTCGAGTGGACCGGGTCCTGGAGCGACAGCTCCTCTGAGTGGCGCTCCGTTGGCCCAGCTGAGCAGAAGCGCCTGTGTCACACGGCTCTGGAC
GACGGGGAGTTTTGGATGGCATTCAGGGACTTCCGGGCCCACTTTGACAAGGTGGAGGTCTGCAATCTCACCCCTGATGCCCTGGAGGAGGATGCTGTCCACAAGTGGGAGGTGACAGTCCATCAGGGAAGCTGGGTGCGGGGCT
CCACGGCTGGCGGCTGCCGCAACTTCCTGGACACCTTTTGGACCAACCCACAAATAAAACTGTCCCTGGAGGGGCAGGAGGAATGCACTTTCCTTGTAGCCCTGATGCAGAAAGATCGAAGGAAGCTCAAGAGGTTTGGTGCCAA
TGTGTTGACCATCGGCTATGCCATTTACCAGTGTCCTGGCCAAGAAGGACACCTGAATAAGGATTTCTTCAGATACCACGCCTCACAGGCCAGAAGCAAAACCTTCATCAACCTGCGAGAAGTCTCCGACCGGTTCAGGCTGCCC
CCCGGGGACTACATCCTGATTCCCAGCACCTTCGAGCCCCATCAGGAAGCTGATTTCTGCCTAAGGATCTTCTCAGAGAAAAAAGCCATTACCCGGGACATGGATGGAAGTGTAGACATCGACCTTCCTGAGCCTCCAAAGCCCA
CAGCCGAGACGGAGGAGGAACGGCAGTTCCAGGCCCTGTTTAAACAAGTTGCCGGTGAGGACATGGAAGTGACAGCTGAGGAGCTCAAATATGTTTTAAATGCTGTGCTGCAAAAGAAAAAAGACATCAAATTCAAGGAGCTCAG
CCTGATTTCCTGTAAAAACATCATTTCTCTAATGGACACCAGCGGCAATGGGAAGCTAGAGTTTAGTGAATTCAAAGTGTTCTGGGACAAGCTGAAGAAGTGGATGAGCCTTTTCCTACAGTTCGATGCTGACAAATCGGGCACC
ATGTCCTCCTACGAGCTGCGGAGCGCCCTGAAAGCCGCAGGCTTCCAGCTCAGCAGCCCCCTCCTGCAGCTGATTGTCCTCAGATATGCGGACCCGGAGCTCCAGCTGGGCTTCGACGACTTCCTCAACTGCCTGGTCCGGCTGG
AGAATGCGAGCCGGATGTTCCAGGCTCTCAGTACGAAGAACATTCATCTCAACATAAACGAGTTCATCGTCTTGACAATGAACATT???????????????????????????????????????????????????????????
?

ACTCATGGCCAGAGCTTCGAGCAGCTGAGGCAGGAGAGCTTGCAGAAGGGCATCCTGTTTGAGGATGCAGACTTCCCAGCCAACAGCTCCTCTCTCTTCTACAGCGAGAGGCCCCAGATCCCCTTCATATGGAAACGACCAGGGG
AAATAGTGAAAAACCCAGAATTCATCCTTGGAGGGGCCACCAGGACTGATATCTGCCAGGGGGAGCTTGGCGACTGCTGGCTATTAGCCGCCATTGCCTCCCTTACGCTGAATGAGAAAGCGCTGGCCAGAGTCGTCCCCCAGGA
CCAAGGCTTCGGCCCCGGTTACGCTGGCATATTCCATTTCCAGTTCTGGCAGCACAGTGAATGGCTGGACGTGGTGATCGATGACCGACTGCCCACCTTCAGGGACCGCCTGATCTTCCTCCACTCGGCCGACCACAATGAGTTC
TGGAGTGCCCTGCTGGAAAAAGCGTACGCCAAGCTGAACGGGAGCTATGAGGCTCTGAAGGGGGGCAACGCCATTGAGGCCATGGAAGACTTCACTGGGGGCGTAGCAGAGACCTTCGAAACTAAACAGGCTCCAGAGAACTTCT
ATGAGATTCTAGAGAAGGCCTTGAAGAGGGGCTCTCTGGTGGGCTGCTCCATTGATATCAGAAATGCTGCAGAATCTGAAGCCCGGACACCTTTTGGTCTTATTAAGGGTCATGCCTACACTGTGACAGGAATTGACCAGGTAAA
CTGCCGAGGCCAGGAAATCGAGCTCATCAGAGTCCGGAACCCTTGGGGCAAGGTTGAGTGGAATGGGTCCTGGAGCGACAGTTCTTCTGAATGGCGTTTCGTCACCCCAGCTGAGCAAAGGCGCCTGTGTCACACTGCCCTCGAC
GACGGGGAATTCTGGATGGCATTTAGTGACTTCAAGGCCCATTTTGACAAAGTAGAGATCTGCAACCTCACTCCTGATGCCCTGGAGGTGGATGCTCTCCACAAATGGGAGGTGACAGTCCATCAAGGAAGCTGGGTGCGGGGCT
CCACAGCTGGGGGTTGCCGCAATTTCCTGGATACCTTTTGGACCAATCCACAAATAAAACTGTCCCTGGAGGGGCAGGAGGAGTGCACTTTCCTTGTAGCCCTGATGCAGAAAGATCGAAGGAAACTCAAGAGGTTTGGTGCCGA
CGTGCTGACCATCGGCTATGCCATTTACCAGTGCCCCAGCAAAGATGAACACCTGAACAAGGACTTCTTCAGATACCATGCTTCCCAGGCCAGAAGCAAAACCTTCATCAACCTGAGAGAAGTCTCAGACAGGTTCAAGCTGCCC
CCTGGGGAATACATCCTGATTCCCAGCACTTTTGAGCCCCATCAGGAAGCCGATTTCTGCCTGAGGATCTTTTCAGAGAAGAAAGCCATTACCCAGGACATGGATGGAAGTGTCGACATTGACCTTCCTGAGCCTCCAAAGCCGA
CTCCAGAGACAGAGGAGGAACAGCAGTTCCAGGCCCTGTTTGAGCAAGTGGCTGGTGAGGACATGGAGGTGACAGCAGAGAAACTTGAATATGTTTTAAATGCTGTGCTGCAAAAGAAAAAGGACATCAAATTCAAGAAACTCAG
CCTGATTTCCTGTAAAAACATCATTTCTCTAATGGATACCAGTGGCAACGGGAAGCTGGAGTTCAGTGAATTCAAAGTGTTCTGGGACAAACTGAAGAAGTGGACAAACCTTTTCCTTCAGTTCGACGCTGACAAATCCGGCACC
ATGTCCTCCTACGAGCTGCGGACTGCCCTGAGAGCCGCAGGCTTCCAGCTGAGCAGTCACCTCTTGCAGCTGATCGTCCTCAGGTACGCGGATGAGGAGCTCCAACTGGGCTTTGATGACTTCCTCAACTGCATGGTCCGGCTGG
AGAACGCGAGCCGGGTGTTCCAGGCCCTCAGCACCAAGAACATTCATCTCAACATAAACGAGTTCATCGTCTTGACAATGAACATT???????????????????????????????????????????????????????????
?

ACCCACGGCCAGAGCTTTGAGCAAATGAGGCAGGAGTGCCTGCAGAGAGGCACCCTGTTTGAGGATGCAGACTTCCCAGCCAGCAATTCCTCCCTGTTCTACAGTGAGAGGCCGCAGATCCCCTTTGTGTGGAAACGACCAGGGG
AAATCGTGAAAAACCCAGAATTCATTCTTGGAGGGGCCACCAGGACTGATATCTGCCAGGGAGAGCTGGGAGACTGCTGGCTATTAGCCGCCATCGCCTCCCTTACGCTTAATCAAAAAGCACTGGCCAGAGTCATCCCCCAGGA
CCAAAGCTTTGGCCCTGGTTATGCCGGGATATTCCATTTCCAGTTCTGGCAGCACAGTGAGTGGCTGGACGTGGTGATCGATGACCGCCTGCCCACCTTCAGGGACCGCTTGGTTTTCCTCCACTCTGCCGACCACAACGAGTTC
TGGAGCGCCTTGCTGGAAAAAGCCTACGCCAAGCTAAATGGGAGCTATGAAGCTCTGAAGGGAGGCAGCGCCATCGAGGCCATGGAAGACTTCACTGGGGGTGTGGCAGAGACCTTCCAAACTAAAGAGGCCCCCGAGAACTTCT
ATGAGATTCTAGAGAAGGCTTTGAAGAGAGGCTCCCTGCTGGGCTGCTTCATTGATACCAGAAGTGCTGCAGAATCTGAGGCCCGGACGCCGTTTGGTCTTATTAAGGGTCATGCCTACAGTGTAACGGGAATTGACCAGGTAAG
CTTCCGAGGCCAGAGAATCGAGCTCATCCGAATCCGGAACCCTTGGGGCCAGGTTGAGTGGAACGGGTCGTGGAGCGACAGTTCTCCGGAGTGGCGTTCTGTTGGTCCAGCTGAGCAGAAGCGTCTGTGTCACACTGCTCTGGAT
GATGGGGAATTCTGGATGGCATTTAAGGACTTCAAGGCCCACTTTGATAAAGTGGAGATCTGCAACCTCACTCCCGATGCCCTGGAGGAAGACGCGATCCACAAATGGGAGGTGACGGTCCATCAGGGAAGCTGGGTTCGCGGCT
CCACGGCTGGGGGCTGCCGCAATTTCCTGGATACCTTTTGGACCAATCCACAAATAAAATTGTCTCTGGAGGGGCAGGAGGAGTGTAGTTTCCTTGTAGCCCTGATGCAGAAAGATAGAAGGAAACTCAAGAGATTTGGTGCCAA
TGTGCTGACAATCGGCTATGCCATTTATGAGTGCCCTGACAAAGACGAACACCTGAACAAAGACTTCTTCAGATACCACGCTTCTCGGGCCAGAAGCAAGACGTTCATCAACCTGAGAGAAGTCTCCGACCGGTTCAAGCTGCCC
CCTGGGGAGTACATCCTGATTCCCAGCACTTTTGAGCCCCACCAGGAAGCTGATTTCTGTCTGAGAATCTTTTCAGAGAAAAAAGCCATTACCCGGGATATGGATGGAAATGTAGACATTGACCTTCCTGAGCCTCCAAAGCCAA
CTCCAGAGACAGAGGAGGAGCAGCGGTTTCGGGCTCTGTTTGAACAAGTCGCTGGTGAGGACATGGAGGTGACAGCAGAGGAACTTGAGTATGTTTTAAATGCTGTGCTGCAAAAGAAAAAGGACATCAAATTCAAGAAGCTAAG
CCTGATCTCCTGTAAAAACATCATTTCCCTGATGGACACCAGCGGCAATGGGAAGCTGGAGTTTGATGAATTCAAAGTGTTCTGGGACAAGCTGAAGCAGTGGATTAACCTTTTCCTTCGGTTTGATGCTGACAAGTCCGGCACC
ATGTCTACCTATGAACTACGGACTGCACTGAAAGCTGCAGGCTTTCAGCTGAGCAGCCACCTCCTGCAGCTGATTGTGCTCAGGTATGCGGATGAGGAGCTCCAGCTGGACTTCGATGACTTCCTCAACTGCCTGGTCCGGCTGG
AGAATGCGAGCCGGGTGTTCCAGGCTCTCAGTACAAAGAACATTCATCTCAATATAAATGAGTTCATCCATTTGACAATGAACATC?????????????????????????????????????????????????????????????
?

ACCCACGGCCAGAGCTTCGAGCAGCTGAGGCAGGAGTGTGTGCAGAAGGGCATCCTGTTTGAGGATGCAGACTTCCCAGCCGACAGCTCCTCGCTCTTCTACAGTGAGAGGCCCCAGATCCCCTTCGTGTGGAAACGGCCAGGGG
AAATAGTGGAAAACCCAGAATTCATCCTTGGAGGGGCCACCAGGACAGATATCTGCCAGGGGGAGCTTGGCGACTGCTGGCTATTAGCTGCCATCGCCTCCCTGACGCTGAATGAAAAAGCTCTGGTTCGAGTCGTCCCCCAGGA
CCAAAGCTTTGGACCCGGTTATGCCGGGATATTCCACTTCCAGTTCTGGCAGCACAGCGAATGGCTGGATGTGGTCATCGATGACCGACTGCCCACCTTCAGGGACCGCCTGATCTTCCTCCACTCGGCCAACCACAGAGAGTTC
TGGAGTGCCCTGCTGGAAAAAGCCTATGCCAAGCTGAGCGGTAGCTACGAGGCTCTGAAGGGAGGCAGCGCCATCGAGGCCATGGAAGACTTCACCGGGGGTGTAGCAGAGACCTTTGCAACTAAAGAGGCTCCAGAGAACTTCT
ATGAGATTCTGGAGAAGGCCTTGAAGAGGGGCTCTCTCGTGGGCTGCTCCATTGATATCCGAAATGCTACAGAGTCTGAAGCCCGAACACCTTTTGGTCTCATTAAGGGCCATGCCTACAGTGTGACAGGAATTGACCAGGTAAA
CTTCCAAGGCCGGAACGTGGAGCTCATCAGAGTCCGGAACCCTTGGGGCCAGGTTGAGTGGAACGGGTCCTGGAGTGACAGTTCCTCCGAGTGGCTTTCGGTCGGGCCAGCTGAGCAGAAGCGCCTGTGTCACACCTCTCTCGAC
GACGGGGAATTCTGGATGGCGTTTAGGGACTTCAAGACCCACTTTGACAAGGTAGAAATCTGCAACCTGACTCCCGACGCCCTGGAGGAGGATGCTGTCCACAAATGGGAGGTGACGGTCCATCAAGGAAGCTGGGTGCGGGGCT
CCACCGCCGGGGGCTGCCGCAATTTTCTAGATACCTTCTGGACCAATCCACAGATAAAACTGTCCTTGGAGGGGCAGGAGGACTGCACTTTCCTTGTAGCCCTGATGCAGAAAGATCGAAGGAAACTCAAGAGGTTTGGTGCCGA
CATGCTGACCATTGGCTATGCCATTTACCAGAGCCCCCGCAAAGACGAACACCTGAACAAAGACTTCTTCAGGTATCATGCTTCCCAGGCCAGAAGCAAAACGTTCATCAACCTGAGAGAAGTCTCCGACCGCTTCCAACTGCCC
CCTGGGGAGTATATCCTGATACCCAGCACTTTTGAGCCCCATCAGGAAGCTGATTTCTGCCTGAGGATCTTTACAGAGAAGAAAATTATCACCCAGGACATGGATGGAGATGTGAACATTGACCTTCCCGAGCCTCCGAAGCCGA
CTCCAGAGACAGAGGAGCACCTGCAGTTCCGGGCCCTGTTTGAACAAGTTGCCGGCGAGGACATGGAGGTAACTGCAGAGAAACTGGAATATGTTTTAAACGCTGTGCTGCAAAAGAAAAAGGACGTCAAATTCAAGAAGCTCAG
CCTGATTTCCTGTAAAAACATCATTTCTCTAATGGACACCAGCGGCAGCGGGAAGCTGGGGTTCGCGGAGTTCAAAATGTTCTGGGACAAGCTGCAGAAGTGGACAAACCTGTTCCTTCAGTTCGATGCTGACAAGTCTGGCACT
ATGTCCTCCTATGAGCTGCGGACCGCCCTGAAAGCCACAGGCTTCCAGCTGAGCGGTCGCCTCCTGCAGCTGATTGTCCTCAGGTACACAGACGAGAACCTCCAGCTCGGCTTTGATGACTTCCTCAACTGCCTGGTCCGGCTGG
AGAACGCGAGCCGGGTGTTCCACGCGCTCAGTACAAAGAACATTCATCTCAACATAAACGAGTTCATCAAGCTGACAATGAACATC?????????????????????????????????????????????????????????????
?

ATCCACGGTCAGAGCTTTGAGCAGCTGAGGCAGGGCTGCCTGCAGTCTGGCACCTTGTTTGAGGATGCTGACTTCCCTGCCAGCAATGTCTCCCTGTTCTACAGCGAGAGGCCCCAGGTCCCCTTTGTGTGGAAACGGCCAGGGG
AAATTGTGGAAAAACCAGAATTCATTCTTGGAGGGGCCACCAGGACCGACATTTGCCAAGGGGAGCTGGGAGACTGCTGGCTCCTGGCGGCCATTGCCTCCCTAACCCTCAATCAGAAAGCACTGACCAGGGTGGTTCCCCAGGA
CCAAGGATTTGGTTCTGGCTATGCCGGGATATTTCATTTCCAGTTCTGGCAGCACAGTGAGTGGCTGGACGTAGTGATTGATGACCGCCTGCCCACCTTCAAGGACCGCCTGGTCTTCCTCCACTCTGCTGATCACAATGAGTTC
TGGAGCGCACTGCTGGAGAAAGCCTATGCCAAGCTTAACGGGAGTTATGAGGCACTGAAGGGAGGCAGTGCCATTGAAGCCATGGAAGACTTCACTGGGGGTGTGGCTGAGAACTTCCAAATCCGAGAGGCACCAGAGGATTTCT
TTGAGATTCTGGAGAAGGCTTTGAAGCGTGGTTCCTTGCTGGGATGCTCCATAGATACCCTGAATGCCTCAGAATCTGAAGCTCGAACATCTCTTGGTCTCATTAAGGGCCATGCCTACACCGTGACGGGGCTCGATCAGGTAAA
CTTCCATGGCCAGAGAATCAAGCTCATCAGAGTCCGTAACCCTTGGGGCCAGGTGGAGTGGAACGGGCCATGGAGTGACAGCTCTCCTGAGTGGCGCTCTGTGGACCTGGAGGAGCAGAAGCGCCTGGGCCACACTGCCCTTGAT
GACGGGGAGTTCTGGATGGCATTCAAAGACTTCAAGATCCATTTTGACAAAGTGGAGATCTGCAACCTCACACCTGATGCCCTGGAGGACAGCGCCCTCCACAGGTGGGAGGTGACCATCCACCAAGGAAGCTGGGTCCGTGGCT
CCACTGCTGGTGGCTGTCGCAACTTCCTGGACACCTTCTGGACCAACCCTCAGATAAAGCTGTCCCTGGAGGGCCAGGAGGGCTGCACTTTCCTAGCAGCCCTTGATGCAGAAAGACCGCAGGAGGCTCAAGAGGTTTGGCGCCAA
CATGCTGACCATTGGCTACGCCATTTACCAGTGCCCAGACAAGGACGGACACCTGAGTAGGGACTTTTTCAGGTACCATGCCTCCCTGGCCCGAAGCAAGACGTTCATCAACTTGAGAGAAGTCTCAGAGCGATTCCAGCTGCCC
CCGGGAGACTATATCCTCATCCCCAGCACCTTTGAGCCGCATCAGGAGGCCGACTTCTGCCTGAGAATCTTCTCTGAGAAGAGAGCTGTGACTCGGGACCTGGACGAGAACATAGACATTGACCTTCCTGAGCTTCCAAAGCCGA

CTCCAGAAACAGAAGAGGAACAGCAGTTCCGGGCCCTGTTCCAGCGAGTTGCTGGCGAGGACATGGAGGTGTCAGCTGAGGAGCTTGAATATGTTCTAAATGCTGTTCTGCAGAAGAAAACAGCCCTCAAGTTCAAGAGGCTGAG
CCTGCTGTCCTGCAGAAACATCATCTCTCTGATGGATACCAGTGGCAACGGGAAGCTGGAGTTTGAAGAGTTCCGGGTCTTCTGGGACAAGCTGAAACACTGGATGGACCTGTTTCTCCAGTTCGATGTGGACAAGTCTGGCACC
ATGTCTTCCTATGAGCTGCGGACGGCACTGAAAGCTGCAGGCTTTCAGCTGGGTGGCCATCTCCTGCAGCTGATTGTCCTCAGGTATGCAGATGAGGACCTCCAGCTGGATTTCGATGACTACCTCAACTGCCTGGTGCGGCTGG
AGAATGCAAGCCGGGTGTTCCAATCTCTCAGTGTGAAGAACATCCATCTCAACATAAACGAGTTCATCAGCCTGACCATGAACATC????????????????????????????????????????????????????????????????
?

ATCCACGGCCAGACCTATGAACAACTGAGGCAGGACTGCCTACAGAGGGGCATCCTTTTTGAAGATGGTGACTTTCCAGCCAACAATTCCTCCCTCTTCTTCAGTGAGAGGCCCTCCATACCTTTTGTGTGGAAACGTCCAGGGG
AAATAGTAAAGAATCCAGAATTTATTCTTGGAGGAGCCACAAGAACTGATATTTGTCAAGGGGAACTTGGAGACTGTTGGCTTTTAGCAGCCATTGCCTCTCTCACACTTAATGAAAAGACCCTGGCCAGAGTTGTGCCCCAAGA
TCAAAATTTTGGATCTGGTTATGCTGGGATATTTCACTTTCAGATCTGGCAGCACAGCGAATGGTTGGATGTGGTCATTGATGACCGACTCCCGACCTTCCGAGATCGTTTGGTTTTCCTCCACTCTGCTGATCACAATGAATTC
TGGAGTGCCTTGCTGGAAAAGGCATATGCCAAACTGAATGGAAGCTATGAAGCCTTGAAGGGTGGAAGCACAATTGAAGCTATGGAAGATTTCACTGGAGGAGTGGCAGAGACTTTTGAAGTTAAAAAGGCCCCGGAAAACTTCT
ATGAGATCCTCGAGAAAGCCTTGAAGAGAAGCTCTATGGTGGGCTGCTCCATTGATATCAGCAACTCTTCAGAGTCAGAAGCACGGACACCTTTTGGTCTCATTAAGGGTCACGCATACACTTTGACGGGAATTGACCAGGTGAA
TTATCGAGGTCAAAAGGTTGAACTCATCAGAGTCCGGAATCCTTGGGGCCAGGTAGAATGGAATGGTCCTTGGAGTGACAATTCTGGTGAGTGGAGTTCTGTTGAGTCTTCTGAACAAAAACGCCTATGCCATTCTGCTCTTGAT
GATGGAGAATTCTGGATGACTTTTAAGGATTTTAAAATCCACTTTGACAAAGTAGAGATCTGCAATCTCACTCCTGATGCTCTGGAGGAAGATGCCCTTCACAAATGGGAAGTGACAATCCATCAAGGGAGCTGGGTCCGGGGAT
CTACTGCTGGAGGATGCCGTAATTTCCTGGACACCTTCTGGACCAATCCCCAGATCAAATTGTCTCTGGATGGGCAAGAGGAATGTACTTTTATTGTAGCCCTCATGCAAAAGGACAGAAGGAAACTCAAGAAACTTGGAGCTGA
TTTGCTGACTATTGGATATGCTATTTACCAGAGTCCTGCCAAAGATGAGCATCTCACCAAGGATTTTTTCAGATATCATGCTTCACAGGCCAGAAGCAAAACATATATTAACTTAAGAGAAGTTTCTGAGAGATTTAAGCTGCCC
CCTGGGGAATATATCCTGATTCCTACAACTTTTGAGCCACACCAAGAAGCCAATTTCTGCCTTCGAATCTTTTCAGAGAAGAAAGCCATTACTCAGGAAATGGATGAAAATGTGGATATTGCTCTCCCAGAACCGTCTAAACCAA
CTTCAGAAAGCATGGAAGATAAACAGTTTCGGACACTGTTTGAACAGATTTCAGGAGAGGACATGGAGGTAACAGCAGAGGAACTGGAATATGTTTTGAATACTGTGCTACAAAAAAAAAAGAATATTAAATTCAAGAAGCTAAG
TCTCATTTCCTGCAAAAACATCATTTCTCTAATGGATAACAATGGCAATGGAAAACTGGAGTTCAATGAATTCAAAGTTTTCTGGGACAAACTGAAGACGTGGATAAATATCTTCCTTCAATTCGATGCTGACCGATCTGGCACC
ATGTCCTCCTATGAGCTGCGGCTAGCCTTGAAAGCTGCAGGCTTCCAGCTGAACAATTACCTTTTGCAGCTAATTGTCCTAAGGTATGCTGATGAGCAGTATCAACTTGACTTTGATGATTTCCTGAACTGTATGATCCGGTTAG
AGAATGCGAGCCGGGTGTTCCAGATTCTCAGTGCAAAGAACATTCAGCTCAATATAAATGAGTTCATCAGTTTGGCGATGAATATT????????????????????????????????????????????????????????????????
?

ATCCATGGCAAGACCTATGAGGAGCTGCGACAGGAATGCCTACAGAGGGGCATCCTGTTTGAAGATAGGGACTTTCCAGCCAATGACTCCTCCCTCTTCTTCAGCGACAGACCCTCCATTCCTTTTGTGTGGAAACGTCCAGGGG
AAATAGTTAAAAACCCAGAATTCATTCTTGGAGGAGCCACAAGAACTGATATTTGTCAAGGGGATCTTGGAGACTGTTGGCTCTTAGCAGCCATTGCCTCTCTTACACTTAATGAAAAGACTTTGGCTAGAGTTGTGCCCCCTGA
TCAAAATTTTGGCCCTGGTTATGCTGGGATATTTCACTTTCAGATCTGGCAGCATGGCGAGTGGTTGGATGTGGTCATTGACGACCGGCTCCCGACCTTCAGAGATCGTTTGGTTTTCCTTCACTCTGCTGATCACAATGAATTT
TGGAGTGCATTGCTGGAAAAGGCATATGCCAAATTGAGTGGAAGCTATGAAGCCCTGAAGGGTGGAAGCACAATTGAAGCTATGGAAGACTTCACTGGAGGAGTAGCAGAGACTTTTATCATTAAAGCAGCCCCAAAAAACTTAT
ATGAGATCCTAGAAAAGGCTGTGAAGAGAGGCTCTCTGGTGGGCTGCTTCATAGAAGTCAAAGATTCTTCAGAGACAGAAGCAAAAACACCTTTTGGCCTCATTAAGGGTCACGCATACACAGTGACTGGAATTAACCAGGTGAA
TTACAAAGGCCAAAAGGTTGAGCTCATCAGAGTCCGGAATCCTTGGGGCCAGGTAGAATGGAATGGTCCTTGGAGTGACAATTCTCCTGAGTGGAATTTTGTCAACTCATCTGAGCAAAAACGCCTCTGCCATTCTGCTCTTGAT
GATGGAGAATTCTGGATGACTTTCAAGGATTTCAAAGTCCACTTTGACAAAGTAGAGATCTGCAACCTCACACCTGATGCCCTGGAGGAAGACACCCTTCACAAATGGGAAGTAACAATTTATCAAGGGAGTTGGGTCCGAGGAT
CCACTGCAGGAGGATGCCGTAATTATATTGACACCTTCTGGACCAACCCCCAGATCAAATTATCTCTGGATGGCCAAGAAGAATGCACTTTTATTGTTGCCCTTATGCAAAAGGACAGAAGGAAACTCAAGAAATTTGGAGCCAA
TTTGCTGACAATTGGATATGCTATTTACCAGAGCCCTGCCGAAAATGAACACCTCAACAAGGATTTTTTAAATTTCATAGTTCTCAGGCCAGGAGCAAAGAATACATTAACCTAAGAGAAGTCTCTAACAGATTCAAGCTGCCC
CCTGGAGAATATATCTTGATTCCTTCAACTTTTGAACCACATGAGGAAGCCAATTTCTGCCTTCGAATCTTTTCAGAGAAGAAGACTAAATACCAGGATATGGATGAAACAGTGGATATTGCCCTGCCAGAGCCATCTAAACCAA
CTTCAGAAACCAAGGAAGATAAAGAATTGCGGTCACTGTTTGAACAGGTTTCAGGAGAGGACATGGAAGTGACAGCAGAGGAACTGAGGCATGTTTTGAATACCATGCTGCAAAAAGAAAAGAATATTAAATTCAAGAAGCTAAG
CCTCATTTCCTGCAAAAACATCATTTCTCTAATGGATACCAATGGCAGTGGGAAAATGGAGTTCAATGAATTCAAAATGTTCTGGATCAGGCTGAAGCAGTGGATAAATATCTTCCTTCAGTTTGATGCTGACAAATCTGGCACT
ATGTCCTCCTACGAGCTGCGGTTAGCCTTGAAAGCTGCAGGCTTCCAGCTAGGCAATTACCTTTTGCAGTTAATTGTCCTAAGATATGCTGATGAGCTATACCAACTTGATTTTGATGATTTCCTCAACTGCATGATCCGGTTAG
AGAATGCAACCCGGGTGTTCCAGGTTCTCAAAGCAAAGGACATTCATCTCAATATAAATGAGTTCATCTGTTTAGCGATGAATATT????????????????????????????????????????????????????????????????
?

 ------
GGCCGGACCTTTGAGGAACTGCGGGACCGGTGCCTGCGGCGGGGGGTCCTGTTCGAGGACCCCGACTTCCCCGCCGACGATTCCTCGCTTTTCTTCAGCGAGATGCCCCCCATTCCATTTGCGTGGAAGCGTCCTGGGGAAATAG
CGAGAAACCCAGAGTTCGTGTCCGACGGCGCCACCAGGACCGACATCTGCCAGGGCGAACTCGGAGACTGCTGGCTCCTGGCAGCCATCGCTTCCCTCACCCTGAACCCCAGGGTCCTGGCCAGAGTCGTGCCACCCCAGCAGAG

TTTCGGCCCGGACTACGCAGGGATATTTCACTTCCAGCTTTGGCAGCACGGGCAGTGGCTGGACGTGGTCATTGACGATAGACTCCCGACCTTCAAGGACCGTTTGGTTTTCCTCCACTCGGCCGATCACAACGAGTTTTGGAGC
GCCTTGCTGGAGAAGGCTTATGTCAAGCTGAACGGCAGCTATGAAGCACTGAAGGGGGGCAGCACGATCGAAGCCATGGAGGATTTCACCGGCGGGGTCGCCGAGACCTTGGACGTGAAGGCGATCTCACAAGGCCTGTCCGAGA
TCCTGAGGAAGGCCCTGGACAGGGGCTCTCTGGTCGCCTGTTCCATCGAGGCCCGCAACGCCTCCGACTCGGAAGCTCGGACACCTTTCGGCCTCGTGAAGGGCCATGCGTACTCCGTGACCGGCACTGACCAGGTCACTTTTAG
GGGCCGGAGGGTCGAGCTGGTCCGAGTCCGGAACCCGTGGGGGCAAGTGGAGTGGAACGGGCCGTGGAGCGACGACTCTTCTGAATGGCGCTCGGTTGATCCGTCTGAGAAGAAACGCCTGTACCACACGGCTCTGGACGACGGG
GAGTTCTGGATGCCATTTGCGGATTTCGAAGCCCACTTCGACAAAGTGGAGATCTGTAACCTCACCCCTGATGCACTGGAGGGCGGGACGCTCCTACAATGGGAGGTGGCTGTACACCACGGCAGCTGGGTCAAGGGGGCCACTG
CCGGAGGCTGCCGGAATTTCCTAGACACTTTCTGGACCAACCCCCAGATCAAGCTGTCTCTGGGAGGGAAAGAAGACTGCACCTTGGTGGTCGCTCTGATGCAAAAGAATCGACGTAAACTCAAGAAATTTGGAGCGAATATGCT
GACCATCGGCTATGCCATTTACCAGTGTCCTGGTCAAGATGGGCACCTAGGCAGAGATTTCTTCCGGTGCCACGCATCTCAGGCCCGGAGCAAAACCTACGTTAACCTGCGGGAAGTGTCGGGCCGGTTCAAGCTGCCCCCCGGG
GAGTACGTCCTTGTTCCGTCCACCTTCGAGCCCCACCAGGAGGCGGATTTCTGCCTGCGCATCTTTTCTGAGAAAGCGGCCGTCACCCAGGACCTGGATGGCCAAGTGAATGTCGAGCTTCCAGAGCCACTGAAACCGACTTCCG
AAACGGAGGAGGAGAGGCAGTTCCGGATTCTATTTGAGAAGGTGGCCGACAAGGACCTCCAGATCTCAGCAGAGGAGCTGCAGGAGATTCTGAATGCGGTGCTCCGAAACACCAAGAATATCCAGTTCAAGAAGCTGAGCCTTGT
TTCCTGTCGAAATATAATTTCTCTCATGGACACCAACGGCAGCGGGAAGCTGGAACTGGATGAGTTCAAGGCGTTCTGGGCCCGGCTGAAGGCCTGGACTAACATCTTCTTCCAGTTTGACGGTGACAAGTCAGGGACCATGTCA
TCCTATGAGCTGCGGGGTGCACTGAAGGCGGCCGGATTCCAGTTAAACAACAACCTCCTGCAGCTGCTTGTCCTCCGCTATGCTGATGAGCGGCTCCAGGTCGACTTCGACGACTTCCTCAACTGCCTAGTTCGCCTGGAAAACA
CAAGCCGGGTGTTCCAGGCTCTCAAGGCCACAGACCTCAGTCTCAGTGTGGGCGAGTTTATCCTCTTGGCCATGAACATC??????????????????????????????????????????????????????????????

ACGCAGGGCAAGTCCTTCGAGGAGCTGAGGCACGAGTGCCTGCAGAAGGGGGTTCTGTTCGAGGACCAGGACTTCCCCGCCGCCGACTCCTCGCTGTACTTCAGCGAGAGCGTCCCGGTCAACATCGAATGGAAGAGGCCCAAGG
AGATCTGCAAAGACCCAAAGTTCATCGTGGGCGGCGCAGACAGGACGGACATCTGCCAAGGACAGCTCGGTGACTGCTGGCTACTGGCGGCCATCGCGTCCTTGACCCTCAAAAAAGAAGCCCTGGCCCGGGTCGTCCCCCCCGA
CCAGGACTTCGACCGCAGATACGCCGGCATCTTCCACTTCCAGTTCTGGAGTCACAACCGGTGGCTGGACGTGGTGGTGGACGACCGGCTGCCGGTGGTCCGAAACAAGCTCATCATGCTGCACTCCGCCTCCAACGACGAGTTC
TGGAGCGCCCTGCTGGAGAAGGCCTACGCCAAACTGCACGGCAGCTACGAGTCCCTGAAGGGCGGCAGCACCATGGAGGCCATGGAGGATTTCACCGGCGGGGTCGGGGAAAATTACGAGACCAAGAACTGCCCGGACAACCTGT
TCTCCATCATGAAGAAGGCGCTGGACAGAGGCTCCATGATGGGATGCTCCATCGACATCACCAGCTCTGCCGAGTCCGAGGCCAAGACCACCACGGGCCTGGTGAAGGGTCACGCGTACTCCATCACGAGCCTGGAGGAGGTGAA
CTTCCGAGGTAAGACGGTCCAGCTGATCCGGGTGAGGAACCCGTGGGGTCAGGTGGAGTGGAACGGGCCCTGGAGTGACGGATCCAGAGAGTGGAATTACGTTGACAGCGGTGATAAAAATCGCCTCCTGCAGCTTTCA---
GACGACGGGGAATTCTGGATGGAGTTCAGCGACTTCAAGAAGAACTACGACAAGGTGGAGATCTGCAACATGACCCCCGACGACCTGACCGACGACTCCAAGCGCCAGTGGGCGGTCAGCATGTTCGAGGGGAACTGGATCCGCG
GCTCCACCGCCGGAGGCTGCAGGAACTTCATCGACACCTTCTGGACCAACCCGCAGTTCAAGCTGAAGCTGGACGACGACGACGTGTGCAGCGTGGTGATCGCGCTGATGCAGAAGAACAGGAGGAAGCTGAGGAAGGAGGGCAT
GGACATGGAGACCATCGGCTTCGCCGTGTACGAGGCTCCGGAGGACGAGGACCAACTGGGGAAAGACTTCTTCCGCTACAACGCGTCCAAAGCTCGCAGCAGGACCTACATCAACGTGCGGGAGATATCGGAGCGCTTCACGCTG
CCTCCCGGCAGCTACCTGCTGGTCCCCACCACCTTCCAGCCGCACCACGAGGCCGACTTCATCGTCAGGGTCTTCTCGGAGAAGAAGGCCGAAGCTCTCGAGATGGGGAGCAACGTTGACGCTGACCTCCCAGATCCGCCCCCCC
CCTCTGCCGAGTCCGACGAGGAGAAAGGCCTGAGGAGGCTGTTCGAACAGCTGGCCGGAGACGATTCTCGCGTCTCAGTATGCAAAATTATCGAGATTTTGAATAAAAAAAAGCACAGAAGAAAAGAGATCAAATTCGACGGCCT
GAGTCTGAGCACCTGCCACAGCATCATCAACCTGATGGATGTGGACAACACGGGGCAGCTGGAGTTCCAGGAGTTCAAGGTCTTCTGGGAAAAGATGAAGAAGTGGATCATGCTCTTCTTGGCCTTTGACACCGACCGCTCGGGG
AAGATGTCGTCCTACGAGCTCCGCAGCGCTCTCAAAGCTGCAGGAATGCAGCTGAACAACCGGCTGCTGCAGCTCATCGGCTTGAGGTTCGCTGACGACAAATACGACATCGACTTCGACGACTACCTCACCTGCATCGTCCGCC
TGGAGAACATGTTCATTATATATTTCCAAAGCAAAATAACCAAGGTAATCTTCTCTAAAGTTTTATGGATGGTTCTATTAATTCAATCT?????????????????????????????????????????????????????????
????

ACGCAGGGGAAATCCTTTGAGCAGCTGAGGCAGGAGTGTCTGCAGAAGGGCATCCTGTTTGAGGACCCAGATTTCCCTGCCACCGACTCCTCACTCTACTTTAGCCAAAGCGTTCCAGTGGCCATCGAGTGGAAGAGGCCCACGG
AGATCTGCTCCAACCCAAAGTTCATCCTAGACGATGCAGACAGGACGGACATTTGCCAAGGACAGCTTGGGGATTGCTGGCTCCTGGCAGCAATTGCATCCTTGACGCTCAAGAAGGATGCCCTGACCCGTGTCCTTCCCCACGA
CCAGGAGTTTGACCACAGATATGCTGGAATCTTTCATTTCCAGTTCTGGCAACACAATAAGTGGCTGGACATTGTTGTCGACGATCGTTTGCCCACAGTGAGAAATAAACTTATCATGCTTCACTCTGCCTCCAACAACGAGTTC
TGGAGCGCCTTGCTGGAGAAAGCTTATGCCAAGCTACAT

## (*i*) CAPN11

```
#NEXUS

BEGIN TAXA;
	DIMENSIONS NTAX = 37;
	TAXLABELS
		'Homo_Sapiens_ENSP00000381758'	'Gorilla_gorilla_gorilla_ENSGGOP00000019099'	'Macaca_mulatta_ENSMMUP00000016912'
'Oryctolagus_cuniculus_ENSOCUP00000015069'	'Ochotona_princeps_ENSOPRP00000015695'	'Otolemur_garnettii_ENSOGAP00000011397'
```

```
'Equus_caballus_ENSECAP00000004727'  'Sus_Scrofa_ENSSSCP00000001850'  'Bos_Taurus_ENSBTAP00000004690'  'Ailuropoda_melanoleuca_ENSAMEP00000012638'
'Mustela_putorius_furo_ENSMPUP00000012533'            'Myotis_lucifugus_ENSMLUP00000004763'            'Rattus_norvegicus_ENSRNOP00000059934'
'Mus_musculus_ENSMUSP00000113132/1-2142'            'Cavia_porcellus_ENSCPOP00000017159'            'Ornithorhynchus_anatinus_ENSOANP00000015579'
'Anolis_carolinensis_ENSACAP00000001318'            'Pelodiscus_sinensis_ENSPSIP00000019228'            'Meleagris_gallopavo_ENSMGAP00000011308'
'Gallus_gallus_ENSGALP00000016538'   'Taeniopygia_guttata_ENSTGUP00000008345'   'Westernclawfrog'   'AfricanclawedfrogNP'   'AfricanclawedfrogAA'
'Oreochromis_niloticus_ENSONIP00000009131'            'Gasterosteus_aculeatus_ENSGACP00000026345'            'Danio_rerio_ENSDARP00000046065'
'Danio_rerio_ENSDARP00000095870'            'Takifugu_rubripes_ENSTRUP00000021242'            'Tetraodon_nigroviridis_ENSTNIP00000019733'
'Oreochromis_niloticus_ENSONIP00000012531'            'Xiphophorus_maculatus_ENSXMAP00000006029'            'Oryzias_latipes_ENSORLP00000014711'
'Gasterosteus_aculeatus_ENSGACP00000006578'         'SmallerSpottedCatshark_TranscriptomeContig69801'         'bamboo_dataset1_plus_2_contig12303'
'ElephantShark_TranscriptomeContig20333' ;
END;

BEGIN CHARACTERS;
        DIMENSIONS NCHAR = 2127;
        FORMAT
                DATATYPE = DNA

                GAP=-
                MISSING=?
        ;

MATRIX
        'Homo_Sapiens_ENSP00000381758'                                                                                                  ------------------
ATGGTGGCTCACATAAACAACAGCCGGCTCAAGGCCAAGGGCGTGGGCCAGCACGACAACGCCCAGAACTTTGGTAACCAGAGCTTTGAGGAGCTGCGAGCAGCCTGTCTAAGAAAGGGGGAGCTCTTCGAGGACCCCTTATTCC
CTGCTGAACCCAGCTCACTGGGCTTCAAGGACCTGGGCCCCAACTCCAAAAATGTGCAGAACATCTCCTGGCAGCGGCCCAAGGATATCATAAACAACCCTCTATTCATCATGGATGGGATTTCTCCAACAGACATCTGCCAGGG
GATCCTCGGGGACTGCTGGCTGCTGGCTGCCATCGGCTCCCTTACCACCTGCCCCAAACTGCTATACCGCGTGGTGCCCAGAGGACAGAGCTTCAAGAAAAACTATGCTGGCATCTTCCATTTTCAGATTTGGCAGTTTGGACAG
TGGGTGAACGTGGTGGTAGATGACCGGCTGCCCACAAAGAATGACAAGCTGGTGTTTGTGCACTCAACCGAACGCAGTGAGTTCTGGAGTGCCCTGCTGGAGAAGGCGTATGCCAAGCTGAGTGGGTCCTATGAAGCATTGTCAG
GGGGCAGTACCATGGAGGGCCTTGAGGACTTCACAGGAGGCGTGGCCCAGAGCTTCCAACTCCAGAGGCCCCCTCAGAACCTGCTCAGGCTCCTTAGGAAGGCCGTGGAGCGATCCTCCCTCATGGGTTGCTCCATTGAAGTCAC
CAGTGATAGTGAACTGGAATCCATGACTGACAAGATGCTGGTGAGAGGGCACGCTTACTCTGTGACTGGCCTTCAGGATGTCCACTACAGAGGCAAAATGGAAACACTGATTCGGGTCCGGAATCCCTGGGGCCGGATTGAGTGG
AATGGAGCTTGGAGTGACAGTGCCAGGGAGTGGGAAGAGGTGGCCTCAGACATCCAGATGCAGCTGCTGCACAAGACGGAGGACGGGGAGTTCTGGATGTCCTACCAAGATTTCCTGAACAACTTCACGCTCCTGGAGATCTGCA
ACCTCACGCCTGATACACTCTCTGGGGACTACAAGAGCTACTGGCACACCACCTTCTACGAGGGCAGCTGGCGCAGAGGCAGCTCCGCAGGGGGCTGCAGGAACCACCCTGGCACGTTCTGGACCAACCCCCAGTTTAAGATCTC
TCTTCCTGAGGGGGATGACCCAGAGGATGACGCAGAGGGCAATGTTGTGGTCTGCACCTGCCTGGTGGCCCTAATGCAGAAGAACTGGCGGCATGCACGGCAGCAGGGAGCCCAGCTGCAGACCATTGGCTTTGTCCTCTACGCG
GTCCCAAAAGAGTTTCAGAACATTCAGGATGTCCACTTGAAGAAGGAATTCTTCACGAAGTATCAGGACCACGGCTTCTCAGAGATCTTCACCAACTCACGGGAGGTGAGCAGCCAACTCCGGCTGCCTCCGGGGGAATATATCA
TTATTCCCTCCACCTTTGAGCCACACAGAGATGCTGACTTCCTGCTTCGGGTCTTCACCGAGAAGCACAGCGAGTCATGGGAATTGGATGAAGTCAACTATGCTGAGCAACTCCAAGAGGAAAAGGTCTCTGAGGATGACATGGA
CCAGGACTTCCTACATTTGTTTAAGATAGTGGCA---
GGAGAGGGCAAGGAGATAGGGGTGTATGAGCTCCAGAGGCTGCTCAACAGGATGGCCATCAAATTCAAAAGCTTCAAGACCAAGGGCTTTGGCCTGGATGCTTGCCGCTGCATGATCAACCTCATGGATAAAGATGGCTCTGGCA
AGCTGGGGCTTCTAGAGTTCAAGATCCTGTGGAAAAAACTCAAGAAATGGATGGACATCTTCAGAGAGTGTGACCAGGACCATTCAGGCACCTTGAACTCCTATGAGATGCGCCTGGTTATTGAGAAAGCAGGCATCAAGCTGAA
CAACAAGGTAATGCAGGTCCTGGTGGCCAGGTATGCAGATGATGACCTGATCATAGACTTTGACAGCTTCATCAGCTGTTTCCTGAGGCTAAAGACCATGTTCACATTCTTTCTAACCATGGACCCCAAGAATACTGGCCATATT
TGCTTGAGCCTGGAACAGTGGCTGCAGATGACCATGTGGGGA
        'Gorilla_gorilla_gorilla_ENSGGOP00000019099'                                                                                    ------------------
ATGGTGGCTCACATAAACAACAGCCGGCTCAAAGCCAAGGGCGTGGGCCAGCACGACAACGCCCAGAACTTTGGTAACCAGAGCTTTGAGGAGCTGCGAGCAGCCTGTCTAAGAAAGGGGGAGCTCTTCGAGGACCCCTTATTCC
CTGCTGAACCCAGCTCACTGGGCTTCAAGGACCTGGGCCCCAACTCCAAAAATGTGCAGAACATCTCCTGGCAGCGGCCCAAGGATATCATAAGCGACCCTCTATTCATCATGGATGGGATTTCTCCAACAGACATCTGCCAGGG
GATCCTCGGGGACTGTTGGCTGCTGGCTGCCATCGGCTCCCTTACCAGCGTCCCCAAACTGCTGTACCGCGTGGTGCCCAGAGGACAGAGCTTCAAGAAAAACTATGCTGGCATCTTCCATTTTCAGATTTGGCAGTTTGGACAG
TGGGTGAACGTGGTGGTAGATGACCGGCTGCCCACAAAGAATGACAAGCTGGTGTTCGTGCACTCAACCAAACGCAGTGAGTTCTGGAGCGCCCTGCTGGAGAAGGCGTATGCCAAGCTGAGTGGGTCCTATGAAGCATTGTCAG
GGGGCAGTACCATGGAGGGCCTTGAGGACTTCACAGGAGGCGTGGCCCAGAGCTTCCAACTCCAGAGGCCCCCTCAGAACCTGCTCAGGCTCCTTAGGAAGGCCGTGGAGCGATCCTCCCTCATGACACGGACCCAGGGTGTCAC
CAGTGATAGTGAACTGGAATCCATGACTGACAAGATGCTGGTGAGAGGGCATGCTTACTCTGTGACTGGTCTTCAGGATGTCCACTACAGAGGCAAAATGGAAACACTGCTTCGGGTCCGGAATCCCTGGGGCTGGATTGGGTGG
AATGGAGCTTGGAGTGACAGTGCCAGAGAGTGGGAAGAGGTGGCCTCAGACATCCAGATGCAGCTGCTGCACAAGACAGAGGACAGGGAGTTCTGGATGTCCTACCAAGATTTCCTGAACAACTTCACGCTCCTGGAGATCTGCA
ACCTCACGCCTGATACACTCTCTGGGGACTACAAGAGCTACTGGCACACCACCTTCTACGAGGGCAGCTGGCGCAGAGGCAGCTCTGCAGGGGGCTGCAGGAACCACCCTGGCACGTTCCGGACCAACCCCCAGTTTAAGATCTC
TCTTCCTGAGGGGGATGACCCAGAGGATGACGCAGAGGGCAATGTTGTGGTCTGCACCTGCCTGGTGGCCCTAATGCAGAAGAACTGGTGGCATGCACGGCAGCAGGGAGCCCAGCTGCAGACCATTGGCTTTGTCCTCTACTCG
```

GTCCCAAAAGAGTTTCAGAACATTCAGGATGTCCACTTGAAGAAGGAATTCTTCACAAAGTATCAGGACCACGGCTTCTCAGAGATCTTCGCCAACTCACGGGAGGTGAGCAGCCAACTCCGGCTGCCTCCGGGGGAATATATCA
TTATTCCCTCCACCTTTGAGCCACACAGAGATGCTGACTTCCTGCTTCGGGTCTTCACCGAGAAGCACAGCGAGTCATGGGAATTGGATGAAGTCAACTATGCTGAGCAACTCCAAGAGGAAAAGGTCTCTGAGGATGACATGGA
CCAGGACTTCCTACATTTGTTTAAGATAGTGGCA---
GGAGAGGGCAAGGAGATAGGGGTGTATGAGCTCCAGAGGCTGCTCAACAGGATGGCCATCAAATTCAAAAGCTTCAAGACCAAGGGCTTTGGCCTGGATGCTTGCCGCTGCATGATCAACCTCATGGATAAAGATGGCTCTGGCA
AGCTGGGGCTTCTAGAGTTCAAGATCCTGTGGAAAAAACTCAAGAAATGGATGGACATCTTCAGAGAGTGTGACCAGGACCATTCAGGCACCTTGAACTCCTATGAGATGCGCCTGGCTATTGAGAAAGCAGGCATCAAGCTGAA
CAACAAGGTAATGCAGGTCCTGGTGGCCAGGTATGCAGATGATGACCTGATCATAGACTTTGACAGCTTCATCAGCTGTTTCCTGAGGCTAAAGACCATGTTCACATTCTTTCTAACCATGGACCCCAAGAATACTGGCCATATT
TGCTTGAACCTGGAACAGTGGCTGCAGATGACCATGTGGGGA
'Macaca_mulatta_ENSMMUP00000016912' ------------------
ATGGTGGCTCACATAAACTACAGCCGGCTCAAGGCCAGCGGCGTGGGCCAGCATCACAATGCCCACAACTTTGGTAACCAGAGCTTTGAGGAGCTGCGAGCAGTCTGTCTAAGAAAGGGGGAACTCTTTGAGGACCCCTTATTCC
CTGCTGAACCCAGCTCACTGGGCTTCAAGGACCTGGGTCCCAACTCCAAAAATGTGCAGAACATCTGCTGGCAGCGGCCCAAGGATATCATAAGCAACCCTCTGTTCATCATGGATGGGATTTCTCCAACAGACGTCTGCCAGGG
GATCCTCGGGGACTGCTGGCTGCTGGCTGCCATCGGCTCCCTTACCACCTGCCCCAAACTGCTGTACCGCGTGGTGCCCAGGGGGCAGAGCTTCAAGAAAAACTATGCTGGCATCTTCCATTTTCAGATTTGGCAGTTTGGGCAG
TGGGTGAACGTGGTAGTGGATGACCGGCTGCCCACAAAGAATGACAAGCTGGTGTTCGTGCACTCAACCGAACGCAGTGAGTTCTGGAGCGCCCTGCTGGAGAAGGCATATGCCAAGCTGAGTGGGTCCTATGAAGCACTGTCAG
GGGGCAGTACCATAGAGGGCTTTGAGGACTTCACGGGAGGCGTGGCCCAGAGCTTCCAACTCCAGAGGCCCCCTCAGAACCTGCTCAGGCTCCTTAGGAAGGCGGTGGAGCGATCCTCCCTCATGGGTTGCTCCATTGAAGTCAC
CAGCGACAGTGACCTGGAGTCCATGACCAACAAGATGCTGGTGAGAGGGCACGCTTACTCAGTGACTGGCCTTCAGGATGTCCACTACAGAGGCAAAATGGAAACACTGATTCGGGTCCGGAATCCCTGGGGCCGGATTGAGTGG
AATGGAGCTTGGAGTGACAGTCCCTTGGAGAGGAAGGAGGGAACTGAGGATGCAAGTGTG---
CTGCTGATGGGCTCCACCCCCACCCACCACAGGATGTCCTACCAAGATTTCCTGAACAACTTCACGCTCCTGGAGATCTGCAACCTCACGCCTGATGCACTCTCTGGGGACTACAAGAGCTACTGGCACACCACCTTCTATGAGG
GCAGCTGGCGCAGAGGCAGCTCCGCAGGGGGCTGCAGGAACCACCCTGGCACGTTCTGGACCAACCCCCAGTTTAAGATCTCTCTCCCTGAGGGAGATGACCCAGAGGATGACACAGAGAACGATGCTGTGGTCTGCACCTGCCT
GGTGGCCCTGATGCAGAAGAACTGGCGGCATGCACGGCAGCAGGGAACCCAGCTGCAGACCATTGGCTTTGTCCTGTATTCGGTGGGTGCCCTGTTTCAGAACATCCAGGATGTCCCCTTGAAGAAGGAATTCTTCGTGAAGTAT
CAGGACCATGGCTTCTCAGAGATCTTCGCCAACTCACGGGAGGTGAGCAGCCAACTCCGGCTGCCTCCGGGGGAGTATATCATTATTCCCTCCACCTTTGAGCCACACAGAGATGCCGACTTCCTACTTCGTGTCTTCACCGAGA
AGCACAGCGAGTCATGGGAATTGGATGAAGTCAACTATGCTGAGCAACTCCAAGAGGAAAAGGTCTTTGAGGATGACATGGACCAGGACTTCCTACATTTGTTTAAGATAGTGGCA---
GGAGAGGGCAAGGAGATAGGGGCGCACGAGCTCCAGAGGCTGCTCAACAGGATGGCCATCAAATTCAAAAGCTTCAAGACCAAGGGCTTTGGCCTGGATGCTTGCCGCTGCATGATCAACCTCATGGATAAAGATGACTCTGGCA
AGCTGGGGCTTCTAGAGTTCAAGATCCTGTGGAAAAAACTCAAGAAATGGACGGACATCTTCAGAGAGTGTGACCAGGACCATTCAGGCACCTTGAACTCCTATGAGATGCGGTTGGCTATTGAGAAAGCAGGCATCAAGCTGAA
CAACAAGGTAACGCAGGTCCTGGTGGCCAGGTATGCAGATGATGACTTGATCGTAGACTTTGACAGCTTCATCAGCTGTTTCCTGAGGCTAAAGACGATGTTCAGT----------------------------------------
-------------------------------------------
'Oryctolagus_cuniculus_ENSOCUP00000015069' ------------------
ATGGTGGCTTACATAAACCACAGCCGACTCAAGGCCAAGGGCGTGGGCCAACACCACAATGCCCACTGCTACAGGAACCAGAGTTTTGAGGAGCTGCGGGCCACCTGTCTGAGGAGGGGTGAGCTGTTCGAGGACCCCCTGTTCC
CCGCGGAGCCCTGCTCGCTCGGCTTCAAGGACCTTGGCCCCAACTCCAAACATGTGCAGGCCATCAGCTGGCAGCGGCCCAAGGACATCACGAGCAACCCTCAGTTCATCGCTAACGGGATCTCTCCGACTGACATCTGCCAGGG
GATACTCGGGGACTGCTGGCTGCTGGCCGCCATCGGCTCCCTCACCACATGCCCCAAACTGCTGTACCGCGTGGTGCCCAGGGGGCAGAGCTTCAGGAAGAACTATGCAGGCATCTTCCACTTCCAGCTCTGGCAGTTCGGGCAG
TGGATGGACGTGGTGGTGGACGACCGGCTGCCCACCAAGAACAACAAGCTGGTGTTCGTGCACTCGGTCCAGAACTCGGAGTTCTGGAGCGCCCTGCTGGAGAAGGCCTACGCCAAGCTGAGCGGGTCCTACGAAGCGCTGGCGG
GGGGCAGCACCATGGAGGGCCTGGAGGACTTCACCGGGGGCGTGGCCCAGAGCTTCCAGCTGCAGAGGCCGCCGCCTGACCTGCTGAGGCTCCTGCGGAAGGCAGTGGAGCGCTCCTCCCTCATGGGCTGCTCCATCGAAGTCAA
CAGTGACAGTGACCTGGAGTCCGTGACCCACAGGATGCTGGTGAGGGGACATGCGTATGCAGTGACCGGACTTCAGGACGTCCTCTTCAGAGGCAAGGCGGAAACGCTGATTCGGGTGCAGAATCCATGGGGCCGGATTGAGTGG
AACGGAGCCTGGAGTGACAACGCCAGGGAATGGGAAGAGGTCAGCCCGGACATGCAGATAGAGTTGCTGCACAAGAAGGAGGACGGGGAGTTCTGGATGTCCTACCAGGATTTCCTGAAGAACTTCACGCTCCTGGAGATCTGCA
ACCTCACACCCGACACGCTGTCCGGGGAATACAAGAGCTACTGGCACACCACCTTCTACGAGGGCAGCTGGCGCAGGGGCAGCACCGCGGGGGGCTGCAGGAATAACCCCGACACATTCTGGACCAACCCCCAGTTCAGGATTTC
TCTCCCTGAGGAGGACGACCCTGACGAGGACTCGGAGGACAACACAATCGTCTGCACCTGCCTTGTGGCCCTGATGCAGAAGAACTGGCGGCACGGGCGGCCGCAGGGCTCCCAGCTCCACACCATTGGCTTCGTCATCTTCTCG
GTCCCGAAGGAGTTTCAGAACATCCAGGATGTCCACTTGAAGAAGGACTTTTTCACAAAGTATCAGGACCATGGCTTCTCTGAGATCTTCACCAACTCCCGGGAGGTGAGCAGCCAGCTCCGGCTGCCGCCCGGGGACTACATCA
TCATCCCTTCCACCTTCGAGCCGCACAGAGACGCCGACTTCTTGCTTCGGGTCTTCACGGAGAAGCACAGCGAGTCCTGGGAGCTGGATGAAGTCAACTACTTGGAGCAGCTTCAGGAGGAGAATGTCTCCGAGGAGGCGTTGGA
CCAGAACTTCATCCGTCTGTTTGAGATCGTGGCA---
GGAGAGGTCTTTGAGATATACACGTATGAGCTCCAAAGGCTGCTCAACAGGATGGCCATGAAACTCAGAAACTTCAAGAGCAAGGACTTTGGCTTGGACTCCTGCCGCTGCATGGTCAACCTCATGGATAAAGATGGCTCTGGCA
AGCTGGGGCTTCTGGAGTTCCAGATCCTGTGGAAAAAAATCAAGAAATGGACGGACATCTTCCAAGAGTGTGACCAGGACAACTCAGGGACCCTGAACTCCTACGAGATGCGCTTGGCAATTGAGAAAGCAGGCATCAAGCTGAA
CAACAAGGTGATGCAGGTGGTGGTGGCGAGGTACGCGGATGACAACATGATCGTGGACTTTAACAGCTTCATCAGCTGTTTTCTGAAGCTGAAGGCCATGTTCACCTTCTTCCTGACCATGGATCCCCAGAACACTGGCTGCATT
CACGTGAACCTGCAGCAGTGGCTGCAGGCGATCATGTGGGGA
'Ochotona_princeps_ENSOPRP00000015695' ------------------
ATGGTGGCTTACATAAACCACAGCCGACTCAAGGCCAAGGGTGTGGGCCAGCACAACAACGCCCACAGCTATGGGAACCAGAACTTTGAGGAGTTGCGGGCTACCTGCCTGAGAAGGGGAGAGCTGTTCGAAGACCCCTTATTCC
CTGCAGAACCCTGCTCCCTCGGCTTCAAGGAGCTGGGCCCCAACTCCAAACATGTGCAAAACATCATCTGGCAGCGGCCCAAGGACATCTCAAGCAACCCTCAGTTCATCATGAACGGCATCTCGCCAACAGACATCTGCCAGGG
CATCCTTGGGGACTGCTGGCTGCTGGCTGCCATTGGGGCCCTCACCACGTGCCCCAAACTGATGTACCGCGTGGTACCCCGGGGGCAGAGCTTCCGGAAAAACTATGCCGGCATCTTCCACTTCCAGTTCTGGCAGTTCGGGCAG
TGGATAGACGTGGTGGTAGATGACCGGCTGCCCACCAAACACGACAAGCTGGTGTTCGTGCACTCGGCCCAGAACACGGAGTTCTGGAGCGCCCTGCTGGAGAAAGCCTACGCCAAGCTGAATGGCTCCTATGAGGCCCTGGCAG
GGGGCAGCACCATGGAGGGCTTTGAGGACTTCACGGGGGGCGTGGCCCAGAGCATCCAGCTGCAGAACCCCCCTCCAGACCTGCTGAGGCAGATGCGGAAGGCAGTGGAGCGGTCCTCCCTCGTGGGCTGCTCCATCGAGGTCAC
CAGTGACAGAGACCTGGAATCTGTGACCCACAGGATGCTAGTGAGAGGCCACGCCTACTCAGTGACTGGCCTCCAGGATGTCCTCTACAGAGGCAGGATAGAAACCCTGATTCGGGTGCAGAATCCCTGGGGCCGGATTGAGTGG
AATGGAGCCTGGAGTGACAATGCCAGGGAGTGGGAAGAAGTCTCGCCTGACATCCAGGTTGAACTGCTGCACAAGAAGGAGGACGGCGAGTTCTGGATGTCCTACCAAGATTTCCTAAAGAACTTCACCCTCCTGGAGATTTGTA

GTCTGACACCTGATGCGCTGTCTGGAGAATACAAGAGCTACTGGCACACCACTCTATACGAGGGCAGCTGGCGCAGAGGCAGCACCGCAGGGGGCTGTAGGAATAACCCCGACACATTCTGGACCAACCCCCAGTTCAGGATTTC
TCTCCCAGAGGAGGATGACCCTGACGCAGATTCAGTGGACAACTTGGTCGTGTGCACCTGCCTGGTGGCCCTGATGCAGAAGAATTGGCGGCACGGGCGGCCGCAGGGCTCCCAGCTGCATACCATTGGTTTTGTCATCTTCTCG
GTCCCAAAGGAGTTTCAGAATATCCACGATGTGCACTTGAAGAAGGACTTTTTCACCAAGTACCAGGACCACGGTTTTTCCGAAATCTTCACCAATACCCGGGAGGTGAGCAGCCAGCTCCGGCTGGCCCCTGGGGACTACATCA
TCGTCCCCTCCACCTTTGAGCCTCACAGAGATGCTGACTTCCTGCTACGGGTCTTCACGGAGAAGCACAGCGAGTCCTGGGAGCTGGATGAAGTCAACTACGCAGAGCAGCTTCAGGAGGAGAACATCTCTGGGGATGACATGGA
CCCAAACTTCATCCATTTGTTTGAGGTCGTGGCA---
GGAGAGGACCATGAGATAGATGTGTATGAGCTACAGAGGCTACTCAACAAGATGTCCTCGAAATTCAAAAACTTCAAGAGCAAGGAGTTTGGCCTGGATGCTTGCCGCTGTATGATCAACCTCATGGATAAAGACTGCTCAGGCA
AGCTGGGGATTCTGGAATTCCAGATTCTGTGGAAAAAGATCAAGAAATGGACGGACATCTTCCAAGAGTGTGACCAGGACCACTCAGGGACCTTGAACTCCTATGAGATGCGCTTGGCAATTGAGAAAGCAGGCATCAAGCTGAA
CAACAAGGTGATGCAGGTGCTGGTGGCGAGGTATGCGGATGACAACATGATCGTGGATTTTAACAGTTTCATCAGCTGTTTCCTGAAGCTGAAGGCCATGTTCACATTCTTCCTCACTATGGATCCCCAGAACACTGGCCACATT
CACGTGAACTTGCAGCAGTGGCTGCAGACGATCATGTGGGGA
'Otolemur_garnettii_ENSOGAP00000011397' ------------------
ATGGTGGTTCACATACACAACAGTCGACTCAAGGCCAGTGGTGTGGGCCAACACCATAATGCGTGCAACTATGGCGACCAGAACTTTAAGAAGCTTCACGAAGTCTGTCTGAAGAACGGGCAGCTGTTTGAGGACCCCTTATTCC
CTGCCAGCCCCAGTTCACTGGGCTTCAAGGAACTAGGCCCCACCTCCAAAAATGGTCAAGACATTTCCTGGCAGCGGCCCAAGGAAATCACCAAGAACCCTTGCTTCTTTGCAAATGGAATCTCTCCAACAGACATCTGTCAGGG
GATACTTGGAGATTGCTGGCTGCTGGCTGCCATCGGCTCCCTTACCACATGCCCCAGACTGCTGTACCGTGTGGTGCCCAAGGAGCAGAGCTTCGAAAAAGACTATGCTGGCATCTTCCATTTCCAGATTTGGCAGTTTGGGCAG
TGGGTGGACGTAGTGGTGGATGACCGGCTGCCCACCAAGAATAACAAGCTGGTGTTCCTACACTCACCTGAGCACTCTGAGTTCTGGGGTGCCCTGCTGGAGAAAGCATATGCCAAGTTGAGCGGGTCCTATGAAGCACTGTCTG
GGGGCAGCACAATGGAGGGCTTTGAGGACTTCACCGGGGGTGTGGCCCAGAACTTCCAGCTCCAGAGCCCCCCTCAAGACCTGCTGAGGCTCCTTAGAAAGGCTATGGCTCGATCCTCCCTCATGAGTTCACATCTTCATATCGC
CAACGACACTGAGTTGGAATCAATGACCCAGAAGAAGCTGGTTAGAGGCCATGCTTACTCAGTGACTGGCCTTCATGATGTCACCTACAGAGGCAAGATGGAAACACTGATTCGGGTCCGGAATCCCTGGGGTCGGATTGAGTGG
ACCGGAGCATGGAGTGACAACTCTAGGGAGTGGGAAGAGGTGGCCCGAGACATTGAGATAAAGCTGCTGCACAAGAAGGAGGATGGGGAGTTCTGGATGTCCTACGAAGATTTCCTGAGCAACTTCTCTCTCCTGGAGGTCTGCA
ACCTCACACCTGACGCGCTGTCTGGGGACAGCAAGAGCTATTGGCACACAACCTACTTTGAGGGCCGCTGGCGCAGAGGCAGCACCGCGGGGGGCTGCAGGAACCACCCCAGTACCTTCTGGACCAACCCCCAGTTTAAGATCTT
TCTCCCTGAGGAGGACGACCCAGATGATGACTTAGAGGACAATCCAGTCGTCTGCACCTGCCTGGTGGCCCTGATGCAGAAGAACTGGCGGCATGCAAAGCCTGAGGGGGCCCAGCTGCAGAGCATTGGCTTTGTCATCTTCACG
GTCCCAAAGGAGTTTCAGAACATTCAGGATGTCCACTTGAAGAGGGACTTCTTCGTGAAGTACCAGGACCTCGGCTTTTCAGAGATGTTTGCCAACTCGAGGGAGGTAAGCAACCAACTGCGGCTGCCACCGGGGGAATATATCA
TTATTCCCTCCACCTTCGAGCCACACAAAGATGCTGACTTCCTTCTTCGGATCTTCACAGAGAAGCACAGCGAGTCCTGGGAACTGGATGAAGTCAACTATGCTGAGCAGCTCCAAGAGGAGGAACCCTCTGAGAAAGAAATGGA
CAAGGAATTCATACATTTGTTTGAGATAGTGGCA---
GGAGAGGGCAAGGAGCTAGGTGTATTTCAACTGCGGAGGCTGCTCAACAAGATGGTCTCCAAACTCAAAAGCATCAGGAGCCAGGACTTTGGCGTGGATGCTTGCCGCTGCATGATCAACACCATGGATAAAGACGGCTCTGGCA
AGCTGGGACTTCTAGAGTTCCAGGTGCTGTGGAAAAAAATCAAGAAATGGACGGACATCTTCAGAGAATGTGACCTGGACCACTCAGGAACCTTGAACTCTTACGAGATGTGGTTGGCTATTGATAAAGCAGGCATCAAGGTGAA
CAACAAGGTGCTGCAGGTACTGGTGGCCAGGTATGCAGATGATGACATGATCATAAAGTTTGACAGCTTCATTACCTGTTTCCTGAGGCTAAAGGCCATGTTCACGTTCTTTCTAACCATGGACCCCAGGAATACTGGCCATATT
TGCTTAAACCTGGAACAGTGGCTGGAGATCACCATGTGGGGA
'Equus_caballus_ENSECAP00000004727' ------------------
ATGGCAGCTCACATCAGCCAGAGCCGACTCAAGGCCAAGGGCGTGGGCCGGTACAACAATGCCCAGAGCTACAGCAACCAGATCTTCGAGGAGCTGCGAGCAGTCTGCCTGAGGAAGGGGGAGCTGTTCGAGGACCCCTTATTCC
CTGCTGAGCACAGTTCGCTGGGCTTCAAGGACCTGGGCCCCGACTCCAAAAATGCGCAGAACATCCGCTGGCAGCGGCCCACACAAATTACGAGCAATCCTCAGTTCATCGTGGATGGGGTCGGTCCCACAGACATCTGTCAGGG
GGTGCTCGGGGACTGCTGGCTGCTGGCTGCCATTGGCTCCCTTACCACATACCCCAAACTGCTGAACCGCGTGGTACCCAAGGGGCAGAGCTTCAAGAAAAACTATGCTGGCATCTTCCATTTTCAGATTTGGCAATTTGGGCAG
TGGGTGGACGTGGTGGTGGATGACCGGCTGCCCACAAAGAACGGCAAGCTGGTGTTCGTGCACTCACCCGAGCGCACTGAGTTCTGGAGTGCCCTGCTGGAGAAGGCATATGCCAAGTTGAACGGGTCCTATGAAGCACTTTCAG
GGGGCAACACGGTGGAGGGCTTTGAGGACTTCACAGGGGGTGTGACCCAGAGCCTCCAACTCCAGAGGCCCCCTCGGAACCTGCTGAGGATGCTTAGGAAGGCCATAGAGCGATCCTCCCTCTTGGGCTGCTCTATCGAGATCAC
CAGCAACAGCGACTTGGAATCCTTGACCCAGAAGATGCTGGTGAGAGGACATGCTTACTCAGTGACTGGCCTTCAGGACGTCTTGTACCAAGGCAAGATGGAAACATTGATTCGGGTCCGGAATCCCTGGGGCCGGATTGAGTGG
AATGGATCCTGGAGTGACAATGCCAGGGAGTGGGAAGAGGTGGACCTAGACATCCAGAAGCAGCTGCTACACAAGAAGGAGGACGGGGAGTTCTGGATGTCCTACCAAGATTTCCTGAACAACTTCACACTCCTGGAGATCTGCA
ACCTGATGCCTGACACGCTCTCTGGGGACTGCAAGAGCAGTTGGCACGCCACCTTCTACGAGGGCAGCTGGCGGAGGGGCAGCACCGCGGGGGGCTGCAGGAACTACCTCGACACATTCTGGACCAACCCCCAGTTTAAGATCTC
TCTCCCCGAGGAG---------
GACGACGACCCAGAGAACAATGAAGTTGTTTGCACCTGCCTGGTGGCCCTGATGCAGAAGAACTGGAGGCAGGCACGGCCCCAGGGAGCCCAGCTGCAGACCATTGGCTTTGTCATCTACACGGTCCCAAAGGAGTTTCAGAACA
TCCAGGATATCCACTTGAAGAAGGACTTCTTCATGAAGTATCAGGACCACGGCTTCTCAGAGATCTTCTCCAACGCACGGGAGGTGAGCAGCCATCTCCGGCTGCCTCCAGGGGACTATATCATCATTCCTTCCACCTTTGAGCC
GCACAAGGATGCTGACTTCTTGCTTCGGGTCTTCACAGAGAAGCATAGCGAGTCCTGGGAACTGGATGAAGCCAACTATGCTGAGATGCTCCAAGAGGAAAACATCTCTGAGGACAAAGTAAACCAGGACTTCACACGTCTGTTT
GAGACAGTGGCTGGAGGAGAGGGCAAGGAGATAGGCATGTACGGGCTACAAAAGCTGCTCAACAAGGTGGTCTCCAAATTCACACACTTCAAGACCCAGGGCTTCGGCCTGGACGTGTGCCGCTGTATGGTCAACCTCATGGATA
AAGATGACTCTGGCAAGCTGGGGTTTCTGGAGTTCCAGATCCTGTGGAAAAAAATCAAGAAATGGACGGACATCTTCCGTGAGTGTGACGAGGACAATTCAGGCGCCTTGAACTCCTATGAGATGCGCTTGGCCATTGAGAAAGC
AGGCATCAAGCTAAATAACAAGGTGATGCAGGTGCTGGTGGCCAGGTATGCAAATAAGAACATGATGGTGGATTTTGACAGCTTCATCTACTGTTTCCTGAAGCTGAAGGCCATGTTTAGGTACTTTCTAACCATGGACCCCAGA
AATACTGGTTATATTCACTTGAACCTGAATGAGTGGCTGCAGATAACCATGTGGGGA
'Sus_Scrofa_ENSSSCP00000001850' ------------------
ATAGCGGCTCACATAACCCAGAGCCGACTAAGGGCCAAAGGTGTGGGCCAGCAGCACAATGCCGAGAACTACAACAACCAGAGCTTTGAGGAGCTGCGAGCAATCTGCCTACGGAAGGGGCTGCTGTTTGAAGATCCCTTATTCC
CTGCCGAGCCCAGTTCGCTGGGCTTCAGGGAACTGGGCCCCAACTCCAAAAATGTGCAGAACATCAGCTGGCAGCGACCCAGTCAAATCACCAGCAATCCTCAATTCATTGTGAATGGGGTCAGCCCGACAGACATCTGCCAGGG
GGTGCTTGGGGACTGCTGGCTGCTGGCTGCCATCGGCTCCCTCACCGTGTACCCCAAACTCTTCCACCGTGTGGTGCCCAAGGGCCAGAGCTTCAAGAAAAACTATGCCGGCATCTTCCACTTCCAGATCTGGCAGTTTGGCCAG
TGGATGGATGTGGTGGTGGATGATCGGCTGCCCACAAAGAATGGCAAGCTGGTGTTTGTGCACTCAGCCGAGCGCACTGAGTTCTGGAGCGCCCTGCTGGAGAAGGCCTACGCCAAGCTGAACGGATCCTATGAAGCACTGTCAG
GGGGGAACACAATGGAAGGCTTTGAGGACTTCACGGGGGGCGTGACCCAGAGCTTCCAGCTCCAGACACCCCCTCAGAACCTGCTGAGGATGCTTAGGAAGGGCATAGAGCGATCCTCCCTCATGAAAAAAAAAACAAACGTCAC

CAGCAACAGCGACTTGGAGTCGCTGACACAGAGGATGCTGGTAAAAGGACATGCTTACTCGGTGACCGGCCTTCAGGATATCTGGTACCACGGCAAGACAGTAACACTGATTCGGGTCCGGAATCCCTGGGGCCGGATTGAGTGG
AATGGAGCCTGGAGTGACAATGCCAAGGAGTGGGAAGAGGTGTCCCCAGACTTCCAGAGGCAGATGCTGCACAGGAAGGAAGACGGGGAGTTCTGGATGTCCTACCAAGATTTCCTGGACAACTTCACCCTCCTGGAAATCTGTA
ACCTGATGCCTGACACACTCTCT---
GACTACAAGAGCTGTTGGCACACCACCTTCTACGAGGGCAGCTGGCGGAGGGGCAGCACTGCGGGGGGCTGCAGGAGCCACCTCGACACGTTCTGGACCAACCCCCAGTTTAAGCTCTCTCTCCCCGAGGAGGAT------
GACGATGACCCAGAGGCCGAGGAAGCCGTCTGCACCTGCCTGGTGGCCCTGATGCAGAAGAACTGGAGACGGGGGCGGCCGCACGGAGCCCAGCTGCAGACCATCGGCTTTGTCATCTACACGATCCCGAAGGAGTTTCAGAACC
TCCAGGATATCCACTTGAAAAGGGACTTCTTCGAGAAGTATCAGGACCTGGGCTTCTCAGAGATCTTCACCAATTCCCGGGAGGTGAGCAATCACCTCCGGTTGCCTCCAGGCGAATATATCATCATCCCCTCCACTTTTGAGCC
GCATAAGGATGCCGACTTCCTGCTCCGGGTCTTCAGCGAGAAGCACAGCGAGTCCTGGGAACTGGATGAAGCCAACTGCACTCAGCTTCTGCAAGAGGAGACCATCTCGGAGAATGAAATAGACCAGAACTTTCTCCATTTGTTT
CAGATAGTGGCTGGTGGAGAGGATAAGGAGATTGGCGTGTACGAGCTGCAGAAGCTGCTCAATAAGGTGGCATCCAAATTCAGACACCTCAAAACCAAAGGCTTCAGCCTGGACGTGTGCCGCTGTATGATCAACCTCTTGGATA
AAGACAGCTCTGGCAAACTGGGGCTTCAGGAGTTCCAGATCCTGTGGAGAAAAATCAAGAAATGGACGGACATCTTCCAAGAGTGTGACCAGGACCATTCAGGCACCTTGAACTCCTATGAGATGCGCTTGGCAATTGAGAGAGC
AGGGATCAAACTCAGTAACAAGGTGACACAGGTGCTGGTGGCCAGGTATGCAAATGACGACATGATCTTGGACTTTGACAGCTTCATCAGCTGTTTCCTGAGGCTGAAGGCCATGTTCACGTACTTTCTAACCATGGACCCCAAT
AATACTGGCCAGATTTGCTTGAACCTGAACCAGTGGCTGCAGATAACCATGTGGGGA
'Bos_Taurus_ENSBTAP00000004690' ------------------
ATTGCAGCCCGCATAAGCCAGAACCGACTCAAGGCCAAGGGCATGGGCCAGCACCACAACGCCCAGAACTACAACAACCAGAGCTTTGAGGAACTGCGAGCACTCTGCCTACGGAGGGGAGAGCTGTTCGAGGACCCCTTTTTCC
CTGCAGAACCCACTTCACTAGGCTTCAAGGAACTGGCACCCAACTCCAAACACGTGCAGAACATCAGCTGGCAGCGGCCCAGTCAAATCACAAGCAATCCTCAATTCATCGTGAATGGGGTCAGCCCAACCGACATCTGCCAGGG
TGTGCTCGGAGACTGCTGGCTGCTGGCTGCCATTGGCTCTCTCACTGCGTACCCCAAACTGCTGTCCCGTGTGGTGCCCAAGGGGCAGAGCTTCAAGAAGAACTATGCTGGCATCTTCCATTTTCAGATCTGGCAGTTTGGGGAG
TGGATGGACGTTGTGGTGGATGACCGGCTGCCCACAAAGAATGGCAAGCTGGTGTTTGTGCACTCAGCGGAGCGCACTGAGTTCTGGAGCGCCCTGCTGGAGAAGGCATATGCCAAGCTGAACGGATCGTATGAAGCTCTGTCGG
GGGGCAACACGGTGGAGGGCTTTGAGGACTTCACAGGGGGCGTGACGCAGAGCTTCCAACTCCAGAATCCCCCTAGGAACCTGCAGAGGATTCTTAGGAAGGCCGTGGAGCGATCTTCCCTCATGGGATGTGTTCTGCAGGTCAC
CCACGAACATGAGCTGGAGTCCTTGACACAGGGTATGCTGGTGAGAGGACATGCTTACTCAGTGACCGGCCTCCAGGATGTCTCCTACCAAGGCAGGACAGAAACACTGATCCGAGTCCGGAATCCCTGGGGCCGGATTGAGTGG
AATGGAGCCTGGAGTGACAACGCCAAGGAGTGGGAGGAGGTGGCCCCAGATGTCCAGAGGCAGCTGCTGCACAGGAAGGAGGACGGGGAGTTCTGGATGTCCTACCAAGATTTCCTTGGCAACTTCACCCTCCTGGAAATCTGCA
ACGTGATACCCGACACGCTCTCCGGGGACTACAAGAGCTGTTGGCATACCACCTTCTACGAGGGCAGCTGGCGGAGGGGCAGCACCGCAGGGGGCTGCCGGAGCTACCTGGACACATTCTGGACCAACCCCCAGTTTAAGATCTG
TCTCCCCGAGAAG---------
GATGACGACATAGAGGACGATGAAGCCGTCTGCACCTGCCTGGTGGCCCTGATGCAGAAGAACTGGAGGCGGACGAGGCCCCACGGAGCCCAGCTGGAGACCATTGGCTTCGTCATCTATTCGATCCCAAAGGAGTTTCAGAACC
TCCAGGATGTCCACTTGAAAAAGGACTTCTTCGTCAAGTACCAAGACCTGGGTTTCTCGGAGATTTTCACCAACTCGCGGGAGGTGAGCAGCCAGTTCCAGCTGCCCCCAGGGGAATACATCATCATCCCCTCCACCTTCGAGCC
GCACAAGGACGCCGACTTCCTGCTGCGGGTCTTCACAGAGAAGCACAGCGAGTCCTGGGAACTGGATGAAGCCAACTACGCTGAGCTTCTCCAAGAGGAGAGCATCTCTGAGACCGACATAGACCAGGACTTCGTACGTTTGTTT
CACATAGTGGCCGGTGGAGAGGGCAAGGAGATAGGCATGTATGAGCTACAGAAGCTGCTCAACAAGGTGGTCTCCAGATTCAAAAACTTCAAAACCAAGGGCTTCAGCCTGGACGTGTGCCGCTGCATGGTCAACCTCTTGGATA
AAGATGGCTCTGGCAAACTGGGGCTTCGGGAGTTTCAGGTCCTGTGGAGAAAAATCAAGAAATGGACGGACATCTTCCGAGAGTGTGACCAGGACCAGTCAGGCACCTTGAATTCCTATGAGATGCGCTTGGCAGTTGAGAAAGC
AGGCATCAAGCTGAACAACAAGGTCACGCAGGTGCTGGTGGCCAGGTACGCGAATGACAGCTTGATCATGGAGTTTGACAGCTTCATCAGCTGTTTCCTGAGGCTGAAGGCCATGTTCGCGTACTTTCTAACCATGGACCCTGAG
AACACTGGCCAGATTTCCCTGGACCTGAACCAGTGGCTGCAGATAACCATGTGGGGT
'Ailuropoda_melanoleuca_ENSAMEP00000012638' ------------------
ATGGTGGCTTCCATAAGCCTGAACCGACTCAAAGCCAAAGGCGTAGGCCAACACCACAATGCCCAGAACTACAATAACCAGAACTATAAGGACCTGCTGGCAGCCTGTCTGAGGAGGGGAGAACTGTTTGAGGACTCCTTTTTCC
CTGCTGAGCCCAGTTCGCTGGGCTTCAAGGATCTGGGCCCCGGCTCCAAGAGTGTGCAGAACATCTCTTGGCAACGGCCCGGTCAAATCACAAGCAATCCTCACTTCATTGTGAACGGGATCAGCCCAACAGACATCTGTCAAGG
TGTACTAGGGGATTGCTGGCTGCTGGCTGCCATCGGCTCCCTTACCACATGCCCCAAACTGCTATACCGGGTGGTACCCAGGGGGCAGAGCTTCAGGAAAAACTATGCCGGCATCTTCCATTTTCAGATCTGGCAGTTTGGGCAG
TGGGTGGACGTGGTGGTGGACGACCGGCTGCCCACAAAGAATGGCAAACTCGTGTTTGTGCATTCCGCTGAGCGCACTGAATTCTGGAGCGCCCTGCTGGAGAAGGCCTATGCCAAGCTGAATGGGTCCTATGAAGCACTGTCGG
GGGGCAACACCATGGAGGGCCTTGAGGACTTCACGGGGGGTGTGACCTATAGCTTCCAACTCCAGAAGCCCCCTCGGAACCTGCTGAAGATTCTTAGGAAGGCCGTTGAACGATCCTCCCTCATGCGCCCCTCCCTGCATGTCAC
CAGCGACAGCGAGTTGGAGACCTTGACCCACAGGATGCTGGTGAGAGGGCACGCTTACTCCGTGACCGGCCTCCAGGATGTCTGGTACCAAGGCAGGACAGAAACCCTGATTCGGGTCCGGAATCCCTGGGGCCGGATCGAGTGG
AATGGAGCCTGGAGTGACACTGCCAGCGAGTGGGACAAGGTGGCCCCGGACGTCCAGAAGGTGCTCCTGCTCAGGAGGGAGGACGGGGAGTTCTGGATGTCCTACGAAGACTTCCTGGACAACTTCACGCTGCTGGAGATCTGCA
ACCTGACGCCCGACGCCCTCTCTGGGGACTACAAGAGCTGCTGGCACACCACGTTCTACGAGGGCAGCTGGCGGCAGGGCAGCACTGCGGGGGGCTGCAGGAACCACCTCGACACATTCTGGAGCAATCCCCAGTTTAGGATCTC
TCTCCCCGAGGAGGATGAC---
GACGACGACCCGGAGGACGACCAAGCCGTCTGCACCTGCCTGGTGGCCCTGATGCAAAAGAATTGGCGACAGGCGCGGCCCCAAGGAGCCCAGCTGCAGACCATTGGCTTTGTCATCTACACGGTCCCAAAGGAGTTTCAGAACA
TCCAGGATGTTCACTTGAAGAAGGACTTCTTCATAAAGTATCAGGACCATGGCTTCTCGGAGATCTTCATGAACTCCCGGGAGGTGAGCAGCCATCTCCGGCTGCCTCCGGGGGAGTATGTCATCATCCCCTCCACCTTTGAGCC
GCACAAGGATGCTGACTTCCTGCTTCGGGTCTTCACAGAGAAGCACAGCGAGTCCTGGGAACTGGACGAAGTCAACTATGCTGAGCTACTCCAAGAGGAGAAGTTCTCTGAGAAGGACATAGATCAGGACTTCATAAATTTGTTT
GAGATAGTGGCAGGAGGCAAGGACAAGGAGATAGGCATGTACGAGCTACAGAAGCTGCTCAACAAGGTGGTCTCCAAACTCAGAAACTTCAGGACCAAGGGCTTCGGCCTAGACGTGTGCCGCTGTATGATCAACCTCATGGATA
AAGATGGCTCTGGCAAGCTGGGGCTTCCCGAGTTCCAGATCCTGTGGAAAAAAATCAAGAAATGGACGGACATCTTCCGAGAGTGTGACGAGGACCACTCGGGCAGCCTGAACTCCTATGAGATGCGCTTGGCAATCGAGAAAGC
AGGCATCAAGCTGAGTAACAAGGTGACCCAGGTGCTGGTGGCCAGGTATGCAAACGACGACATGATCGTGGACTTTGACAGCTTCATCAGCTGCTTCCTGAGGCTGAAGGCCATGTTCACGTACTTTCTATCCATGGACCCCAAG
AATACTGGCTATATTCGCCTGAATCTGAACCAGTGGCTGCAGACAACCATGTGGGGT
'Mustela_putorius_furo_ENSMPUP00000012533' ------------------
ATGGCGGCCCACATAAGCCAGAACCGGCTCAGGGCCAAGGGCGTAGGCCAACACCACAATGCCGAGAACTTCAACAACCAGAATTATGAGGACCTGCAAGCAGCCTGTCTGAGGAGGGGAGAACTGTTCGAGGACTCCTTTTTCC
CCGCCGAACCCAGTTCCCTGGGCTTCAACGATCTGGGCCCCAGCTCCAAAAGTGTGCAGAACATTTCCTGGCAGCGGCCTTGTCAAATCACAAGCACCCCTCACTTCATCGTGAATGGGCTGAGTCCAACAGATATATGCCAAGG
CGTACTTGGGGACTGCTGGCTGCTGGCTGCCATTGGCTCCCTTACCACATGCCCCAAACTGCTGTATCGTGTGGTACCCAGGGGGCAGAGCTTCAGGAAAAACTATGCTGGCATTTTCCATTTTCAGATTTGGCAGTTTGGGCAG

TGGGTGGATGTGGTGGTGGACGACCGGCTGCCCACAAAGAATGGCAAGTTGGTGTTCGTGCATTCCGCCGAGCGCACTGAGTTCTGGAGCGCCCTGCTGGAGAAGGCATATGCCAAGCTGAACGGGTCTTATGAAGCGCTGTCAG
GGGGCAACACCGTGGAGGGCTTTGAGGACTTCACGGGGGGTGTGACCTATAGCCTCCAACTCCAGAAGCCCCCTCGGAACCTGCTGAGGATGCTTAGGAAGGCCATCGAGCGATCCTCCCTCATGGTGCCCCAAAATGACGTTAC
CAGCAATAGTGAGTTGGAGACCTTGACCCAGAAGATGCTGGTGAGAGGGCATGCTTATTCCGTGACCGGCATCCAGGATGTCTGGTACCAAGGTCGGGCAGAAACCCTGATTCGGGTCCGGAATCCCTGGGGCCGGATCGAGTGG
AATGGAGCCTGGAGTGACAACGCCAGGGAGTGGGAAAGTGTGTCTCCGGACCTCCAGAACCAGCTCCTGCTCCGGAAGGAGGACGGGGAGTTCTGGATGTCCTACAAAGACTTCCTGGACAACTTCACGCTGCTGGAGATCTGTC
ACCTGACGCCCGATGCCCTCTCCGGGGACTACAAGACCTGTTGGCACACCACCTTCTATGAGGGCAGCTGGCGGCGCGGCAGCAACGCGGGAGGCTGTAGGAACCACCCAGACACATTCTGGAGCAACCCCCAGTTTAGGATCTC
TCTCCCTGAGGAGGATGAT---
GAGGACAACCCAGAGGACAACCAAGCCGTGTGCACCTGCCTGGTGGCCCTTATGCAGAAGAACTGGCGACAGGCGCGGCCCCGGGGAGCCCAGCTGCAGACCATTGGCTTTGTCATCTACGAGGTCCCAAAGGAGTTTCAGAACA
TCCAGGATTTTCGCTTGAAGAAGGACTTCTTCATCAAGTATCAGGACCACGGCTTCTCAGAGATCTTCACTAACTCACGGGAGGTGACCAGCCATCTCCGGCTGCCTCCAGGGGAATATGTCATCATTCCCTCCACCTTTGAGCC
TCACAAGGATGCTGACTTCCTGCTTCGGGTCTTCACAGAGAAGCACAGCGAGTCCTGGGAACTGGATGAAACCAACTATGCTGAGGTACTCCAGGAGGAGAAATTCTCTGAGAAGGACATAGATCCGGACTTCATACGTTTGTTT
GAGATAGTGGCAGGAGGAGAGGACAAGGAGATAGGCATGTATGAGCTACAGAAGCTCCTCAACAAGGTGGTCACCAAACTCAAAAACTTCAGGACCCAGGGCTTTGGCCTAGACGTGTGCCGCTGTATGGTCAACCTCATGGATA
AAGATGGCTCTGGCAAGCTGGGGCTTCCCGAATTCCAGATCCTGTGGAAAAAAATCAAGAAATGGACGGACATCTTCCGGGAGTGTGACGAGGACAACTCGGGCACCTTGAACTCCTATGAGATGCGCTTGGCACTCGAGAGGGC
AGGCATCAAGCTGAATAACAAGGTGACGCAGGTGCTGGTGGCCAGGTACGCAAACGACGACATGATCGTGGATTTTGACAGCTTCATCAGCTGTTTCCTGAGGCTGAAGGCCATGTTCACATACTTTCTATCCATGGACCCCGAG
AATACTGGCTATATTCGCTTGAATCTGAACCAGTGGCTGCAGACCACCATGTGGGGA

'Myotis_lucifugus_ENSMLUP00000004763' ------------------

ATGGCGGCTCACATCAGCCACAACAGACTCAAGGCCAAGGGCGTGGGCCAGCACAACAACGCCCACAACTACAGAAACCAGAGCTTTGAGGACCTGCGAGAGCTCTGTCTGAGGAGGGGGGAACTGTTCAAGGACCCCGTATTTC
CTGCCGAACCCACTTCTCTGGGCTTCAAGGACCTGGGCCCCAACTCCAGAAACGTGCAGAACATCAGCTGGCGGCGGCCCAGTGAAATCACAAACAATCCTCACTTCATTATGAACGGGGTCAGCCCCACGGACATCTGCCAGGG
GGTGCTCGGGGACTGCTGGCTGCTGGCTGCCATCGGCTCCCTTACCACGTGCCCCAAGCTGCTGTACCGCGTGGTGCCCAAGGGGCAGAGCTTCAAGAAAAACTATGCGGGCATCTTCCATTTTCAGATCTGGCAGTTTGGGCAG
TGGGTCAACGTGGTGGTGGATGACCGGCTACCCACAAAGAACGGCAAGCTGTTGTTTGTGCACTCGGGCGAGCGCGCTGAGTTCTGGAGTGCCCTCCTGGAGAAGGCGTACGCCAAGCTGAGCGGGTCCTACGAGGCCCTGTCGG
GGGGCAACACGATGGAGGGCTTTGAGGACTTCACGGGGGGCGTGACCCACAGCTTCGAGCTCCAGCGGCCCCCGAAGAACCTGCTGAGGATGCTCAGGAAGGCCGTAGACCGCTCTTCTCTCATGGGTTGCTCCATCGAAATCAC
CAGCAAGAGTCAGCTGGAATCCGTGACCCAGAGGATGCTGGTGAGAGGTCATGCCTACTCAGTGACCGGCCTGCGGGATGTCTCCTACCGCGGCAACGTGGAAACCCTCATTCGGATCCGGAATCCCTGGGGCCGACTGGAGTGG
AATGGAGCCTGGAGTGACAATGCCAGGGAGTGGGAAGAGGTGTCCCCAGACACTCAGAAGCAGCTGCTGCACAAGAAGGAGGACGGGGAGTTCTGGTCAGGAACCCAAGACTTCATGAGGAATTTCACT---
TCCGAGATCTGCAGCCTCATGCCCGACGCGCTCCTGGGAGACTACAAGAGCTGTTGGCACACCACCTTCTACGAGGGCAGCTGGCGGCGGGGCAGCACGGCGGGCGGCTGCAGGAACCACCTCGACACATTCTGGACCAACCCCC
AGTTTAGGATCTCTCTCCTCAAGGAG---------
GACGACGACCTCGAGGAGGATGAGGTCATCTGCACCTGCCTGGTCGCCCTGATGCAGAAGAACTGGCGGCAGGCGCGGCCCCAGGGGGCACAGCTGCTCACCATCGGCTTCGTCCTCTACAAGGTGCCAAAGGAGTTGCAGGACA
CCCAGGATATTCACCTGAAGAAGGAGTTCTTCCTGAAGTATCCGGACCATGGCTTCTCAGAGATCTACACCACCTCGCGGGAGGTGAGCAGCCACCTCCAGCTGCCCCCGGGGGAATACATCATCATCCCCTCCACCTTCGAGCA
GCACAAGGACGCCGACTTCCTGCTTCGGGTCTTCACGGAGAAGCACAGCGAGTCCTGGGAACTGGATGAAGCCAACTGTGCTGAGATACTCCAAGAGGAGGTCATCTCGGAGAGTGACATAGACCCGGACTTCAAGCGTCTGTTT
GAGATAGTGGCA---
GGAGAGGACAAGGAGATCGGCGCGTACGCGCTCCAAAGGCTGCTCAACAAGGTGGTCTCCAAACTCAGAAACTTCAAGAGCAAGGGCTTTGGCGTGGACGTGTGCCGCTGCATGATCAACATCATGGACAAAGATGGCTCTGGCA
AGCTAGGGCTTCCGGAGTTCCAGATCCTGTGGAGAAAAATCAAGAAATGGACGGACATCTTCCAAGAATGCGACCAGGACAACTCGGGCACCTTGAACTCCTACGAGATGCGCTTGGCAATTGAGAAAGCAGGCATCAAGGTGAA
CAACAAGGTGACGCAGGTGCTGGTGGCCAGGTATGCAAATGACGACATGCTCATGGATTTTGACAGCTTCGTCAGTTGTTTCCTGAGGCTGAAGGCCATGTTCACATACTTTCTAACTATGGACCCCAAGAATACTGGCTATATC
CATTTGGACCTGGACCAGTGGCTGCAGATAACCATGTGGGGA

'Rattus_norvegicus_ENSRNOP00000059934' ------------------

ATGGTGGCAACCATAAACAGCAACAGGCTCAAGGACAAGGGCGTGGGCCAATACCAAAATGCCTACAACTATAAGAATCAGAACTTTGAGGACCTACGAGCATCATGTCTCAAGATGGGGGAACTGTTCGAAGACCCCTTTTTTC
CTGCTGAACCCCAGTCTATAGGCTTCAAGGAGCTGGGCCCCAAGTCTCAAGAAGTGCATAACGTCTACTGGCAGCGGCCCAAGGATATCATACATAACCCCCAATTCATTACGAATGATTTCTCTCCCACGGACATCTGCCAAGG
CATCCTGGGAGACTGCTGGCTGCTGGCCGCCATTGGCTCCCTTACCACGAGCCCCAAACTGCTGTTCCGTGTGGTGCCTAGAAGTCAGAGCTTCAAGAAAAACTATGCTGGCATCTTCCATTTTCAGCTTTGGCAGTTCGGGCAT
TGGTTGAACGTGGTGGTGGACGACCGGCTGCCCACTAAGAACAACAAATTGGTGTTCGTGCACGCCTCCCATCGGCAGGAGTTCTGGGGCGCCCTGCTGGAGAAGGCCTACGCCAAGCTGAATGGATCCTACGAAGCACTGACTG
GAGGCAGCACCATGGAGGGTTTGGAAGACTTCACAGGCGGCATGGCACAGTGCATTCGGCTCCAGAATCCCCCTCACAACCTGCTAAGGCTCCTCAGGAAAGCCTTGGAGAAGTCTTCCCTCATGGGTTGCTCCATCGAAGCCAC
CAACAGTGACGATGTGGAAAAAATGGCCCATAATATGCTGGTGAAAGGTCACGCTTATGCAATCACCGGCCTACAAGATGTTTACTACAGAGACAAGTTGGAAACCCTAATTCGAATCCAGAACCCCTGGGGCAGGATTGAGTGG
AATGGAGCCTGGAGTGACAAAGCCAAGGAGTGGGAAGAGGTGATGCCGGAAGTTCAGATGCAGCTGCTGCACAAGAAGGAAGACGGGGAGTTCTGGATGTCCTATGAAGACTTCATGAGCAACTTTACATTGCTGGAGATTTGCA
ACCTCACCCCGGACGCGCTCTCCTCCGATTACAAGAGCTGCTGGCACACAACCTTCTTTGAGGGCAGCTGGCGAAGGGGGAGCACTGCAGGGGGCTGCAGAAACTACCCCGAGACGTTCTGGAGCAACCCCCAGTTTAGGATCTC
TCTCCCAGAAACGGATGATCCAGAGGATAGCTCTGACAAAAGCGAAATGGTTTGCACGTGTCTGGTGGCGCTGATGCAGAAGAACTGGAGACATGCTCGA---
GAGGGACCCCAGCTGCTCACCATAGGCTTCGTCGTCTTCTCGGTTGGGGTACAGTTTCAGAACCTCCAGGACATCCACCTGAAGAAAGACTTCTTTATGAAATATCGGGACCATGGCTTTTCAGAGATCTTCACCAACACGAGGG
AGGTGAACAGCCATCTTCGGCTGCCCCCCGGGGAGTACGTCATTATCCCCTCCACCTTTGAGCCTCATAAAGATGCTGACTTCCTGCTTCGAGTCTTCACAGAGAAACACAGTGAGACCTGGTTGCTGGATGAAGTCAACATGCT
TGAGCAACTCCAAGAGGAGACCATCACTGATGCAGATTTGGACCAGAATTCTGTAGAGTTGTTTGAGACGTTGGCA---
AACCGGGACAGCCAAGTAGATATGTATGACCTACAAAAACTTCTCAACAAGATGTCAAGTAAATTCAAGAGCTTCAAGTCGAAGGGCTTTAGCTTGGATGTCTGTCGCCGTATGGTCAACCTCATGGACAAAGACGATTCTGGAA
AGCTGGGGCTTCATGAGTTCCATATCCTATGGAAAAAGATCAAGAAATGGATGGAAATCTTCAAAGAGTGTGATCAAGACCGCTCAGGGAACTTGAACTCCTACGAGATGCGCTTGGCAATAGAGAAAGCAGGCATCAGAATGAA
CAACAGGGTGACTGAGGTGGTCGTTGCTAGATATGCAGATGCCAACATGATCGTGGATTTTGACAACTTCATCAACTGTTTCCTAAGGCTCAAGGCCATGTTTGCGTTCTTCTTATCCATGGACACCAAGAAAACTGGTTCTATT
TGCCTGAATATAAACCAGTGGCTGCACATAACCATGTGGGGG

```
       'Mus_musculus_ENSMUSP00000113132/1-2142'                                                                    ----------------
ATGGTGGCTACCATAAACCACTCCAGGCTCAAGGACAAGGGCGTGGGCCAACACCAAAATGCCTACAACTATAAGAATCAGAACTATGAGGACCTTCGAGCAGAGTGTCTCAGGAAGGGGGAACTGTTCGAAGACCCCTTTTTCC
CTGCTGAACCCAGGTCTATCGGTGTCAAGAACCTGGGCCCCAACTCTGAACACATGCAGAATATCTACTGGCAGCGGCCAAAGGATATCATTCATAACCCCCAGTTCATCACGAATGACTTCTCTCCCACGGACATCTGCCAAGG
GATCCTGGGGGACTGCTGGCTCCTGGCTGCCATTGGCTCCCTTACCACCTGCCCCAAACTGCTGTACCGTGTGGTGCCCAGAAATCAGAGCTTCAAGAAGAACTATGCTGGCATCTTCCACTTTCAGCTTTGGCAGTTTGGGCAC
TGGTTGAACGTGGTCGTGGACGACCGGCTGCCCACTAGGAACAACAAGCTGGTATTCGTGCACGCCTCCCATCGCCAGGATTTCTGGAGCGCCCTGCTGGAGAAGGCCTACGCCAAGCTGATCGGATCCTACGGAGCGCTGTCTG
GAGGCAGCACTTTGGAGGGTTTGGAAGATTTCACAGGCGGCGTAGCACAGTGCATTCCGCTCCAGAAGCCCCCTGGCAACATGCTAAGGCTCCTCAAGAAAGCCCTGGAGAAGTCTTCCCTCATGGGCTGTTCCATTGAAGTCAC
AGACAATAGTGAAGTAGAAACCATGACACATAACATGCTGGTGCGAGGACATGCTTATGCAGTGACTGGCCTAGAGGATGTCTACTACAGAGACAAGTTGGAAACTCTAATTCGGATCCAAAATCCTTGGGGTCGGGTTGAATGG
AATGGAGCCTGGAGTGACAAAGCCACAGAGTGGGAAGAGGTGAGCCCAGATGTTCGGGTACAGCTGCTACACAAGAAGGATGATGGCGATTTCTGGATGTCCTATGAAGACTTCATGAGCAACTTTACACTGCTGGAGATCTGCA
ACCTCACCCCGGATGCCCTCAACACCGATTACAAGAGCCGCTGGCACTCCACCTTCTATGAGGGCAGCTGGCGAAGGGGAAGCACTGCAGGGGGCTGCAGAAACCACCCGGAGACGTTCTGGAGCAACCCCCAGTTCAAGATCTC
TCTCCCGGAAGTGGATGACCCAGAGGATGACTCTGAGAAAAACGAAATGGTTTGCACCTGTCTGGTGGCACTGATGCAGAAGAACTGGAGACATGCTCGA---
GAGGGACCCCAGCTCCTCACCATAGGCTTTGTCATCTTCTCGGTCCCAAAGGAGTTTCAGAACCTCCGGGACATCCACCTTAAGAAAGACTTCTTCTTGAAATACCGGGACCACGGCTTTTCAGAGATCTTCATCAACTCGAGGG
AGGTGAACAGCCATCTCCGGCTGCCCCCGGGGGAATACGTCATTATCCCCTCCACCTATGAGCCCCATAAAGATGCTGACTTCCTGCTTCGGGTCTTCACGGAGAAACACAGTGAGACCTGGTTGCTGGATGACGCCAACCGGTT
TGAACATCTCCAAGAGGAGACTGTCACTGACAAGGATTTGGACAAGGATTCTCTTCAGCTGTTTAAGATAATGGCA---
AACGAGGATGGCGAAGTGGATATGTATGCACTACACAAACTTCTCAACAGGATGACAGCTAAATTAAGGAACTTCAAGACGAAGGGCTTTAGCTTGGAAGTCTGTCGCCGGATGATCAACCTCCTGGATAAAGATGGTTCTGGCA
AGCTGGAGCTTCATGAATTCCAGGTCCTGTGGAAAAAGATCAAGAAATGGACGGAAATCTTCAAAGAGTGTGATGAGGACCGCTCAGGGAACTTGAACTCCTATGAGATGCGCCTGGCAATAGAGAAAGCAGGCATCAAGATGAA
CAACAGAGTGACCGAGGTAGTCGTTGCCAGGTACTCGGAT---
AACATGATTGTGGACTTTGACAGCTTCCTCAACTGTTTCCTGAGGCTCAAGGCCATGTTTGCGTTCTTCTTATCCATGGACACCAAGAAAACTGGTTCTATTTGCTTGGATATTAACCAGTGGCTGCAAATAACCATGTGGGGG
       'Cavia_porcellus_ENSCPOP00000017159'                                                                        ----------------
GTGGCAGCCTCCATAACCCACAGCCGGCTCAAGGCCAAGGGCGTGGGTGAGCACCACAACGCCTACAGCTTTCGCGGCCAGAGCTTCGAGAGCCTGCACGAGGCCTGCCTGAGGGAGAGGGAGCTGTTTGAGGATCCCCTGTTCC
CCGCCGAGCCTCGCTCTCTAGGCTTCAAGGACCTGGGTCCCAACTCCAAACAGGCACAGGACATCCACTGGCTGCGGCCCAAGGAGATCACACGCAATCCCCAATTCATTGTGGATGGGCCCACCTCAACGGACATCCGGCAGGG
GGGCGTGGGGGACTGCTGGCTGCTGGCTGCCATCGGCTCCCTGACCACGTGCCCCAGGCTGCTGTCCCGCGTGGTGCCCGGAGGCCAGAGCTTCGAGAAGAACTACGCTGGGATCTTCCATTTCCAGATCTGGCAGTTCGGGCAG
TGGCTGGACGTGGTGGTGGATGACCGTCTGCCCACCAGGGCCAACCAGCTGCTGTTTGTGCACTCGGAACAGCGCCAGGAGTTCTGGAGTGCCCTGCTGGAGAAGGCCTACGCCAAGCTGAGCGGGTCCTATGAGGCACTGGCAG
GGGGCAGCACCATAGAGGGCCTAGAGGACTTCACGGGTGGTGTGGCACTGACCTTCCAGCTCCAGAACCCCCCACCCAATCTGCTGTGGCTGATGCGCCGGGCCCTGGAGCACTGCTCTCTCATGGGCTGCTCTATTCAGGTCAC
CAATAACAGTGAGCTGGAGTCCGTGACGCCGAGCTTGCTGGTGAGGGGCCACGCTTACTCAGTGACTGGCATGGTGGAGATCCCCTACAGGGGCCGGACAGAGACGCTTCTGCGGCTGCTCAATCCCTGGGGCCGGATTGAATGG
AACGGAGCCTGGAGTGACAACTCTGCAGAGTGGGCCGAGGTGGACGACAGGATCCAGAGACAGCTGCTGCACAGGAAGGAGGACGGCGAGTTCTGGATGTCCTTCCACGACTTCCTGGGCCACTTCTCCTTCCTGGAGGTTTGCA
ACCTCACCCCCGATGCCCTGGCCGGGGACACCACGAGGCACTGGCACGTCACCTTCTATGAGGGCAGCTGGCGGAGGGGCAGCACTGCGGGGGGCTGCAGGAACAACCCTGACACATTCTGGACCAACCCCCAGTTCAGGGTCTC
ACTCTTGGTGGGGGATGACCCCGAGGATGACCCAGAAGATGACACGGTTGTCTGCACCTGCGTGGTGGCTCTGATGCAGAAGAACTGGAGGCTTGGCCAGCCACACGGGGCCACGCTGCTGACCATCGGCTTCGTCATCTTCCCG
GTTCCCAAGGAGTTTCAGAGGCTTCAGCACGGCCACCTAAGGAAAGACTTCTTCGCGCGGTACCCAGACCTCGGCTTTTCTGAGATCTTCACCACCGCAAGGGAGGTGCACAACCAGCTGCGGCTGTCGCCGGGCGAGTACATCA
TTGTCCCCTCCACCTTCGAGCCGCACCAGAACGCTGACTTCCTGCTTCGAGTCTTCACGGAGAAACACAGCGAGTCCTGGGAGCTGGATGAAGTCAACTACGCTGAGCATCTCCAGGAGGAGATTGTCTTGGAGCAGGACATGGA
CCAGAGCATCCTCAGTCTATTTGAATCGGTGGCC---
GGTGAGGACGAAGAAATAAACGTGCACAGGCTTCAAGTTCTGCTCAGCAGGTTGGCCTTCAGATTGAGCAACTTCAAGACCACGGGTTTTGGCCTGAATACCTGTCGTCATATGATCAACCTCATGGACAAAGATGGCTCGGGCA
AACTGGACCTTCTGGAGTTCCAGATCCTGTGGAAAAAGATCAGGAAATGGGTGGATGTCTTTCAAGAGTGCGACCAAGACCATTCGGGCACCCTGAACTCCTACGAGATGCGCCTGGCCGTGGAGAGAGCAGGCATTAAGTTAAA
CAACAAGGTGGCAGAGATCTTGGTGACTCGGTATGCAGATGACAACCTGACCGTGGACTTTAACAGCTTCCTCTGCTGTTTCCTGAGGCTGAAGGCTATGTTCATGTTCTTTCTCACCAGTGATCCAAATAACACTGGCTATATT
CGCTTAAACCTGGAGCAGTGGCTACAGATGACCATGTGGCAT
       'Ornithorhynchus_anatinus_ENSOANP00000015579'       -----------------------------------------------------------------------------
---------------------------------------------------------------------------------------------------------------------------------
---------------------------------------------------------------------------
GGTGACTGTTGGCTTCTGGCCGCCATCGGCTCCCTCACCCTCAACGAGGAGCTGCTGCACCGGGTGGTGCCCCACGGGCAGAGTTTCCAAGAGGAGTACGCGGGCATCTTCCACTTCCAGATCTGGCAGTTCGGAGAGTGGGTGG
ACGTGGTGATAGATGACCGCCTGCCCACCAAGGATGGGGAACTGGTGTTTGTCCACTCGGCGGAAGGCAGTGAGTTCTGGAGCGCCCTGCTGGAGAAGGCCTATGCCAAACTGAACGGCTCCTATGAGGCCCTGTCTGGCGGCAG
CACCACCGAGGGGTTCGAAGACTTCACCGGGGGCGTCGCCGAGATGTACGAACTTCGGCGGCCGCCCCGCAACCTCCTGCACATCATCCGCAAGGCTCTGGACCGAGGCTCGCTGCTCGGCTGCTCCATCGACATCACAGGTCTC
TTCGACATGGAGGCTGTGACATTCAAGAAGCTGGTGAAGGGGCATGCCTACTCGGTGACGGGGCTTAGAGAGGTTAACTATCAGAACCGGCAGGAGCCCCTCATTCGCATCCGTAACCCTTGGGGGCAGGTGGAGTGGACGGGGG
CATGGAGTGACAATTCCAATGAGTGGAATGAGATCGACCCTGATGAGCGGGACCAGCTGCAGCTCAAGATGGAGGATGGAGAGTTCTGGATGTCGTATCAATCATTGATAGATGCACTAGAGTTGGTAGATATTGTGTCCATTTC
CTTTTATCTACTCAAGCAGCAGTTAAATGCCAGTTATTTTTATGTGGGCACCGACCCTCCCCGCCGCCACCCCCGTACGAGCCCCTCCGTGCCTGTTCCCGCAGCCACGTTCTGGATCAACCCACAGTTCCGGTTGCGACTCCTG
GAGGAG---------
GATGACGACCCCGGGGACGACGAGGTGGCCTGCTCCTGCCTGGTCGCCCTGATGCAGAAGCACCGGCGCCGGGAGCGGCGTGTGGGCGGAGATATGCACACCATCGGCTTCGCCATCTACGAGGTCCCAGAGGAGTTGCAGGGCA
GCACGGGCGTGCACCTGAAGAAGGAGTTCTTCCTGCGGAACCAGTCCCGAGCCCGTTCCGAGACCTTCATCAACCTGCGGGAGGTGAGCGCCCACGTGCGGCTGCCCCCGGGAGAGTACGTCGTGGTGCCTTCCACCTTCGAGCC
CCACCAGGAGGCCGACTTTGTGCTGCGCGTCTTCACCGAGAAGCAGAGCGACACCCAGGAGCTGGATGAGGAGATCAGTGCAGATCTGCCTGATGAGGAGAACCTCTCCGAAGACGATGTAGACGAGTCCTTCAGAAGCATGTTT
GAGCAGCTGGCA---
GGAGAGGATCTGGAGATCAGCGTGTTTGAGCTGCGCAAGATCCTCAACAGAGTCGTCACAAGACACAAAGACTTGAAGACCGATGGCTTCAGCATGGAATCTTGTCGCAATATGGTCAACCTCATGGATAAAGATGGCAGTGCCC
```

```
GCTTGGGCCTGGTGGAATTCCAGATCCTCTGGAACAAAATCCGCAAGTGGTTGACCATCTTCCGCCAGCATGACCTGGACAAGTCGGGCACCATGAGCTCCTACGAGATGCGCCTGGCGGTAGAGTCAGCGGGCTTCAAGCTGAA
CAATAAGTTACAGCAAGTGCTGGTGGCTCGCTACGCCGATGACAACATGGGGATTGATTTTGACAACTTCGTGTCCTGTTTGGTCAAGCTGGAGGCCATGTTTCGATTCTTCTACGGTCTGGACCCTGAGGGCAGTGGTACCGCT
GTCATGGATCTGGGTCAGTGGCTGATCCTCACCATGTGCGGA
        'Anolis_carolinensis_ENSACAP00000001318'
ATGATGCCCTTTGGAGGAATGGCTGCCAGACTAGAAAGGGACCGTCTGCGAGCAGAGGGCGTGGGCAGTCACCAAAATGCTGTTAAATACCTGGGTCAGGATTACGAAACCCTGAAGCAGGAATGTTTGGAGAGTGGCCGATTGT
TTGAAGATCCCCAGTTTCCTGCCATCCCTTCTGTCCTTGGCTTCAAGGAACTGGGGCCAAATTCTGGCAAGACCCAAGGAGTGCGTTGGAAGCGCCCATCGGAAATTGTGGATGACCCACAGTTTATTGTGGGAGGTGCTACAAG
GACAGATATCTGCCAAGGGGCTCTGGGTGATTGCTGGTTACTGGCTGCCATTGGCTCCCTGACTCTCAATGAGGATCTTCTGCACCGAGTTGTGCCTCATGGCCAGAGCTTCCAGGAGGACTATGCAGGCATCTTCCACTTCCAG
ATCTGGCAATTTGGTGAATGGGTGGATGTGGTAGTTGATGACCGCCTGCCCACTAAAGATGGAGAGCTGGTATTTGTGCATTCAGCAGAATGTCAAGAGTTCTGGAGTGCTCTGCTAGAAAAGGCATATGCTAAGTTGAATGGCT
CTTATGAGGCCCTGTCAGGTGGCAGCACCACAGAAGGCTTTGAGGACTTCACAGGAGGCGTAGCCGAAATGTACGATCTTAAGAAGCCACCTAGGAACATTGCTCAGATCATTTGCAAGGCTCTCGAAAGGGGTTCCCTCCTTGG
CTGTTCCATTGATATCACCAGTGCATTTGATATGGAAGCAGTTACTTTCAAGAAACTAGTGAAAGGTCATGCCTATTCTGTCACAGGATTTAAGAATGTGGACTATCGTGGCCAACAAGAATCACTCATCCGCATAAGGAATCCC
TGGGGTCAGGTGGAGTGGACTGGAGCCTGGAGTGACAGCTCTTCTGAGTGGAATGAAGTGGACCCTGATCAGAGGGAAGAACTGCATCTAAAAATGGAAGATGGTGAATTCTGGATGTCTTTTCGAGAATTTATGCGTCAGTTTT
CCAGGCTGGAAATCTGTAACCTCACCCCAGATGCTCTGGACAAGGATGGGTTAAGCAGATGGCACACCACCCTCTTTGAGGGAACCTGGCGCCGTGGCAGCACAGCTGGAGGTTGCAGGAACCATCCAGCAACATTCTGGATTAA
TCCACAGTTCAAAATCAAGCTCCTAGAAGAG---------
GATGATGACCCAGAAGATGATGAGGTGGCTTGCACTTTCTTAGTTGCCTTGATGCAAAAACATCGCCGAAAAGCTCGCCGAGTTGGAGAAGATATGCATACCATCGGCTTTGCTGTCTATGAGGTTCCTGAGGAGGCACAGGGCT
GCCAAAATGTCCACCTGAAGAAGGATTTCTTTCTGAGAAACCAGTCTCAGGCTCGCTCAGAAACCTTTATCAACCTGCGGGAGGTGAGCAACCATATCCGCCTGCCTCCTGGTGAATACATCATCGTGCCCTCCACCTTCGAGCC
CAACAAGGAAGCAGACTTTGTCCTGCGTGTCTTCACAGAAAAGCAGTCTGATGCAGAGGAACTGGATGAGGAGATCTCTGCAGATCTTCCAGATGAGGAAGAAATCTCTGAGGACGATGTAGATGAGAACTTCAAAAACATGTTC
CGTCAGCTTGCA---
GGAGAGGACATGGAAATCAGTGTCTTTGAGCTCAGAACTGTCCTGAACAGAGTCATTGCCAGGCACAAAGACCTGAAGACAGATGGGTTCAGCATGGATTCTTGCCGCAACATGGTCAACCTAATGGATAAAGATGGCAGTGCTC
GTCTGGGACTTGTGGAGTTTCAGATCTTGTGGAACAAGATCAGGAGCTGGCTGAATATATTTCGTCAGCATGATTTGGATAAGTCAGGCACCATGAGCGCCTATGAGATGCGTCTGGCCTTAGAAACAGCTGGATTCAAACTGGA
CAACAAGTTGCATCAAGTGCTGGTGGCCCGATATGCTGACGACTCATTGGGTGTGGATTTTGATAACTTTGTCTCCTGTTTAGTGAAGCTGGAGACCATGTTCAGGTTCTTCCAAAGCATGGATCCTGAAGGTACTGGCAGTGCA
GTGATGAACCTTGGTGAGTGGCTGACATTCACAATGTGCGGC
        'Pelodiscus_sinensis_ENSPSIP00000019228'
ATGATGCCCTTTGGGGGAATTGCCGCCCGGCTGCAGAGAGACCGGCTGCGAGCGGAGGGAGTGGGTGAGCACCACAATGCCGTCAAGTACCTGAACCAGGACTACGAGGCTCTGAAGCAGCAGTGTCTGGAGAATGGCACACTCT
TCGAGGATCCCCAGTTCCCAGCTGTCCCCTCCGTCCTCGGCTTCAAGGAGCTGGGGCCAAATTCTGGCAAGACGCAGGGGTTGCGCTGGAAGCGCCCGTCGGAAATCGTGAGTGACCCTCAGTTTATTGTTGGAGGTGCCACGAG
GACGGACATCTGTCAAGGGGCTTTAGGTGACTGCTGGCTGTTGGCTGCCATTGGCTCCCTCACCCTCAACGAGGATCTCCTGCACCGCGTGGTGCCCCATGGGCAGAGCTTCCAGGAGGACTATGCTGGAATCTTCCACTTCCAG
ATCTGGCAGTTCGGCGAGTGGGTGGACGTGGTGGTTGATGACCGGCTGCCGACCAAGGACGGGGAGCTGGTGTTCGTGCACTCAGCAGAATGCACTGAGTTCTGGAGTGCACTGCTGGAGAAGGCCTATGCCAAGCTGAACGGCT
CCTATGAGGCCCTGTCGGGGGGCAGCACCACAGAGGGCTTTGAGGATTTCACCGGCGGCGTGTCGGAAATGTATGACCTCAAGCAGCCGCCCAGAGACCTATCCAGGATCATCCACAAGGCGCTGGAGAGGGGATCCCTGCTTGG
CTGCTCCATCGACATCACAAGTGCCTTTGACATGGAAGCAGTGACCTTCAAGAAGCTGGTGAAGGGACATGCCTACTCTGTCACAGGCTTTTCCGAGGTGGATTACCGGGGTCGCCAGGAAGAGCTCATCCGAATCAGGAACCCC
TGGGGTCAGGTGGAGTGGACTGGAGCCTGGTGCGACGGCTCCTCGGAGTGGAACAATGTGGACCCCTCGCAGAGGGAGGAGCTGCAGCTGAAAATGGAGGATGGCGAGTTCTGGATGGCTTTCAGGGACTTCATGAGGGAGTTCA
ACAGGCTGGAGATCTGTAACCTGACCCCTGATGCCCTGGACAAGGACAGCCTGAGCAAGTGGCACACAACGCTGTTCGAGGGCTCCTGGCGTCGAGGCAGCACAGCAGGGGGCTGCAGGAACCACCCAGCCACCTTCTGGATTAA
CCCCCAGTTCAAGATCAAGCTCCTAGAGGAG---------
GACGATGACCCTGAGGACAACGAGGTGGCCTGCAGCTTCCTGGTGGCCTTGATGCAGAAGCACCGGCGGAAGGAGCGGCGGGTGGGGGGCGACATGCACACCATTGGCTTTGCCGTCTACGAGGTCCCAGAGGAGGCCCAGGGCT
GCCAGAACGTGCACCTGAAGAAGGAGTTCTTCCTGCGGAACCAGTCCCGGGCTCGCTCCGAGACTTTCATCAACCTCCGGGAGGTGAGCAACAAGATCCGGCTGCCCCCAGGCGAGTACCTCATCGTGCCCTCCACCTTCGAGCC
ACACAAGGAATCTGACTTTGTCCTGCGCGTCTTCACGGAGAAGCAGTCTGACACAGAGGAGCTAGATGAGGAAATCACAGCGGATCTGCCAGATGAGGAAGAGATCTCCGAAGACGACGTGGACAGCTCCTTTAGGAGCATGTTC
CAGAAGCTGGCA---
GGAGAGGATATGGAAATCAGCGTGTTTGAGCTCAGGACTGTTCTGAACAGAGTCATCACCAGACACAAAGACCTGAAGACGGATGGGTTCAGCATGGAGTCCTGCCGCAACATGGTCAACCTGATGGATAAAGATGGCAGTGCCC
GCCTTGGCCTTGTGGAATTCCAGATCCTGTGGAACAAGATCCGGAGCTGGCTGACAATCTTCCGCGAGCATGACCTGGACAAGTCAGGCACCATGAGCGCCTATGAGATGCGCATGGCTCTGGAGTCAGCTGGCTTCAAGCTGAA
TAACAAGCTGCACCAGGTGGTGGTGGCCCGCTATGCCGATGCAGACTTGGGTGTGGACTTCGATAACTTCGTCTGCTGCCTGGTCAAGCTGGAGGCCATGTTCAGATTCTTCCGCAGCATGGACTCTGAAGGCACCGGCACAGCA
GTGATGAACCTGACTGAGTGGTTGCTGCTGAAAATGTGGGGC
        'Meleagris_gallopavo_ENSMGAP00000011308'
ATGATGCCCTTTGGGGGGATCGCTGCCCGGCTGCAGAGGGACCGCCTGAGAGCTGAGGGGGTCGGTGAGCACAACAATGCTATCAAGTACCTCAACCAGGACTATGAAGCCCTCAAGCAAGAGTGCATCGAGAGTGGTGTCCTCT
TCAGGGACCCCCAGTTCCCAGCTGGCCCCACTGCCCTCGGCTTCAAGGAACTGGGGCCGTACTCCAGCAAGACGCGGGGCGTGGAGTGGAAGCGTCCATCGGAATTAGTGGATGATCCTCAGTTCATTGTTGGTGGTGCAACCCG
GACAGATATCTGCCAGGGAGCTCTGGGTGATTGCTGGCTGCTGGCTGCCATTGGCTCCCTCACTCTCAACGAGGAACTCCTGCACCGTGTGGTGCCCCACGGGCAGAGCTTCCAGGAGGACTACGCTGGCATCTTCCACTTCCAG
ATCTGGCAGTTTGGTGAGTGGGTGGACGTGGTGGTGGACGACCTGCTGCCCACCAAGGATGGGGAGCTCCTGTTTGTGCATTCAGCGGAGTGCACAGAGTTCTGGAGTGCTCTGCTGGAGAAGGCCTATGCCAAGCTGAACGGCT
GCTACGAGTCGCTCTCGGGGGGCAGCACCACTGAGGGCTTTGAGGATTTCACTGGCGGCGTAGCAGAGATGTATGACCTCAAGCGGGCACCGCACAACATGGGCCACATCATCCGCAAAGCACTGGAGAGGGGGTCCCTGCTGGG
CTGCTCCATCGACATCACAAGTGCCTTTGATATGGAAGCAGTGACCTTCAAGAAGCTGGTGAAGGGCCATGCCTATTCTGTCACAGCTTTCAAAGATGTGAACTACCGGGGTCAGCAGGAACAGCTCATCCGTATCAGAAACCCT
TGGGGTCAGGTGGAGTGGACTGGAGCCTGGAGTGATGGCTCTTCCGAGTGGGACAACATTGACCCCAGCGACAGAGAAGAGCTGCAACTGAAGATGGAGGATGGAGAGTTCTGGATGTCTTTCCGGGACTTCATGAGGGAGTTCT
CCAGGCTGGAGATCTGCAACCTAACCCCCGATGCCCTCACCAAAGATGAGCTCAGCAGGTGGCACACACAGGTGTTTGAGGGCACATGGCGCCGAGGGAGCACTGCCGGGGGCTGCAGGAACAACCCAGCCACGTTCTGGATCAA
TCCCCAGTTTAAGATCAAGCTGCTGGAAGAG---------
```

GATGATGACCCTGGGGATGACGAGGTGGCCTGCAGCTTCTTGGTGGCCCTGATGCAGAAGCATCGTAGGAGGGAGCGACGAGTAGGGGGCGACATGCATACCATCGGCTTTGCTGTCTATGAGGTTCCTGAGGAGGCCCAGGGCA
GCCAGAATGTGCACTTGAAGAAGGACTTCTTCCTGCGAAACCAGTCGCGGGCACGCTCTGAGACCTTCATCAACTTGAGGGAAGTGAGCAACCAGATCCGGCTGCCCCCTGGCGAGTACATCGTTGTACCCTCCACCTTTGAGCC
ACATAAGGAGGCTGACTTCATCCTGCGGGTCTTCACTGAGAAGCAGTCAGACACTGCGGAGCTGGATGAGGAGATCTCTGCAGACCTGGCAGATGAGGAGGAAATAACCGAGGATGACATTGAGGATGGCTTCAAGAGCATGTTC
CAGCAGCTGGCA---
GGGGAGGACATGGAAATCAGCGTCTTTGAGCTCAAGACGATTCTGAACAGAGTGATCGCTAGACACAAAGATCTGAAGACGGATGGATTCAGTCTGGACTCCTGCCGCAACATGGTCAACCTGATGGATAAAGATGGCAGTGCCC
GCCTGGGACTGGTGGAGTTCCAGATCCTGTGGAACAAGATTCGCAGCTGGCTGACAATCTTCCGCCAGTATGACCTGGATAAGTCAGGCACCATGAGCTCATATGAGATGCGCATGGCTCTAGAGTCAGCTGGTTTCAAGCTGAA
TAACAAGCTGCATCAGGTGGTGGTTGCCCGTTACGCAGATGCTGAGACGGGTGTGGACTTTGACAACTTTGTCTGCTGCTTGGTGAAGCTGGAGACAATGTTCAGGTTCTTCCATAGCATGGATCCTGATGGCACTGGCACTGCC
GTCATGAACCTCGCTGAGTGGCTGCTGCTGACAATGTGCGGC
'Gallus_gallus_ENSGALP00000016538'
ATGATGCCCTTTGGGGGGATCGCTGCCCGGCTGCAGAGGGACCGCCTGAGAGCCGAGGGGGTCGGCGAGCACAACAACGCTGTCAAGTACCTCAACCAGGACTATGAAGCCCTCAAGCAAGAGTGCATCGAGAGTGGCACCCTCT
TCAGGGACCCCCAGTTCCCAGCTGGCCCCACTGCCCTCGGCTTCAAGGAGCTGGGGCCATACTCCAGCAAGACACGGGGCGTGGAGTGGAAGCGTCCATCGGAATTAGTGGATGACCCTCAGTTCATCGTTGGTGGTGCAACCCG
GACAGATATCTGCCAAGGAGCTCTGGGTGACTGCTGGCTGCTGGCTGCCATCGGCTCCCTCACTCTCAACGAGGAACTCCTGCACCGTGTGGTGCCCCACGGGCAGAGCTTCCAGGAGGACTACGCTGGCATCTTCCACTTCCAG
ATCTGGCAGTTTGGTGAGTGGGTGGACGTGGTGGTGGACGACCTGCTGCCCACCAAGGACGGGGAGCTCCTGTTTGTGCATTCAGCAGAGTGCACAGAGTTCTGGAGTGCTCTGCTGGAGAAGGCTTACGCCAAGCTGAACGGCT
GCTACGAGTCGCTCTCAGGGGGCAGCACCACTGAGGGCTTTGAGGATTTCACCGGCGGCGTAGCGGAGATGTATGACCTCAAGCGGGCACCGCGCAACATGGGCCACATCATCCGCAAGGCACTGGAGAGGGGGTCCCTGCTGGG
CTGCTCCATCGACATCACAAGTGCCTTTGATATGGAAGCAGTGACCTTCAAGAAGCTGGTGAAGGGCCATGCCTATTCTGTCACCGCCTTCAAAGATGTGAACTACCGGGGTCAGCAGGAACAGCTCATCCGTATCAGAAACCCC
TGGGGTCAGGTGGAGTGGACTGGAGCCTGGAGTGATGGTTCTTCCGAGTGGGACAACATTGACCCCAGCGACAGAGAAGAGCTGCAACTGAAGATGGAGGATGGAGAGTTTTGGATGTCTTTCCGGGACTTCATGAGGGAGTTCT
CCAGGCTGGAGATCTGCAACCTAACCCCCGATGCCCTCACCAAAGATGAGCTCAGCAGGTGGCACACACAGGTGTTCGAGGGCACATGGCGCCGAGGGAGCACTGCTGGGGGCTGCAGGAACAACCCAGCCACATTCTGGATCAA
TCCCCAGTTTAAGATCAAGCTGCTGGAAGAG---------
GATGATGACCCTGGGGATGACGAGGTGGCTTGCAGCTTCTTGGTGGCCCTGATGCAGAAGCACCGTAGGAGGGAGCGGCGAGTAGGGGGCGACATGCATACCATCGGCTTCGCTGTCTACGAGGTTCCTGAGGAGGCCCAGGGAA
GCCAGAATGTGCACTTGAAGAAGGACTTCTTCCTGCGAAACCAGTCGCGGGCACGCTCTGAGACCTTCATCAACTTGAGGGAAGTGAGCAACCAGATCCGGCTGCCCCCTGGCGAGTACATCGTTGTGCCCTCCACCTTTGAGCC
ACACAAGGAGGCCGACTTCATACTGCGGGTCTTCACCGAGAAGCAGTCAGACACAGCGGAGCTGGATGAGGAGATCTCGGCAGACCTGGCAGATGAGGAAGAAATAACCGAGGACGACATTGAGGATGGTTTCAAGAATATGTTC
CAGCAGCTGGCA---
GGGGAGGACATGGAAATCAGCGTCTTCGAGCTCAAGACGATTCTGAACAGAGTGATCGCCAGACACAAAGATCTGAAGACGGATGGCTTCAGTCTGGACTCCTGCCGCAACATGGTCAACCTGATGGATAAAGATGGCAGTGCCC
GCCTGGGGCTGGTGGAGTTCCAGATCCTATGGAACAAGATCCGCAGCTGGCTGACAATCTTCCGCCAGTATGACCTGGATAAATCAGGCACCATGAGCTCATACGAGATGCGCATGGCTCTAGAGTCAGCTGGTTTCAAGCTGAA
TAACAAGCTGCATCAGGTGGTGGTTGCCCGTTACGCAGATGCTGAGACGGGCGTGGACTTCGACAACTTTGTCTGCTGCCTGGTGAAGCTGGAGACAATGTTCAGGTTCTTCCACAGCATGGATCGTGATGGCACTGGCACCGCT
GTCATGAATCTCGCTGAGTGGCTGCTGCTGACAATGTGCGGC
'Taeniopygia_guttata_ENSTGUP00000008345'
ATGATGCCCTTTGGCGGGATGGCTGCTCGTGTGGAGAGAGACCGCTTGAGAGCCGAGGGGCTTGGTGAGCACAACAACGCCATCAAGTACCTCAACCAGGACTATGAGGCCCTCAAGCAGCAGTGCATTGAGAGTGGCACCCTCT
TCAGGGATCCCCAATTCCCAGCTGGCCCGTCTGCCCTTGGATTCAAGGAGCTGGGGCCACACTCCAGCAAGACACGGGGAGTAGAGTGGAAGCGTCCGTCGGAATTAGTGGATGACCCTCAGTTCATCGTTGGTGGTGCCACCCG
GACGGACATCTGCCAGGGGGCTCTGGGCGACTGCTGGCTGCTGGCTGCCATTGGCTCCCTCACGCTCAACGAGGAGCTCCTGCACCGCGTGGTGCCCCATGGACAGAGCTTCCAGGAGGACTATGCTGGCATCTTTCACTTCCAG
ATCTGGCAGTTTGGTGAGTGGGTGGATGTGGTGGTGGATGACCAGCTGCCCACCAAGGATGGGGAACTTCTGTTTGTCCACTCAGCGGAGTGCACCGAGTTCTGGAGTGCTGTGTTGGAGAAGGCCTATGCCAAGCTGAACGGCT
GCTACGAGTCACTCTCGGGGGGCAGCACCACCGAGGGCTTCGAGGATTTCACGGGCGGCGTGGCAGAGATGTACGACCTGAAGCGGCCGCCACGCAACATGGGCCACATCATCCGCAAGGCGCTGGAGAGGGGCTCCCTGCTGGG
CTGCTCCATCGATATCACAAGTGCCTTTGATATGGAAGCAGTGACTTTCAAGAAGCTGGTGAAGGGCCACGCCTATTCAGTCACAGCCTTCAGAGATGTGAACTACCGGGGTCAGCAGGAACAACTCATCCGCATCAGGAACCCC
TGGGGTCAGGTGGAGTGGACTGGAGCCTGGAGTGATGGTTCTGCTGAGTGGAACAACATTGACCCTGACGAGAGGGAAGAGCTGCAGCTGAAGATGGAGGATGGAGAGTTCTGGATGTCTTTCCGTGACTTCATGAGGGAGTTCT
CCAGGCTCGAGATCTGCAACCTGACCCCCGATGCCCTCACCAAGGACGAGCTCAGCAGATGGCACACACAGGTGTTCGAGGGCACGTGGCGCCGGGGGAGCACTGCCGGGGGCTGCAGGAATCACCCAGCCACATTCTGGATCAA
CCCCCAGTTTAAGATCAAGTTGCTGGAAGAA---------
GATGATGACCCTGGGGATGATGAGGTGGCCTGCAGCTTCCTGGTGGCTCTGATGCAGAAGCATCGACGGCGCGAGCGGCGGGTGGGAGGTGACATGCACACCATTGGCTTTGCTGTCTACGAGGTTCCTGAGGAGGCCCAGGGCA
TGCAGAATGTGCACTTGAAGAAGGACTTCTTCCTGCGAAACCAGTCCCGGGCACGGTCTGAGACCTTCATCAACCTGCGGGAGGTGAGCAACCAGATCCGGCTGCCCCCCGGCGAGTACATCATTGTGCCCTCCACCTTCGAGCC
GCACAAGGAGGCCGACTTCGTCCTGCGCCGCGAAAGGGAGAAGCAGGCAAGGGGTGGCTGTCTGAAAGAACAAAGAAGAGCTTCCTTTCCAGTTGGCGAGGAAATAACTGAGGATGACATAGAGGACAGCTTCAAGAACATGTTC
CAGCAGCTGGCA---
GGGGAGGACATGGAAATCAGCGTCTTTGAGCTTCGGACCATTCTGAACAGAGTCATCTCTAGACACAAAGATCTGAAAACAGATGGGTTCAGCCTGGACTCCTGCCGCAACATGGTCAACCTGATGGATAAAGATGGCAGTGCCC
GCCTTGGGCTGGTAGAATTCCAGATCCTATGGAACAAGATCCGGAGCTGGCTGACGATCTTTCGCCAGTATGACCTGGATAAGTCAGGCACCATGAGCTCCTATGAGATGCGCATGGCTCTGGAGTCAGCCGGTTTCAAGCTGAA
CAACAAGCTGCATCAGGTGGTGGTGGCGCGCTATGCGGACAATGACATGGGCGTGGACTTTGACAACTTTGTCTGCTGCCTTCTGAAGCTGGAGACCATGTTCAGGTTCTTCCGTAGCATGGACCCTGAAGGTACTGGCACTGCT
GTCATGAACCTTTCTGAGTGGCTGCTGCTGACGATGTGTGGC
'Westernclawfrog'
ATGATGCCGTTCGGGGGTATAGCATCGAAGCTGAAGATCGAGCGGCTGAAGGCGTCGGGCGTGGGCTCCCACGATTGTGCCGTTCGGTACCAGAACCAGGACTACGAGAGCCTGAAGCAGCAGTGCCGGGAGAGCGGCAGCCTCT
TCGAAGATCCAAACTTCCCGCCCATTGGCTCCTCCCTGGGCTTCAAGGAACTGGGCCCCGGAACCAGCAAAACTAAGGGGGTGCAATGGAAACGGCCCCATGATATTGCTGATGATCCGCAGTTTATTGTTGGTGGAGCCACCAG
GACAGACATATGCCAGGGGGCACTGGGGGACTGCTGGCTCCTTGCTGCAATTGGCTCCCTGACGCTCAATGAGGATTTGCTGCACAGGGTGGTTCCGCATGGGCAGGGCTTCCAGGATGACTACGCCGGGATCTTCCACTTCCAG
ATCTGGCAGTTTGGCGAGTGGGTGGATGTGGTGATAGACGACCGTTTGCCGGTGAAGGACGGGGAGTTGGTGTTTGTTCACTCGGCCGAGTGCTCGGAGTTCTGGAGCGCCCTGCTGGAGAAGGCCTACGCCAAGTTGAACGGTT

CCTACGAGGCCCTGTCGGGGGGCAGCACCACCGAGGGATTCGAGGACTTTACCGGGGGAGTCGCGGAAATGTACGAATTGCGCAAAGCCCCCCGGAACCTGGATAAGATCATTAAACGGGCGCTGGACAGGGGGTCCCTATTGGG
GTGCTCCATAGACATAACGAGTGCCTTTGACATGGAAGCCGTTACATTTAAGAAGCTTGTGAAGGGCCACGCCTACTCGGTCACGGCACTCAGAGAGGTTAATTACATGGGGGGGGTGGAAAAGCTGATCCGCATCCGGAACCCC
TGGGGGCAGGTGGAATGGACCGGCGCCTGGAGTGATAACTCCTCAGAATGGAATGAAGTGGACCCTTCAGAGCAGGAGGATCTGCAGTTAAAGATGGAGGATGGAGAGTTCTGGATGTCTTTCCAGGAATTCCTGCGCCAGTTTT
CCCGGCTGGAGATCTGTAACCTGACGCCGGACGCGCTGGACAAGGACGGGCAGAGCAAGTGGCACAGCACCCTGTACGACGGGACGTGGCGCCGGGGCAGCACCGCCGGGGGCTGCAGGAACAACCCAGCCACATTCTGGATTAA
CCCCCAGTTCAAGGTGACCCTGCTGGAGGAG---------
GACGATGACCCCGATGACAACGAGTTGGCCTGCAGTTTCCTGGTGGCGCTGATGCAGAAGAACCGGCGGAAGGAGCGCAAGGTCGGCCAGGACATGCACACCATTGGCTTTGCTGTGTATGAGGTTCCGGATGAGTTCCGGGGGT
GCCAGAATGTGCATCTGAAGAAGGATTTCTTCCTGCGCCACCAGTCCTGCGCCCGCTCCGAAACCTTCATTAACCTGCGGGAGGTGAGCACCCAGATCCGCCTGCCCCCCGGCGAGTACATCATCGTGCCCTCCACCTTCGAGCC
CCACAAGGAGGCAGATTTTGTCCTGAGAATATTCACTGAGAAACATTCAGATATACAGGAGCTGGATGAGGAGGTATCGGCAGATCTTCCTGAGGAGGAGGAACTCACAGAAGACGATGTGGACGATTCGTTTAAGGCCATGTTC
CGCCAACTGGCA---
GGGGAGGACATGGAGATCAGTGTGTTTGAGCTGAAGACGATCCTGAACAGAGTGGTGGCCAAACACAAAGATCTGAAGACGGACGGTTTCGGGATGGAGGCCTGCAGGCAGATGGTCAATCTGATGGACAAAGACGGCAGCGGCA
AACTGGGCGTGGTCGAGTTTCAGATCCTGTGGAACAAAATTCGCCAATGGCTGACAGCTTTCCGACAACACGACCTGGACAAGTCGGGCACCATCAGCGCCTACGAGCTGCGCATCGCCCTGGAGGGTACAGGGTACAAACTGAA
TAACAAACTCATTCAGGTTCTGGTCGCTCGCTACGCGGATTCCGACATGGGAATCGACTTCGATAACTTCGTCTGCTGCCTCGTGAAGCTGGAGGCCATGTTCCGGTTCTTCAAGGCGTTGGAC---
CAAGGGGAGGGCACAGCCGAGATGAGTTTAGGGGAGTGGCTGACGATGACCATGTGTGGA

'AfricanclawedfrogNP'

ATGATGCCGTTTGGGGGCATAGCATCCAAACTCAAGATCGACCGCCTGAAGGCATCTGGCGTGGGCTCACATGATTGCGCCATAAAGTACCAGAACCAGGATTATGAGAGTCTGAAACAGCAGTGTCTGGAGAGTGGCATCCTCT
TCGAAGATCCACATTTCCCAGCCACTCCCTCTTCCTTGGGCTTCAAGGAACTGGGCCCTGGATCCAGCAAAACTAGGGGAGTGCAATGGAAGCGACCCTCAGAAATTGTTGATGACCCACAGTTTATTGTTGGTGGAGCAACCAG
GACAGACATATGCCAGGGGGCACTGGGGGACTGTTGGCTCCTGGCTGCCATTGGCTCTCTGACGCTGAATGAGGATTTGCTGCACAGAGTGGTACCGCATGGGCAGAGCTTCCAGGAGGAATATGCTGGGATATTCCACTTCCAG
ATCTGGCAGTTTGGTGAGTGGGTCGATGTGGTGATAGATGACCGTTTGCCAGTAAAGGATGGAGAGCTGGTATTTGTGCACTCAGCCGAGTGTACTGAGTTCTGGAGTGGTCTACTGGAAAAGGCCTATGCCAAGCTGAACAGTT
CGTATGAGTCCCTGTCTGGTGGGAGCACCACTGAGGGATTTGAGGACTTTACCGGAGGAGTTGCAGAAATGTACGAATTACGCAAAGCACCTCGAGACTTAGATAAGATTATAAAACGTGCTTTGGAGAGAGGGTCCCTTCTGGG
ATGTTCAATAGATATAACAAGTGCCTTTGACATGGAAGCTGTCACATTTAAGAAGCTTGTGAAGGGACACGCATACTCAGTCACTGCTCTCAAAGAGGTTGATTACAGAGGAGGAATGGAAAAGCTCATTCGCATCAGAAATCCC
TGGGGGCAGGTGGAATGGACCGGAGCCTGGAGTGATAATTCGTCGGAATGGAATGAAGTGGACCCTTCAGAGCAGGAGGACTTGCAGCTGAAGATGGAGGATGGAGAGTTCTGGATGTCTTTCCAAGAATTCCTGCGCCAGTTTT
CCCGTCTGGAGATCTGTAACCTGACCCCGGATGCTTTGAATAAAGACGGGCTCGGCAAGTGGCACACCACTCTGTATGATGGCACTTGGCGCCGCGGAAGCACCGCCGGGGGCTGCAGGAACAACCCAGCCACATTTTGGATCAA
CCCTCAGTTCAAAATTACCCTGATGGAGGAA---------
GATGATGACCCTGATGACAATGAGTTGGCCTGCAGTTTCCTGGTGGCGCTGATGCAGAAGAACCGGCGGAAGGAAATGAAGGCAGGACAAGACACGCACACCATTGGCTTTGCTGTGTATGAGGTTCCTGATGAGTTTAAGGGCT
GCCAGAATGTGCATCTGAAGAAAGATTTCTTCCTACGCCACCAATCCTGTGCCCGCTCTGAAACCTTTATCAACCTGCGTGAGGTCAGCACGCAGATCCGCCTGCCCCCTGGGGAGTACATCATTGTTCCTTCGACCTTCGAACC
CCACAAGGAGGCAGACTTTATCATCCGAATATTCACTGAGAAACATTCAGATACACAGGAGCTGGATGAGGAGGTATCTGCAGATCTTCCTGATGAGGAGGAACTCACAGAAGACGATGTGGATGATTCATTCAAGGCCATGTTC
CAGCACCTAGCA---
GGAGATGACATGGAGATCAGTGTGTTTGAGCTGAAGACAATCCTGAACCGAGTGGTGGCCAAACACAAAGATCTGAAGACGGACGGTTTTGGTATGGAGGCCTGTAGGCAGATGGTCAATCTGATGGATAAAGATGGCAGCGGCA
AGCTGGGAGTTCTGGAGTTTCAGATCCTGTGGAACAAAATTCGGAATTGGCTCACTGTTTTCCGACAACACGACCTGGATAAGTCCGGCACCATCAGCGCCTATGAGCTGCGCATAGCCCTGGAGAGTACAGGGTACAAATTGAA
TAACAAGCTTATTCAGGTCCTGGTCGCTCGCTATGCGGATTCCGACATGGGCATCGATTTTGATAACTTTGTGTGCTGTCTAGTGAAGCTGGAGGCCATGTTCAGGTTTTTCAAGGCATTGGAC---
CAAGGCGATGGCACAGCAGAGATGAATCTGGGAGAGTGGCTGACGATGACCATGTGCGGA

'AfricanclawedfrogAA'

ATGATTCCGTTCGGGGGAATAGCATCTAAACTCAAGATCGACCGCCTGAAGGCATCCGGTGTGGGCTCACATGATTGCGCCGTGAAATACCAGAACCAGGATTATGAGAGTCTTAAACAGCAGTGTGTGGAGAGTGGCATCCTCT
TCGAAGATCCAAATTTCCCAGCCATCCCTTCTATCCTGGGCTTCAAGGAATTGGGCCCTGGATCCAGCAAAACTAGAGGCGTGCAATGGAAGCGGCCGTCAGAAATTGTTGATGACCCACAGTTTATTATTGGTGGAGCAACCAG
AACAGACATATGCCAGGGGGCATTGGGGGACTGTTGGCTCCTGGCTGCCATTGGCTCCCTGACGCTGAATGAGGATTTGCTGCACAGAGTGGTACCGCATGGGCAGAGCTTCCAGGAGGATTACGCTGGGATATTCCACTTCCAG
ATCTGGCAGTTTGGTGAGTGGGTCGATGTGGTGATAGATGACCTTTTGCCGGTAAAGGATGGGGAGCTGGTATTTGTGCACTCAGCTGAGTGTACGGAGTTCTGGAGTGCCCTGCTGGAAAAGGCCTATGCCAAGTTGAATGGTT
CATATGAGTCCCTTTCTGGTGGGAGCACCACCGAGGGCTTTGAGGATTTTACCGGAGGAGTTGCAGAAATGTATGAACTACGCAAAGCACCTCGAGACTTAGATAAGATAATAAAACGGGCATTGGAGAGAGGGTCCCTTCTCGG
ATGTTCAATAGATATAACAAGTGCCTTTGACATGGAAGCTGTTACATTTAAGAAGCTTGTGAAGGGACACGCATACTCTGTCACCGCTCTCAAAGAGGTTAATTACATGGGAGAAATGGAAAAGCTTATTCGCATCAGGAATCCC
TGGGGGCAAGTGGAATGGACTGGAGCCTGGAGTGATAACTCCTCAGAGTGGAATGAAGTGGACCCTTCTGAGCAGGAGGACCTGCGGCTAAAGATGGAGGATGGAGAGTTCTGGATGTCTTTCCAAGAATTCCTGCGCCAGTTTT
CACGTCTGGAAATCTGTAACCTGACCCCGGACACGCTAAATAAAGAGGGGCTGAGCAAGTGGCACACCACTCTGTACGATGGCAGTTGGCGCCGGGGAAGCACCGCCGGTGGCTGCAGGAACAACCCAGCCACATTCTGGATCAA
CCCTCAGTTCAAAGTCACTCTGCTGGAGGAG---------
GATGATGACCCTGATGACAATGAGTTGGCCTGCAGTTTCCTGGTGGCCCTGATGCAGAAGAACCGACGGAATGAGCGGAAGGCAGGACAAGACATGCACACCATTGGCTTTGCTGTATATGAGGTTCCTGACGAGTTTAAGGGCT
GCCAGAATGTTCATCTGAAGAAAGATTTCTTCCTGCGCCACCAATCCTGTGCCCGCTCTGAAACCTTTATCAACCTACGTGAAGTCAGCACCAAGATCCACCTGCCCCCTGGGGAGTACATCATTGTTCCCTCGACCTTCGAACC
GCACAAGGAGGCAGATTTTATCATGCGAATATTCACTGAAAAACATTCAGATATACAGGAGTTGGATGAGGAGGTGACAGCAGATCTTCCTGAAGAGGAGGAACTCACAGAAGACGATGTGGATGATTCATTCAAGGCCATGTTT
CAGCAGCTGGCA---
GGAGATGACATGGAGATCAGTGTGTTTGAGCTGAAGACCATCCTGAACAGAGTGGTGGCCAAACACAAAGATCTGAAGACGGATGGTTTTGGTATGGAGGCCTGTAGGCAGATGGTCAATCTGTTGGACAAAGATGGAAGCGGCA
AGTTGGGAGTTGTGGAGTTTCAGATCCTGTGGAACAAAATTCGAAAATGGCTGACTGTTTTTCGAGAACACGACTTAGACAAGTCCGGCACCATCAGCTCTTATGAGCTACGCATTGCCCTGGAAAGTTCAGGGTACAAACTGAA
TAACAAGCTCATTCAGGTTCTGGTCGCTCGTTACGCTGATTCCGACATGGGCATTGATTTTGATAACTTCGTGTGCTGTCTAGTGAAGCTGGAGGCCATGTTCAAGTTCTTCAAGGCATTGGAC---
GAAGGCGATGGCACAGCTGAGATGAATTTGGGGGAGTGGCTGACGATGACTATGAGCGGA

```
        'Oreochromis_niloticus_ENSONIP00000009131'
CCCTTGATGTTTACAGGAGTGTCCGAGCAGATCCAGATGGACCGGGCCCGAGCTGCGGGTCTGGGGTCGGTCCAGCAGGCCGTGCGCTACCTTAACCAGGACTTCGAGGCCCTGAGGCAGGACTGCCTGCAGAACAGAACCCTGT
TTGAGGACCCGATGTTCCCCGCAGAACCCGGCTCACTCGGCTTCAAGGAGCTCGCCCCATTCACGGCCAAAACCAGAGGAGTAGAGTGGAAGAGGCCAACGGAGCTGACACGAAACCCTCAGTTCATTGTGGGTGGAGCCACCAG
GACCGACATCTGTCAGGGCGCTCTGGGTGACTGCTGGCTGCTGGCTGCGATTGGCTCGCTGACGCTGAACGAGCAGCTGCTGCATCGCGTTGTTCCTCACGGTCAGAGCTTCAGCCACCAATACGCCGGTATCTTCCACTTTCAG
TTCTGGCAGTTTGGGCAGTGGGTGGACGTGGTGATCGACGACCGGCTGCCAGTCAAAGATGGCGAGCTGCTGTTCGTCCACTCGGCCGAAGGCACCGAGTTCTGGAGCGCCCTGCTGGAGAAGGCCTACGCCAAGTTGAATGGGT
CATACGAGGCGCTGTCCGGCGGCAGCACCACTGAGGGCTTCGAGGACTTTACGGGAGGCGTGTCAGAGATGTACGAGCTGAAGAAGGCGCCCAAAGATCTTCATCGCATCATCAGCAAAGCGCTAGAGAGAGGCTCGCTGCTCGG
CTGCTCCATCGACATCACCAACAGCTTCGACATGGAGGCGGTCACCTTTAAGAAGCTGGTCAAAGGCCACGCCTACTCTGTGACAGGCCTCAGACAGGTGGAGTACCGGCGCCAGCGCGAGCTGCTGATCCGGATCAGGAACCCC
TGGGGTCAGGTGGAGTGGACCGGAGCATGGAGCGATAACTCCTCGGAGTGGAACGCCATCGACTCGGCAGAGAAAGACGAGATGCTGTGTAAGATGGAGGACGGGGAGTTCTGGATGTCCTTCCAGGAGTTCCTGCGTCAGTTCT
CCCGGCTGGAGATCTGCAATCTGACTCCAGACGCTCTGAGCCAGGACTCCACCAGCTTCTGGAACACCGCCACGTACGAGGGCAGCTGGAGGAGAGGGAGCACCGCCGGGGGCTGCAGGAACCACCCCAACACCTTCTGGATCAA
CCCTCAGTATAAAATCTCCCTGCTAGAGGAG---------
GACGATGACCCCGAAGATGAAGAAGCTGCCTGCAGTTTCTTGGTGGCGCTGATGCAAAAAGACCGCCGCCGCTACCGCCGCCAAGGCCAGGACATGCACACCATTGGCTTTGCCATCTATGAGATCCCTGAACAGCACCGAGGCT
GTCCCAGCGTTCACATGAAGAAGGACTTCTTCCTGCGTCAGTCATCTTGCGCTCGCTCCCAGACCTTCATCAATCTGCGAGAGGTGAGCGGCAGGTTCCGCCTGCCGCCCGGCGAGTACCTGATTGTCCCATCGACCTTTGAACC
CTCGAAGGAGGCGGACTTTGTCCTGCGGGTATTCACAGAGAAGCAGTCTGAGAGCCAGGAGATGGACGACGGCGTGGTGGCGAACTTTGACGAAGAGGAGGAAGTGTCAGAGAGCGACATCGACGACTCCTTCAGGTCCATGTTC
GCTCAGCTGTCT---
GGAGACGACATGGAGATCTCAGTGCGGGAGCTCAGGACCATCCTCAACAGAGTCGTGTCCAAACACAGAGACCTGCAGACTGACGGCTTCAGCATGGAGTCCTGCAGAGCCATGGTCAGCCTCATGGACAAAGATGGCAGCGCTC
GCCTCGGGCTGCTGGAGTTTCAGATCCTCTGGAACAAGATCAGGAAGTGGCTGGGAATCTTCAGAGAGTTTGATCTGGATAAATCCGGCTGCATGAACTCCTACGAGATGCGTCTGGCGCTGGAAAATGGCGGCTTCAAACTGAA
CAACAAGCTGCATCAGATGCTGGTGGCTCGATACGCCGACAATGAGATC---
ATCGACTTTGACAACTTCACCTGCTGCCTCGTCAAACTGGAGGCCATGTTCCGAACCTTCCAGCACCTGGACACAGACGGGATGGGCACCGTGGAGATGAACCTCATCGAGTGGCTCTGTATGAGCATGTGCGGC
        'Gasterosteus_aculeatus_ENSGACP00000026345'
TACAGCATGTTTGGAGGCATATCTGGGCGGATCCAGAAGGACCGGCAGAGGGCTGGAGGTCTGGGCACGGTCCAGCAGGCGGTACCCTACTTGAACCAGAACTTCCAGGACCTGAAGGAAGACTGCCAGCAGAATGGATTCCTGT
TCCAGGACCCGGTTTTCCCAGCAGAACCGGCCTCACTGGGCTTCAAGGAACTGGGACCGTTTACCGCCAAAACCAGAGGGGTCGAGTGGAAGAGACCTACGGAGCTGACAGAGAACCCGCAGTTCATTGTGGGCGGAGCCACCAG
GACGGACATTTGTCAAGGCGCCCTGGGAGACTGTTGGCTGCTTGCTGCTATCGGATCTCTGACTCTGAACGAACGGCTGCTGCACCGCGTTGTTCCTCACGGTCAGAACTTCAGCCAGGAATACGCCGGCATCTTCCACTTCCAG
TTCTGGCAGTTCGGGGAGTGGGTGGACGTTGTCATTGACGACCGTCTGCCAGTCAAAGACGGAGAGCTGATGTTCGTGCACTCAGCTGAAGGCAGCGAGTATTGGAGCGCCCTGCTGGAGAAGGCCTACGCCAAGCTGAATGGCT
CCTATGAGGCGCTGTCCGGGGGCAGCACCACTGAGGGTTTTGAGGACTTTACTGGCGGCGTGTCGGAGATGTACGAACTGAGGAAAGCTCCCAGAGATCTTCATCGTATCATCAGTAAAGCTCTGGAGAGAGGGTCTCTGCTCGG
CTGCTCTATCGACATCACCAGTTCTCTGGACATGGAGGCGGTGACCTTCAAGAAGCTGGTGAAAGGTCATGCCTACTCCGTAACCGCGCTCCGACAGGTGGAGTACCATGGAAACATGGAGAAGCTGATCAGGATCAGGAACCCC
TGGGGTCAGGTGGAGTGGACTGGAGCCTGGAGCGACAGCTCCTCGGAGTGGACCTCTGTCGACCCGGCGGAGAAGGACGAGCTGCTCTGCCAGATGGAGGACGGAGAGTTCTGGATGTCTTTCCAGGATTTTTTGCGCCAGTTTT
CCCGACTGGAGATCTGTAACATGACCCCAGACGCTCTGAGCCAGGACTCCAGCTCGTTCTGGACCACAACCATGTTTGAGGGCGGCTGGAGGAGAGGGAGCACCGCGGGGGGCTGCCGGAACCATCCCAACACCTTCTGGATCAA
CCCACAGTATAAGGTGTCCCTTCTGGAGGAG---------
GATGACGATCCCACGGACGAGGAGCCCGCATGCAGCTTGCTGGTGGCTCTTATGCAGAAAGACCGCCGGCGCTACCGCCGCCATGGGCAGGACGTGCACACCATCGGCTTCGCCATCTACGAGGTTCCTGAAGAGTACCGAGGCC
GTGCCAGCGTGCACATGAAGAAGGACTTCTTCCTGCGCCATTCCTCCTGCGCCCGCTCAGAGACCTTCATCAACCTGCGGGAGGTGAGTGCCAGGCTCCGCCTCCCGCCAGGTGCAGTAAATGGTGGTGCCGTCAACCTACGACC
TTCCAAGGAGATGAACTTT---------------------------------------------------
GACCTTCTCGCCATTCTTCCCTGTAAGGAGGAGGTCTTGGTGAACGACGTGGACGAGTCCTTTCGGTCCATGTTCGCTCAGCTGTCT---
GGAGACGACATGGAGATCTCAGTCCAAGAGCTCAGGACCGTCCTCAACCGGGTCATGTCCAAACACGGAGATCTTCAGACGGATGGTTTCAGTATGGAGTCCTGCAGAGCCATGGTTAGCCTCATGGACAAAGATGGCAGCGCTC
GACTCGGCCTCCTGGAGTTCCAGGTTCTGTGGAACAAGATCCGGAAGTGGCTGGGAATCTTCCGTGAGTTTGATCTGGATAAATCCAGATGCATGAACGCCTACGAGATGCGCCTGGCGCTGGGAGAAGGCGGGTTCAAACTGAA
CAACAAGCTGTACCAGATGCTGGTCGCCCGATACGCCGACAACGAGATC---
ATCGATTTCGACAACTTCACCTGCTGCCTCGTCAAGTTGGAGGCCATGTTCAAGGCCTTCCAGGAGCTGGACAGAGATGGGACAGGACGTGTAGAGCTTAACATCTCCGAGTGGCTCTATGTGACCATGTGTGGA
        'Danio_rerio_ENSDARP00000046065'
ATGTTCTCATACGGCGGGATCTCAGCACACATCAGCGCTAACAGACTGAAGGCGGATGGCGCAGGCTCTTTTGAGCAGGCTTTACACTTCCAGAACCAGGACTACGAGGCCCTCAGACAGGAGTGTTTAGAGGGAGGCTACCTGT
TTGAAGACCCCTGTTTCCCCGCCGAACCGCCCTCTCTGGGCTTCAAAGAGCTCGCCCCGCACTCCTCCAAAACCCGAGATGTGGAGTGGATGCGCCCTACGGAGCTCTGTGATGATCCTCAGTTCATTGTTGGAGGAGCCACACG
CACAGACATCTGTCAAGGAGCTCTGGGTGACTGCTGGCTCCTCGCTGCGATTGGCTCGCTGACTCTTAATGAAAGACTCCTTCACAGGGTTGTCCCTCATGGCCAGAGCTTTCAAGATGACTATGCTGGAATTTTCCACTTTCAG
TTCTGGCAGTTCGGTGAATGGGTGGACGTTGTGATCGATGATCGGCTCCCGGTCAAAGACGGAGAGCTCATGTTTGTCCATTCAGCAGAGGGAAATGAGTTCTGGAGTGCCTTGGTGGAAAAAGCCTATGCTAAGCTAAATGGCT
CTTATGAAGCTCTTTCTGGAGGCTCTACCACTGAGGGTTTTGAGGATTTTACTGGAGGCGTTTCAGAAATGTACGAGCTCCGGAAAGCTCCAAGAGACTTGTACCGAATCATCAGCAAAGCCCTGGACAGAGGCTCCCTGCTGGG
CTGCTCTATTGATATCACCAGTGCCTTTGACATGGAGTCTGTGACATTTAAGAAGCTGGTAAAGGGTCACGCTTACTCCGTGACTGCTCTCAAACAGGTGGAGTACAGGGGGCGGATGGAGAGGCTTATCCGCATCCGTAATCCT
TGGGGTCAGGTAGAATGGACCGGAGCCTGGAGTGATAACTCTCCTGAATGGGATGAGATTGATCCATCAGAGAAGGACGACTTGCACTTACAAATGGAGGATGGAGAGTTTTGGATGTCATTTGGTGAGTTTTTACGTCAGTTCT
CCCGGCTGGAGATTTGCAATCTGACCCCTGATGCTCTGAGTGACGATGATATGAGCCACTGGAACACCATCAAGTTTCATGGAGCCTGGAGGAGAGGAAGCACTGCAGGAGGATGCCGAAATCATCCCAACACGTTCTGGATCAA
TCCACAGTATAAGATCACACTGCTGGAGGAA---------
GATGATGATCCGGAGGATGAAGAAGTGGCCTGCAGCTTCCTGGTGGCTTTAATGCAGAAAGACCGCAGACGGTACCGCCGCCACGGACAAGATATGCACACTATCGGTTTTGCAATTTATGAGGTTCCTGAAGAGTACACAGGCT
GTCAGAACGTGCACCTTAAGAAGGATTTCTTCTTGAGCAACTCATCAGCTGCTCGATCCGAGACCTTCATCAACCTGCGGGAGGTGAGCACTCGCCTGCGTCTGCCTCCTGGAGAATACATCATCGTCCCCTCCACCTTCGAGCC
CAGCAAAGAGGCCGATTTTGTCCTGAGAGTCTTCACCGAGAAACAGTCCGAGACCGAGGAATTGGATGACGAGATTTCAGCTGACCTGGAGGATGAGGAAGAAATTACAGAAGATGACATTGATGACTCCTTTAAGTCCCTGTTT
```

GCCCAGCTGGCT---
GGAGAGGATATGGAGATATCTGTTCATGAACTCAAGACTATTCTCAACAGAGTAGTCTCCAAACACAAAGATGTGAAGACAGACGGCTTCAGCATGGACTCCTGCAGAACGATGGTCAACCTCCTGGATAAAGATGGGAGCGCAC
GTTTGGGCTTAGTCGAGTTTCAGATCCTCTGGAACAAAATAAGGAAGTTGCTGGGGATCTTCAGAGAGTTTGATATTGATAAGTCAGGAACTATGAGCTCTTATGAGATGCGTCTCGCTGTAGAATCAGCAGGCTTTAAACTCAA
TAACAGACTCAACCAGATCCTGGTAGCCAGATATGCAGAGAACGAGGCC---
ATTGATTTTGATAACTTCGTCTGCTGCTTGATCAAACTAGAGGCCATGTTCAGATCTTTTCAACAGCTGGACAGAGAAGAAACAGGAACGGCTGAGATGAACCTATCTGAGTGGCTCTTCATGACCATGTGTGGT
'Danio_rerio_ENSDARP00000095870'
ATGTTTTCAATTGGAGGTGTGTCGACGCGCATCTACAAGGAGCGCCTGCAGGCAGAAGGGATGGGAGCGAATGACTTGGCCATCAAGTTCCTGAACCAGGACTATGAGGAGCTGAGAAGGGAGTGTGTGGAGAGTGGCAGGCTGT
TTGAGGACCCTTGCTTTCCTGCAGTGCCCCAATCACTGGGCTTCAAGGAGCTCGCGCCAAACTCCTCCAAAACACGTGGTGTGAAGTGGATAAGACCCACTGAGCTGTCAGAGAATCCTCAGTTTATAGTGGGCGGAGCCACGCG
TACAGATATTTGCCAGGGGGCCCTAGGTGACTGTTGGCTTTTGGCTGCCATAGCGTCGTTGACATTAAACGACAAGCTGCTTCACAGAGTTGTACCACATGGGCAAAGCTTTACAAATGAGTATGCTGGAATCTTCCACTTCCAG
TTCTGGCAGTTTGGCGAGTGGGTAGACATTGTAATCGATGACCGCTTGCCTGTGAAGGATAAAGAGCTGATGTTTGTTCACTCAGCAGAGGGAAATGAGTTCTGGAGCGCCCTGCTGGAGAAGGCATATGCTAAGCTAAATGGCT
CATATGAAGCTCTGTCCGGAGGTTCCACCACCGAAGGCTTTGAGGACTTTACTGGCGGAGTTGCCGAAATGTATGAACTGCGCAGCGCTCCCAAAGACCTGCACCGCATCATTGCCAAAGCCCTGGAGAGAGGCTCACTGCTGGG
CTGCTCCATTGATATCACCAGTGCCTTTGACATGGAGGCTGTGACCTTTAAGAAGCTAGTGAAGGGCCATGCTTACTCCGTCACAGCTCTCAGAGAGGTGAATTTCCGTGGCAACAGAGAGAGATTGATCCGTATCCGAAACCCT
TGGGGACAAGTTGAATGGACTGGGGCTTGGAGCGACAATTCGTCCGAGTGGAATGGGATTGATCCATCGGAAAGAGAGGAACTGAACAACCACATGGAGGATGGCGAGTTTTGGATGTCATTTCAGGAGTTCAAACGTCAGTTTT
CTCGTCTGGAGATCTGCAACCTCACTCCTGATGCTCTGAGTGATGATTCCCAACATTTCTGGAACACAATTCAGTTTAACGGCACTTGGAGGAAAGGAAGTACAGCAGGAGGCTGCAGGAACAATCCCAACACATTCTGGATAAA
CCCTCAATATAAGATCACACTACTGGAGGAA---------
GATGATGATCCTGAGGACAATGAGGTGGCCTGCACCTTCATGGTGGCTTTGATGCAGAAGGACAGACGGCGTTTCCGCAAGCAGGGACAGGACATGCACACCATCGGCTTCGCCTTATAT------------
GAGTTTCTTGGCTCCCAGAATGTTCACCTGAAGAAGGATTTTTTCTTGCGTCACTCGTCCTGTGCACGCTCCGAGACCTTTATTAATCTGCGAGAGGTGAGCACGCGACTGAGGCTGCCACCGGGCGAGTATCTCATTGTCCCTT
CCACTTTTGAGCCTGGAAAAGAGGCTGACTTCGTCCTGCGAGTATTCACTGAGAAACAGTCAGAAACTCAAGAAATGGATGATGAAATTTCATTTAACCTGGAAGATGAAGAGGCAGTCTCAGAGGAGAACATTGATGCCTCGTT
TAAAAAGATGTTTGCTCAGCTGTCT---
GGAGAGGACATGGAGATTTCAGTTCACGAGCTCCGGACCATTCTGAACCGAGTGATGACAAAACACCGAGACTTAAAGACAGATGGGTTCAGTTTGGAGTCTTGTAGGTGCATGATCAACCTGATGGACAAAGACGGCAGTGCAC
GACTGGGAATTGTGGAGTTCCAGATTCTGTGGAATAAAATCAGGAAGTGGCTGGGTGTCTTCAGACAGTTCGATCTGGATAAATCTGGAACTATGAGCTCCTACGAAATGCGCCTGGCTGTGGAGTCTGCAGGTTTTAAACTCAA
TAACAAGCTACATCAGATCCTGGTGGCCCGATATTCAGACGGAGATGTG---
ATTGACTTCGACAACTTTGTCTGCTGCCTGGTTAAACTGGAGGCCATGTTCAAGTCTTTCAAAGAGCTGGAGAAAGAGGGCTCAGGTGTCGCAGAGCTCAATATTAGCGAGGTCAGTCATTTCTTACTTTCTAAG
'Takifugu_rubripes_ENSTRUP00000021242'
ATGTATTCAGTTGGAGGGATCTCTGCAAGCATATATGCCAACAGGCTGCGAGCGGAGGGCATGGGCTCCAACGATCGTGCGGTGCCGTTCTGGAACCAGGACTACGAGGCACTGAAGCAGCAGTGTGTGGAGTCAGGGTGCCTGT
TCGAAGACCCCTGTTTCCCTGCCGAACCTCCATCCCTGGGCTTTAAGGAGCTGGCCCCGCACTCCTCAAAGACCCAGGGGGTGGAGTGGCAGAGGCCCACGGAATTGACAGATGATCCTCAGTTTATTGTGGGCGGAGCCACAAG
AACAGACATATGTCAGGGAGCCCTGGGTGACTGCTGGCTCTTAGCGGGCATCGCGTCTCTCAACCTGAATGAAAGGCTTCTGCACCGGGTTGTCCCGCATGGACAGTCCTTCCAGGATGACTATGCTGGCATCTTCCACTTTCAG
TTCTGGCAGTTTGGCGAATGGGTGGACGTTGTCATCGACGACAGACTACCTGTCAAAGACGGCGAGGTGATGTTCGTCCACTCGGCTGAGGGGAATGAATTCTGGAGTGCACTTCTGGAGAAAGCTTACGCCAAGCTGAACGGGT
CGTACGAGGCCCTGTCTGGAGGAAGCACCACGGAAGGGTTCGAGGACTTCACGGGCGGCGTCTCCGAGATGTACGAGCTGCGCAGCGCCCCAAAATTATTAGCAAAGCCCTGGACAGGGGCTCTCTGCTGGG
CTGCTCCATCGATATCACAAGTGCCTTTGATATGGAGGCTGTTACTTCAAGAAGCTTGTAAAGGGCCACGCCTACTCAGTCACTGGCCTGAAGGAGGTTAACTTCCGGGGCAGAATGGAGCGCCTGATCCGTGTACGTAACCCC
TGGGGCCAGGTGGAGTGGACCGGGGCCTGGAGTGACAACTCCTCTGAGTGGAATGAGATCGATCCTTCTGAAAGGGAAGATTTGCATCTAAAAATGGAAGATGGAGAATTTTGGATGTCCTTCAATGAGTTCAAGAAGCAGTTTT
CTCGCATAGAAATCTGTAATTTGACTCCTGACGCCCTGAGTGAGGACGCCCTCAGCCATTGGAACACAATGACCTACTCCGGCATGTGGAGGAGGGGGAGCACGGCAGGAGGCTGCAGGAACCATCCCAACACATTTTGGATTAA
CCCCCAATTTAAGATCACGCTCCTGGAAGAG---------
GATGACGATCCGGAGGATGACGAGGTGGCGTGCACTTTCTTAGTGGCTCTCATGCAGAAGGACCGCCGCAGATATCGGCGTCAGGGTCAGGACATGCACACCATCGGCTTCGCCGTGTACGAAATTCCAGAGGAGTTCAAAGGCT
GCCAAAATGTCCACTTGAAGAAGAACTTCTTTTTGAATCACTCGTCATGTGCACGCTCGGAAACGTTCATCAACCTGCGAGAGGTGAGCACACGTCTCCGCCTGCCCCCCGGAGAGTACCTGATCGTCCCCTCCACCTTTGAGCC
CCACAAAGAGGCCGACTTTGTCCTCAGGGTCTTCACGGAGAAACAGTCCGAGACAGAAGAAATGGACGATCAGATCTCTGCTGATTTAGGAGATGAAGATGAAATAAGTGAAGAAGACATCGACGACTCCTTTAAGTCCATGTTT
GCTCAGCTGGCA---
GGGGAGGACATGGAGATTTCAGTTCACGAACTAAAGACCATTCTAAACAGAGTTGTGACCCGGCATAAAGATCTGAAGACTGATGGCTTCAGTACAGTGTCTTGCAGAACCATGGTCAATCTCATGGATAAAGATGGCACTGCCC
ATCTGGGACTGGTCGAGTTTCAAATCCTCTGGAATAAGATCCGAAAATGGCTGGTCATTTTTAGACAGTTTGACCTGGACAAATCAGGGGCGATGAGCTCATACGAGATGCGTCTGGCTGTGGAGGCAGCAGGCTTTAAGCTGAA
CAACAAACTGAACCAGATTCTGGTAGCTCGGTACGCAGAGAACGACATG---
ATAAACTTTGACAACTTCATCTGCTGCTTGGTCAAGCTGGAGGCCATGTTTAGGCATTTCCAGCAGCTTGATAAAGAGGGTTCAGGAGTGGCTGAGATTAATATCACAGAGTGGCTTTACGTGACAATGTGTGGT
'Tetraodon_nigroviridis_ENSTNIP00000019733'
ATGCAACCAGTTGGGGGGATCTCTGCCAGGATATACGCCGAGCGGCTGCGTGCGGAGGGCATGGGCTCCAACCAGCAGGCCGTCCCCTTCTGCAGCCAGGACTACGAGGCCCTGAAGCAGCAGTGTGTGGAGTCAGGATGCCTCT
TTGAAGACCCCTTATTCCTCGCCCAGCCTGCTTCCCTGGGCTTCAAGGAGCTGGCGCCCCACTCCTCCAAGACCCGGGGGGTGGAGTGGCAGAGGCCCACGGAATTGACAGGTGATCCTCAGTTTATTGTGGGCGGGGCCACGAG
AACGGACATCTGTCAGGGAGCCCTGGGCGACTGCTGGCTCCTGGCGGCCATCGCGTCCCTCACCCTGAACGAGAGGCTCCTGCACCGGGTGGTCCCTCACGGACAGTCCTTCCAGGACGACTATGCCGGCATCTTCCACTTTCAG
TTCTGGCAGTTTGGCGAGTGGGTGGACGTGGTGATCGACGACAGGCTGCCCGTCAAAGACGGCGAGCTGATGTTCGTCCACTCTGCTGAGGGCAACGAGTTCTGGAGTGCACTCCTGGAGAAAGCTTACGCCAAGCTGAACGGCT
CCTACGAGGCTCTGTCTGGAGGAAGCACCACGGAGGGGTTTGAGGACTTCACGGGCGGCGTGTCGGAGATGTACGAGCTGCGCAGCGCCCCCAGAGATCTGGCCAAGATCATCAGCAAAGCCCTGGACAGGGGCTCTCTGCTGGG
CTGCTCCATCGATATTACAAGTGCCTTCGATATGGAGGCCGTCACTTTCAAGAAGCTGGTGAAAGGCCACGCCTACTCCGTCACCGGTTTGAAGGAGGTTAACTTCCGGGGCAGAATGGAGCGCCTGATCCGCGTACGGAACCCC
TGGGGGCAAGTGGAGTGGACCGGGGCCTGGAGTGACAACTCTCCTGAGTGGAATGAGATCGATCCTTCTGAAAGGGAAGACTTGCATCTGAAAATGGAAGATGGCGAGTTTTGGATGTCTTTCAACGAGTTCAAGAAGCAGTTTT

```
CTCGCATCGAAATCTGTAACCTGACGCCTGACGCGCTGAGTGAGGAGGCTCTCAGCCACTGGAACACGATGACGTACTACGGCATGTGGAGGAGGGGCAGCACGGCCGGAGGCTGCAGGAACCACCCCAACACGTTTTGGATCAA
CCCTCAGTTTAAGATCACGTTGCTGGAGGAA---------
GATGACGACCCCGAGGATGACGAGGTGGCGTGCAGTTTCTTGGTGGCCCTCATGCAGAAGGACCGCCGCAGATATCGGCGCCAGGGTCAGGACATGCACACCATCGGCTTCGCTGTGTACGAAATTCCAGAAGAGTTCAGAGGCT
GCCAGAATGTCCACCTGAAGAAAAATTTCTTTCTGAGCCATTCGTCGTGTGCGCGCTCCGAGACCTTCATCAACCTGCGGGAGGTGAGCACGCGCCTGCGCCTGCCCCCCGGGGAGTACCTGGTCGTCCCCTCCACCTTCGAGCC
CAACAAAGAGGCTGACTTTGTCCTCCGGGTCTTCACCGAGAAGCAGTCGGAGACAGAAGAAATGGACGACCAGATCTCTGCTGATTTAGGAGAAGAAGACGAAATAACCGAAGAAGACATTGACGACTCGTTTAAATCCATGTTT
GCTCAGCTGGCA---
GGAGAGGACATGGAGATTTCAGTCCACGAACTCAAGACCATTCTGAACAGAGTTGTGACCCGGCATAAAGATCTGAAGACCGATGGCTTCAGTACAGAGTCTTGCAGAACCATGGTCAATCTCATGGATAAGGATGGAAGTGCCC
ATCTGGGACTGGTCGAGTTTCAAATCCTCTGGAATAAGATTCGAAAATGGCTGGTCGTTTTTAGGCAGTTTGACCTGGACAAATCCGGGTCCATGAGCTCGTATGAGATGCGCTTGGCTGTGGAGGCAGCAGGCTTCAAGCTGAA
CAACAAGCTGAACCAGATTCTGGTAGCTCGGTACGCAGAGAACGATATG---
ATCAACTTTGACAACTTCATCTGCTGCCTGGTCAAGCTGGAGGCCATGTTCAGGCACTTCCAGCAGTTGGATGAAGACGGGTCAGGAGTGGTTGAGATGAATATCACACAGTGGCTTTACCTGACAATGTGCGGT
        'Oreochromis_niloticus_ENSONIP00000012531'
ATGTATTCGGTTGGAGGGATATCTGCAAGCATATATGCCAACAGGCTCCGGGCGGAAGGGATGGGCTCCAAGGACCAAGCTGTGCACTTTGCGGGCCAGGATTACGAGGCACTGAAGCAGGAATGTCTGGAGTCAGGCTGCCTGT
TTGAAGACCCCTATTTCCCTGCTGAGCCTCCATCCCTGGGCTTTAAGGAACTTGCCCCCTATTCCTCTAAAACAAAGGATGTGGAATGGATGAGGCCCACGGAACTAACACAAGACCCTCAGTTCATCGTTGGTGGTGCAACCAG
AACAGACATCTGTCAGGGAGCACTGGGTGACTGCTGGCTCTTAGCAGCCATTGCTTCTCTTACGCTGAATGAGAAGCTTCTTCATCGGGTTGTCCCACACGGTCAGTCCTTCCAAGATGATTATGCAGGAATCTTCCACTTTCAG
TTCTGGCAGTTTGGCGAATGGGTAGATGTCGTGATTGATGACAGATTGCCAGTGAAGGATGGGGAGCTAATGTTTGTCCATTCAGCTGAGGGCAATGAATTCTGGAGTGCACTCCTGGAGAAAGCTTATGCTAAGCTGAATGGCT
CCTATGAGGCTTTGTCTGGAGGGAGCACCACTGAAGGGTTTGAGGACTTCACAGGTGGTGTGTCTGAGATGTACGAGCTCCGCCGAGCTCCCAGAGATCTCTACAGGATAATTGGCAAAGCGCTGGAGAGAGGCTCCCTGCTGGG
CTGCTCCATCGATATCACCAGTGCCTTTGACATGGAGGCTGTTACGTTCAAGAAGCTTGTGAAAGGCCACGCCTACTCAGTCACTGGGCTGAAGGAGGTTAATTTCCATGGCAACAACGAACGCCTCATCCGAATACGTAACCCT
TGGGGCCAGGTGGAGTGGACTGGTGCATGGAGTGACAATTCTCCTGAATGGGATCAGATCGACCCCTCTGAACGAGAAGACTTGCATCTGCAGATGGAAGATGGCGAGTTTTGGATGTCCTTCAGTGATTTCATCAGGCAGTTTT
CTCGACTAGAGATCTGCAACTTGACTCCAGATGCTCTGAGTGACGACAGCCTGAGCCACTGGAACACCATCAAGTTCTACGGCACGTGGAGGAGGGGCAGCACTGCCGGGGGCTGCAGGAACCATCCCAACACGTTTTGGATCAA
CCCTCAGTATAAGATCACGCTGCTGGAGGAG---------
GACGACGACCCGGAGGACGATGAGGTGGCGTGCAGTTTTTTAGTAGCCCTCATGCAGAAAGACCGACGCAGATATCGACGTCAAGGTCAAGACATGCACACCATTGGCTTTGCTCTGTATGAGATTCCAGAAGAGTACAGAGGCT
GCCAAAATGTCCATTTGAAGAAGAATTTCTTTTTGAGCCATTCCTCCTGTGCTCGCTCGGAAACCTTCATCAACCTCCGGGAGGTGAGCACACGGCTCCGTCTGCCTCCAGGAGAGTACCTCATCGTTCCCTCCACCTTTGAGCC
CAGTAAAGAGGCTGACTTTGTCCTAAGGGTGTTCACTGAGAAGCAGTCAGAGACCGAAGAGCTGGATGATGAAGTCTCTGCCGATTTGGGAGAGGATGAAGAAATAACTGAAGATGACATCGATGACTCTTTTAAGGCCATGTTT
GCTCAACTAGCA---
GGAGAGGACATGGAGATTTCTATTCGCGAGCTTAGGACCATTCTAAACAGAGTTGTCACCCGGCACAAAGACCTGAAGACTGATGGCTTCAGTATGGAGTCCTGTAGGACCATGGTGAACCTGATGGATAAAGATGGTAGTGCTC
GTTTAGGGCTCGTGGAGTTTCAGATCCTCTGGAACAAGATCCGAAAGTGGCTGGTCATTTTCAGACAGTTCGACCTCGACAAGTCAGGGGCCATGAGCTCATATGAGATGCGACTTGCTGTGGAAGCAGCAGGTTTCAAACTGAA
TAATAGACTCAACCAGATTCTGGTGGCCCGGTATGCAGAGAATGAGATG---
ATTGACTTTGACAACTTCATCTGCTGCTTGGTTAAGCTTGAAGCCATGTTTAGGTCTTTCCGAGACTTTGACAAGGCGGGATCAGGAGAGGCAGAGATGAACCTGACAGAGTGGCTTTATCTGACCATGTGTGGT
        'Xiphophorus_maculatus_ENSXMAP00000006029'
ATGTATCCGGTTGGTGGGATATCTGCAAGCATATACGCCAACAGGCTCCGGTCAGAGGGCATGGGTTCCAATGAGCAGGCCGTGCACTTTGCCAGCCAGGACTACGAGGCTCTGAAGCAGGAGTGCGTGGAGGCGGGCTGCCTGT
TTGAAGACCCCTGCTTCCCCGCTGAGCCCCCATCTCTGGGCTTCAAAGAGCTTGCACCCTATTCATCTAAAACCAGAGATGTGGAGTGGATGAGACCCACGGAACTGACAGGTGATCCCCAGTTTATTGTGGGTGGAGCCACCAG
AACAGACATCTGTCAGGGTGCTCTTGGCGACTGCTGGCTCTTGGCAGCCATTGGCTCTCTGACCCTGAATGAGAGGCTCCTTCATAGGGTTGTTCCACACGGCCAGTCCTTCCAAGATGACTACGCTGGAATCTTTCACTTCCAG
TTCTGGCAGTTCGGCGAGTGGGTAGACGTTGTGATTGACGATCGATTGCCTGTCAAAGACGGAGAGCTCATGTTTGTCCATTCAGCTGAGGGCAATGAATTTTGGAGTGCACTCTTAGAGAAAGCTTATGCCAAGCTAAATGGCT
CCTATGAGGCTCTCTCTGGAGGAAGCACCACCGAGGGCTTTGAGGATTTCACAGGGGGGGTGTCTGAGATGTACGAGCTGCGCAAGGCTCCCAAAGATCTCTACAGGATAATCAGTAAAGCCTTGGAGAGGGGCTCCCTGCTGGG
CTGCTCTATTGATATCACCAGTGCCTTCGACATGGAGGCTGTGACCTTCAAGAAGCTTGTGAAGGGCCATGCCTACTCTGTCACTGCATTGAAGGAGGTTGATTACGCTGGCAACATGGTGCGCCTTATCCGAATACGTAACCCC
TGGGGCCAGGTGGAGTGGACTGGAGCCTGGAGTGACAATTCCAGTGAATGGGATGAAATTGACCCATCTGAAAGGGAAGACCTGCATCTTAGCATGGAAGACGGAGAGTTTTGGATGTCCTTCAGTGACTTTTTGAGGCAGTTTT
CCCGACTGGAAATCTGTAACTTGACTCCGGACGTCCTGAGTGAGGATAGCCTCAGTCACTGGAACACTATGAAGTTCTACGGCACATGGAGGAGAGGCAGCACGGCCGGGGGCTGCAGGAACCATCCCAACACATTTTGGATAAA
TCCTCAGTATAAGATCACGTTGCTGGAGGAG---------
GATGATGACCCAGAGGATGATGAGGTGGCGTGCAGTTTCCTTGTAGCCCTCATGCAGAAGGACCGGCGCAGATACCGACGCCATGGTCAGGACATGCACACCATTGGCTTTGCTGTCTATGAAATTCCAGAGGAGTACAGAGGCT
GCCAGAATGTCCACTTGAAGAAAAATTTCTTCTTGAGTAATTCATCGTGCGCTCGATCTGAGACCTTCATCAACCTTCGTGAAGTGAGCACTCGGCTGCGACTGCCGCCCGGGGAGTACCTCATTGTGCCCTCCACCTTCGAGCC
TGGCAAAGAGGCCGACTTCGTCCTCAGGGTTTTCACCGAAAAGCAGTCAGAGACAGAGGAACTGGATGATGAAATATCTGCTGATTTTGGAGATGAGGAGGAAATAACGGAAGATGATATTGATGATTCATTTAAGTCCATGTTT
GCCCAACTAGCA---
GGAGAGGACATGGAAATCTCTATTCGTGAGCTCAGGACAATTCTCAACAGAGTGGTCACCCGACACAAAGATCTAAAGACTGATGGCTTCAGCATAGAATCATGCAGAACCATGGTCAATCTGATGGATAAAGATGGCAGTGCTC
GCCTGGGACTGGTAGAATTTCAGATCCTGTGGAACAAAATCCGAAAGTGGCTGGTCACTTTCAGAGAGTTTGATCTTGACAAGTCAGGAGCCATGAGCTCATATGAGATGCGACTCGCTGTGGAAGCAGCAGGCTTTAAACTGAA
TAACCGGTTGCACCAGATTCTGGTGGCGCGGTATGCAGAGAATGAACTG---
ATTGATTTTGATAACTTCATCTGCTGCTTGGTCAAGCTTGAAGCCATGTTCAGGTCTTTTCAGCAGCTAGACAAGGAGGGATCAGGAGCTGCTGAGATGAATCTCACAGAGTGGCTTTATCTGACCATGTGTGGA
        'Oryzias_latipes_ENSORLP00000014711'
ATGTATCCAGCTGGAGGGATTTCTGCTGCCATTTACAACGACAGGCTTCGCTCTGAGGGGATGGGCTCCAAGGACCACGCCGTGCTCTTCGCCAACCAGGACTTCGAGTCCTTGAAGCGGGACTGTTTGGAGTCGGGGTGCCTGT
TCGAAGACCCCTGCTTCCCTGCAGAACCTCCGTCTTTGGGCTTCAAAGAGCTCGCACCCCATTCCTCTAAGACCAGGGGTGTGGAATGGATCAGACCCACGGAGCTGACGAGTGAGCCCCAGTTCATCGTGGGAGGAGCCACCCG
```

AACGGACATCTGCCAGGGAGCGCTCGGTGACTGCTGGCTTTTAGCGGCCATCGCCTCCCTGACCCTGAATGAGAGGCTCCTTCACCGCGTTGTTCCACACGGCCAATCCTTCCAGGAGGACTACGCTGGAATCTTCCACTTTCAG
TTCTGGCAGTTTGGCGAATGGGTGGATGTTGTGATTGACGATCGATTGCCGGTGAAAGACGGAGAGCTGATGTTTGTGCATTCAGCTGAGGGCAACGAGTTCTGGAGCGCCCTCATGGAGAAAGCGTACGCCAAGCTGAATGGTT
CCTACGAGGCTCTGTCCGGAGGAAGCACCACCGAGGGCTTCGAGGACTTCACAGGCGGAGTGTCTGAGATGTACGAGCTGCGGAAAGCTCCCAGAGATCTGCACAGGATCATCTCCAAAGCTCTGGAGAGAGGCTCCCTCCTGGG
ATGCTCTATTGATATCACGAGTGCCTTTGACATGGAGGCCGTTACATTCAAGAAGCTGGTGAAGGGACACGCCTACTCAGTGACTGGACTGAAGGAGGTCGATTACCGCGGCAACATGGTGCGCCTCATCAGGATACGTAACCCC
TGGGGTCAGGTGGAGTGGACCGGAGCCTGGAGCGACAACTCCCATGAGTGGAATGATGTCGACCCGTCTGACCGAGAAGACTTACACCTGAAAATGGAAGATGGCGAGTTTTGGATGTCCTTCAGTGATTTTCTGAGGCAGTTTT
CCCGTCTGGAGATCTGCAACCTGACTCCAGACGCCCTGAGTGAAGATGCCCTCAGCCACTGGAACACCATGAAGTTCTACGGCACGTGGAGACGGGGCAGCACCGCCGGGGGCTGCAGGAACCACCCCAACACCTTTTGGATCAA
CCCGCAGTATAAAATCACGCTGCTGGAGGAA---------
GATGATGACCCAGAGGACGACGAGGTGGCGTGCAGCTTTTTAGTCGCCCTCATGCAGAAGGACCGCCGCAGGTATCGGCGCCACGGTCAGGACATGCACACCATCGGGTTTGCCCTCTATGAGATTCCAGAGGAGTACAGGGGCT
GTCACAATGTCCATTTGAAGAAAAACTTCTTCCTGAGTCACTCCTCATGCGCTCGCTCAGAGACCTTCATCAACCTGCGGGAGGTAAGCGCTCGGCTGCGTCTACCTCCTGGAGAGTACCTCGTTGTCCCCTCCACCTTTGAGCC
CAGCAAAGAGGCTGACTTTGTCCTCAGAGTCTTCACTGAGAAGCAGTCGGAGACAGAGGAGCTGGATGATGAGATTTCTGCTGACTTTGGAGATGAGGACGAAATAACGGAAGATGACATCGATGACTCTTTTAGATCCATGTTT
GCCCAACTAGCA---
GGAGAGGACATGGAGATTTCTATCCGCGAGCTAAAGACCATTCTCAACAGAGTCATCTCTCGTCACAAAGACCTGAAGACGGATGGATTTAGCTCAGAATCCTGTCGGACCATGGTCAACCTAATGGATAAAGATGGCAGCGCCC
GCTTAGGACTCTTGGAGTTTCAGATCCTCTGGAACAAGATCAGGAAGTGGCTTGGCATTTTCCGGGAGTTTGATCTCGACAAATCGGGGGCCATGAGCTCATATGAGATGCGTCTGGCTGTGGAGGGAGCAGGTTTTAAACTGAA
TAATCAGCTGAACCAGATTCTGGTGGCCCGCTATGCAGACAACGAGATG---
GTTGACTTTGATAACTTCATCTGCTGCTTGGTCAAGCTGGAAGCAATGTTTAAGTATTTCCAGGAGTTGGACAAAGATGGATCAGGAGTTGTTGAGATGAATCTCACAGAGTGGCTTTACCTGACCATGTGCGGC
'Gasterosteus_aculeatus_ENSGACP00000006578'
ATGTATCCAACCGGAGGGATATCTTCAGCCATACAGGCCAACAGGCTACGAGCTGACGGCATGGGCTCCAAGGAGCAGGCGGTGCTCTTCTCCAACCAGGACTACGAGACCCTGAAGCAGGAATGTTTGGAGTCCGGCTGCCTGT
TTGAAGACTCCTGTTTCCCCGCCGAGCCTCCATCACTGGGCTTCAAGGAGCTCGCCCCCTATTCCTCCAAAACTAAGGATGTGGTGTGGATGAGGCCCTCGGAACTGAATGACGATCCTCAGTTCATCGTGGGTGGGGCCACCAG
AACTGACATCTGTCAGGGAGCGCTAGGCGACTGCTGGCTCTTAGCTGCTATCGGCTCTCTCACCCTCAACGAGAGGCTTCTTCATCGGGTTGTCCCTCACGGCCAGTCCTTTCAAGATGACTACGCAGGAATCTTCCACTTTCAG
TTCTGGCAGTTCGGCGACTGGGTAGATGTTGTGATAGATGACAGGCTGCCTGTCAAAGACGGCGAGCTCATGTTTGTCCATTCTGCTGAGGGCAATGAATACTGGAGTGCGCTCCTGGAAAAAGCTTACGCCAAGCTGAGTGGCT
CCTACGAGGCTCTGTCCGGAGGAAGCACCACTGAGGGGTTCGAGGACTTCACAGGGGGGTGTGTCTGAGATGTACGAGCTCCGCAGTGCTCCCAGAGACCTGCACAGGATAATAGGCAAAGCCCTGGAGAGAGACTCTCTGCTGGG
CTGCTCCATCGATATCACCAGTGCCTTCGACATGGAGGCCGTTACATTCAAGAAGCTGGTAAAGGGCCACGCCTACTCGGTAACCGGAATGAAGGAGGTCCACTTCCGCGGCAACATGGAACGCCTAATCCGGATACGTAACCCT
TGGGGTCAGGTGGAGTGGACCGGTGCCTGGAGTGACAATTCTCCTGAATGGAACGAGATTGACCCGTCTGAACGAGAAGACCTGCATCTGAAGATGGAAGACGGGGAGTTTTGGATGTCCTTCAGTGAATTCAAGAGGCAGTTTT
CTCGGCTAGAGATCTGCAACTTGACTCCTGACACGCTGAGTGAGGACAGTCTCGGCAAGTGGCACACCATGAAGTTCTACGGCGCTTGGAGGAGGGGCAGCACTGCCGGAGGCTGCAGGAACAGTCCCAACACGTTTTGGATCAA
CCCTCAGTACAAGATCACGCTGCTGGAGGAG---------
GATGACGACCCGGAGGATGATGAGGTGGCGTGCAGTTTTTTAGTAGCCCTCATGCAGAAGGACCGCCGCAGATATCGGCGCCATGGTCAGGACATGCACACTATTGGCTTTGCTCTTTATGAGATTCCAGAAGAGTACAGAGGCT
GCCAGAATGTCCACTTGAAGAAAAATTTCTTCTTGAGCCATTCGTCTTGCGCTCGCTCCGAGACCTTCATCAACCTGCGAGAGGTGAGCACGCGGCTCCGCCTGCCCCCCGGAGAGTACCTCATCGTCCCCTCCACCTTTGAGCC
CAGCCAGGAGGCCGACTTTGTCCTCAGAGTCTTCACTGAGAAGCAATCAGAAACGGAGGAACTGGATGATGAAATCTCTGCCGATTTAGGAGATGATGATGTAATAACTGAGGACGACATCGATGACTCCTTTAAGTCCATGTTT
GCCCAGCTAGCA---
GGAGAGGACATGGAGATTTCTGTTCGTGAGCTTCGGACCATTCTAAACCGAGTCGTCTGCAGGCACAAAGATCTAAAGACCGACGGCTTCAGTGTGGAGTCATGCAGGACCATGGTCAACCTGATGGATAAAGATGGTAGTGCCC
GTTTAGGGCTTGTGGAGTTCCAGATCCTCTGGAATAAAATCCGAAAGTGGCTGGTCATCTTTCGAGACTTTGACCTCGACAAGTCAGGGGCCATGAGCTCATATGAGATGCGTCTTGCTGTGGAGGCTGCGGGTTTTAAACTGAA
TAACCGACTCAACCAGATTCTGGTAGCCCGGTATGCGGAGAACGAGATG---
GTTGACTTTGACAACTTCATCTGCTGCTTGGTCAAGCTTGAAGCCATGTTTAGGTCTTTCCAGCAGTTTGACAAGGAAGGATCAGGAGAGGCTGAAATGAATGTCACAGAGTGGCTTTACCTGACCATGTGTGGT
'SmallerSpottedCatshark_TranscriptomeContig69801' -----------------------------------------------------------------------------------------
------------------------------------------------------------------------------------------------------------------------------------------------------
------------------------------------------------------------------------------------------------------------------------------------------------------
------------------------------------------------------------------------------------------------------------------------------------
ATGTTCGTCCATTCAGCCGAAAACAATGAATTCTGGAGCGCGCTGCTGGAGAAGGCGTACGCCAAGCTCAGTGGATCGTATGAAGCCCTGTCCGGGGGTAGTACCACTGAAGGATTTGAAGACTTCACCGGAGGTGTGGCTGAGA
TGTATGACCTGGGCAGCGCTCCCCGTGATCTCTACACCATCATCCAGAAGGCAGTGGACAGAGGCTCACTGCTGGGATGCTCCATTGATATCACTGGTGCCTTTGATATGGAAGCCGTGACCTTCAAGAAGCTGGTCAAGGGTCA
CGCCTACTCGGTGACAGGGGTCAAAGAGGTTGACTATCGGGGAAGGAAGGAGCGACTGATCAGGATCCGAAATCCCTGGGGCCAGGTGGAGTGGACCGGAGCTTGGAGTGACAATGCATCGGAATGGAATCAAATCGATGAGGAT
GAGCGTGATGGAATG---
GTCCAGATGGAAGATGGGGAATTCTGGATGGCATTCCAGGAGTTCCTGAAACAGTTTTCCCGGCTGGAGATCTGTAACCTGACTCCCGACACGCTGCAGGATGACATGATGAAGAAGTGGAACATGTCTGCGTTCAACGGCTCCT
GGAGGAGGGGTAGTACTGCTGGGGGTTGCCGAAACCATGCAGCGACCTTTTGGATCAATCCGCAGTTTAAGATCACATTGGAGGAGGAG---------
GATGATGACCCCGAGGATAACGAGATTGCCTGCAGCTTCCTGTGCGCTCTGATGCAGAAAGACCGGCGTCGACACCGCAAACACGGCCAGGATATGCACACCATCGGCTTCGCTGTGTATGAGGTTCCCGATGAGTATCGTGGTT
GTCAGAACGTACATTTGAAGAAGGATTTCTTCCTGACTCACAGTTCGTGCGCTCGCTCAGAAACGTTTATTAACCTGCGAGAGGTTAGCAACCGCATCAAACTTCCTCCTGGAGAGTACGTGATCGTGCCTTCAACCTTCGACCC
CAGTCAGGAGGCCGACTTTGTCCTGAGGGTTTTCACAGAGAAGCAGGCGGAGTCCGAGGAGCTGGATGACCCGATCAGTGCTGACCTGGAGGAAGAGGATGATCTGACTGAAGATGATGTTGATGAAGGTTTCAAGGCCATGTTC
TCCCAGCTTGCT---
GGTCAGGACATGGAGATCTCTGTGTTTGAACTACGGACGATCCTCAATCGGGTGCTGTCCAGACATGGTGATTTAAAGACAGATGGATTCAGCTTGGATTCCTGTCGTGGGATGGTCAATCTCTTGGATAAAGATGGCAGTGGTC
GCTTGGGTGTGTTGGAGTTTCAAATCATGTGGAACAAGATTCGCAAGTGGCTGGGTATTTTCCGACAATATGACTTGGACAAGTCTGGCACCATGAGCTCGTATGAAATGAGATTGGCCCTGGAATCAGCAGGTTTTAAAATCAA

```
CAACCGAATCCACCAGCTGATAGTGGGGCGTTACGCTGAAAATGATGCT---TTGGATTTCGATAACTTCATCTCCTGTCTGGTTAGATTGGAAGCAATGTTCAGATCATTCAAAGCCCTGGAGCAAGAG---
GATGGCACGGTGGAGATGAATATTGTTGATTGGCTCAGTCTAACCATGTCCGGC
	'bamboo_dataset1_plus_2_contig12303'	------
ATGTTGATGGGCGTGTCAGGAAGACTTTACAAAGAGCGGTTAAAGCAGGAAGGAATGGGTTCTCATGAGAAGGCCCTGAGGTTCCTGAACCAGGATTACGAGGCTTTGAAGCAAGAATGTCTGGAGAATGGGACCCTCTTTGAAG
ACCCACAGTTTCCAGCTGTCCCCACAAGCATCGGATTCAAGGAACTCGCTCCACACAGCTCCAAAACCCGCGGCATCATCTGGAAAAGACCACAGGAGATCGCAGCTGACCCACAGTTCATTCTGGCTGGAGCATCTCGAACTGA
TGTTTGTCAGGGAGGACTCGGTGACTGCTGGCTGTTGGCAGCCATTGCCTCACTGACTCTGAACGAGAAAGTGCTTCACCGCGTCGTGCCTCATGGTCAGAACTTCCAGGAGGAATACGCAGGAATTTTCCACTTCCAGTTCTGG
CAGTTTGGGGAGTGGGTGGACGTGGTGATTGATGACCGGCTGCCCACCAAGGACGGAGAGCTGGTGTTTGTCCACTCGGCCGAGAACAATGAATTCTGGAGCGGACTCCTGGAGAAGGCCTACGCCAAGTTGAATGGTTCCTATG
AAGCCCTGTCAGGGGGCAGCACCACCGAGGGGTTTGAGGATTTTACCGGGGGGGTGGCAGAGATGTACGAACTCCGGAATGCTCCGCGGGATCTCTACACCATCATCAGGAAGGCCCTGGACAGAGGATCACTCCTCGGCTGCTC
CATCGACATCACCAGTGCTTTCGACATGGAAGCAGTGACCTTCAAGAAGCTGGTCAAAGGTCATGCCTACTCTGTGACTGGGGTCAAAGAGGTTGATTATCGGTCTCGGATGGAGAGATTGATCCGGATCCGGAATCCCTGGGGT
CAAGTGGAATGGACTGGAGCATGGAGTGACAATTCCTCGGAGTGGAATTATATTGACCAGGAAGAGCGTGAAGGGATG---
GTGCAAATGGAAGATGGAGAGTTCTGGATGGCCTTCCCTGAATTCCTGCGGCAGTTCTCGCGGTTGGAGGTGTGTAACCTGACACCGGACACACTGCAGGATGACCTGATGAAGAAGTGGAACATGGCCGTGTTTAATGGCTCCT
GGCGCCGAGGGAGCACTGCAGGGGGCTGCCGTAACCATCCCGCTACGTTCTGGATCAATCCGCAGTTTAAAATCAAGTTGGAGGAAGAG---------
GATGATGATCCTGATGATAATGAGCTTGCTTGCAGCTTCCTGTGTGCTCTGATGCAGAAGGATCGCCGTAGATACCGTCGCCATGGGCAGGACATGCACACCATCGGCTTTGCCATCTACGAGGTTCCAGATGAGTATCGCGGTT
GTCAGAACGTCCATTTGAAGAAGGATTTTTTCCTAACTCACAGCTCGTGTGCTCGATCCGAAACCTTCATTAATCTCCGTGAGGTCAGCAACCGCATCAAGCTGCCCCCTGGGGAATATGTGATTGTCCCGTCAACCTTTGACCC
CAGTAAGGAAGCTGACTTTGTCCTTCGGGTGTTCGCAGAGAAGAACGCTGACACAGAGGAGCTGGATGACCCAATCTCTGCAGACCTGGAAGACGAGGAGGAGATCACAGAGGATGATGTTGAAGAAAGTTTCAAGGCCATGTTC
CGGCAGCTCGCG---
GGTGAGGATATGGAGATCAGTGTATTTGAACTGCGGACTATTCTCAATCGAGTTCTCTCCAAACACCGTGATTTGAAAACAGATGGGTTCAGTCTGGATTCTTGCCGGGGAATGGTTAATCTCCTGGATAAAGATGGCAGTGGGC
GTCTTGGTGTGGTGGAGTTTCAGATCATGTGGAATAAAATTCGCAAATGGCTGGGTATTTTCCGACAGTATGATCTGGATAAGTCAGGAACGATGAGTTCCTATGAGATGCGCCTTGCCTTGGAGTCAGCAGGTTTTAAAATCAA
CAACCGAATCCACCAAGTGATCGTTGGCCGATACTCAGAGAATGATGTG---CTGGACTTCGATAACTTCATCTCCTGCCTCGTCAAACTGGAGGCCATGTTCCGGTCATTCAAAGCTCTGGAGCAGGAG---
GATGGGACAGTGGACTTGAATCTTGCTGAGTGGCTGAGTTTGACCATGTCGGGT
	'ElephantShark_TranscriptomeContig20333'	------
ATGCTGACAGGAGTCTCCGCTCGGATACAGAAAGACCGTCAGAAACGTGAAGGGATGGGATCGAATGAGAGAGCGCTGCGTTTTCTGAACCAGGATTACGGGGTGCTGCGTGGGGAGTGTCTGGAGAACTGCTGCCTCTTCGAAG
ACCCCCAGTTCCCCGCGATTGCCTCATCCATCGGCTTCAAGGAGCTTGGCCCGTACACCTCCAAAACCAGAGGCATCGTCTGGCAGAGACCCACTGAACTTGTTGCTGATCCACAATTCATCACCGATGGAGCAAGCAGGACTGA
TGTGTGCCAGGGAGCTTTGGGTGACTGCTGGCTCCTGGCTGCCATCGCGTCTCTGACCCTGAACAATGAGGTGCTGCACCGGGTCGTGCCACATGGACAGAGCTTCCAGGAGGAATATGCTGGAATATTCCATTTCCAGTTCTGG
CAGTACGGGGAGTGGGTGGATGTGGTGATTGACGACCAGCTGCCCACCAAGGATGGAGAACTGGTGTTCGTCCACTCGGCCGAAAACAACGAGTTCTGGAGCGCACTGGTGGAGAAAGCTTATGCCAAGCTGAACGGCTCGTACG
AGGCGCTGTCGGGCGGCAGCACCACGGAAGGGTTTGAGGATTTCACTGGCGGAGTGGCTGAGATGTATCAACTGAACAAAGCGCCACGAGACCTGTACTCCGTCATCAGGAAAGCCGTGGAGCGAGGCTCACTGCTCGGCTGCTC
CATTGATATCACTAGCGCCCACGACCAAGAAGCCGTGACCTTTAAGAAGTTGGTGAAAGGTCACGCTTACTCAGTCACCGGGGTGAAAGAGGTGGATTACCGCGGGCGCCAGGTCAGACTGATCCGAATCCGGAACCCATGGGGA
CAAGTGGAGTGGACAGGATCGTGGAGTGACGGCTCCTCAGAATGGGATCACATTGGCGACGATGAGAGGGATGGAATG---
GTGAACAAGGAGGACGGAGAGTTCTGGATGGATTTCTCTGAGTTCCTGCGGCAGTTCTCGAGGCTGGAGATCTGTAACCTGTCTCCGGACGCGCTGCAGATGGAGGAGGTCAGGAAGTGGCACACGGCTGTGTATGACGGGTGTT
GGAGACGGGGCAGCACCGCTGGCGGCTGCCGCAACAACCCGGCCACGTTCTGGATTAACCCACAGTTTAAGATCACTCTGTCGGAGGAG---------
GATGATGACCCGGAAGATGAGGAGTTGGCCTGTAGCTTCCTGTGCGCGCTGATGCAGAAAGACCGTCGTCGTTTTCGGCGCCAGGGGCAGGACATGCACACCATCGGCTTCGCCATCTACGAGGTCCCAGAGGAGTATCACGGCT
GTCAGAACGTGCACCTGAAGAAGGATTTCTTCTTGACCCACGGGTCCTGTGCTCGCTCCGAGACCTTCATCAACTTGCGTGAGGTCAGCTCCCGTATCAGGCTGCCCCCTGGAGAGTACATCATCGTCCCCTCTACCTTCGAGCC
CCGGCAGGAAGCTGACTTCGTCCTGAGGGTCTTCACTGAGAAACAAGCTGAGTCTGAGGAGCTGGATGATGAGATCACAGCAGATCTGGAAGATGAGCCAGAGATGTCGGAAGATGATGTGGACGAGAATTTCAAAAACATGTTT
CGTCAACTGGCT---
GGAGAGGACATGGAGATCAGTGTCTTTGAACTGAAGACGATCCTAAACCGGGTTCTGTCCCGACATCACGATCTGAAGACTGACGGCTTCTGTCTGGACTCGTGCCGAACAATGGTTAATTTGATGGACAAGGATGGCAGTGCTC
GGCTCGGAATCCTGGAGTTTCAGGTCCTTTGGAACAAGATTCGCAAATGGCTGGGCATCTTCCGACAGTTTGACCTGGACAAGTCTGGAACAATGAGCACTTACGAAATGAGACTTGCCCTGGAAGCTTCAGGATTTAAGCTGAA
CAATCGGATCCTCCAGTCCATCGTCGGGCGCTACGCGGACAATGATACC---ATCGACTTTGACAACTTCATTGGTGCCCTAGTCAAACTGGAGGTCATGTTCAGATCCTTCAAAGCTCTGGAGAAGGAG---
GATGGCACAATAGAGATGAACATCATGGAGTGGCTTACGCTGACCATGTCCAGC;
END;
```

## (*j*) CAPN12

```
#NEXUS


BEGIN TAXA;

	DIMENSIONS NTAX = 15;
```

```
        TAXLABELS

                'XENOPUS_TROPICALIS_ENSXETG00000002784'                    'XENOPUS_LAEVIS_BC154987'                    'SUS_SCROFA_ENSSSCG00000002967'
'CERATOTHERIUM_SIMUM_XM_004441492'           'HOMO_SAPIENS_AK127398'           'MUS_MUSCULUS_ENSMUSG00000054083'           'OVIS_ARIES_XM_004015671'
'MONODELPHIS_DOMESTICA_ENSMODG00000013388'              'ANOLIS_CAROLINENSIS_ENSACAG00000011727'              'TAKIFUGU_RUBRIPES_ENSTRUG00000010051'
'OREOCHROMIS_NILOTICUS_ENSONIG00000003718'          'XIPHOPHORUS_MACULATUS_ENSXMAG00000018623'          'GASTEROSTEUS_ACULEATUS_ENSGACG00000009801'
'DANIO_RERIO_ENSDARG00000010758' 'LEPISOSTEUS_OCULATUS_GENSCAN00000011248' ;

END;

BEGIN CHARACTERS;
        DIMENSIONS NCHAR = 1707;
        FORMAT
                DATATYPE = DNA

                GAP=-

                MISSING=?

                NOLABELS

        ;

MATRIX

 AAGCTCAGTTACCTAGGGCAGGATTACCACAAACTTGTCCAGGAATGTCTGCAGCAAGGGCGACTGTTTGAGGATCCCCACTTCCCTGCAGATGCAAAATCATTGGGGTACAATGAG---
GCAGTAACCAGAGGAATTCAATGGAGAAGACCACAGGAGATCTGCCAG------
CCTAAATTCATCACTGAAAACATGAAGTGGACAGATGTGTGCCAGGGACAACTTGGTAACTGCTGGTTTCTTGCTGCTGCTGCCTCCCTGACCCAGTACCCATTACTTATGGCACGTGTTGTTCCCCCCCAGCAGGGTTTT---
AAGGACCGGTATGTTGGAATATTCCATTTTCAGTTCTGGCAGTATGGAGAATGGGTGGACGTAGTGGTTGATGACAGATTGCCAGTGAAAAATGGGCAGTTGGTTTTTGTCAGCTCTGCACAGAAAAGTGAATTTTGGGCTGCTC
TTCTTGAAAAAGCGTATGCAAAGTTAAATGGATCCTATGAAGCTCTAAATGGGGGGTTTATGAATGAAGCATTTGTGGATTTCACTGGTGGATTGGATGAGACTGTGGATCTCAAGGTT------------
CTCTACCATCTGATTGAGAAGGCAGTGAAGAAAAGATCACTTATGGGA---
TTGACAACCCCAGAAGGACTAGTAAAAGGACACGCATATTCTATCATAGCAACTTGGAAGATGGAACAAAATGGGCGAACTATTCATCTGCTACGCCTGAGAAATCCATGGGGTAAAGTAGAGTGGAACGGACGCTGGAGTGATA
ACTCCCCCTTGTGGTCACAAGTTACCTTTGAACTCAGAGAAAAAATGCAGGTC---GAAGATGGAGAATTCTGGATGCAGATGGAAGACTTTCTACGTTTTTTTGATATTCTGGAGGTTTGCAACCTTACACCAGAGTCAATG--
--------------------TGGAATACAAACACCTTCTCTGGGAGATGGATGATGGGCCACAATGCTGGAGGGTGCCGAAATTACAGA------ACTTTCTGGACCAACCCACAGTACATAGTAACTCTA---------------
------------TGTACGTTACTGGTTTCCCTCATGCAGAAGAACAGACGCCAGTATAGACAGGACTTTCTGGTTGTGGGGTTTGAGATCTACCAGGTT------------
GCCAAGCGAGACGTTACTGAACGGTACCAGCTGCCTCCTGGACGATACATCCCAAGCACATTCCAACCCCATCAGGAATCTGATTTCATCTTAAGGGTCTTC-------------------------------------------
--GGGGTAATTGTCAGGTACACAGCACAT------------------GACAGAGAAATCAGCCCTGAAGAATTACAACGTATTCTGGTA---------
TTCCCTCTGATAAAAATTACAGATCACAATGAGTTTAGGCAACTGTGGTTCAAGATCAAGGAGTGGGAGATATTCACAAAATATGATAAAGACAGGTCAGGAACAATGGATGTTCAGGAACTGCGTCTAGCACTGGAAGCGGCT-
--GGATTTACACTGAACAACCAACTGGTAGAATCTCTGTGCCAGAAATATGGTGATGATGTAAGGCAGGTGGATTTTGATAGTTTTCTTTCCTGCTTGGCATACCTCGTCTGTGTTTTT---------

 ---------TATCTGGGACAGGACTACCATAAACTTGTCCAGGAATGTCTGCAGCAAGGACGTCTGTTTGAGGACCCCCTCTTTCCTGCAGATGAAAAATCATTGGGGTACAATCAG---
GGACAAACCAGAGGCATTCAGTGGAGAAGACCACAGGAAATCTGTCAG------
CCTCAATTCATCTGCGAAAACATGAAATGGACAGATGTGTGCCAGGGACAACTTGGTAACTGCTGGTTTCTTGCTGCTGCTGCCGCCCTGACTCAGTACCCATTACTTATGGCACGTGTTGTCCCCCCACAGCAGAGTTTT---
AAGGATAGATATGCTGGAATATTCCATTTTCAGTTCTGGCAGTATGGAGAATGGGTGGACGTAGTGGTTGATGACAGATTGCCAGTGAAGAATGGACAATTGGTTTTTGTCAGCTCTGCACAGAAAAGTGAATTTTGGGCATCTC
TTCTTGAAAAAGCGTATGCAAAGTTAAATGGATCATATGAAGCCCTGGATGGAGGGTTTATGAATGAGGCATTTGTGGATTTCACTGGTGGATTGGATGAGACTGTGGATCTCAAGGTC------------
CTCTTCCATCTGATTGAAAAGGCAGTAAAGAAGAGATCACTTATGGGA---
CTGCAAACCCCAGAGGGACTGGTAAAGGGACATGCTTATTCTATCATAGCAACTTGGAAGATGGAACAAAATGGCCGAACAATTCATCTGCTACGTCTGAGGAATCCGTGGGGTAAAGTAGAGTGGAACGGACGCTGGAGTGATA
ACTCTCCTTTATGGTCACAAGTTACCGTTGAACTTCAAGCAAAACTGCAGGCC---GAAGATGGAGAATTCTGGATACAGATTGAAGACTTTCTGCGCTTTTTTGATATCCTGGAAGTCTGCAACCTTACACCAGAATCAATG--
--------------------TGGAATACAAACACCTTCTGTGGAAGATGGATAATGGGGCACAATGCTGGAGGGTGCCGGAATTATAGA------ACTTTCTGGACCAACCCACAGTACATGGTGACTCTG---------------
```

------------TGTACATTACTGGTCTCTCTCATGCAGAAGAACAGACGCCAGATTAGACAGGACTTCCTGGCTGTGGGATTTGAGATCTACCAGGTT------------
GCCAAGCGAGATGTTACTGAACGGTACCAGCTGCCTCCTGGGAAATACATCCCAAGCACTTTCCAACCCCACCAGGAATCTGACTTTATCCTGAGGGTCTTC-------------------------------------------
--GGCACCATGGAAGAATTCACAGCTCACTTTACAAGACTGTCCGGGGACAGAGAAATCAGCCCTGAAGAATTACAAAAGATTCTGGTA---------
TTTCCTCTGATAAAAATGGTAGATCGCAATGAGTTTAGGAAACTGTGGACCAAGATCAAGGAGTGGGAGATATTCACAAAATACGACAAAGACAGGTCAGGAACTATGGATGTTCAGGAACTGCGGTTAGCGCTGGAAGCGGCT-
--GGATTTACACTGAACAACCAATTGGTAGAATCTCTGTGCCAGAGATATGGTGATGACGTTAGGCAGGTGGATTTTGATAGTTTTCTTTCGTGCCTGGCCTATCTCGTCTGTGTTTTT---------

---------TTTCGGGGCCAGAGCTACAGAGCAATCCAAGCAGCCTGCCTGGATGAGGGCATCCTGTTCCGAGATCCCTACTTCCCTGCTGGCCCTGATGCCCTTGGCTATGACCAG---
GAGAAGGCCAAAGGGGTGGAATGGAAGAGGCCCCATGAGTTCTGTGCT------
CCCCAGTTCATCTGTGAGGACATGAGCCGAACAGATGTGTGTCAGGGGAGCCTGGGTAACTGCTGGTTTCTCGCGGCCGCTGCCTCCCTCACCCTGTACCCCCGGCTCCTGTGCCGGGTGGTCCCCCCGGGTCAGGGTTTC---
CAAGATGGCTACGCAGGTGTCTTCCACTTCCAGCTCTGGCAGTTTGGACACTGGGTGGACGTCGTGGTGGACGACAGGCTGCCTGTGCGTGAGGGGAAGCTGGTGTTCGTGCACTCGGCTCAGCGGAACGAGTTCTGGGCCCCCC
TTCTGGAAAAGGCCTATGCTAAGCTCCACGGCTCCTACGAGGTGATGCGAGGCGGCCACATGAATGAGGCTTTCGTGGACTTCACAGGCGGCGTGGGCGAGGTGCTCTACCTGAGGCAA------------
CTCTTCGCCGCCCTGCGCCACGCCCTGGCCAAGGAGTCCCTCGTGGGT---
TACCGTACGGGAGACGGGCTGGTGAAAGGACACGCATATTCTGTCACAGGCACACACAAGGTGTCACTGGGCTTCACTAAGCTGCGGCTGCTGCGGCTGCGGAACCCGTGGGGCCGCGTGGAGTGGACCGGAGCCTGGAGCGACA
GCTGCCCGCGCTGGGACGAGCTCCCCACAGAGTGGCGAGATGCCTTGTTGGTG---GAAGACGGCGAGTTCTGGATGGAGCTGCAGGACTTCCTCTGCCACTTCGACACCGTCCAGATCTGTTCACTGAGCCCGGAGGTGCTG--
-------------------TGGCACATCCACACCTTCCAAGGCCGCTGGGTGCGTGGCTTCAGCTCTGGCGGGAGCCAGCCCGGTGCC------ACCTTCTGGACCAACCCCCAGTTCCGGCTGACGCTG---------------
------------TGCACTGTTCTCCTGTCACTCATCCAGCGCAACCGGCGGCGCCTGAGGCTCATGTACCTCACCGTGGGCTTCCACGTGTTCCAGATC------------
GCCCGCCGCGACGTAAGCCGCCGCTGCCGGCTGCGCCCCGGCCACTACGTGCCCAGCACCGCCCGCGCCGGCGACGAGGCCGACTTCACGCTGCGTGTCTTC-------------------------------------------
-----------------------------------GAGCCCTCAAGAGAGGAAGAACTCCGTGCGCCTCAACTCCAGACCTTGCTAAGCTTGGAGCCTATCGGGCTCCTGCGGTGCTTTGGG------
CACTTCCAGCAGCTCTGGGGCCACCTCCTGGAGTGGCAGACATTTGACAAATTCGACGAGGACGCCTCTGGAACCATGAACTCCTACGAGCTGAGGCTGGCCTTGAACGCAGCA---
GGCTTCCACCTGAACAACCAGCTGACGCAGGCCCTCACGAGCCGCTACCGGGACAGCCGTCTGCGTGTGGACTTTGAGCGCTTTGTGTCCTGTATGGCCCAGCTTCTCTGCCTCTTC---------

---------TTTCGGGGCCAGAGCTACGAGGCAATCCGAGTGGGCTGCCTGGATGAGGGGGTTCTCTTCTGCGATCCCTACTTCCCTGCTGGGCCTGATGCCCTTGGCTATGACAAG---
GAGAAGACCAAAGGGGTGAAATGGATGCGGCCCCATGAGTTTTGTGCT------
CCCCAGTTCATCTGTGAGGACATGCGCCGGACAGACGTGTGTCAGGGGAGCCTGGGTAACTGCTGGTTCCTCGCTGCCGCGGCCTCCCTCACTCTGTACCCCCGACTCCTGTGCCGGGTGGTCCCCCCTGGACAGGGTTTC---
CAGGACGGCTATGCAGGTGCCTTCCACTTCCAGCTCTGGCAGTTCGGCAGCTGGGTGGACGTCGTGGTGGACGACAGGCTGCCCGTGCGTGAGGGGAAGCTGATGTTCGTGCGCTCGGACCAGCGGAACGAGTTCTGGGCCCCGC
TCCTGGAGAAGGCGTACGCCAAGCTCCACGGCTCCTATGAGGTGATGCGAGGTGGCCACATGAATGAGGCTTTCGTGGACTTCACAGGCGGCGTGGGTGAGGTGCTCTACCTGAGGCAA------------
CTCTTCTCCGCCCTGCGCCATGCCCTGGCCAAGGAGTCCCTCGTGGGC---
TACCGAACAGAAGATGGGCTGGTGAAGGGACACGCATATTCAGTCACGGGCACGCACAAGGTGTCCCTGGGCTTCACCAATGTGCGGCTGCTGCGGCTGCGGAACCCATGGGGCCGCGTGGAGTGGACCGGGGCCTGGAGTGACA
GTTGCCCACGCTGGGACACGCTCCCCACCGAGTGGCGAGATGCCCTGCTGGTG---GAGGATGGCGAGTTCTGGATGGAGCTGCGGGACTTCCTCTGCCACTTCGACACCGTCCAGATCTGCTCGCTGAGCCCGGAGGTGCTG--
-------------------TGGCACATCCACACCTTCCACGGCCGCTGGGTGCGCGGCTTCAACTCTGGCGGGAGCCAGCCCGGCGCC------ACCTTCTGGACCAACCCCCAGTTCCGGCTGACGCTG---------------
------------TGCACTGTCCTCCTGTCGCTCATCCAGCGGAACCAGCGGCGCCTGAGGCTCACCTACCTCACCGTGGGCTTCCACGTCTTCCAGATC------------
NNNNNNNNNGACGTGAGCCGCCGCTGCCGCCTGCGCCCCGGCCACTACGTGCCCAGCGCCGCCCGCGCCGGCGACGAGGCCGACTTCACGCTGCGCGTCTTC-------------------------------------------
-----------------------------------GAGCTGGCAGGAGAGGAAGAACTCAGTGCTCCTCAGCTCCAGACCTTACTAAGCCTGGAGCCTCTCGGGGTGCTGCAGTGTTTTGGG------
CACTTCCAGCAGCTCTGGTGTCACCTCCTGGAGTGGCAGACATTCGACAGGTTCGACGAGGACGCCTCAGGAACCATGAACTCCTACGAGCTGAGGCTGGCACTGAATGCGGCA---
GGCTTCCACCTGAACAACCAGCTGACCCAGGCCCTGACCAGCCGCTACCGGGACAGCCGCCTGCGCGTGGACTTCGAGCGCTTCGTGTCCTGTGTGGCTCAGCTCACCTGCATCTTC---------

---------TTTCGGGGCCAGAGCTATGAGGCAATTCGGGCAGCCTGCCTGGATTCGGGGATCCTGTTCCGCGACCCTTACTTCCCTGCTGGCCCTGATGCCCTTGGCTATGACCAG---
GAGAAGGCCAAAGGCGTGAAATGGATGAGGCCCCATGAGTTCTGTGCT------
CCGAAGTTCATCTGTGAAGACATGAGCCGCACAGACGTGTGTCAGGGGAGCCTGGGTAACTGCTGGTTCCTTGCAGCCGCCGCCTCCCTTACTCTGTATCCCCGGCTCCTGCGCCGGGTGGTCCCTCCTGGACAGGATTTC---
CAGCATGGCTACGCAGGCGTCTTCCACTTCCAGCTCTGGCAGTTTGGCCGCTGGATGGACGTCGTGGTGGATGACAGGCTGCCCGTGCGTGAGGGGAAGCTGATGTTCGTGCGCTCGGAACAGCGGAATGAGTTCTGGGCCCCAC
TCCTGGAGAAGGCCTACGCCAAGCTCCACGGCTCCTATGAGGTGATGCGGGGCGGCCACATGAATGAGGCTTTTGTGGATTTCACAGGCGGCGTGGGCGAGGTGCTCTATCTGAGACAA------------
CTGTTCTCTGCCCTGCGCCATGCCCTGGCCAAGGAGTCCCTCGTGGGC---
TACCGCACAGAAGAGGGCCTGGTAAAGGGACACGCGTATTCCATCACGGGCACACACAAGGTGTTCCTGGGCTTCACCAAGGTGCGGCTGCTGCGGCTGCGGAACCCATGGGGCTGCGTGGAGTGGACGGGGGCCTGGAGCGACA
GCTGCCCACGCTGGGACACACTCCCCACCGAGTGCCGCGATGCCCTGCTGGTG---GAGGATGGCGAGTTCTGGATGGAGCTGCGGGACTTCCTCCTCCATTTCGACACCGTGCAGATCTGCTCGCTGAGCCCGGAGGTGCTG--
-------------------TGGCACGTCCACACCTTCCAAGGCCGCTGGGTGCGTGGCTTCAACTCCGGCGGGAGCCAGCCTAATGCT------ACCTTCTGGACCAATCCTCAGTTCCGTTTAACGCTG---------------
------------TGCACGGTCCTTCTGTCCCTCATCCAGCGCAACCGGCGGCGCCTGAGACTCACTTACCTCACCGTTGGCTTCCACGTGTTCCAGATT------------
GCCCGCCGCGACGTGACCCGCCGCTGCTGCCTGCGTCCAGGCCACTACGTGCCGAGCACCGCCCACGCCGGCGACGAGGCTGACTTCACTCTGCGTGTCTTC-------------------------------------------
-----------------------------------GAGCTGGCTGGAGAGGAAGAACTCAATGCCTCTCAGCTCCAGGCCTTACTAAGCCTGGAGCCTATCGGGCTCCTGCAGTGTTTCGGG------

CACTTCCAGCAGCTCTGGGGCTACCTCCTGGAGTGGCAGATATTCAACAAGTTCGATGAGGACACCTCTGGAACCATGAACTCCTACGAGCTGAGGCTGGCACTGAATGCAGCA---
GGCTTCCACCTGAACAACCAGCTGACCCAGACCCTCACCAGCCGCTACCGGGATAGCCGTCTGCGTGTGGACTTCGAGCGGTTCGTGTCCTGTGTGGCCCACCTCACCTGCATCTTC---------

---------TTTAAAGGCCAGAACTACGAAGCCATCCGAAGAGCTTGCCTGGATTCCGGGATCCTGTTTCGTGACCCTTGCTTTCCTGCTGGCCCTGATGCCCTTGGCTATGACAAG---
GAGAAGGCCAAAGGGGTGGAATGGAAGAGGCCCCATGAGTTTTGTGCT------
CCCCAGTTCATCTGTGAAGACATGAGCAGAACAGATGTGTGCCAGGGAAGCTTGGGAAACTGCTGGCTTCTTGCAGCTGCTGCCTCCCTCACACTCTACCCCAGGCTCCTGTACCGGGTGGTCCCCCCTGGACAAGGTTTC---
CAAGATGGCTACGCGGGGGTCTTCCATTTTCAGCTATGGCAGTTTGGCCGCTGGGTGGATGTGGTGGTAGACGACAAACTGCCTGTGCGTGAGGGGAAGCTGATGTTCGTGCGCTCAGAACAAAGGAACGAGTTCTGGGCCCCTC
TGCTGGAAAAGGCCTATGCCAAGCTCCATGGCTCCTACGAGGTAATGCGAGGAGGTCACATGAACGAGGCTTTTGTGGACTTTACAGGAGGCGTGGGTGAGGTCCTCTACTTGAGACAA------------
GTCTTTGCTGCCCTTCGCCACGCATTGGCCAAGGAGTCCCTTGTGGGT---
ATCCGCACAGATGAAGGGCTGGTGAAGGGACATGCTTATTCTGTCACAGGCACGCACAAGATGTCTCTGGGCTTCACCAAGGTGCGGCTGCTGCGGCTGAGGAACCCCTGGGGCCGCGTGGAGTGGTCCGGGCCCTGGAGTGACA
GCTGCCCACGCTGGGACATGCTCCCTTCTGAGTGGCGAGATGCCCTGCTTGTG---GAGGATGGCGAGTTCTGGATGGAGCTTCAAGACTTTCTCACGCACTTCAACACAGTGCAGATTTGTTCACTGAGTCCTGAGGTGTTG--
-------------------TGGCATATCCACATCTTCCAGGGCCGCTGGGTGCGAGGCTTCAACTCCGGTGGGAGTCAGCCCAGCGCT------AACTTCTGGACCAACCCCCAGTTCCGGCTGACACTG---------------
------------TGCACGGTCCTGTTGTCACTCATCCAGCGCAACCGCCGGTGTCTGAGGCTCACTTACCTCACTGTGGGCTTCCACGTGTTCCAGATT------------
GCCCGCCGCGACGTGAGCCGTCGCTGTCGCCTGCCGCCTGGCCACTACGTACCCAGCGCCTCGCGCGTAGGCGATGAAGCCGACTTCACTCTGCGCATCTTC-------------------------------------------
-----------------------------------GAGCTGGCTGGAGAGGAGGAACTCAACGCGCTTCAGCTGCAGACCTTAATAAGCCTGGAACCTATTGGGCTTGTGCAGTGTTTTGGG------
CACTTCCAGGAGCTCTGGGGCCATCTCATGTCATGGCAGACATTTGACAAGTTTGATGAAGATGCCTCTGGGACAATGAACTCCTGTGAACTGAGGCTGGCACTGACTGCTGCA---
GGCTTCCACCTCAACAACCAGCTGACCCAGTCCCTCACTAGCCGCTACCGGGACAGCCGGCTCCGTGTGGACTTCGAGCGCTTCGTGTGCTGTGCAGCCCGGCTCACCTGCATCTTC---------

---------TTTCGCGGCCAGAACTACAAAGCAATCCGAGCAGCCTGCCTGGATGAGGGGATCCTGTTCCGAGATCCTTACTTCCCTGCTGGCCCTGATGCCCTTGGCTACGACCAG---
GAGAAGGCCAAAGGGGTGGAATGGAAGAGGCCCCACGAGTTTTGCACT------
CCCCAGTTCATCTGTGAGGACATGAGTCGAACAGATGTGTGTCAGGGGAGACTGGGTGACTGCTGGTTTCTTGCGGCTGCTGCCTCCCTCACTCTGTACCCCCGACTCCTGTCCCGTGTGGTACCCCCGGGACAGGGCTTC---
CAACATGGCTACGCAGGTGTCTTCCACTTTCAGCTCTGGCAGTTTGGCCGCTGGGTGGATGTCGTGGTGGATGACAGGCTGCCTGTGTGCGAGGGGAAGCTGATGTTCGTGCGCTCTGATCAGCGGAACGAGTTCTGGGCTCCGC
TCCTGGAAAAGGCCTATGCTAAGCTCCACGGCTCCTATGAGGTGATGCGAGGCGGCCACATGAATGAGGCTTTTGTGGACTTCACAGGGGGTGTAGGTGAGGTGCTTTACCTGAAGAAGCCAAACCCT---
CTCTTCTCCATCCTGCGCCGTGCCCTGGCCAAGGAGTCCCTCGTGGGG---
TACCGGACAGACGACGGGCTGGTGAAGGGACACGCATATTCAGTCACGGGCACACACAAGGTGTCACTGGGCTTCACTAAGGTGCGGCTGCTGCGGCTGCGGAACCCATGGGGCCGAGTGGAGTGGAATGGGGCCTGGAGCGACA
GCTGCCCACGCTGGGATGCGCTCCCTACAGAGTGGCGTGATGCCTTGCTGGTG---GAGGATGGCGAGTTCTGGATGGAGCTGCTGGACTTCTTCCGCCACTTTGACACTGTCCAGATCTGCTCGCTGAGCCCTGAAGTGCTG--
-------------------TGGCACATCCACACCTTCCAAGGCCGCTGGGTGCGTGGCTTCAACTCTGGTGGGAGCCAGCCTGGTGCC------ACCTTCTGGATGAACCCCCAGTTCCGGCTGACGTTG---------------
------------TGCACTGTTCTCCTATCGCTCATCCAGCGCAACCAGCGGCGCCTGAGGCTCAAATACCTGACCGTGGGCTTCCACGTGTTCCAGATC------------------------------------------
CTGGGAAAGGGGCGCTAT-------------------------------------------------------------------------------------------------------------------------------
-----GAGCTGGCAGGAGAGGAAGAACTTGGCGCCCCTCAGCTCCAGATCTTGTTAAGCCTGGAGCCTATCGGGCTCCTGCAGTGTTTTGGG------
CACTTCCAGCAGCTCTGGGGCCACCTCCTGGAGTGGCAGACATTCGATAAGTTCGACGAGGACGCCTCTGGAACCATGAACTCCTATGAGCTGAGGCTGTCA---------------------
CACCCCGCCCCCGCACCCACAAAGCCCACACCTTACCGCTACCGTGACAGCCGCCTGCGTGTGGACTTTGAGCGCTTCGTGTCCTGCATGGCCCAGCTCATCTGCATCTTC---------

---------TTCCGGGGCCAGTGCTACCATGCAATCCAGGACTGCTGCCTGCAGGCAGGGACCCTCTTCCTGGATCCCTGCTTCCCCGCTGGGCCCTCTGCCCTGGGCTATGACCAG---
GAGAAGGCCAAGGGGGTGGAGTGGCTGAGGCCTCACGAGTTTTGCGAG------
CCCCAGTTCATCTGTGAAAACATGGATAGGACCGATGTGTGCCAGGGCAGTCTGGGCAACTGCTGGTTCCTGGCCGCAGCCGCCTCCCTCACCTTGTACCCGCGCCTCCTCCATCGGGTGGTGCCTCCCGGGCAGAGCTTT---
CAGTGGGGGTACGCTGGAGTCTTCCACTTCCAGCTCTGGCAGTTCGGCCACTGGGTGGACGTGGTGGTGGACGATCGGCTGCCCGTGCGGGATGGGAAGCTGCTTTTTGTTCGCTCTGCGCAGAGGGCCGAGTTCTGGGCTTCGC
TGCTGGAGAAGGCCTACGCCAAGCTCCACGGCTCCTACGAAGTGATGCGGGGCGGCCACATGAACGAGGCCTTCGTGGACTTCACGGGAGGCGTGGGGGAGGTCCTGTACCTGCAGCCC------------
CTCTTCTCGGCTCTCCGGCACGCCCTGGCCAAGGAGTCCCTCGTGGGC---
TACAGGACGGAGGACGGGCTGGTCCGAGGCCACGCTTACTCCGTCACCGGGACCCACAAGATAACTCTGGGCTTCGCTAAAGTGCGGCTCCTCCGGCTCCGCAACCCCTGGGGTCACGTGGAGTGGAGCGGGGCGTGGAGCGACA
GCTGCCCCCGATGGGCTGCCCTCCCTGAGGCATGGCGGGAGGCCCTGCTCGTG---GAGGACGGAGAATTCTGGATGGGGCTGGAGGACTTCCTGCAACATTTTAACACCGTCCAGATCTGCAGCCTGAGCCCCGAGGTGCTC--
-------------------TGGCACGTCCACACCTTCCAGGGCCGATGGGTTCGAGGCTTCAATGCTGGTGGGGGCCAGCCAGGCAAT------ACCTTTTGGACAAATCCCCAGTTCCAGCTAACACTG---------------
------------TGCACAGTGCTCCTCTCCCTGATCCAGAGGAATCGGAGGCGGCTCCGTGTCACCTACCTCACCGTTGGCTTCCACGTTTTCAGGATT------------
ATCCGCCGAGATGTCAGCCGTCGCTGCCGCCTGGCGCCGGGCCATTATGTTCCCAGCACTGCTCACGTGGGCGAGGAGTCTGACTTCACGTTCCGCATCTAC-------------------------------------------
-----------------------------------GAACTGGCTGGAGAAGAAGCCATCAGTACCGCCCAGCTGCAGGCCTTACTGAGCCTAGAGCCTATTGGGTTCATCCACTGTTTTGGGCAA---
GAGTTTCAGCAGCTTTGGTATCGACTCCAGGGGTGGCAGACATTCAACAAGTTCGATGCTGACAAATCTGGGACCATGAATTCACACGAGCTGAGGCTGGCACTCAATACAGCA---
GGCTTCCAGCTTAACAACCAGCTGACCCAGCTCCTCACCAGCCGATACCGCGACAGTCGCCTGCGGGTCGACTTGGACCGCTTCGTGTCCTGCGTGACCCAGCTCACCTGCCTCTTC---------

```
 ---------TACCATGAACAAAACTATCAGGAGCTGAAACGTCAGTGCCTCCAACAGCAACGTCTCTTCCTTGACCCCGAATTCAAACCTTGTGCAGAGTCGCTTGGGTACAAAGAA---
GTGAATATCCAAGGGGTGGTATGGAAGAGGCCAAAGGACATCTGCAAACATGGACCACATTTTATTTGTAAAGGAATGAATCGGACCGATGTCTGCCAGGGGCAACTGGGTGACTGCTGGTTCTTGGCAGCAGCCGCCTCCCTTA
CCCTGTACCCAGAACTCCTTTATCGGGTGGTTCCACAAGATCAACATTTCGGAGGCACTGAATATGCTGGCATCTTCCATTTCCAGTTCTGGCAGTATGGGCAATGGGTGGATGTGGTGGTGGATGACCTCCTGCCCACCATAAA
TAACGAGCTGCTTTTTGTGCGCTCTCCTGAACATGACGAGTTCTGGATGCCTTTGTTGGAAAAAGCCTATGCCAAACTGAATGGCTCCTATGAAGCTATGAATGGTGGCTACATGAATGAGGCCTTTGTGGACTTCACTGGTGGA
ATTGGGGAGACTCTGTCCCTAAAAATA------------CTCTTCAAGACCATTCGAGCAGCCCTGAGTAGGAACTCTTTGATGGGC---
AAACCAACCCCGGAAGGGCTGGTGAAGGGACATGCCTACTCCATCACTGGAATTCACAAGATTGATTTCGGGGAGAAGGTGGTGAGGCTACTGAGGCTGCGAAACCCCTGGGGCTACCAGGAATGGACCGGCCGCTGGAGTGACA
AATCACCACTGTGGTCCTCTTTGGACCCCGAACTCCTGAAGAAGCTTCACGTGGATAAAGATGGAGAATTTTGGATGCAATTGGTTGATTTTATACGTCATTTCGATGTCTTGGAGATCTGCCACTTTAGT-------------
-------------------TGGAACGTCAACTGCTTCCAAGGCTGTTGGATCAAAGGCTACACCGCTGGTGGATGCCAGACTTTCCAACCATGGACTTTTTGGATGAACCCTCAATTTCATGTTTCACTG---------------
------------TGCACCTTCCTTGTTTCTTTGATGCAGAGAGAACGGCGCAGGAGCAGGAAGGACTTCCTTCTCATCTGCTTTCAGATCTTCAGGGTG------------------
AGAGACATCACGAGATACCTCCAGTTACCCCCTGGAGATTATATCCCCAACACCCAGTCTCCTTTGGAAGAGGCAAACTTCACCCTCCGCATTTTC-------------------------------------------------
-----------------------------AAGTATGCAGGAGACCAAGAAGTGGATGCCAATCAATTCCAAAGCATTTTGAAT---------
TTTTCTAGAATCCAGCATTTCAGTACCACAGATTTTCAGCAGTTTTCCTGTAGGATGCAAGAATGGCAGATATTCCTGGCTTACGATGTGGACAGGTCTGGCAGCATGAACACCCATGAAATCCAGCTGGCATTGGATGCTGCA-
--GGATTCCACTTAAATAATCGGACAACAGAGGCCCTTGTGAAAAAATATGGCAACCCCTGGCTCCAGATAGACTTCGACAGCTTTGTTTCCCTGATGGTGCACCTCGAAAGTGTTTTCCTATCTATG

 ---------TTTAAAGATCAGGACTTCAAGAAGCTCCTGGAAGCGTGTGTGAAGTCAGGAGAGCTGTTCGATGATCCGGCTTTTCCAGCTGAGCAGAAGTCCATCGGC------------
GATCCAAAGAACGCCATAAAGTGGCTGCGCCCCAAGGAAATCGGTAAG------
GCTGTGTTCGTGGAGGGAACCACTGGAACCACTGACATCTGCCAGGGCCAACTGGGCAACTGTTGGCTGCTGGCCGCCCTGTCCTGCCTGACCATGCACCCCACCCTGTTTGTGAAGGTGGTGCCGCCGGGCCAGAGCCTG---
TCCCAGTCGTACGCTGGCATCTTTTATTTTCGGTTCTGGCAGTATGGTGAGTGGGTGGAGGTGGTTGTCGATGACAGGCTGCCAGTACGCGGAGGACGTCTGCTCTTCAGCTACTCTCACACTTGCAACGAGTACTGGAGCGCCC
TGGTGGAGAAGGCCTATGCCAAGTTAATCGGATGCTACGGCAGCCTGAAGGGAGGGAACATATCGGAGGGGATGGAGGATTTCACAGGAGGCATCGCCTACTCCACCAGGGTGTCTTCT---------
GTCCTGTGGAGGTCTCTGACAGCCGCCCTGTCTCGAGGCAGCCTGCTGAGC---
AAAGTGACAGGAGAGGGTCTGATAAAGGGCCACGCTTACGCCATCACCGACACCGTAAAGGTGCTGAACGCTTCGGTTGAGACTTTGCTGCTGAGGCTGAGGAACCCCTGGGGCTTTGTGGAGTACAGAGGACCATGGAGCGACA
AAGCCAAAGAGTGGGACGATGTGGACAAAGCCGAGAAGGAAAAGATTGAACTAAAGGAAGATGGAGAGTTCTGGATCAGCGTTGAGGATCTCTCCAGGCTCTTTGACGTGGTGGAGCTCTGCAGCGTGAATCCCGACACTCTGCC
CGTCCCTCCCCCCTCCACCTGGACTATCAGCGAACATGAAGGATTCTGGGTCCCGGGGAGCTCCGCCGGCGGCAGCCGCAAATACAAA------TCATTTTGGAAGAACCCCCAGTTCCAGCTGGTGCTC---------------
------------TGCACCGTGCTGGTGGAGCTGCTGCAGAAGAACCGCCGGCAG------GTCCACTTCCTCTACATAGCCTTCCACGTCTACAAGGTCGCCGCT------
GCTCAGAGAGGTGTTTGGCGGAAGCTGCTTCTGGACCCAGGGAGATACGTGGCCTCCACCTACAGACCCAATCAGTCCGGGGAATTCTTTGTCCGCATTTTT------------------------------------------
AAA---------------------------------GAGGCGGCCGGTGATGATAGAGTGAATGCTAAGGAGCTCATGGGGCTGTTCAACCTG------
CTGCCACTGATCTTTGGCGAGGATACGCGCCAAGCAGAAACCTTGCTGACCAGCCTGCGCCAACTGCAGATTTTCTTCCAGTTTGACGAGGACTCATCGGGCACCATGAGCCCCTTTGAGCTCAGCGCAGCCCTTGAAGCCGTC-
--GGGATGCAGTGCGACAGGAAGATAGTTGAGCTGCTGTCTGAGCGCTTTGCTTCTGGAGCCCTGCACGCGCCCTTCCACAGCTTTGTCTCATGTGTCACCCGGCTGCGCAGACTCTTC---------

 ---------TTCAAGGACCAGGACTTTGAAACTCTCCAGGCAGAGTGTCTGAAGTCCGGCGTGCTGTTCTCTGATCCAACCTTTCCAGCGGAGCAGAAGTCCATCGGA------------
GACCCTAAAAAGGCTATTCAATGGAAGAGACCCAAGGAAATCAGTAAG------
GCTGCGTTTGTCGAGGACACGACTGCAACCACTGATATCTGTCAGGGCCAACTTGGTGACTGCTGGCTGCTGGCCGCCCTATCCTGTCTGACCGCGCACCCCTCGCTCTTTGTGAAGGTGGTGCCACCAAACCAAAGCCTG---
AGCGACCGTTACGCAGGGATCTTCCATTTTAAGTTCTGGCAGTATGGTGAGTGGGTGGAGGTGGTTGTGGATGACAGGCTGCCAGTACGCGAAGGCCGTCTGCTCTTCAGCTACTCTCGCAGCCGCAATGAGTTTTGGAGCGCCC
TGGTGGAAAAGGCCTATGCCAAGTTAATCGGATCTTATGGGAGCCTGAAGGGAGGCAACATTTCAGAGGGGATGGAGGACTTCACTGGAGGCATCGCCCGCTCGCTGGAGATCTCTTCT---------
GTCCTCTGGAGGTCCTTGGCTGCTGCTCTGTCCCGAGGGAGCCTCCTCAGC---
AAGGTGACAGGAGAGGGGCTGATAAGGGGACATGCTTACGCCATAACTGACGCTGACAAGTTGACAAAAACATCAGATGAGATTTTGCTGCTGAGGCTGAGAAACCCCTGGGGTTTTATTGAATATTGCGGACCCTGGAGTGACA
AGGGCAAAGAGTGGGAGAACGTGGACCCGACAGAGAAGGAAAGGATCGAGGTGTGCGAAGACGGGGAGTTCTGGATCAGTGCCGAGGACTTCTGCAACCTCTTTGATGTCGTGGAGCTCTGCAGCGTCAATCCAGACTCTTAT--
-------------------TGGGCCATCACCGAACACGAAGGCTGCTGGCTGCCGGGGAGTTCTGCTGGTGGTAGCCGCAAATATAAC------ACATTCTGCAAAAATCCCCAGTATACGCTGGTCCTC---------------
------------TGCACGGTGCTGGTGGAGCTGCTGCAGAAAAACCGAAGGCAG------CTCAACTTCCTCTACATCGCCTTTCACGTCTATAAGGTT------------
GCTCAAAGAGGTGTGTGGAGAAAGATGCGTCTGGATCCAGGTACTTACGTGGTATCTACCTATCGACCCAACCTCCCCGGAGAGTTCTTCGTCCGCACTTTC------------------------------------------
AGA---------------------------------AAGCAGGCTGGCGATGACAGGCTGAACGTCAAAGACATCATGAAGCTGTTCAACCTT------
CTGCCTTTGATCTTTGGAGAGGATACTCGCCAAGCAGGAACCCTGCTGGCCAGTGTGCGCAATCTG---ATCTTTTCTCAGTTTGATGAGGACTCTTCTGGAAGCATGAGCCGCTTTGAACTCAGCTCAGCACTGCATTTTCCA-
--GAATTCCAGTGCGATAACAAGGTGGTTGAGCTGCTGTCTGAGCGGTTCACATCTGGAGAGCTGGACATGCCCTTCCACGGCTTTGTGTCATGTGTCACCAGGCTGCGCAAGCTTTTT---------

 ---------TTCCAGAACCAGGACTTCGACGCTCTGCTGCAGGAGTGTCTGAAGGATAAGAAGCTGTTTGCAGATCCCACTTTTCCTGCTGAGCAGAAGTCCATCGGG------------
AATCCAGCAAAGGAGATCAAATGGAAGCGACCCAAGGAAATCAGTGCA------
GCCTTGTTTGTGGAGGACACCATTGGGACGACAGATATCTGTCAGGGCCAACTGGGTGACTGCTGGCTGCTGGCTGCCCTCTCCGCGCTCACCGTCCATTCGAAGCTCTTTGCTAAGGTGGTTCCCCCCAACCAGAGCCTG---
TCAGATTCGTACGCAGGGATCTTCCATTTCAGGTTCTGGCAGTATGGTGAGTGGGTGGAGGTGGTTGTGGATGACAGGCTGCCAGTGCGCGAAGGCCGTCTGCTCTTCAGCTACTCTCGCACCCGCAACGAGTTTTGGAGCGCCC
TGGTGGAGAAGGCCTATGCCAAGTTGGTGGGGTCCTACGGGAGCCTGAAGGGCGGCAACATCTCGGAGGGCATGGAGGACTTCACGGGAGGCATCGCGTACTCTCTTCCTGTCGCGTCC---------
```

GTCCTCTGGAGGATCCTGACCGCCTCTCTGTCCAGAGGCAGCCTGCTCAGC---
AAGGTGACGGCGGACGGGCTGGTAAAGGGCCACGCCTACGCCATCACGGACACAAACAAGGTAGCAAAAGGACCGGAGGAGGTTCTGCTGCTGAAGCTGAGGAACCCGTGGGGTTTTGTTGAGTTTTGCGGCTCCTGGAGTGACA
AGTGCAAGGAGTGGGCGGACACGGAGCAGTCGGAGAAGCAGCGGATCAAGCTCCTGGAAGACGGGGAGTTCTGGATCAGCGCCAGTGACTTCAGCGCGATGTTTAACGTCGTGGAGCTCTGCAGTGTGAGTCCAGAGTCTCTG--
-------------TCCACCTGGACCATCAGCGAACACAAAGGCTCCTGGGTGTCAGGAAGCTCCGCTGGCGGCAGCCGCAAATACAAC------TCCTTCTGGAAGAACCCCCAGTTCGAGTTGGTGCTC---------------
------------TGCACGGTGCTGGTGGAGCTGCTGCAGAGAAACCGCCGGCAG------ATCAACTTCCTGTACATCGCTTTCCACGTCTACAAGGTT------------
GCCCAGAGGGGCATCTGGAGGAAGCTGCGTCTGGATCCGGGTCACTATGTCGGATCCACTTATCGGCCCAACCAGCCGGGAGAGTTCTTCATTCGCATTTTC-------------------------------------------
AAA----------------------------------
GAGAAGGCCGGCGATGACAGACTGAACGCTCAAGAGATCATGGAGCTGATCAACCTGTCCCGTCTGCCTCTGATCTTCGGAGAGGATACCCGCCAAGCAGAGATCGTGCTGGCCGACCTGCTCACTCTGCAGGTCTTTGTTCAGT
TCGATGAGGACTCTTCTGGAAGCATCAGCCCCTTCGAGCTCAGCTTGGCACTAGAAGCCGTC---
GGAATGACGTGTGACGGCAAAGTAGTCCAGCTCCTTTCTGAGCGCTTTGTGGGCGGAGAGCCTCACCTGTCCTTCCATGGCTTCGTCTCCTGTGTCTCGAGGCTGCGCAAACTCTTC---------

---------TTCATGGATCAGGACTTTCAGACTCTACTGGAGAAATGTCTGAAGTCTCGAACATTGTTTGCTGATCCCTGCTTCCCCCCCCAACAGACGTCCATCGGT------------
GATCCAAAAAAGGCGATCAAGTGGCGGCGACCCAAGGAAATTGGTGTG------
GCTGTGTTTGTGGAGGACACAATTGGGACAACTGACATCTGTCAGGGCCAACTGGGTAACTGTTGGTTGCTAGCAGCCCTGTCCTGTCTCACCATGCACCCCACCCTCTTTGAGAAGGTGGTGCCCCCCAACCAAAGCCTG---
TCGGAGAGCTATGCAGGGATCTTCCATTTTAGGTTCTGGCAGTATGGTGAGTGGGTGGAGGTGGTTGTGGACGACAGGCTGCCAGTACGCGAAGGCCGTCTGCTCTTCAGCTACTCTCACACCCGCAACGAGTACTGGAGCGCCC
TGGTGGAGAAGGCCTATGCCAAGTTAGTCGGATGCTACGGAAGCCTGAAGGGGGGCAACATTTCCGAGGGAATGGAGGATTTCACAGGAGGAATCGCGTACTCGCTGCAGGTCTCGTCT---------
GTCCTCTGGAGGTCTCTGACAGCCGCACTGTCTCGAGGGAGCCTGCTCAGC---
AAAGTGACCGGAGACGGGCTGGTTAAGGGCCACGCTTACGCCATCACCGACAGCGACAAGGTAACAAAAGCGTCGGATGAGACGTTGTTGCTGAGGCTGAGGAACCCCTGGGGCTTTGTTGAATATCGAGGACCCTGGAGTGACG
CGAGTAAAGAGTGGGATGACGTGGTCAAAGAAGAGAAGGAGAGGATTGACCTGCAGGAAGATGGAGAGTTCTGGATCAGCGCTGAGGACTTCAGCAGGCTGTTTAACGTTGTGGAGCTCTGCAGTGTGAATCCTGACAACCTG--
----------------ACCTGGAGTATCAGTGAATATAAAGGATGTTGGGTTTCGGGGAGCTCTGCTGGCGGTAGCTCCAAATACACC------TCGTTCTGGAAGAATCCTCAATTCCAGTTGGATCTC---------------
------------TGCACCGTGTTTGTGGAGCTGCTGCAGAAAAACCGCAGGAAG------GCCGACTTTCTTCACATCGCTTTCCACATCTACAAGGTT------------
GCTCAGAGGGGTTCGTGGAGGGAGATGCAACTGGACCCGGGGAACTACGTGCCCTCCACCTTCCAACCCAACCAGCCAGGAGAATTCTTTGTCCGCATTTTCAGGGCAGCCAATAGCGGCTTAGAATACCGTGCGTCTCGTTTGA
GA----------------------------------GAGGAGGCTGGCGATGAAAGACTGAACCCCAAGGAGCTCATGAAGCTGTTCAACTTA------CTTCCGTAT---------------------
CAAGGTGACAACGAAATG---------------------
ATCTTCTTACAGTTTGATGAGGACTCTTCTGGGACCATGAGCCCCTTTGAGCTCAGTGCAGCGCTGCAAGCCGTTGGTGGGATGCAGTGTGATGGAAAGGTGTTGGAGCTGCTGTGTGAGCGCTTTGCATCTGGAGAGCTTCACA
TGCCTTTCCATAGCTTCGTGTCATGTGTCACCAGGATGCAGAAGCTTTTT---------

---------TTCAAGGATCAAGACTATGAAGTCCTGCTGCAGACGTGCCTCAAATCCGCAGTTCTGTTCTCAGACTCGGTGTTTGCAGCGAATCAAAGCTCCCTCGGT------------
GATCCCAAGAAGGCAGTGAAGTGGCTGCGCCCTAAGGAAATCACCAGC------
GCCGTCTTTGTGGAAGGCACCATGGGCACCACCGATATCTGCCAGGGCCAGCTGGGTAACTGCTGGCTGTTGGCGGCCCTGTCCTGTCTCACCATGCATCCAACTCTATTTGTGAAAGTGGTTCCAGCTGGACAAAGCCTG---
AGTGAATCCTACGCTGGCATCTTCCGATTTAAGTTCTGGCAGTATGGTGAGTGGGTGGAGGTGGTAGTGGATGACAGGCTGCCTGTGAGAGAGGGCCGTCTGCTTTTCAGCTACTCTCGCACCAGCAACGAGTTCTGGAGCGCCC
TGGTGGAGAAAGCTTATGCCAAGCTGATTGGTTCTTATGGCAGTCTAAAGGGCGGGATTATCTCAGAAGGAATGGAGGATTTTACAGGAGGAATTGCGTACTCTCTTCCGGTGTCTTCG---------
ATGCTCTGGAAAGCCATCACCGCAGCCCTGGCCCGGAACAGCCTGCTAAGC---
ACTGTGACCCCAGAGGGTCTCATCAAAGGTCATGCCTATGCCATCACTGAAACAGGCAAGGTCAAGAAGGCTTCTGAAGAGGTCTTTCTGCTGAGACTGCGTAACCCCTGGGGATTTGTTGAGTACTGCGGACCCTGGAGTGACA
AGTGTAAAGACTGGGACTTCGTAGACAACACTGAGAAGACGAAACTTGAATTGAAAGAAGATGGAGAATTCTGGATTAGCATAGAGGACTTTTGCAGATTTTATAATACAGTGGAGATGTGCAGTGTGGATCCAGACTCTATG--
----------------TCCTGGACACTGAGCTTACATCAGGGAACCTGGGTCCCAATGTGCTCTGCTGGCGGCAGCCGACGATACCCA------
TCTTTCTGGAAAAACCCTCAGTATCAGATGATTCTGCACGATTATGGAGAGGGTGAGGAAGGATGCACAGTGCTGGTGGAGCTTCTCCAGAAGTACCGCAGACAA------
GTCAACTTCCTCTACATCGCGTTTCATATCTATAAGGTT------
CCTGGAAGCATCAGGAGCGTCTGGAGGAAAGTTCACCTGGAGCCCGGCAGTTACCTGGCCTCCACTTACAGACCCAACCAGCAGGGGGAGTTTTTCCTCCGCATTTAC-------------------------------------
-----AGG----------------------------------GAAAAGGCTGGAGATGAGAGAGTGAATGCTGTGCAGTTTATGAATCTGGTAAACTTG------
CTTCCTCTGATCTTTGGAGAAGATACTCGACAAGCAGAGAAGCTGCTCTCCTCCCTCCGCAATCTGCAGATCTTTTTTCAGTTTGATGAAGATTCATCAGGAACTATGAGTCCGTTTGAGCTCAGTTTGGCTCTGAATGCTGCA-
--GGTGTGGAGTGTGACAGCGTTGTGGTGCAGATGTTGTGGGAAAGGTTTGGCGCTGGTGAACAGTACCTGCCCTTCTATGGCTTTGTGTCTTGTGTTGCCAGACTCCAGGTGCTCTTT---------

---------TTTAAGAACCAGGAGTTCGCGAGCCTGCGCGCCCAGTGCCTGCGGTCCGGCTCGCTGTTCGAGGACCCGGAGTTCCCGGCTCAGCAGGACTCCCTGGGG------------
GACCCGGCCAAAGCCGTCGTGTGGCGTCGACCCAAGGAGATCAGCAAG------
CCCAAGTTTATCGAGGGCACAGCAAGTACCACCGACATCTGCCAAGGACAGCTGGGTGACTGCTGGCTGCTGGCTGCCCTGTCATGTCTCACTCTCCACCAGCCGCTGTTCAACAATGTGGTGCCCGGAGACCAGAGCCTG---
TCTGAAGAGTACGCTGGCATCTTCCACTTCAAGTTCTGGCAGTATGGGCAGTGGGTGGAGGTCGTGGTGGATGACCGGCTGCCGGTCCAGAGAGGCCGCCTGCTGTTCAGCTACTCCCGCACGCAGAACGAGTTCTGGAGCGCGC
TGCTGGAGAAGGCCTACGCCAAGGTCAACGGCTGCTACGCCAGTCTGAAGGGGGGCAACATCTCGGAGGCCATGGAGGATTTCACCGGGGGCATCGCCCGGTCCTTGCCCGTGAAGTCC---------
GTGCTGTGGCGGGCGGTGGGAGAGTCCCTGTCCCGGGGCACACTGCTGTCC------------------------------------------------------------

```
GACCGCTGTGCCCTGCGCTGCTCCCTGTGCAGGCTG---------------------------------
CCAGTGCTGCCGAGGTGCGAGGACTGGGACAGAGTGGACAACGCAGAGAAGCAGAGGATTAAGCTGCGTGAGGACGGAGAGTTCTGGATCAAGGCGGATGACCTCTCGCACCTGTTCACCACAGTGGAGATGTGCAGTGTCAATC
CGGATGCCCTA------------------CTCTGGAGGATCACCGCTCATGAAGGGGCCTGGGTGCCAGGCTGCTCTGCTGGGGGAAGCCGCAAGTTCAGA------ACCTTCTGTAAGAACCCTCAGTTCCGGCTGATCCTG--
-------------------------GGCAGTGTGGTCCTGGAGCTGCTGCAGAAGCACCGCAGGCAG------ATCAGCTTCCTCTACATCGCCTTCCATGTCTACAGGATT------
CCCGGCCAGCTGCGCGGGGTCAGGAAGAAGGTCAAGCTGGAGCCCGGTCACTACGTGACGTCCAGCTACAAGCCCAACGTCGAGGGCAGCTTCTTCCTGCGCATCTAC-------------------------------------
-----ACT---------------------------------GAGCTGGCGGGG-----------------------------------------------------------------------------------------------
----------------------------ATCTTCTCTCAGTTTGATGAGGATTCGTCTGGGACCATGAGCCCCTTTGAGCTGAGCCTGGCCTTGCAGGCAGCT---
GGGTTCCAGCTGGACGCCCCAGTGCTGCAGCTGCTGTGGCTCCGTCACGGCACGGCTGACCTCTCGCTGACCTTCGACGGCTTGGTGGCGTGCGTTGGCAAGCTGCGCAAACTCTTC---------;

END;



BEGIN TREES;

        TREE tree =
(((((((OVIS_ARIES_XM_004015671,SUS_SCROFA_ENSSSCG00000002967),CERATOTHERIUM_SIMUM_XM_004441492),(HOMO_SAPIENS_AK127398,MUS_MUSCULUS_ENSMUSG00000
054083)),MONODELPHIS_DOMESTICA_ENSMODG00000013388),ANOLIS_CAROLINENSIS_ENSACAG00000011727),(XENOPUS_TROPICALIS_ENSXETG00000002784,XENOPUS_LAEVIS_
BC154987)),(DANIO_RERIO_ENSDARG00000010758,(TAKIFUGU_RUBRIPES_ENSTRUG00000010051,(GASTEROSTEUS_ACULEATUS_ENSGACG00000009801,(XIPHOPHORUS_MACULATU
S_ENSXMAG00000018623,OREOCHROMIS_NILOTICUS_ENSONIG00000003718))))),LEPISOSTEUS_OCULATUS_GENSCAN00000011248);

END;
```

## (*k*) CAPN13

```
#NEXUS

BEGIN TAXA;

        DIMENSIONS NTAX = 40;

        TAXLABELS

                'XENOPUS_CAPN1L_ENSXETG00000022058_ENSXETT00000047750' 'X_LAEVIS_CAPN13_NM_001093584'
'XENOPUS_CAPN13_ENSXETG00000022056_ENSXETT00000047741' 'X_LAEVIS_CAPN13_NM_001086019' 'CHICKEN_CAPN13_ENSGALG00000020084_ENSGALT00000014759'
'TURKEY_CAPN13_ENSMGAG00000005407_ENSMGAT00000006081' 'TURTLE_CAPN13_ENSPSIG00000005500_ENSPSIT00000005985'
'ANOLE_CAPN13_ENSACAG00000007376_ENSACAT00000007402' 'PIG_CAPN13_ENSSSCG00000020986_ENSSSCT0000031346' 'HUMAN_CAPN13_NM_14457'
'CAT_CAPN13_XM_003984358' 'HORSE_CAPN13_XM_001500391' 'MOUSE_CAPN13_ENSMUSG00000043705_ENSMUST00000095208'
'OPOSSUM_CAPN13_ENSMODG00000015519_ENSMODT00000030511' 'COELOCANTH_CAPN13_ENSLACG00000011037_ENSLACT00000012619'
'COD_CAPN14_ENSGMOG00000009688_ENSGMOT00000010648' 'MEDAKA_CAPN14_ENSORLG00000019002_ENSORLT00000023709'
'TILAPIA_CAPN14_ENSONIG00000008269_ENSONIT00000010414' 'PLATYFISH_CAPN14_ENSXMAG00000000132_ENSXMAT00000000184'
'PLATYFISH_CAPN14_ENSXMAG00000000065_ENSXMAT00000000126' 'STICKLEBACK_CAPN14_ENSGACG00000005683_ENSGACT00000007524'
'PLATYFISH_CAPN14_ENSXMAG00000000188_ENSXMAT00000000185' 'ZEBRAFISH_CAPN14_ENSDARG00000008553_ENSDART00000122453'
'ZEBRAFISH_CAPN14_ENSDARG00000052917_ENSDART00000074854' 'FUGU_CAPN14_ENSTRUG00000000168_ENSTRUT00000000342'
'STICKLEBACK_CAPN14_ENSGACG00000020515_ENSGACT00000027179' 'TILAPIA_CAPN14_ENSONIG00000005520_ENSONIT00000006943'
'TILAPIA_CAPN14_ENSONIG00000005532_ENSONIT00000006957' 'TILAPIA_CAPN14_ENSONIG00000005524_ENSONIT00000006948'
'XENOPUS_CAPN14_ENSXETG00000033183_ENSXETT00000062505' 'CHICKEN_CAPN14_ENSGALG00000009069_ENSGALT00000014761'
'TURKEY_CAPN14_ENSMGAG00000005468_ENSMGAT00000006245' 'TURTLE_CAPN14_ENSPSIG00000017963_ENSPSIT00000020410' 'HUMAN_CAPN14_NM_001145122'
```

'CAT_CAPN14_XM_003984351' 'RHINO_CAPN14_XM_004418593' 'SHEEP_CAPN14_XM_004005801' 'PIG_CAPN14_XM_003125263'
'TASMANIAN_DEVIL_CAPN14_ENSSHAG00000012346_ENSSHAT00000014582' 'ANOLE_CAPN14_ENSACAG00000007149_ENSACAT00000007269' ;

END;

BEGIN CHARACTERS;

DIMENSIONS NCHAR = 1530;

FORMAT

DATATYPE = DNA

GAP=-

MISSING=?

NOLABELS

;

MATRIX

AATCAGGACTTCCACGCACTGAGGGATCTGCACCTGAGGAAGGGCGTGTTGTTCAACGATGAGGAATTCCCAGCAGACATGCGCGTCATACATTTAATAGATGAGTTCAAGATACACAACATGGAATGGAGACGGCCGCCCCATT
TAATTGTGGACGGAGCCAGTTACTTTGACATTGTACAAAGTAAAATAGGTGATTGCTGGGTTTTGTCTACAATTGGATCCGTTACCCAGAAGCAAACGTTACTGAGAAACATCATTCCAGCTGATCAAGGGTTTACCAACTACGC
TGGCATCTTCCATTTCAGGTTCTGGTATTCCGGGAAATGGGTCGACGTTGTGATAGACGACCGACTGCCTTTT---
GGGGACTTTTTTTCTGTACGGGCCAGTTGTGCGAAGGAATACTGGCCCTGCCTTCTGGAGAAAGCATACGCCAACTTATATCAAGGTTATTGGTATATAATATGGACTACTGGGGCTTTTCAATGTAATAATTTCACAAATGTTA
CCACAACTGACTTTAAGGCTGTC------ATT---GTGCTGGTTCAAAAACCC---AAC---------TTGTTTCTGTGTCACGTTGATAACGGCCTAGTGGATCGACATGCTTATACTGTAACTGACACGGCGCAGGTT---
TACAGGGAAGGGTGTGTGAATTTAATTCGCCTGTGGAATCCATGGGGACGAGGAGAATGGATCGGCCGCTGGAGCGACAATTGCTCCTGGTGGAACGACATTGATCCAGAAGACAGAAAGAGGTTGAATAGAGAAGATGGAGAGT
TTTGGATGTCTTGGGAAACTTTCGTCCAGCAGTTTTCCCGGGTCATTATCTGCAGCCCCACGCTGGACTTTTTGAATAGGTCTTGGCACAAAACTTCCTATGAGAACATCTGGGATTTATTCTGCAAGAATCCCCAGTACCTTAT
ACGGATC------
TATAATGTCATCATTTCGCTGATGAGGCTGCCGATTGGCTTTCTGGTTTCCGATGTGACCAAAGGTTTAAAACTGGCATCCGGCCGATACGTCATCACCCCCAATGCGGATCGGAGCCAGGAGTCATCGTTTCTGCTGCAGGTTT
TCCTGAGGGGAGGCAAAATGAATTCCCAGGATCTTCAGAAGTTTCTGAATGATGTAATTTCAAAAAGGTTCAGTACAGAGGCCAGCCGATCCATGTTGGCATCAATGGACTTCACTTGCAATGGAAAATTGGAACTTGAATTCTT
CATGCGACTCTGGAGGTACCTCAACCATTTTAAGGCGATATTCACTGATGTTGATGTCGATCAAAATGGATTTATTGGTCTCTCGGAATTAAGGAAAGCTGCGAAA------------
GGAATGGCGGTCAGCAGTGACCAACTTACCATCTTGCTCTTAAGATACGGAGATCTCAATTTTGAAGACTACTTGTGCTGCATGGTGCGACTGAAATCCGCCTTCAGTAAGTGGCACTTTTCCAGTTTCCACACCGGGGGAATCA
ATATTTCCGTTTTTTCGTGC

AATCAGGACTTCTATGCATTGAGGGAACTGCACCTGAAGAAGGGTGTGTTGTTTCAAGATGAGGAATTTCCAGCAGATTTGCGCGTCATACATTTAATAGATGAACTCAAGATACAGAACATGCAATGGAGACGGCCACCCCATT
TAATTGTTGATGGAGCTAGTTACTTTGACATTTTGCAAAATAAAATAGGTGATTGCTGGGTCTTGTCTACAATTGGTTCTGTTACCCAGAAACAAAATTTACTGATAAAGATCATTCCAGCTGATCAAGGGTTTACCAACTACAT
TGGAATCTTCCATTTCAGGTTCTGGTGTTCCGGGGAATGGGTCGATGTTGTGATAGACGATCGGCTGCCTTTT---
GGCGACTTCTTTTCTGTTCGGCCCAGCTGCACAAAGGAATACTGGCCCTGCCTTTTGGAGAAAGCATATGCCAAACTCCTTGGGGGATACCAAAACTTGCACTGGGGAGATCCAGCAGAAGCCATGGTCAACTTTACAGGAGCCT
TAACAATGACATTGAAGCTGAAAGGAGGAATATGGGAGATGGTCCATAAATCA---AAACATACGGCACTGATCACTTGTCAGATTGATAACGGCCTAGTGGATCGCCATGCTTACACTGTAACTGACACCGCTCAGGTT---
TACAGGGAAGGGTTAGTGAATTTAATTCGCCTGTGGAATCCATGGGGACGAGGAGAATGGATCGGGCGCTGGAGCGACAATTGTTCCTGGTGGAATGATATTAATCCTGAGGACAGAAAGAAGCTGAATAGAGAAGACGGAGAGT
TTTGGATACCTTGGGTAAATTTCGTCCAGCAGTTTTCCCGGATAATCATCTGCAGTCCCACCCTGGACTTTTTGAATACATCTTGGCACAAAACTGCCTATAAGAACATCTGGGATTTGTTCTGCAAGAATCCCCAGTACCTTAT

ATGGATA------
TATAATGTCATCATATCGCTGATGAGACTGCCGATTGGCTTTCTGGTTTCCGATGTGACCAAAGGATTCAGACTGGCGGCAGGACGATATGTAATCACCCCCAATGCGGACCGGAATCAGGAGTCATCCTTTCTGCTGCAGGTTT
TCCTGAGGGGAGGCAAAATGAATGCCCGGGATCTACAAAAGTTTCTGAATGATGTAATTTCAAAAAGATTCAGTATAGAGGCCAGTCGATCCATGTTGGCATCAATGGACTTCACTTGCAATGGAAAGTTGGAATGTGATTTCTT
TAAGCGGCTCTGGAGGTACCTCGACTACTTTAAGGCAATATTCACTGATGCTGATGTCGATGGAAGTGGATTTATTGGCCTTGAAGAATTAAGTAAAGCTGTGAAA------------
GGAATAGCAGTTAGCAGTGATCAACTTACCATCTTGCTCTTAAGATACGGAGATCTCAACTTTGAAGATTATTTGTGCTGCATGGTGCGACTGAAATCAGCCTTCAAAAGGTTTCAGATGCTGACAAGTGATGGAAAAGGAGTTT
ATCTTTCCCACGACTCCTGC

GGGCAAGATTTCGACTCCATCCGAGAAAGCTGCTACAGCAGGAGGCAACTGTTCGAGGATGAAACGTTTCCCGCGAGTGTCAGCTCCATCAAACTGCTGCCCAAGGACCAACTGCTCAGCATCAAGTGGGAGAGGCCAGCCCGGC
TCTTGGTGGACGGGGCGAGTATCTTCGATATGGTCCAAGGCTATCTAGGGGACTGCTGGGTACTGGCCGCCGTTGGGGCTTTGACTTTGCACCAGAAGTTTCTAGATATCGTTATCCCGAAGGACCAGGAGTTTAATAAATATGC
CGGCGTCTTCCACTTTAGGTTCTGGCAGTTCGGCGAGTGGGTGGATGTGGTTATAGACGACCGACTGCCAACG---
GGAAAGTACCTGTCGGTCCATCCGCGCAGTGACAATGAGTTCTGGCCCACCCTGCTGGAGAAAGCCTACGCCAAGCTGCGGGGCTCGTACCAGAACCTACACTGGGGTTACATTTCGGAGGCGCTGGTGGACTTCTCAGGGGGGG
TACTGGTGGAGTTTGATCTGACGAAGCCCCTGAGGGACATTGTTATAGCAGCCGCCAAATCAGGGTCCCTCATGAATTGTGAATTACAGAACGGCCCTTGTGCAGGGTCATGCCTACACCGTGACTGATGCTACACAGGTGGAGTA
CAAGAATGGTACAGAAGATCTGGTCCGTGTTTGGAATCCCTGGGGGAAGGGAGAATGGAACGGACGATGGAGTGATAATGCTCCTCAGTGGGACCGGGTCAGAGCGGATGTGAGACAAAAGCTAAATAAGAACGACGGAGAATTT
TGGATGTCTTGCCAAGATTTCCTGCAGAATTTCAGCTGTGCGAGTATTTGTAACCACACGCCGGCGTATTTCGACCGCACTTGGCAGACGCTCACGTATTTCAGCCGTTGGGAGGATCTATGGAGGAACCCCCAGTTTGTGATAT
CGGTG------
TACAATGTGACTGTGGCGCTGATGGAGATGGGCATCGGCTTTGCACTGTGCGAAGTCACTCGCTCATTCATGGCTCCCCCCGGGACGTACGTCGTTATCCCCACAGAAAGCAAAGGCCAAGAGTCGGAATTCCTCCTTCGGATCT
TCCTGAAGAGCTCGGAATTATACGCAGAACAGCTACAGCGACTCCTGAATGAGGTGATCATTAAAGACTTCACCTTGGACGCCTGCCGCGGGATCCTGATGCTGATGGATCTAAATGCCAACGGGAGGCTCAGTCTGCAAGAATT
TGGGCGCCTTTGGAAACGACTCAATATGTGTAAGGACATGTTCAGATCAATAGACGGGAACCAGACAGGATTCATTGACGCCTCCGGTTTGAAGAAAGCAGTTCAA------------
GGTACAGAGTTGGACAACGCTCTTATTAACGTCATGGTGCTGAGATACGCCAACCTCAGCTTTGCCGATTTCGTTTGCTGCATGATCCGCCTGGAAACCGTAACCAAGGTATTTAAGAACGTATCAAAGGACGGCAGAGGGGTTT
ATTTTCTTGTGTTCTCCAGC

GGGCAAGACTTTGACTCAATCAGGGAAACCTGCTACAGCAGGAGGCAACTGTTTGAGGATGAAACTTTTCCCGCGCACGTCAACTCCATCAAAGTGCTGCCCAAAGACCAGCTGCTCAGCGTACAGTGGGAGAGGCCAGCCCGGC
TCTTGGTGGATGGGGCGAGTATCTTTGATATTGTCCAGGGTCAAATAGGTGACTGCTGGGTTCTGTCTGCTGTCGGGGCTTTGACTTTGCACCAGAAATTTCTAGATAATGTCATCCCCAAAGATCAGGAGTTTAATAAATACGC
AGGTGTCTTCCACTTTAGGTTCTGGCAGTTTGGTGAGTGGGTGGATGTGGTTATAGATGACCGGCTGCCAATG---
GGAAAGTACCTGTCTGTCCAGCCACGCAGTGGCAATGAATTCTGGCCCGGCCTGCTGGAGAAAGCCTACGCCAAACTTCATGGCTCCTACCAGAACCTGCATTGGGGTTACATATCAGAAGCCCTGGTAGATTTCTCAGGAGGGG
TGCAAGTGGAGTTTGATCTAATGAGGCCCCTGAGGGACATTGTTATAGCAGCTGCCAAAACGGGTTCACTTTTGGGCTGTGAGTTGAAAAATGGGCTTGTGCAGGGTCATGCCTACACCGTGACTGATGCTACAAAGGTGGAGTA
CAAGAATGGTACAGAAGATTTGATCCGTATTTGGAATCCCTGGGGGAAGGGAGAATGGAATGGACGATGGAGCGATAATTCACCTCAGTGGGACCAGGTCAGAGCCGATATAAGACACAAGCTAAATAAGAACGACGGAGAATTT
TGGATCTCCTGCCAAGATTTCCTGCAGAATTTCAGCTTTGTGAGTATTTGTAATCACACTCCGGCGTATTTTGACCGCGCTTGGCAGGAGCTCACGTACTTTGACCGCTGGGAGGACCTGTGGAGGAACCCCCAATTTGTGATAT
CAGTG------
TATAATGTGACTGTGGCGCTAATGGAGATGGGCATTGGCTTTGCATTTTGCGAAATCACTCGCTCATTTAGGGCTCCTCCCGGGACGTATGTTGTTATCCCCACCCAAAGTAAAGGCCTGGAGTCCGAATTTCTCTTACGGATCT
TCTTGAAAAGCTCGGAATTATACGCAGAACAGCTACAGAAACTCCTGAATGAGGTGTTAATTAAAGACTTCACCTTGGATGCCTGCCGGGGGATCCTAATGTTGATGGATCTTAATGCCAACGGCAGGCTTAACCTGCAAGAATT
TGAGCGCCTCTGGAAACGACTCAGTATGTGCAAAGACATATTCAGATCCATAGATCTGAACCAGACAGGATTC--------------------------------------------------------------------------------------------------------------------------------------------------------------------------------------------------------------------------------------------------------------------------------------------------------------------------------------------------------

AATCAGGACTTCGAGCACCTGCGATCCCTGTGTCTGAGCCAAGGCCTGTTGTTTGAGGATGACATCTTCCCTGCTGATACCAGCTCCATCAACCTGCTCCCAAAGGATAAGCTGCAGCAAATAGAGTGGAAGAGGCCACCCTGCT
TGATAATGGATGGAGTTAGCAGATTTGATATTATTCAAGGAGAAATAGGTGACTGCTGGATGCTGGCTGCCCTGGGCTCCCTGACAATGCAGAAGCAATTTTTGGAAAAGGTTTTACCAAAGGACCAAGGATTCCAGGATTATGC
TGGGATTTTCCATTTTCGGTTCTGGCAGTTTGGAGACTGGGTGGATGTAGTGATAGATGACCGGCTGCCTTTC---
GGGAATTACCTGTCTGTACACGCTCGAACATCAAATGAATTCTGGCCATCCTTGCTGGAAAAAGCATATGCCAAGCTGCAGGGCTCCTACCAGAACTTGCACGGGGGCTACATTTCTGATGCTTTAGTAGACTTCACAGGTGGAG
TCCAAATGCAGTTCTCACTAAAGGACCCCCTGGAGGACGTGCTGAAAGCAGCTAGCAAATCTCGGTGTCTGATGGGATGCGTATTAAGGAATGGGATTGTGCAAGGTCATGCGTACACTGTCACAGGAGCCGTGAAGATACGATT
CAAGAACCAGTGGGAACATATCATCAGAGTCTGGAATCCATGGGGCCATGGGGAGTGGAGGGGGGCCCTGGAGTGATGGCTCTCCAGAATGGGACTATGTTGAGCCTGAAATTAAGGAAGAGCTCTACAAAAATGATGGAGAATTC
TGGATGTCCTATGAAAACTTTCGGGAGCAGTTTTCCTGGCTGTGTGTATGTAACTGCACCCCAACGTTTCTGGACACAGGATGGTCTGTGGAAAGACACATCAACCTGTGG------
GTTTCAAAGAACCCTCAGTATTTCTTCAAAGTG------
TACAATGTGGTCATTTCACTCATAGCATTGAAGATTGGCTTTTTCATCACCGACGTGAGCAGCTGCTTTAATTTGAGCCCAGGAACTTATGCTGTCACTCCTACGACAGAGGAC---
CGAGAATTTGAATTTGTCTTACGAATTTTTGTAAAGGGCTCAGCTATTGATGCTTTGCAGTTGCAACGACTTCTTAATGACATGGTCCTGCAAGAGTTCAGCTTTGATTCGTGTCGAGCCATTTTAGCTCTGATGGATCTCAATT
CAAATGGACAACTCACACTGCAGGAGTTTGGGAGCCTCTGGCGAAGTGTCACCAAGTACATGGATCTCTTCAGGAGGGAAGACAGAAATTGTTCTGGATTTCTTGATGTATACGAGCTGAAGAGTGCGATACAG-----------
-

GGCCTGCCCGTCAATGAGCAGATCCTGCACCTGATGGCCCTGCGCTATGGCAACATGGGCTTTGCTGACTTCGTGAGCTGCATGCTGCGCCTTGAAACCATGACCTATGCATTTCAGAACTTAGCCAAAGGTGGACCGCAAGTTT
TGATGACGGTGACGTACATC

AATCAGGACTTCGAGCACCTGCGATCCCTGTGTCTGAGCCAAGGTCTGTTGTTTGAGGATGACACCTTCCCTGCCGATATCAGCTCCATTAACCTGCTCCCAAAGGATGAGCTGCAGCAAATAGTGTGGAAGAGGCCACCCTTTT
TGATAATGGATGGAGTTAGCAGATTTGATATTATTCAAGGAGAAATAGGTGACTGCTGGATGCTGGCTGCCCTGGGCTCCCTGACAATGCAGAAGCGATTTCTGGAAAATGTTTTACCAAAGGACCAAGGATTCCAGGATTATGC
TGGGATTTTCCATTTTCGGTTCTGGCAATTTGGAGACTGGGTGGATGTAGTGATAGATGACCGGCTGCCTTTC---
GGCAATTACCTGTCTGTACACCCTCGAACATCAAATGAATTCTGGCCATCCTTGCTGGAAAAAGCATATGCTAAGCTGCAGGGCTCCTACCAGAACTTGCACGGGGGCTACATTTCTGATGCTTTAGTAGACTTCACAGGCGGAG
TCCAAATGCAGTTCTCATTAAAGGACCCCCTGGAGGACATTCTGAAAGCTGCTAGCAAATCTCAGTGTCTGATGGGATGTGTATTAAGGAATGGGATTGTGCAAGGTCATGCATACACTGTCACAGGAGCCGTGAAGATACGATT
CAAGAACCACTGGGAACATATCATCAGAGTCTGGAATCCATGGGGCCATGGGGAGTGGAAGGGGCCCTGGAGTGATGGCTCTCCAGAATGGGACTACGTTGAGCCTGAAATTAAGGAAGAGCTTTACAAAAATGATGGAGAATTC
TGGATGTCCTGTGAAAGCTTTCGGGAGCAGTTTTCCTGGCTGTGTGTTTGTAACTGCACCCCAACGTTTCTGGACACAGGATGGTCTGTGGACAGGCACATCAACCTGTGG------
GTTTCAAAGAACCCTCAGTATTTCTTCAAAGTA------
TACAATGTGGTCATTTCACTCATAGCATTGAAGATTGGCTTCTTTATCACCGACGTGAGCAGCTGCTTTAATTTGAGCCCAGGAACTTATGCTGTCATTCCTACAACAGAGGAC---
CGAGAATTTGAATTTGTCTTACGAATTTTTGTGAAGGGCTCAGCTATTGATGCTTTGCAGTTGCAACAACTTCTTAATGACATGGTCCTGCAAGAGTTCAGCTTTGATTCATGTCGAGCCATTTTAGCTCTGATGGATCTCAATT
CAAATGGACAACTCACGCTGCAGGAGTTTGGGAGCCTCTGGCGAAGTGTCACCAATCTCTGCGATCTCTTCAAGAGAGAAGACAGAAATCGTTCTGGATTTCTCGATGTATCTGAACTGAAGAGTGCGATACAG-----------
-
GGCCTGCCCGTCAATGAGCAGATCCTGCGCCTGATGGCCCTGCGCTACGGCGATATGGGCTTTGCTGACTTCGTGAGCTGCATGCTGCGCCTTGAAACCATGACCTATGCGTTCCAAAACTTAGCCAAAGGTGGACCACAAGTTT
TGATGACGGTG---------

AATCAGGACTTTACCCAACTGAGAGACTACTGCCTGAGACGACGCCTGCTGTTTGAAGATGAGACCTTCCCAGCACATGTCAGTTCCATTAGTCTGCTTTCCGAAGACAAGCTGAGCCACATACAGTGGAAACGACCACCCCATT
TAATCATGGATGGTGCTAGCAGATTTGATATTCTTCAAGGAGAAATAGGCGATTGCTGGGTTCTGGCTGCCCTGGGATCTTTGACATTACAGAGGCAATTTCTGGAAAATGTTCTGCCAAAGGACCAAGGATTCCATAGTTATGC
TGGGATTTTTCATTTCCGGTTCTGGCATTTTGGAGACTGGGTGGATGTGGTGATAGATGATCGGCTGCCTTTT---
GGGAAATACCTGTCTGTCAGACCTCGCTGCAAAAATGAATTCTGGCCATCCCTGCTAGAGAAAGCATATGCCAAGTTGCGAGGCTCCTATCAGAACTTGCACTTGGGTTACATTTCCGAAGCACTAGTGGACTTGACGGGTGGAG
TTCAAGTGCAATTTTCTTTACAGAGCCCTCTGCAGGAGATACTGAAAGCAGCTGTGAAATCACAATGCCTGATGGGATGTGAGCCAAAGAATGGACTTGTGAGAGGCCATGCCTATACTGTGACAGGGGCTGAAGAAATACCATA
CCAGTACAGCCAGGAGGAGATCATCAGAGTGTGGAATCCTTGGGGCCACCGGGAGTGGAAAGGGCCTTGGAGTGATCGTTCTCCGGAATGGGATCAAATTCCAGCTGAATATAAGAAAGCACTCTGTAAAGACGATGGAGAATTC
TGGATCTCTCGTCGAGATTTCGTCAAGCAGTTTTTCTCACTGTGTATTTGCAATGTCGTCCCATCATTTCTGGACACAACATGGGCCATGACCGCATATGTAAACCAATGG------
TTTTCAAGGAACCCACAATATTTCATCCAGATA------
TATAATGTGGTGGTGTCACTGATGGTCTTGCTCATTGGATTTTTCATCTTCGATGTCACCGATTGCTTTTTCTTGAGTCCAGGGATCTATGTTGTTGTTCCAACATCAGAGGAAGGCCAAGAGGCTGAATTCCTTCTACGAATCT
TCCTAAAGGACTCAGACATGAATGCTTCACAGCTGCAAAGGCTCCTCAATGAAGTGTTCCTGAAAGAATTCGGCTTTGATTCATGCAGAGGCATTTTAGCTCTGATGGATCTCAAGTCTAATGGACGACTATCCCTGAAAGAATT
TAAGCACCTCTGGAAACTGCTTGTTAAGTACAAGGACATTTTCAGGATGGAGGAGAGAACGCATGCGGGATTCCTTGACGTGTCAGATTTCAGGCCCCCATCTCATTAACCTGATGAAAGGCCTGGCTGTCAATGACCAGCTCTTC
CATCTGATGGCTCTGAGGTACAGTGACGTTTCCTTCCCTGATTTTGCATGCTGCATGATCCGGTTTGAAACTATGGCAAGTGTATTTCGTAATTTATCAAAGGATGGA---AAGATTTCCTTTTCTGCAACATATTGC

AACCAGGACTTTGTTTACTTGACAGACTATTGCTTGAAGCATGGCATACTTTTTGAAGATGATACGTTCCCTGCACATTACAGTTCTATTAAAGTCCTCCCAGAAGACAAGTTGCGCCGAATAAAGTGGCTGAGACCACCCCGTT
TATTTGTGGATGGTGTGAACAGAGCTGATATTCTTCAAGGACAAATTGGTGATTGTTGGGTCCTAGCAGCCCTGGGTTCTTTGACCCGGCAACAGCGGTTCCTGAAAAATGTTATTCCAAAAGACCAAGGATTCGAAAGCTATGC
AGGGATTTTCCATTTTCGGTTCTGGCAGTTTGGAGACTGGGTGGACGTGGTGATAGATGACCGACTTCCTTTT---
GACAATTTCTTATTTGTCCATCCTCGCAGCAAAAAATGAATTTTGGCCACCCCTTGCTTGAAAAGGCCTATGCCAAATTGCGTGGTTCCTATACAAATTTGGACCAGGGTTACCTTACAGAAGCTTTGGTGGACTTCACTGGTGGAG
TTGAGATGCCTTTTAACCTTAAGAACCCTCTATTTGAGATGCTAAAAACAGCTGCAGAGTCCGGCTGCCTTATGGGATGCCAATTGGAGAATGGGATTGTGCAGCTTCATGCTTACACTGTCATATGGGCAACAGAGGTACCCTA
TATGGATGGGAAGGAACGTTTAGTCAGACTTTGGAATCCGTATGGTAATACAGAATGGAAAGGCGCTTGGAGTGATCGTTCTGTGGAGTGGCAGCACGTTCCAATACCGTACAGGAAAAAACTCTATAAGGATGATGGCGAGTTT
TGGATATCTTATAAAGATTTCAGAGACAACTTTTCATTCCTGATTATTTGCAACGATGTGCCAACATGCCTGGACACTACTTGGTCTGTGGATAAGCATGTCAACAAATTTGATATCCTTTCCAGAAATCCTCAGTATTTCATCC
AGGTC------
TACAATCTTGTAGTAGCACTTTCAAGAGTACACATTGGCTTTGTAATTGTTGATGTTGTTAATTGCTTTTACTTGAGCCCTGGGACTTACATCATTATTCCAACAACACAAGAAGGTGAAGGGACCGAGTTCCTCCTACGAACCT
TTCTAAAGAGCTCATTCCTCGATGCTTCACAGTTACAAAGAATTCTCAATGAAGTACTCCTGAAAAGATTCAGCTTCGATTCAAGCAAAAGCCTTTTAGCTCTGATGGATATCAATGCTAACGGAAGGCTTTCGCTAGACGAATT
TGGAGAACTTTGGAGGGAGCTCAATATATATAAGGACATTTTTATAAAGGAAGATGAACAGAACTCTGGGTTCCTGAATGCCTCCAATCTGAAAAGAATAATCCAG-----------
GGCCTGTCGGTCAGTGAAAAACTTCTTGGACTGATGACTGTACGATATGGGGATATGAACTATCCTGGTTTTGTGTGTTGTATGATTCGTCTGGAGACCATGGAAAAAGTATTTCACAATTTATCTAAAGATGGGAGAGAAATCT
GTTTTACTGAAGATTATTGC

GAACAGGACTTTTACTCCTTGCGGGATCATTGCCTAAAAAGAGGCGTGCTATTTGAGGATGAGACGTTTCCTGCTGACACTTATTCCATACAGCTGCTCAAGGGCAAGAACCTCTCCAGCCTAAGCTGGATACGCCCCCCTCACT
TCATCCTGGAAGGTGCAAGCAGATTCGACATCCAACAAGGAGATGCAGGTGATTGCTGGTTCCTGGCAGCACTGGGCTCCTTGACACAGAACCCACAGCATCTGCAGAAGATCTTG---
ACAGATCAAAGCTTTTCCCACTATGCGGGGATTTTCCTTTTCCGGTTCTGGCAGTGTGGCCAGTGGGTGGAAGTGGTGATC---------------------
AAGGAGTTCCTCTTTGTGCATCCTCGCAACAAACGGGAGTTTTGGCCGTGCCTGCTGGAGAAAGCTTACGCCAAGTTTCATGGGTCCTATGCCAACCTGCACTATGGCTACCTCTCTGACGCCCTGATGGACCTCTCGGGAGGGG
TGGTCACCAACATCCGCCTGCACTCCTCCCTGATGATGATGGTGAAGACAGCGGCCGAGGCAGGCTCTCTCATAACCTGCATGATGGTGAATGGGCTGGTGAGTCGGCATGCCTACACGGTGACTGGCGCCGAGAAGATTCAGTA
CAGGAAGAGCTGGGAATATCTTATCCGCCTGTGGAACCCCTGGGGAAGGATGGAATGGAGAGGACCCTGGAGTGATGGGTATGGCTTCTGGAAACCTCTCTCTCCTTCTTGGAGACGGGGGGTGACATTCCGTGAGGGCAGCATC
TGGTGTGGGTGCAGGTCTGTCCAGCCCACACCTTTGTGT------GACTGTCAGTCCATCTCACACTCCCGTGATAGTAGGTATTTCCCCATTCAGACACACACATACATC------------
ACAAGTTTCCATCATAATTACAGCAGT------ATAAGCGCCCTTCTCTGTCTCACC---TTTCCTTCTGATTTTACCATCATTGGCTTCAAGAAAAGGCATGAGCCCCAGCCTGAGAAGTACAGGGTGAGG---
TGTGATTGCAGC---
TCGAAGACCCCCCCACACACACCTGTGTACACACCGGGACGGTTCTTGGACGCTACCCAGCTTCAGAGTCTCCTCAACCAGGAATTCCTGAAAACATTCTCTTTGGATGAGTGCCGGGGCATCGTGGCTCTGATGGATCTGGAGG
TGAACGGGCGGCTTAACGAACAGGAGTTTTCAAGGCTGTGGCGCCGTCTTGTCCATTGCCAGAGTGTTTTCTGGAACGCCCGGAAGAAC---
TCCGGAGTTTTCCTGAGCTCAGATTTGCAGAAGGCCATACAGGACTTCCTTGCAGGGGTCTCTGTCACCAGCGAGCTTCTGGATCTCAGCACCAACCCCCACAGCAGCATTAGT---------------------------
AAGGTGGAGAGAACAGAGCAAGCTTTCCAGAATCTCTCCCAGGATGGAAAAGGACTCTACCTGACAAAAATGTACAAC

GACCAGGACTTTACCACCTTGCGGGATCACTGCCTGAGCATGGGCCGGACGTTTAAGGATGAGACATTCCCTGCAGCAGATTCTTCCATAAAGCTGCTCCAGGAAAAACGCCTCTCCAATGTGATATGGAAGCGGCCACCTCACT
TCATCCTGGATGATATAAGCAGATTTGACATCCAACAAGGAGGCGCAGCTGACTGCTGGTTCCTGGCAGCACTGGGATCCTTGACTCAGAACCCACAGTACAGGCAGAAGATCCTG---
ATGGTCCAAAGCTTTTCACAGTATGCTGGCATTTTCCGTTTCCGGTTCTGGCAATGTGGCCAGTGGGTGGAAGTGGTGATTGATGACCGCCTACCTGTC---
GATAAATGCCTCTTTGTGCGTCCTCGCCAAAACCAAGAGTTCTGGCCCTGCCTGCTGGAGAAGGCCTATGCCAAGCTGCTCGGATCCTATTCCGATCTGCACTATGGCTTCCTCGAGGATGCCCTGGTGGACCTCACAGGAGGCG
TGATCACCAACATCCATCTGCACTCTTCCCTGGTGAAGGCAGTGAAGACAGCGACCAAGGCAGGCTCCCTGATAACCTGTGCGATGGAGAATGGGCTGGTGAGTCTCCATGCCTACACTGTGACTGGGGCTGAGCAGATTCAATA
CCGAAGGGGCTGGGAAGAAATTATCTCCCTGTGGAACCCCTGGGGCGAGGCCGAATGGAGAGGGCGCTGGAGTGATGGGTCTCAGGAGTGGGAGGAAACCTGTGATCCGCGGAAAAGCCAGCTACATCGGGAAGATGGCGAGTTT
TGGATGTCGTGTCAAGATTTCCAACAGAAATTCATCGCCATGTTTATATGTAGCGAAATTCCAATTACCCTGGACGAAGGATGGTCCCAAATAATGTTTAGGAAGCAAGTG------------
AATGATGCTCAATTCAACTTCTCTGTG------ACCAATGTTGTCGTGTGCGTCACA---TTTCCACTCGATTTCCAAGTG---AACTTCACCATGACTTACCATCTGAGCCCTGGGAACTATGTTGTGGTT---
CAGACACGGAGA---
AAATCAGCGGAGTTCTTGCTCCGAATCTTCCTGAAAAGGCTGGACATTGATGCCACCCAGCTTCAGGGCCTTCTCAACCAGGAGCTTCTAACAATGTTCTCCTTAGATGAGTGCCGCAGCTTGGTGGCTCTGATGGAACTGAAAG
TGAATGGGCGGCTAGACCAAGAGGAGTTTGCGCGACTGTGGAAGCGCCTTGTTCACTACCAGCATGTTTTCCAGAAGGTTCAGACAAGC---
CCTGGAGTCCTCCTGAGCTCGGACTTGTGGAAGGCCATAGAGGACTTCCTCAGAGGGATCTTCATCAGCCGTGAGCTGCTGCATCTGGTGACCCTCAGGTACAGCGACGTCAGCTTCCCCAGCCTGGTCTGCTTCCTGATGCGGC
TTGAAGCCATGGCAAAGACCTTCCGCAACCTCTCTAAGGATGGAAAAGGACTCTACCTGACAGAAATGTACAAC

GGCCAAGACTTCAACTTCTTGCGGGATCATTGCCTAAGCAGAGGTCTGCTGTTTGAGGATGAGACCTTCCCTGCAGAGACTTCCTCCATACAGCTGCTCCAGGGAAAAAACCTCTCCAGACTGATGTGGAGACGGCCACCTCACT
TCATCCTGGAAGGTGCAAGCAGATTTGACATCCAACAAGGAGAGGCCGGAGATTGCTGGTTCCTGGCAGCACTGGGCTCCTTGACACAAAACCCACAGCACCTGCAGAAGATCCTG---
ATGGACCAAAGCTTTTCACACTACGCAGGGATCTTCCGTTTCCGGTTTTGGCAGTGTGGCCAGTGGGTTGAAGTGGTGGTTGATGATCGCCTGCCTATC---
AGGGAATATCTCTTTGTGCATCCTCGCAACAACCAAGAGTTTTGGCCCTGCCTGCTGGAGAAAGCTTATGCCAAGTTTCATGGGTCCTATCTCAACCTGCACTACGGCTTCCTTCCTGATGCCCTGGTGGACCTCACAGGAGGCG
TGGTCACCGGCATCGACCTGCACTCCTTTCTGGTGATGATACTGAAGACAGCAGCCAAGGCAGGCTCCCTGGTGACCTGCAGAATGGAAGACGGCCTGGTGAGTCAGCATGCCTACACAGTGACCGGAGCCGAGCGGATTCAGTA
CCGGGGGAGCTGGGAGGATCTTATCTGCCTGTGGAACCCCTGGGGCAACACGGAATGGAGAGGGCGCTGGGCAGACGGGTCACCAGAATGGCAGGAAACCCACCACCAATGGAGAAGACTGCTGTATAAGGATGATGGCGAGTTT
TGGATGTCATGTCAAGATTTCCAAGACAACTTTTCCTGCCTATACATATGTAACCAATTTCCAGTCAGTCTAAACGAAAGATGGTCCCAAATGATGTTTAAGAACCATGCA------------
AGGGACACGCAGTTCATCTTCTCTGTG------AACAATGTCGTCATGTCCTTCAAC---TTTCCACTCACCTTCAAGGTATTCAACCTCACAAAGTACTTCAGTCTGAGCCCTGGGACCTACGTGGTGGTC---
GCCACGTACAGA---GAA---
GTTGAGTTTTTGCTCCGGATCTTCCTGAAAGGGCTGGACATTGATGCAACCCAGCTTCAGAGCCTTCTCAACCAAGAGCTCCTGAGAACCTTCTCTTTGGATGAGTGCCGGAGCATCGTGGCCCTGATGGATCTCAAAGCGAATG
GGAAGCTTGAGCAAGAGGAGTTTTCAAGGCTGTGGAGCCGTCTCGTCCAGTGCCAGAGTGTTTTCCAGAACATCCAGAAACGT---
TCCGGAGTTTTCCTGAGTGTGGATTTGTGGAAGGCTATAAAGGACTTCCTTGCAGGGACCTCCGTCAGCAACGAGCTTCTGGATCTCATGACCCTCCGGTACAGTGATGTCAGCTTCCCCAGCCTGGTCTGCTTCCTGTTGCGAC
TTGAAGCCATGGCAAAGACTTTCCAGAACCTCTCCAATGATGGAAAAGGACTCTACCTGACAAAAATGTACAAT

GGCCAGGACTTTAACTCCTTGCGGGATCATTGCCTCAGCAGAGGCCTGCTGTTTGAGGATGAGACCTTCCCTGCCAAGACTTCTTCCATACAGCTGCTCCAGGGACGAAACCTCTTCAGCCTGAAGTGGAAGCGGCCACCTCACT
TCATCCTGGAAGGTGCAAGCAGATTTGACATCCAACAAGGACAGGCAGGAGATTGCTGGTTCCTGGCAGCACTGGGCTCCTTGACACAGAACCCACAGCATCTACAGAGGATCCTG---
ATGGACCAAAGCTTTTCACCGTATGCAGGAATTTTCCGTTTTCGGTTCTGGCAATGTGGCCAATGGGTGGAAGTAGTGATTGATGATCGCCTGCCAGTC---

AAGGATTACCTCTTTGTGCATCCTCGCAAAAACCAAGAGTTTTGGCCCTGCCTGCTGGAGAAAGCCTACGCCAAGTTTCGAGGGTCCTATCTCCACCTGCACTATGGCCACCTCCCTGATGCCATGGTGGACCTCACAGGAGGGG
TGATCACCACTGTCAACCTGCACTCCTCCCTGGTGATGATGGTAAAGATGGCAGCCAAGGCGGGCTCCCTGATGACCTGCAGAATGCCAAATGGCCTGGTGAGTCAGCATGCCTATACAGTGACAAGCGCTGAGCGGATTTGGTA
CAGGAATGGCTGGGAAGATCTTATCCACCTGTGGAACCCCTGGGGCAACACCGAATGGAGAGGGCGCTGGAGCGATGGGTCTCTGGAGTGGCAGGAAATCCATAACCAACAGAAAAGCCAGCTGTTTAAAAACGATGGCGAGTTT
TGGATGTCATGTCGAGATTTCCAAGAGAACTTCTCCTGCTTGTTTATATGTAACCAATTTCCAATCCTCCTGGACGAAAGATGGTCCCAAAACACGTTTAACGACCGTGCA------------
AGGGAGACACAATCCATCTTCTCTGTG------TACAATGTCGTCATGTCCTTCAAC---TTTCCACTCAGCTTCCATGTGTTCAACCTCACGAAGTGCTTCAGTCTGAACCCTGGGACCTATGTAGTGGCT---
ACTGCACACACA---
GAAGCAGTTGATTTCCTGCTCCGAATCTTCCTGAAAGGACTGGACATTGATGCCACCCAGCTTCAGGGCCTTCTCAACCAGGAATTCCTGAAAACTTTCTCCTTGGATGAGTGCCGGAGCATCGTAGCCCTGATGGATCTGAAAG
TGAATGGGAGGCTTGACCAAGATGAGTTTTCAAGGCTGTGGAGGCGTCTTGTCCACTACCAGAGTGTTTTCCAGAACACCCAGGAGAAC---
TCTGGAGTTTTCCTGAGCCAGGATTTGTGGAAGACCATAAAAGACTTTCTTGCAGAGGTCTCCATCAGCGATGAGCTGCTGGACCTCATGGCTCTCCGGTACAGTGACATCAGCTTCCCCAGCCTGGTCTGCTTCCTGATGCGGC
TTGAAGTCATGGCAAAGGCTTTCCAGAAGCTATCCACAGATGGAGAAGGACTCTACCTGACAAAAAGGTACAAC

AATCAGGACTTCAGGACCTTACGGGATCATTGCCTGAGCAGGGGTCAGCTGTTTATAGATGATACATTCCCTGCTGCGGCATCTTCCATAAAGCTACTCAGGGGGAAGCACCTTTCGAAACTGGAGTGGAAACGACCACCTCACT
TCATTCTGGAAGGTGCAAGCAGATTTGACATCCACCAAGGAATAGCAGGAGACTGCTGGTTCCTGGCAGCACTGGGCTCCCTGACCCAGAACCCGCAGTGTCTGCAGAAGATCCTG---
ATGGACCAAAGCTATTCGCAGTATGCTGGGATTTTCCAGTTCCGGTTCTGGCAGTGTGGCCAGTGGGTGGAAGTAGTGATAGATGACCAATTGCCCGTC---
GATAACTTCCTTTTTGTGCATCCTCGAGGCAACAAGGAATTTTGGCCCTGCCTGATGGAGAAAGCCTATGCCAAGCTACTTGGCTCCTATTCCCAATTACACTATGGCTACCTTCCTGATGCCCTGGTGGACCTCACAGGAGGAG
TGGTCACCATCATCAACCTGCACTCCTCTCTACTGACGACAGTGAAGACCGCAATCCAAGCAGGCTCAATGGTGGCCTGTGTGATGGAGAATGGACTGGTGAGCCAGCACGCATACACAGTGACTGGAGCTGAGAAGATTCAGTA
CCAGGGGCGTTGGGAAGACATCATCCGCCTTTGGAACCCCTGGGGCAAGACAGAATGGAAAGGACGCTGGAAAGATGGGTCCAAGGAGTGGAGAGAAACCCATGACCCACGGAAAAGCCAGCTGTATAAGGAAGATGGCGAGTTT
TGGATGTCCTGTCAAGATTTCCAGGAGAACTTCTCCTGCCTGTTCATATGTAACCAGATCCCGATCACCATGGATGAGAGCTGGCGACAAATGAGGTTTACAAACCAAGTG------------
AGGGATATGCAGTATGTCTTTAGTGTT------AATAATGTCGTGGTGGCCTTCACA---TTTCCACTACAGTTCCAGGTATTCAACTTCACAAAGTCCTTCCACCTGAATCCTGGTACCTATGTAGTGGTT---
ACAGCAAACGGA---
AAAGAAGTGGAATTCCTACTCCGAATATTCCTCAAAGGGCTGGACATTGATGCCACCCAACTTCAGAGCCTTCTCAATCAGGAGTTCCTAACAACTTTCTCCTTGGACCAGTGCCAAAGCATCATGGCCCTGATGGATCTGAAAG
TGAATGGGCGGCTAGATCAGGAAGAGTTTGCAAGACTCCGAAGTCGCCTTATCCACTGCCAGCATATTTTCCAGAGCATCCAGAGACGC---
CCTGGAGTTCTCCTCAGCTCAGACTTATGGAAAGTCATAGAGGACTTCCTTGTAGGAATTTTCATCAGCAGCGAGCTGCTGAGTCTCATGGCACTCAGGTACAGCGATGTCAGCTTCCCCACTCTTGTCTGCTTCCTGATACGGC
TGGAAACCATGGCAAAGGCATTTCGCAACCTCTCCAAGGATGGCAAAGGCATCTACCTGACAGAAACATACAGC

AACCAGGACTTCACTTCCTTGAGGGATCACTATTTAAGGAATGGCATGCTATTTGAAGATGACATGTTTCCTGCCGATGACAGTGCCATAAAACTCCTAAGGGAAAAAAATCTTTCAAGCCCTATATGGTATAGACCACCTCACT
TTATTCTGGATGGAATCAGCAAGTTTGATATTCAACAAGGAATAACAGGTGACTGCTGGTTTCTAGCAGCACTAGGATCTTTAACTCAGAGACCCAAACTTCTAGCAAAGATTATACCACCAAACCAAAGCTTCAAAGGATATGC
AGGGATTTTCCATTTCCAGTTCTGGCAATGTGGACAGTGGGTGGATGTGGTGGTTGATGACCGTCTCCCTGTA---
AACAAGTACCTGTTTGTTCATCCTCGTGGGAACAATGAATTTTGGCCCTGTTTGCTTGAGAAGGCTTATGCCAAGCTCCATGGCTCCTACTCCAACTTGCATTCAGGCAGTCTGGCTGACTCACTGGTGGAACTGACAGGATGGA
TAGTCACTGCAATTGATCTTAAGAAAGCCATGGTCCAGAATTTGAAAGTGGCTGAGCAATGTGGCTCAATAATAACATGT------
CAGTTTGGACTAGTGAATAATCATGCCTATGCAGTGACAGGAACTGCAGAGGTTCATTTGGGCACTGGCCCGGAAGAACTCATTCAATTGTGGAATCCCTGGGGATGTGAAGAATGGCTAGGACGTTGGAGTGATAAATCAGAAG
AATGGTGGAAAATCCAAGATTCAGAGAGAACCAGACTTTATAAAGCAGATGGAGAATTTTGGATGTCCTTTCAAGATTTCCAGATGAAATTTTCCCACATTTATATTTGCTATCAAGAGCCAATGGAATTGAACGGAACATGGAT
CCAAATTCCATGCAAAGTCCAAAAA------------AATACATGGCAGTGCCCCTTCTCTGTG------GTCAATGTGGTAGTCTCCATGAACAACATGCCACTT---
TTTGAAATATACAATTACACAGAAAGTATCCACCTGGTCCCTGGTAACTACATAGTGGTCCCCACCACTAATGCA---
AGAGAGTTAAGGTTCTTCCTTCGAATCTTCCTGAAAGGCCTAGACATTGGTGCATCACAGCTACAAAGTCTTCTCAACAAAGAACTCCTTAAAAAGTTCACTTTTGATGAGTGCCGGAGTATTTTGGCTCTGATGGATGTCAAGG
TCAATGGACGTCTTGATCTAGAAGAATTTGAGCAGCTATGGAAGAAGCTCATTCGATGTCAGAGCATTTTCCAGAAAGTAGAGAAGAATGCCTCTGGATTCCTCCCTGGGTTCAGATCTGTGGAAAGTGATACAAGGATTATTCCT
AGATACTTCCAATAACAGTGAGCTGCTAGACCTCATGGCCATTAGGTATGGAGATATATATTTCTCAGACCTGGTCTGTTTGCTCATCCGCCTTGAAATTGTGTCAAAGGCTTTTCATAACCTTGCAAGGGATGGGAGA---
ATCTACTTGACCAAAACTTATTGC

---
CAGGACTTTGAGAACCTGAGGGAGGAGTGTCTGAGCCAGGGGGTCTTATTTGAGGACAAAACCTTTCCCCCTGGTGTTGAGTCCATTGCTCTGTTGTCCGAGGAACAGCTCAGGAATTTGACCTGGAAGAGGCCGCCCCAGCTGT
ATGTCAAAGGAGCCAGCAGATTTGACATCAAACAAGGTTCTGTAGGTGACTGCTGGTTTTTAGCGGCTTTGGGTTCCTTGACGTGTCACGATCACCACTTGGAGTGGATCTTACCAAAAAACCAAGGATTCTCAGACTACGCTGG
GATTTTTCATTTTCGGTTCTGGCATTTTGGGCAGTGGGCGGACGTGGTTATAGATGACAGGCTGCCTATG---
GAGCGGTATTTATTTGTCTCACCTCGACAATCAAATGAGTTCTGGCCGGCCCTGCTGGAGAAGGCATACGCCAAGTTGTGTGGATCTTACCAGCACATTGACGGGGGGAACATTGCAGACGCGCTGGTGGATTTCACAGGAGGCA
TTAAGAAGGACATCTATTTGAAGAAGAAGGTGTGGGAGATGATGAAGAGATCTGACAAGCTGAAGTGTCTCATGGCGGCCGTTCTGGCCAAGGGCCTAGTAGAGGGACACGCTTACTCTGTGACCGGCATCGCTCAGGTAGATTA
CAAAGGCCGAACCGAACGCTTGATCCGACTGTGGAACCCATGGGGCCAAGAGGAGTGGAAGGGCCGATGGAGTGACGAAGCTGTGCAATGGGAACGGATTAGCAAAGAAGAACAAGACAATTTGAATAAAGAGGACGGTGAATTC
TGGATGGCCTTACAGGATTTTCGGGATTATTTTAATGGCTTGGTAATTAGTGACGCTAATCCAGAGTTTATGTTCCAGAAGTGGATGGTGAGGACGTATGAGAACGGCTGGCCCACGTTTTTGAAAAATCCGCAGTACAAGTTAC

AACTGAAAAAGTACAATGTCACCGTGTCCCTAATA---
CATGGCATCAGCATCGTGCTCCACGAGATCTCATGCAAGTTCATCCTGGAACCAGGAACTTATGTCCTTATCCCCACCAATAAGGCAGAACAGGAGGCTGGTTTCATCCTGCGTGTCTTCTACCGC-------------------
-------------------------------------------------------------------------------------------------------------------------------------------------
-------------------------------------------------------------------------------------------------------------------------------------------------
---------------------------------------------------------------------------------------------------------------

AACCAAGACTTCCAGCAGTTGAAAAAGTACTGCATTGAACGTCAATTAAGATACATTGATGACATGTTTCCTCCGAACCAAAACTCCATT---
TTTCTCTATCCCATTCGCCTGGCGAGAGTTGAGTGGCTGAGACCACCATCCTTTGTTGTCGATGGAGTTTCAAGATTTGATTTTTGCCAAGGAGTAGTTGGTAATTGCTGGTTCCTAGCTTCAGTTGGAGCTCTAACATTCCAGA
GCCAAATCCTTCAGCGAGTTCTGCCTTCTGAGCTCATT---------CAGTGCTGGCTATTCCACTTCAGGTTCTGGAGATTTGGATACTGGGTGGATGTTGTCATCGATGATAAGTTACCGACA---
GGCAAACTCATCTTTGTTCGCTCAAAAACCCCAAATGAGTTTTGGCCTGCTTTGCTGGAAAAAGCCTATGCTAAGGTTTGCGGCTCCTACGCAGACATGAATGCAGGAACCCCATCAGAAGCCTTGGTCGATTTTACAGGAGGCG
TGCACATATGCTTCCAACTGGATAGTGCTCTCTGGAACACAATGCACAGAGCTGCACAGAGCAATTCATTGATGGGGTGTGTCTCAGCCAATGGTTTAGTGGAAGGACATGCCTACGCCGTTACTGGTGTCACACAAGTGATCAG
CAATGGGCAGCCAATAAATCTTGTTCGCTTGTTAAACCCATGGGGCAATGGAGAGTGGATTGGCGATTGGAGTGACAGTTCTGTTTTGTGGCAATCTATTAGTGCTGAAGACCGTGAAATGTGTGTAGCTGATGATGGGGAATTT
TGGATGGCGATGGAGGACTTCTGTCGTTTCTTTTCAGATCTTGACATTTGTTGCCTATGCCCTGACTTCCTTGATTCTCACTGGACGCCCTTGGTCTATGAAAGCAGATGGGAAATGTTCAGGATGAACCCTCAGTATCGGTTCA
GGATT------
AACAACATTCTAGTGTCTCTCATGGAACTTTACATCGGTTTCTCAATTTTTGAGGTCATGGATATGTTCAAGTTGGCTCCTGCCGAATACCTGATTGTGCCAAGTTTCAAGCCAGATGAGACGGCTTCCTTCATTCTCAGAATTC
TCTCCAAGTTTGAAGAAGTCCATGCAGAGCAGCTCCAGAACATTTTAAATGAAAAGATCCTTCAAGGCTTCAGTCTGGATGCGTGTCGCAGCATGGTGGCTCTAATGGATACATCCATCACTGGCAAACTGAAACGTGATGAATT
CTTGCGTTTGTGGAGGAAAGTCACCACCTACAACGACATCTTCTCCCAAATTGATCCAACCCGCAAAGGTACTTTGTCCCTGCATGAGCTGAGAAAGGCTATAAAG------------------------
AAGGATGGTATGCTGAACCTGATGGCTGTTCGGTATGGCACCATTAGCCTGGAGAGCTACATTAATCTACTCCTTCGTTTGGAATGCATGGGAAAAATCTACACACAACTCTCG---
GGAGGAACGACCATGTCTCTCAGTGAATCT------

GACCAAGACTTCCAGCAGCTGGCAAAGTACTGCCTCTCTCAAAGAGTAAAGTTCCTTGATGAAACGTTCCCACCTGAAAGAAGCTCCATCGGGGTTCTGAGCCCCTCCGACCTGGCCAAAGTGGAATGGCTGAGACCACCTTCCT
TTGTCCTTCGCGGGGTCTCTAGGTTTGACTTTGGTCAAGGAACTGTTGGAAACTGCTGGTTTCTCGCATCGATTGGAGCTCTGACCTTCCAAAAAGAAATTTTCAACCAGGTGGTTCCTCTTGAGCAAACATTTGACAACTACTG
CGGATTGTTTCACTTCCGGTTTTGGAGGTTTGGGAAATGGGTGGATGTGGTCATTGATGATAAGCTGCCCACC---
GGCAGATTAATCTTTGTGCGGTCACAAAGTCAGAATGAATTCTGGCCTGCTCTGCTGGAGAAAGCCTATGCCAAGGTTTGTGGTTCCTACACTGACATGACAGCTGGAACGCCTTCTGAGGCCATGATGGACTTCACAGGTGGCG
TCCACATTTACATCCAGCTATCTGAGCCTCTGTGGGAGCTGATGTGCAGAGCTGGAAAATCTGGAGTACTGATGTGCTGCTTGTCACCAAATGGGCTGGTGCTGGGACATGCCTATACTGTGACTGGTGTCATACAGTTGGTGAG
TCGGGGGAAACCTGTGAACCTGGTGCGTTTGTGGAACCCCTGGCGCACAGGAGAGTGGAGAGGAGACTGGTGCGATCGGTCCCCTTTGTGGCAAACTGTGAGCCCTAAGGATCGGGAAAAGTGCCATGACGATGATGGAGAGTTC
TGGATGACACTGGAGGACTTCTGTAAGCTGTACAGTGATCTTGACATCTGCGGCATGGATCCAAACTTCCTCGATTGCCAGTGGAAGACATCCGTGCACGAAGGCCGCTGGGAAACTTTCTGGACAAATCCTCAATTTAGAGTCC
AAATT------
AAAAACACGTTTGTGTCTTTGATTGACCAGCACATCGGATTTTCCATCTTTGAAGTGATGGAATTCATCAGGCTGAAGCCCGGAGAGTACCTGATCGTTCCGACCTTCAAACCCAATGAGAGCGCCTCTTTCCTCCTGACCATCC
ACTCCAAGTATGAGGAAGTGGATGCTGAGCAGCTCCAGAAGATCCTGAATGACCAAATTCTGAAAGGATTCAGCATCGATGCCTGTCGCAGCATGGTTGCCCTGATGGATTCATCAATAACAGGCAGCCTGAACAGCAAGGAGTT
TGTGCGACTGTGGAACAAAGTTATTGCATTCAAGGACATTTTCTTTCAGACAGATGTTTCACGGACAGGAACTCTTTCGCTCAGTGAGCTAAGAAATGCTATTATA------------
GGAATAAAGGTCAACGACGAGCTGCTGAATCTGATGGCTCTGCGCTACAGCGCTGTGACCCTGGAGAGCTTCATCAGCCTCATCCTGCGCTTAGAATGCATGTGCAAAATCTTTAAACAGCTGTCC---
GATGGGAAAACAATGACCCTTCAGGAGTCATAC---

GGCCAAGACTATGAGCAGTTGAAACAGTACTGTCTCACAAGAGGCGTAAGCTTCACTGATGAGACATTTCTACCTGACAGAAGGTCCATTGGCATACTGAGCCCTTCTGAACTGGCCTGTGTGGAGTGGCGGAGACCACCAGCCT
TTGTACTTGACGGAGTCTCCAGGTTTGATTTTGGTCAAGGGAAACTTGGAAACTGCTGGTTTCTTGCGTCAATCGGAGCGCTGACGTTCCAGAAATCCATTTTCGCAGAAGTTGTCCCACTTGAACAAAGATTTGATAACTACTG
TGGGATTTTTCACTTCAGGTTCTGGAGATTTGGGCAGTGGGAGGATGTGGTCATTGATGACAAACTACCAACA---
GGCAGACTAATCTTTGTTCATTCCAAAGACCAAAATGAGTTCTGGCCTGCTCTCCTGGAGAAAGCCTATGCCAAGGTTTGTGGTTCCTACTCCGACATGATTGCTGGAACTCTCTCAGAGGCTATGATGGACTTCACCGGTGGCG
TCCACATTAACATCGAGCTGTCACGCCCGCTGTGGGGGCTGATCTGTAGAGCTGGCAAATCAAACACACTGATGGGCTGTATATTACCAAATGGACTGGTTGCAGGCCATGCATACACTGTGACAGGTGTCAAAGAGATGATGAG
TCGAGGAAAAATTGTATACCTGGTGCGTTTGTGGAATCCCTGGGGTAACGGAGAGTGGAAAGGAGACTGGAGTGATCGGTCTCCTCTGTGGCAAACTGTGAGTCCTGAGGACCGTGAAATGTGCCTTGCTGATGATGGAGAGTTT
TGGATCACCTTTGAAGATTTCTGTAAGTTCTACACAGATATTGACATCTGTGGGCTGTCTCCTGACTTCCTTAATAGTCAGTGGAAGACCTCTGTGTATGACGGCCGATGGGACAGTTTCTGGACTAATCCACAGTGTCGTTTCA
AAATT------
AAAAACATAATGGTGTCTCTCATGGAACTCCACATTGGGTTTTTCATATTTGAGGTGATGGAGTTCTTTATGTTAAAGCATGGTGAATACTTGATTGTGCCAACCTTCAATCCCAATGAGACAGCCTCCTTTATCCTGACCATCC
ACGCAAAATATGAAGAAGTGGATGCTGAGCAGCTTCAGAGGCTTCTAAATGAAAACATCCTAAAAGGTTTCAGTATTGACGCCTGTTGCAGCATGGTTGCACTAATGGATGTATCCTGTTGTGGAAAACTCAACAGTGAGGAATT
TCTTCGTCTGTGGAACAAAGTTGTTACATTCAAGGAAATTTTCTTTCGCACTGACATTTCACGAACAGGAACGCTGTCACTGAGGGAACTACGAAATGCATTCTTA------------
GGAATAAGGGTGAGTGACGACATGCTGAATCTGATGGCTCTGCGCTATGGCGCCATGACGCTGGAGAGCTTCATCAGTCTCATCCTTCGCTTTGACTGCATGAACAAAATCTTCAGACAGTTATCT---
GATGGAAAAGCCATGACTCTTCAAGAGTCTTACACT

GGACAGGACTTCCGGCAGCTGAAGGAGTTCTGTGTCGGCAGGCGGCTCAAGTTCATCGACGACATGTTCCCTCCTGACAGCAAGTCCATCGGAGTCCTGAGCCCCTCGGACCTAGCCCGGGTCGTCTGGCTCCGGCCACCGTCCT
TCGTCCTGCAAGGGTTCTCCAGGTTTGACTTTGGTCAAGGAGTTCTGGGAAACTGCTGGTTTCTGGCGTCGATCGGAGCGCTGACGTTCCAGGATCAAATCCTCCAGCAGGTCGTCCCTCAGGAACAAGACTTTGATAACTACTG
CGGCCTGTTTCATTTCAGGTTCTGGAGATTTGGGAAGTGGGTGGACGTCGTGATTGATGACAAGCTGCCAACC---
GGCAGCCTGATCTTCGTTCATTCCAAGAAACAGAACGAGTTCTGGCCTGCCCTGCTGGAGAAAGCATATGCCAAGGTGTGTGGCTCCTACACCGACATGAATGCTGGGACTCCTGCAGAGGCCATGATGGACTTCACTGGAGGCG
TCCACATGAGCATCCAGCTGTCGGATCCTCTGTGGGGGCTGATGTCCAGAGCCGGGAACTTTAGCGCTCTGCTCAGCTGTTTGTCTCCCAACGGCCTAGTCCAGGGCCACGCCTACACTGTGACTGGCGTTGTCCAGATGATGAG
CAGAGGGAAGCCGGTGAAGATGCTGCGTCTGTGGAATCCCTGGGGAAAAGGAGAGTGGAACGGAGACTGGAGCGACCGCTCGTCTCTGTGGCAAACCGTGAGCTCCCAAGATCGAGAGAAATGCCTTGTCGACGACGGAGAGTTT
TGGATGACACTGGAGGACTTCTCCAAGTTCTTCACAGATCTTGACATCTGTGGTCTGAACCCGAACTTCCTGGACTCCCAGTGGACGACCTCTGTGGCGGAGGGCCGGTGGGACAGCTTCTGGACCAATCCGCAGTACCGAGTCA
AGCTG------
AAAAACATCCTGGTGTCTCTGATGGACCTCCACATCGGCTTCTCCATATTTGAAGTGATGGAGCTGCTGACGCTGAAGCCTGGAGACTACATGATCGTCCCGACCTTCCATCCCAACGAGACGGCGTCCTTCCTCCTGACCATCC
TGTCCAAGTATGAAGAGATGGATGCAGAGCAGCTCCAGAGAATCCTAAACGAGAACCTTCTGAAAGGTTTCAGCATCGACGCCTGTCGGAGCATGGTGGCCCTCATGGACACGTCAGTAACTGGGAGACTGAACAGCAGTGAGTT
CATCCGACTGTGGAACAAAGTCATCATTTACAAGGACATTTTCTTTCAAACTGATGTTTCTCGGACTGGAACCCTGTCGCTGACGGAGCTCAGGAACGCACTCGTC------------
GGGATGAGAGTCTCTGACGACATGCTGAACCTGATGGCGCTGCGCTACGGCGCCATGACGCTGGAGAGCTTCATCAGCCTCATCCTGCGTCTGGACTGCATGTTCAAGATCTTCCGGCAGCTGTCT---
GATGGGAAAACGATGAGCCTGCAGGAGTCATACACG

AACCAGGACTTCCAGCAGCTGAAGGAGTACTGCGTCATCCGCCGGGTGAGGTTCATCGACGACATGTTCCCCCCCGACCACCGCTCCATCGACGTCCTGCAGCCGGCCGACCTGGTCCGGGTGGAGTGGGTCAGACCGCCTTCCT
TCATTGTTGACGGAGTCTCCAGATTTGACTTTGGCCAAGGAATGGTTGGAAACTGCTGGTTTCTTGCCTCCATCGGAGCGCTGACCTTCCAGGACTTGGCCCTGAAACAAGTCGTTCCTCTTGAGCAAACATTTGATGATTACTG
CGGCCTGTTCCACTTCAGGTTCTGGAGGTTTGGAAAATGGGTCGACGTAGTCATTGACGATAAGCTGCCAACA---
GGCCAGTTAATTTTTGTCCACTCTAAGAACCCGACTGAGTTCTGGCCCGCTCTGATGGAGAAGGCCTACGCCAAGGTCTGTGGCTCCTATGCAGACATGAATGCAGGAACTCCAGTGGAGGCCTTGGTGGACTTCACCGGCGGCG
TCCACATGTGTGTGCAGCTGTCTGAGCCGCTGTGGGAGCTGATGTGCAGAGCCAGCCAGGCCAAGTCGCTGATGGGCTGCGTCTTACCAAACGGCCTAGTTCAAGGCCACGCCTACGCCGTCACTGGAGTCAAGGAGGTGCTGAG
CAGAGCGAAGGCTGAGCGTCTGGTGCGTCTGTGGAACCCCTGGGGCCAAGGAGAGTGGAGCGGAGACTGGAGCGACCGGTCACCTCTGTGGCAGACGGTGAGTCCTCAGGATCGGGAAACCTGCCTTCGTGAAGACGGCGAGTTC
TGGATGACTCTGCAGGATTTCTGTAAGTTTTACTCTGACCTGGACATCTGCTGCTCGTGTCCGGACTTCCTCGATTGTCACTGGAAATCCTCGTTTTACGAAGGCCGATGGGGGACCTTCTGGACCAATCCGCAGTACCGGGTGG
AGGTT------
AAGAACATGCTGGTGTCTCTCATGGACCTCCACATCGGCTTCTGTGTGTTTGAGGTGATGCAGCTGCTGAGCCTGAAGCCCGGAGAGTTCCTGATCGTCCCGACCTTTGGCCCCAACGAGACGGCGTCCTTCATCCTCACCGTGC
TGTCCAAGTACGAAGAAGTGGACGCCGAGCAGCTGCAGAGCCTCCTGAACGGCAAAATCCTGCGAGGCTTCAGCACCGACGCCTGCCGCAGCATGGTGGCCCTCATGGACACATCCATCACTGGGAAGCTGAACAGTGAGGAATT
TGTCAGTTTGTGGAGGAAGATTGTCTCCTACAAGGACATCTTCTCTCACTGCGACGTCTCTCAGACGGGGACGCTGTCGCTCAGCGAGCTGAGGAAGGCCATCATG------------
GGAAAGACGATCAGTGACGGCATGCTGAACATCATGGCTCTGCGCTACGGCGCCATAACGCTGGAGAACTTCATCGCTTTGGTCCTTCGATTTGACCGCATGTACCAAATATTCAGAGAGTTATCT---
GATGGAGGAGCCGTGACTCTTCGCGAACCATATTCC

AACCAAGACTACGAGCAGCTGAAACAGTATTGTCTCATCCAAGGGGTGAGGTTCATCGACAAAATGTTCCCCCCCGACCAAAGGTCCATCGACGTACTCAAACCTGCTGACTTGAGCCGAGTGGAGTGGCTCAGACCCCCATCTT
TTGAAGTTGACGGGATCTCCAGGTTTGACTTTGGTCAAGGAATCGTTGGAAACTGCTGGTTTCTCGCGTCTATCGGATCTCTGACGTTCCAGCAACAAATCTTTCAGCAAGTTGTTCCTCTTGAGCAAAGGTTTGATAAATACTG
CGGGCTGTTCCACTTCAGGTTTTGGAGATTTGGGAAATGGGTGGACGTCGTTATTGACGATAAGTTACCAACG---
GGCCAACTGATCTTCGTTCAGTCCAGTGACCCGACTGAGTTCTGGCCTGCTCTGCTCGAGAAGGCCTATGCCAAAGTGTGTGGCTCTTACTCAGACATGAATTCAGGAACCCCTGATGAGGCTTTGGTGGACTTCACCGGAGGTG
TTCACATGTGCATTGAACTGCAAGACCCTCTTTGGGAACTGATGTGTAGAGCTGGCCAGTCCAAGTCCCTGATGGGCTGTGTGTTACCGAATGGATTGGTCCAAGGCCACGCCTACGCTGTCACAGGGGTCAAGCAGATGATGAG
TAATGGGACACCAGTACAGCTGGTACGTCTGTGGAACCCCTGGGGCGCAACAGAGTGGAACGGAGCCTGGAGTGATGAGTCGTCTTTGTGGCAAACTGTGAGTCCTCAAAATCGTGAGATGTGCCTTGGGGATGATGGTGAGTTT
TGGATGAACCTGAAGGACTTCTGTAAATTCTACTCATCACTGGACATCTGCTCCCTGTGTCCCGATTTCCTGGATTGTCATTGGAAGACCGCCTTCTATGAAGGACGATGGGACAGTTTCTGGACTAACCCACAATATTGTTTCA
AGATTGATGAAAAAAACATGCTGGTGTCTCTCATGGACCTCCACATTGGATTCTCTGTGTTTGAGGTGATGGCGTTCTTTGCACTGAAGCCCGGCGAATACCTGATAGTGCCGACCTTCAGTCCAAATGAGACGGCCTCCTTCAT
CCTGACCATCGTCTCCAAGTATGAGGAGGTGGATGCCGAGAAGCTCCAGAGGCTTCTGAATGAACACGTCCTGAAAGGCTTCAGCATTGACGCCTGTCGCAGCATGGTTGCTCTGATGGACACATCCATCACCGGAAAACTGAAT
GATGAGGAGTTTGTTCATCTGTGGAACAAGGTCGTCGGGTACAAGGATGTTTTCTTCCGCTCTGATGTTTCACAAACAGGAACACTTTCCCTGATTGAGCTGAGGAACGCAATCACG------------
GGAAACACGATCAACGATGAGATTCTGAACCTGATGGCGTTGCGCTACGGCGCCATCACGCTGGAGAGCTTCATCAGTCTCATCCTTCGCTTCGACCGCGTGAACCAAATCTTCAGACATTTGTCT---
AATGGAAAGGACATGACTCTTCGTGAACCATACACC

CAGCAGGACTACCTGCAGCTGAAACGGTTCCTCCTGACCCAAAACAAGCTGTTCAGAGACGAGACGTTCCCTCCGGACCAGCGCTCCATCGGCAAGCTGGAGCCGGCGGAGCTGGCTCAGGTGCAGTGGCTCAGACCACCGTTCT
TCATCCTCGATGGCGTCTCCAGGTTTGACTTCGGTCAGGGACAAGTTGGAAACTGCTGGTTCCTGGCGTCTCTCGGAGCGCTGACCTTCCACAAAGAGATCTTCAAGCTGATTGTTCCTCTGGACCAGACCGTGGGGGACTACTG
TGGGCTGTTTCACTTCAGGTTCTGGAGGTTTGGGAAGTGGGTGGACGTGGTGATTGACGACAAGCTGCCAACA---

CGGAAACCCATCTTTGCCCGTTCCAAAGACGAGCGTGAGTTCTGGCCCGCTTTGCTTGAGAAGGCCTACGCCAAGGTTTGCGGATCCTATGCGGATATGACGTCTGGGACTCCGGCAGAGGCCATGAGGGACTTCACCGGCGGCG
TCCACATGTGCATCCAGCTGTCAGATCCTCTCTGGAAGCTCCTGTGCAGAGCCGGACGCTCCAGGACGTTCATGAGCTGCAGGCTACCGAACGGACTGGTCCCGGGCCACGCCTACACGGTGACGGGTCTGAAACAGCTGCAGAG
CCAGGAGACGGAGGTGAACCTGGTGCGCCTGTGGAACCCCTGGGGGCACGGAGAGTGGAACGGAGACTGGAGCGACAAGTCTCCTCTGTGGCGAAGCGTCAGCACAAAGGACCGCGAAAAGTGCCTCGAGAATGATGGAGAGTTT
TGGATGAGCCTGGAGGATTGCTGCAGATATTACACCAACATTGAAATCTGCGGCATGAGACCCGACTTCCTGGACTGCCACTGGAAGACGTCCATGTACGAGAACCGCTGGGAGACTTTCTGGACCAATCCCCAGTATCGGATCA
AGGTC------
AGGAATACTCTGGTGTCTCTCATGGACTTCTTCATCGGCTTCATCATCTTTGAAGTGACGGAGTTCCTCACGCTGAAGCCCGGAGAATACGTCATCGTGCCGACCAACGAGCCCAACCAGACCGCCTCCTTCCTCCTGACCATCT
TCTCCCGGTATGAAGAAGTCGATGCTGAGCTCCTGCAGCAGCTTCTGAAA---------------
AGCTTTAGCATCGATGCATGTCGCAGCATGGTGGCTCTGATGGATGAATCTGGAGATGGAAAGCTGGACAGTCAGGAGTTTGGCTATCTGTGGCACAAGGTTATGAAATATAAGCGAGTTTTTGCCAAAATGGATGTTTCTCAAA
CGGGAACGCTGTCACTGACGGAGCTGAGGAACGCTTTGAGA------------
GGTATGAGCATCAGCGATGAACTGCTTAACCTGATGGTGGTTCGTCACGGCGCCATGACCCTGGAGAACTTCATCAGCCTCAGCCTCCGCCTGAGCCGCATGAACAAAATCTTTACGGAGCTGTCG---
GATGGGAGAAACGTCACGCTGTCCAGGTCATACACC

GATCAGGACTTCCAGCAGCTGAAGCAGTTCTGCCTGAAGGAGAGACTGAGGTTTAGAGACAACCTGTTCCCACCAGAGCTCAAATCCATCGGGCCTCTGAAGCGGGACGATCTCTGGAAAGTGGTGTGGAAAAGACCACCTGTTT
ACATCGCTCAAGGCACTTCAAGGTTTGACTTCATCCAGGGCAGATTAGGGAACTGCTGGTTTCTGGCATCTGTCGGAGCGCTTACATTTCAGAAGCGCATCATGAAGCAGGTCATTCAGGACGATCAAACGTTCTCTGATTATGC
TGGAATATTTCACTTTAGGTTCTGGAGATTTGGATCATGGGTTGATGTGGTTATCGATGATAAACTGCCAACT---
AACCAACTTGTCTTTGTCCAGTCCAAAACACCGAATGAATTCTGGCCTGCTCTGTTAGAGAAAGCTTATGCCAAAGTGTGTGGCTCTTATGCGGATATGGACGCAGGGAACATCTCAGAGGCTCTGATGGATTTCACCGGTGGTC
CACACATGACCATTAAACTGAGTCAAGCGCTGTGGGACATCATGAGACGAGCCGGCAAATCAGAATCACTGATGGGATGCGTGTTGCCCAATGGTCTTGTGGAAATGCATGCGTACACGGTCACAGGAGTCACAGAGGTTGTATG
TAAAGGTCGTCCAGTGAAGCTGGTGAGAATTTTTAATCCGTGGGGTTCTGGAGAGTGGAACAGAGACTGGAGCGACAGATCTCCACTGTGGGAACTTGTACGTCCCGAGGAC---
CAAAAATACAATCTCGACAATGGAGAATTCTGGATGTCAATGGAGGATTTCTGCAGGAATTTTTCTGAAATGGATATCTGTTGTTCAGATGTGAATGTCCTTGCCTCCTCCTGGAAAACTGAAGTGCACAAGGGCCAGTGGGACA
GTTTTTCGAAGAATCCTCAATATCGTGTGACTCTTAAAGAGGCAAATCTCCTGGTGTCTCTCATTCACTTCCACATCGGCTTTAACATTTATGAGGTGATGGAGTTTTTCAGATTTAAAGCAGGCGACTACCTCATCGTTCCCAC
TTTCCAGCCCAATGAAGCTTCGTCTTTCCTTCTGACGGTCTATTCAAAGTATGGTGAAGTGGACGCTGAGCGGCTTCAGAAACTCCTGAACGAGAATCTTCATGCAGGATTCAGTGTGGATTTGTGTAAGAGTCTGGTGGCGCTG
ATGGATCTCAGTGTCACAGGAAGACTCAGTGAATTTGAATGCCTTCGTTTGTGGAAACGAGCCGTTTTTCTAAAGGATATTTTCTACGACATGGATGTGTCACACACTGGAAGTCTCTCTGTCAATGAACTCCGAAATGCTCTTA
AA------------
GGGTTTGTATTGAGTGACGGTATGCTGAATCTCATGGCTCTCCGATACGCTGACATTTCTCTGGAAAACTTTATTGTGCTCGTGCTGCGCATGGACTGCATGGCCAAAACATTCAAGAGACTCTCTGCTGGGGGCCCAAACATGA
TGCTGGGAGAAAATTACTCA

GACCAAGACTACAACTTCCTGCAAGACTACTGTCTCAAGACAAGGCAGAGATTTGTTGATGAGTTTTTCCCACCAGACCTTCGTTCCATTGGGCTGCTGAAGCCTGAGGTCATGGCCCGAGTGGAGTGGATTAGACCAGCTGAAT
TCGTTGTAGATACGGTGTCCAGGTTTGACTATGCCCAAGGGTTTATAGGAAACTGTTGGTTTCTGGCCTCTGTTGGTGCCTTAACATTTCAAAGCAAATTACTGCAAAAGGTTGTTCCAGATGGACAGTCACTCAGAAATTACAC
TGGCTTATTTCACTTCAGGTTCTGGCGCTTTGGAAAATGGTTCGATGTAGTGATTGACGACAAGCTGCCAACA---
CGCCAGCTGATCTTTGTCAAATCTAAAACATATAATGAGTTCTGGCCAGCCTTGCTGGAGAAAGCATATGCCAAGGTATGTGGCTCCTATGCTGACATGCACACCGGCCGAGTGTCTGAGGCCCTGCTGGACTTCACTGGCGGGG
TTCATATGCACTACGATCTGAAAACGGCTCTGTGGGAGATAATGTACCGTGCTTCCCAGTCAGAGGTCCTTATGGGCTGTCGGTTACCAAATGGCATAGTTCTGGGCCACGCTTACACTGTGACAAAGGTCTACCAGGTCATGAG
TGGTAGAAATCCTGTTCAGCTGGTGAGATTGTTCAACCCATGGGGAGACAGTGAGTGGAATGGAGACTGGAGTGACAATTCACCTTTATGGAACACGGTCGATGATGAGGACCGCAAACAGCTTCTCTCTGATAATGGAGAGTTC
TGGATGTCAATGAAAGATTTCCTTAGAACATTTGACAACATGGACATCTGTTGTAACTGTCCTGATTTTCTGGAATGCAAGTGGACCTCCAAGTCCCACAACGGCAGCTGGGACACCTTCTGCACCAACCCCCAGTTCTGGCTGA
GAATTAATGAGAACAATGTTCTGGTGTCCCTCATAGACCACGGAATTGGCTTCTGCGTGTTTCAGGTGATGAAGTTCTTCAGGCTGGAACCAGGAGAGTATCTCATTGTACCGACTTACTTGCCTAATAAGAACGCAGATTTCAT
TCTGTCCATCCTCTGCAAGTACAAAGTTGTGGATGCTGAGAAACTCCAGCAGATTCTTCATGAAAATCTGCTTAGAGGATTTGGGCTGGATTCCTGTAGAAGTATGATTGCCATGTCAGATTTTTGTGTCACTGGAAGACTTCAG
GGATCAGAATTTGTTCGTCTGTGGGACCGGATTACAACCTACAGGGACATCTTCTACAACATGGACTCTTCTAAAGATGGTGTTCTGTCTTTGAATGAACTTCAAAATGCATTAGAG------------
GGCCTACATCTAAACGAAGACATCTTGAATCTCATGGTGGTGCGGTACGGTGGTATCTCGTTGGAGGGATTCATATGCCTTGTCATGCGTTTGAACTGCATGGCCAGCATATTTCAGAAACTCTGTGAAAATGGGAAG---
ATAAGTCTTGATGAGAGTTACTCA

GACCAGGACTATGCACAGCTGAGAGACACCTGTCTCCACACGGGGAGCCTGTTTGTAGACAGCACTTTCCCACCTAACAGCCAGTCTCTGCCCGATCTCAGCAGCTGGCGCGAGGCCCAAGTGGAGTGGCTTCGACCAGCCGTTT
TCATCCTGGATGGAGCTTCGAGGTTTGACTTCAGTCAAGGCGCCGTGGGTAACTGCTGGTTTCTGGCTGCGATCTCTGCGCTCACATTCCATAAAAGCGTGCTGGGACAAGTGGTTCCCACAGAGCAGTCTTTT---
AACTACGCCGGGATATTTCACTTCAGGTTCTGGAGGTTTGGGGAGTGGGTGGACGTGGTCGTTGATGACTACCTGCCAGTG---
GGAACTTTACTGTCTGTGCGCTCCAAAGGAGGAATTGAGTTCTGGGTGGCCTTACTGGAGAAAGCATATGCCAAAGTGTGCGGTTCTTATGCCGACATGAACGCCGGGCTGCCTTCGGAGGCCTGCAAGGACTTCTGCGGCGGCA
TCAACATGATCTACGAGCTCAAGGACGCGCTGTGGCTCACGCTGGAGAGGGCCACAACAAGTCACGCGATGATCTGCTGCGTATCGCGCACCGGGCTGGTGGACGGCCACGCCTACACCATAACCGGGGTCACCAAGGTGAATTG
TTTTGGGTGTGACGTGAAGCTGGTGAGGCTGATGAACCCCTGGGGCAAGCAAGAATGGAGCGGGAAGTGGAGCGACAAGTCCAGCGAGTGGAACAGGGTGAGCGCAGAAGATCAGAAAAAGCGCAATAGAGAGGACGGGGAATTC
TGGATGGACCTGGAAGACTTCTGCTACTATTTCCAAATGTTGTTCATCTGCGGTGAGAATCCCAGCTTTATCGACTGTCGCTGGAAGTACCAGATCTACCAGGGCAGCTGGTATACTTTTGACCAGAATCCTCAGTACCGCATCC

AGGTG------
AGGAACGTGTTGTTTTCTCTCATGCAGCTCCCCATTGGACTGAGCATCTTTGAAATGACTGAGTTTTACAGCCTGGAGCCAGGAGAGTACGTGGTGGTCCCCACCATGAGGGCTTACATGAACGCAGACTTCGTTCTCACCGTCT
ACTTCAAA---
GATAAGTTGAACGCCAGGCAGCTCCAGAAGCTCCTGAATGAAAACTTTCCTCGAAACTTCTCTCTGGACAGCTGCAGGAGCTTGATCGCTCTGATGGATGCGAAGAAAAAGATGAAGATGAACATCTCCGAGTTCTCGGCTCTCT
GGGAGAAGATCGACAAATTCAAAAACCTCTTCCAAAGCTCAGATACAAACAGGAATAGGTACCTGAATAAGTATGAGCTGAAGAAAGCCCTGAGT------------
GCAATGGAGCTGAGCGACGAGACGGTGGACCTGCTGATGTACCGCTATTCCGACTTGTCGCTCAACGGCTTCATCCCCTTCATGATGCGCATGGACAGGATGCTGAATGTGTTCAATGAAAACTCAGGTAAACAC------
ATGCACCTAAGCTTCGAAAAT---

AACCAAGACTACGCGCAGCTGAGAGATAACTACCGCCGCAGAAGGACGCAGTTTGTGGACAACACCTTCCCCCCCAACAACCAATCTCTGCCTAATTTAAGCCGTTGGCAGGAAGCCCAAGTGGAGTGGCTCCGACCACCTATTT
TCTGTAGCAAAGGAGCCTCCCGGTTTGACTTTGATCAAGGCAGCGTGGGTAACTGCTGGTTTCTGGCTGCAATTTCCTCCCTGACATTCCACAAAGGCCTGTTAGCTCAAGTTGTGCCTATGGACCAGAACTTC---
GATAATGCCGGGATATTTCATTTCAGGTTCTGGAGGTTCGGCGTGTGGGTAGATGTTGTCATTGATGACCACCTGCCAACG---
AATGGCTTGATGTTTGTCAGCTCCAAGGATGGAACCGAGTTCTGGGCTCCTCTGCTGGAGAAAGCTTACGCCAAAGTGTGCGGCTCATACGCGGACATGAGCTCTGGATTGCCGTCAGAGGCCTGCAAGGACTTCAGTGGAGGCT
TGAACATGGATTACAAACTCCATGAGGCCCTGTGGAACGCGCTGAGCAGAGCCACCAGGTGCCAGTCGATGATCTGCTGC------
GACACTGGGATTGTGGATGCACATGCTTATTCCGTGACAGCAGTCACTGAGGTGGATTATTACGGCTCTAAAGTGAAGCTGGTGCGTCTCATGAATCCTTGGGGCCGGACGGAGTGGAAGGGGAAGTGGAGTGACAAGTCTGACA
TGTGGAGTAAAGTGAGGCCAGAAGATCGGAAAAACTTTGTTCGCAATGACGGAGAGTTCTGGATGGAGTTGGAGGACTTCTGTCATTATTTCCAAAACTTGTTCATCTGCTGTGAGAACCCCAACTTCATTGACTGCCAGTGGAA
ATCCATGACTTACGATGGCAGCTGGTCTACCTTTGAGGCGAACCCCCAGTATCGCTTGCAGGTT------
CAAAACATCTTCCTTTCTCTGATGCAGCACCCGATTGGACTTACCGTCTTTGATCTGATTGAGTCGTACAGTCTGGAGCCGGCCGAGTATCTGATCGTCCCCACTCTCAAGCCCAACATGAGTGGAGACTTTGTTCTCACCGTCT
ACACCAAGTTTGGTCGGCTGGCGGCCGGACAGCTCCAGAAGCTCCTGAATGACAACTTCCCTCACGGTTTTGGTTTGGATACCTGCATGAGCATGATGGCCATGGTGGATACCAACCAAACAATGAGGATGACTTTCACGGAGTT
CTCAACTCTCTGGGAGAAGATCACCAAATACAAGAAACGCTTTCATCTTGCGGATGTGAATGAGAATGGATCTCTGTCTGAACGTGAGCTCCAGAAGGCCATGGAG------------
GGAATGGACACTGACGACTTCATGGTGAGGCTGATGACGGCCCGCTACTCGGGG---TCCATGGAGAACTTTATCACTCTCATGTTACGCCTGGAGAAGATCTCGGATGTTTTCAAGGACAAATCCTCAAACGGAGTG---
ATCCACTTGTCCTGGACCTACAAC

AATCAGGACTTCGCTCAGATAAAAGCTGACTGCCTCCAGAAAGGAGAACTGTTTGTGGACAATGAGTTCCCACCAAACGGTCGTTCTTTGCCTGATCTGAGTTCCTCACAG------------
GATTTATTGAGAAAACCTGCTTTCAGTAAAGATGGTATGTCACGATTTGACTTTGGACAAGGCGACGTGGGTGACTGCTGGTTTTTGTCTGCAATATCTGCACTGACCTTCCAAGAAAATCTGATGGCACAAGTTGTACCAATGG
ATCAGTCCTTT---AACTATGCAGGAATATTTCACTTCAGGTTCTGGAGGTTTGGAAAGTGGGTAGATGTTGTCATTGATGACAATCTGCCAACG---
AAGCGGTTACTGTTTGTGTCCTCAAAATGTGGAAATGAGTTCTGGGCTCCTCTGCTGGAGAAGGCATATGCCAAACTTTGTGGCTCGTATGCAGACATGCATGCTGGGTTTCCATCAGAGGCCTGCAAAGATTTCACTGGAGGTG
TAAACCAGACTTACAAACTGAAGGAGATGCTTTGGCTCACATTAAACAGAGCAACTAGGTGCCAATCACTGATTTGTTGTGTCTCTGACACTGGATTGGTGAATGGGCATGCCTATTCCATCACAGGTGTCACTAAGGTGGAATT
AAACGGCTCCAAAGTCCGACTGGTGCGAGTCATGAACCCATGGGGTGAACGGGAGTGGAACGGAAAATGGAGCGACAAGTCAGATTTATGGGACAGAGTAAGTCCAGAAGTTAAAAAAAAGTGTTTCCGTGACGACGGAGAGTTC
TGGATGCAAATGGAAGACTTCTGTTCCTATTTTGCTTACGTATCCATCTGCTGTGAGACCCCTAACTTTCTTGATTGTCAGTGGAATTGCATGATTTACGATGGCAGCTGGACCACCTTTGCAACAAACCCTCAGTATCGCATCC
GAGTG------
AAAAACATCGTGCTCTCCCTGATGCAGCACCGAACTAGACTTACCATCTATGATCATATTGAACTCCACAGTCTGGAACCTGGAGAGTACGTGATCATCCCAACCTATAAACCCAACATCACTGCAGATTTCGCTCTCGCTGTCT
ACACCAAGAATGATGAGCTGCTTACTGGACAACTCCAGAAACTCCTGAATGACAGGTTCCCTCATGGCTTTGGTTTGGATACCTGCAGGAGTATGATTGCTTTGGTGGATCTTGACCAAAGGATGACAATGTCTTTTATTGAATT
TTTGATACTTTGGAACAAGATTCAAGAATACAAGAAACTCTTCCATCAATCTGATCTTAATCAAAGTGGATCCTTGACTGACCTTGAGCTCCAGAAAACAGTTGAG------------
GGTCTAAACGTGAATGACAAGACGGTGAGGCTGATGATGTTTCGGTATTCACGC---ATGTTGGAGGATTTCATCACTCTCATGTTGCGACTGGATAAGATGTCAAACATTTTCAAGGACAAATCCTCTGATGGAACT---
ATGCGCTTGACCTGGGATTACAAC

AATCAAGATTTTGCTCAGATAAAAGCTGACTGCCTCCACAGCGGGCGACTGTTTGTGGACAACACCTTCCCACCTAACAGTGGTTCTTTGCCTGATCTGAGCACCTCGCAGGAGAACGACGTGGAATGGCTTCGACCGCCTGCTT
TCTGTATCGATGGAGCCTCACGATTTGACTTCGGGCAAGGCCTTGTGGGTAACTGCTGGTTTCTTTCTGCAATATCAGCACTGACGTTTAAAAAAAAACTGTTGGCACAAGTTGTGCCGTTGGACCAGTCCTTC---
AATTACGCAGGAATATTTCACTTCAGGTTCTGGAGGTTTGGAAAGTGGGTAGATGTTATCATTGATGATTATCTGCCAACA---
AAGCAGTTGTTGTTAGCACATAGCAAATGTGGAAATGAGTTCTGGGTTCCACTGCTGGAGAAGGCATATGCCAAAATTTGTGGTTCATATGCAGACATGCATTGGGGGTCACCATCAGAATCCTTTAAAGACTTCACTGGAGGGG
TGACCATGACTTACAAACTCAGGGAGGCCCTGTGGCTCACGCTCAAGAGAGCTATTCAGTGTAAATCACTGATTTGCTGTGTTTCTCACACTGGACTGGTGGATGCTCACGCTTATTCTGTCACAGCTGTCACTGAGGTGGAGTT
ATACAGCTCCAAAGTAAGGCTGGTGCGACTCATCAACCCCTGGGGCAAACAGGAGTGGAATGGAAAGTGGAGCGACAAGTCAGATTTGTGGTACAAAGTGAGGATAGAAGATCGGAAAAAATGTTTCCGCGAGGACGGAGAGTTC
TGGATGCAACTGGAGGACGTCTGTTACTATTTCAGCTATTTATCCATCTGCTGTGAGAACCCTAACTTCATAGACTGTCAGTGGAAATGCATGACTTACGATGGCAGCTGGTCCACCTTTGCAACAAACCCTCAGTATCGCATCC
AGGTA------
AAAAACGTCTTGCTCTCCCTGATGCAGTACCCCATTGGACTAACCATTTATGATCTGACCGAACTACACAGTCTGGAACCTGGAGAGTATGTGATCATCCCGACCATGAAACCCAACATGACTGCCGATTTCGTTCTTACTGTCT
ACACCAACAATGGTGAGCTGCTTGCTGGACAACTCCAGAAACTCCTGAATGACAGGTTCCCTCATGGCTTTGGTTTGGATACCTGCAGGAGTATGATTGCTTTGGTGGATCTTGATCAAAGGATGACAATGTCTTTTACTGAATT
TTTGATTCTTTGGAACAAGATTCAAGAATACAAGAAACTCTTCCATCAATCTGATCTTAATCAAAGTGGATCCTTGACTGACCTCGAGCTCCAGAGAGCAGTTGAG------------

GGTCTAAACGTGAATGACAAGACGGTGAGGCTGATGATGTTTCGGTATTCACGC---ATGTTGGAGGATTTCATCACTCTCATGTTGCGACTGGATAAGATGTCAAACATTTTCAAGGACAAATCCTCTGATGGAACA---
ATGCGCTTGACCTGGGATTACAAC

AATCAAGATTTCGCTCAGATAAAAGCTGACTGCCTCCGCAAAGGAGGGCTATTTGTGGACAATGCGTTCCCACCAAACAGTCGTTCTTTGCCTGACATGAGTCCCTCACAGGAGAGTGAAGTGAAATGGCTTCGACCACCTGCTT
TCTGTACAGATGGTACATCACGATTTGACTTTGGACAAGGTGAAGTGGGTAACTGCTGGTTTTTGTCTGCAATATCTGCACTGACCTTCCAAAAAGGTCTAATGGCACAAGTTGTGCCAATGGACCAGTCCTTT---
GATTATGCAGGAATATTTCATTTCAGGTTCTGGAGGTTTGGAAAGTGGATAGATGTTGTCATCGATGATTATCTGCCAACG---
AAGCGGCTGCTGTCTGTGTCCTCAAAAGATGGAAATGAGTTCTGGGCTCCTCTGCTGGAGAAGGCATATGCCAAAGTTTGTGGTTCATACGCAGACATGCATGGTGGGTCATCATCAGAGGCTTGCAAAGATTTCACTGGAGGTG
TGAACCAGATTTACCATCTCACAGAGGCCCTTTGGCTGACACTGAGCAGAGCCACTGAGTGCAAATCATTGATTTGCTGTTTGGCCAACATTGGATTGGTAGATGGGCACGCTTATTCTATCACAGAAATCACTGAGGTGGAATT
AAACGGCTCCAAAGTAAGACTGGTGCGAATCATGAACCCATGGGGCAAACGGGAGTGGAGCGGAAAGTGGAGCGACAAGTCAGATTTGTGGAACAAAGTAAGGCCAGATGTTCGAAAAAAGTGTTTCCGTGATGATGGAGAGTTC
TGGATGGAACTGGAGGACTTCTTTCGCTATTTCAGTGCCGTATTCATCTGCTGTGAGACCCCTAACTTTCTTGATTGTCAGTGGAAATGCATGATTTACGAT---------
TCCACCTTTGCAACAAACCCTCAGTATCGCATCCAGGTG------
AAAAACATCTTGCTCTCCCTGATGCAGAACTCCATTGGACTTACCATCTATGAGGTGGTTGAACTCCTAAGTCTGGAACCTGGAGAGTATGTGATCATCCCGACGAACAAACCCAACGTCACTGCAGACTTTGTTCTTACCGTCT
ACACCAAAAATGATGAGCTAATTCCTAGACAACTCCAGAAGCTCCTGAATGACAGGTTCCCTCATGGCTTTAGTTTGGATACCTGCAGGAGTATGATTGCCTTGGTGGATCTCGACCAAAGGATGACGATGACCTTTGCTGAATT
CTCAATTCTCTGGAAGAAGATTCAAGAATACAAGAATCTCTTCCATCGCTGTGATGTGAATGAAAATGGATCCCTGTCTAGCCCTGAGCTCCAGAAGGCGATGGAA------------
GGAATGGATGTGAATGATGGCATTGTGGGGCTGATGATGTTTCGGTATTCAGGC---TCTTTGGAGGAGTTCATCACCCTCATGTTGCGCCTGGATAAGATGTCAGACATTTTCAAGGGCAAATCATCTGATGGCGTA---
ATCCACCTGAACTGGGAT------

AGTCAGGACTACCAGGCCCTCCTGGAGAAGAGCTTAGGGGGGAAACAGTTGTTTGTGGACGAGACTTTCCCGGCCAGTTTGGCCTCCATTGGGTGCCTGCTGGACAAGCTCCCAGACTGCGTGGAGTGGAAAAGGCCTCCCCAAT
TCTGTACGGAAAATGCCAGTGAATTTGGGCTCCACCAAGGCCTCACAGAGAACTGCTGGTTCCTGGCCGCCCTCTCCTCCCTGACGTTCCATCCTGACATCCTGACCAACGTGGTGCCGCAGAACCAGAGCTTCAACAGTTACGG
GGGCATCTTCCACTTTCGGTTCTGGCGTTTCGGGGAATGGGTCGACGTTGTGGTGGATGACCGACTTCCCGTGGAAGGAAAACTCCTGTTCGTCTCGTCGGTCCGAAAAAACCTCTTTTGGGGCCCGCTCCTGGAGAAAGCCTAC
GCCAAACTGTGCGGCTCCTACGAGGACATGCAGATTGGCCAAGTGTCCGAGGCCCTGGTGGACTTCACCGGTGGGGTAAATATGAGCATCAAGATGGCGCAGGCGCTGTTCCAGATCATGATGAGAGCTCAGAACAGCGGATCCC
TAATGGGCTGCGTTCTGGACAACGGGCTGGTTACAGGCCACGCCTACACGGTGATGGACGTCAGGCAGGTGACAGTCAAATCTGTGACGGAGAACTTGGTGAAACTGAGAAATCCCTGGGGAAAGATCGAATGGACGGGAAAATG
GAGCGACAGGTGCCCCAAATGGGAGCAGCTCAGCTACAAGGAGAGGCTTTTCCTCAGAAGAGAAGATGGAGAATTTTGGATGTCCATCGAAGACTTTGAAGCCCACTTTGTAGAACTGGTGATCTGCAAACTGACCCCAGACCTA
ATGAGCAAAGAGTGGACTTTATCCATGCAGTGCGGCAGATGGAAGTCCTATTGGCTGAACCCACAGTACCGCCTCAAGATTCTCAAATGCAACGTTCTGGTGTCCCTTATGAGCCTGTACATTGGCATGTCTGTATTCGAGATTA
CCGATAAGTTT---------
CCAGTACAAACAACAGTATTTCCCACTCCAGAGGCCCATCAGTACTCCCTGTACCCCTTCAGGATATATTCCTGCCACCCGGAAATCAAAGTCGCCCAACTGCAGCAGCTGTTGAACAAAATCACATGGACAAAATTCAGTTTTG
ACGACTGCAAACTCATAATGGCCCTATTGGATATGAAAACATCTGGAACCCTAAACATGCAAGAGTTCCGAGGTCTGTGGAAGCGACTTCTCTCTTATCAAGAGATTTTCCAAAGGAGGGACGTGGATAAAACCGGATATTTAAA
GCTTAATGACCTGCAGGCTGCCATTAAA------------
GGAATTACGTTGCACCGTCAGTTCTACAACCTCGTGGCTCTGCGATACGGTGACATGAGTTTTGAGAATTTCGCCTGTCTTATGCTGAGGATAGAGATTAATGATGAAGTTTTTCACAACCTGAGCCAAGATGGGAAAGGATTT
ATCTCCAGGAGGCC------

CAGCAGAACTACCAAGCCTTGCTGGAGATGTGCTTGAAGAATAAACAGCTGTTCACTGATGAGAGTTTTCCTGCTGACATCAGTTCTATTGGAGCAATACTGAAGAAACTCCCCCGAAATGTGCAGTGGAAGAGGCCACCAGTAT
TTTATGCTGCAAACAGAAAGCAACTTGACCTCTGCCAAGGATTAGTGGGGAACTGCTGGTTCCTGGCGGCTCTGCAAGCCCTGACATTCCACCAGGACATCTTAGCTGCAGTCGTGCCACAAAACCAGAGCTTTGAGAAATATGC
TGGCATTTTCCACTTCCGGTTCTGGCACTTTGGTGAATGGGTTGACGTGGTGGTTGATGATCGCCTGCCGGTGGCGGGGGAGCTGGTCTTTGTTTCTTCAGTCTATAAGAATGTGTTCTGGGGAGCCCTTTTGGAGAAGGCATAT
GCTAAACTCTATGGCTCCTATGAAGATCTACAGATTGGGCAAGTTTCAGAAGCCTTGGTGGATTTCACAGGTGGTGTTAATATAAGAATCAAGCTTCCAGCAGCTCTGTGGGATATCCTGACAAGAGCAACCTACAGCAGATCTC
TCATGTGCTGTGTCTTGAAGAATGGACTTGTTGCTGGCCATGCTTACACAGTAACTGGTATTAGAAAGGTGACCTGCCAATATGGACCAGAAAACCTAGTGAGACTGAGAAATCCATGGGGTAAAATTGAATGGAACGGAGACTG
GAGTGACAGCTCTTACAAGTGGGAGTTGCTGAGCCCAAAGGAGAAAATTCTGCTGAGGAAGGAAGATGGAGAGTTCTGGATGTCTCTGCAAGATTTTAAGATTCATTTTGTAGACGTTGTGATCTGTAAATTAACTCCAGACCTG
GTGAGCAAAAAGTGGATGTATTCCCTGAAGAATGGGAGATGGGGCATGTTTTGGATGAACCCCCAGTACTGGTTGAATATTTTAGCATGCAGTGTGGTTATATCCCTGATGAGCCTCTTCATTGGCTTTTCACTCTACGAAGTGA
CTTGTGACTTCCACCTGGAGCCTGGTGTCTATGTGATTGTGCCAACTCTGGAGCCTCATCAGGAGTCTGAATTCATTCTGCGTGTCTTTTCCAGGAATCCTGAAATAAATGCTGTTCAGCTCCAGAGGATCCTGAATAATGTATC
TTGGAGAAGTTTCAGTCTGGACGCCTGCCAGAGTATCCTGGCACTCCTGGATCTGAATGCCTCTGGCACACTGAGTATCCAAGAGTTCCGAGTCCTGTGGAAAAGGCTGCTTTTCTACTTGGAGGTTTTCCAGAAAAGAGACACT
AGCAGATCAGGAAAGCTTGACCTGGTAGAACTGCGTGCAGCTGTACAG------------
GGTATTTCACTCAGCAATGAAGTCTGTAATTTGATGGCGATCAGATATGGTGACATCAGCTTTGAGAGCTTCATGTGTTTCATGCTTCGAGTAGAGATCATGGGAGAGGCCTTTCGCAACTTAACGCAAGATGGTAAAGGGATAT
ACCTGCAGGAATCTTATTCC

CAGCAGAACTACCAGGCCTTGCTGGAGATGTGCTTGAGGAATAAACAACTGTTCAGTGATGAGAGTTTCCCTGCTGACATCAGCTCTATTGGAGCAATACTGAAAAAACTCCCTCGAAATGTACAGTGGAAGAGGCCACCAGTAT

TTTATGCTGCAAACAGAAAGCAACTTGACCTCTGCCAAGGATTAGTGGGGAACTGCTGGTTCCTGGCTGCTCTGCAAGCCTTAACATTCCACCAGGACATCTTAGCTGCAGTCGTGCCACAAAACCAGAGCTTTGAGAAATATGC
TGGCATTTTCCACTTCCGGTTCTGGCACTTTGGTGAATGGGTTGACGTGGTGGTTGATGATCGCCTGCCGGTGGCGGGGGAGCTGGTCTTTGTTTCTTCAGTCTATAAGAATGTGTTCTGGGGAGCCCTTTTGGAGAAGGCGTAT
GCTAAACTTTACGGCTCCTATGAAGATCTACAGATTGGGCAAGTTTCAGAAGCCTTGGTGGATTTCACAGGTGGTGTTAATATAAGGATCAAGCTTCCAGCAGCTCTGTGGGATATCCTGACAAGAGCAACCTACAGCAGATCTC
TCATGTGCTGTGTCTTGAAAAATGGACTTGTTGCTGGCCATGCTTACACAGTAACTGGTATTAGAAAGGTGACCTGCCAATATGGACCAGAAAACCTAGTGAGACTGAGAAATCCATGGGGGAAAATTGAATGGAATGGAGACTG
GAGTGACAGCTCTTACAAGTGGGAGTTGCTGAGCCCAAAGGAGAAAATTTTGCTGAGGAAGGAAGATGGAGAGTTCTGGATGTCTCTGCAAGATTTTAAGATTCATTTTGTAGACGTTGTGATCTGTAAATTAACTCCAGACCTG
GTGAGCAAAAAGTGGATGTTTTCACTGAAGAATGGGAGATGGGGCATGTTTTGGATGAACCCCCAGTACTGGTTGAATGTTTTAGCCTGCAATGTGGTTATATCCCTGATGAGCCTCTTCATTGGCTTTTCACTCTACGAAGTGA
CTTGTGACTTCCACCTGGAGCCTGGTGTCTATGTGATTGTGCCAACTCTGGAGCCTCAGCAGGAGTCTGAATTCATTCTACGTGTCTTTTCCAGGAATCCTGAAATAAATGCTGTTCAGCTCCAGAGGATCCTGAATAATGTATC
TTGGAGAAATTTCAGTCTGGACGCCTGCCAGAATATCCTGGCACTCCTGGACCTGAATGCCACTGGCACACTGAGTATCCAGGAGTTCCGAGTCCTGTGGAAAAGGCTGCTTTTCTACCTGGAGGTTTTCCAGAAAAGAGACACT
AACAGATCAGGAAAACTAGACCTGGTAGAACTGCGTGCAGCTGTACAG------------
GGCATTTCACTCAGCAATGAAGTCTGTAATTTGATGGCGATCAGATATGGTGACATCAGCTTTGAGAGCTTCATGTGTTTCATGCTTCGAGTAGAGATCATGGGAGAGGCCTTTCGCAACTTAACACAAGATGGTAAAGGGATAT
ACCTACGGGAATCTTTT---

TATCAGAACTACGAGACCTTGCTGGAGACATGCTTGAAGAATAAATGCCTGTTCAAGGATGAGAACTTCCCAGCTGACCTTAGCTCTATTGGGCCACTTCTACAGAAACTACCCCCAAAGCTACAGTGGAAGAGGCCACCAGTAT
TTTATCCTGCAAACACAAGACAGCTTGATCTCTGTCAAGGATTGCTAGAAAACTGCTGGTTTCTGGCCGCCTTGCAAGCGCTGACATTCCACCGAGACATCTTGACTGTGATTGTGCCTCAGTACCAAAGCTTTGATAAGTACGC
TGGCATTTTCCATTTCCGGTTTTGGCACTTTGGCGAATGGGTCGATGTGGTAGTAGACGACCGACTGCCTGTCTCGGGTCAGCTGATTTTTCTGTCCTCCGTTTGTAAGAATATTTTCTGGGGAGCCCTTTTGGAGAAGGCATAT
GCCAAGTTGTATGGCTCATATGAAGATCTGCAGATTGGGCATGTGTCAGAAGCACTGGTGGATTTTACAGGCGGAGTTAATATGACTATAAAACTGGCCGAGGCTCTGTGGAATATATTGACAAGAGCAGCCTACAGCAGATCTC
TCATGGGATGCGTCCTAAAGAATGGGCTGGTGGCAGGCCACGCCTACACGATCACTGGTATTAGAAAGGTGACTTGCAAATATGGACCGGAATATTTGGTGAGACTGAGGAATCCATGGGGGAAAATTGAATGGAAAGGAGCCTG
GAGCCATAGCTCTGGAAACTGGGAGCTGCTCAGCCTAAAGGAGAAGATTTTGCTGCGGCAGGATGATGGAGAATTCTGGATGTCTCTGCAAGATTTTAAGACCCACTTTGAAGATCTGATCATCTGTAAATTATCTCCCGACCTG
ATGAGCAAGAAGTGGATGTATTCTCTGCAGACTGGGAGCTGGGACACATTTTGGATGAACCCCCAGTACTGGCTGGATGTTTACCAGTGCAGCATGCTGGTGTCTCTGATCAGACTCCCCATAGGGTTGTCCATCTTCGAAGTGA
CCCAGGATTTCCGGCTGCAGCCTGGCAGCTACGTCATTGTCCCCACTGCAGAACCCCAACAGGAGTCCGAGTTTGTCCTGAGGGTCTTCTCCAGGCATCCTGAAATAAATGCTGTCCAACTCCAGAGGATCCTGAATAACATGGT
CTGGACAAGATTTAGTCTGGATGCCTGCCAAAGTATCCTGGCGGTGCTGGATCTTAACACCACCGGCACACTGAGTATTCAGGAATTCAGGCTCCTGTGGAAGCGGCTGCTTTTCTATATGGAAGTTTTCCTGAAGAAAGATGTT
GCTAGATCAGGAAATCTGAACTTAATGGAATTGCATGCAGCTGTGCAG------------
GGTAAGCAGTTGAGCAACCAAGTCTGTAACCTGATGGTGATCAAATATGGCAAAATCAGCTTTGAGAGCTTCTTCTGCTTCATGCTGCGCGTGGAGATCATGGGAAAAAGCAAGAGGAACCCCACT---
GACACCAATGGAGTGAAT------------------

CAACAGGACTTTGAGGCCCTGCTGGCAGAGTGCCTGAGGAATGGCTGCCTCTTTGAAGACACCAGCTTCCCGGCCACCCTGAGCTCCATCGGCTCCCTGCTGCAGAAGCTGCCACCCCGCCTGCAGTGGAAGAGGCCCCCCCAGT
TTTATTTTGCCAAGGCCAAAAGGCTGGATCTGTGCCAGGGGATAGTAGGAGACTGCTGGTTCTTGGCTGCTTTGCAAGCTCTGGCCTTGCACCAGGACATCCTGAGCCGGGTTGTTCCCCTGAATCAGAGTTTCACTAAGTATGC
TGGCATCTTCCGGTTCTGGTTCTGGCACTATGGGAACTGGGTTCCTGTGGTGATCGATGACCGTCTGCCTGTGGCTGGCCAGCTGGTCTTTGTCTCCTCCACCTATAAGAACTTGTTCTGGGGAGCACTTCTGGAAAAGGCCTAT
GCCAAGCTCTCTGGTTCCTATGAAGACTTGCAGTCAGGACAGGTGTCTGAAGCCCTTGTAGACTTCACTGGAGGGGTGACAATGACCATCAACCTGGCAGAAGCCCTCTGGGACATCCTCATCGAAGCCACCTACAACAGAACCC
TCATTGGCTGCATTCTGGAGAATGGGCTGGTGGAAGGCCATGCCTATACTCTCACAGGAATCAGGAAGGTGACCTGCAAACATAGACCTGAATATCTCGTCAAGCTACGGAACCCCTGGGGAAAGGTGGAATGGAAAGGAGACTG
GAGTGACAGTTCAAGTAAATGGGAGCTGCTGAGCCCCAAGGAGAAGATTCTGCTTCTGGACAATGACGGAGAATTCTGGATGACGCTGCAGGACTTTAAAACACATTTCGTGCTCCTGGTTATCTGTAAACTGACCCCAGGCCTG
TTGAGCCAGAAGTGGACGTACACCATGCGGGAGGGGAGATGGGACACATTTTGGAAGAACCCGCAGTTCCTGCTGTCTGTCTGGAGGTGCAGCGTGCTGGTGTCCCTGCTCAGGCTCGCCATTGGCTTCTACCTGTATGAAGTGA
GTCAGGAGCTGTGTCTGGAACCAGGGACGTACCTCATCGTGCCCATATTGGAGGCCCACCAGAAGTCAGAGTTCGTCCTCAGGGTCTTCTCCAGGCATCCAGAGATTAATGCAGTTCAACTTCAGAACCTCCTGAACCAGATGAC
CTGGTCATTCTTTAGCCTGGAAGCCTGCCAGGGGATCCTGGCCTTACTGGACCTTAATGCATCAGGTACTATGAGCATCCAGGAATTCAGGGACCTGTGGAAGCAGCTGAAGCTCTCTCAGAAGGTTTTCCACAAGCAAGACCGT
GGG---TCAGGATACCTGAACTGGGAGCAGCTGCACGCTGCCATGAGG------------
GGAATCATGCTCAGTGATGACGTCTGTCAGCTGATGCTCATCCGCTACGGCGGCATGGACTTTGTCAGTTTCATCCACTTGATGCTGCGTGTAGAGAACATGGAGGATGTCTTCCAAAACTTAACCCAAGATGGCAAAGGGATAT
ACCTCCAGAAGCCATACTCC

AAGCAGGACTACAAGGCCCTGAGGGAAAGGTGCCTGATGGAAGGCCGCCTCTTTGAAGATGACAGCTTCCCGGCCAGCCTGTGCTCTGTGGGGCCCCTATTGCGGAAGCTGCCGCCCGGCCTGCAGTGGAGGAGGCCTCCCCAGT
TCTATTCTGCTGACACCAAAAGGCTGGACCTGTGCCAGGGAGTGGTAGGTGACTGCTGGTTCTTGGCTGCTTTGCAAGCTCTGACTTTGCATCAGGACATCCTGAGCCGTGTCGTTCCCCTGAATCAGAATTTCACTAACTATGC
CGGCATTTTCCAGTTCTGGTTCTGGCACTTTGGGAAGTGGGTTCCTGTGGTGATAGATGACCGACTTCCTGTGGCTGGCCAGCTGGTCTTTGTCTCTTCCACTTACAAAAACGTGTTCTGGGGAGCTCTTCTGGAAAAGGCCTAT
GCTAAGCTGTCTGGCTCCTATGAAGACTTGCAGCGTGGACAGGTGTCTGAAGCACTTGTGGACTTCACTGGAGGGGTGACAATGACCATCAAGCCGGCAGAGGCCCTCTGGGACATCCTTACCTGGGCCACCTACAGCAGAACCC
TCATTGGCTGCGTGCTGGAGAACGGGCTGGTGGGCGGCCATGCCTATACCCTCACAGGAATCAGAAAGGTGACCTGCAGACACGGACCAGAATATCTAGTCAAGCTGCGGAACCCCTGGGGCAAAGTGGAATGGAAAGGGGACTG
GAGTGACAGCTCGGGTATGTGGGAGCTACTGAGCCCCAAGGAGAAGATTCTGCTCCTGAGCAATGACGGAGAATTCTGGATGACACTGAAGGACTTTAAAACACATTTCATGCTTCTGGTCATCTGCAAACTGACCCCGGGCCTG
CTGAGTCAGAAGTGGACATACACCATGCGGGAAGGAAGATGGGACACATTCTGGAAGAACCCGCAGTTCCTGCTGTCTGTCTGGAGCTGCAGCGTGCTGGTGTCCCTGCTGAGGCTGGCCATTGGCTTCTACCTCTTTGAAGTGA
GTCAGGAACTGCAACTGGAGCCAGGGACATACCTCGTTGTGCCCACATCCAAGGCTGGCCAGGAAGCAGAGTTTGTCCTGAGAGTATTCTCCAGGTGTCCAGAAATAAACGCAGTGCAGCTGCAGAAGGTCCTGAACCACATGAC

CTGGTCATTCTTCAGCCTGGATGCCTGCCAGGGGATCCTGGCCCTACTGGATCTAAATGCGTCGGGTACCGTGAGCATCCAGGAGTTCAGGGACCTGTGGAAGCAGCTGATGCTGTGTCAGGAGGTTTTCCACAAGCACGACAGC
AGTCACTCAGGATACCTGAACAGGACCCAGCTGCAGGCTGCTATGAGG------------
GGAATCATGCTCAGCAATGACGTCTGCCAGCTGATGCTTATCCGCTATGGCGGCATGGACTTCGGCAGCTTCGTCCACTTGATGCTGCGTGCAGAGTACATGGAGGATGCCTTTCAGAACTTAACCCAAGATGGCAAAGGGATCT
ACCTCCAGAAGCCATACTCC

AAACAGGACTACGAGGCCCTGCAGGAAAGGTGCCTGAGGGACGGCCGCCTCTTTGAAGACAGCAGCTTCCCGGCCACCCTGAGCTCCATCGGGTCTCTGCTGCAGAAGCTGCCGCACCGCCTGCAGTGGAGGAGGCCTGCTCAGT
TCTATTGTGCCAAGGCCAAAAGGCTGGATCTGTGCCAGGGAGTTGTAGGCGACTGCTGGTTCTTGGCTGCTTTGCAAGCTCTGACTTTGCACCAGGACATCCTGAAGCGGGTGGTTCCCCTGAATCAGAGTTTCACTAAGTACGC
TGGCATATTCCAGTTCTGGTTCTGGCACTTTGGAAAATGGGTTCCTGTGGTGATAGATGACCGACTGCCTGTGGCTGGCCAACTGGTCTTTGTGTCTTCTACCTACAAGAACTTGTTCTGGGGAGCTCTTCTGGAAAAGGCCTAT
GCTAAGCTCTCTGGCTCCTATGAAGATTTGCAGCTTGGACAAGTGTCCGAAGCCCTTGTGGACTTCACCGGAGGGGTGACAGTGACCATCAACCTGGCAGAAGCACTCTGGGACATCCTAACCCAAGCCGCCTATAACAGAACCC
TCATTGGCTGCGTGCTGGAGAACGGGTTGGTGGGCGGCCATGCCTATACTCTCACAGGAATCAGGGAGGTGACCTGCAAGCATGGACCTGAATATTTAGTCAAGCTGCGGAACCCCTGGGGGAAGGTGGAATGGAAAGGAGCCTG
GAGTGACAGCTCAAGTACATGGGAGCTACTGAGCCCCACAGAGAAGATTCTGCTGCTGGATGATGATGGAGAATTCTGGATGACACTGAAGGACTTTAAAGCACATTTCATGGCTCTGGTCATCTGTAAACTGACCCCAGGCCTG
CTGAGTCAGAAGTGGGCGTACACCATGAGGGAGGGAAGATGGGACACATTTTGGAGGAACCCGCAGTTCCTGCTGTCCGTCTGGCGGTGCAGCGTGCTGGTGTCCCTGCTCAGGCTCGCCATTGGCTTCTATCTCTTTGAAGTGA
GTCGGGAGCTGTGGCTGGAGCCAGGGATGTACCTCGTTGTGCCCACGTCTGAGGCCCACCAGGAGTCGGAGTTCGTCCTCAGAGTCTTCTCCAGGCACCCAGAAATAAATGCAGCTCAACTGCAGAAGATCCTGAACCGTGTTAC
CTGGTCACTCTTCAGCCTCGACGCCTGCCAGGGGATCCTGGCCCTACTGGACCTTAACGAGTCAGGTACCATGAACATCCAGGAATTCAGGGACCTGCGGAAGCAGCTGACTCTCTACCAGGAAGTTTTCCACAAGCAAGACAGT
AACCGGTCAGGATGTCTGGACTGGGCCCAGCTGCGGGCTGCCATGAGG------------
GGAATCGTGCTCAGTGATGACGTCTGCCAGCTGTTGCTTATCCGCTACGGTGACATGGACTTTGTCAGCTTTGTCCACTTGATGCTGCGTGCGGAGAAAATGGAGGATGTCTTCCAAAACTTAACCCAAGATGGCAAAGGGATAT
ACCTCCAGAAGCCAAGCAGC

AGACAGGACTATGAGGCCCTGAAGGAAAGGTGCCTGAGGGACGGCTGTCTCTTTGAGGACAAGAGCTTCCCGGCCACCCTGAGCTCCATCNNTGGG------------------CCCCGG---
CAGTGGAGGCGGCCTCCCCTTTTCTATTCTGCCAAGGCCAAACGGCTGTATCTGTGCCAGGGATTGGTAGGTGACTGCTGGTTCTTGGCAGCTTTGCAAGCTCTGAGTTTGCACCAGGACATCCTGAGTCGTGTTGTTCCCCTGA
ATCAGAGTTTTACGAAGTATGCTGGCATCTTCCAGTTCTGGTTCTGGCACTTTGGAAAGTGGGTTCCGGTTGTGGTGGATGACCGACTGCCTGTGGCTGGCCAGCTGGTCTTTGTCTCTTCCACCTACAAGAACTTGTTCTGGGG
AGCTCTTCTGGAAAAGGCCTATGCTAAGCTCTCTGGCTCCTATGAAGACTTGCAGTGTGGACAGGTGTCTGAAGCCCTTGTGGACTTCACTGGAGGGGTGACAATGACCATCAACCTGGCAGAAGCCCTCTGGGATGTCCTAACC
CGAGCCATCTACAGCAGAACCCTGATTGGCTGCGTGCTGGAGAATGGGTTGGTGGATGGCCATGCCTACACACTCACAGGCATCAGGAAGGTGACCACCAGCCATGGACCTGAATATCTCGTCAGGCTCCGGAACCCCTGGGGGA
AGATAGAGTGGAGAGGAGACTGGAGTGACAGCTCGAGTATGTGGGATCTACTGAGCCCCAAGGAGAAGATTCTGCTGCTGGATAATGATGGAGAATTCTGGATGTCACTGCGGGACTTTAAAGCACATTTCATGCTTCTGGTCAT
CTGTAAACTGAGCCCAGGCCTGCTGACTCAGAAGTGGTCGTACACCATGCTGGAGGGGAGATGGGACACATTTTGGAAGAACCCGCAGTTCCTGCTGTCCATCTGGAGGTGCAGTGTGCTGGTGTCCCTGCTCAGACTCGCCATT
GGCTTCTACCTCTTCGAAGTGAGCCAGGAGCTGTGGCTGGAGCCAGGGACGTACGTGATCGTGCCCACATCTAAGGCCCGTCAGGAGTCTGAGTTCGTCCTCAGAGTCTTCTCCAGGTATCCAGAAATAAATGCGATCCAACTGC
AGAATATCCTGAACCACATGCCCTGGTCACTCTTCAGCCTTGAGGCCTGTCAGGGTATCCTGGCCCTGCTGGATCTTAACGCATCCGGCACTGTGAGTATCCAGGAATTCAGGGACCTGTGGAAGCAGCTGATGTTCTACCAGGA
GGTTTTCCGGAAGCCTAACACAGGGGAGCTAGGTGTGAGGTCCCAGCACCCACGGCGGGAGCCTGTGTTC------------
GGAATCGTGCTCAGTGATGGCGTCTGCCAGCTGATGCTTATCCGCTACGGCGGCATGAACTTCGTCAGCTTTGTCCACTTGATGCTGCGTGTGGAGAACATGGAGGATGTCTTCCAAAACTTGACCCAAGACGGCAAAGGGATAT
ACCTCCAGAAGCCATACTGC

AAACAGGACTACGAGGCCCTGCAGGAAAGATGCCTGATAGACGGCTGCCTCTTTGAGGACCAGAGCTTCCCGGCCACCCTGCGCTCCATCGGGCCCCTGCTGCGGAAGCTGCCGGAGCGTGTGTACTGGAGGCGGCCTCCCCAGT
TCTATTCCGCCAAGGCCAAAAGGCTGGATCTGTGCCAGGGATTGGTAGGTGACTGCTGGTTCTTGGCGGCTTTGCAAGCTCTGACTTTGCACCAGGATATCCTGAGTCGCGTTGTTCCCCTGAATCAGAGTTTCACTAAGTATGC
TGGCATCTTCCGGTTCTGGTTCTGGCACTTTGGGAAGTGGATTCCGGTGGTGGTAGATGACCGACTGCCCGTGGCTGGCCAGCTGGTCTTTGTCTCTTCCACCTACAAGAACTTGTTCTGGGGAGCTCTTCTGGAAAAGGCCTAT
GCTAAGCTCTCTGGCTCCTATGAAGACTTACAGCGCGGACAGGTATCTGAAGCCCTTGTGGACTTCACTGGAGGGGTGACAATGACCATCAACCTTGTGGAAGCCCTCTGGGACATTCTAACCCGAGCCATCTACAGCAGGACCC
TGATTGGCTGCGTGCTGGAGAATGGGCTGGTGGACGGCCATGCCTACACTCTGACAGGCATCAGGAAGGTGACCTCCAAACATGGACCTGAGTATCTAGTCAAGCTGAGGAACCCCTGGGGGAAGGTGGAGTGGAAAGGTGACTG
GAGTGACAGCTCAAGTACATGGGAACTACTGAGCCCCAAGGAGAGGATTCTGCTGCTAGAGAATGATGGCGAATTCTGGATGACACTGCGGGACTTTAAAACACATTTCACACTTCTAGTCATCTGTAAACTGAGCCCAGGCCTG
CTGAGTCAGACCTGGTTGTACACCATGCTGGAGGGGAGATGGGACATGTTTTGGAAGAACCCACAGTTCCTGCTGTCGGTCTGGAGGTGCAGTGTGCTTGTATCCCTGCTCCGGCTTGCCATTGGCTTCTACCTCTTCGAAGTGA
GTCGGGAGCTGTGGCTGGAGCCAGGCACGTACCTCATCGTGCCCACGTCTGAGCCCCGCCAGGAGTCTGAGTTCGTCCTCAGAGTCTTCTCCAGGTATCCAGAAATAAATGCGATTCAACTGCAGAAGATCCTGAACCGCATGCC
CTGGTCACTCTTCAGCCTCGAAGCCTGCCAGGGGATCCTGGCCCTACTGGATCTTAATGCATCTGGCACCGTGAGTATCCAGGAATTCAGGCACCTGTGGAAACAGCTGATGTTCTATCAGAAGGTTTTCCATAAGCAAGACACT
CACCGGTCAGGATCCCTGAACTGGGCCCAGCTGCAAGCTGCCATGAGG------------GCA---------GCAGACTCA---------------------------------------------------------------
GAGCTGAGAGAGCAGGACATC---------------AATGTGGCCATTGACACAGGA---------------CAGCCC------

CACCAAGACTATGAGACCTTGTTGGAAACATGCCTGAGGAACAATTGCCTCTTTGAGGATCCCAACTTTCCAGCTGACATGAATTCCATTGGGTCCCTGCTGCAGAAGCTGCCCCCAACCCTGCACTGGAAGAGACCTCCTGTAT
TCTATTCAACCAAAGCAGAAAGGCTGGGCCTGTGCCAGGGAGTAGTAGGAAACTGCTGGTTCTTGGCTGCACTGGAAGCTCTTACATTCCATGGGGACATTCTAAACCGGGTTGTACCTCTGAACCAGAGTTTCACCAGGTATGC
TGGCATCTTCCTCTTTCGGTTCTGGCATTTTGGTGAATGGATTCCTGTCGTAATTGATGACCGTCTACCTGTAGCTGGTCACCTGGTCTTTGTCTCTTCTACCTGCAAAAACTTGTTCTGGGCAGCTCTACTGGAGAAGGCCTAT

```
GCCAAGCTCTCAGGTTCCTATGAAGACCTGCAGATTGGGCATGTTTCAGAAGCTTTTGTGGACTTCACAGGAGGCGTGACAGCTACAATTCAACTATCAAAGGCCCTCTGGGACATCCTCACCAGAGTTGCTTCCAAGAGATCCC
TCATTGGCTGTATTCTGAATAATGGACTGGTGGATGGTCATGCATATACTGTCACTGGAATCAGGAAGGTGACTTGTCATAATAGGCCTGAATACCTGGTAAGGCTGAGAAACCCTTGGGGAAAGGTTGAATGGAAAGGAGACTG
GAGTGACAGGTCAAAAAAATGGGATCTCCTAAATCCAAAGGAGAAGATTCTGCTTCTGGATGAGGATGGAGAGTTCTGGATGTCCTTCCATGACTTTAAATTCCATTTTGTGTCTCTGGTCATCTGTCAGCTGACACCAGACCTG
ATGAGTAAGAAGTGGATGTGCTCTCTGAGTACTGGGAGATGGGAAACATTTTGGATGAATCCACAATTTCAACTTACTGTCCTAAGTTGCAGTGTCTTGGTTTCGCTTATGAAGCTCCCCATTGGCATCTTTCTTTTCGAAGTGA
GCTATGAGTTTTCCCTGGAGCCAGACACTTACCTCATTGTTCCCACTTCTGAGCCTTGGCAGGAATCTGAATTCATCCTAAGAGTCTTTTCCAGGTGCCCAGAAATAAATGCAATTCAATTGCAGAAGATTCTGAATAACATGAC
CTGGAAGACCTTCAGTATGGATGCTTGCCTAGGGATTCTGGCGCTGCTTGATTTTAATGCCACAGGGACTGTGGGCATTCAAGAATTCAGGTACCTGTGGAAGCGACTATTGATCTATCAGGAAGTTTTCCACAAACAAGACAAA
CACAAGTCAGGATTTCTGGAATCAGGGCAACTGCGGGCTGCTGCTCAG---------
GAAGGAATTTTACTCAGTGATGAGGTGTGCAATCTGATGGCTATTCGCTATGGCAACATTGGCTTTGAGAACTTTGTCTACTTCATGCTCCGAGCGGAGAATGTGGAAGGTGCCTTTCAAAACGTGACCCAGGATAACAAAGGAA
TATATCTTCTGAAGTCATACTGC

 CTACAGAATTATCAGACTTTGCTGGAAGATTGCAGGAAGAAGGGCTACCTCTTTGAGGATGAGCTCTTCCCAGCTGATCTTAGCTCCATTAAT---
ATACTCTCGAAACTACCTGCCAAAATACAGTGGAAGAGGCCTCCTGTGTTTTTTTCTTCAAAGAAAATAAGTTGTATGCTGTGCCAAGGAGAGTTAGATGATTGCTGGTTTTTGGCTGCTGTGGAAGCTTTAACTTTTCATCAAG
ATATCTTGTTTAAGGTAGTGCCACAGAACCAAAGCTTTGAGAAATATGCTGGGATATTCCATTTCAAGTTCTGGCATTTTGGTGAATGGGTTGATGTGGTAGTAGACGATCGACTGCCTGTATTTGGTCAATTCATTTTCCTCTC
TTGTATTTACAAAAACATATTCTGGGGAGCTCTTCTGGAGAAGGCATATGCCAAGTTGTGTGGTTCCTATGGTGATCTGCAAATTGGGTATGTGTCTGAAGCTCTGGTGGATTTCACGGGTGGAGTGAACATCACTATAAAGCTG
GCTGAAGCTCTCTGGAATATCTTGACTCGAGCAATCTACAGCAGATCTTTTATAGGCTGCGTCCTAGAGAATGGATTAGCAACCTGCCATGCCTACACAATGACTGCCATCCAAAAAGTGACCTGCAAACATGGAACTGAAAATT
TAGTAAGACTGAGAAATCCCTGGGGAAAAACCGAATGGAAAGGTGACTGGAGTGACAAGTCTGAGAAATGGGAACTGCTCAATCCGAAAGAGAAGATTTTGTTGCGATTCGAAGATGGTGAATTCTGGATGTCTCTGCAAGATTT
TCAAATTCATTTTGTTGACCTGATAATCTGTAAATTAACCCCTGATCTCATGAGCAAGAAGTGGATGTGCTCCATGCAGTTTGGCAAATGGAATACATTTTGGATGAACCCTCAATACCTGCTAAAAGTCCTGCGACACAGTGTG
TTGATATCTTTGATCAACCTTCCCATAGGACTCACAGTCTACGAAGTGACTCAGGACTTCCGGCTCCCTCCTGGCATCTATGTCATTGTTCCCACTGCAGAACCTCATCAGGAATCAGTGTTTCTTTTGCGGGTCTTCTCCAGGC
ATCGTCATGTTAAC-----------------------------------------
AGATTTAGCTTGGATGTCTGCCGGAGTATCCTGGCCCTCCTGGATTTTAATAGCACTGGAGACTTGAGCATTCAAGAATTCAGACTCCTATGGAAGAAGCTGCTACTTTATCAGGAAGTTTTCCAGAAAAAAGATATTCACCATA
CAGGATGCTTGGAACAAGCAGAGGTACAAGCAGCTTTAGAA------------
GGCATTCCCATCAGCAGTGAGCTTTGTAAGATGATGGCATATCGCTATGGGGACGTCACCTTTGAAAACTTTGCGTGCTTTGTGCTGCGTGTGGAACTCATGATGAAGGCCTTTCATAATATGAGTAAAGATGGCAAAGGGATAT
ATATCAAGGAATCTTACTCA;

END;



BEGIN TREES;

       TREE                                        tree                                        =
((((((XENOPUS_CAPN1L_ENSXETG00000022058_ENSXETT00000047750,X_LAEVIS_CAPN13_NM_001093584),(X_LAEVIS_CAPN13_NM_001086019,XENOPUS_CAPN13_ENSXETG0000
0022056_ENSXETT00000047741)),((OPOSSUM_CAPN13_ENSMODG00000015519_ENSMODT00000030511,(((HORSE_CAPN13_XM_001500391,CAT_CAPN13_XM_003984358),PIG_CAP
N13_ENSSSCG00000020986_ENSSSCT00000031346),(MOUSE_CAPN13_ENSMUSG00000043705_ENSMUST00000095208,HUMAN_CAPN13_NM_14457))),(ANOLE_CAPN13_ENSACAG0000
0007376_ENSACAT00000007402,(TURTLE_CAPN13_ENSPSIG00000005500_ENSPSIT00000005985,(TURKEY_CAPN13_ENSMGAG00000005407_ENSMGAT00000006081,CHICKEN_CAPN
13_ENSGALG00000020084_ENSGALT00000014759))))),COELOCANTH_CAPN13_ENSLACG00000011037_ENSLACT00000012619),((((TURKEY_CAPN14_ENSMGAG00000005468_ENSMG
AT00000006245,CHICKEN_CAPN14_ENSGALG00000009069_ENSGALT00000014761),(TURTLE_CAPN14_ENSPSIG00000017963_ENSPSIT00000020410,ANOLE_CAPN14_ENSACAG0000
0007149_ENSACAT00000007269)),(TASMANIAN_DEVIL_CAPN14_ENSSHAG00000012346_ENSSHAT00000014582,(((CAT_CAPN14_XM_003984351,RHINO_CAPN14_XM_004418593),
(PIG_CAPN14_XM_003125263,SHEEP_CAPN14_XM_004005801)),HUMAN_CAPN14_NM_001145122))),XENOPUS_CAPN14_ENSXETG00000033183_ENSXETT00000062505)),(((((((
TILAPIA_CAPN14_ENSONIG00000008269_ENSONIT00000010414,MEDAKA_CAPN14_ENSORLG00000019002_ENSORLT00000023709),PLATYFISH_CAPN14_ENSXMAG00000000188_ENS
XMAT00000000185),PLATYFISH_CAPN14_ENSXMAG00000000132_ENSXMAT00000000184),(STICKLEBACK_CAPN14_ENSGACG00000005683_ENSGACT00000007524,PLATYFISH_CAPN
14_ENSXMAG00000000065_ENSXMAT00000000126)),COD_CAPN14_ENSGMOG00000009688_ENSGMOT00000010648),((((TILAPIA_CAPN14_ENSONIG00000005524_ENSONIT0000000
6948,TILAPIA_CAPN14_ENSONIG00000005520_ENSONIT00000006943),TILAPIA_CAPN14_ENSONIG00000005532_ENSONIT00000006957),STICKLEBACK_CAPN14_ENSGACG000000
20515_ENSGACT00000027179),FUGU_CAPN14_ENSTRUG00000000168_ENSTRUT00000000342)),ZEBRAFISH_CAPN14_ENSDARG00000008553_ENSDART00000122453),ZEBRAFISH_C
APN14_ENSDARG00000052917_ENSDART00000074854));

END;
```

(*i*) CAPN17

```
#NEXUS

BEGIN TAXA;

	DIMENSIONS NTAX = 9;

	TAXLABELS

		'XENOPUS_CAPN12_ENSXETG00000034127_ENSXETT00000065487'	'COD_CAPN12_ENSGMOG00000007927_ENSGMOT00000008723'
'FUGU_CAPN12_ENSTRUG00000009564_ENSTRUT00000024129'	'MEDAKA_CAPN12_ENSORLG00000000963_ENSORLT00000001176'
'STICKLEBACK_CAPN12_ENSGACG00000013147_ENSGACT00000017419'	'TILAPIA_CAPN12_ENSONIG00000001284_ENSONIT00000001620'
'ZEBRAFISH_CAPN12_ENSDARG00000055094_ENSDART00000131180' 'GAR_GENSCAN00000011023_LG2' 'PLATYFISH_CAPN12_ENSXMAG00000003111_ENSXMAT00000003132' ;

END;

BEGIN CHARACTERS;

	DIMENSIONS NCHAR = 1251;

	FORMAT

		DATATYPE = DNA


		GAP=-

		MISSING=?

		NOLABELS
	;


MATRIX

 ------------------------------------------------
TGGAGGAGACCACAGGAGATATGTGCTAAGCCAAGGTTTATTTTAGATGGAGCAACAAGAATGGATGTCTGCCAGGGAAAGCTGAGTAATTGCTGGTTTCTGTCAGCTGTTGCGTGTCTGTCTCTATATCCTCAACTTCTGGAGA
AGGTAGTCCTCCCTGGGCAGGACTTTGTAGGTGCTTATGATGGAAAGTTCAGATTCCAGTTTCCTGGCCAAGGAGCCGCCGTGGATGGAGATATTCACGGATGCCTCAGGGGCCAGNTAATAAGGACGAAACCAGCTCACAAACC
AGAGGTGTGGCAGAGCTTTGCTGAAATGGTATACCTGAAAGGTGGATACTCTGCTCTTCAACTAGGATTTGCAGGAGAAGCTTTAGTAGATATGACTGGTGGAGTAGCACAGACACATCTTCTACAGCAAGGGGCCCTTACTTGT
TGTGGAAACACAGAGGGTGAACTAGAGATGGCTAACTCCTTGGGAATTCTAAGTCATCACATGTACTCAGTGACTGGGGCTAAACAGGTTCAAGCATTACACGGCTCTGTTTCTTTACTTAGGGTTCGAAATCCATGGGGACACA
CAGAGTGGTCTGGACCTTGGAGAGAT---TCAGAGTGGCTCTCTGTAATAAACATCAAAGAAATAGAGGTTGAAAACCTA------
GATGGGGAGTTTTGGATGGATGTGGAAGATTTCCAAAAGAATTTTCAGGTCCTGGAAATCTGTCACTCCAGTGGTTGCAGCATTATTGTGTCTGTGATGTTGAAGCACAAACGCCTGAAGGCTAAAATGCGAGTAATGTGTGTCT
ACCTCCAGTGCTCCCAGCCCGTGCTGGTTGATAAAAAACGGGAAGCAGTCCTCTCCACAAGCCTGCCACCAGGGAGATATATTATTATCCCATCATTAGAAACAACAAGTGATGAGGGCGAGTTCCTGATCAGAATTCTAACTGA
GAAAGGCAGCAGTGCTAATCCATATGGAAGTCTCATATTCTCTAACTTGCAAAGTAGCAAGGACAGAAACCTTGACAAGGATCAAATTGGAGCAGCTCTACAGTCTGCAGGCCTGATAACAGACAATTTTCTAGTGCGACTGGTC
CAGCTGAGATATGCAGATAAGGATGGATCACTAAGCTACTCTGCATTCATCTGCTGTCTCCTAAAAATAAAAGCTGTAACGGGAGACAATGCTTTGTACAGC
```

GGCCTCTTCATTGACTACACCTTTCCTGTTGGACAAGAGTCCGTTGAATGGAAGCGGCCCATGGAGCTGTGTAAATCACCTCAGTTCATCGTGGACGGAGCCACACGCATGGATGTGTGCCAAGGGAATCTGGGGGACTGCTGGT
TCCTGTCGGCCCTGGCCTCCCTCTCCATGCACCGCTCCCTACTGAAGAGGGTGGTGCCTCCGGGCCAGGGCTTCCAGAACGGATACGATGGCAGCTTCTACTTCAGGTTCTGGCAGTATGGGAAGTGGGAGGAGGTGAGGGTGGA
TGACCTCCTGCCCGGTCAGCTGTACTACCTCCGCTCTCCCAACAGAAACGAGTTCTGGAGCTGTCTGCTGGAGAAGGCCTACCTGAAGGGGGGCTACCAGGCTCTGGAGATGGGCTTCCCCCACGAGGCCATGGNGGACATGACG
GGCGGGGTGACGGAGGTTCACCTGCTGCGCAAGGGGGCCCTCATCAACTGCGCCAACTGTCAGGGCCCTCTGGAACAGAAGAATGAACAGGGGATCCTGTTCAGACATGCCTACTCTCTGACTGCAGTGGAGAGGGTGGAGACGG
CG---
GGCCCCGTGACCCTGGTGCGTCTGCACAACCCCTGGGGCGGCACCGAGTGGAAGGGCCGCTGGAGCGACCTCCCAGAGTGGAACACCGTGAGTGAGGATGAGCAGAGACGTCTGGGCAGAGTCAGCAAAGACGGAGAGTTCTGGA
TGTCGGTGTCGGACTTCCGTAGGAACCTGGAGATCATTGAGCTGTGTCACTCCGCAGGCTGCTCCTTCCTGGTGGCTCTGATGCAGAAGCACCAGAAGCTCCGG------CTGTCTATAGGCATG------
CTGATGGGGCTCGCCCCCGTCCTG---
GTCATGCGGCGGGAGACGGTGTTGCGTGGCGCCCTGGCGCCCGGGCATTACGTCATCATCCCCTATGCCTCCCTACCCAATCAGGAGGGGCGCTTCATCCTCAGGGTCCTGACGGAGAAGGGCAACGAT---
CAGCTATTCTGGACGGATGCATTCCTGATACATGACAAGAATGGCTCTAAGAAACTCGAGTACGTGGAGGTCAGCTCTGCCATGAAGTCCGCAGGCATCAACATGGACAGCTCTGTGATGCAGTTTGTTGGGCTGAGATACACAG
AGCCAGACATGACCGTCAGCTACCCTGGGTTCCTGTACCTCATGATGAAACTGGACAGCATGATC---------------------

GGACTCTTCGTGGACCGACACTTTCCCCTTGGGGAGCTGGAGGTCAAGTGGAAACGACCAAAGGAGCTCTGCTCTTCACCAAACTTCATTGTAAGTGGAGCGACGCGTTTGGACATCCGCCAAGGAGAACTGAATGACTGCTGGC
TGCTCTCTGCTATTGCTTCTCTGTCTCTGCACCCCTCTCTGCTCGAGCGAGTGGTGCCTCTGCAGCAGAGCTTTCAGGATGGCTACAACGGCAGCTTCACCTTCAGATTCTGGATGTACGGTCAGTGGGAGGAGGTGAGGATAGA
TGACTTGTTGCCCAACAGACTGATCTACCTCAGCTCCCCAGACAAGTGTGAGTTCTGGAGCCCCCTCCTGGAGAAGGCCTATCTGAAAGGAGGCTACAGAGCCCTAAATATGGGCTTCCCCCACGAGGCCATGGTGGACATGACG
GGTGGGGTGACTGAAGTCTACCTGCTGTCCAAAGGAGCTCTCATCAACTGTGCTACCAGCCAGGGTCCTTTAGAACAGAAGAATGAGCTGGGGATCATGTTCAGACACGCTTACTCTCTCACAGCAGTTGAGCAGGTTAAGACAG
CACATGGGACTGAATGTTTGGTGCGGCTCCTGAACCCCTGGGGCAACACAGAGTGGGAAGGGGCCTGGAGTGAT---
CCAGAGTGGAACTCTGTGAGCATTCAGGAGCAGCAGAGACTGCAAAGAGTCAGTCGGGACGGGGAGTTCTGGATGTCGGTGTCAGACTTTCGACAGAACTTCGAGATAATGGAGGTGTGCCACACAGCTGGTTGCTCCTTCGTTT
TAGCACTGATGCAGAAACACCAGAGACGCAGCGGCATCCTTTCTATAGCCCTGACGTACCTGAGTAAGCTGCGTCCCGTCCTCAGTTCTTCTCGCCGGGAGGTTGTTCTTCGCAGCTCGCTGCCCCCTGGTCGTTACCTTATTAT
CCCATCTACTTCCGAACCGAACCAACAGGGAGAATTCCTGCTGCGGGTGCTGACAGAGAACGAGAACATTTCCCAGCTTTTCTGGACAGATATCTTCCTGGTGTTTGACAAGAACAAAACGAAGCGTCTTGAGTATCAAGAGGTC
GGCCCCGCTCTCAAGGCAGCAGGCATTGTTGTGGATGATCTGATCATGCAGCTGGTTGGACTGAGGTACACAGAGCCAGACATGACCATCAGCTACCCCGGCTTCCTCTACCTGCTTCTGAAGCTGGAGAACATGATCTAT----
--------------

GGGCTGTTCGTGGACTACCAGTTCCCCGTGGGGGAGCTGGGACTGAAGTGGAAGCGACCGAAGGAGCTTTGTTCCTCGCCGCAGTTCATCATCGACGGAGCGTCACGGCTGGACATCGTTCAGGGAAAACTCAGTGACTGCTGGC
TGCTGGCTGCCATAGCGTCCTTGGCGATGCATCGATCTCTGCTGAAGAAGGTGGTTCCTCTTCATCAGAGCTTTGAGGAGGACTACAACGGCAGCTTCGTCTTCAGGTTCTGGCAGTACGGTCAGTGGGAGGAGGTGAGGATCGA
CGACCTGCTGCCCGACAAGCTGGTCTACCTGAGCTCGCCGGAGAGACGCGAGTTCTGGAGCTCGCTGCTGGAGAAGGCCTACCTGAAAGGAGGCTACAGAGCTCTGGACATGGGCTTCCCTCATGAGGCCATGGTGGACATGACG
GGCGGCGTGACGGAGGTTTACCTGCTGTCCAAAGGAGCCCTCATAAACTGTGCCAACTCCCATGGCGTGACGGAGACCATGAACGACCTGGGTATTCTGTTCAGACACGCCTACGCTCTGACTGCGGTGGAGAAGGTGAAGACCA
CGAATGGCCCCGTGGACTTGGTGCGGATTCTCAATCCCTGGGGTAGAACAGAGTGGAAAGGACCATGGAGCGACTTACCAGAGTGGCAGACGGTGAGTTTGGAGGAGCAGAGGCGCCTGGACCGGGTCAGCCGTGATGGCGAGTT
CTGGATGTCGATCTCGGACTTCCGGCAGAACTTCGAGGTGATGGAAGTGTGTCAC---------
TGCTCTTTTGTCTTGGCTCTGATGCAGAAACACCAGAGGCGCAGGGGCATTCTCTCCATCGGCATGCCATACCTCCGCATGCTCCAGCCGGTCCTC---
TCCTCCCGCCGGGAGGTCGTCCTCCGCGGCTCCCTACCCCCCGGTCGCTACATCATCGTCCCCTCCACCCTGGAGCCCAACCAGGAGGGGGCGTTCCTCCTGCGGGTGCTGACGGAGCGGGGAAACGCCGCCCAGCTGTTTTGGA
CTGACATATTTCTGATGTTTGACAAAAACAAAACAAAACACCTCGAGTATCAGGAGGTCAAACCTGCTCTGATGGAAGCGGGCATCAAGGTGGACGACTTGGTGATGCAGCTGGTCGGACTGCGATACACAGAGCCCGACATGAC
GGTCAGTTTCCCAGGTTTCCTGTACCTGATGATGAAGATGGAGAGCATGATCCATGACAGCACCATGTACAAC

GGGCTGTTCGTGGACCACCACTTCCCCGTGGGGGAGCTGGGGGTCAAGTGGAAACGACCGACGGAGCTCTGTTCGGCCCCCTCAGTTCATCGTGAACGGAGCCACGCGCCTGGACGTCTGCCAGGGAGCGCTCAATGATTGCTGGT
TGCTCTCCGCCATCGCGTCCCTCTCCGTGAACCGCTTCCTGCTCAATAAGGTCATGCCTCTGGAACAGAGCTTCAAAGACGGGTACAAGGGTTGCTTCACCTTCTCGTTCTGGCAGTACGGTCAGTGGGAGGAGGTGAAGATAGA
CGACTTTTTGCCAAACAAACTGATATACCTCAGCTCCCCAGAGAGAGATGAGTTCTGGAGCGCCCTCCTGGAGAAGGCCTACCTTAAAGGCGGCTACAGAGCCCTGGACATGGGCTTCCCTCACGAGGCGATGGTGGATATGACG
GGCGGGGTGACTGAGGTTCACCTGCTGGCTAGAGGAGCGCTCATTAACTGTGCCAACTGCCAGGGTCCTTTGGAGCAAAGTAATGGACTGGGAATCATGTTCAGACATGCCTACTCTCTGACTGCAGTGGAACAGGTGAAGACGG
CACACGGCATCGTGGATTTGGTGCGGATCCTCAACCCTTGGGGCAACACAGAGTGGTCGGGGCCGTGGAGCGACTTGCCAGAGTGGGAGGCGGTTAACAAGGAGGAGCAGAAAAGACTGGAAAGAGTGAAACGTGACGGGGAGTT
CTGGATGTCTGTGTCAGACTTCCGGCGGCACTTCGAGACAATGGAGGTGTGTCACTCAGCAGGATGCTCCTTCGTTTTGGCTCTGATGCAGAAACACCAGAGACGTAGGGGTGTCCTGGCTATAGCCCTGACCTACCTGCAAGCG
TTCCGCCCGGTCCTC---
TCCATGCGGCGGGAGGTGGTCCTCCGCGGCTCCCTCCCACCGGGCCGCTACATCATCGTCCCTTCAACCGCCGAGCCCAATCAGCCGGGAGCCTTCCTCCTCCGGGTGCTGACGGAGCAGGGCAACGCCGCCCAGCTGTTCTGGA
CGGATATCTTCCTGGTGTTTGACAAGAACAAAACAAACCACCTGGAGTACAAAGAGGTGGCCCCCGCTCTGCAGAAAGCAGGCATCATGGTGGACGATTTGATAATGCAGCTGGTCGGACTGCGGTACACGGAGCCAGACCTGAC
CATCAGTTACCCCGGTTTCCTGTACCTGCTGATGAAGCTGGAGAGCATGGCCCACGACAGCACCATGTACAAC

GGGCTGTTCGTGGACTTTCACTTCCCCGTGGGAGAGCTGGGGGTGAAGTGGAAGCGACCAAAGGAACTCTGTCCATCACCTCAGTTCATAGTAGATGGAGCAACGAGGCTGGATGTTCACCAGGGGAAACTCAGTGACTGCTGGT
TACTTTCGGCTATAGCGTCTCTCTCCATGTATCAATCGCTCCTCAAGAAGGTTGTGCCTCTGAGCCAGAGCTTCCAGGACGGATACAATGGTTGCTTCACCTTCAGGTTCTGGCAGTACGGTCAGTGGGAGGACGTACAGATCGA
TGACCTGCTGCCCAACAATCTGATCTTCCTCAGCTCCCCAGAAAAACAAGAGTTCTGGAGCGCCCTGCTGGAGAAAGCCTACTTGAAGGGTGGCTACCGAGCTTTGGACATGGGCTTCCCTCATGAGGCCATGGTGGATATGACG
GGCGGGGTGACGGAGGTTCACCTGCTGGTTAGAGGAGCACTCATCAACTGTGCCAACTGCCAGGGTCCTCTGGAACGGAGGAATGAACTGGGGATCATGTTTAGACATGCCTATGCTCTGACTGCAGTGGAAAAGGTGAGGACGA
CACACGGCACAGAATATTTGGTGCGAATCCTCAATCCTTGGGGCAACACAGAGTGGGAGGGGCCCTGGAGTGACTTCTCAGAGTGGAACACGGTGAGCGTCGAGGAGCAGAAACGACTCGGCAGAGTCAGACAGGACGGAGAGTT
CTGGATGTCGGTGTCAGACTTCCGACAGAACTTCGAGGTGATGGAGGTTTGTCACACAGCAGGATGCTCCTTCGTTTTGGCTCTGATGCAGAAGCACCAGAGACGAAGAGGCATTCTAGCCATCGCCCTTACGTACCTGAGCGCG
AGCCGCCCAGTCCTCAGCGTCACACGGCGGGAGATTGTTCTTCGCGGCTCCCTCCCACCGGGTTCCTACATCATCATCCCATCCACCGCAGAGACCAATCAGCAGGGAGCCTTCCTCCTGCGGGTGCTGATGGAGCAGGGCAACA
ATGCCCAGCTTTTTTGGACGGATATCTTCCTCATGCATGACAAGAACAAAACTCAGCGCCTCGAGTATCAGGAGGTCATGCCCTCGCTGAAAGCAGCAGGCATCAGGGTGGACGAGTTGGTGATGCAGCTGGTTGGACTGAGATA
CACGGAGCCGGACATGACCATCAGTTACCCCGGCTTCCTGTACCTGCTGATGAAACTGGAGAGCATGATCCACGACAGCACCATGTACAAC

GGGCTGTACGTGGACACCAGATTCCCGGTGCAGAACATGGACCTAGTTTGGAGACGACCGAAGGAGATCTGTCTGTTTCCCCAGTTTATTGTGGATGGAGCCACTCGTATGGACATCTGCCAGGGAGTTTTGGGTGATTGCTGGT
TCTTGTCTGCGATGGCGTCTCTTTCTCTCTATCCTGCCCTTCTGGATCGTGTGGTTCCCGCAGGCCAGGGTTTTCAGCAGGGATACAACGGCTGCTTCTGCTTTCAGTTTTGGCAGTACGGCGAGTGGATAAGTGTGAAAATCGA
TGACCTTTTGCCGAATCAGCTGATCTACCTGCACTCCGCCAGCAGAGATGAGTTCTGGAGCGCGCTGCTGGAGAAAGCTTATCTGAAGGGAGGTTACAGTGCTCTGAATATGGGTTTTCCTCACGAGGCAATGGTGGACATGACA
GGTGGGATCACGGAGGTTTCTTTACTGGAGAAAGGAGCTCTGATCAACTGCGCAAACTCACAGGGTCCCCTTGAGAAGAGTAATGAGTTTGGGATTTTGTTCAGACATGCCTATTCTGTGACCGCTCTGGAGACGGTAAAGAGCA
CTATTGGTCCAGTTCAGCTGGTGCGGATCAGGAACCCGTGGGGTAAAGCTGAGTGGGAAGGACCCTGGAGTGACAATATCGAGTGGCATATGGTGACCGCTGAAGAACAGCGGCGTGTGCAGAGAATCCAGCAGGATGGAGAGTT
CTGGATGTCTTTGGCAGATTTCAGACAAAACTTTGAGCTGATGGAGGTTTGTCAC---------TGTTCATTCCTGCTGGCGCTCATGCAGAAACACACTCGGCAGAGAGGAACGCTCTCCATAGCACTG---------------
---
TCTCCGCTGCTCTCCCAGCAGCGGCCGGAGCTGGTGCTGCGCGGTCGCCTGGCACCCGGCCATTACATCATCATTCCTGCCATACAAGAGACCAATCAGAGCGGAGAATTCCTGCTCAGAGTCTTGACTGAGAAAGGAAACACAA
CCGATCTGTTTTGGACGAGTATTTTTATCACGTTTGACAAAAACAAGAACCAGGCGCTGGATTATCTAGAAATCTCCCCCGCTCTTTCAGCAGCAGGCATAAAGGTGGACGAGTTCATCCTGCAGCTGATCAGCCTGCGCTACAC
GGAGCCAGACATGACCCTCAGTTTCCCAGGATTCCTCTTCCTGCTCATGAAACTGGACTGCATGATGCGCGACAACACGTTGTACAAC

GCGCTGTTCGTGGATAAGAGTTTCCCCGTG---------
AAACTAGTGTGGAAAAGACCCAAGGACATCTGTGTGTCTCCGAAGTTCATTGTGGACGGCGCAACAAGGACAGACGTTTGTCAGGGAATACTGAGTGATTGCTGGTTCCTGTCAGCTGTGGCCTCTCTCTCCCAGCACAGAGCCC
TGCTGGAGCGCGTTGTCCCCGAAGGGCAGGGATTTGACCGCGAGTATACCGGCTGCTTCCGCTTCCAGTTCTGGCAGTATGGAGAGTGGAAGGAAGTGGAGGTGGACGACTTCCTGCCCGGCCAGCTGGTGTACCTCCACTCGGC
GGAACGGGACGAGTTCTGGAGTGCGCTGCTGGAGAAGGCCTACCTGAAGGGCGGGTACCAGGCTCTGCAGTTGGGCTTCCCTCACGAGGCCTTAGTGGACATGACGGGGGGCGTGACCGAAATCCCGCTGCTGGAGAAGGGTGCG
CTCATCAACTGCGTCAACACTCAGGGGGAAATGGAGAAGAAGAATGAGCAGGGCATCCTCTTCAAGCATGCCTACTCTGTCACCGGCCTGGAGAAGGTGAGGTGTTACTTGGGGGAGGCTGACCTGGTGCGCGTGAGGAACCCCT
GGGGTCACACAGAGTGGACAGGCCCCTGGAGCGACAACTTGGAATGGGCCAAGGTCAGCGCCGAGGAACAGGCCAGGGTGCAGAGGGTGCAGCAGGATGGGGAGTTCTGGATGGAGGTGTCTGACTTCCAGCGCAATTTCAACAT
GATGGAGGTTTGCCACTCCGCAGGGTGCAGTTTTCTGGTGGCATTGATGCAAAAGCACCAGAGACAGAAAGGAGTTCTCGACATCGGTCTCATGTACCTGGCCAGAGCCCAGCCCCTGATCACGAACCAGCAGAGAGAGGTGGTG
ATTCGCAGTGCCCTGGCACCTGGACATTACATCATCATCCCTTCCACCTCGATGGCCAATCAGGAGGGAGAGTTCGTCCTGCGCGTCTACACTGAGAAGGGCAACAAGGCAGAGCTCTTTTGGACGGATATCTTTGTGCACTTTG
ACAAGAACAGGTCCCAGTCGCTGGATTACCCCGAGATCATCCCTGCCCTGCAGGCTGCAGGGCTGCAGGTTGATGATTTCGTGTTGCAGCTGATCGGCCTGCGATACACTGACCCCGACCTGACCATCAGCTACCCAGGATTCCT
GCACCTCCTGCTTAAACTGGACATCATGATA---------------------

----------------------------------------------
ACTTCCCGACGTCTCCAGGAGCTTTTTCCTTCGCCCAAGTTCATAGTGGACGGAGCTTCACGGCTCGACATCTGCCAGGGGAAACTGAGTGACTGCTGGGTGCTCTCCGCCATAGCATCTCTGGCGAATTACCCAGAACTCCTCA
AGAAGGTCGTTCCTCTCAATCAGAGCTTCCAGGACGGCTACAACGGCAGATTCTCCTTCAGATTCTGGCAGTATGGGCAGTGGGAGGAGGTGAGGATTGACGACTTCCTGCCAAACAAGCTGATCTACCTCAGCTCCCCTGAAAG
ACACGAGTTCTGGAGCTCTCTGCTGGAAAAGGCCTACCTGAAAGGAGGCTACCGAGCGCTGGAACTGGGTTACCCTCATGAGGCCATGGTGGACATGACCGGCGGAGTGACCGAGGTCAACCTGCTGGCCAAAGGAGCGCTCATC
AACTGTGCCAACGGCAAGGGTGAGGTGGAAAAGAAGAACGAGATGGGAATCATGTACCGACATGCCTACTCCCTGACGGCGGTGGAGAAGGTGAAAACAACTGATGGGTTTAAAGATTTGGTGCGAATCCTCAATCCATGGGGCC
ACACGGAGTGGTTAGGGGCCTGGAGCGACAACAACGAGTGGACAAAGGTGAGCGCTGAGGAGCAGAAACGACTGGGACGGGTCCGGCAGGACGGGGAGTTCTGCCTGACTGTTATAGACACGCGCTCAAAAATAGAAACTCTGTT
TGCCTGCCTG---------TGTCTGTTTTACCTTCCAGCTTCTGTTAGTGAAGAAGCAGGAGATGCTGTGTTTGCATCGTCTCTGGCCTTCCTGCGG------
AAAGGCGTCCTCTGGTCGACGCGCCGGGAGGTCGTCCTCCGCAGCTCGCTCCCTCCAGGTCGCTACATCATCATCCCCTCGACCTTTGAACCCAACCAGCAGGGGGAGTTCCTGCTCAGGGTGCTGACGGAGCCGGGCAGCGACG
CCCAGCTTTTATGGACGGATATCTTCCTGGTTTACGACACGAATAAAACTCATCACCTGGAGTACAAAGAGGTCGAACCGGCCCTGAAGGCAGCAGGCATCATGGTGAACGATCTGGTGATGCAGCTGGTGGGACTGAGATACAC
GGAGAGCGACCTGACCATCAGCTACCCCGGCTTCCTGTTCCTGCTCATGAAGATGGAGAGCATGATCCACGACAGCACCTTGTACAGC;

END;

```
BEGIN TREES;

	TREE tree = ((((((FUGU_CAPN12_ENSTRUG00000009564_ENSTRUT00000024129,(TILAPIA_CAPN12_ENSONIG00000001284_ENSONIT00000001620,PLATYFISH_CAPN12_ENSXMAG00000003111_ENSXMAT00000003132)),STICKLEBACK_CAPN12_ENSGACG00000013147_ENSGACT00000017419),MEDAKA_CAPN12_ENSORLG00000000963_ENSORLT00000001176),COD_CAPN12_ENSGMOG00000007927_ENSGMOT00000008723),ZEBRAFISH_CAPN12_ENSDARG00000055094_ENSDART00000131180),GAR_GENSCAN00000011023_LG2,XENOPUS_CAPN12_ENSXETG00000034127_ENSXETT00000065487);

END;
```
